# Flavin Hydroperoxide and Semiquinone: Structures and Biomimetic Activity

**DOI:** 10.1021/jacs.6c11146

**Published:** 2026-08-12

**Authors:** Ümit Beser, Thomas Pickl, Oksana Storcheva, Tim Hieke, Philipp Palmen, Eva Rentschler, Jan Freudenberg, Alexander Pöthig, Golo Storch

**Affiliations:** † Technical University of Munich (TUM), School of Natural Sciences and Catalysis Research Center (CRC), Lichtenbergstr. 4, 85747 Garching, Germany; ‡ Johannes Gutenberg University Mainz (JGU), Department of Chemistry, Duesbergweg 10-14, 55128 Mainz, Germany

## Abstract

Flavins are unique natural redox cofactors that can switch between an oxidized, a radical half-reduced, and a fully reduced state. In nature, the interplay between these redox states is orchestrated by the surrounding flavoenzymes and unlocks a myriad of unique chemical transformations. One of the most archetypical flavin properties is the activation of molecular oxygen from air by the reduced cofactor, which proceeds via the half-reduced semiquinone radical and leads to a flavin hydroperoxide, the key species in flavin-mediated oxygenations. While central to many natural and biomimetic catalytic processes, both flavin semiquinone and hydroperoxide are transient reactive species and, therefore, have so far evaded structural characterization at the molecular level, including single-crystal diffraction. We close this gap and disclose solid-state structures of both isolated key intermediates, linking their reactivity to enzymatic transformations. The biomimetic oxygenation of tetrahydrocarbazoles, which constitute the core structure of many biologically active natural products, was achieved in high yields using a flavin hydroperoxide. Orthogonal activation of the same substrates was achieved by embedding the flavin semiquinone in a catalytic cycle. A total of seven distinct product classes were accessed, all resembling structures occurring in natural products. Our findings clearly link the reactivity of isolated flavin species to well-studied flavoenzymes. We expect these results to promote the application of flavin catalysts as oxygenation agents at both laboratory and industrial scales.

## Graphical abstract

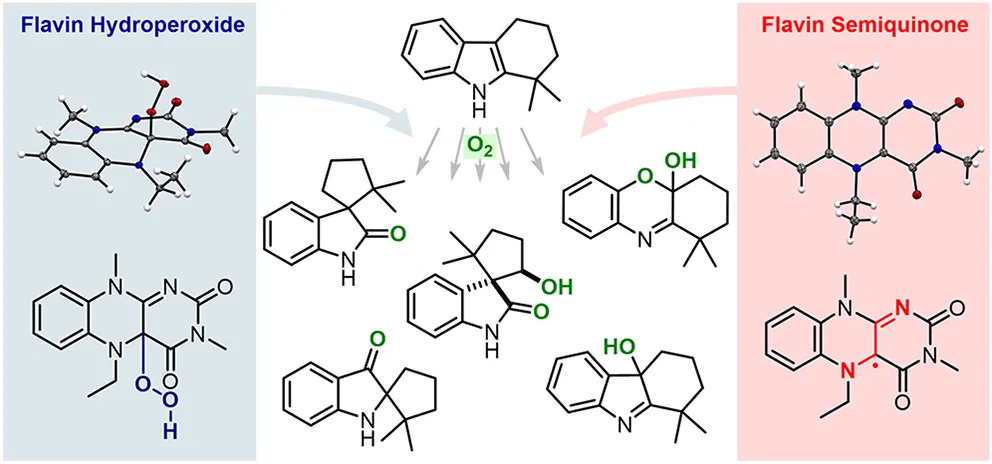

## Introduction

### Key Structures in Nature: Fl_SQ_ and Fl_OOH_

Flavoenzyme-mediated oxygenations are among the most versatile aerobic functionalization strategies in nature.^1,2^ Central to achieving this reactivity is the conversion of molecular oxygen into reactive oxygenating species. The flavin cofactor is predestined for this activity by enabling the reversible formation of reactive hydroperoxides at the C4a and N5 positions.^3–6^ This process involves the initial reduction of the quinoid cofactor **Fl**_**BQ**_ to the hydroquinone **Fl**_**HQ**_ (Figure 1a),^7^ typically by nicotinamide adenine dinucleotide phosphate (NADPH). Subsequent reaction with O_2_ generates a semiquinone **Fl**_**SQ**_ superoxide radical pair, which combines to the hydroperoxide **Fl**_**OOH**_.^8,9^ The latter oxygenates substrates accompanied by the formation of a hemiaminal **Fl**_**OH**_.^10^ The catalytic cycle is closed by water elimination. Owing to their unique activity, the two key catalytic intermediates **Fl**_**SQ**_ and **Fl**_**OOH**_ have long been of interest in chemistry and the life sciences.

**Figure 1:**
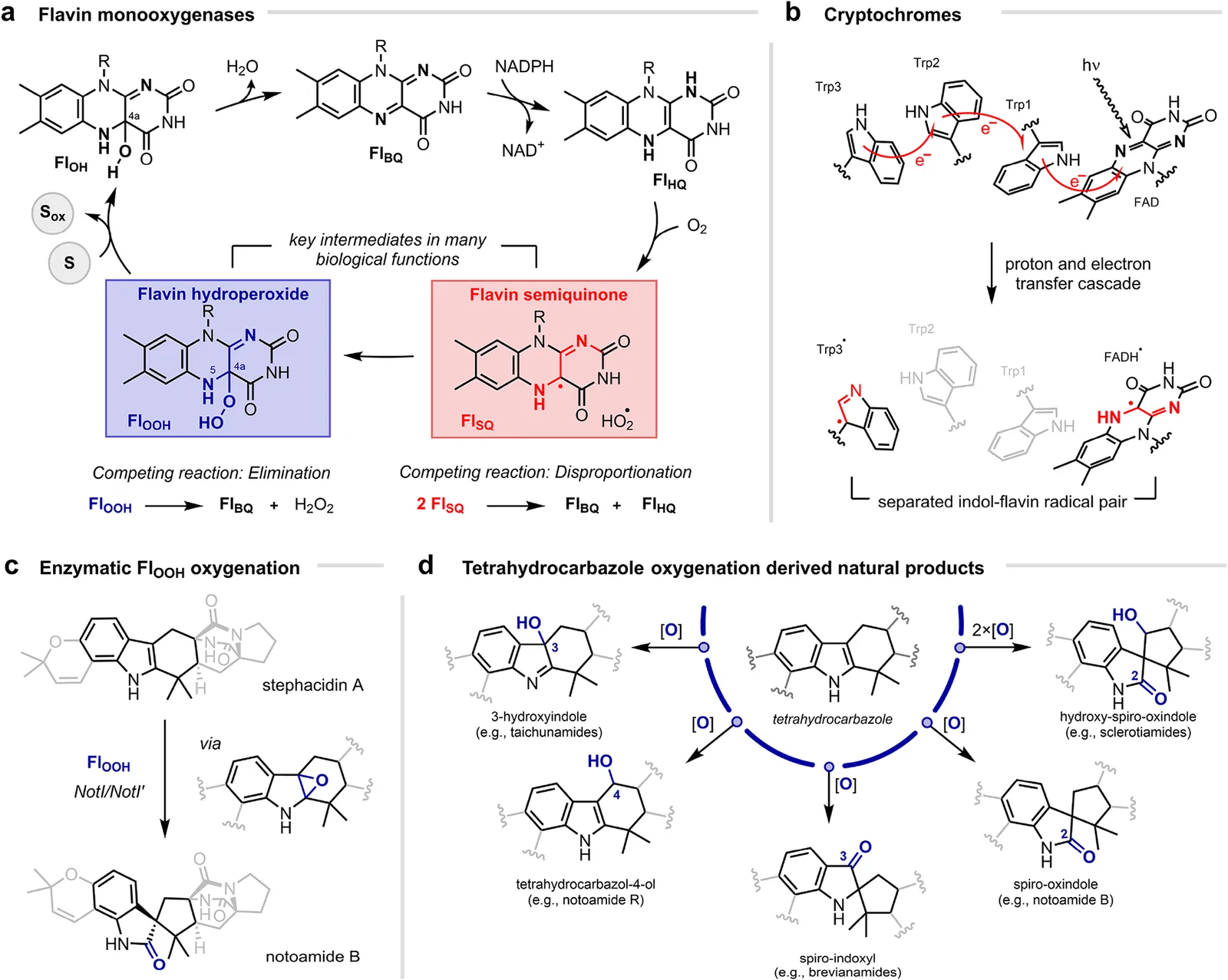
Flavin–indole interactions in nature. (a) General mechanism of aerobic oxygenation in flavin monooxygenases. Schematic illustration of the key intermediates involved in the flavin-dependent conversion of substrates (S) to oxidized products (S_OX_). The substituent R denotes the ribityl–adenine diphosphate moiety of flavin adenine dinucleotide (FAD). (b) Flavin–indole electron-transfer cascade in cryptochromes. Upon FAD photoexcitation, a spatially separated tryptophan (Trp)–flavin (Fl) radical pair is generated, which is assumed to play a key role in magnetoreception of certain birds. (c) The NotI/NotI’-mediated oxygenation-rearrangement of stephacidin A. (d) Diversity of oxygenative reactions of tetrahydrocarbazole-containing natural products.

The radical semiquinone **Fl**_**SQ**_ state of flavin cofactors is essential for many biological functions, including magnetoreception in cryptochromes and light reception of phototropins.^11–15^ On a molecular level, flavin semiquinones are formed by electron transfer from a donor such as tryptophane, which generates the key radical pair after light excitation (Figure 1b). A similar mechanism is at the heart of designed flavoenzymes for quantum spin resonance.^16^ Several flavoenzyme mechanisms also involve **Fl**_**SQ**_ intermediates, crucial for rationalizing their activity.^17–19^ However, unless kinetically stabilized by their enzymes, isolated flavin semiquinones quickly disproportionate in solution. While they can be generated by partial reduction in solution allowing spectroscopic analysis by UV–vis absorption^20–22^ and EPR studies,^23–25^ the instability of **Fl**_**SQ**_ species hampers isolation and comprehensive structural characterization.

Isolated flavin hydroperoxides **Fl**_**OOH**_ are also transient intermediates, since they rapidly decompose by C–O bond heterolysis with rate constants (*k*_obs_) ranging from tens to hundreds s^–1^, as determined by pulse radiolysis under neutral aqueous conditions.^26^ Monooxygenases such as NotI/NotI’ use the increased reactivity^27^ of **Fl**_**OOH**_ compared to alkyl hydroperoxides *inter alia* for the conversion of the tetrahydrocarbazole stephacidin A into (−)-notoamide B (Figure 1c),^28,29^ a member of the fungal bicyclo[2.2.2]­diazaoctane indole alkaloid class with antitumor, antimicrobial, and antiparasitic properties.^30^ The key oxygen transfer step by **Fl**_**OOH**_ is required for several biosynthetic pathways, as a diverse range of rearrangement chemistry is unlocked upon initial oxygenation (Figure 1d). Based on this synthetic relevance, biomimetic indole and tetrahydrocarbazole oxygenations are frequently applied in total synthesis,^31–35^ typically with strong oxidants.^36–40^ While model systems of flavin hydroperoxides have been generated in solution^41–43^ and their decomposition has been studied,^44^ flavin-mediated indole oxygenation and definitive structural proof of **Fl**_**OOH**_, including single-crystal analysis, remain elusive.

## Results and Discussion

### Structure and Reactivity of Fl_OOH_

To solve the missing structural puzzle piece of flavin hydroperoxide **Fl**_**OOH**_, we drew inspiration from an early study by *Bruice* and added H_2_O_2_ to flavinium salt **1**,^45^ which led to the crystallization of greenish crystalline material **2** directly from the reaction mixture (Figure 2a). This nucleophilic addition approach circumvents the intermediacy of **Fl**_**sq**_ and superoxide. The structure of hydroperoxide **2** was unambiguously determined (see Supporting Information) by NMR spectroscopy, high-resolution mass spectrometry, and single-crystal X-ray diffraction (SC-XRD). Successful isolation likely relies on rapid crystallization of the hydroperoxide **2**, effectively protecting it from decomposition in solution: This process is most plausibly driven by dimer formation through two intermolecular hydrogen bonds, where the hydroperoxide group of one flavin connects to both the O2′ (1.89(3) Å) and N1 (2.40(2) Å) positions of a neighboring molecule (see Supporting Information); these dimers form one-dimensional columns along the *b*-axis with extensive π–π stacking among adjacent flavin molecules (distance 3.1 Å). The long O–O bond distance of 1.4665(15) Å observed in **2** (*cf*. 1.453 Å in H_2_O_2_)^46^ confirms a peroxide bound to the sp^3^-hybridized C4a carbon. All bond lengths around C4a are consistent with predominant single-bond character, and the sum of bond angles (∼336°) closely approximates that of an ideal tetrahedron (∼328°).

**Figure 2:**
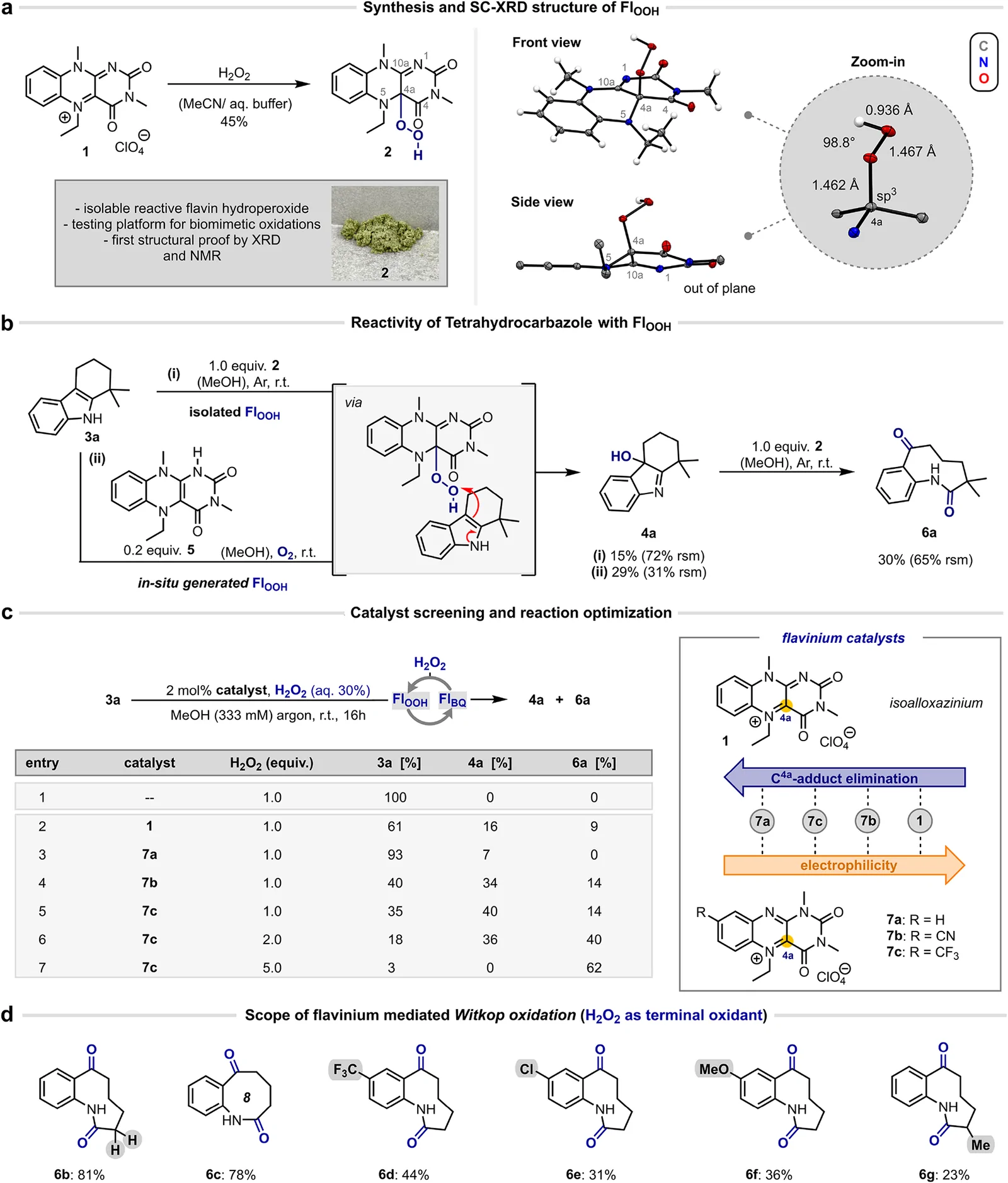
Biomimetic oxygenation reactions mediated by Fl_OOH_. (a) Single crystals of the reactive flavin hydroperoxide **2** obtained from MeCN:aqueous phosphate buffer. (b) Oxygenation activity is confirmed by a biomimetic conversion of tetrahydrocarbazole **3a** into alcohol **4a**. The *in situ* generated **Fl**_**OOH**_ from reduced flavin **5** also mediates the reaction (rsm = remaining starting material). (c) Screening of catalysts and conditions for the catalytic oxygenation with flavinium salts and aqueous H_2_O_2_. (d) Scope of the catalytic reaction. Standard reaction conditions (see Supporting Information): 4 equiv. H_2_O_2_ (aq. 30%), 2 mol % flavin **7c**, (MeOH), r.t., 16 h. Yields refer to isolated material.

This structural evidence enabled us to link the characteristic oxygenating activity of **Fl**_**OOH**_ in flavoenzymes with the isolated hydroperoxide **2** through a biomimetic test reaction.^47^ Similar to the oxygenation mediated by the flavoenzyme NotI/NotI′, tetrahydrocarbazole **3a** was converted into tertiary alcohol **4a** (Figure 2b), confirming the oxygenating activity of the isolated **Fl**_**OOH**_. As expected, substrate oxygenation competes with decomposition of hydroperoxide **2** in solution. Analogous reactivity was observed with flavin hydroquinone **5**,^48^ where the reactive species was generated *in situ* by reduction of O_2_. With excess flavin hydroperoxide **2**, the intermediate is oxidized once again and lactam **6a** is formed.

To establish an efficient synthetic protocol for this biomimetic *Witkop-Winterfeldt* oxygenation, we evaluated a series of flavinium catalysts (**1**, **7a**–**c**) using aqueous H_2_O_2_ for this transformation (Figure 2c). The ideal method only required 2 mol % of alloxazine catalyst **7c**, providing lactam **6a** in 62% yield. Optimal catalyst performance required a careful balance between electrophilicity at the C4a position, required for efficient H_2_O_2_ addition, and feasibility of water elimination from the **Fl**_**OH**_ intermediate.^49–51^ The oxygenation conditions facilitate the formation of an array of lactams (**6b**–**g**), which confirm that altering the ring size and omission of the methyl substituents are tolerated (Figure 2d).

### Structure and Reactivity of Fl_SQ_

We subsequently turned to characterize the flavin semiquinone **Fl**_**SQ**_, the second previously elusive flavin species. Key to its successful characterization was direct contact of hydroquinone **5** with air using methanol as the solvent, which led to the formation of dark crystalline **8** (Figure 3a). To demonstrate the versatility of this approach, we extended the protocol to the oxidation of reduced alloxazine **9** to radical **10**. Both species were characterized by infrared spectroscopy and high-resolution mass spectrometry (see Supporting Information). Analysis of the dark crystalline material provided the first unambiguous structural confirmation of both semiquinone radicals by SC-XRD. Structural analysis revealed considerably elongated C4a–N5 bonds (**8**: 1.385(3) Å; **10**: 1.395(12) Å), consistent with a loss of double-bond character when compared to the fully oxidized isoalloxazinium analogue **1** (1.321(2) Å, see Supporting Information). In both cases, the C4a and N5 centers are completely planar (sum of bond angles ∼360°), in line with sp^2^ hybridization and delocalization of the unpaired electron. Semiquinone **8** displays columnar packing in a head-to-tail fashion with overlapping isolumazine moieties and π-stacking distance of 3.5 Å. This is consistent with the presence of isolated radicals in the solid state rather than pancake bonding.^52^ Radical **10** adopts a similar columnar motif, with slightly shorter π-stacking of 3.4 Å between the alloxazine radicals through oppositely oriented bisimide groups. Prolonged exposure of the crystals to oxygen fully oxidized the material, leading to the dissolution of the crystals under formation of methanol adduct **11**, presumably through addition of methanol to the alloxazinium salt. The entire multistep oxidation process was recorded for the alloxazine semiquinone (Figure 3b, see Supporting Information for details).

**Figure 3:**
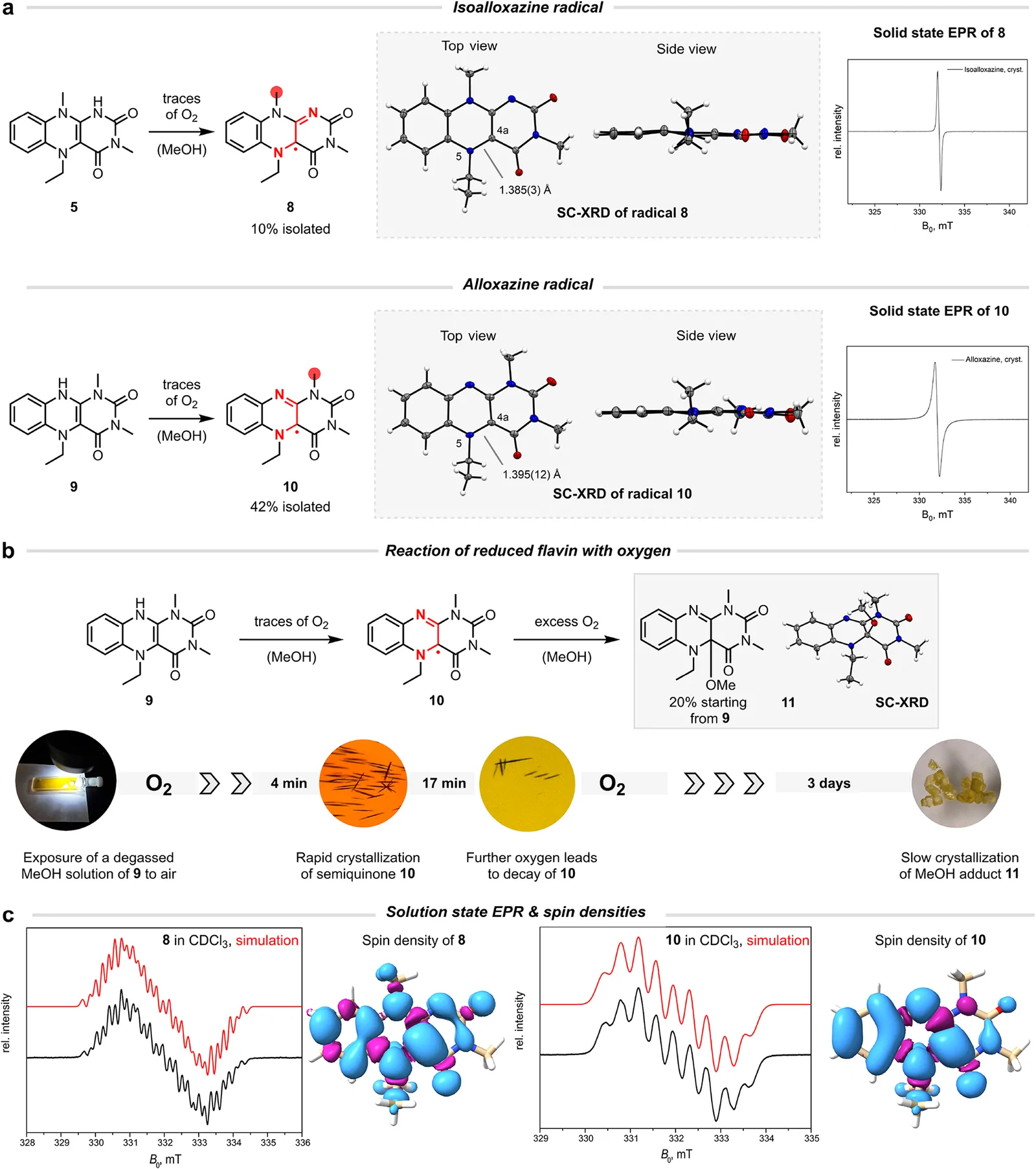
Isolation and characterization of stable flavin semiquinone radicals. (a) Preparation and SC-XRD of isoalloxazine and alloxazine derived semiquinones **8** and **10** (ellipsoids shown at the 30% probability level). The paramagnetic character of the crystalline material was confirmed by solid-state EPR spectroscopy. (b) Exposure of hydroquinone **9** to trace oxygen in methanol leads to the formation of crystalline flavin semiquinone **10** (dark needles). Prolonged exposure of the same solution to air results in the formation of single crystals of the methanol adduct **11**. (c) The open-shell characteristics of **8** and **10** in CHCl_3_ solution were analyzed by EPR spectroscopy (black: measured spectra, red: simulation). Spin density plots (DFT, UB3LYP-D3BJ/def2-TZVP, iso value ±0.0005) localize the unpaired spin predominantly on the reduced quinoxaline core.

The open shell nature of both crystalline **Fl**_**SQ**_ species was also confirmed by EPR spectroscopy, which clearly supports their radical nature. In contrast to the unresolved solid-state spectra of the crystalline material (cf. Figure 3a), both semiquinones in CDCl_3_ solution afforded EPR spectra (**8**: *g* = 2.0039; **10**: *g* = 2.0039) with pronounced hyperfine splitting (Figure 3c). EPR simulations provided a reasonable fit only considering coupling with two chemically nonequivalent nitrogen (A­(^14^N) = 20.41 MHz, A­(^14^N) = 10.84 MHz) and two equivalent hydrogen atoms (A­(^1^H) = 10.15 MHz) in case of alloxazine radical **10**. In contrast, to simulate the more complex spectrum of isoalloxazine **8**, additional couplings with the proximal methyl group and aromatic protons had to be taken into account (see Supporting Information).^53^ In accordance with the experimental EPR spectra, the spin density plot (DFT, see Supporting Information) localizes the unpaired electron predominantly on the reduced quinoxaline cores with significant less spin density on the bisimide, rationalizing the observed coupling to only the two out of four nitrogen atoms. The astonishing stability of **8** and **10** in aerated methanol is attributed to (i) the π-delocalization of the unpaired spin as well as the (ii) captodative effect imposed by the amine and the carbonyl adjacent to C4a.^54^

### Fl_SQ_-Induced Catalytic Activity

The remarkable stability of isolated **Fl**_**SQ**_ prompted us to explore their utility in catalytic transformations. We specifically aimed for using flavinium salts as electron acceptors that facilitate the conversion of substrates into their respective reactive open-shell forms. Related to frustrated radical pairs,^55,56^ our approach would lead to a persistent^57^ **Fl**_**SQ**_ species alongside an activated substrate radical.^58^ This strategy proved successful: Tetrahydrocarbazole **5a** was activated through an orthogonal autoxidation strategy, generating radical **12**. Subsequent reaction with molecular oxygen delivers hydroperoxide **13**, which was converted into hydroxyindolenine **4a** by reduction with P­(OMe)_3_ (Figure 4a). For the reaction to occur, a nitrogen base and the flavin catalyst are required (see Supporting Information). From a synthetic point of view, this method offers a practical route to monooxygenation products that are inaccessible using conventional oxidants such as *m*CPBA, which suffer from overoxidation and unselective competing processes.^59^ A screening of substrates demonstrated tolerance for several substituents at the aromatic positions, though in general, electron donating functionalities led to faster conversion and halogen-bearing substrates required longer reaction times (Figure 4b and Supporting Information).

**Figure 4:**
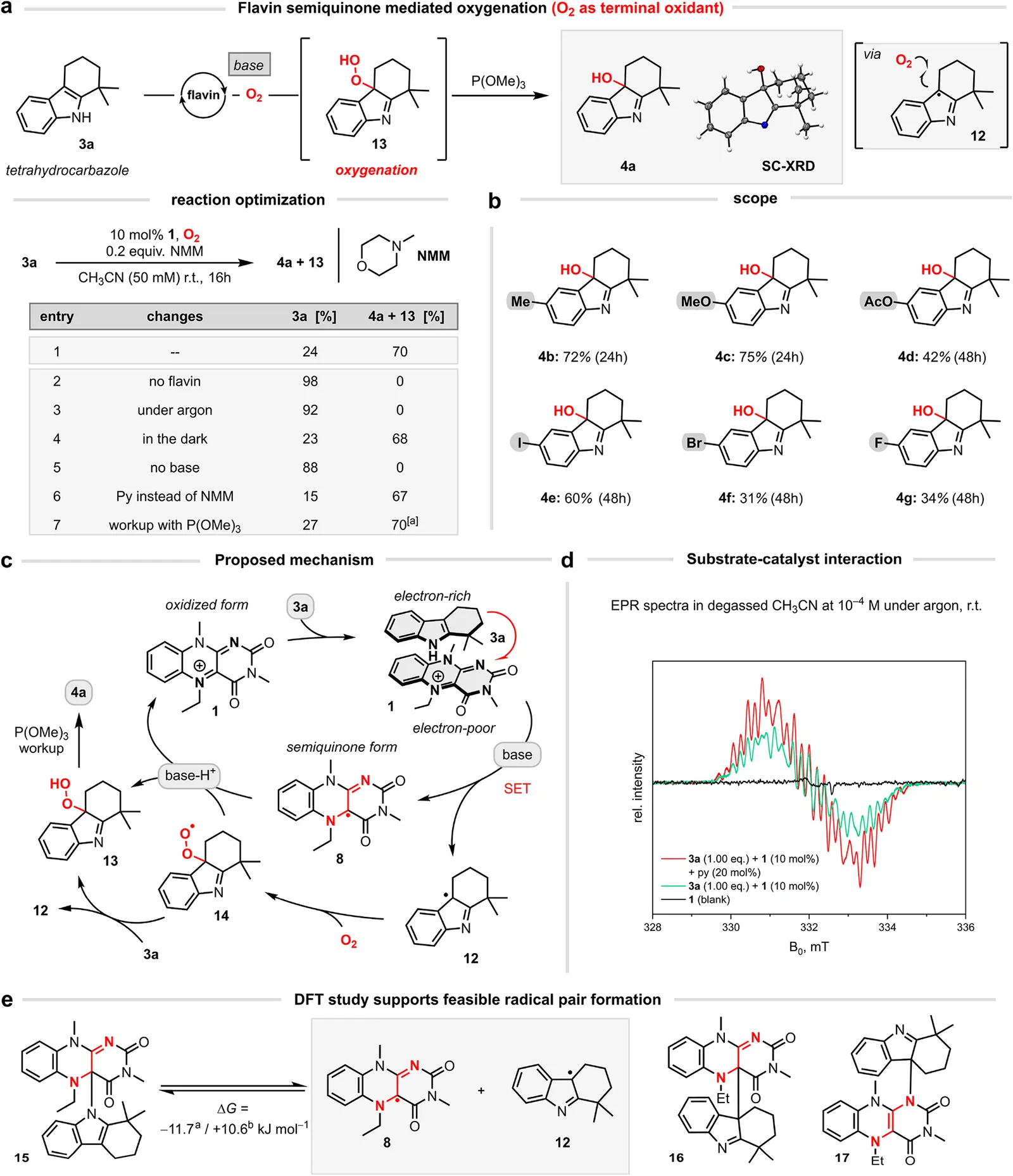
Oxygenation reactions mediated by flavin semiquinones. (a) Catalytic protocol for flavinium-mediated oxygenation of tetrahydrocarbazole **3a** to hydroxyindolenine **4a**. (b) Synthetic application of flavinium-mediated oxygenation of tetrahydrocarbazoles. All yields refer to isolated material. Reaction conditions: 0.2 equiv. NMM, 10 mol % **1**, MeCN (50 mM), r.t., O_2_, 16 h. (c) Proposed mechanism for the oxygenation of tetrahydrocarbazoles, which operates through initial substrate oxidation by flavinium catalyst **1**. (d) Evidence for the ground state interaction of **1** with **3a** observed by EPR. (e) DFT study at the (a) UB3LYP-D3BJ­(PCM:CH_3_CN)/maug-cc-pVTZ and (b) M06–2X­(PCM:CH_3_CN)/maug-cc-pVTZ level of theory. Py = pyridine, NMM = *N*-methylmorpholine.

The proposed mechanism of this reaction relies on an initial ground-state interaction between flavinium **1** and tetrahydrocarbazole **3a**,^60^ which leads to electron transfer in the presence of base and O_2_ (Figure 4c). Once generated, 3*H*-indolyl **12** ^61^ forms peroxyl **14** with O_2_ and is subsequently reduced to hydroperoxide **13** by either **Fl**_**SQ**_ or another substrate molecule.^62,63^ Support for this mechanism arises from EPR spectroscopy, as no electron spin resonance was detected for pure flavinium **1** in acetonitrile solution. However, the addition of tetrahydrocarbazole **3a** led to a radical signature, further strengthened by addition of base (Figure 4d). DFT calculations (Figure 4e) suggest that the frustrated radical pair comprising semiquinone **8** and carbon-centered radical **12** is very similar in Gibbs free energy compared to the corresponding covalent adduct **15**. Hypothetical products of covalent bond formation at alternative flavin positions (**16** and **17**) are even higher in Gibbs free energy (see Supporting Information).

### Unified Access to Natural Products

The oxygenation products of tetrahydrocarbazoles represent an entry point for accessing complex indole alkaloid scaffolds, which parallels the diversity of natural products that arise from this pathway mediated by flavoenzymes. The divergent rearrangement of alcohol **4a** under acidic or basic conditions serves as the archetypical reaction toward bicyclo[2.2.2]­diazaoctane indole alkaloids and illustrates this versatility (Figure 5): Under basic conditions, spiro-indoxyl **18** is selectively obtained, while in the presence of *Lewis* acids, spiro-oxindole **19** forms either from alcohol **4a** or via product **18**.^64,65^ Activation of the hydroxy group with the *Burgess* reagent leads to an unusual carbamate (**20**) of the tetrahydrocarbazol-4a-amine, while exposure to hydrogen peroxide initiates a *Witkop* oxidation to ketolactam **6a**.

**Figure 5:**
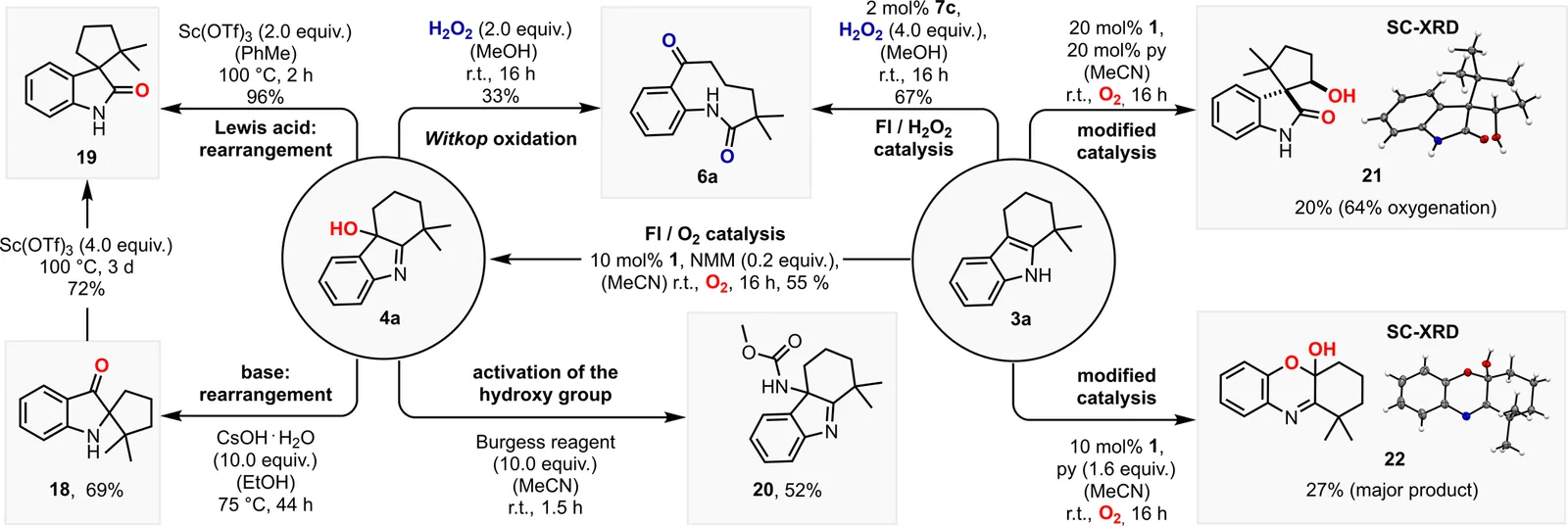
Synthetic diversity of tetrahydrocarbazole oxygenation products. Left, starting from alcohol **4a**: Syntheses of spirocyclic isomers **18** and **19**, *Witkop* oxidation to ketolactam **6a**, and formation of carbamate **20**. Right, starting from tetrahydrocarbazole **3a**: Divergence in product distribution arising from subtle changes to the reaction conditions: Yields refer to isolated material.

Slight variations of our catalytic protocol also revealed previously unexplored reaction pathways. Subtle changes of base and flavinium catalyst loading shifted the product distribution in the aerobic tetrahydrocarbazole oxidation, which led to a considerable amount of spirocyclic alcohol **21**. The new species was isolated as a single diastereomer, assigned with SC-XRD. In analogy to similar steps in the total syntheses of notoamide R and sclerotiamide,^36,66^ the alcohol likely stems from hydrolysis of an intermediate elimination product (see Supporting Information). With excess pyridine base, the oxygenation with flavinium catalyst **1** led to the predominant formation of hemiketal **22** under aerobic conditions. Single crystal analysis confirmed the structure, which is likely formed through aryl migration in hydroperoxide **13**.^67–69^

## Conclusion

We disclose the first complete structural investigation of **Fl**_**OOH**_ and **Fl**_**SQ**_ at the molecular level. Both elusive compounds are crucial intermediates in catalytic cycles of flavoenzymes and in organic catalytic reactions. Our findings link molecular structure to individual reactivities, allowing an unprecedented level of control over the activity in biomimetic oxygenation reactions, which are notoriously difficult to direct. This new level of understanding of reactive flavin species paves the way for a broad application of flavin catalysis in aerobic functionalization, a long-standing goal in sustainable chemistry.

## Supplementary Material



## Data Availability

Research data is published at Gutenberg Open Science, the institutional open access repository of the Johannes Gutenberg University Mainz, under the following DOI: https://doi.org/10.25358/openscience-16066.

## Notes

Experimental procedures and compound characterization, detailed catalysis screening, SC-XRD data, EPR experiments, and additional analytic data (PDF)
