# Supplementary material for: Flavin Hydroperoxide and Semiquinone: Structures and Biomimetic Activity
Source: J Am Chem Soc. 2026 Aug 12;148(33):36142–51. doi: 10.1021/jacs.6c11146 (PMC13523690; doi:10.1021/jacs.6c11146)
Supplement: Supplementary file 1 [file ja6c11146_si_001.pdf]

Flavin Hydroperoxide and Semiquinone: Structures and Biomimetic Activity

Ümit Beser[1], Thomas Pickl[1], Oksana Storcheva[1], Tim Hieke[2], Philipp Palmen[2], Eva Rentschler[2], Jan Freudenberg[2], Alexander Pöthig[1], and Golo Storch[1,2]*

[1] Technical University of Munich (TUM), School of Natural Sciences and Catalysis Research Center (CRC), Lichtenbergstr. 4, 85747 Garching, Germany

[2] Johannes Gutenberg University Mainz (JGU), Department of Chemistry, Duesbergweg 10-14, 55128 Mainz, Germany

* Corresponding author: golo.storch@uni-mainz.de

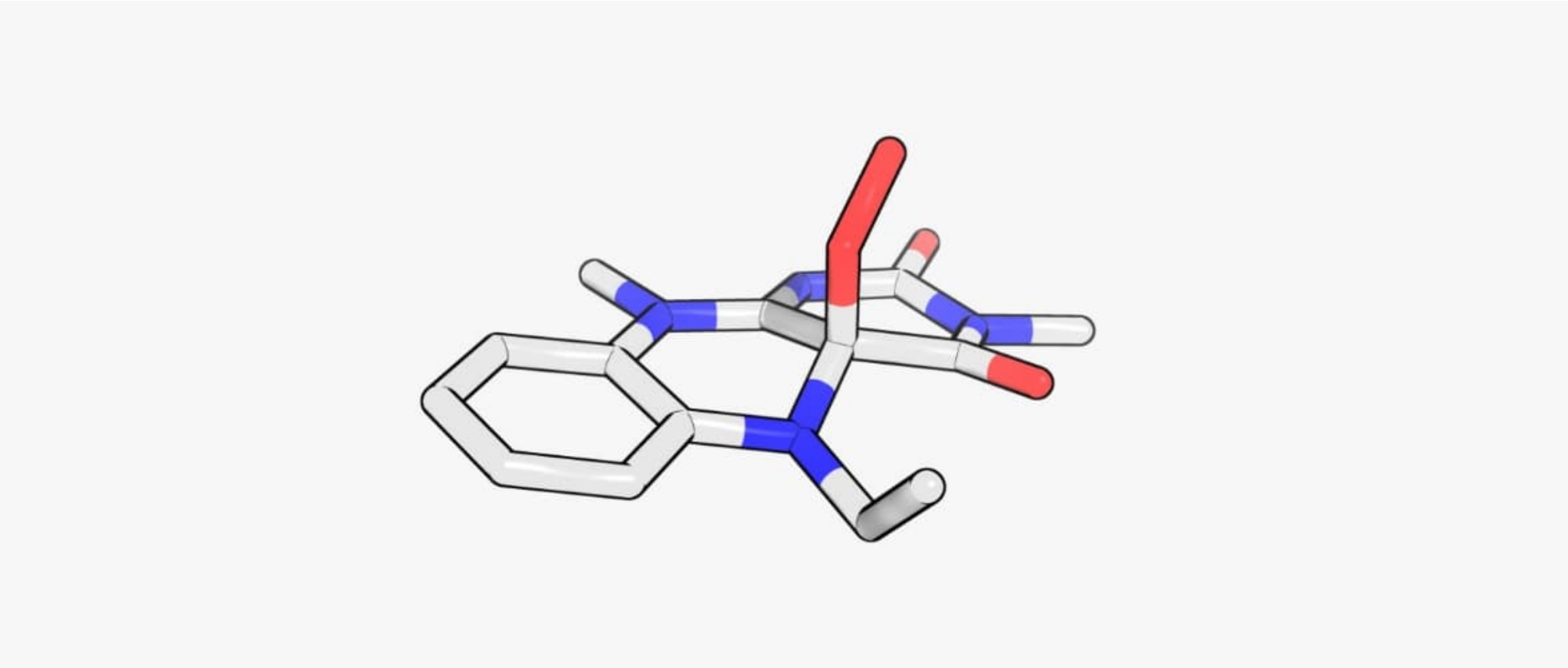

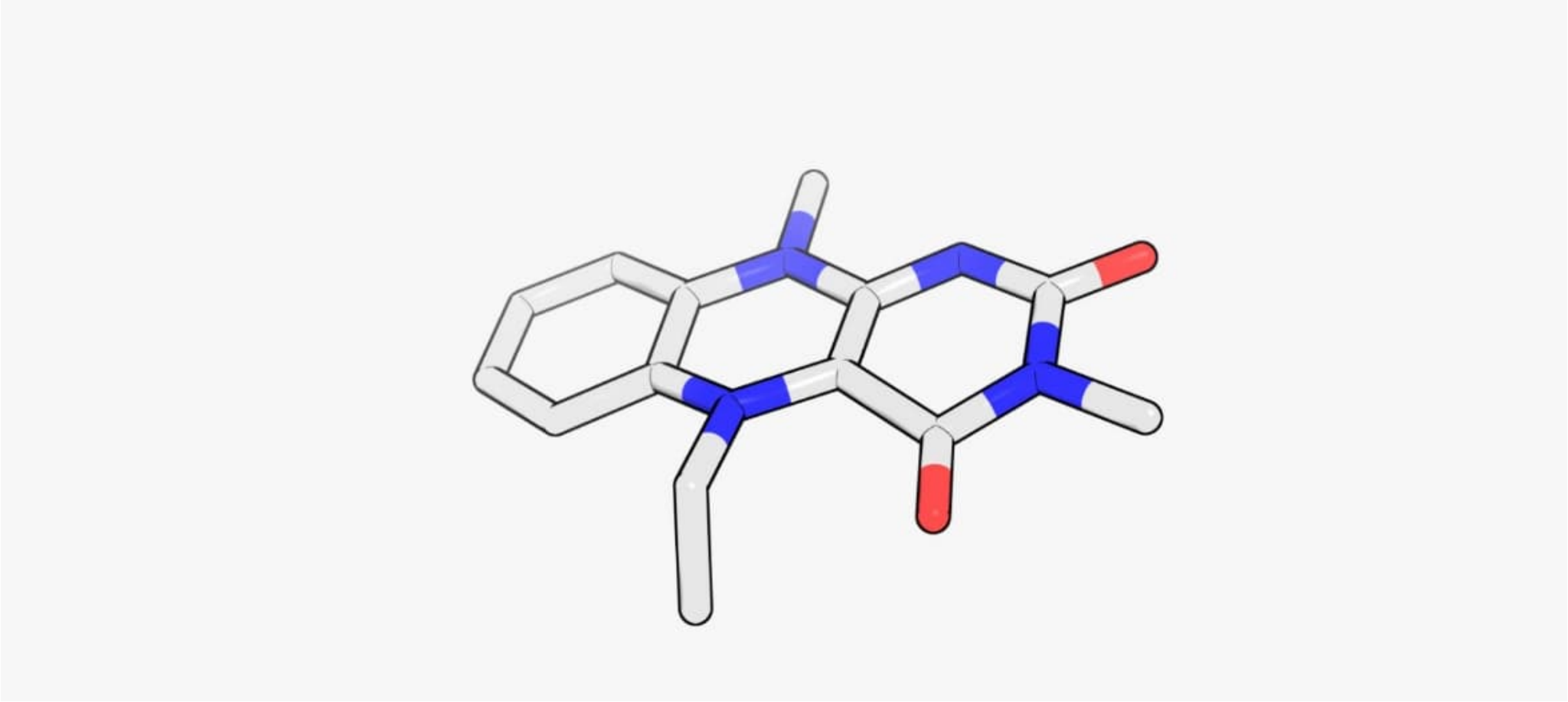

Table of Contents

1. Materials and Methods....................3
2. Synthetic Procedures....................5
3. Reaction Development....................91
4 UV/Vis Studies....................99
5 EPR Measurements....................101
6 Computational Studies....................105
7 Oxidation Experiments....................108
8 X-Ray Crystallographic Data....................116
9 NMR Data....................151
10 References....................219

1. Materials and Methods

1.1. Synthetic Techniques, Solvents, and Chemicals

Room temperature is defined as 21–23°C. All reactions with air-sensitive reactants were carried out under an argon atmosphere (Ar 4.8) applying standard *Schlenk* technique in glassware, which was thoroughly dried with a heat gun under vacuum and conditioned under argon. Unless otherwise noted, all chemicals were obtained from Sigma-Aldrich, Acros, TCI, abcr, Fisher Scientific, or Alfa Aesar and used without further purification. Dichloromethane (2×MB-KOL-A type 2, aluminum oxide), diethyl ether (1×MB-KOL-A type 2, aluminum oxide), and tetrahydrofuran (2×MB-KOL-M type 2, 3 Å molecular sieves) were obtained from an MBSPS 800 MBraun solvent purification system. All other solvents were purchased from commercial suppliers and used without further purification unless otherwise noted.

1.2. NMR Spectroscopy

NMR spectra were recorded on Bruker AVHD-300, AVHD-400, AVHD-500, or AV-III-500 spectrometers at ambient temperature unless otherwise noted. Deuterated NMR solvents were purchased from Deutero GmbH or Sigma-Aldrich and were used without further purification. Spectra were processed with MestReNova 10.0.1 using the manual phasing and polynomial baseline correction capabilities. Splitting was determined using the multiplet analysis function with manual intervention as necessary. Spectral data is reported as follows: Chemical shift (multiplicity [singlet (s), doublet (d), triplet (t), quartet (q), pentet (p), multiplet (m)], coupling constant, integration, assignment). Broad signals are labeled as such (br). Chemical shifts are reported in ppm (δ) and coupling constants are reported in Hz. Resonances are referenced to solvent residual signals.[1] Assignment of all signals was performed by two-dimensional experiments (^{1}H-^{1}H COSY, ^{1}H-^{13}C HSQC-ME, and ^{1}H-^{13}C HMBC). In cases where an unambiguous assignment was not possible, this is indicated by "/" between atom positions in question, while "," is used in cases where resonances of two or more atoms overlap. Apparent multiplets which occur because of accidental equality of coupling constants to those of magnetically non-equivalent protons are marked as virtual (*virt.*). Note: Small deviations in chemical shifts may be observed depending on the concentration of NMR samples.

1.3. Mass Spectrometry

High-resolution mass spectrometry was performed on Thermo Scientific LTQ-FT Ultra (HR-ESI) or DFS GC-MS (HR-EI) instruments.

1.4. Infrared Spectroscopy

Infrared spectra were recorded on a Perkin Elmer Frontier ATR/FT-IR spectrometer, and $\tilde{\nu}_{max}$ are reported in cm^{-1}. The signal intensity is assigned using the following abbreviations: s (strong), m (medium), and w (weak).

1.5. Liquid Chromatography

Analytical HPLC measurements were performed on Thermo Fisher Ultimate 3000 series instruments equipped with a Diode Array Detector and/or MS Detector.

Analytical thin-layer chromatography was performed using 60 Å Silica Gel F254 (Merck) pre-coated glass plates. TLC plates were visualized by irradiation with a UV lamp or staining. Preparative flash column chromatography was performed on silica 60 (Merck, 230-400 mesh).

1.6. EPR

EPR spectra were recorded on a JES-FA 200 spectrometer by JEOL at X-band frequency (9.25 GHz). A Mn^{2+} (nuclear spin I = 5/2) standard embedded in MgO-matrix was used to determine g-values.

1.7. UV/Vis Spectroscopy

UV/Vis spectroscopy was performed on a Perkin Elmer Lambda 365+ UV/Vis spectrometer.

2. Synthetic Procedures

2.1. Substrate Synthesis

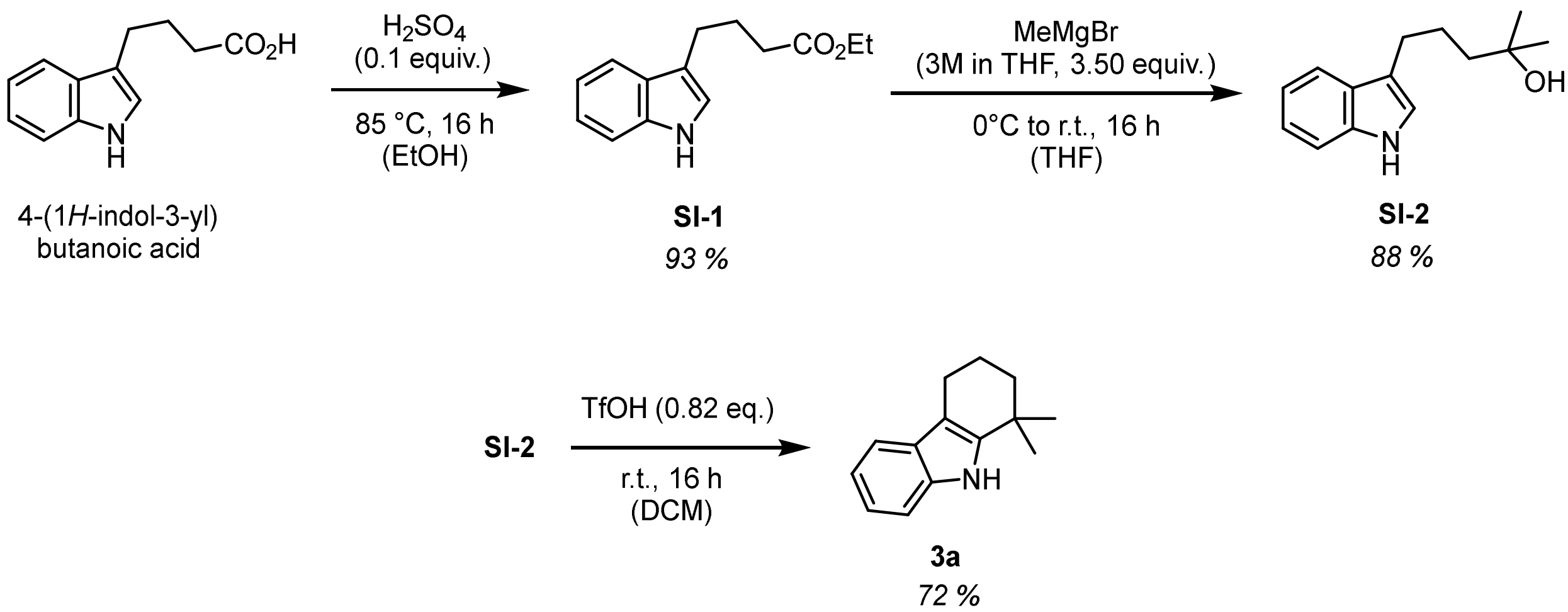

Figure SI-1. Synthesis of substrate **3a**. *Note*: **3a** can alternatively be obtained by *Fischer-indole* synthesis by combining phenyl hydrazine with 2,2-dimethyl-cyclohexan-1-one.

2.1.1. Ethyl 4-(1*H*-indol-3-yl)butanoate (SI-1)

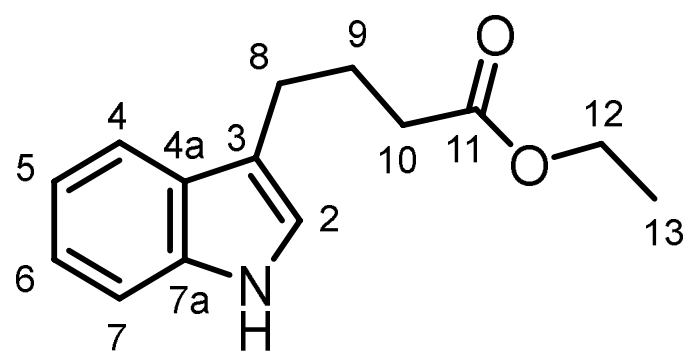

SI-1

Compound **SI-1** was prepared *via* a modified literature procedure.[2]

In a 250 mL *Schlenk* flask, sulfuric acid (132 µL, 2.46 mmol, 0.10 equiv.) was added to a solution of 4-(1*H*-indol-3-yl)butanoic acid (5.00 g, 24.6 mmol, 1.00 equiv.) in EtOH (176 mL, 140 mM). The mixture was stirred at 85 °C for 16 h and then cooled down to room temperature. The reaction mixture was concentrated *in vacuo* and diluted with EtOAc (50 mL). The resulting solution was washed with a saturated aqueous $NaHCO_3$ solution (2×30 mL). The organic layer was washed with brine and dried over Na_2SO_4. After filtration and removal of all volatiles under reduced pressure the crude material was obtained as a brown oil, which crystallized upon standing and was used without further purification due to high purity.

Brown solid, 5.30 g (93%); **TLC** (*n*-pentane/EtOAc, 6/4): R_f = 0.82 [UV]; **^{1}H NMR** (500 MHz, $CDCl_3$, 298 K): δ (ppm) = 7.98 (s, 1H, N*H*), 7.62 (dd, 1H, $^3J_{H-H}$ = 7.9 Hz, $^4J_{H-H}$ = 1.0 Hz, H^4), 7.36 (dd, 1H, $^3J_{H-H}$ = 8.2 Hz, $^4J_{H-H}$ = 1.0 Hz, H^7), 7.20 (ddd, 1H, $^3J_{H-H}$ = 8.2 Hz, 7.0 Hz, $^4J_{H-H}$ = 1.0 Hz, H^6), 7.12 (ddd, 1H, $^3J_{H-H}$ = 7.9 Hz, 7.0 Hz, $^4J_{H-H}$ = 1.0 Hz, H^5), 6.99 (d, 1H, $^4J_{H-H}$ = 1.0 Hz, H^2), 4.13 (q, 2H, $^3J_{H-H}$ = 7.1 Hz, H^{12}), 2.82 (td, 2H, $^3J_{H-H}$ = 7.5 Hz, $^4J_{H-H}$ = 1.0 Hz, H^8), 2.39 (t, 2H, $^3J_{H-H}$ = 7.5 Hz, H^{10}), 2.06 (*virt.* p, $^3J_{H-H} \approx {}^3J_{H-H'}$ = 7.5 Hz, 2H, H^9), 1.26 (t, 3H, $^3J_{H-H}$ = 7.1 Hz, H^{13}); **^{13}C{^{1}H} NMR** (126 MHz, $CDCl_3$, 298 K): δ (ppm) = 174.0 (C^{11}), 136.4 (C^{7a}), 127.5 (C^{4a}), 122.0 (C^6), 121.6 (C^2), 119.3 (C^5), 119.0 (C^4), 115.8 (C^3), 111.2 (C^7), 60.4 (C^{12}), 34.1 (C^{10}), 25.5 (C^9), 24.6 (C^8), 14.4 (C^{13}); **HR-MS** (ESI^+): *m/z* calculated for $[C_{14}H_{17}NO_2+H]^+$: 232.1332 ($[M+H]^+$); found: 232.1337; **IR:** $\tilde{\nu}$ = 3332 (m, NH), 3036 (w, sp^2-CH), 3063 (w, sp^2-CH), 2981 (w, sp^3-CH), 2945 (w, sp^3-CH), 2927 (w, sp^3-CH), 2903 (w, sp^3-CH), 2874 (w, sp^3-CH), 1694 (s, C=C), 1556 (m, C=C), 1492 (m, C=C), 1321 (m), 1302 (s, C-O-C), 1120 (m), 1081 (m), 856 (m), 813 (m), 744 (s) cm^{-1}.

2.1.2 5-(1*H*-Indol-3-yl)-2-methylpentan-2-ol (SI-2)

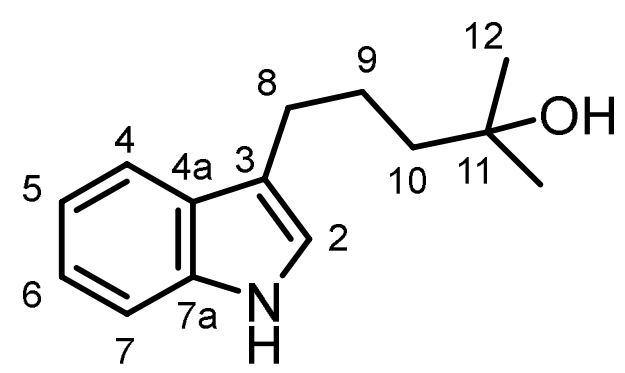

SI-2

Compound **SI-2** was prepared *via* a modified literature procedure.[3]

Indole ester **SI-1** (5.30 g, 22.9 mmol, 1.00 equiv.) and THF (76 mL, 0.30 M) were added to a *Schlenk* flask under inert conditions. The solution was cooled to 0 °C and 26.7 mL of a methyl magnesium bromide solution (3 M in THF, 27.7 g, 80.2 mmol, 3.50 equiv.) was added slowly. The reaction mixture was stirred at room temperature overnight, then quenched with 15 mL of saturated aqueous NH_4Cl solution and 25 mL of EtOAc were added. After extraction with EtOAc (3 × 75 mL), the organic phases were combined, washed with brine, dried over Na_2SO_4 and filtered. All volatiles were removed under reduced pressure. The crude product was purified by column chromatography (dry load, *n*-pentane/EtOAc = 6/4) to yield 4.38 g of the tertiary alcohol **SI-2** (20.1 mmol, 88%) as a beige solid.

Beige solid, 4.38 g (88%); **TLC** (*n*-pentane/EtOAc, 6/4): R_f = 0.38 [UV]; **^{1}H NMR** (500 MHz, $CDCl_3$, 298 K): δ (ppm) = 7.94 (s, 1H, N*H*), 7.61 (dd, 1H, $^3J_{H-H}$ = 7.9 Hz, $^4J_{H-H}$ = 1.1 Hz, H^4), 7.36 (dd, 1H, $^3J_{H-H}$ = 8.2 Hz, $^4J_{H-H}$ = 1.0 Hz, H^7), 7.19 (ddd, 1H, $^3J_{H-H}$ = 8.2 Hz, 7.1 Hz, $^4J_{H-}$

$_{H}$ = 1.1 Hz, H^6), 7.11 (ddd, 1H, $^3J_{H\text{-}H}$ = 7.9 Hz, 7.1 Hz, $^4J_{H\text{-}H}$ = 1.0 Hz, H^5), 7.00 (d, 1H, $^4J_{H\text{-}H}$ = 1.0 Hz, H^2), 2.78 (td, 2H, $^3J_{H\text{-}H}$ = 7.5 Hz, $^4J_{H\text{-}H}$ = 1.0 Hz, H^8), 1.84 – 1.74 (m, 2H, H^9), 1.63 – 1.56 (m, 2H, H^{10}), 1.22 (s, 6H, H^{12}); **^{13}C{^{1}H} NMR** (126 MHz, $CDCl_3$, 298 K): δ (ppm) = 136.4 (C^{7a}), 127.7 (C^{4a}), 122.0 (C^6), 121.2 (C^2), 119.3 (C^5), 119.1 (C^4), 116.9 (C^3), 111.2 (C^7), 71.2 (C^{11}), 44.0 (C^{10}), 29.4 (2C, C^{12}), 25.7 (C^8), 25.1 (C^9);); **HR-MS** (ESI^+): *m/z* calculated for $[C_{14}H_{20}NO+H]^+$: 218.1539 ($[M+H]^+$); found: 218.1540; **IR:** $\tilde{\nu}$ = 3536 (s, OH), 3257 (m, NH), 3062 (w, sp^2-CH), 2970 (w, sp^3-CH), 2930 (w, sp^3-CH), 2877 (w, sp^3-CH), 2842 (w, sp^3-CH), 1618 (s, C=C), 1583 (m, C=C), 1493 (m, C=C), 1317 (m), 1121 (m), 1070 (m), 840 (m), 795 (m, sp^2-CH), 748 (s, sp^2-CH) cm^{-1}.

2.1.3 1,1-Dimethyl-2,3,4,9-tetrahydro-1*H*-carbazole (3a)

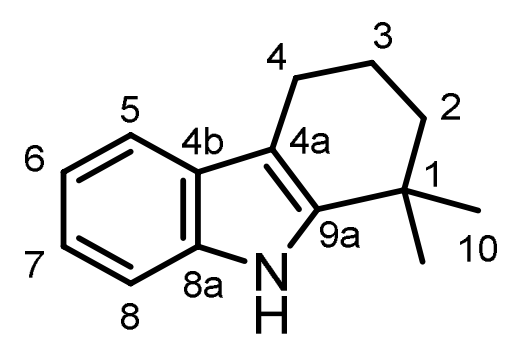

3a

Compound **3a** was prepared *via* a modified literature procedure. [3]

To a solution of alcohol **SI-2** (4.31 g, 19.9 mmol, 1.00 equiv.) in dichloromethane (200 mL, 10 mM), 439 µL TfOH (744 mg, 4.96 mmol, 0.25 equiv.) were added under stirring, turning the mixture green. After three hours of stirring at room temperature, further TfOH (1 mL, 1.70 g, 11.3 mmol, 0.57 equiv.) was added. The mixture was stirred overnight at room temperature, after which TLC showed complete conversion. Subsequently, 20 mL of saturated aqueous $NaHCO_3$ solution was added and the brown organic layer was separated, then washed with more saturated aqueous $NaHCO_3$ solution (3 × 60 mL). The aqueous phases were combined and extracted with dichloromethane (1 × 10 mL). All organic phases were combined, dried over Na_2SO_4 and filtered. All volatiles were removed under reduced pressure and the crude product was purified by column chromatography (*n*-pentane/EtOAc = 9/1) to yield 2.85 g of tetrahydrocarbazole **3a** (14.3 mmol, 72%) as a beige solid. **3a** shows minor oxidation when stored under air (very slowly over months) and minor decomposition on silica (*n*-pentane/EtOAc, 8/2): R_f = 0.39 [UV], as observed by two dimensional TLC. Upon storage under inert conditions no oxidation were observed.

Beige solid, 2.85 g (72%); **TLC** (*n*-pentane/EtOAc, 8/2): R_f = 0.84 [UV]; **^{1}H NMR** (500 MHz, $CDCl_3$, 298 K): δ (ppm) = 7.72 (s, 1H, N*H*), 7.46 (dd, $^3J_{H\text{-}H}$ = 8.0 Hz, $^4J_{H\text{-}H}$ = 1.3 Hz, H^5), 7.30

(dd, 1H, ${}^{3}J_{H\text{-}H}$ = 8.0 Hz, ${}^{4}J_{H\text{-}H}$ = 1.0 Hz, H^{8}), 7.12 (ddd, 1H, ${}^{3}J_{H\text{-}H}$ = 8.0, 7.1 Hz, ${}^{4}J_{H\text{-}H}$ = 1.3 Hz, H^{7}), 7.07 (ddd, 1H, ${}^{3}J_{H\text{-}H}$ = 8.0, 7.1 Hz, ${}^{4}J_{H\text{-}H}$ = 1.0 Hz, H^{6}), 2.69 (t, 2H, ${}^{3}J_{H\text{-}H}$ = 6.2 Hz, H^{4}), 1.94 – 1.87 (m, 2H, H^{3}), 1.77 – 1.71 (m, 2H, H^{2}), 1.33 (s, 6H, H^{10}); **^{13}C{^{1}H} NMR** (126 MHz, $CDCl_3$, 298 K): δ (ppm) = 141.9 (C^{9a}), 135.8 (C^{8a}), 127.7 (C^{4b}), 121.2 (C^{7}), 119.2 (C^{6}), 118.3 (C^{5}), 110.6 (C^{8}), 108.9 (C^{4a}), 39.4 (C^{2}), 31.6 (C^{1}), 29.5 (2C, C^{10}), 21.5 (C^{4}), 20.4 (C^{3}); **HR-MS** (ESI^{+}): *m/z* calculated for $[C_{14}H_{17}N+H]^{+}$: 200.1434 ($[M+H]^{+}$); found: 200.1439; **IR:** $\tilde{\nu}$ = 3399 (m, NH), 3052 (w, sp^{2}-CH), 2960 (w, sp^{3}-CH), 2917 (w, sp^{3}-CH), 2842 (w, sp^{3}-CH), 2163 (w, sp^{3}-CH), 1672 (s, C=C), 1582 (m, C=C), 1459 (m, C=C), 1317 (m), 1121 (m), 1044 (m), 837 (m), 789 (m, sp^{2}-CH), 746 (s, sp^{2}-CH) cm^{-1}.

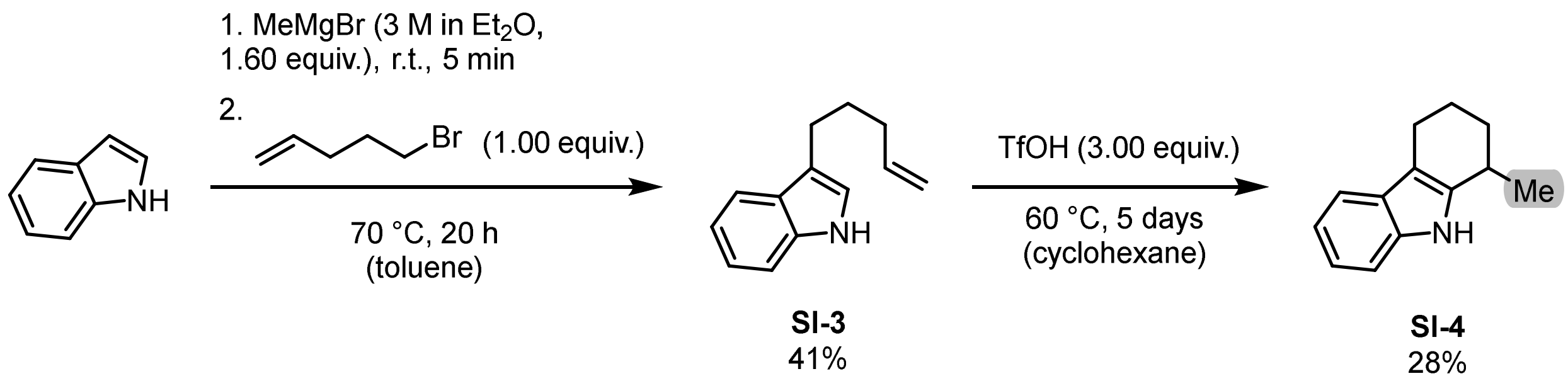

Figure SI-2. Synthesis of monomethyl substrate **SI-4**.

2.1.4 3-(Pent-4-en-1-yl)-1*H*-indol (SI-3)

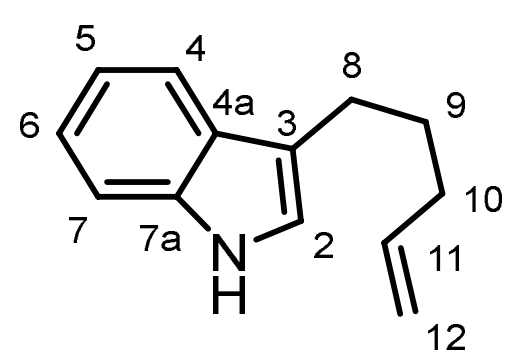

SI-3

Compound **SI-3** was prepared *via* a modified literature procedure. [4]

To a solution of MeMgBr (1.80 mL, 5.40 mmol, 3 M in Et_2O, 1.60 equiv.) in toluene (5 mL) was added a solution of indole (593 mg, 5.06 mmol, 1.50 equiv.) in toluene (5 mL) at room temperature. After 5 minutes, 5-bromopentene (0.40 mL, 503 mg, 3.38 mmol, 1.00 equiv.) was added and the reaction was stirred at 70 °C for 20 h. The red suspension was cooled to room temperature and was quenched with saturated NH_4Cl. The organic phase was separated and the aqueous phase was extracted with EtOAc thrice. The combined organic layers were washed once with water and brine, dried over Na_2SO_4, filtered and then concentrated *in vacuo*. The

crude was purified by column chromatography on silica gel (dry load, *n*-pentane → *n*-pentane/EtOAc = 98/2) to give 3-(pent-4-en-1-yl)-1*H*-indol (**SI-3**) (258 mg, 1.39 mmol, 41%) as a colorless oil.

Colorless oil, 258 mg (41%); **TLC:** R_f = 0.33 (*n*-pentane/EtOAc, 98/2) [UV]. **^{1}H NMR** (500 MHz, $CDCl_3$, 298 K): δ (ppm) = 7.90 (br s, 1H, N*H*), 7.62 (dd, $^3J_{H\text{-}H}$ = 8.0 Hz, $^4J_{H\text{-}H}$ = 1.1 Hz, 1H, H^4), 7.36 (dt, $^3J_{H\text{-}H}$ = 8.0 Hz, $^4J_{H\text{-}H}$ = 1.1 Hz, 1H, H^7), 7.20 (ddd, $^3J_{H\text{-}H}$ = 8.0, 7.0 Hz, $^4J_{H\text{-}H}$ = 1.1 Hz, 1H, H^6), 7.12 (ddd, $^3J_{H\text{-}H}$ = 8.0, 7.0 Hz, $^4J_{H\text{-}H}$ = 1.1 Hz, 1H, H^5), 7.00 – 6.97 (m, 1H, H^2), 5.88 (ddt, $^3J_{H\text{-}H\,(trans)}$ = 17.0 Hz, $^3J_{H\text{-}H\,(cis)}$ = 10.2 Hz, $^3J_{H\text{-}H}$ = 6.6 Hz, 1H, H^{11}), 5.05 (dq, $^3J_{H\text{-}H\,(trans)}$ = 17.0 Hz, $^2J_{H\text{-}H\,(gem)}$ = 1.5 Hz, 1H, H^{12}), 4.99 (ddt, $^3J_{H\text{-}H\,(cis)}$ = 10.2 Hz, $^4J_{H\text{-}H}$ = 2.3 Hz, $^2J_{H\text{-}H\,(gem)}$ = 1.5 Hz, 1H, H^{12}), 2.85 – 2.70 (m, 2H, H^8), 2.23 – 2.12 (m, 2H, H^{10}), 1.87 – 1.78 (m, 2H, H^9); **^{13}C{^{1}H} NMR** (126 MHz, $CDCl_3$, 298 K): δ (ppm) = 139.0 (C^{11}), 136.4 (C^{7a}), 127.7 (C^{4a}), 122.0 (C^6), 121.3 (C^2), 119.2 (C^4), 119.1 (C^5), 116.8 (C^3), 114.7 (C^{12}), 111.2 (C^7), 33.8 (C^{10}), 29.4 (C^9), 24.7 (C^8); **HR-MS** (ESI^+): *m/z* calculated for $[C_{13}H_{15}N+H]^+$: 186.1277 ($[M+H]^+$); found: 186.1276.

2.1.5 1-Methyl-2,3,4,9-tetrahydro-1*H*-carbazole (SI-4)

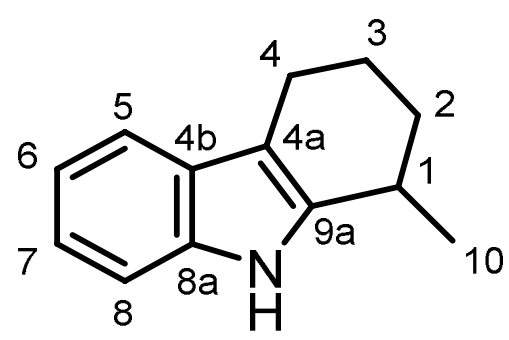

SI-4

Compound **SI-4** was prepared according to a modified literature procedure.[3] The compound is known in the literature.[4]

In a *Schlenk* flask, 3-(pent-4-en-1-yl)-1*H*-indole (174 mg, 939 µmol, 1.00 equiv.) was dissolved in cyclohexane (7 mL). Then, TfOH (249 µL, 2.82 mmol, 3.00 equiv.) was added, and the reaction mixture was stirred for 5 days at 60°C under an inert atmosphere. Subsequently, saturated aqueous $NaHCO_3$ (5 mL) was carefully added, and the reaction mixture was left to stir for 30 min. The mixture was then extracted with DCM (2×12 mL), and the combined organic phases were washed with brine and dried over Na_2SO_4. All volatiles were removed under reduced pressure and the crude product was purified by reverse phase flash chromatography (CH_3CN/H_2O = 1/9 → 100% CH_3CN). After removal of solvents by freeze drying, 48.7 mg of tetrahydrocarbazole **SI-4** (263 µmol, 28%) was obtained as a beige solid.

Beige solid, 48.7 mg, 28% yield; **TLC** (*n*-pentane/acetone = 98/2): R_f = 0.17 [UV], **^{1}H NMR** (500 MHz, $CDCl_3$, 298 K): δ (ppm) = 7.74 (br s, 1H, N*H*), 7.46 (dd, $^3J_{H\text{-}H}$ = 7.5 Hz, $^4J_{H\text{-}H}$ = 1.2 Hz, 1H, H^5), 7.30 (dd, $^3J_{H\text{-}H}$ = 8.0 Hz, $^4J_{H\text{-}H}$ = 1.1 Hz, 1H, H^8), 7.12 (ddd, $^3J_{H\text{-}H}$ = 8.0, 7.5 Hz, $^4J_{H\text{-}H}$ = 1.2 Hz, 1H, H^7), 7.07 (*virt.* td, $^3J_{H\text{-}H} \approx {}^3J_{H\text{-}H}$ = 7.5 Hz, 1.1 Hz, 1H, H^6), 3.03 – 2.94 (m, 1H, H^1), 2.79 – 2.62 (m, 2H, H^4), 2.10 – 2.03 (m, 1H, H^2), 2.03 – 1.96 (m, 1H, H^3), 1.83 – 1.72 (m, 1H, H^3), 1.57 – 1.51 (m, 1H, H^2), 1.31 (d, $^3J_{H\text{-}H}$ = 7.0 Hz, 3H, H^{10}); **^{13}C{^{1}H} NMR** (126 MHz, $CDCl_3$, 298 K): δ (ppm) = 138.7 (C^{9a}), 135.8 (C^{8a}), 127.8 (C^{4b}), 121.2 (C^7), 119.3 (C^6), 118.1 (C^5), 110.5 (C^8), 109.9 (C^{4a}), 32.5 (C^2), 28.8 (C^1), 22.1 (C^3), 21.3 (C^4), 20.4 (C^{10}); **HR-MS** (ESI^+): *m/z* calculated for $[C_{13}H_{15}N+H]^+$: 185.1199 ; found: 185.1186; **IR:** $\tilde{\nu}$ = 3396 (m, NH), 3049 (w, sp^2-CH), 2957 (w, sp^3-CH), 2921 (m, sp^3-CH), 2848 (w, sp^3-CH), 1619 (w, C=C), 1585 (w, C=C), 1469 (m), 1453 (m), 1006 (m), 738 (s), 725 (m), 702 (m) cm^{-1}.

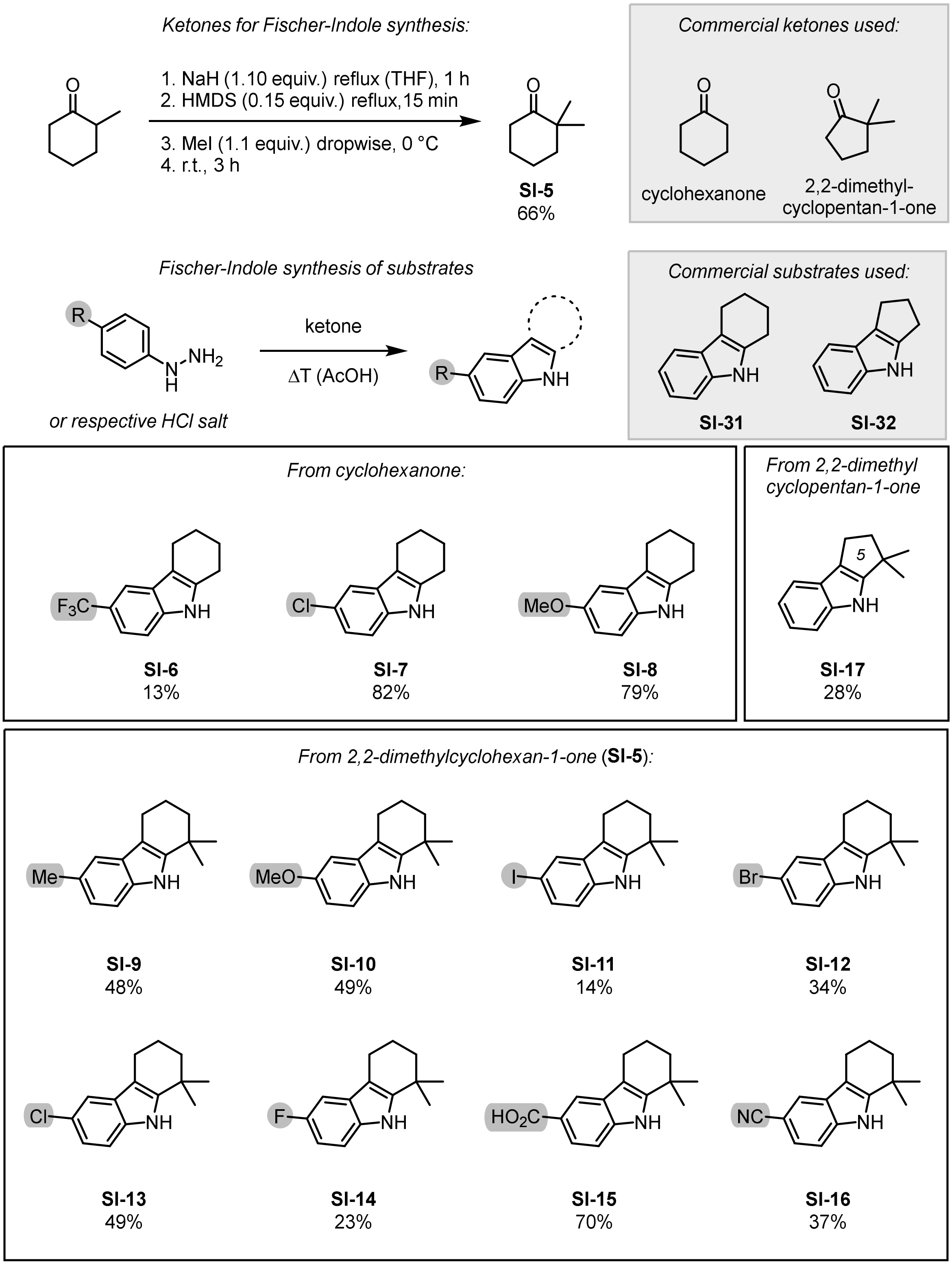

Figure SI-3. Synthesis of substrate precursor **SI-5** and overview over substrates synthesized by *Fischer-Indole* synthesis.

2.1.6 2,2-Dimethylcyclohexanone (SI-5)

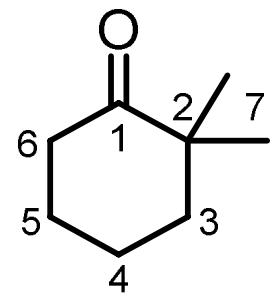

SI-5

Compound **SI-5** was prepared *via* a modified literature procedure. [5]

A 2 L *Schlenk* flask connected to a three-neck attachment with reflux condenser, dropping funnel and septum was charged with 36.5 g (912 mmol, 1.10 equiv.) NaH and 800 mL dry THF. First, 100 mL (829 mmol, 1.00 equiv.) of 2-methylcyclohexanone were added and the reaction mixture was refluxed at 75 °C for 1.5 h. Second, 25.9 mL (124 mmol, 0.15 equiv.) of bis(trimethylsilyl)amine were added and reaction mixture was refluxed for another 15 min at 75 °C. The reaction mixture was allowed to cool to room temperature, then to 0 °C and 56.8 mL (912 mmol, 1.10 equiv.) of methyl iodide were added slowly dropwise to the solution and the reaction mixture was stirred at room temperature overnight. Excess methyl iodide was removed *in vacuo* with an external cold trap and then Et_2O was added to precipitate NaI, which was filtered afterwards. Removal of solvent under reduced pressure (50 °C, 100 mbar) resulted approximately 130-150 mL of crude product. Purification by fractional vacuum distillation with medium-sized column (20 mbar, silicon bath temperature: 83 °C, head temperature: 50 °C) yielded 75.3 mL (544 mmol, 66%) of 2,2-dimethylcyclohexanone (**SI**-5) as colorless liquid. The material was used without further purification.

Colorless liquid, 75 mL (66%); **TLC:** R_f = 0.85 (*n*-pentane/EtOAc, 7/3) [UV]; **^{1}H NMR** (500 MHz, $CDCl_3$, 298 K): δ (ppm) = 2.37 (m, 2H, H^6), 1.82 (m, 2H, H^5), 1.72 (m, 2H, H^4), 1.64 (m, 2H, H^3), 1.09 (s, 6H, H^7); **^{13}C{^{1}H} NMR** (126 MHz, $CDCl_3$, 298 K): δ (ppm) = 216.5 (C^1), 45.4 (C^2), 41.3 (C^3), 38.5 (C^6), 27.8 (C^5), 25.4 (C^7), 21.6 (C^4); **IR:** $\tilde{\nu}$ = 2966 (w, sp^3-CH), 2932 (m, sp^3-CH), 2866 (w, sp^3-CH), 1705 (s, C=O), 1453 (m, sp^3-CH), 1428 (w, sp^3-CH), 1385 (w, sp^3-CH_3), 1365(w, sp^3-CH_3), 1314 (w), 1125 (m), 1100 (w), 992 (w), 982 (w), 960 (w), 855 (w) cm^{-1}.

2.1.7 6-(Trifluoromethyl)-2,3,4,9-tetrahydro-1*H*-carbazole (SI-6)

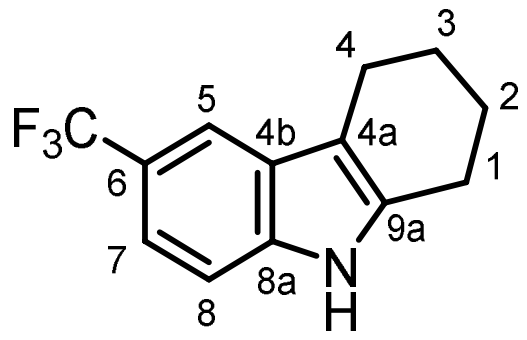

SI-6

Compound **SI-6** was prepared *via* a modified literature procedure.[6]

Cyclohexanone (1.18 mL, 1.11 g, 11.4 mmol, 1.00 equiv.) was added to a solution of 4-(trifluoromethyl)phenylhydrazine (2.00 g, 11.4 mmol, 1.00 equiv.) in acetic acid (40 mL). The mixture was stirred at 70°C for 16 h with reaction progress monitored *via* TLC. To end the reaction, the mixture was diluted with water (70 mL). The precipitated solid was filtered, washed with water, and purified by column chromatography (*n*-pentane/EtOAc = 8/2) to yield 350 mg of tetrahydrocarbazole **SI-5** (1.48 mmol, 13%) as beige solid.

Off-white solid (350 mg, 13%); **TLC:** R_f = 0.63 (n-pentane/EtOAc = 8/2) [UV]; **^{1}H NMR** (500 MHz, CD_2Cl_2, 298 K): δ (ppm) = 8.03 (br s, 1H, N*H*), 7.75 – 7.72 (m, 1H, H^5), 7.39 – 7.30 (m, 2H, $H^{7,8}$), 2.80 – 2.69 (m, 4H, $H^{1,4}$), 1.99 – 1.83 (m, 4H, $H^{2,3}$); **^{13}C{^{1}H} NMR** (126 MHz, $CDCl_3$, 298 K): δ (ppm) = 137.1 (C^{8a}), 136.1 (C^{9a}), 127.4 (C^{4b}), 125.7 (q, $^1J_{C\text{-}F}$ = 271.5 Hz, *C*F_3), 121.6 (q, $^2J_{C\text{-}F}$ = 31.4 Hz, C^6), 117.9 (q, $^3J_{C\text{-}F}$ = 4.2 Hz, C^5), 115.5 (q, $^3J_{C\text{-}F}$ = 4.2 Hz, C^7), 111.3 (C^{4a}), 110.4 (C^8), 23.3 ($C^{1/2/3}$), 23.2 ($C^{1/2/3}$), 23.1 ($C^{1/2/3}$), 20.8 (C^4); **^{19}F NMR** (376 MHz, $CDCl_3$, 298 K): δ (ppm) = – 60.1; **HR-MS** (ESI^+): *m/z* calculated for $[C_{12}H_{13}NO_2+H]^+$: 240.0995; found: 240.0996; **IR:** $\tilde{\nu}$ = 3405 (m, NH), 2944 (w, sp^3-CH), 2847 (w, sp^3-CH), 1628 (w, C=C), 1470 (w), 1444 (w), 1356 (w), 1339 (m), 1321 (s), 1156 (s), 1125 (m), 1105 (s), 1051 (s), 998 (m), 883 (m), 812 (s) cm^{-1}.

2.1.8 6-Chloro-2,3,4,9-tetrahydro-1*H*-carbazole (SI-7)

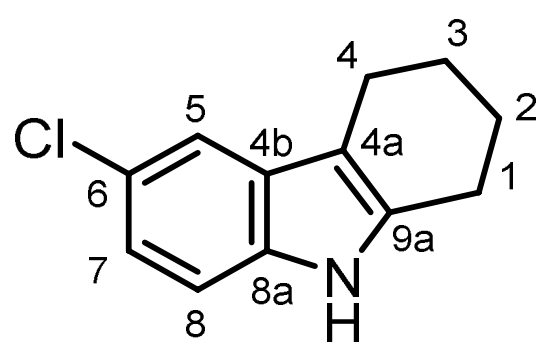

SI-7

Compound **SI-7** was prepared *via* a modified literature procedure.[6]

To a solution of cyclohexanone (476 µL, 4.61 mmol, 1.00 equiv.) in acetic acid (15 mL) was added 4-(chlorophenyl)hydrazine (825 mg, 4.61 mmol, 1.00 equiv.) portion wise over 30 min. The resulting reaction mixture was refluxed for 8 h, and the reaction progress was monitored by TLC. After cooling to room temperature, the reaction mixture was poured onto crushed ice. The precipitated solid was collected by filtration and dried in vacuo to afford 780 mg of tetrahydrocarbazole **SI-7** (3.78 mmol, 82% yield) as a beige solid. Due to its high purity, the product was used without further purification.

Beige solid, 780 mg (82% yield); **^{1}H NMR** (500 MHz, $CDCl_3$, 298 K): δ (ppm) = 7.70 (br s, 1H, N*H*), 7.41 (d, $^{4}J_{H\text{-}H}$ = 2.1 Hz, 1H, H^{5}), 7.17 (d, $^{3}J_{H\text{-}H}$ = 8.4 Hz, 1H, H^{8}), 7.05 (dd, $^{3}J_{H\text{-}H}$ = 8.4 Hz, $^{4}J_{H\text{-}H}$ = 2.1 Hz, 1H, H^{7}), 2.74 – 2.69 (m, 2H, $H^{1,4}$), 2.67 – 2.63 (m, 2H, $H^{1.4}$), 1.95 – 1.82 (m, 4H, $H^{2,3}$); **^{13}C{^{1}H} NMR** (126 MHz, $CDCl_3$, 298 K): δ (ppm) = 135.9 (C^{9a}), 134.1 (C^{8a}), 129.1 (C^{4b}), 124.9 (C^{6}), 121.2 (C^{7}), 117.5 (C^{5}), 111.3 (C^{8}), 110.2 (C^{4a}), 23.4 ($C^{1/2/3}$), 23.3 ($C^{1/2/3}$), 23.2 ($C^{1/2/3}$), 20.9 (C^{4}); **HR-MS** (ESI^{+}): *m/z* calculated for $[C_{12}H_{13}ClN+H]^{+}$: 206.0731; found: 206.0730; **IR:** $\tilde{\nu}$ = 3404 (m, NH), 2940 (w, sp^{3}-CH), 2849 (w, sp^{3}-CH), 1579 (w, C=C), 1470 (w), 1441 (w), 1436 (w), 1313 (w), 1057 (m), 862 (w), 800 (s), 657 (w) cm^{-1}.

2.1.9 6-Methoxy-2,3,4,9-tetrahydro-1*H*-carbazole (SI-8)

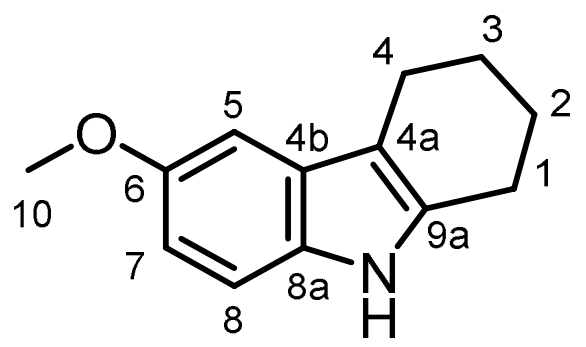

SI-8

Compound **SI-8** was prepared *via* a modified literature procedure. [6]

To a solution of cyclohexanone (2.98 mL, 28.6 mmol, 1.00 equiv.) in acetic acid (100 mL) was added 4-(methoxyphenyl)hydrazine (5.00 g, 28.6 mmol, 1.00 equiv.) portion wise over 30 min. The resulting reaction mixture was refluxed for 8 h, and the reaction progress was monitored by TLC. After cooling to room temperature, the reaction mixture was poured onto crushed ice. The precipitated solid was collected by filtration and dried in vacuo to afford 4.54 g of tetrahydrocarbazole **SI-8** (22.6 mmol, 79%) as a light brown solid. Due to its high purity, the product was used without further purification.

Light brown solid, 4.54 g (79%); **^{1}H NMR** (500 MHz, $CDCl_3$, 298 K): δ (ppm) = 7.55fz (br s, 1H, N*H*), 7.16 (d, $^{3}J_{H\text{-}H}$ = 8.7 Hz, 1H, H^{8}), 6.93 (d, $^{4}J_{H\text{-}H}$ = 2.5 Hz, 1H, H^{5}), 6.76 (dd, $^{3}J_{H\text{-}H}$ = 8.7

Hz, ${}^4J_{H\text{-}H}$ = 2.5 Hz, 1H, H^7), 3.86 (s, 3H, H^{10}), 2.75 – 2.63 (m, 4H, $H^{1,4}$), 1.96 – 1.83 (m, 4H, $H^{2,3}$); **^{13}C{^{1}H} NMR** (126 MHz, $CDCl_3$, 298 K): δ (ppm) = 154.0 (C^6), 135.2 (C^{9a}), 130.8 (C^{8a}), 128.3 (C^{4b}), 111.1 (C^8), 110.6 (C^7), 110.2 (C^{4a}), 100.3 (C^5), 56.1 (C^{10}), 23.5 ($C^{1/2/3}$), 23.4 ($C^{1/2/3}$), 23.3 ($C^{1/2/3}$), 21.1 (C^4); **IR:** $\tilde{\nu}$ = 3387 (m, NH), 2951 (w, sp^3-CH), 2915 (w, sp^3-CH), 2850 (w, sp^3-CH), 1590 (w, C=C), 1484 (m), 1452 (m), 1430 (m), 1217 (s), 1136 (m), 1029 (m), 830 (m), 799 (s) cm^{-1}.

2.1.10 1,1,6-Trimethyl-2,3,4,9-tetrahydro-1*H*-carbazole (SI-9)

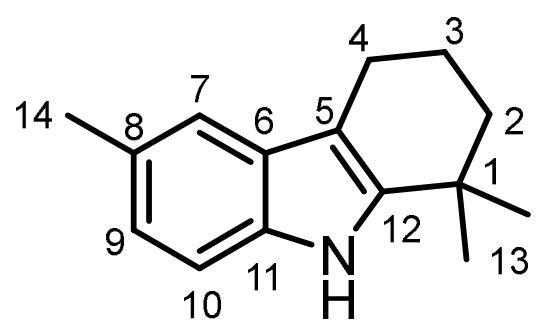

SI-9

Compound **SI-9** was synthesized following a modified procedure based on the method reported by *Lin* for a related analogue.[6]

Para-tolylhydrazine hydrochloride (1.00 g, 6.30 mmol, 1.00 equiv.) was suspended in 15 mL of acetic acid. 872 µL (6.30 mmol, 1.00 equiv.) of 2,2-dimethylcyclohexanone was added and the mixture was stirred at 110 °C overnight. After allowing the mixture to cool to room temperature, the acetic acid was removed under reduced pressure, and the crude was partitioned between EtOAc and aqueous saturated $NaHCO_3$ solution. The layers were separated, and the aqueous layer was extracted with EtOAc (3 x 30 mL) and the combined organic layers washed with 30 mL aqueous saturated NaCl solution, dried over Na_2SO_4 and filtered. Purification by column chromatography (silica, *n*-pentane/EtOAc = 99/1) resulted in 643 mg (3.02 mmol, 48%) of target compound, a white solid.

White solid, 643 mg (48%); **TLC:** R_f = 0.34 (*n*-pentane/EtOAc, 99/1) [UV]; **^{1}H NMR** (500 MHz, $CDCl_3$, 298 K): δ (ppm) = 7.62 (s, 1H, N*H*), 7.24 (m, 1H, H^7), 7.18 (d, ${}^3J_{H\text{-}H}$ = 8.1 Hz, 1H, H^{10}), 6.93 (dd, ${}^3J_{H\text{-}H}$ = 8.1 Hz, ${}^4J_{H\text{-}H}$ = 1.7 Hz, 1H, H^9), 2.65 (t, ${}^3J_{H\text{-}H}$ = 6.2 Hz, 2H, H^4), 2.43 (s, 3H, H^{14}), 1.93 – 1.81 (m, 2H, H^3), 1.75 – 1.68 (m, 2H, H^2), 1.31 (s, 6H, H^{13}); **^{13}C{^{1}H} NMR** (126 MHz, $CDCl_3$, 298 K): δ (ppm) = 142.1 (C^{12}), 134.1 (C^{11}), 128.4 (C^8), 128.0 (C^6), 122.6 (C^9), 118.1 (C^7), 110.2 (C^{10}), 108.5 (C^5), 39.5 (C^2), 31.6 (C^1), 29.5 (2C, C^{13}), 21.6 (C^4), 21.5 (C^{14}), 20.5 (C^3); **HR-MS** (ESI^+): m/z calculated for $[C_{15}H_{19}N+H]^+$: 214.1590 ($[M+H]^+$); found: 214.1545; **IR:** $\tilde{\nu}$ = 3412 (m, NH), 2924 (w, sp^3-CH), 2847 (w, sp^3-

CH), 1585 (w, C=C), 1474 (m, C=C), 1462 (m, C=C), 1363 (w, sp^3-CH_3), 1305 (m), 1252 (w), 1205 (w), 1186 (w), 1121 (w), 943 (w), 919 (w), 883 (w), 876 (w), 812 (w), 799 (s), 710 (w) cm^{-1}.

2.1.11 6-Methoxy-1,1-dimethyl-2,3,4,9-tetrahydro-1*H*-carbazole (SI-10)

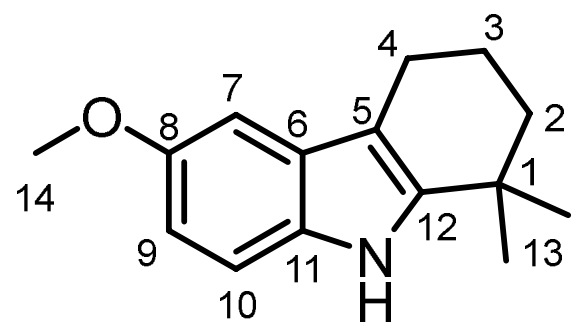

SI-10

Compound **SI-10** was synthesized following a modified procedure based on the method reported by *Lin* for a related analogue. [6]

(4-Methoxyphenyl)hydrazine hydrochloride (1.00 g, 5.73 mmol, 1.00 equiv.) was dissolved in 15 mL of acetic acid and 2,2-dimethylcyclohexanone (792 μL, 5.73 mmol, 1.00 equiv.) was added under an argon atmosphere to the solution. First, the reaction mixture was stirred for 1 h at 70 °C and then for 30 min at 110 °C to give a dark suspension. The reaction mixture was allowed to cool to room temperature, concentrated under reduced pressure and neutralized with an aqueous saturated $NaHCO_3$ solution. The organic and aqueous layers were separated and the aqueous layer was extracted with EtOAc (3 x 30 mL). The combined organic layers were washed with 40 mL of aqueous saturated NaCl solution, dried over Na_2SO_4 and filtered. The solvent was removed *in vacuo* and the dark crude residue was adsorbed on a small amount of Celite and purified by column chromatography (silica, *n*-pentane/EtOAc = 97/3). Removal of the solvent under reduced pressure resulted in 644 mg (2.81 mmol, 49%) of **SI-10** as a yellowish solid.

Pale yellow solid, 644 mg (49%); **TLC:** R_f = 0.21 (*n*-pentane/EtOAc, 97/3) [UV]; **^{1}H NMR** (500 MHz, $CDCl_3$, 298 K): δ (ppm) = 7.61 (br s, 1H, NH), 7.18 (d, $^3J_{H\text{-}H}$ = 8.7 Hz, 1H, H^{10}), 6.93 (d, $^4J_{H\text{-}H}$ = 2.5 Hz, 1H, H^7), 6.78 (dd, $^3J_{H\text{-}H}$ = 8.7 Hz, $^4J_{H\text{-}H}$ = 2.5 Hz, 1H, H^9), 3.86 (s, 3H, H^{14}), 2.66 (t, $^3J_{H\text{-}H}$ = 6.2 Hz, 2H, H^4), 1.93 – 1.87 (m, 2H, H^3), 1.74 – 1.71 (m, 2H, H^2), 1.32 (s, 6H, H^{13}); **^{13}C{^{1}H} NMR** (126 MHz, $CDCl_3$, 298 K): δ (ppm) = 154.0 (C^8), 142.9 (C^{12}), 130.8 (C^{11}), 128.1 (C^6), 111.2 (C^{10}), 110.9 (C^9), 108.8 (C^5), 100.7 (C^7), 56.2 (C^{14}), 39.4 (C^2), 31.7 (C^1), 29.5 (2C, C^{13}), 21.5 (C^4), 20.4 (C^3); **HR-MS** (ESI^+): m/z calculated for $[C_{15}H_{19}NO+H]^+$: 230.1539 ($[M+H]^+$); found: 230.1538; **IR:** $\tilde{\nu}$ = 3415 (m, NH), 2953 (w, sp^3-

CH), 2930 (w, sp^3-CH), 2910 (w, sp^3-CH), 1621 (w, C=C), 1587 (m, C=C), 1482 (s, C=C), 1457 (s, C=C), 1429 (s, C=C), 1291 (m), 1250 (w), 1218 (s, C-O-C), 1202 (s), 1170 (m), 1088 (m), 1027 (m), 948 (w), 933 (w), 922 (w), 829 (s), 822 (m), 800 (s), 776 (w), 709 (w), 688 (w) cm^{-1}.

2.1.12 6-Iodo-1,1-dimethyl-2,3,4,9-tetrahydro-1*H*-carbazole (SI-11)

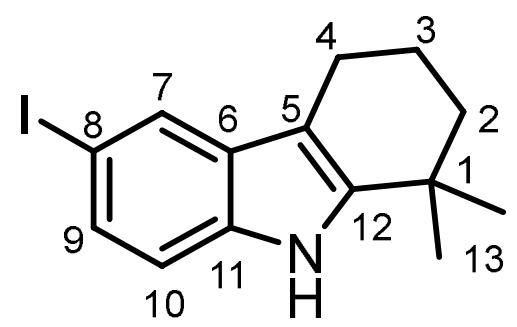

SI-11

Compound **SI-11** was synthesized following a modified procedure based on the method reported by *Lin* for a related analogue. [6]

(4-Iodophenyl)hydrazine hydrochloride (1.00 g, 3.70 mmol, 1.00 equiv.) was dissolved in 15 mL of acetic acid. 2,2-dimethylcyclohexanone (512 µL 3.70 mmol, 1.00 equiv.) was added to the solution under an argon atmosphere. The reaction mixture was stirred at 100 °C overnight. After allowing to cool to room temperature, most of the solvent was removed using a rotary evaporator. The residue was then neutralized with a saturated aqueous $NaHCO_3$ solution, and the resulting mixture was separated into organic and aqueous layers. The aqueous phase was extracted with DCM (3 × 30 mL). The combined organic layers were washed with 30 mL of saturated NaCl solution, dried over Na_2SO_4, filtered, and concentrated under reduced pressure. Purification of the crude product by normal phase Biotage flash chromatography afforded 172 mg (529 µmol, 14%) of **SI-11** as a white solid.

White solid, 172 mg (14%); **TLC:** R_f = 0.75 (*n*-pentane/EtOAc, 8/2) [UV]; **^{1}H NMR** (500 MHz, $CDCl_3$, 298 K): δ (ppm) = 7.78 (d, $^4J_{H\text{-}H}$ = 1.7 Hz, 1H, H^7), 7.74 (s, 1H, NH), 7.36 (dd, $^3J_{H\text{-}H}$ = 8.4 Hz, $^4J_{H\text{-}H}$ = 1.7 Hz, 1H, H^9), 7.07 (d, $^3J_{H\text{-}H}$ = 8.4 Hz, 1H, H^{10}), 2.63 (t, $^3J_{H\text{-}H}$ = 6.2 Hz, 2H, H^4), 1.94 – 1.81 (m, 2H, H^3), 1.76-1.67 (m, 2H, H^2), 1.32 (s, 6H, H^{13}); **^{13}C{^{1}H} NMR** (126 MHz, $CDCl_3$, 298 K): δ (ppm) = 142.9 (C^{12}), 134.9 (C^{11}), 130.5 (C^6), 129.4 (C^9), 127.2 (C^7), 112.5 (C^{10}), 108.5 (C^5), 82.5 (C^8), 39.2 (C^2), 31.6 (C^1), 29.4 (2C, C^{13}), 21.3 (C^4), 20.3 (C^3); **HR-MS** (ESI^+): m/z calculated for $[C_{14}H_{16}IN+H]^+$: 326.0400 ($[M+H]^+$); found: 326.0401; **IR:** $\tilde{\nu}$ = 3424 (m, NH), 2923 (m, sp^3-CH), 1473 (m, C=C), 1455 (m, C=C),

1429 (m, C-H), 1299 (s), 1273 (m), 1246 (m), 1206 (w), 1166 (m), 912 (m), 867 (m), 853 (w), 792 (s), 744 (w), 726 (w), 702 (w) cm^{-1}.

2.1.13 6-Bromo-1,1-dimethyl-2,3,4,9-tetrahydro-1*H*-carbazole (SI-12)

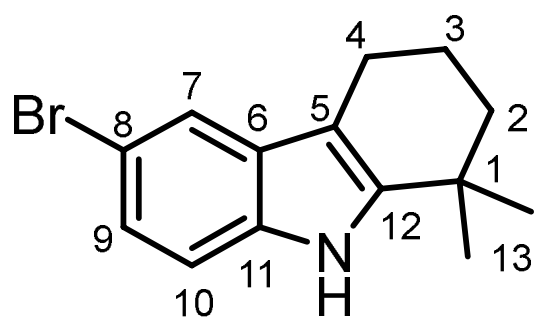

SI-12

Compound **SI-12** was synthesized following a modified procedure based on the method reported by *Lin* for a related analogue. [6]

A round-bottom flask with reflux condenser was charged with 1.00 g (5.35 mmol, 1.00 equiv.) of (4-bromophenyl) hydrazine and dissolved in 10 mL of acetic acid. 740 µL (5.35 mmol, 1.00 equiv.) of 2,2-dimethylcyclohexanone was added to the solution and stirred at 70 °C for 1.5 h, and afterwards at 110 °C for 30 min. The reaction mixture was allowed to reach room temperature and subsequently acetic acid was removed under reduced pressure. EtOAc and water were added along with an aqueous saturated $NaHCO_3$ solution, and the layers were separated. The aqueous layer was extracted with EtOAc (3 × 30 mL), and the combined organic layers were washed with 30 mL of aqueous saturated NaCl solution, dried over Na_2SO_4 and filtered. Purification by column chromatography (silica, *n*-pentane/EtOAc = 99/1) and removal of all volatiles *in vacuo* yielded 501 mg (1.80 mmol, 34%) of target compound as an orange solid.

Orange solid, 501 mg (34%); **TLC:** R_f = 0.23 (*n*-pentane/EtOAc, 99/1) [UV]; **^{1}H NMR** (500 MHz, $CDCl_3$, 298 K): δ (ppm) = 7.75 (br s, 1H, NH), 7.56 (d, $^4J_{H\text{-}H}$ = 1.7 Hz, 1H, H^7), 7.19 (dd, $^3J_{H\text{-}H}$ = 8.5 Hz, $^4J_{H\text{-}H}$ = 1.7 Hz, 1H, H^9), 7.15 (d, $^3J_{H\text{-}H}$ = 8.5 Hz, 1H, H^{10}), 2.63 (t, $^3J_{H\text{-}H}$ = 6.2 Hz, 2H, H^4), 1.92 – 1.86 (m, 2H, H^3), 1.74 – 1.71 (m, 2H, H^2), 1.32 (s, 6H, H^{13}); **^{13}C{^{1}H} NMR** (126 MHz, $CDCl_3$, 298 K): δ (ppm) = 143.3 (C^{12}), 134.4 (C^{11}), 129.6 (C^6), 123.9 (C^9), 120.9 (C^7), 112.4 (C^8), 111.9 (C^{10}), 108.8 (C^5), 39.3 (C^2), 31.7 (C^1), 29.4 (2C, C^{13}), 21.3 (C^4), 20.3 (C^3); **HR-MS** (ESI^+): m/z calculated for $[C_{14}H_{16}BrN+H]^+$: 278.0539 ($[M+H]^+$); found: 278.0538; **IR:** $\tilde{\nu}$ = 3423 (m, NH), 2953 (w, sp^3-CH), 2927 (w, sp^3-CH), 2860 (w, sp^3-CH), 2839 (w, sp^3-CH), 1572 (w, C=C), 1472 (m, C=C), 1451 (m, C=C), 1432 (m, C=C), 1363

(w, sp^3-CH_3), 1300 (s), 1274 (m), 1244 (w), 1205 (w), 1167 (w), 1049 (w), 943 (w), 921 (w), 911 (w), 871 (m), 788 (s), 729 (w), 704 (w) cm^{-1}.

2.1.14 6-Chloro-1,1-dimethyl-2,3,4,9-tetrahydro-1*H*-carbazole (SI-13)

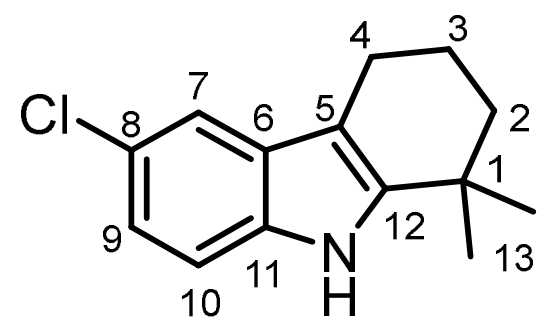

SI-13

Compound **SI-13** was synthesized following a modified procedure based on the method reported by *Lin* for a related analogue.[6]

To a solution of (4-chlorophenyl)hydrazine hydrochloride (1.00 g, 5.59 mmol, 1.00 equiv.) in 15 mL of acetic acid, 2,2-dimethylcyclohexanone (773 µL, 5.59 mmol, 1.00 equiv.) was added under argon atmosphere. The reaction mixture was stirred for 1 h at 70 °C and after that 30 min at 110 °C. The suspension was allowed to reach room temperature, and the solvent was largely removed using a rotary evaporator. Subsequently, the reaction mixture was neutralized with an aqueous saturated $NaHCO_3$ solution and organic and aqueous layers were separated. The aqueous layer was extracted with EtOAc (3 x 30 mL). The combined organic layers were washed with 30 mL of an aqueous saturated NaCl solution, dried over Na_2SO_4, filtered and concentrated *in vacuo*. Purification by column chromatography (silica, *n*-pentane/EtOAc = 97/3) yielded 633 mg (2.71 mmol, 49%) of **SI-13** as a yellow-orange solid.

Yellow-orange solid, 633 mg (49%); **TLC:** R_f = 0.29 (*n*-pentane/EtOAc, 97/3) [UV]; **^{1}H NMR** (500 MHz, $CDCl_3$, 298 K): δ (ppm) = 7.74 (s, 1H, NH), 7.41 (d, $^4J_{H-H}$ = 2.1 Hz, 1H, H^7), 7.19 (d, $^3J_{H-H}$ = 8.5 Hz, 1H, H^{10}), 7.06 (dd, $^3J_{H-H}$ = 8.5 Hz, $^4J_{H-H}$ = 2.1 Hz, 1H, H^9), 2.64 (t, $^3J_{H-H}$ = 6.2 Hz, 2H, H^4), 1.92-1.86 (m, 2H, H^3), 1.74-1.72 (m, 2H, H^2), 1.32 (s, 6H, H^{13}); **^{13}C{^{1}H} NMR** (126 MHz, $CDCl_3$, 298 K): δ (ppm) = 143.5 (C^{12}), 134.1 (C^{11}), 128.9 (C^6), 124.8 (C^8), 121.3 (C^9), 117.8 (C^7), 111.5 (C^{10}), 108.8 (C^5), 39.2 (C^2), 31.7 (C^1), 29.4 (C^{13}), 21.3 (C^4), 20.2 (C^3); **HR-MS** (ESI^+): *m/z* calculated for $[C_{14}H_{16}ClN+H]^+$: 234.1044 ($[M+H]^+$); found: 234.1044; **IR:** $\tilde{\nu}$ = 3426 (m, NH), 2964 (w, sp^3-CH), 2924 (w, sp^3-CH), 2859 (w, sp^3-CH), 2845 (w, sp^3-CH), 1695 (w, C=C), 1575 (w, C=C), 1472 (m, C=C), 1451 (m, C=C), 1434 (m, C=C), 1385 (w, sp^3-CH_3), 1362 (w, sp^3-CH_3), 1300 (m), 1273 (w), 1244 (w), 1232 (w), 1207 (w), 1168 (w),

1127 (w), 1103 (m), 1059 (m), 1029 (w), 982 (w), 938 (m), 912 (m), 878 (m), 865 (m), 858 (m), 850 (m), 839 (m), 792 (s), 737 (m), 706 (m), 660 (m) cm^{-1}.

2.1.15 6-Fluoro-1,1-dimethyl-2,3,4,9-tetrahydro-1*H*-carbazole (SI-14)

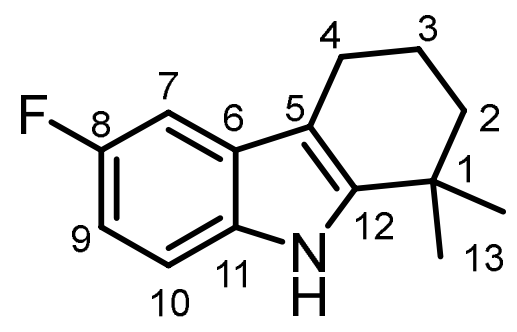

SI-14

Compound **SI-14** was synthesized following a modified procedure based on the method reported by *Lin* for a related analogue.[6]

(4-Fluorophenyl)hydrazine (1.00 g, 7.93 mmol, 1.00 equiv.) was dispersed in 8 mL of acetic acid. To this suspension, 2,2-dimethylcyclohexanone (1.21 mL, 8.72 mmol, 1.10 equiv.) was added under an argon atmosphere. The reaction mixture was then heated to 100 °C and stirred overnight. After allowing to cool to room temperature, the acetic acid was removed under reduced pressure. The residue was treated with water and dichloromethane (DCM), and the aqueous layer was extracted with DCM (3 × 30 mL). The combined organic layers were washed sequentially with saturated aqueous $NaHCO_3$ (2 × 30 mL) and saturated NaCl solution, dried over Na_2SO_4 and filtered. After evaporation of the solvents under vacuum, the crude product was purified by column chromatography (silica, *n*-pentane/Et_2O, 9/1) to afford 389 mg (1.79 mmol, 23%) of the desired product, a yellowish solid.

Pale yellow solid, 389 mg (23%); **TLC:** R_f = 0.75 (*n*-pentane/Et_2O, 8/2) [UV]; **^{1}H NMR** (500 MHz, $CDCl_3$, 298 K): δ (ppm) = 7.69 (s, 1H, N*H*), 7.19 (dd, $^3J_{H-H}$ = 8.7 Hz, $^4J_{H-F}$ = 4.3 Hz, 1H, H^{10}), 7.08 (dd, $^3J_{H-F}$ = 9.5 Hz, $^4J_{H-H}$ = 2.6 Hz, 1H, H^7), 6.85 (ddd, $^3J_{H-F}$ = 9.5 Hz, $^3J_{H-H}$ = 8.7 Hz, $^4J_{H-H}$ = 2.6 Hz, 1H, H^9), 2.63 (t, $^3J_{H-H}$ = 6.2 Hz, 2H, H^4), 1.92 – 1.86 (m, 2H, H^3), 1.74 – 1.71 (m, 2H, H^2), 1.32 (s, 6H, H^{13}); **^{13}C{^{1}H} NMR** (126 MHz, $CDCl_3$, 298 K): δ (ppm) = 157.9 (d, $^1J_{C-F}$ = 233.5 Hz, C^8), 144.0 (C^{12}), 132.3 (C^{11}), 128.2 (d, $^3J_{C-F}$ = 9.7 Hz, C^6), 111.0 (d, $^3J_{C-F}$ = 9.6 Hz, C^{10}), 109.3 (C^5), 109.1 (d, $^2J_{C-F}$ = 26.0 Hz, C^5), 103.4 (d, $^2J_{C-F}$ = 23.2 Hz, C^7), 39.3 (C^2), 31.7 (C^1), 29.4 (2C, C^{13}), 21.4 (C^4), 20.3 (C^3); **HR-MS** (ESI^+): *m/z* calculated for $[C_{14}H_{16}FN+H]^+$: 218.1340 ($[M+H]^+$); found: 218.1341; **IR:** $\tilde{\nu}$ = 3423 (m, NH), 2932 (w, sp^3-CH), 1582 (w, C=C), 1484 (m, C=C), 1456 (m, sp^3-CH), 1445 (s, sp^3-CH), 1305

(w), 1286 (w), 1167 (s), 1079 (w), 938 (m), 859 (m), 817 (m), 790 (s), 779 (m), 711 (w), 692 (w) cm^{-1}.

2.1.16 1,1-Dimethyl-2,3,4,9-tetrahydro-1*H*-carbazole-6-carboxylic acid (SI-15)

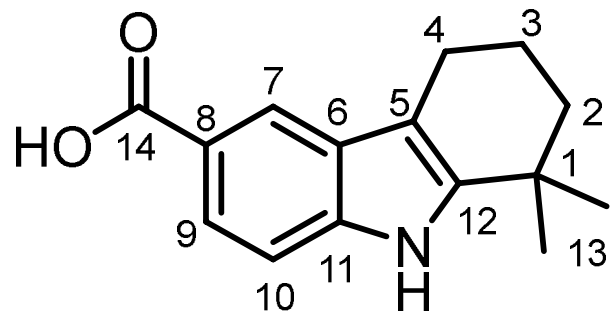

SI-15

A suspension of 4-hydrazineylbenzoic acid hydrochloride (1.00 g, 5.30 mmol, 1.00 equiv.) and 2,2-dimethylcyclohexanone (734 µL, 5.30 mmol, 1.00 equiv.) in acetic acid (15 mL) was stirred under argon atmosphere at 100 °C overnight. The dark reaction mixture was poured onto ice water to precipitate a solid. The solid was then collected by filtration and washed with little ice water. After drying *in vacuo* 907 mg (3.73 mmol, 70%) of **SI-15** was obtained in form of white/beige solid.

White/beige solid, 907 mg (70%); **TLC:** R_f = 0.11 (*n*-pentane/EtOAc, 8/2) [UV]; **^{1}H NMR** (500 MHz, DMSO-d_6, 298 K): δ (ppm) = 12.27 (s, 1H, OH), 11.07 (s, 1H, NH), 8.01 (d, $^4J_{H-H}$ = 1.8 Hz, 1H, H^7), 7.64 (dd, $^3J_{H-H}$ = 8.5 Hz, $^4J_{H-H}$ = 1.8 Hz, 1H, H^9), 7.30 (d, $^3J_{H-H}$ = 8.5 Hz, 1H, H^{10}), 2.61 (t, $^3J_{H-H}$ = 6.1 Hz, 2H, H^4), 1.85-1.80 (m, 2H, H^3), 1.69-1.66 (m, 2H, H^2), 1.29 (s, 6H, H^{13}); **^{13}C{^{1}H} NMR** (126 MHz, DMSO-d_6, 298 K): δ (ppm) = 168.7 (C^{14}), 143.9 (C^{12}), 138.5 (C^{11}), 126.6 (C^6), 121.8 (C^9), 120.5 (C^8), 120.2 (C^7), 110.3 (C^{10}), 108.2 (C^5), 39.0 (C^2), 31.4 (C^1), 28.9 (C^{13}), 20.9 (C^4), 20.0 (C^3); **HR-MS** (ESI^+): *m/z* calculated for $[C_{15}H_{17}NO_2+H]^+$: 244.1332 ($[M+H]^+$); found: 244.1331; **IR:** $\tilde{\nu}$ = 3398 (m, NH), 2933 (w, sp^3-CH), 2862 (w, sp^3-CH), 2839 (w, sp^3-CH), 1647 (s, C=O), 1619 (m, C=C), 1582 (w, C=C), 1571 (w, C=C), 1474 (m, C=C), 1443 (m, , C=C), 1418 (m, C-H), 1393 (m, sp^3-CH_3), 1365 (w, sp^3-CH_3), 1342 (w), 1315 (s), 1264 (s), 1248 (m), 1207 (m), 1163 (m), 1126 (w), 1074 (w), 1043 (w), 1029 (w), 987 (w), 944 (w), 926 (w), 905 (m), 877 (w), 851 (w), 842 (w), 816 (m), 786 (w), 773 (m), 747 (m), 723 (w), 705 (w) cm^{-1}.

2.1.17 1,1-Dimethyl-2,3,4,9-tetrahydro-1*H*-carbazole-6-carbonitrile (SI-16)

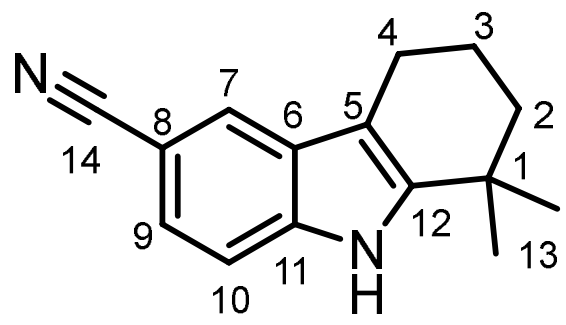

SI-16

A suspension of 4-hydrazineylbenzonitrile hydrochloride (1.00 g, 5.90 mmol, 1.00 equiv.) in 8 mL of acetic acid was prepared. Subsequently, 2,2-dimethylcyclohexanone (897 µL, 6.49 mmol, 1.10 equiv.) was added under an argon atmosphere. The resulting mixture was stirred at 100 °C overnight. After the acetic acid was removed under reduced pressure, water and dichloromethane were added. The aqueous phase was extracted with DCM (3 × 30 mL), and the combined organic extracts were washed with saturated aqueous $NaHCO_3$ (2 × 30 mL) and saturated NaCl solution, dried over Na_2SO_4 and filtered. Following evaporation of all volatiles under vacuum, the crude residue was purified by normal phase *Biotage* flash chromatography (*n*-hexane/ EtOAc) to yield 494 mg (2.20 mmol, 37%) of **SI-16** as a beige solid.

Beige solid, 494 mg (37%); **TLC:** R_f = 0.41 (*n*-pentane/EtOAc, 8/2) [UV]; **^{1}H NMR** (500 MHz, $CDCl_3$, 298 K): δ (ppm) = 8.05 (s, 1H, NH), 7.78 (d, $^4J_{H-H}$ = 1.5 Hz, 1H, H^7), 7.36 (dd, $^3J_{H-H}$ = 8.4 Hz, $^4J_{H-H}$ = 1.5 Hz, 1H, H^9), 7.32 (d, $^3J_{H-H}$ = 8.4 Hz, 1H, H^{10}), 2.67 (t, $^3J_{H-H}$ = 6.2 Hz, 2H, H^4), 1.94-1.88 (m, 2H, H^3), 1.76-1.73 (m, 2H, H^2), 1.34 (s, 6H, H^{13}); **^{13}C{^{1}H} NMR** (126 MHz, $CDCl_3$, 298 K): δ (ppm) = 144.3 (C^{12}), 137.6 (C^{14}), 127.8 (C^6), 124.5 (C^9), 123.8 (C^7), 121.3 (C^{11}), 111.3 (C^{10}), 109.9 (C^5), 102.2 (C^8), 39.1 (C^2), 31.7 (C^1), 29.3 (C^{13}), 21.2 (C^4), 20.1 (C^3); **HR-MS** (ESI^+): *m/z* calculated for $[C_{15}H_{16}N_2+H]^+$: 225.1386 ($[M+H]^+$); found: 225.1387; **IR:** $\tilde{\nu}$ = 3306 (m, NH), 2937 (w, sp^3-CH), 2847 (w, sp^3-CH), 2220 (s, CN), 1619 (w, C=C), 1580 (w, C=C), 1471 (m, C=C), 1455 (m, sp^3-CH), 1389 (w, sp^3-CH), 1365 (w,), 1319 (m), 1295 (w), 1276 (w), 1254 (w), 1240 (w), 1206 (w), 1166 (w), 1133 (w), 1048 (w), 1028 (w), 925 (w), 881 (w), 872 (m), 851 (w), 808 (s), 752 (w), 717 (w), 687 (w) cm^{-1}.

2.1.18 3,3-Dimethyl-1,2,3,4-tetrahydrocyclopenta[*b*]indole (SI-17)

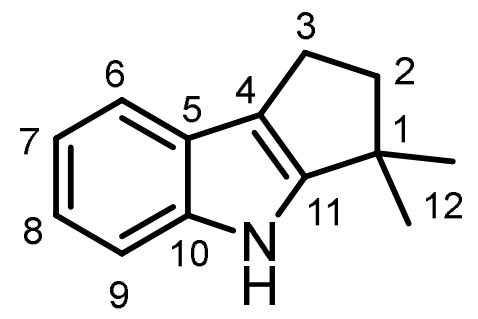

SI-17

Acetic acid (10 mL) was placed in a 50 mL round-bottom flask. Afterwards 878 µL (8.91 mmol, 1.00 equiv.) of phenylhydrazine and 1.12 mL (8.91 mmol, 1.00 equiv.) of 2,2-dimethylcyclopentanone were added and the reaction mixture was stirred at 110 °C for 2 h. Subsequently, acetic acid was removed under reduced pressure and water, an aqueous saturated $NaHCO_3$ solution and DCM were added. The resulting layers were separated, and the aqueous layer was extracted with DCM (3 x 30 mL). The combined organic layers were washed with 30 mL of an aqueous saturated NaCl solution, dried over Na_2SO_4 and filtered. Purification by column chromatography (silica, *n*-pentane/EtOAc = 9/1) yielded 457 mg (2.47 mmol, 28%) of the target compound as red-orange solid.

Red-orange solid, 457 mg (28%); **TLC:** R_f = 0.64 (*n*-pentane/EtOAc, 9/1) [UV]; **^{1}H NMR** (500 MHz, $CDCl_3$, 298 K): δ (ppm) = 7.71 (s, 1H, NH), 7.49-7.41 (m, 1H, H^6), 7.35-7.28 (m, 1H, H^9), 7.15-7.03 (m, 2H, $H^{7,8}$), 2.84 - 2.80 (m, 2H, H^3), 2.37-2.34 (m, 2H, H^2), 1.34 (s, 6H, H^{12}); **^{13}C{^{1}H} NMR** (126 MHz, $CDCl_3$, 298 K): δ (ppm) = 151.2 (C^{11}), 140.7 (C^{10}), 125.0 (C^5), 120.6 (C^7/C^8), 119.7 (C^7/C^8), 118.8 (C^6), 117.0 (C^4), 111.6 (C^9), 45.9 (C^2), 39.5 (C^1), 28.0 (C^{12}), 23.0 (C^3); **HR-MS** (ESI^+): *m/z* calculated for $[C_{13}H_{15}N+H]^+$: 186.1277 ($[M+H]^+$); found: 186.1277; **IR:** $\tilde{\nu}$ = 3383 (m, NH), 2953(w, sp^3-CH), 2941 (w, sp^3-CH), 2855 (w, sp^3-CH), 1472 (w, C=C), 1454 (m, C=C), 1445 (w, C=C), 1431 (w, C=C), 1363 (w, sp^3-CH_3), 1310 (s), 1294 (w), 1237 (w), 1139 (w), 1073 (w), 1009 (w), 994 (w), 747 (s), 696 (m) cm^{-1}.

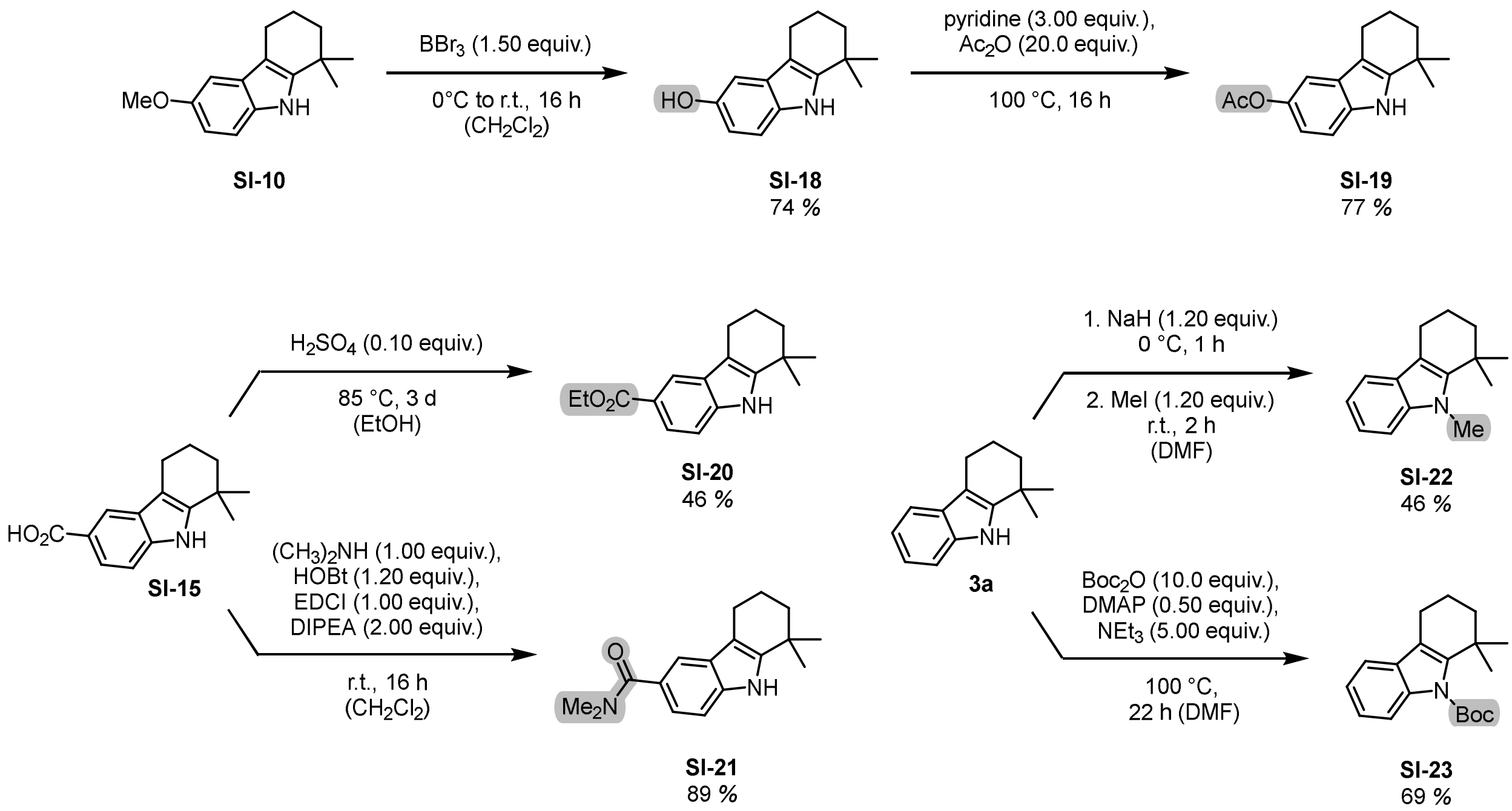

Figure SI-4. Synthesis of various other substrates based on functional group interconversions.

2.1.19 1,1-Dimethyl-2,3,4,9-tetrahydro-1*H*-carbazol-6-ol (SI-18)

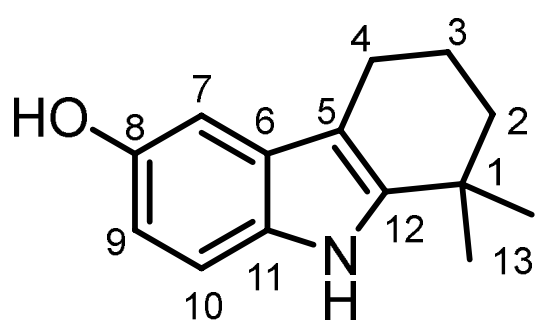

SI-18

200 mg (872 µmol, 1.00 equiv.) of 6-methoxy-1,1-dimethyl-2,3,4,9-tetrahydro-1H-carbazole (**SI-10**) were added to a *Schlenk* tube, which was evacuated and refilled with argon three times. 3.50 mL of DCM was added to dissolve the starting material. After the solution was cooled to 0 °C, 1.31 mL (1.31 mmol, 1.50 equiv.) of boron tribromide was added slowly and the rection mixture stirred at 0 °C for 30 min, then at room temperature overnight. The reaction mixture was quenched by adding water and neutralized with an aqueous saturated $NaHCO_3$ solution. The aqueous layer was then extracted with DCM (3 x 30 mL). The combined organic layers were washed with 30 mL of an aqueous saturated NaCl solution, dried over Na_2SO_4 and filtered. Removal of the solvent under reduced pressure gave a dark crude product. Purification by column chromatography (silica, *n*-pentane/EtOAc = 7/3) yielded 140 mg (649 µmol, 74%) the target compound **SI-18** as pale grey solid.

Pale grey solid, 140 mg (74%); **TLC:** R_f = 0.51 (*n*-pentane/EtOAc, 7/3) [UV]; **^{1}H NMR** (500 MHz, $CDCl_3$, 298 K): δ (ppm) = 7.60 (br s, 1H, N*H*), 7.14 (d, $^3J_{H\text{-}H}$ = 8.5 Hz, 1H, H^{10}), 6.86 (d, $^4J_{H\text{-}H}$ = 2.5 Hz, 1H, H^7), 6.68 (dd, $^3J_{H\text{-}H}$ = 8.5 Hz, $^4J_{H\text{-}H}$ = 2.5 Hz, 1H, H^9), 4.44 (br s, 1H, O*H*), 2.61 (t, $^3J_{H\text{-}H}$ = 6.2 Hz, 2H, H^4), 1.90 – 1.86 (m, 2H, H^3), 1.73 – 1.70 (m, 2H, H^2), 1.31 (s, 6H, H^{13}); **^{13}C{^{1}H} NMR** (126 MHz, $CDCl_3$, 298 K): δ (ppm) = 149.3 (C^8), 143.3 (C^{12}), 131.0 (C^{11}), 128.5 (C^6), 111.1 (C^{10}), 110.4 (C^9), 108.5 (C^5), 103.1 (C^7), 39.3 (C^2), 31.7 (C^1), 29.4 (2C, C^{13}), 21.45 (C^4), 20.3 (C^3); **HR-MS** (ESI$^+$): *m/z* calculated for $[C_{14}H_{17}NO+H]^+$: 216.1383 ($[M+H]^+$); found: 216.1382; **IR:** $\tilde{\nu}$ = 3425 (m, NH), 3301 (b, OH), 2961 (w, sp^3-CH), 2927 (w, sp^3-CH), 2840 (w, sp^3-CH), 1623 (w, C=C), 1591 (w, C=C), 1488 (w, C=C), 1475 (w, C=C), 1460 (s, sp^3-CH_3), 1440 (w, sp^3-CH_3), 1380 (w), 1361 (w), 1306 (w), 1250 (w), 1234 (w), 1207 (m), 1191 (s), 1168 (m), 1081 (w), 938 (w), 931 (w), 853 (w), 846 (m), 820 (m), 800 (w), 783 (w), 739 (w), 705 (m), 695 (m) cm^{-1}.

2.1.20 1,1-Dimethyl-2,3,4,9-tetrahydro-1*H*-carbazol-6-yl acetate (SI-19)

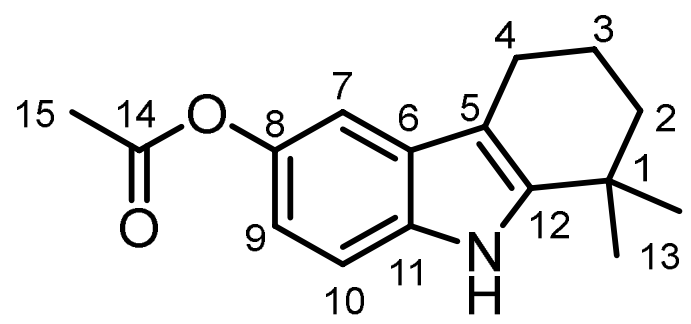

SI-19

135 mg (627 µmol, 1.00 equiv.) of 1,1-dimethyl-2,3,4,9-tetrahydro-1*H*-carbazol-6-ol (**SI-18**) were dissolved in 152 µL (1.88 mmol, 3.00 equiv.) pyridine. To the stirred solution 1.19 mL (12.5 mmol, 20.0 equiv.) of acetic anhydride were added and the reaction mixture was stirred at 100 °C overnight. After cooling to room temperature, the reaction mixture was extracted with EtOAc (3 x 30 mL) and the combined organic layers were washed with 30 mL of an aqueous saturated NaCl solution, dried over Na_2SO_4 and filtered. The crude product was purified by azeotropic distillation with toluene. The crude residue was dissolved in a minimal amount of DCM, followed by the addition of approximately 30 mL of toluene. The solvent was then removed under reduced pressure using a rotary evaporator. This procedure was repeated three times in total resulting in 125 mg (485 µmol, 77%) of the target compound as a slightly brown solid.

Slightly brown solid, 125 mg (77%); **TLC:** R_f = 0.45 (*n*-pentane/EtOAc, 8/2) [UV]; **^{1}H NMR** (500 MHz, $CDCl_3$, 298 K): δ (ppm) = 7.73 (s, 1H, NH), 7.24 (d, $^3J_{H\text{-}H}$ = 8.6 Hz, 1H,

H^{10}), 7.13 (d, $^4J_{H-H}$ = 2.3 Hz, 1H, H^7), 6.81 (dd, $^3J_{H-H}$ = 8.6 Hz, $^4J_{H-H}$ = 2.3 Hz, 1H, H^9), 2.63 (t, $^3J_{H-H}$ = 6.2 Hz, 2H, H^4), 2.31 (s, 3H, H^{15}), 1.89 – 1.87 (m, 2H, H^3), 1.73 – 1.71 (m, 2H, H^2), 1.31 (s, 6H, H^{13}); **^{13}C{^{1}H} NMR** (126 MHz, $CDCl_3$, 298 K): δ (ppm) = 170.7 (C^{14}), 144.2 (C^8), 143.5 (C^{12}), 133.7 (C^{11}). 128.2 (C^6), 115.0 (C^9), 110.9 (C^{10}), 110.6 (C^7), 109.4 (C^5), 39.3 (C^2), 31.7 (C^1), 29.4 (2C, C^{13}), 21.4 (C^4), 21.3 (C^{15}), 20.3 (C^3); **HR-MS** (ESI^+): *m/z* calculated for $[C_{16}H_{19}NO_2+H]^+$: 258.1489 ($[M+H]^+$); found: 258.1490; **IR:** $\tilde{\nu}$ = 3399 (m, NH), 2956 (w, sp^3-CH), 2910 (w, sp^3-CH), 1739 (s, C=O), 1484 (w, C=C), 1461 (w, C=C), 1447 (w, sp^3-CH), 1369 (m), 1313 (w), 1249 (m), 1237 (s, C-O-C), 1150 (m), 1118 (w), 1080 (w), 1042 (w), 1014 (w), 988 (w), 946 (w), 926 (w), 912 (w), 847 (w), 820 (w), 800 (w), 782 (w), 705 (w), 659 (w) cm^{-1}.

2.1.21 Ethyl 1,1-dimethyl-2,3,4,9-tetrahydro-1*H*-carbazole-6-carboxylate (SI-20)

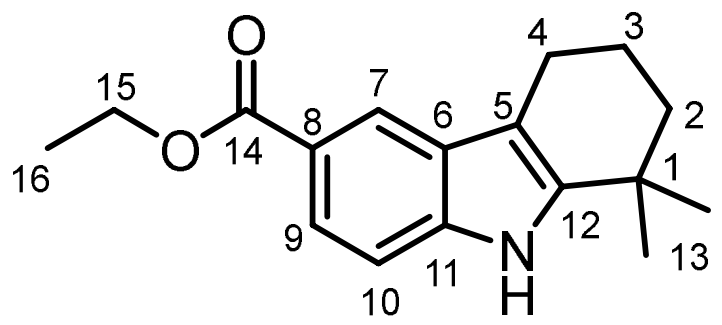

SI-20

860 mg (3.53 mmol, 1.00 equiv.) of 1,1-dimethyl-2,3,4,9-tetrahydro-1H-carbazole-6-carboxylic acid (**SI-15**) were dissolved in 25.3 mL (3.53 mmol) of ethanol. To the reaction solution 18.9 µL (353 µmol, 0.10 equiv.) of sulfuric acid was added. The reaction mixture was stirred under reflux and argon atmosphere at 85 °C over the weekend. Ethanol was removed under reduced pressure, the reaction mixture was neutralized with aqueous saturated $NaHCO_3$ solution and water and dichloromethane (DCM) were added. The aqueous layer was extracted with DCM (3 x 30 mL), the combined organic layers were washed with 30 mL of an aqueous saturated NaCl solution, dried over Na_2SO_4 and filtered. Purification by column chromatography (silica, *n*-pentane/Et_2O = 9/1, after first fraction 8/2) and removal of all volatiles *in vacuo* resulted in 443 mg (1.63 mmol, 46%) of **SI-20**, a white-yellowish solid.

Pale yellow solid, 443 mg (46%); **TLC:** R_f = 0.20 (*n*-pentane/Et_2O, 9/1) [UV]; **TLC:** R_f = 0.11 (P/Et_2O, 8/2) [UV]; **^{1}H NMR** (500 MHz, $CDCl_3$, 298 K): δ (ppm) = 8.22 (d, $^4J_{H-H}$ = 1.8 Hz, 1H, H^7), 7.69 (s, 1H, NH), 7.84 (dd, $^3J_{H-H}$ = 8.5 Hz, $^4J_{H-H}$ = 1.8 Hz, 1H, H^9), 7.28 (dd, $^3J_{H-H}$ = 8.5 Hz, $^4J_{H-H}$ = 0.8 Hz, 1H, H^{10}), 4.39 (q, $^3J_{H-H}$ = 7.1 Hz, 2H, H^{15}), 2.71 (t, $^3J_{H-H}$ = 6.2 Hz, 2H, H^4), 1.94-1.88 (m, 2H, H^3), 1.76-1.73 (m, 2H, H^2), 1.42 (t, $^3J_{H-H}$ = 7.1 Hz, 3H, H^{16}), 1.33 (s,

6H, H^{13}); **^{13}C{^{1}H} NMR** (126 MHz, $CDCl_3$, 298 K): δ (ppm) = 168.1 (C^{14}), 143.2 (C^{12}), 138.5 (C^{11}), 127.5 (C^{6}), 122.8 (C^{9}), 121.5 (C^{8}), 121.2 (C^{7}), 110.3 (C^{5}), 110.1 (C^{10}), 60.6 (C^{15}), 39.2 (C^{2}), 31.6 (C^{1}), 29.3 (C^{13}), 21.4 (C^{4}), 20.3 (C^{3}), 14.6 (C^{16}); **HR-MS** (ESI^+): *m/z* calculated for $[C_{17}H_{21}NO_2+H]^+$: 272.1645 ($[M+H]^+$); found: 272.1644; **IR:** $\tilde{\nu}$ = 3294 (m, NH), 2949 (w, sp^3-CH), 2923 (w, sp^3-CH), 1692 (s, C=O), 1618 (m, C=C), 1476 (w, C=C), 1464 (w, C=C), 1455 (w, C=C), 1364 (m, sp^3-CH_3), 1321 (m), 1287 (s), 1273 (s), 1245 (w), 1203 (w), 1187 (w), 1165 (w), 1133 (m), 1114 (w), 1028 (m), 932 (w), 905 (w), 768 (m), 742 (m), 728 (m), 712 (m) cm^{-1}.

2.1.22 *N,N*,1,1-Tetramethyl-2,3,4,9-tetrahydro-1*H*-carbazole-6-carboxamide (SI-21)

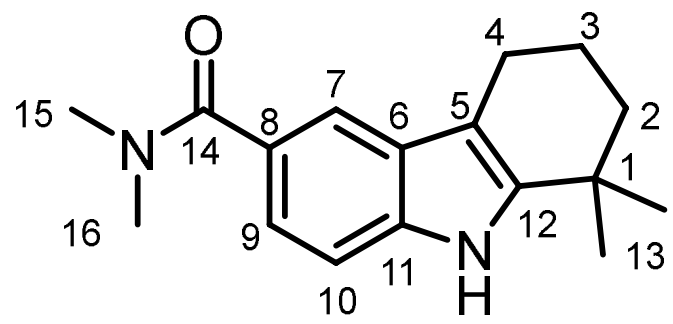

SI-21

1,1-Dimethyl-2,3,4,9-tetrahydro-1*H*-carbazole-6-carboxylic acid (402 mg, 1.65 mmol, 1.00 equiv.) (**SI-15**) was dissolved in a solvent mixture of 15 mL of DCM and 10 mL of ethanol. 297 µL (1.65 mmol, 1.00 equiv.) of dimethylamine, 268 mg (1.98 mmol, 1.20 equiv.) of hydroxybenzotriazole (HOBt), 317 mg (1.65 mmol, 1.00 equiv.) of 1-ethyl-3-(3-dimethylaminopropyl)carbodiimide (EDCI) and 576 µL (3.30 mmol, 2.00 equiv.) of *N*,*N*-diisopropylethylamine (DIPEA) were added one after another to the reaction mixture and stirred at room temperature overnight. The aqueous layer was extracted with citric acid (3 x 30 mL) and the combined organic layers were washed with 30 mL of an aqueous saturated NaCl solution, dried over Na_2SO_4 and filtered. After removal of all volatiles under reduced pressure, the crude product was purified by column chromatography (silica, *n*-pentane/ EtOAc = 5/5), resulting in 397 mg (1.47 mmol, 89%) of **SI-21** in form of pale-yellowish crystalline solid.

Pale yellow solid, 397 mg (89%); **TLC:** R_f = 0.20 (*n*-pentane/EtOAc, 5/5) [UV]; **^{1}H NMR** (500 MHz, $CDCl_3$, 298 K): δ (ppm) = 7.92 (s, 1H, N*H*), 7.57 (d, $^{4}J_{H\text{-}H}$ = 1.7 Hz, 1H, H^7), 7.26 (dd, $^{3}J_{H\text{-}H}$ = 8.3 Hz, $^{4}J_{H\text{-}H}$ = 0.7 Hz, 1H, H^{10}), 7.19 (dd, $^{3}J_{H\text{-}H}$ = 8.3 Hz, $^{4}J_{H\text{-}H}$ = 1.7 Hz, 1H, H^9), 3.08 (br s, 6H, $H^{15,16}$), 2.67 (t, $^{3}J_{H\text{-}H}$ = 6.2 Hz, 2H, H^4), 1.92 – 1.86 (m, 2H, H^3), 1.75 – 1.72 (m, 2H,

H^2), 1.33 (s, 6H, H^{13}); **^{13}C{^{1}H} NMR** (126 MHz, $CDCl_3$, 298 K): δ (ppm) = 173.4 (C^{14}), 143.0 (C^{12}), 136.3 (C^{11}), 127.2 (C^8), 127.1 (C^6), 120.8 (C^9), 118.1 (C^7), 110.1 (C^{10}), 109.5 (C^5), 40.2 ($C^{15/16}$), 39.3 (C^2), 35.8 ($C^{15/16}$), 31.7 (C^1), 29.4 (C^{13}), 21.4 (C^4), 20.3 (C^3); **HR-MS** (ESI^+): *m/z* calculated for $[C_{17}H_{22}N_2O+H]^+$: 271.1805 ($[M+H]^+$); found: 271.1802; **IR:** $\tilde{\nu}$ = 3186 (w, sp^2-CH), 3163 (w, sp^2-CH), 2956 (w, sp^3-CH), 2925 (w, sp^3-CH), 2837 (w, sp^3-CH), 1740 (s, C=O), 1601 (w, C=C), 1564 (w, C=C), 1490 (w, C=C), 1483 (w, C=C), 1455 (w, C=C), 1435 (w, sp^3-CH_3), 1392 (m, sp^3-CH_3), 1364 (w, sp^3-CH_3), 1312 (m, C-N), 1280 (w), 1263 (w), 1234 (w), 1204 (w), 1192 (w), 1167 (w), 1098 (w), 1063 (w), 1051 (w), 899 (w), 806 (w), 761 (m), 738 (m), 684 (w) cm^{-1}.

2.1.23 1,1,9-Trimethyl-2,3,4,9-tetrahydro-1*H*-carbazole (SI-22)

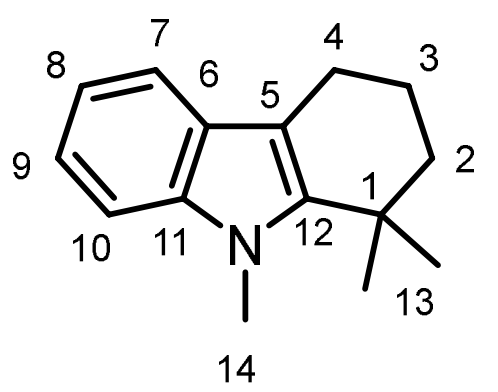

SI-22

Compound **SI-22** was prepared according to a modified literature procedure.[7] The compound is known in the literature.[8]

In a *Schlenk* flask, 1,1-dimethyl-2,3,4,9-tetrahydro-1*H*-carbazole (**3a**, 500 mg, 2.51 mmol, 1.00 equiv.) was dissolved in DMF (10 mL, 250 mM) and cooled to 0 °C with an ice bath. Sodium hydride (60% dispersion in mineral oil, 120 mg, 3.01 mmol, 1.20 equiv.) was added in two portions and the reaction was allowed to stir for one hour at 0°C. Then, methyl iodide (187 µL, 427 mg, 3.01 mmol, 1.20 equiv.) was added and the mixture was allowed to come to room temperature and stirred for additional 2 h. Afterwards, an aqueous ammonia solution (25%, 5 mL) was added to quench remaining MeI and the mixture was further diluted with water (30 mL). The aqueous layer was extracted with diethyl ether (3 x 50 mL) and the combined organic layers washed with 200 mL of an aqueous saturated NaCl solution, dried over Na_2SO_4 and filtered. Purification by column chromatography (silica, *n*-pentane/EtOAc = 9/1) yielded 473 mg (2.22 mmol, 88%) of **SI-22** as a colorless solid.

Colorless solid, 473 mg (88%); **TLC** (*n*-pentane/EtOAc, 9/1): R_f = 0.83 [UV]; **^{1}H NMR** (500 MHz, $CDCl_3$, 298 K): δ (ppm) = 7.50 (dd, $^3J_{H-H}$ = 7.8 Hz, $^4J_{H-H}$ = 1.0 Hz, 1H, H^7), 7.28 (dd,

$^{3}J_{H\text{-}H}$ = 8.1 Hz, $^{4}J_{H\text{-}H}$ = 0.9 Hz, 1H, H^{10}), 7.21 (ddd, $^{3}J_{H\text{-}H}$ = 8.1, 7.0 Hz, $^{4}J_{H\text{-}H}$ = 1.2 Hz, 1H, H^{9}), 7.11 (ddd, $^{3}J_{H\text{-}H}$ = 7.8, 7.0 Hz, $^{4}J_{H\text{-}H}$ = 1.1 Hz, 1H, H^{8}), 3.84 (s, 3H, H^{14}), 2.75 (t, $^{3}J_{H\text{-}H}$ = 6.1 Hz, 2H, H^{4}), 1.95 – 1.85 (m, 2H, H^{3}), 1.82 – 1.76 (m, 2H, H^{2}), 1.46 (s, 6H, H^{13}); **^{13}C{^{1}H} NMR** (126 MHz, $CDCl_3$, 298 K): δ (ppm) = 142.2 (C^{12}), 137.5 (C^{11}), 126.9 (C^{6}), 121.0 (C^{9}), 118.8 (C^{7}), 118.1 (C^{7}), 109.3 (C^{5}), 108.5 (C^{10}), 42.9 (C^{2}), 32.5 (C^{1}), 32.0 (C^{14}), 28.5 (C^{13}), 22.2 (C^{4}), 20.5 (C^{3}); **HR-MS** (ESI^+): *m/z* calculated for $[C_{15}H_{19}N+H]^+$: 214.1590 ($[M+H]^+$); found: 214.1587; **IR:** $\tilde{\nu}$ = 3054 (w, sp^2-CH), 2924 (s, sp^3-CH), 2856 (m, sp^3-CH), 1645 (w, C=C), 1610 (w, C=C), 1566 (w, C=C), 1468 (s), 1438 (m), 1359 (s), 1329 (w), 1276 (m), 1240 (s), 1179 (m), 1124 (w), 811 (w), 729 (s) cm^{-1}.

2.1.24 *tert*-Butyl 1,1-dimethyl-1,2,3,4-tetrahydro-9*H*-carbazole-9-carboxylate (SI-23)

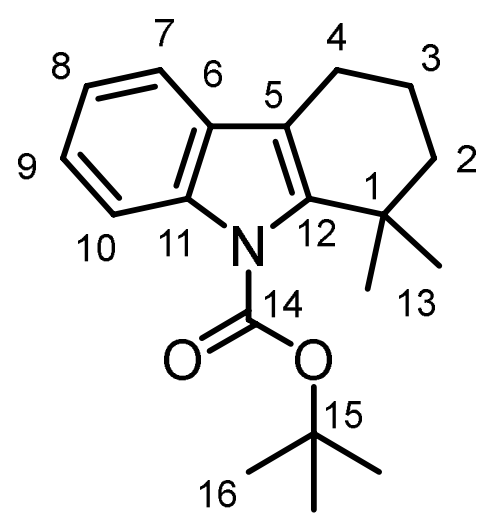

SI-23

In a 50 mL *Schlenk* flask, 1,1-dimethyl-2,3,4,9-tetrahydro-1*H*-carbazole (**3a**, 1.07 g, 5.36 mmol, 1.00 equiv.) and 4-dimethylaminopyridine (327 mg, 2.68 mmol, 0.50 equiv.) were added to a solution of di-*tert*-butyl-dicarbonate (11.5 mL, 53.6 mmol, 10.0 equiv.) and triethylamine (3.71 mL, 26.8 mmol, 5.00 equiv.) in DMF (27 mL, 200 mM). The reaction mixture was stirred for 22 h at 100 °C. The reaction was allowed to cool to room temperature before being diluted with a saturated brine wash (15 mL) and extracted with EtOAc (2×25 mL). The combined organic layers were dried over Na_2SO_4. After filtration and removal of all volatiles under reduced pressure the crude material was purified by flash column chromatography (*n*-pentane/Et_2O = 95/5) to obtain **SI-23** (1.10 g, 3.67 mmol, 69%).

Colorless oil, 1.10 g (69%); **TLC:** R_f = 0.92 (*n*-pentane/Et_2O, 9/1) [UV]; **^{1}H NMR** (500 MHz, $CDCl_3$, 298 K): δ (ppm) = 7.90 (dd, $^{3}J_{H\text{-}H}$ = 8.4 Hz, $^{4}J_{H\text{-}H}$ = 0.9 Hz, 1H, H^{10}), 7.41 – 7.36 (m, 1H, H^{7}), 7.22 (ddd, $^{3}J_{H\text{-}H}$ = 8.4, 7.2 Hz, $^{4}J_{H\text{-}H}$ =1.5 Hz, 1H, H^{9}), 7.19 – 7.15 (m,

1H, H^8), 2.65 (t, $^3J_{H\text{-}H}$ = 6.3 Hz, 2H, H^4), 1.95 – 1.87 (m, 2H, H^3), 1.70 (s, 9H, H^{16}), 1.70 – 1.66 (m, 2H, H^2), 1.50 (s, 6H, H^{13}); **^{13}C{^{1}H} NMR** (126 MHz, $CDCl_3$, 298 K): δ (ppm) = 151.1 (C^{14}), 143.0 (C^{12}), 136.4 (C^{11}), 129.4 (C^6), 123.6 (C^9), 122.1 (C^{18}), 118.1 (C^7), 116.8 (C^5), 115.0 (C^{10}), 83.5 (C^{15}), 43.8 (C^2), 33.8 (C^1), 28.4 (C^{16}), 27.5 (C^{13}), 22.2 (C^4), 19.0 (C^3); **IR:** $\tilde{\nu}$ = 3051 (w, sp^2-CH), 3005 (w, sp^2-CH), 2961 (m, sp^3-CH), 2938 (m, sp^3-CH), 2867 (w, sp^3-CH), 1734 (s, C=O), 1609 (w, C=C), 1571 (w, C=C), 1479 (m), 1455 (s), 1368 (m), 1308 (s), 1254 (m), 1219 (m), 1147 (m), 1104 (m), 1055 (m), 1009 (m), 844 (m), 776 (m), 740 (s), 712 (m), 682 (m).

2.1 Flavin Synthesis

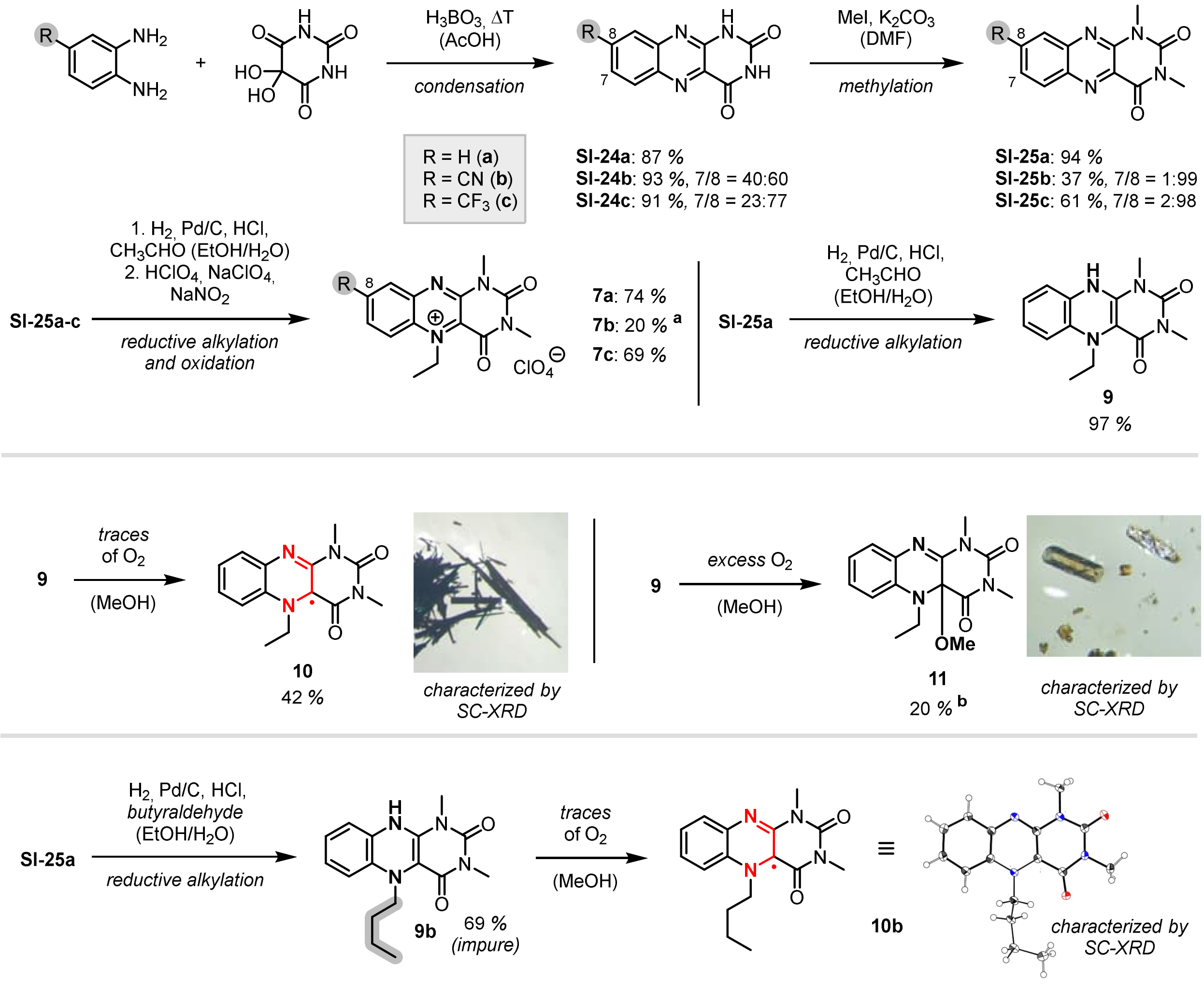

Figure SI-5. Catalyst synthesis of alloxazinium perchlorates **7a-c** and the preparation of reduced alloxazine **9**. Exposure of hydroquinone **9** to trace oxygen in methanol leads to the formation of crystalline flavin semiquinone **10** (dark needles, bottom left). Prolonged exposure of the same solution to air results in the formation of single crystals of the methanol adduct **11** (bottom right). Similarly, butylated derivate **9b** can be oxidized to radical **10b**, as confirmed by SC-XRD.

[a] Reductive alkylation on Pd/C could not be used for the preparation of **7b** with an easily reducible cyano group. Therefore, milder conditions with 1. $Na_2S_2O_4$, $NaBH_3CN$, AcOH, H_3CHO (DMF) were chosen instead. [b] The isolated yield of 20% is low because only the formed single crystals were collected, while the remaining concentrated solution was not further worked up. As NMR studies of **9** in aerated CD_3OD show (Section 7.1 NMR studies on the aerobic oxidation of reduced flavins), the conversion to **11** is, in fact, nearly quantitative.

2.2.1 Alloxazine (SI-24a)

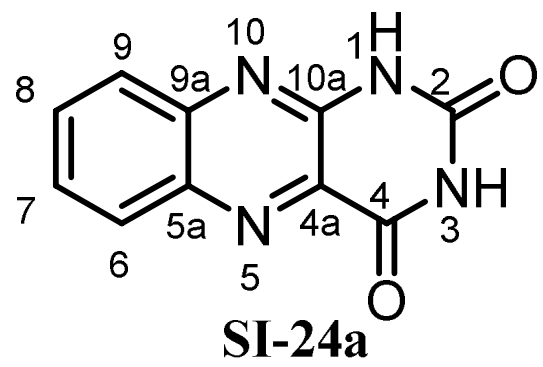

SI-24a

Compound **SI-24a** was prepared *via* a modified literature procedure.[9]

In a 250 mL round-bottom flask, *o*-phenylenediamine (1.00 g, 9.25 mmol, 1.00 equiv.) was dissolved in HOAc (20 mL). In a separate 100 mL round-bottom flask, a solution of alloxan (1.31 g, 9.25 mmol, 1.00 equiv.) and H_3BO_3 (0.63 g, 10.3 mmol, 1.11 equiv.) in HOAc (55 mL) was prepared at 90 °C. After complete dissolution, the light green solution from the 100 mL flask was added to the solution in the 250 mL flask. The reaction mixture was stirred at room temperature for 3 h. The solid formed was collected by filtration and washed with HOAc (3 × 10 mL), followed by Et_2O (3 × 15 mL). After drying the solid *in vacuo*, the desired compound **SI-24a** was obtained as a yellow solid (1.73 g, 7.97 mmol, 87%).

Yellow solid, 1.73 g (87%); **^{1}H NMR** (500 MHz, DMSO-d$_6$, 298 K): δ (ppm) = 11.97 (d, $^4J_{H-H}$ = 2.0 Hz, 1H, N*H*), 11.78 (d, $^4J_{H-H}$ = 2.0 Hz, 1H, N*H*), 8.16 (*virt.* dt, $^3J_{H-H} \approx {}^3J_{H-H}$ = 8.4 Hz, $^4J_{H-H}$ = 1.1 Hz, 1H, $H^{6/9}$), 7.97 – 7.88 (m, 2H, $H^{6/7/8/9}$), 7.78 (m, 1H, $H^{7/8}$); **^{13}C{^{1}H} NMR** (126 MHz, DMSO-*d*$_6$, 298 K): δ (ppm) = 160.6 ($C^{2/4/10a}$), 150.2 ($C^{2/4/10a}$), 146.9 ($C^{2/4/10a}$), 142.6 ($C^{9a/5a}$), 139.2 ($C^{9a/5a}$), 133.4 ($C^{6/7/8/9}$), 131.8 (C^{4a}), 130.2 ($C^{6/9}$), 128.5 ($C^{7/8}$), 127.0 ($C^{6/7/8/9}$); **HR-MS** (ESI$^+$): *m/z* calculated for $[C_{10}H_6N_4O_2+H]^+$: 215.0564 ($[M+H]^+$); found: 215.0569; **IR:** $\tilde{\nu}$ = 3175 (w, NH), 3086 (w, sp^2-CH), 2844 (w, sp^3-CH), 1787 (w), 1735 (s, C=O), 1689 (s, C=O), 1619 (m, C=C), 1584 (m, C=C), 1506 (m, C=C), 1486 (m), 1449 (m), 1392 (m), 1365 (m), 1335 (m), 1273 (s), 1249 (m), 1214 (m), 1154 (m), 1036 (m), 1015 (m), 990 (m), 916 (m), 868 (m, sp^2-CH), 812 (m, sp^2-CH), 768 (s, sp^2-CH), 706 (m, sp^2-CH) cm^{-1}.

2.2.2 1,3-Dimethyl alloxazine (SI-25a)

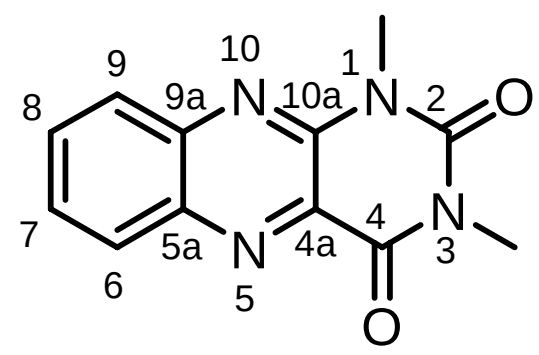

SI-25a

Compound **SI-25a** was prepared *via* a modified literature procedure.[10]

Alloxazine (**SI-24a**, 2.00 g, 9.34 mmol, 1.00 equiv.) and K_2CO_3 (6.71 g, 48.6 mmol, 5.20 equiv.) were evacuated in a 100 mL *Schlenk* flask. The flask was put under argon and the solids were suspended in dry DMF (32 mL). MeI (3.98 g, 1.74 mL, 28.0 mmol, 3.00 equiv.) was added in two portions. The reaction mixture was stirred at room temperature overnight. The mixture was then quenched with a conc. aqueous ammonia solution (25 ml, 25%) and stirred for an additional 30 minutes at room temperature. The solids were filtered and washed with water (5x, to make sure that all the DMF is removed). The yellow powder was dried on the *Schlenk* line to afford 2.13 g (8.78 mmol, 94%) of dimethylalloxazine (**SI-25a**) as a bright yellow powder.

Yellow powder, 2.13 g (8.78 mmol, 94%); **TLC** (CH_2Cl_2/MeOH, 95/5): R_f = 0.84 [UV]; **^{1}H NMR** (400 MHz, $CDCl_3$, 298 K): δ (ppm) = 8.35 (ddd, $^3J_{H\text{-}H}$ = 8.5 Hz, $^4J_{H\text{-}H}$ = 1.5 Hz, $^5J_{H\text{-}H}$ = 0.6 Hz, 1H, H^6), 8.04 (ddd, $^3J_{H\text{-}H}$ = 8.5 Hz, $^4J_{H\text{-}H}$ = 1.4 Hz, $^5J_{H\text{-}H}$ = 0.6 Hz, 1H, H^9), 7.90 (ddd, $^3J_{H\text{-}H}$ = 8.5, 6.9 Hz, $^4J_{H\text{-}H}$ = 1.5 Hz, 1H, H^8), 7.76 (ddd, $^3J_{H\text{-}H}$ = 8.5, 6.9 Hz, $^4J_{H\text{-}H}$ = 1.4 Hz, 1H, H^7), 3.84 (s, 3H, N^1-CH_3), 3.61 (s, 3H, N^3-CH_3); **^{13}C{^{1}H} NMR** (101 MHz, $CDCl_3$, 298 K): δ (ppm) = 160.0 (C^4), 150.9 (C^2), 145.5 (C^{10a}), 143.5 (C^{9a}), 140.2 (C^{5a}), 134.0 (C^8), 131.0 (C^6), 129.8 (C^{4a}), 129.3 (C^7), 128.0 (C^9), 29.8 (N^1-CH_3), 29.4 (N^3-CH_3); **HR-MS** (ESI^+): *m/z* calculated for $[C_{12}H_{10}N_4O_2+H]^+$: 243.0877 ($[M+H]^+$); found: 243.0874; **IR:** $\tilde{\nu}$ = 2952 (w, sp^2-CH), 1667 (s, C=O amide), 1559 (s, sp^2-CC), 1462 (s, sp^2-CC), 1418 (m, sp^3-CH), 1380 (s, sp^3-CH), 1359 (s), 1299-1068 (s, sp^2-CH), 782 (s), 746 (s, sp^2-CH) cm^{-1}.

2.2.3 5-Ethyl-1,3-dimethyl-5,10-dihydrobenzo[*g*]pteridine-2,4(1*H*,3*H*)-dione (9)

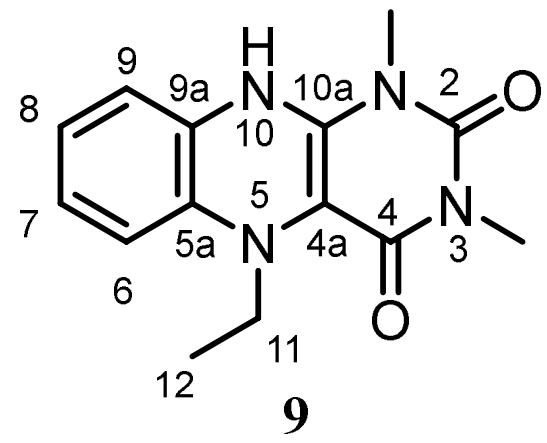

9

Compound **9** was prepared *via* a modified literature procedure.[9]

1,3-Dimethylalloxazine (**SI-25a**, 500 mg, 2.06 mmol, 1.00 equiv.) and Pd/C (10 wt.% Pd, 220 mg, 0.10 equiv.) were evacuated in a 250 mL *Schlenk* flask for 15 minutes. The atmosphere was changed to argon, and degassed EtOH (30 mL) and degassed H_2O (30 mL) were added. Concentrated hydrochloric acid (37%, 3.50 mL) and acetaldehyde (3.48 mL, 61.9 mmol, 30.0 equiv.) were added and the mixture was hydrogenated with a hydrogen gas filled balloon (1 bar) at room temperature overnight. The workup was done under inert atmosphere using conventional *Schlenk* techniques. The mixture was filtrated through a *Whatman* filter into a second 250 mL *Schlenk* flask. Note that the solution must be yellow during this step, attempts where there is a hint of orange or red always failed due to the presence of oxygen. The pH was adjusted to 7-8 by addition of concentrated, degassed (argon bubbling) NH_3 (5 mL, 25% aq.). At this pH the product is extremely air-sensitive. As soon as the color of the mixture started to change from yellow to orange-brown, small amounts of aqueous $Na_2S_2O_4$ were added which regenerated the yellow color back again. Most of the solvents were evaporated. The suspension was cooled in an ice bath, filtered over a *Whatman* filter, and the remaining solids were washed once with degassed H_2O. *Whatman* filtration and drying the solid on the *Schlenk* line gave 545 mg of the N^5-ethylated flavin **9** (2.00 mmol, 97%) as a yellow powder. Since **9** is very sensitive towards molecular oxygen, the *Schlenk* flask was brought into the glovebox for transferring and weighing the product into a small dram vial. An NMR sample was prepared in a *J. Young tube* using degassed $CDCl_3$ with a very thin layer of degassed $D_2O/Na_2S_2O_4$ on top.

Yellow powder, 545 mg (2.00 mmol, 97%); **^{1}H NMR** (400 MHz, $CDCl_3$, 298 K): δ (ppm) = 6.95 – 6.84 (m, 2H, $H^{8,9}$), 6.79 (ddd, $^3J_{H\text{-}H}$ = 7.7, 6.7 Hz, $^4J_{H\text{-}H}$ = 2.1 Hz, 1H, H^7), 6.55 (dd, $^3J_{H\text{-}H}$ = 7.7 Hz, $^4J_{H\text{-}H}$ = 1.3 Hz, 1H, H^6), 3.46 (m, 5H, H^{11}, N^1-CH_3), 3.34 (s, 3H, N^3-CH_3), 1.14 (t, $^3J_{H\text{-}H}$ = 7.0 Hz, 3H, H^{12}); **^{13}C{^{1}H} NMR** (126 MHz, $CDCl_3$, 298 K): δ [ppm] = 158.1 (C^4), 150.5 (C^2), 146.2 (C^{5a}), 136.5 (C^{10a}), 135.0 (C^{9a}), 125.3 (C^9), 123.6 (C^7), 122.7 (C^8), 114.7 (C^6), 99.9 (C^{4a}), 50.7 (C^{11}), 28.7 (N^1-*C*H_3), 28.5 (N^3-*C*H_3), 11.7 (C^{12}); **UV/Vis** (CH_3CN, c = 0.368

mM): λ_{max} (absorption) = 219 (0.3344), 244 (0.3012), 340 nm (0.0496). Absorption values are absolute (UV/Vis spectrum is depicted in section 4).

2.2.4 5-Ethyl-1,3-dimethyl-2,4-dioxo-1,2,3,4-tetrahydrobenzo[*g*]pteridin-5-ium perchlorate (7a)

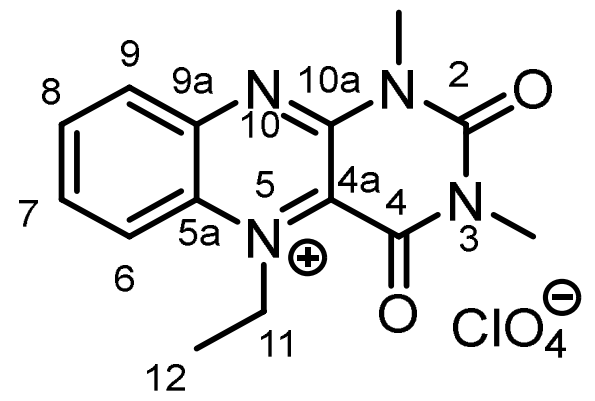

7a

Compound **7a** was prepared according to a modified literature procedure.[11] The compound is known.[12]

In a 250 mL *Schlenk* flask 1,3-dimethylalloxazine **SI-25a** (500 mg, 2.06 mmol, 1.00 equiv.) and Pd/C (10 wt.% Pd, 220 mg, 10 mol%) were evacuated and carefully purged with argon three times. The solids were suspended in degassed EtOH (30 mL) and degassed H_2O (30 mL). Concentrated hydrochloric acid (37 wt.%, 4.17 g, 3.50 mL, 42.3 mmol, 20.5 equiv.) and acetaldehyde (2.73 g, 3.48 mL, 61.9 mmol, 30.0 equiv.) were added to the reaction mixture. An oxygen free hydrogen balloon (1 bar) was attached and the reaction mixture was sparged with hydrogen gas for approximately 30 seconds. Then a freshly filled hydrogen ballon was attached and the reaction mixture was stirred for 16 h under H_2 atmosphere at room temperature. Hereafter, the light-yellow suspension was filtered into a 250 mL *Schlenk* flask using *Whatman* filtration and subsequently the solvents were removed *in vacuo* at 50 °C. Under ice bath cooling and stirring, $HClO_4$ (2 M, 10 mL), $NaNO_2$ (900 mg, 13.0 mmol, 6.33 equiv.) and $NaClO_4$ (2.75 g, 22.5 mmol, 10.9 equiv.) were successively added. After 20 minutes of stirring at 0 °C the suspension was collected by filtration and washed with cooled water (5 x 15 mL). The precipitate was dried *in vacuo*, yielding **7a** (567 mg, 1.53 mmol, 74%) as an orange powder.

Orange powder, 567 mg (74%); **^{1}H NMR** (500 MHz, CD_3CN, 298 K): δ (ppm) = 8.56 (dd, $^3J_{H-H}$ = 8.7 Hz, $^4J_{H-H}$ = 1.2 Hz, 1H, $H^{6/9}$), 8.36 – 8.33 (m, 1H, $H^{6/9}$), 8.32 – 8.28 (m, 1H, $H^{7/8}$), 8.25 (ddd, $^3J_{H-H}$ = 8.6, 6.7 Hz, $^4J_{H-H}$ = 1.7 Hz, 1H, $H^{7/8}$), 3.83 (s, 3H, N^1-CH_3), 3.52 (s, 3H, N^3-CH_3), 1.80 (t, $^3J_{H-H}$ = 7.2 Hz, 3H, H^{12}), the signal of CH_2 group (6.1 and 5.0 ppm) is very broad, often not visible; **^{13}C{^{1}H} NMR** (126 MHz, CD_3CN, 298 K): δ (ppm) = 156.4 (C^4), 149.7 (C^2),

148.7 ($C^{4a/5a/9a/10a}$), 148.0 ($C^{4a/5a/9a/10a}$), 137.8 ($C^{7/8}$), 137.1 ($C^{7/8}$), 131.1 ($C^{6/9}$), 120.7 ($C^{6/9}$), 52.9 (C^{11}), 31.6 (N^1-CH_3), 30.7 (N^3-CH_3), 15.6 (C^{12}); two quaternary carbon signals are not observed, these findings are consistent with literature values;[12] **HR-MS** (ESI^+): *m/z* calculated for [$C_{14}H_{15}N_4O_2$]: 271.1190 ($[M]^+$); found: 271.1186; **IR:** $\tilde{\nu}$ = 3056 (w, sp^2-CH), 2998 (w, sp^3-CH), 2952 (w, sp^3-CH), 1723 (s, C=O), 1676 (s, C=O), 1612 (m, C=C), 1590 (m, C=C), 1554 (m, C=C), 1498 (m, C=C), 1470 (m), 1421 (m, C=C), 1386 (m), 1339 (m), 1299 (m), 1248 (m), 1208 (m), 1169 (m), 1105 (m), 1072 (s), 972 (m), 922 (m), 861 (m, sp^2-CH), 817 (m, sp^2-CH), 802 (m, sp^2-CH), 765 (m, sp^2-CH), 744 (m, sp^2-CH), 694 (m, sp^2-CH). **UV/Vis** (CH_3CN, c = 0.368 mM): λ_{max} (absorption) = 214 (0.3209), 261 (0.5317), 378 (0.1111), 380 (0.1116), 450 nm (0.0750). Absorption values are absolute (UV-Vis spectrum is depicted in section 4).

2.2.5 Semiquinone of 9 (10)

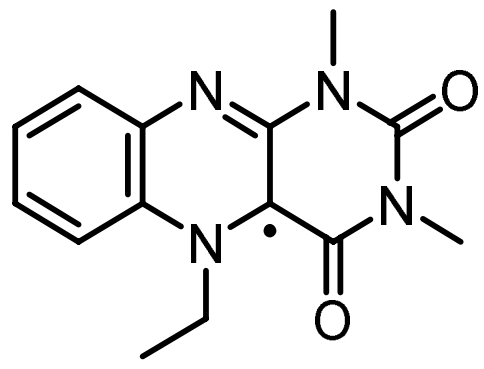

10

Dihydroalloxazine **9** (100 mg, 367 µmol, 1.00 equiv.) was mostly dissolved in 7 mL of dry and degassed MeOH. The clear solution was carefully transfered into a new *Schlenk* flask, ensuring that no solids were transfered with the solution. With a syringe, air (5 mL) was bubbled into the solution, causing the yellow solution to turn slightly red, and the flask was stored in the fridge. After 24 h, dark red, needle shaped crystals formed from the solution. The crystals were collected by filtration and dried to yield **10** (42.7 mg, 154 µmol, 42%). Upon storage under inert conditions no oxidation was observed (visually, EPR).

Dark fine needle shaped crystals, 42.7 mg (42%); **^{1}H NMR:** Due to the open-shell characteristics of the organic radical, no product signals were expected. A sample (1.00 mg, 3.69 µmol, 0.33 equiv.) was dissolved in dry and degassed THF-d_8 under argon with 1,3,5-trimethoxbenzene as internal standard (204.6 µg, 1.22 µmol, 0.33 equiv.) in a *J. Young* NMR tube. The spectrum shows only minor amounts of diamagnetic impurities. The solution was monitored by ^{1}H NMR over the course of two days, while still under argon atmosphere. Nevertheless, slow oxidation occurred and a new signal set was observed (see below); **MS** (EI, 70 eV): *m/z* (%) = 271 (55) $[M]^+$, 256 (2) $[M - CH_3]^+$, 243 (100) $[M - C_2H_5]^+$, 213 (7), 157 (37), 143 (4), 130 (47), 102 (24), 90 (8); **HR-MS** (EI, 70 eV): *m/z* calculated for $[C_{14}H_{15}O_2N_4]^+$: 271.1190 ($[M]^+$); found: 271.1189; **HR-MS** (ESI$^+$): *m/z* calculated for $[C_{14}H_{15}O_2N_4]^+$: 271.1190 ($[M]^+$); found: 271.1183; **HR-MS** (LIFDI): *m/z* calculated for $[C_{14}H_{15}O_2N_4]^+$: 271.1190 ($[M]^+$); found: 271.1189; **IR:** $\tilde{\nu}$ = 2982 (w, sp^3-CH), 2945 (w, sp^3-CH), 1727 (s, C=O), 1674 (m, C=O), 1624 (m, C=C), 1592 (m, C=C), 1574 (m, C=C), 1471 (m), 1455 (m), 1408 (m), 1381 (m), 1361 (m), 1330 (m), 1309 (m), 1296 (m), 1282 (m), 1267 (m), 1234 (m), 1208 (m), 1170 (m), 1156 (m), 1131 (m), 1100 (m), 1084 (m), 1058 (m), 1045 (m), 1031 (m), 976 (m), 942 (m), 920 (m), 862 (m), 814 (m), 777 (m), 757 (m), 744 (m), 736 (m), 694 (m), 670 (m) cm^{-1}. **UV/Vis** (CH_3CN) λ_{max} (ε) = 211 (19145), 258 (38182), 347 (8910), 368 (6210), 394 (8131), 447 nm (3373 cm^{-1}M^{-1}). ε was determined from a linear fit of absorbance vs. concentration ($R^2 \geq 0.999$). UV/Vis spectrum is depicted in section 4 UV/Vis Studies.

EI-MS (sample ionized as solid) and ESI-MS (compound dissolved in MeCN) were not measured under argon. For the LIFDI-MS measurement under argon (substance dissolved in dry and degassed THF) a setup as described by *Fischer et. al.* was used.[13] In all MS measurements the cation $[M]^+$ was detected; no **10** + H^+ adducts were observed in the measurements.

The compound **10** was further characterized by EPR (see section 5 EPR Measurements) and SC-XRD (see section 8 X-Ray Crystallographic Data).

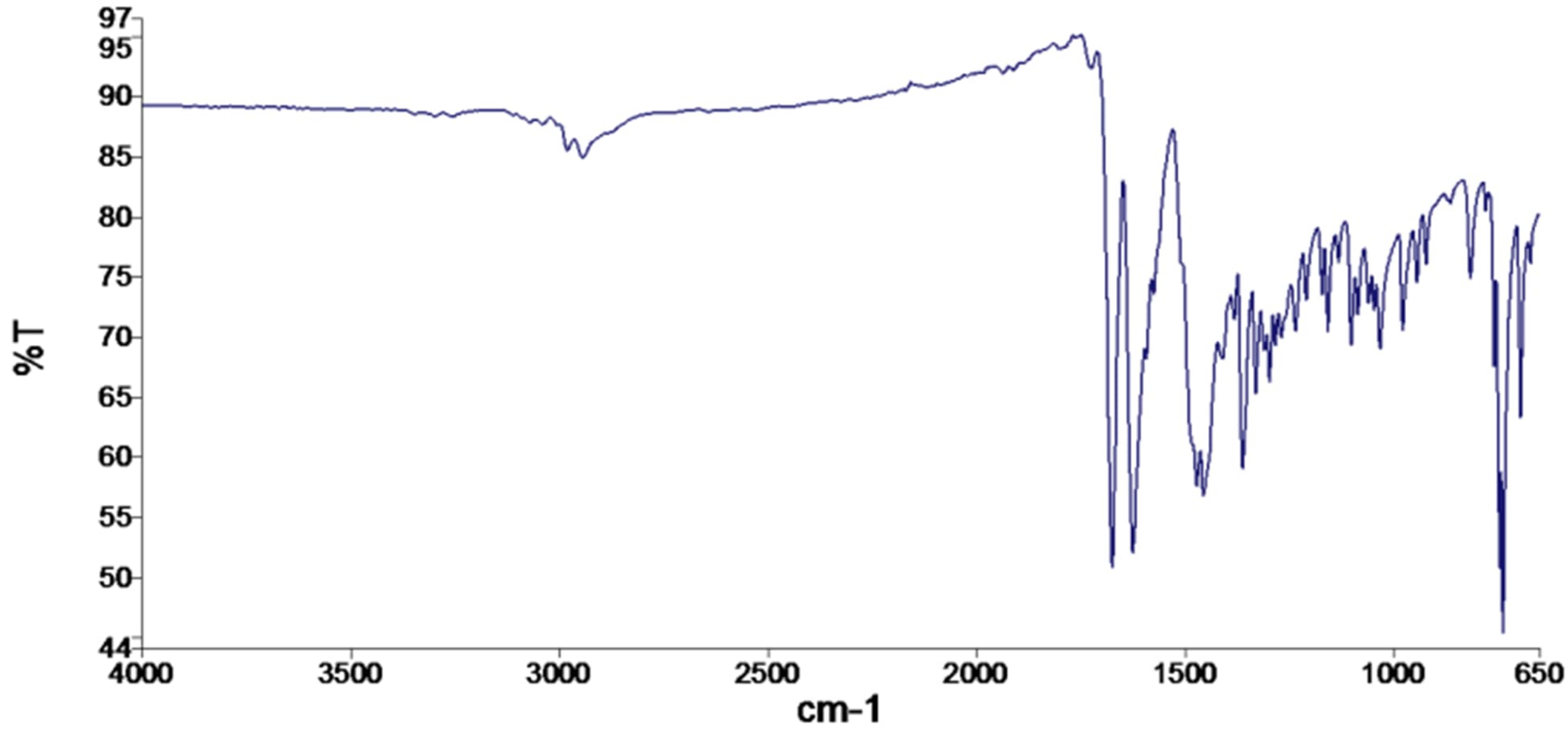

Figure SI-6. IR spectrum of compound **10**.

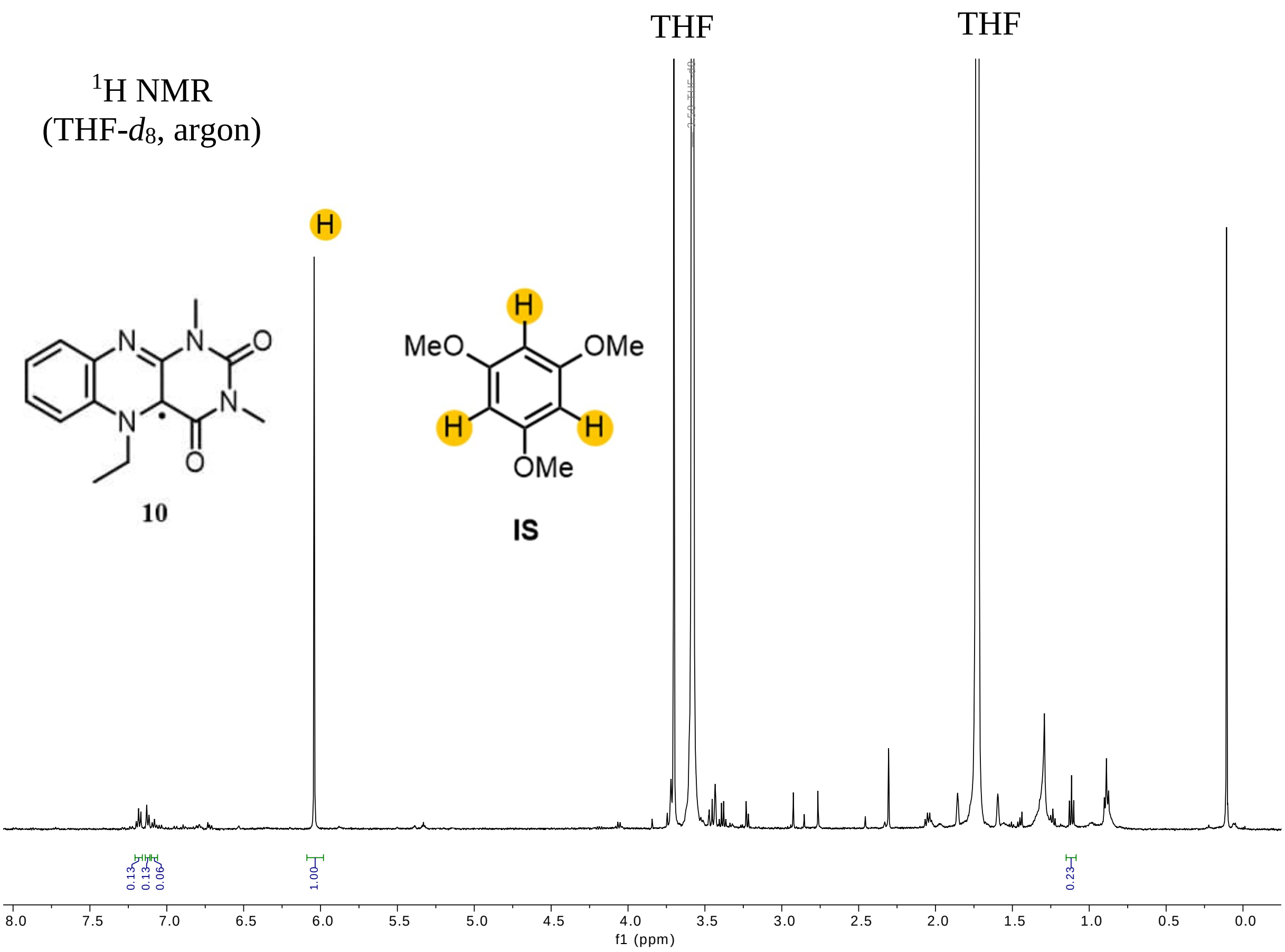

Figure SI-7. ^{1}H NMR spectrum of compound **10** (THF-d$_8$, argon) with 1,3,5-trimethoxbenzene as internal standard (204.6 µg, 1.22 µmol, 0.33 equiv.) in *a J. Young* NMR tube after 5 min.

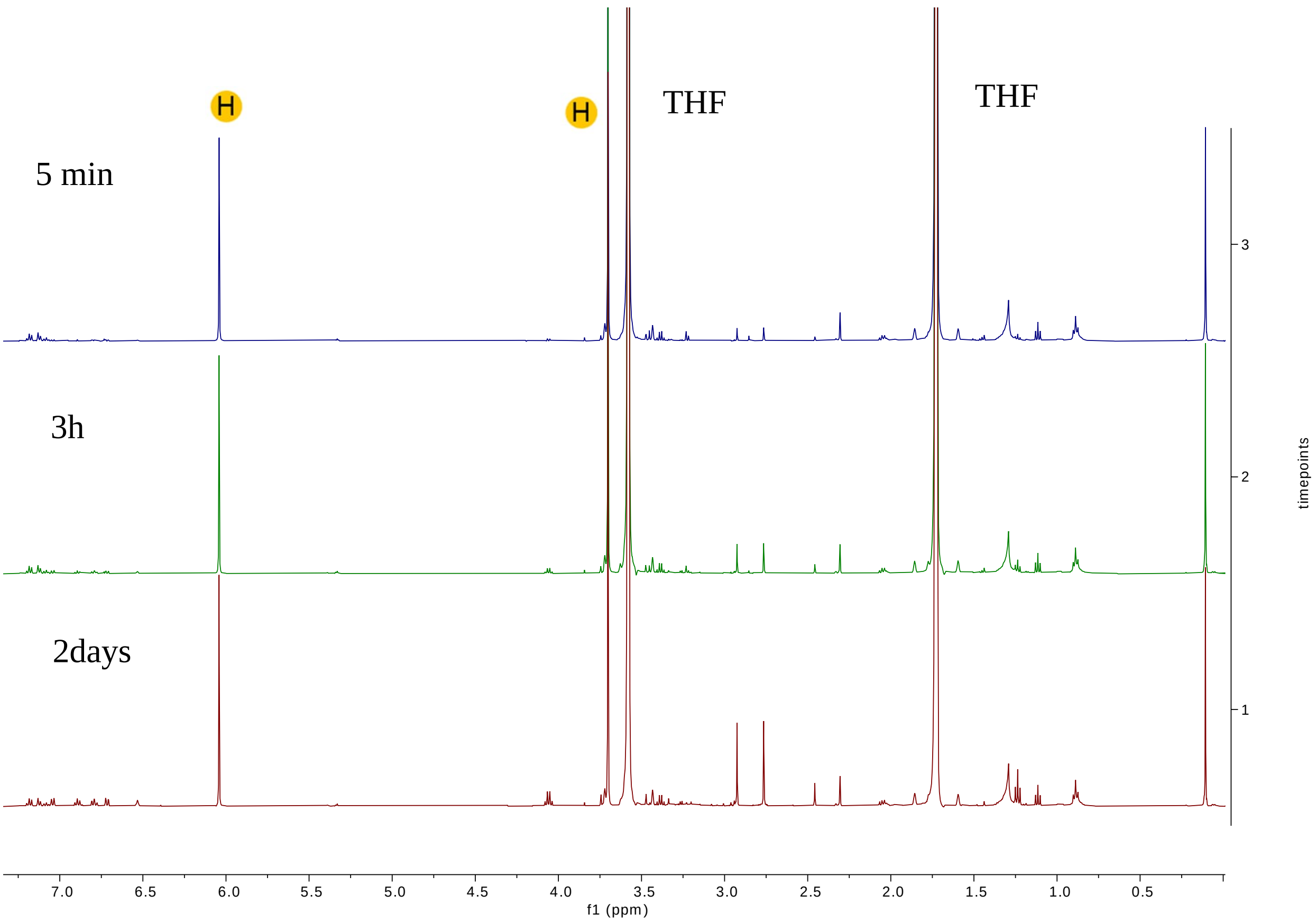

Figure SI-8. ^{1}H NMR stack plot of compound **10** at 5 min, 3 h, and 2 days (THF-d_8, argon) with 1,3,5-trimethoxbenzene as internal standard (204.6 µg, 1.22 µmol, 0.33 equiv.) in a *J. Young* NMR tube.

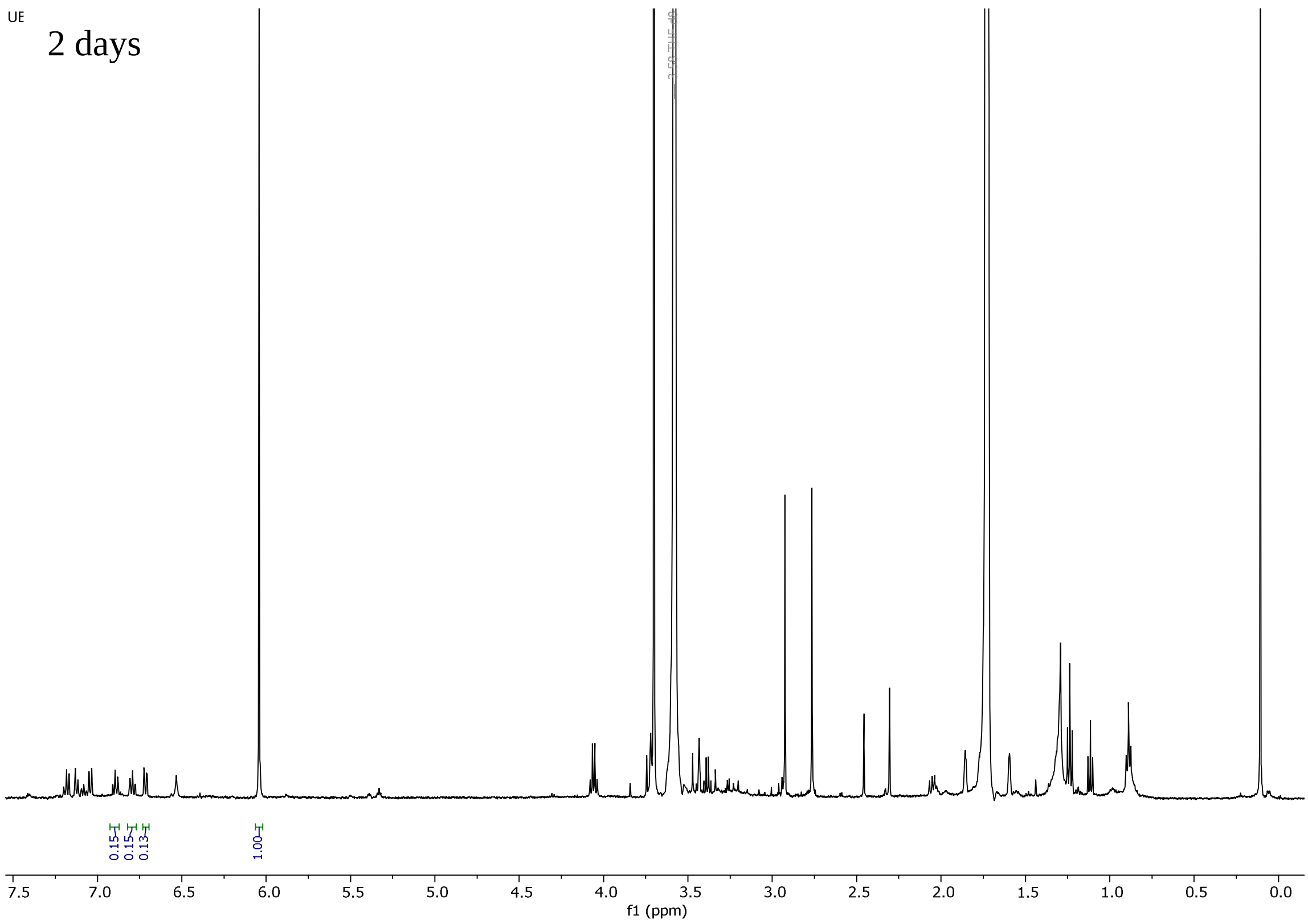

Figure SI-9. ^{1}H NMR stack plot of compound **10** at 5 min, 3 h, and 2 days (THF-d_8, argon) with 1,3,5-trimethoxbenzene as internal standard (204.6 µg, 1.22 µmol, 0.33 equiv.) in a *J. Young* NMR tube after 2 days.

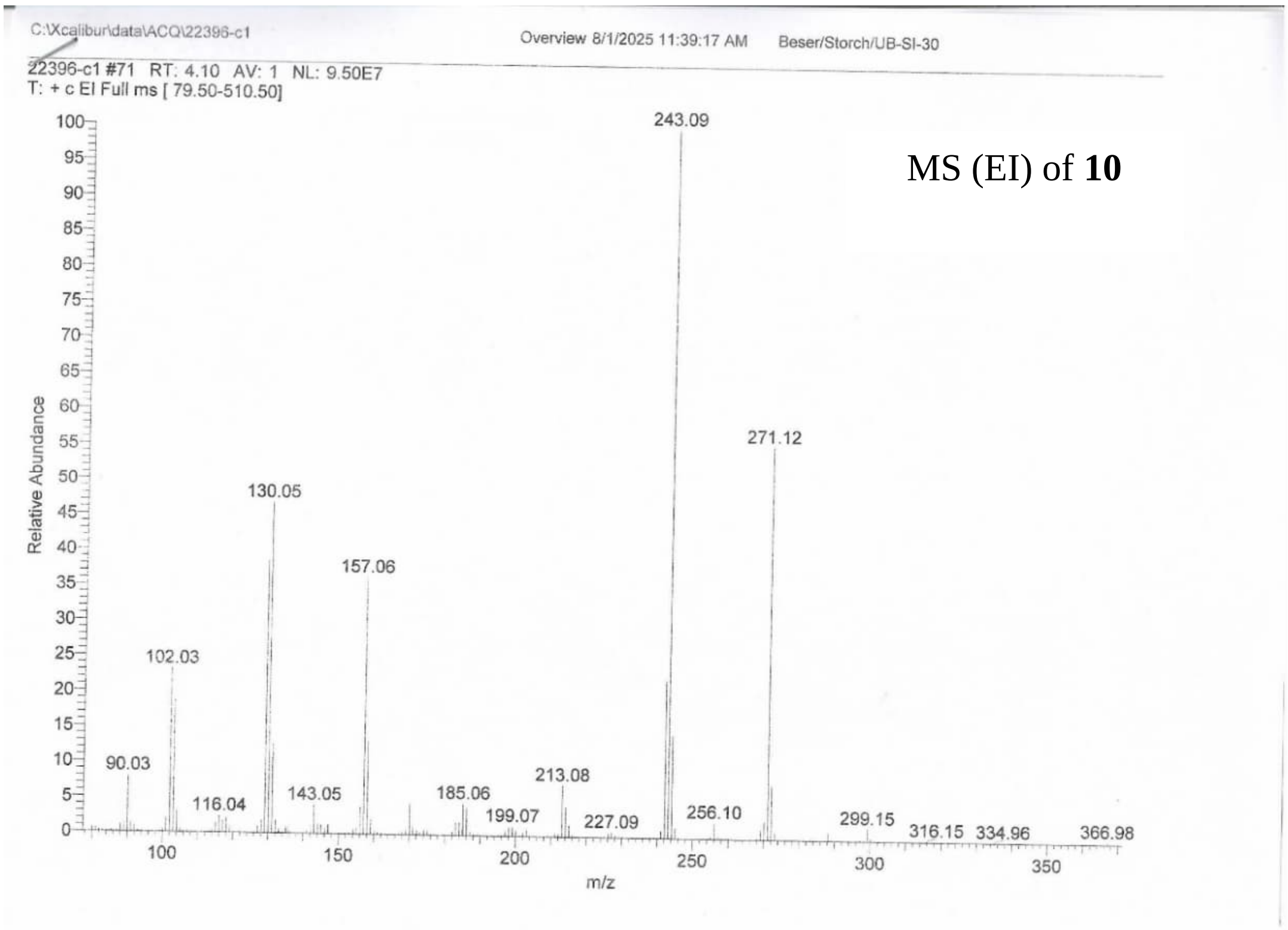

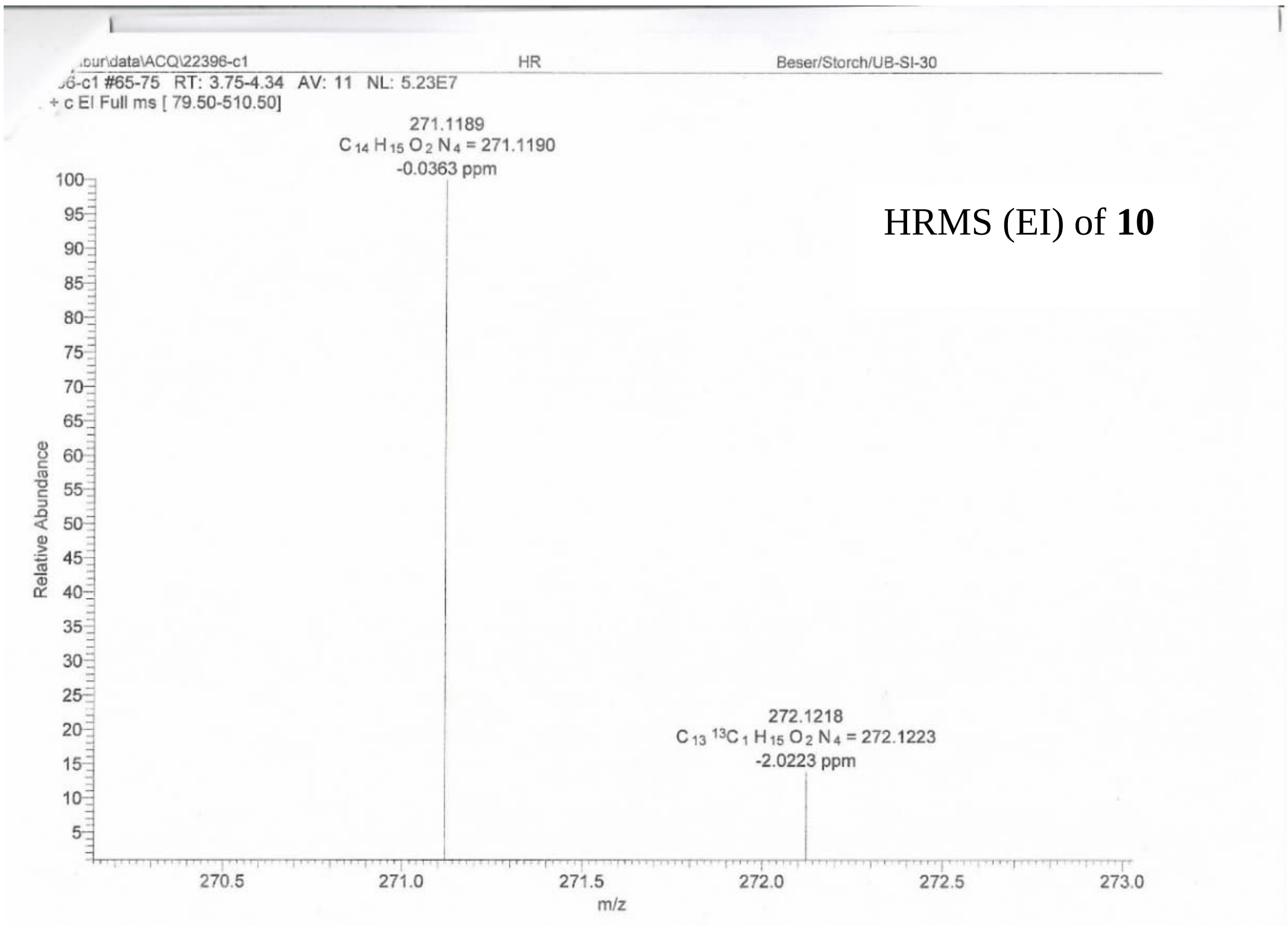

Figure SI-10. HRMS (EI) spectrum of compound **10**.

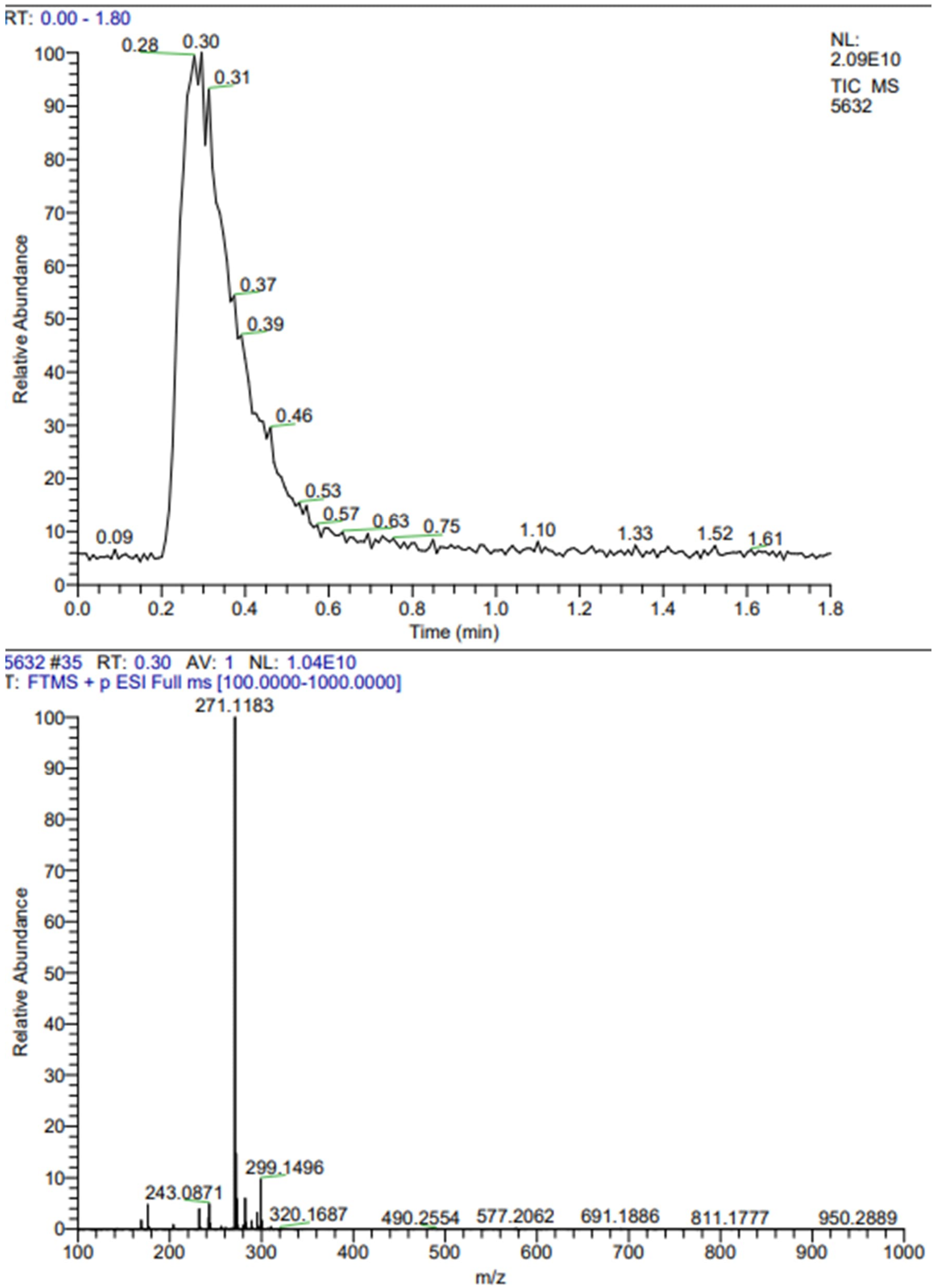

Figure SI-11. HRMS (ESI$^+$) spectrum of compound **10**.

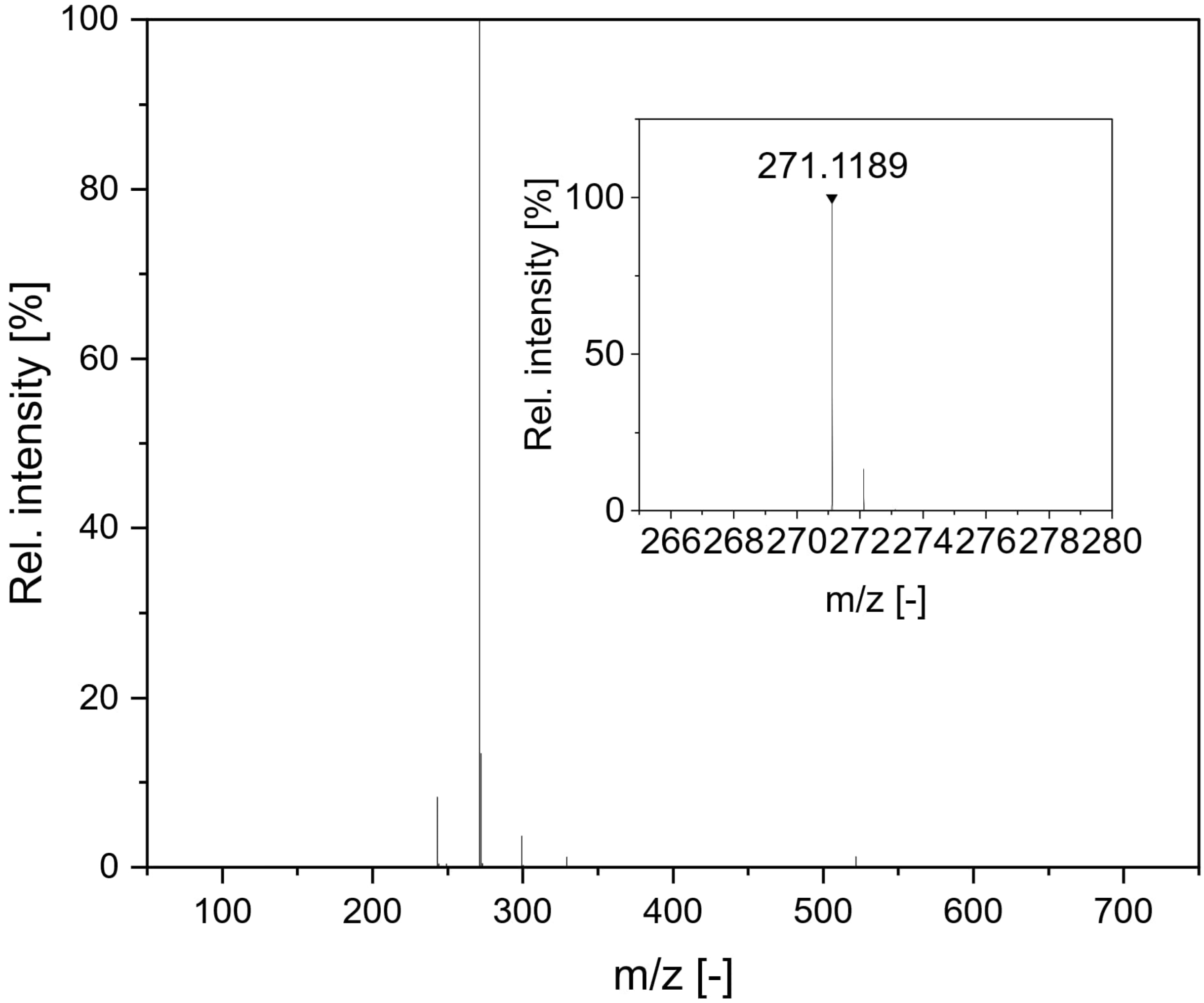

Figure SI-12. HRMS (LIFDI) spectrum of compound **10**.

2.2.6 5-Ethyl-4a-methoxy-1,3-dimethyl-4a,5-dihydrobenzo[*g*]pteridine-2,4(1*H*,3*H*)-dione (11)

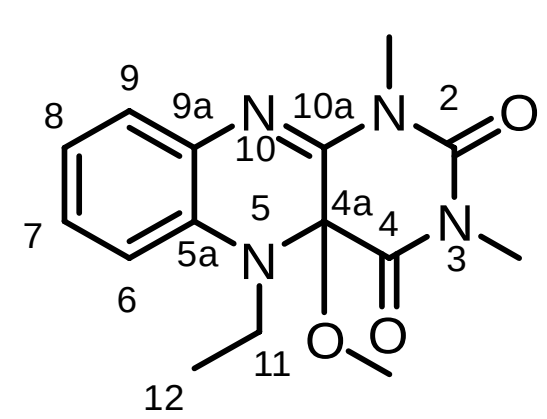

11

The analytical data of compound **11** is consistent with literature.[14]

Dihydroalloxazine **9** (100 mg, 367 µmol, 1.00 equiv.) was partially dissolved in MeOH (dry, 7 mL) under inert atmosphere. The suspension was taken up with a syringe and then filtered

through a 0.45 μm PTFE syringe filter into a new flask (under air). The yellow solution turned brown and was stored in the fridge. After 3 days, the formed yellow/brown crystals were collected by filtration and washed with ice-cold MeOH using centrifugation. The product was dried under reduced pressure to yield crystalline **11** (22.2 mg, 73.4 μmol, 22%).

Colorless solid (22.2 mg, 22%); **^{1}H NMR** (400 MHz, $CDCl_3$, 298 K): δ (ppm) = 7.36 (dd, $^{3}J_{H\text{-}H}$ = 7.8 Hz, $^{4}J_{H\text{-}H}$ = 1.6 Hz, 1H, H^9), 7.23 (ddd, $^{3}J_{H\text{-}H}$ = 8.8, 7.4 Hz, $^{4}J_{H\text{-}H}$ = 1.6 Hz, 1H, H^7), 6.99 – 6.94 (m, 1H, H^6), 6.92 – 6.88 (m, 1H, H^8), 4.12 (dq, $^{2}J_{H\text{-}H}$ = 14.4 Hz, $^{3}J_{H\text{-}H}$ = 7.0 Hz, 1H, H^{11}), 3.64 – 3.46 (m, 1H, H^{11}), 3.55 (s, 3H, N^1CH_3), 3.31 (s, 3H, N^3CH_3), 2.90 (s, 3H, OCH_3), 1.52 (t, $^{3}J_{H\text{-}H}$ = 7.0 Hz, 3H, H^{12}); **^{13}C{^{1}H} NMR** (126 MHz, $CDCl_3$, 298 K): δ (ppm) = 164.3 (C^4), 150.7 (C^2), 142.5 (C^{10a}), 135.1 (C^{5a}), 128.9 (C^{9a}), 128.8 (C^7), 128.3 (C^9), 119.4 (C^8), 111.1 (C^6), 82.5 (C^{4a}), 51.7 (OCH_3), 41.2 (C^{11}), 31.1 (N^1CH_3), 29.3 (N^3CH_3), 13.9 (C^{12}).

2.2.7 5-Butyl-1,3-dimethyl-5,10-dihydrobenzo[*g*]pteridine-2,4(1*H*,3*H*)-dione (9b)

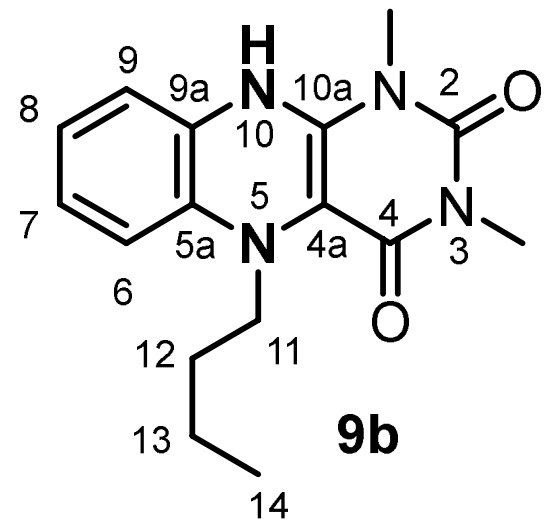

9b

Compound **9b** was prepared *via* a modified literature procedure.[9]

1,3-Dimethylalloxazine (**SI-25a**, 400 mg, 1.65 mmol, 1.00 equiv.) and Pd/C (10 wt.% Pd , 176 mg, 0.10 equiv.) were evacuated in a 250 mL *Schlenk* flask for 15 minutes. The atmosphere was changed to argon, and degassed EtOH (20 mL) and degassed H_2O (20 mL) were added. Concentrated hydrochloric acid (37%, 2.80 mL) and butyraldehyde (3.48 mL, 61.9 mmol, 10.0 equiv.) were added and the mixture was hydrogenated with a hydrogen gas filled balloon (1 bar) at room temperature overnight. The workup was done under inert atmosphere using conventional *Schlenk* techniques. The mixture was filtrated through a *Whatman* filter into a second 250 mL *Schlenk* flask. The pH was adjusted to 7-8 by addition of concentrated, degassed (argon bubbling) NH_3 (5 mL, 25% aq.). At this pH the product is extremely air-sensitive. Most of the solvents were evaporated. The suspension was cooled in an ice bath, filtered over a *Whatman* filter, and the remaining solids were washed once with degassed H_2O. *Whatman* filtration and drying the solid on the *Schlenk* line gave 340 mg of the N^5-ethylated flavin **9b** (1.13 mmol, 69%) as a beige powder. Since **9b** is very sensitive towards molecular oxygen, the *Schlenk* flask was brought into the glovebox for transferring and weighing the product into a small dram vial. An NMR sample was prepared in a *J. Young tube* using degassed $CDCl_3$. Besides the product signals, the contains non-aromatic impurities with a butyl chain, maybe aldol condensation products of butyraldehyde, that could not be removed *in vacuo*. For a clean isolation of **9b** a lower excess of butyraldehyde and/or a washing of the product with degassed unpolar solvents (*e.g. n*-pentane/ diethylether) may be tried. Compound **9b** was used as is without further purification for the crystallization of **10b**.

Beige powder, 340 mg (1.13 mmol, 69%); **^{1}H NMR** (400 MHz, $CDCl_3$, 298 K): δ (ppm) = 6.93 – 6.80 (m, 2H, $H^{8,9}$), 6.76 (*virt.* td, $^3J_{H\text{-}H} \approx {}^3J_{H\text{-}H}$ = 7.4 Hz, $^4J_{H\text{-}H}$ = 1.7 Hz, 1H, H^7), 6.56 (dd, $^3J_{H\text{-}H}$ = 7.7 Hz, $^3J_{H\text{-}H}$ = 1.4 Hz, 1H, H^6), 5.94 (s, 1H, NH), 3.47 (s, 3H, N^1-CH_3), 3.43 – 3.38 (m, 5H, N^3-CH_3, H^{11}), 1.66 – 1.54 (m, 2H, H^{12}), 1.30 – 1.21 (m, 2H, H^{13}), 0.85 (t, $^3J_{H\text{-}H}$ =

7.4 Hz, 1H, H^{14}); **^{13}C{^{1}H} NMR** (126 MHz, $CDCl_3$, 298 K): δ [ppm] = 157.9 (C^{4}), 150.5 (C^{2}), 146.1 (C^{5a}), 137.4 (C^{10a}), 134.9 (C^{9a}), 125.2 (C^{9}), 123.2 (C^{7}), 122.1 (C^{8}), 114.9 (C^{6}), 100.2 (C^{4a}), 55.7 (C^{11}), 29.2 (C^{12}), 28.8 (N^{1}-*C*H_3), 28.4 (N^{3}-*C*H_3), 20.3 (C^{13}), 14.2 (C^{14}).

2.2.8 Semiquinone of 9b (10b)

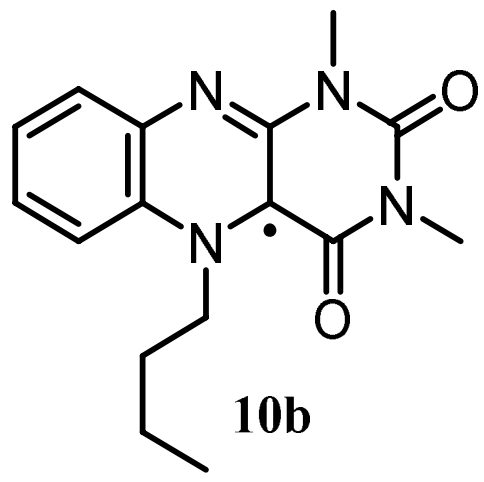

10b

Dihydroalloxazine **9b** (100 mg, 367 µmol, 1.00 equiv.) was mostly dissolved in 7 mL of dry and degassed MeOH. The clear solution was carefully transfered into a new *Schlenk* flask, ensuring that no solids were transfered with the solution. With a syringe, air (5 mL) was bubbled into the solution and the flask was stored in a dark place. After 2 weeks, crystals had formed from the solution which were used for measuring a SC-XRD. No yield was determined and no further analysis was performed.

2.2.9 2,4-Dioxo-1,2,3,4-tetrahydrobenzo[*g*]pteridine-7&8-carbonitrile (SI-24b)

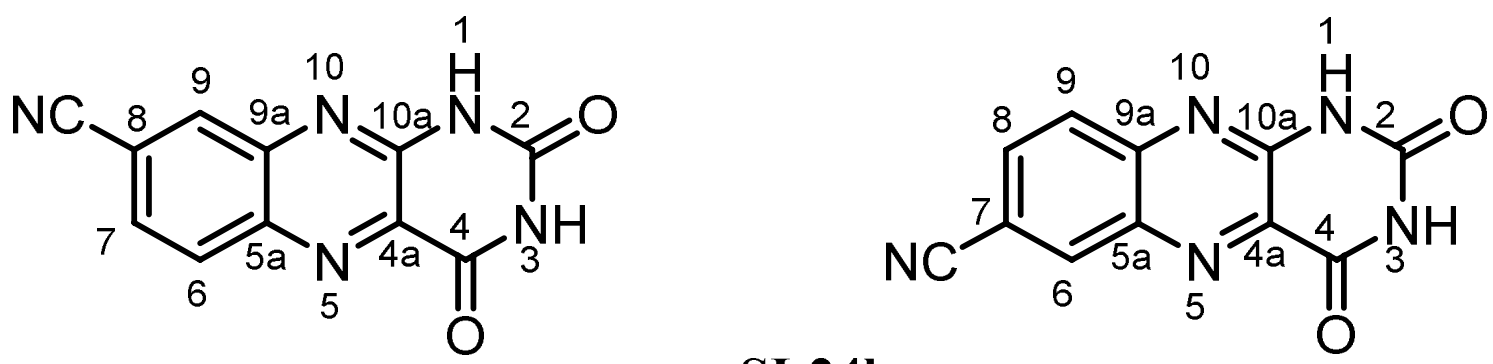

SI-24b

Compound **SI-24b** was prepared *via* a modified literature procedure. [11]

In a 100 mL round bottom flask, alloxan monohydrate (804 mg, 5.02 mmol, 1.00 equiv.) and boric acid (342 mg, 5.53 mmol, 1.10 equiv.) were dissolved in glacial acetic acid (20 mL) and heated to 116 °C using a findenser. Once the solids were dissolved, a solution of 3,4-diaminobenzonitrile (669 mg, 5.02 mmol, 1.00 equiv.) in glacial acetic acid (10 mL) was added to the reaction mixture, after which the formation of a yellow precipitate was observed. After stirring at room temperature for 18 h, the solids of the suspension were collected by filtration and successively washed with diethyl ether and water (3 x 20 mL). The precipitate was dried

in vacuo to afford a mixture of 7/8-regioisomers of **SI-24b** in a 1:1.5 ratio (1.12 g, 4.67 mmol, 93%) as a yellow solid. The compound was used without further separation of the regioisomers.

Yellow solid, 1.12 g (93%, 7/8 = 40:60); **TLC** (CH_2Cl_2/MeOH, 9/1): R_f = 0.48 [UV, both regioisomers]; **^{1}H NMR** (7-regioisomer): (500 MHz, DMSO-d_6 , 298 K): δ (ppm) = 12.20 (br s, 1H, N^3*H*), 11.88 (br s, 1H, N^1*H*), 8.80 (d, $^4J_{H\text{-}H}$ = 1.8 Hz, 1H, H^6), 8.18 (dd, $^3J_{H\text{-}H}$ = 8.8 Hz, $^4J_{H\text{-}H}$ = 1.9 Hz, 1H, H^8), 8.02 (d, $^3J_{H\text{-}H}$ = 8.7 Hz, 1H, H^9); **^{1}H NMR** (8-regioisomer): (500 MHz, DMSO-d_6, 298 K): δ (ppm) = 12.15 (s, 1H, N^3*H*), 11.87 (s, 1H, N^1*H*), 8.51 (d, $^4J_{H\text{-}H}$ = 1.8 Hz, 1H, H^9), 8.32 (d, $^3J_{H\text{-}H}$ = 8.6 Hz, 1H, H^6), 8.05 (dd, $^3J_{H\text{-}H}$ = 8.6 Hz, $^4J_{H\text{-}H}$ = 1.8 Hz, 1H, H^7); **^{13}C{^{1}H} NMR** (signals from either 7- or 8-regioisomer,*,** 7-regioisomer*, 8-regioisomer**): (126 MHz, DMSO-d_6, 298 K): δ (ppm) = 160.2 (C$^{2/4/4a/10a}$)*,**, 160.1 (C$^{2/4/4a/10a}$)*,**, 150.2 (2C, C$^{2/4/4a/10a}$)*,**, 148.6 (C$^{2/4/4a/10a}$)*,**, 148.0 (C$^{2/4/4a/10a}$)*,**, 144.6 (C^{9a})*, 141.8 (C^{9a})**, 140.4 (C^{5a})**, 138.0 (C^{5a})*, 136.4 (C^6)*, 134.6 (C$^{2/4/4a/10a}$)*,**, 134.2 (C$^{2/4/4a/10a}$)*,**, 133.7 (C^8)*, 133.0 (C^9)**, 131.7 (C^6)**, 129.0 (C^7)**, 128.6 (C^9)*, 118.3 (C≡N)*, 118.2 (C≡N)**, 115.0 (C^8)**, 110.4 (C^8)*; **IR:** $\tilde{\nu}$ = 3578 (w, NH), 3447 (w, NH), 3182 (m), 3095 (m, sp^2-CH), 3046 (m), 2827 (m), 2233 (w, C≡N), 1702 (vs, C=O), 1625 (m, C=O), 1585 (s), 1499, 1453, 1414, 1391, 1370, 1351, 1341, 1302, 1282, 1203, 1162, 1143, 1118, 1039, 961, 918, 896, 860, 834, 815, 789, 757, 700, 665, 656 cm^{-1}.

2.2.10 8-Cyano-1,3-dimethylalloxazine (SI-25b)

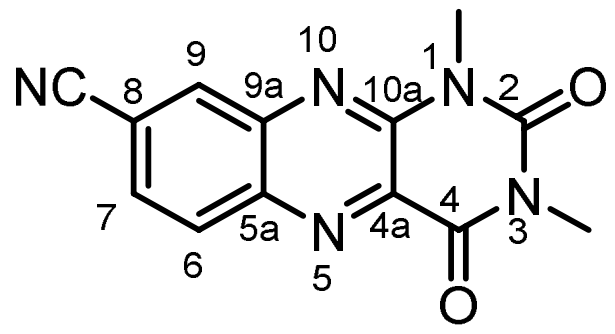

SI-25b

Compound **SI-25b** was prepared *via* a modified literature procedure. [11]

In a 100 mL round bottom flask a regioisomeric mixture of 7/8-cyanoalloxazine **SI-24b** (1.00 g, 4.18 mmol, 1.00 equiv., 7/8 = 40:60) and K_2CO_3 (3.00 g, 21.7 mmol, 5.20 equiv.) were suspended in dry DMF (30 mL). Methyl iodide (1.39 g, 609 µL, 9.78 mmol, 2.34 equiv.) was added to the reaction mixture. After stirring at room temperature for 22 h, the suspension was quenched with an aqueous ammonia solution (25%, 20 mL), the solid collected by filtration and washed with water (3 x 20 mL). The precipitate was dried *in vacuo* to afford a mixture of 7/8-regioisomers in a 33:67 ratio (990 mg, 3.71 mmol, 89%). Recrystallization from ethanol

(300 mL) afforded a crystalline mixture of the 7/8-isomers in a 1:3 ratio and a mother liquor containing the 7/8-isomers in a 3:1 ratio. A series of successive crystallizations (10 times) from ethanol yielded the nearly pure 8-cyano regioisomer **SI-25b** (367 mg, 1.38 mmol, 37%, 99% regioisomer purity) as a yellow solid.

Yellow solid, 3.67 mg (37%); **TLC** (CH_2Cl_2/MeOH, 9/1): R_f = 0.66 [UV]; **^{1}H NMR** (500 MHz, $CDCl_3$, 298 K): δ (ppm) = 8.45 (d, $^3J_{H\text{-}H}$ = 8.7 Hz, 1H, H^6), 8.42 (d, $^4J_{H\text{-}H}$ = 1.8 Hz, 1H, H^9), 7.89 (dd, $^3J_{H\text{-}H}$ = 8.7 Hz, $^4J_{H\text{-}H}$ = 1.8 Hz, 1H, H^7), 3.83 (s, 3H, N^1-CH_3), 3.62 (s, 3H, N^3-CH_3); **^{13}C{^{1}H} NMR** (126 MHz, $CDCl_3$, 298 K): δ (ppm) = 159.1 (C^4), 150.4 (C^2), 146.5 (C^{10a}), 142.5 (C^{4a}), 141.0 (C^{5a}), 133.8 (C^9), 132.4 (C^6), 132.0 (C^{9a}), 129.7 (C^7), 117.6 (C^{11}), 117.1 (C^8), 30.1 (N^1-CH$_3$), 29.6 (N^3-CH$_3$); **HR-MS** (ESI$^+$): *m/z* calculated for $[C_{13}H_9N_5O_2+H]^+$: 268.0829 ($[M+H]^+$); found: 268.0830; **IR:** $\tilde{\nu}$ = 3378 (w), 3092 (w, sp^2-CH), 3078 (w, sp^2-CH), 3060 (w, sp^2-CH), 2965 (w), 2285 (w), 2233 (m, C≡N), 1976 (w), 1839 (w), 1791 (w), 1716 (s), 1673 (vs, C=O), 1618 (m, C=O), 1555 (vs), 1499, 1465, 1413, 1364, 1321, 1292, 1234, 1218, 1207, 1148, 1100, 1060, 982, 957, 898, 850, 821, 810, 783, 770, 745, 710, 686, 652 cm^{-1}.

2.2.11 5-Ethyl-1,3-dimethyl-2,4-dioxo-8-(cyano)-1,2,3,4-tetrahydrobenzo[*g*]pteridin-5-ium perchlorate (7b)

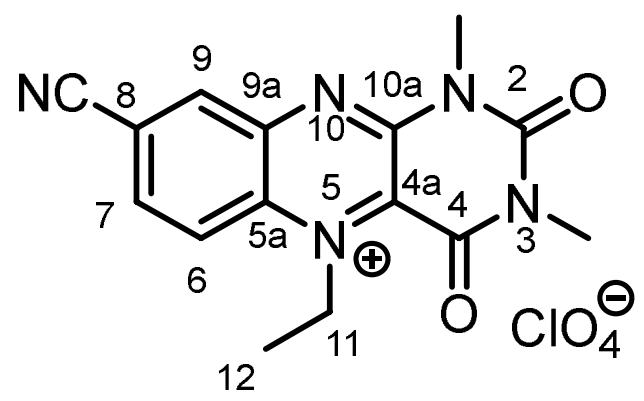

7b

Compound **7b** was prepared *via* a modified literature procedure.[11]

A 250 mL *Schlenk* flask filled with 8-cyano-1,3-dimethyl-alloxazine (**SI-25b**, 150 mg, 550 µmol, 1.00 equiv.), sodium cyanoborohydride (354 mg, 5.63 mmol, 10.2 equiv.) and sodium dithionite (392 mg, 2.25 mmol, 4.10 equiv.) was evacuated and carefully purged with argon three times. The reaction mixture was suspended in degassed DMF (15 mL). Acetic acid (178 mg, 170 µL, 2.95 mmol, 5.40 equiv.) and acetaldehyde (2.35 g, 3.00 mL, 53.8 mmol, 97.2 equiv.) were added to the reaction mixture. After vigorous stirring at 60 °C for 24 h, more acetaldehyde (1 mL, 17.8 mmol, 32.4 equiv.) was added to the dark brown suspension. After stirring at 60 °C for another 16 h, water (20 mL) was added to the suspension and the reaction mixture was extracted with dichloromethane (10 x 10 mL). The combined organic phases were washed with brine (3 x 15 mL) and dried in vacuo. Under ice bath cooling (0 °C) and stirring, $HClO_4$ (aq. 2 M, 6 mL), $NaNO_2$ (300 mg, 4.35 mmol, 7.91 equiv.) and $NaClO_4$ (780 mg, 6.37 mmol, 11.6 equiv.) were successively added to the remaining brown solid. After 90 minutes of stirring at room temperature, the solid of the suspension was collected by filtration and successively washed with $HClO_4$ (aq. 2 M, 3 x 5 mL) and cooled water (3 x 10 mL). The precipitate was dried *in vacuo*, transferred to a 25 mL round flask and dissolved in a minimal amount of dry acetonitrile. After crashing it out with diethyl ether (10 mL), the precipitate was filtered off, washed with diethyl ether (3 x 10mL) and dried *in vacuo*, yielding **7b** (33.4 mg, 113 µmol, 20%) as an ocher yellow solid.

Ocher yellow powder, 33.4 mg (20%); **^{1}H NMR** (400 MHz, CD_3CN, 298 K): δ (ppm) = 8.78 (d, $^4J_{H\text{-}H}$ = 1.8 Hz, 1H, H^9), 8.68 (d, $^3J_{H\text{-}H}$ = 9.5 Hz, 1H, H^6), 8.37 (dd, $^3J_{H\text{-}H}$ = 9.5 Hz, $^4J_{H\text{-}H}$ = 1.8 Hz, 1H, H^7), 3.83 (s, 3H, N^1-CH_3), 3.53 (s, 3H, N^3-CH_3), 1.80 (t, $^3J_{H\text{-}H}$ = 7.2 Hz, 3H, H^{12}); the signal of CH_2 group (5.76 ppm) is very diffuse, often not visible; **^{13}C{^{1}H} NMR** (101 MHz, CD_3CN, 298 K): δ (ppm) = 155.9 (C^4)*, 149.5 (C^2)*, 147.0 (C^{9a}), 136.8 (C^9), 136.4 (C^7), 122.8 (C^6), 120.7 (C^8), 117.4 (C≡N), 53.8 (C^{11}), 31.9 (N^1-CH_3), 31.0 (N^3-CH_3), 15.6 (C^{12}); ($C^{4a,\ 5a,}$

^{10a}) are missing due to low intensity; **IR:** $\tilde{\nu}$ = 3087 (w, sp^2-CH), 2240 (w, C≡N), 1730 (w), 1683 (m, C=O), 1619 (w, C=O), 1589 (w), 1558 (m), 1508 (w), 1473, 1423, 1375, 1343, 1298, 1248, 1209, 1168, 1081, 971, 892, 825, 797, 744, 694 cm^{-1}.

* low intensity in ^{13}C

2.2.12 7- & 8-(Trifluoromethyl)benzo[*g*]pteridine-2,4(1*H*,3*H*)-dione (SI-24c)

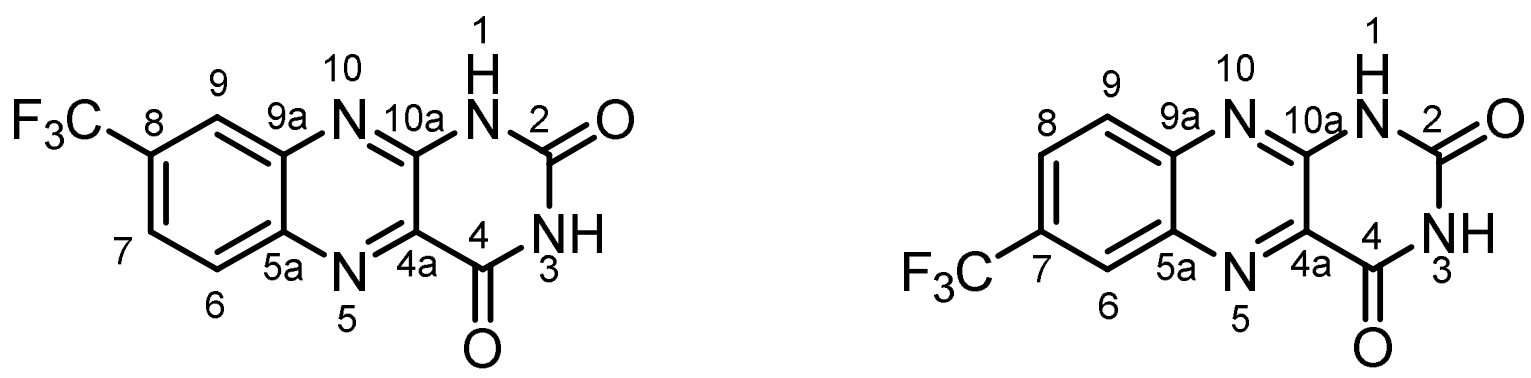

SI-24c

Compound **SI-24c** was prepared *via* a modified literature procedure.[11]

In a 100 mL round bottom flask alloxan monohydrate (844 mg, 5.27 mmol, 1.05 equiv.) and boric acid (341 mg, 5.52 mmol, 1.10 equiv.) were dissolved in glacial acetic acid (20 mL) and heated to 110 °C using a findenser. Once the solids were dissolved, a solution of 4-trifluoromethylbenzene-1,2-diamine (895 mg, 5.02 mmol, 1.00 equiv.) in glacial acetic acid (10 mL) was added to the reaction mixture, after which a dark brown coloration of the suspension was observed. After vigorous stirring at room temperature for 22 h, the solid of the suspension was collected by filtration and successively washed with diethyl ether and water (3 x 20 mL). The precipitate was dried *in vacuo*, to afford a mixture of the 7/8-regioisomers **SI-24c** in a 23:77 ratio (1.29 g, 4.59 mmol, 91%) as a pale yellow solid. The compound was used without further separation of the regioisomers.

Pale yellow solid, (1.29 g, 4.59 mmol, 91%, 7/8 = 23:77); **TLC** (CH_2Cl_2/MeOH, 9/1): R_f = 0.47 [UV] (both regioisomers); **^{1}H NMR** (7-regioisomer): (500 MHz, DMSO-d_6 , 298 K): δ (ppm) = 12.14 (br s, 1H, N*H*), 11.88 (br s, 1H, N*H*), 8.56 (d, $^4J_{H-H}$ = 2.1 Hz, 1H, H^6), 8.15 (dd, $^3J_{H-H}$ = 8.9 Hz, $^4J_{H-H}$ = 2.1 Hz, 1H, H^8), 8.09 (d, $^3J_{H-H}$ = 8.9 Hz, 1H, H^9); **^{1}H NMR** (8-regioisomer): (500 MHz, DMSO-d_6, 298 K): δ (ppm) = 12.14 (br s, 1H, N*H*), 11.88 (br s, 1H, N*H*), 8.38 (d, $^3J_{H-H}$ = 8.8 Hz, 1H, H^6), 8.24 (d, $^4J_{H-H}$ = 2.0 Hz, 1H, H^9), 8.01 (dd, $^3J_{H-H}$ = 8.8 Hz, $^4J_{H-H}$ = 2.0 Hz, 1H, H^7); **$^{13}C\{^1H\}$ NMR** (7-regioisomer): (126 MHz, DMSO-d_6, 298 K): δ (ppm) = 160.19 ($C^{2/4/4a/10a}$), 150.22 ($C^{2/4/4a/10a}$), 148.5 ($C^{2/4/4a/10a}$), 144.2 (C^{9a}), 137.9 (C^{5a}), 133.9 ($C^{2/4/4a/10a}$), 128.9 (C^9), 128.3 (q, $^3J_{C-F}$ = 3.3 Hz, C^8), 128.09 (q, $^3J_{C-F}$ = 4.4 Hz, C^6), 128.06 (q, $^2J_{C-F}$ = 32.4 Hz, C^7), 123.9 (q, $^1J_{C-F}$ = 273.2 Hz, *C*F_3); **$^{13}C\{^1H\}$ NMR** (8-regioisomer):

(126 MHz, DMSO-d_6, 298 K): δ (ppm) = 160.24 ($C^{2/4/4a/10a}$), 150.20 ($C^{2/4/4a/10a}$), 148.0 ($C^{2/4/4a/10a}$), 141.8 (C^{9a}), 140.2 (C^{5a}), 134.4 ($C^{2/4/4a/10a}$), 132.3 (q, $^2J_{C\text{-}F}$ = 32.3 Hz, C^8), 132.1 (C^6), 124.8 (q, $^3J_{C\text{-}F}$ = 5.0 Hz, C^9), 123.7 (q, $^1J_{C\text{-}F}$ = 273.2 Hz, CF_3), 123.4 (q, $^3J_{C\text{-}F}$ = 3.1 Hz, C^7); **^{19}F NMR**: (376 MHz, $CDCl_3$, 298 K): δ (ppm) = − 60.9 (7-regioisomer), − 61.4 (8-regioisomer); **HR-MS** (ESI$^+$): *m/z* calculated for $[C_{11}H_5F_3N_4O_2+H]^+$: 283.0437 ($[M+H]^+$); found: 283.0439; **IR:** $\tilde{\nu}$ = 3566 (w, NH), 3448 (w, NH), 3187 (w), 3099 (m, sp^2-CH), 2829 (w), 1741 (s), 1702 (s, C=O), 1646 (m, C=O), 1589 (s), 1512 (w), 1455, 1401, 1372, 1362, 1350, 1322, 1299, 1266, 1210, 1160, 1128, 1058, 1039, 981, 945, 911, 896, 872, 844, 826, 812, 793, 765, 711, 681 cm^{-1}.

2.2.13 1,3-Dimethyl-8-(trifluoromethyl)benzo[*g*]pteridine-2,4(1*H*,3*H*)-dione (SI-25c)

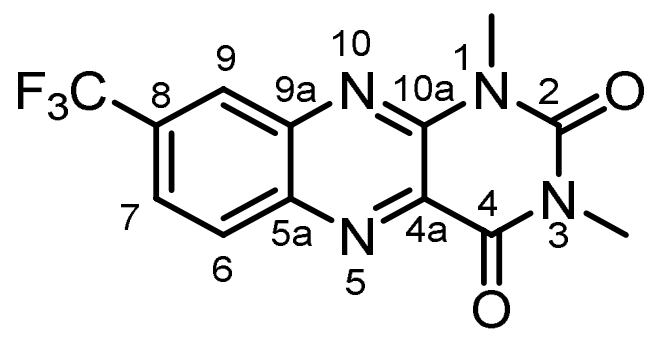

SI-25c

Compound **SI-25c** was prepared *via* a modified literature procedure.[11]

In a 100 mL round bottom flask a regioisomeric mixture of 7/8-(trifluoromethyl)alloxazine **SI-24c** (1.00 g, 3.54 mmol, 1.00 equiv., rr = 23:77) and K_2CO_3 (2.55 g, 18.4 mmol, 5.20 equiv.) were dissolved in dry DMF (25 mL). Methyl iodide (1.18 g, 516 µL, 8.29 mmol, 2.34 equiv.) was added to the reaction mixture. After vigorous stirring at room temperature for 68 h, the suspension was quenched with an aqueous ammonia solution (25%, 15 mL), the solid collected by filtration and washed with water (3 x 20 mL). The precipitate was dried *in vacuo*, yielding mainly the 8-trifluoro-regioisomer (**SI-25c**) (3.17 g, 12.3 mmol, 61%, 98% regioisomer purity) as a light yellow solid.

Pale yellow solid, 3.17 g (61%); **TLC** (CH_2Cl_2/MeOH = 9/1): R_f = 0.72 [UV]; **^{1}H NMR** (400 MHz, $CDCl_3$, 298 K): δ (ppm) = 8.48 (d, $^3J_{H\text{-}H}$ = 8.8 Hz, 1H, H^6), 8.36 (s, 1H, H^9), 7.93 (dd, $^3J_{H\text{-}H}$ = 8.8 Hz, $^4J_{H\text{-}H}$ = 2.1 Hz, 1H, H^7), 3.85 (s, 3H, N^1-CH_3), 3.63 (s, 3H, N^3-CH_3); **^{13}C{^{1}H} NMR** (126 MHz, $CDCl_3$, 298 K): δ (ppm) = 159.4 (C^4), 150.6 (C^2), 146.3 (C^{10a}), 142.6 (C^{9a}), 140.8 (C^{5a}), 135.1 (q, $^2J_{C\text{-}F}$ = 33.1 Hz, C^8), 132.2 (C^6), 131.6 (C^{4a}), 126.0 (q, $^3J_{C\text{-}F}$ = 4.5 Hz, C^9), 124.9 (q, $^3J_{C\text{-}F}$ = 3.0 Hz, C^7), 123.4 (q, $^1J_{C\text{-}F}$ = 273.3 Hz, CF_3), 30.0 (N^1-CH_3),

29.5 (N^3-CH_3); **^{19}F NMR**: (376 MHz, $CDCl_3$, 298 K): δ (ppm) = − 63.22; **HR-MS** (ESI^+): *m/z* calculated for $[C_{13}H_9F_3N_4O_2+H]^+$: 311.0750 ($[M+H]^+$); found: 311.0750; **IR:** $\tilde{\nu}$ = 3388 (w), 3050 (w, sp^2-CH), 2960 (w, sp^2-CH), 2003 (w), 1861 (w), 1728 (m), 1672 (vs, C=O), 1628 (m, C=O), 1569 (s), 1508 (m), 1472, 1449, 1419, 1370, 1347, 1323, 1301, 1286, 1230, 1196, 1160, 1125, 1071, 1055, 1005, 988, 969, 942, 893, 863, 818, 812, 788, 775, 748, 694, 685, 655 cm^{-1}.

2.2.14 5-Ethyl-1,3-dimethyl-2,4-dioxo-8-(trifluoromethyl)-1,2,3,4-tetrahydrobenzo[*g*]pteridin-5-ium perchlorate (7c)

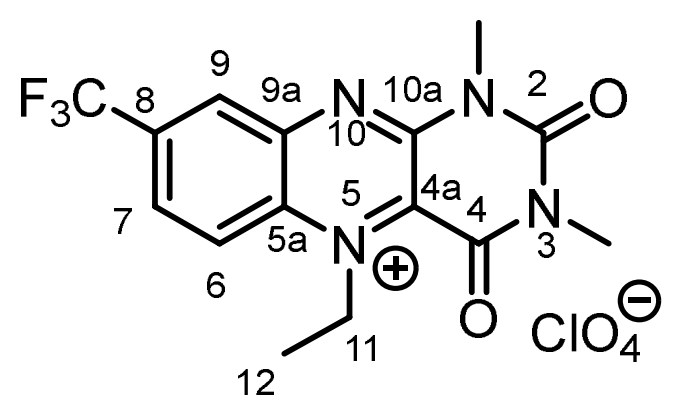

7c

Compound **7c** was prepared *via* a modified literature procedure.[11]

In a 250 mL *Schlenk* flask 1,3-dimethyl-8-(trifluoromethyl)alloxazine **SI-17c** (415 mg, 1.34 mmol, 1.00 equiv.) and Pd/C (10 wt.% Pd, 220 mg, 10 mol%) were added. After purging with argon three times, the solids were suspended in degassed EtOH (25 mL) and degassed H_2O (25 mL). Concentrated hydrochloric acid (37 wt.%, 4.17 g, 3.50 mL, 42.3 mmol, 20.5 equiv.) and acetaldehyde (3.18 g, 4.06 mL, 72.2 mmol, 35.0 equiv.) were added to the reaction mixture. A balloon was purged with argon thrice, filled with hydrogen gas and used to hydrogenate the reaction mixture. After stirring at room temperature for 19 h, the suspension was filtered into a 250 mL *Schlenk* flask using *Whatman* filtration and subsequently the solvents were removed *in vacuo*. Under ice bath cooling and stirring, aq. $HClO_4$ (2 M, 10 mL), $NaNO_2$ (585 mg, 8.48 mmol, 6.33 equiv.) and $NaClO_4$ (1.79 g, 14.6 mmol, 10.9 equiv.) were successively added to the remaining yellow solid. After 20 minutes of stirring at 0 °C the solid of the suspension was collected by filtration and successively washed with aq. $HClO_4$ (2 M, 3 x 10 mL) and cold water (3 x 15 mL). The precipitate was dried *in vacuo*, transferred to a 25 mL round flask and dissolved in a minimal amount of dry acetonitrile. After crashing it out with diethyl ether (10 mL), the precipitate was collected by filtration, washed with diethyl ether (3 x 10 mL) and dried *in vacuo*, yielding **7c** (408 mg, 930 µmol, 69%) as a yellow solid.

Yellow solid, 408 mg (69%); **^{1}H NMR** (500 MHz, CD_3CN, 298 K): δ (ppm) = 8.74 (d, $^3J_{H-H}$ = 9.5 Hz, 1H, H^6), 8.69 (m, 1H, H^9), 8.39 (dd, $^3J_{H-H}$ = 9.5 Hz, $^4J_{H-H}$ = 2.2 Hz, 1H, H^7), 3.83

(s, 3H, N^1-C*H*$_3$), 3.52 (s, 3H, N^3-C*H*$_3$), 1.81 (t, $^3J_{H-H}$ = 7.2 Hz, 3H, H^{12}), the signal of CH_2 group (5.74 ppm) is very broad, often not visible; **^{13}C{^{1}H} NMR** (126 MHz, CD_3CN, 298 K): δ (ppm) = 156.0 (C^4), 149.6 (C^2), 149.5 (C^{10a}), 147.2 (C^{9a}), 137.2 (q, $^2J_{C-F}$ = 34.3 Hz, C^8), 132.1 (C^{5a}), 131.2 (q, $^3J_{C-F}$ = 3.0 Hz, C^7), 129.1 (q, $^3J_{C-F}$ = 4.3 Hz, C^9), 123.4 (q, $^1J_{C-F}$ = 273 Hz, *C*F_3), 123.1 (C^6), 124.8 (C^{4a}), 53.7 (C^{11}), 31.8 (N^1-*C*H_3), 30.9 (N^3-*C*H_3), 15.5 (C^{12}); **HR-MS** (ESI^+): *m/z* calculated for $[C_{12}H_{11}N_4O_3]^+$: 339.106 ($[M]^+$); found: 339.106; **IR:** $\tilde{\nu}$ = 3399 (br), 3094 (w, sp^2-CH), 2959 (w), 1731 (m), 1686 (m, C=O), 1635 (w, C=O), 1596 (w), 1567 (m), 1514 (w), 1478, 1424, 1375, 1345, 1314, 1301, 1281, 1243, 1228, 1206, 1172, 1136, 1112, 1082, 974, 965, 943, 917, 889, 832, 819, 800, 760, 748, 698, 674 cm^{-1}.

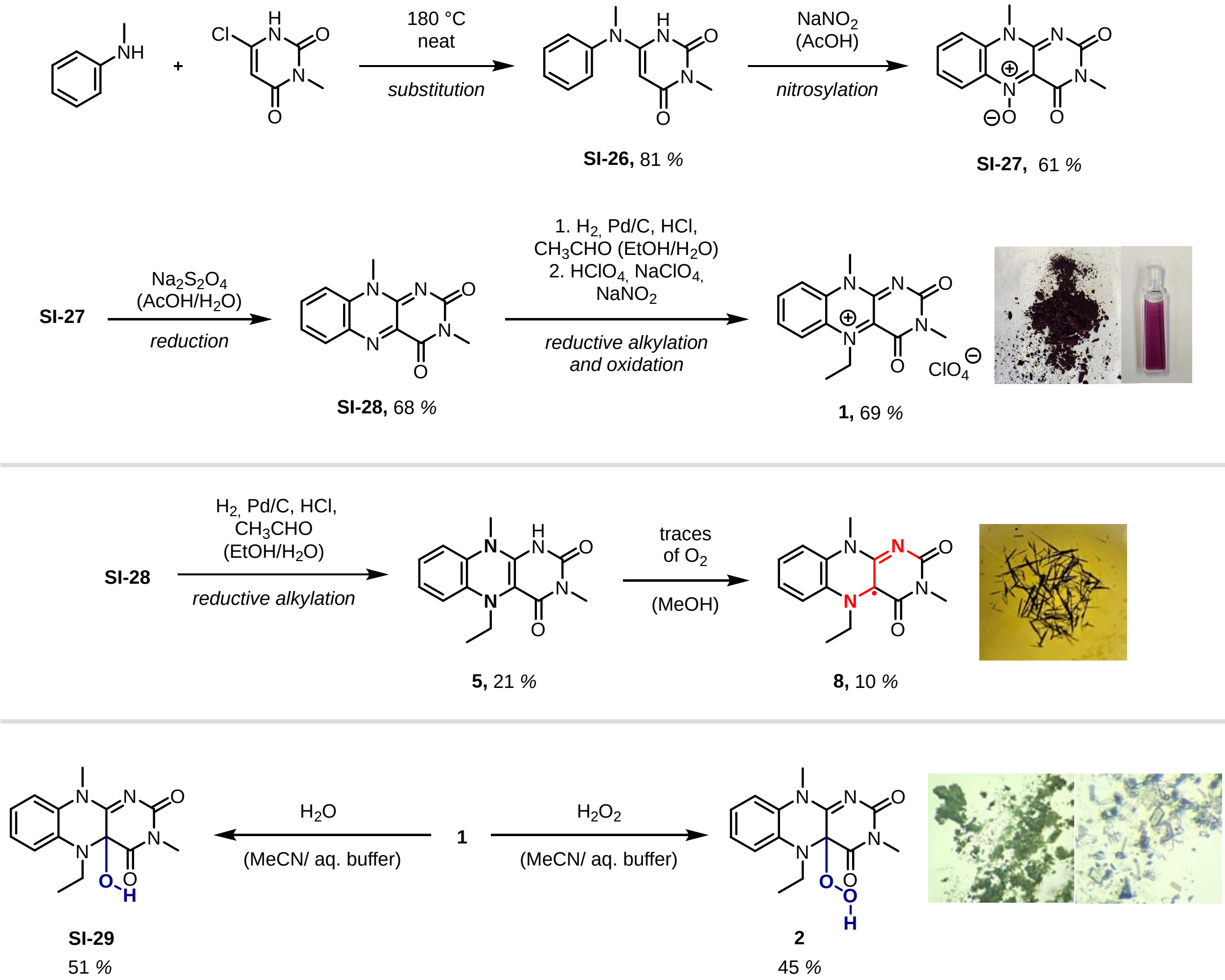

Figure SI-13. Catalyst synthesis of isoalloxazinium perchlorate **1**, its reduced form **5**, and its semi-reduced radical form **8**; precipitation reactions of **1** with water (**SI-29**) and hydrogen peroxide (**2**).

2.2.15 3-Methyl-6-(methyl(phenyl)amino)pyrimidine-2,4(1*H*,3*H*)-dione (SI-26)

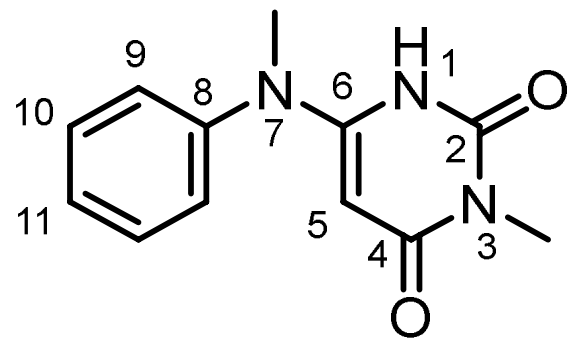

SI-26

Compound **SI-26** was prepared *via* a modified literature procedure.[15]

6-Chlorouracil (4.01 g, 25.0 mmol, 1.00 equiv.) was added to a 100 mL *Schlenk* tube, evacuated, and refilled with argon twice. Under inert conditions, *N*-methylaniline (10.7 g, 10.8 mL, 100 mmol, 4.00 equiv.) was added, and the suspension was heated to 180 °C, causing the chlorouracil to melt. The reaction mixture was stirred for 30 minutes at 180 °C under an argon atmosphere. The solution was then allowed to cool to room temperature overnight, and the resulting white crude solid was suspended in 50 mL of diethyl ether. The crude suspension was stirred and sonicated for a few minutes to pulverize the solid pieces. The solid of the suspension was then collected by filtration and washed with diethyl ether (3 × 50 mL, removal of excess amine) and water (3 × 50 mL, removes *N*-methylbenzenaminium chloride). The crude product was dried under reduced pressure, yielding 4.66 g of the target compound **SI-26** (20.2 mmol, 81%) as a white powder.

White solid, 4.66 g (81%); **TLC** (DCM/MeOH, 95/5): R_f = 0.47 [UV]; **^{1}H NMR** (500 MHz, DMSO-d_6, 298 K): δ (ppm) = 10.57 (d, 1H, $^4J_{H\text{-}H}$ = 2.5 Hz, H^1), 7.48 – 7.42 (m, 2H, H^{10}), 7.35 – 7.29 (m, 1H, H^{11}), 7.29 – 7.25 (m, 2H, H^9), 4.44 (d, 1H, $^4J_{H\text{-}H}$ = 2.5 Hz, H^5), 3.25 (s, 3H, N^7-CH_3), 3.06 (s, 3H, N^3-CH_3); **^{13}C{^{1}H} NMR** (126 MHz, DMSO-d_6, 298 K): δ (ppm) = 162.8 (C$^{2/4}$), 153.5 (C^6), 151.4 (C$^{2/4}$), 144.2 (C^8), 129.9 (C^{10}), 127.0 (C^{11}), 126.48 (C^9), 78.3 (C^5), 39.4 (N^7-CH$_3$)*, 26.2 (N^3-CH$_3$); **HR-MS** (ESI$^+$): *m/z* calculated for $[C_{12}H_{13}N_3O_2+H]^+$: 232.1081 ($[M+H]^+$); found: 232.1078; **IR:** $\tilde{\nu}$ = 3173 (br w, NH), 3057 (w, sp^2-CH), 2960 (w, sp^3-CH), 1694 (m, C=O carbamate), 1638 (s, C=O amide), 1607 (s, C=C), 1585 (s), 190 (s), 1451 (s, sp^3-CH), 1369 (s), 1341 (s), 985 (m), 778 (m), 765 (s), 744 (m, sp^2-CH), 693 (s), 679 (m) cm^{-1}.

* Due to signal overlap with the solvent, the presence of the peak was confirmed by HSQC.

2.2.16 3,10-Dimethyl-2,4-dioxo-2,3,4,10-tetrahydrobenzo[*g*]pteridine 5-oxide (SI-27)

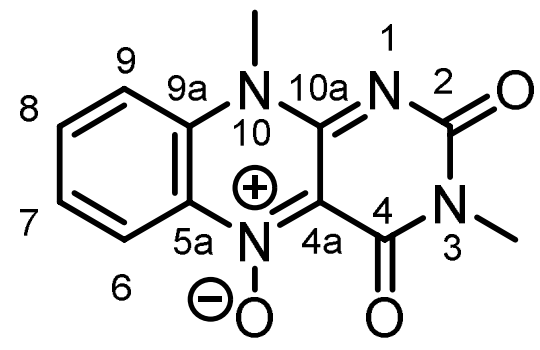

SI-27

Compound **SI-27** was prepared *via* a modified literature procedure.[15]

To a solution of **SI-26** (4.64 g, 20.1 mmol, 1.00 equiv.) in glacial acetic acid (40 mL, 0.5 M) was added $NaNO_2$ (6.92 g, 100 mmol, 5.00 equiv.) in a 100 mL round bottom flask (open to air). The reaction mixture was stirred for 2 h at room temperature. 20 mL of water were added to the mixture, the crude material was filtered and washed with water (3 × 50 mL). The resulting orange solid was dried under reduced pressure to yield the title compound **SI-27** (3.17 g, 12.3 mmol, 61%) as bright orange powder.

Orange solid, 3.17 g (61%); **TLC** (DCM/MeOH, 95/5): R_f = 0.40 [colored compound]; **^{1}H NMR** (500 MHz, $CDCl_3$, 298 K): δ (ppm) = 8.52 (dd, $^3J_{H\text{-}H}$ = 8.5 Hz, $^4J_{H\text{-}H}$ = 1.5 Hz, 1H, H^6), 7.90 (ddd, $^3J_{H\text{-}H}$ = 8.6, 7.2 Hz, $^4J_{H\text{-}H}$ = 1.5 Hz, 1H, H^8), 7.67 (dd, $^3J_{H\text{-}H}$ = 8.6 Hz, $^4J_{H\text{-}H}$ = 1.1 Hz, 1H, H^9), 7.56 (ddd, $^3J_{H\text{-}H}$ = 8.5, 7.2 Hz, $^4J_{H\text{-}H}$ = 1.1 Hz, 1H, H^7), 4.07 (s, 3H, N^{10}-CH_3), 3.45 (s, 3H, N^3-CH_3); **^{13}C{^{1}H} NMR** (101 MHz, $CDCl_3$, 298 K): δ [ppm] = 156.4 ($C^{2/4}$), 155.1 ($C^{2/4}$), 151.8 ($C^{4a/10a}$), 136.0 (C^8), 135.0 ($C^{5a/9a}$), 134.7 ($C^{5a/9a}$), 126.2 (C^7), 125.1 ($C^{4a/10a}$), 122.3 (C^6), 116.3 (C^9), 32.1 (N^{10}-CH_3), 28.4 (N^3-CH_3); **HR-MS** (ESI$^+$): *m/z* calculated for $[C_{12}H_{10}N_4O_3+H]^+$: 259.0826 ($[M+H]^+$); found: 259.0823; **IR:** $\tilde{\nu}$ = 3078 (w, sp^2-CH), 1646 (s, C=O amide), 1535 (s, N-O asymmetric), 1500 (s), 1417 (s, C=C), 1375 (m, N-O symmetric), 1274-1176 (s, sp^2-CH), 765-756 (s, sp^2-CH) cm^{-1}.

2.2.17 3,10-Dimethylbenzo[*g*]pteridine-2,4(3*H*,10*H*)-dione (SI-28)

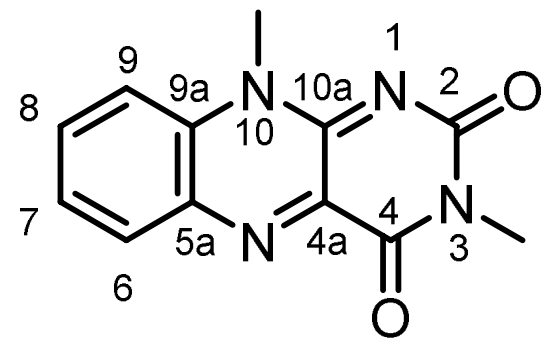

SI-28

Compound **SI-28** was prepared *via* a modified literature procedure. [15]

In a 100 mL round bottom flask (open to air), a suspension of N-oxide **SI-27** (3.12 g, 12.1 mmol, 1.00 equiv.) in glacial acetic acid (40 mL, 0.30 M) was prepared. A solution of $Na_2S_2O_4$ (12.6 g, 72.5 mmol, 6.00 equiv.) in water (20 mL, 3.63 M) was added in one go, turning the suspension from orange to brown. The reaction mixture was stirred for 24 h at room temperature open to air. The solid was collected by filtration and successively washed with acetic acid (3 x 15 mL) and water (3 x 15 mL). The precipitate was dried *in vacuo*, yielding **SI-28** (2.00 g, 8.26 mmol, 68%) as a yellow solid.

Yellow powder, 2.00 g (68%); **TLC** (DCM/MeOH, 95/5): R_f = 0.39 [colored compound]; **^{1}H NMR** (500 MHz, $CDCl_3$, 298 K): δ (ppm) = 8.35 (m, 1H, H^6), 7.92 (m, 1H, H^7), 7.69 – 7.62 (m, 2H, $H^{8,9}$), 4.14 (s, 3H, N^{10}-CH_3), 3.54 (s, 3H, N^3-CH_3); **^{13}C{^{1}H} NMR** (126 MHz, $CDCl_3$, 298 K): δ (ppm) = 159.8 ($C^{2/4}$), 155.9 ($C^{2/4}$), 149.4 (C^{10a}), 137.1 (C^{4a}), 136.0 (C^{5a}), 135.8 (C^7), 133.5 (C^6), 133.5 (C^{9a}), 126.8 (C^8), 115.3 (C^9), 32.2 (N^{10}-*C*H_3), 29.0 (N^3-*C*H_3); **HR-MS** (ESI^+): *m/z* calculated for $[C_{12}H_{10}N_4O_2+H]^+$: 243.0877 ($[M+H]^+$); found: 243.0875; **IR:** $\tilde{\nu}$ = 3049 (w, sp^2-CH), 1645 (s, C=O amide), 1612 (s, C=C), 1550 (s), 1515 (s, C=C), 1464 (m, sp^3-CH), 1423 (s), 1276-1132 (s, sp^2-CH), 967 (s), 778-712 (s, sp^2-CH) cm^{-1}.

2.2.18 5-Ethyl-3,10-dimethyl-2,4-dioxo-2,3,4,10-tetrahydrobenzo[*g*]pteridin-5-ium perchlorate (1)

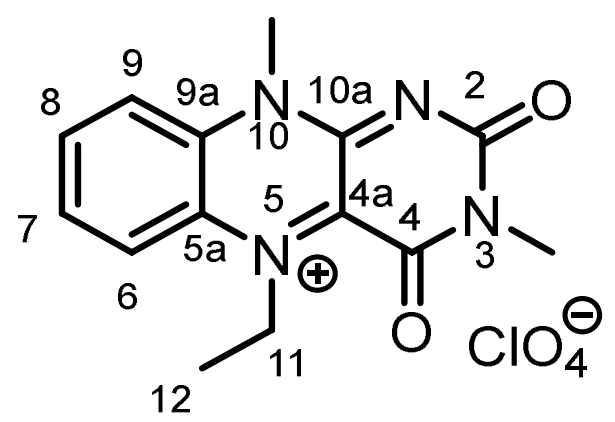

1

Compound **1** was prepared *via* a modified literature procedure.[16]

In a 250 mL *Schlenk* flask 3,10-dimethylisoalloxazine **SI-28** (500 mg, 2.06 mmol, 1.00 equiv.) and Pd/C (10 wt.% Pd, 220 mg, 10 mol%) were evacuated and carefully purged with argon three times. The solids were suspended in degassed EtOH (30 mL) and degassed H_2O (30 mL). Concentrated hydrochloric acid (37 wt.%, 4.17 g, 3.50 mL, 42.3 mmol, 20.5 equiv.) and acetaldehyde (3.18 g, 4.06 mL, 72.2 mmol, 35.0 equiv.) were added to the reaction mixture. An oxygen free hydrogen balloon (1 bar) was attached and the reaction mixture was sparged with hydrogen gas for approximately 30 seconds. Then a freshly filled hydrogen ballon was attached and the reaction mixture was stirred for 16 h under H_2 atmosphere at room temperature. Hereafter, the light-yellow suspension was filtered into a 250 mL *Schlenk* flask using *Whatman* filtration and subsequently the solvents were removed *in vacuo* at 50 °C. Under ice bath cooling and stirring, $HClO_4$ (2 M, 10 mL), $NaNO_2$ (900 mg, 13.0 mmol, 6.33 equiv.) and $NaClO_4$ (2.75 g, 22.5 mmol, 10.9 equiv.) were successively added to the remaining yellow solid. Precipitation was observed immediately, changing color from yellow to red to dark brown. After 20 minutes of stirring at 0 °C the solid was collected by filtration and washed with cooled water (5 x 15 mL). The precipitate was dried *in vacuo*, yielding **1** (383 mg, 1.41 mmol, 69%) as a dark violet solid.

Dark violet solid, 383 mg (69%); **^{1}H NMR** (500 MHz, CD_3CN, 298 K): δ (ppm) = 8.48 (dd, $^3J_{H\text{-}H}$ = 9.2 Hz, $^4J_{H\text{-}H}$ = 1.1 Hz, 1H, H^6), 8.24 (ddd, $^3J_{H\text{-}H}$ = 8.8, 7.0 Hz, $^4J_{H\text{-}H}$ = 1.1 Hz, 1H, H^8), 8.12 (dd, $^3J_{H\text{-}H}$ = 8.8 Hz, $^4J_{H\text{-}H}$ = 1.2 Hz, 1H, H^9), 7.93 (ddd, $^3J_{H\text{-}H}$ = 9.1, 7.0 Hz, $^4J_{H\text{-}H}$ = 1.2 Hz, 1H, H^7), 6.21 – 5.98 (m, 1H, H^{11}), 5.15 – 4.93 (m, 1H, H^{11}), 4.18 (s, 3H, N^{10}-C*H*$_3$), 3.42 (s, 3H, N^3-C*H*$_3$), 1.80 (t, $^3J_{H\text{-}H}$ = 7.1 Hz, 3H); **^{13}C{^{1}H} NMR** (126 MHz, CD_3CN, 298 K): δ (ppm) = 156.6 (C^4), 153.4 (C^2), 150.7 (C^{10a}), 140.1 (C^8), 138.4 (C^{9a}), 133.9 (C^{4a}), 130.9 (C^7), 128.1 (C^{5a}), 123.1 (C^6), 119.8 (C^9), 53.7 (C^{11}), 35.7 (N^{10}-*C*H_3), 30.2 (N^3-*C*H_3), 15.5 (C^{12}); **HR-MS** (ESI$^+$): *m/z* calculated for $[C_{14}H_{15}N_4O_2]^+$: 272.1268 ($[M]^+$); found: 272.1267; **IR:** $\tilde{\nu}$ = 3098 (br, sp^2-CH), 1714 (w, C=O), 1663 (s, C=O), 1612 (m), 1561 (s), 1460, 1423, 1376, 1345, 1317, 1282, 1238, 1204, 1189, 1163, 1147, 1127, 1080, 1019, 999, 966, 927, 860, 839, 797, 762, 746, 741, 685 cm^{-1}.

2.2.19 5-Ethyl-3,10-dimethyl-5,10-dihydrobenzo[*g*]pteridine-2,4(1*H*,3*H*)-dione (5)

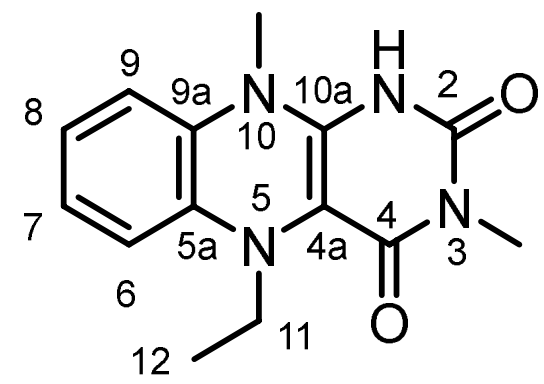

5

Compound **5** was prepared according to a modified literature procedure.[9] The compound is known in the literature.[17]

Isoalloxazine **SI-28** (500 mg, 2.06 mmol, 1.00 equiv.) and Pd/C (10 wt.% Pd, 220 mg, 206 μmol, 0.10 equiv.) were added to a 250 mL *Schlenk* flask, which was evacuated and refilled with argon three times. 31 mL of degassed H_2O, 28 mL of degassed EtOH, 3.5 mL of concentrated hydrochloric acid (37%), and 3.48 mL of acetaldehyde (2.73 g, 61.9 mmol, 30.0 equiv.) were added. The reaction mixture was hydrogenated by H_2 bubbling and stirred overnight under H_2 atmosphere. After *Whatman* filtration under Ar, aqueous NH_3 (25%, 7 mL) was added to the yellow solution to adjust the pH to 7. The solvents were removed *in vacuo* using an external cooling trap. Degassed H_2O (4 mL) was added, and the mixture was stirred for 20 minutes. The solvent was removed by *Whatman* filtration and the solid was washed once more with degassed H_2O (4 mL) that was subsequently removed via *Whatman* filtration. All volatiles were removed in vacuo to give 120 mg dihydroisoalloxazine **5** (439 μmol, 21%) as light green powder. Since **5** is very sensitive towards molecular oxygen, the *Schlenk* flask was brought into the glovebox for transferring and weighing the product into a small dram vial. An NMR sample was prepared in degassed $CDCl_3$ with a very thin layer of degassed D_2O solution of $Na_2S_2O_4$ on top.

Light green powder, 120 mg (21%); **^{1}H NMR** (400 MHz, $CDCl_3$, 298 K): δ (ppm) = 10.64 (br s, 1H, N^1*H*), 6.99 – 6.86 (m, 3H, H$^{7-9}$), 6.62 (dd, $^3J_{H-H}$ = 8.1 Hz, $^4J_{H-H}$ = 1.7 Hz, 1H, H^6), 3.47 (q, $^3J_{H-H}$ = 7.0 Hz, 2H, H^{11}), 3.31 (s, 3H, N^3-C*H*$_3$), 3.27 (s, 3H, N^{10}-C*H*$_3$), 1.14 (t, $^3J_{H-H}$ = 7.0 Hz, 3H, H^{12}); **^{13}C{^{1}H} NMR** (101 MHz, $CDCl_3$, 298 K): δ (ppm) = 158.6 (C^2), 151.9 (C^4), 147.4 (C^{10a}), 139.6 (C^{9a}), 138.9 (C^{5a}), 124.7 (C^7), 123.7 (C^8), 122.7 (C^9), 113.0 (C^6), 101.4 (C^{4a}), 50.4 (C^{11}), 31.9 (N^{10}-*C*H$_3$), 27.4 (N^3-*C*H$_3$), 11.8 (C^{12}).

2.2.20 Semiquinone of 5 (8)

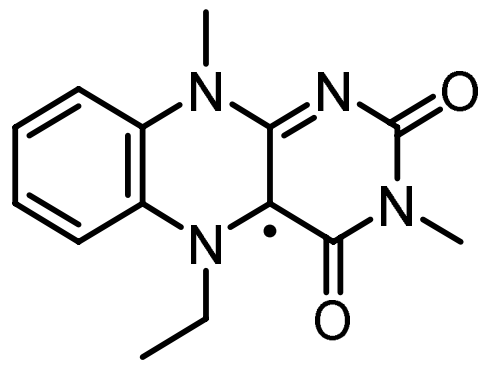

8

Dihydroisoalloxazine **5** (20.0 mg, 73.4 µmol, 1.00 equiv.) was mostly dissolved in MeOH (dry, degassed, 1 mL). The suspension was filtered under argon in a new *Schlenk* flask. With a syringe, 0.5 mL of air were bubbled into the solution, causing the solution to turn slightly very dark turquoise, and the flask was stored in the fridge. After 24 h, dark, needle shaped crystals formed from the solution. The crystals were collected by filtration and dried to yield 2.00 mg (7.38 µmol, 10%) of radical **8**. Upon storage under inert conditions no oxidation was observed.

Dark fine needle shaped crystals, 2.00 mg (7.38 µmol, 10%); The compound was characterized by EPR and SC-XRD.

2.2.21 5-Ethyl-3,10-dimethyl-5,10-dihydrobenzo[g]pteridine-2,4(1H,3H)-dione (2)

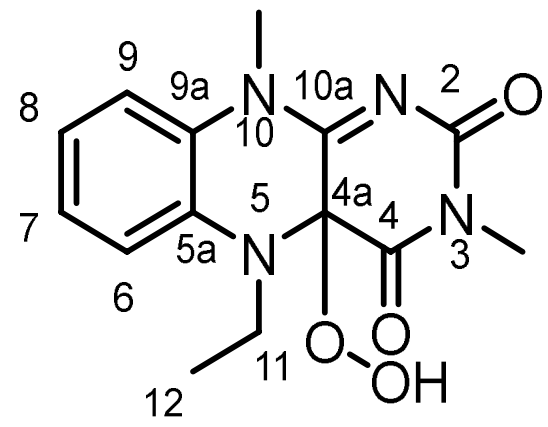

2

Compound **2** was prepared according to a modified literature procedure.[18]

Flavinium salt **1** (100 mg, 270 µmol, 1.00 equiv) was dissolved as completely as possible in dry acetonitrile (1 mL) to obtain a saturated solution. Separately, $NaH_2PO_4(H_2O)_7$ (570 mg) and $Na_2HPO_4(H_2O)$ (30.0 mg) were dissolved in aqueous H_2O_2 (10 mL, 4 M), affording a buffer solution at approximately pH 5. The flavinium solution was added to the H_2O_2 buffer solution in a centrifuge tube, resulting in immediate discoloration and formation of a light green–yellow precipitate. The suspension was centrifuged, and the supernatant was decanted. The residue was washed once with aqueous H_2O_2 solution (5 mL, 4 M, without buffer) and subsequently with a small amount of chilled methanol. The solid was dried *in vacuo* to afford flavin hydroperoxide **2** (37.0 mg, 121 µmol, 45%). A clean NMR spectrum of the compound was obtained in acetone-d_6.

Green solid, 37.0 mg (45%); **^{1}H NMR** (500 MHz, acetone-d_6, 298 K): δ (ppm) = 11.52 (s, 1H, O*H*), 7.40 (d, $^3J_{H-H}$ = 8.1 Hz, 1H, H^6), 7.25 – 7.17 (m, 2H, $H^{8,9}$), 7.09 – 6.99 (m, 1H, H^7), 3.96 (dq, $^2J_{H-H}$ = 15.4 Hz, $^3J_{H-H}$ = 7.0 Hz, 1H, H^{11}), 3.75 (s, 3H, N^{10}-CH_3), 3.77 – 3.66 (m, 1H, H^{11}), 3.24 (s, 3H. N^3-CH_3), 1.44 (t, $^3J_{H-H}$ = 7.0 Hz, 3H, H^{12}); **^{13}C{^{1}H} NMR** (126 MHz, acetone-d_6, 298 K): δ (ppm) = 164.0 ($C^{2/4}$), 156.9 (C^{10a}), 155.3 ($C^{2/4}$), 133.9 (C^{5a}), 128.2 (C^{9a}), 126.0 ($C^{8/9}$), 121.0 (C^7), 116.9 (C^6), 114.8 ($C^{8/9}$), 85.3 (C^{4a}), 43.3 (C^{11}), 32.7 (N^{10}-CH_3), 28.7 (N^3-CH_3), 14.7 (C^{12}); **MS** (EI, 70 eV): *m/z* (%) = 288 (71), 259 (5), 243 (10), 230 (25), 202 (100), 175 (30), 147 (70), 131 (16), 119 (26), 90 (16); **HR-MS** (EI, 70 eV): *m/z* calculated for $[C_{14}H_{16}O_4N_4]^{\bullet+}$: 304.1166 ($[M]^{\bullet+}$); found: 304.1169, *m/z* calculated for $[C_{13}{}^{13}CH_{16}O_4N_4]^{\bullet+}$: 305.1200 ($[M]^{\bullet+}$); found: 305.1205; **IR:** $\tilde{\nu}$ = 3202 (w, OH), 3000 (w, sp^2-CH), 2941 (w, sp^3-CH), 2339 (w), 1718 (s, C=O), 1656 (s, C=O), 1609 (m, C=C), 1599 (m, C=C), 1568 (m, C=C), 1509 (m, C=C), 1489 (m, C=C), 1468 (m), 1443 (m), 1422 (m), 1408 (m), 1362 (m), 1332 (m), 1309 (m), 1270 (s, C–O), 1243 (m), 1194 (m), 1182 (m), 1140 (m), 1091 (m), 1083 (m), 1054

(m), 1039 (m), 1000 (m), 963 (m), 902 (m), 874 (m), 849 (m), 818 (m), 764 (s), 754 (s), 724 (m), 707 (m), 684 (m) cm^{-1}.

2.2.22 5-Ethyl-4a-hydroxy-3,10-dimethyl-5,10-dihydrobenzo[*g*]pteridine-2,4(3*H*,4a*H*)-dione (SI-29)

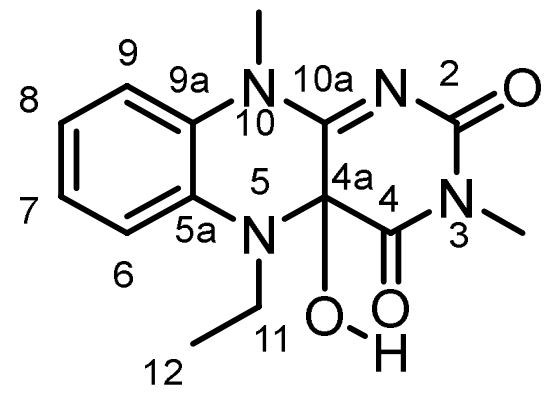

SI-29

Compound **SI-29** was prepared according to a modified literature procedure.[14]

Certipur® buffer solution (pH = 7, phosphate buffer, 2.20 mL) was added to a solution of flavinium salt **1** (40.0 mg, 108 µmol, 1.00 equiv) in acetonitrile (0.5 mL) and water (50 µL). The reaction mixture was left at room temperature for 5 minutes upon which a green precipitate formed. The suspension was centrifuged, and the supernatant was decanted. The residue was washed with water once and dried *in vacuo* to afford **SI-29** as a green solid (15.9 mg, 55.0 µmol, 51%).

Green solid (15.9 mg, 55.0 µmol, 51%); **^{1}H NMR** (500 MHz, acetone-d_6, 298 K): δ (ppm) = 7.41 (dd, $^{3}J_{H\text{-}H}$ = 8.1 Hz, $^{4}J_{H\text{-}H}$ = 1.4 Hz, 1H, H^{6}), 7.26 (dd, $^{3}J_{H\text{-}H}$ = 8.2 Hz, $^{4}J_{H\text{-}H}$ = 1.5 Hz, 1H, H^{9}), 7.21 (ddd, $^{3}J_{H\text{-}H}$ = 8.2, 7.2 Hz, $^{4}J_{H\text{-}H}$ = 1.4 Hz, 1H, H^{8}), 7.10 (ddd, $^{3}J_{H\text{-}H}$ = 8.1, 7.2 Hz, $^{4}J_{H\text{-}H}$ = 1.5 Hz, 1H, H^{7}), 6.22 (s, 1H, OH), 3.77 (dq, $^{2}J_{H\text{-}H}$ = 15.0 Hz, $^{3}J_{H\text{-}H}$ = 7.0 Hz, 1H, H^{11}), 3.70 (s, 3H, N^{10}-CH_3), 3.56 (dq, $^{2}J_{H\text{-}H}$ = 15.0 Hz, $^{3}J_{H\text{-}H}$ = 7.0 Hz, 1H, H^{11}), 3.26 (s, 3H, N^{3}-CH_3), 1.19 (t, $^{3}J_{H\text{-}H}$ = 7.0 Hz, 3H, H^{12}); **^{13}C{^{1}H} NMR** (126 MHz, acetone-d_6, 298 K): δ (ppm) = 167.1 ($C^{2/4}$), 159.3 (C^{10a}), 155.2 ($C^{2/4}$), 133.9 (C^{5a}), 131.0 (C^{9a}), 125.8 (C^{8}), 122.4 (C^{7}), 119.2 (C^{9}), 117.1 (C^{6}), 75.4 (C^{4a}), 44.7 (C^{11}), 32.6 (N^{10}-CH_3), 28.3 (N^{3}-CH_3), 14.4 (C^{12}); **MS** (EI, 70 eV): *m/z* (%) = 288 (67), 271 (5), 243 (8), 232 (15), 202 (100), 175 (39), 147 (91), 131 (17), 119 (28), 90 (16); **HR-MS** (EI, 70 eV): *m/z* calculated for $[C_{14}H_{16}O_3N_4]^{\bullet+}$: 288.1217 ($[M]^{\bullet+}$); found: 288.1236, *m/z* calculated for $[C_{13}{}^{13}CH_{16}O_3N_4]^{\bullet+}$: 289.1250 ($[M]^{\bullet+}$); found: 289.1273 **IR:** $\tilde{\nu}$ = 3204 (w, O–H), 3000 (w, sp^2-CH), 2941 (w, sp^3-CH), 1718 (s, C=O), 1656 (m, C=O), 1609 (m, C=C), 1599 (m, C=C), 1568 (m, C=C), 1509 (m, C=C), 1489 (m, C=C), 1468 (m), 1422 (m), 1408 (m), 1362 (m), 1332 (m), 1309 (m), 1270 (m), 1243 (m, C–O), 1194 (m), 1182 (m), 1140 (m), 1091 (m), 1082 (m), 1054

(m), 1039 (m), 999 (m), 963 (m), 902 (m), 874 (w), 849 (w), 818 (w), 764 (s), 753 (m), 724 (m), 707 (m), 684 (m).

2.3 Catalysis Products

Catalytic reactions:

As multiple catalytic reactions were conducted simultaneously, stock solutions were prepared to ensure the accurate quantities of the used chemicals. The amounts specified below correspond to the quantity required for one catalytic reaction.

General procedure A (alloxazinium salt catalyzed *Witkop* oxidation)

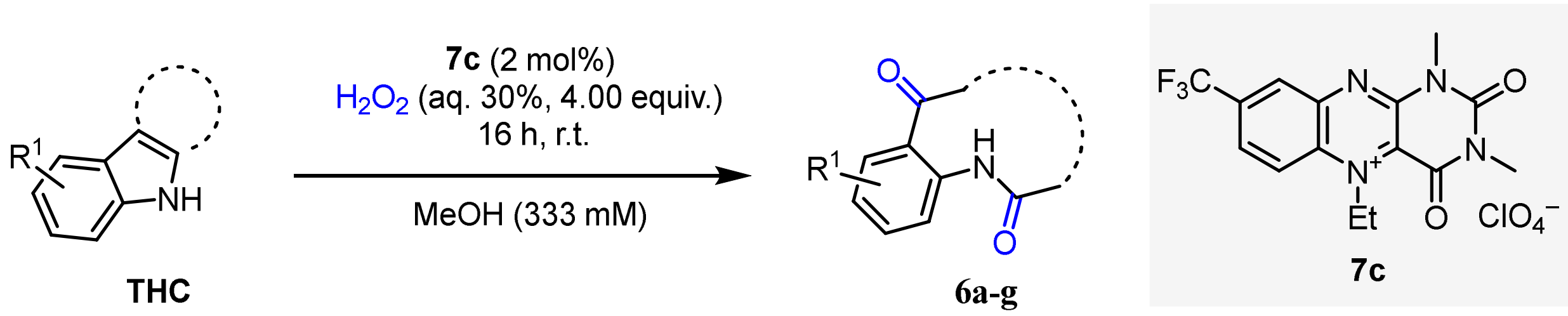

Catalyst **7c** (438.7 µg, 1.00 µmol, 0.02 equiv.) and 30 vol-% aqueous H_2O_2 (20.4 µL, 200 µmol, 4.00 equiv.) in 150 µL methanol (333 mM) were added to the respective catalysis substrate **THC** (50.0 µmol, 1.00 equiv.) in a GC-MS vial. The reaction mixture was stirred for 16 – 48 h (depending on substrate) at room temperature. Subsequently, the solvent was gently removed under a flow of nitrogen, and the crude residue was dissolved in a minimal amount of acetone and loaded on a *Biotage* C18 samplet. Purification by reverse-phase flash chromatography (CH_3CN/H_2O = 1/9 → 100% CH_3CN) and removal of the solvents by lyophilization using a freeze dryer afforded ketolactams **6a**-**g**.

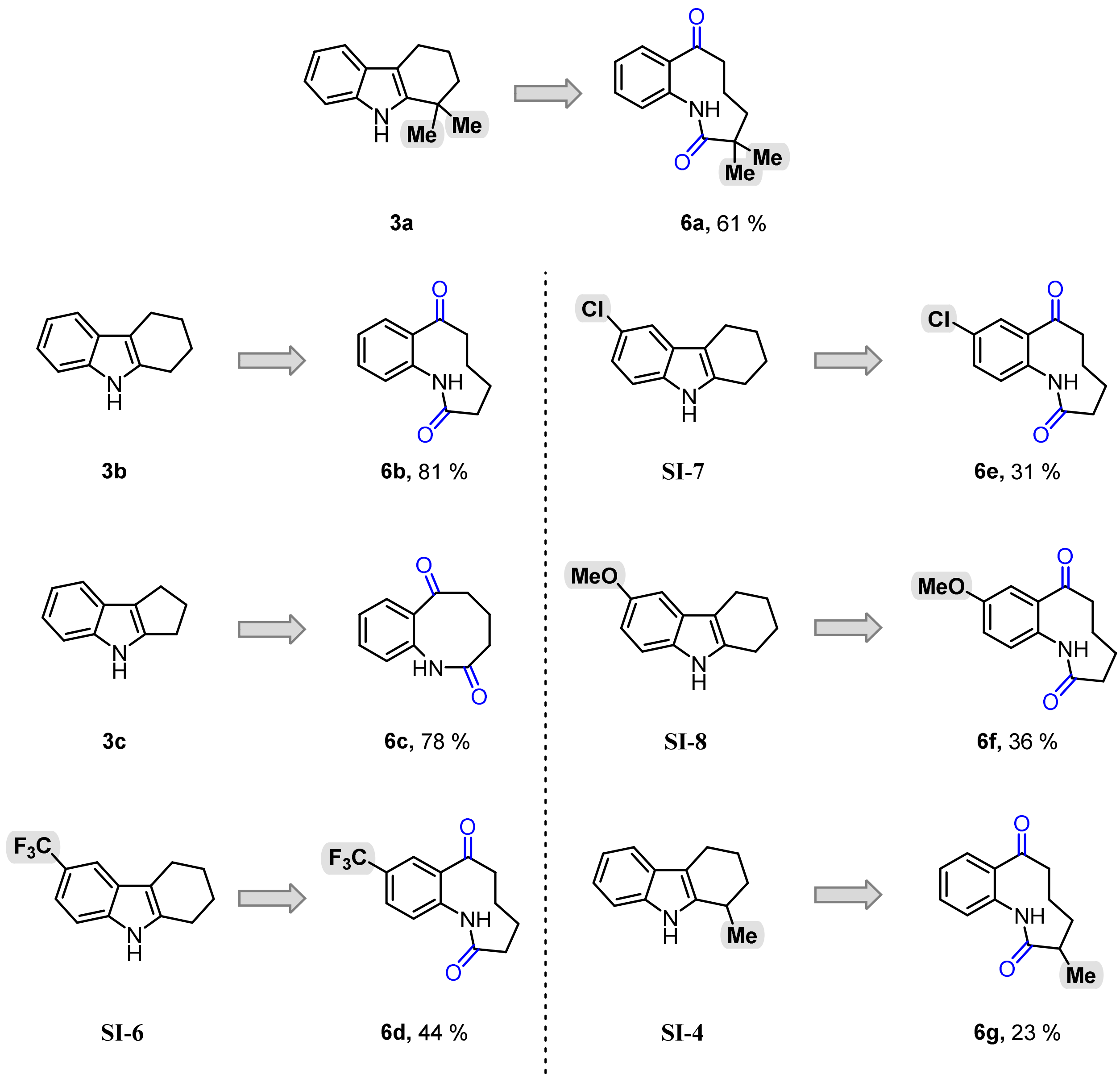

Figure SI-14. Substrate – catalysis product overview of the alloxazinium salt catalyzed *Witkop* oxidation. All yields refer to isolated ones.

2.3.1 3,3-Dimethyl-3,4,5,6-tetrahydro-1*H*-benzo[*b*]azonine-2,7-dione (6a)

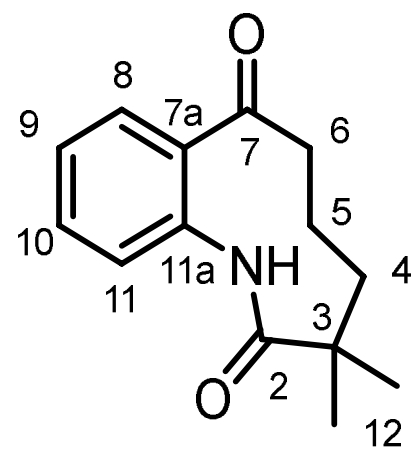

6a

According to general procedure **A**, tetrahydrocarbazole **3a** (9.96 mg, 50.0 µmol) was used to prepare ketolactam **6a**.

Colorless solid (7.05 mg, 30.5 µmol, 61%); **TLC** (CH_2Cl_2/MeOH, 95/5): R_f = 0.46 [UV]; **^{1}H NMR** (500 MHz, $CDCl_3$, 298 K): δ (ppm) = 7.53 – 7.48 (m, 1H, H^{10}), 7.46 – 7.42 (m, 1H, H^8), 7.37 – 7.32 (m, 1H, H^9), 7.31 (br s, 1H, NH), 7.23 (m, 1H, H^{11}), 2.64 – 2.57 (m, 2H, H^6), 2.05 –2.00 (m, 2H, H^5), 1.80 – 1.74 (m, 2H, H^4), 1.31 (s, 6H, H^{12}). **^{13}C{^{1}H} NMR** (126 MHz, $CDCl_3$, 298 K): δ (ppm) = 206.4 (C^7), 177.2 (C^2), 139.9 ($C^{7a/11a}$), 135.3 ($C^{7a/11a}$), 131.7 ($C^{9/10}$), 127.7 ($C^{8/11}$), 127.5 ($C^{8/11}$), 127.4 ($C^{9/10}$), 43.9 (C^4), 42.6 (C^6), 42.2 (C^3), 25.0 (2C, C^{12}), 21.6 (C^5). **HR-MS** (ESI^+): *m/z* calculated for $[C_{14}H_{17}NO_2+H]^+$: 232.1332 ($[M+H]^+$); found: 232.1331; **IR:** $\tilde{\nu}$ = 3202 (w, O–H), 3157 (w, sp^2-CH), 3020 (w, sp^2-CH), 2948 (w, sp^3-CH), 2935 (w, sp^3-CH), 2862 (w, sp^3-CH), 1682 (m, C=O), 1665 (w, C=O), 1645 (m, C=C), 1601 (m, C=C), 1521 (m, C=C), 1482 (m), 1439 (m), 1351 (w), 1338 (m), 1306 (w), 1296 (w), 1274 (m), 1257 (w), 1242 (w), 1230 (m), 1213 (w), 1182 (m), 1154 (w), 1139 (m), 1108 (m), 1058 (m), 1050 (m), 1033 (m), 987 (w), 928 (m), 846 (m), 802 (m), 774 (m), 762 (m), 749 (s), 727 (m), 699 (m), 670 (m).

2.3.2 3,4,5,6-Tetrahydro-1*H*-benzo[*b*]azonine-2,7-dione (6b)

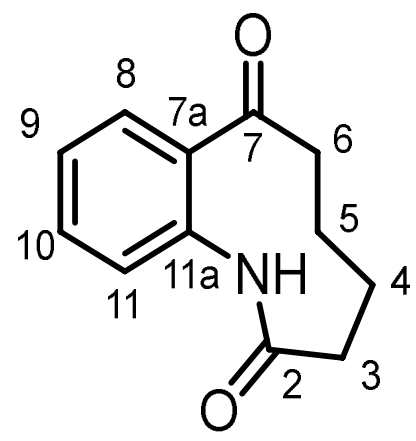

6b

According to general procedure **A**, tetrahydrocarbazole **3b** (8.56 mg, 50.0 µmol) was used to prepare ketolactam **6b**. The reaction was performed in EtOH (instead of MeOH). The analytical data of compound **6b** are consistent with literature data.[19]

Colorless solid (8.23 mg, 40.5 µmol, 81%); **^{1}H NMR** (500 MHz, $CDCl_3$, 298 K): δ (ppm) = 7.61 – 7.57 (m, 1H, $H^{8/9/10/11}$), 7.55 (br s, 1H, N*H*), 7.53 – 7.49 (m, 1H, $H^{8/9/10/11}$), 7.45 – 7.39 (m, 1H, $H^{8/9/10/11}$), 7.24 – 7.22 (m, 1H, $H^{8/9/10/11}$), 2.94 – 2.86 (m, 2H, $H^{3/6}$), 2.29 – 2.20 (m, 2H, $H^{3/6}$), 1.95 – 1.83 (s, 4H, $H^{4/5}$); **^{13}C{^{1}H} NMR** (126 MHz, $CDCl_3$, 298 K): δ (ppm) = 206.3 (C^7), 176.5 (C^2)*, 139.4 ($C^{7a/11a}$)*, 134.3 ($C^{7a/11a}$), 132.3 ($C^{8/9/10/11}$), 128.8 ($C^{8/9/10/11}$)*, 128.6 ($C^{8/9/10/11}$), 128.4 ($C^{8/9/10/11}$), 41.6 ($C^{3/6}$), 32.4 ($C^{3/6}$), 25.0 ($C^{4/5}$), 24.7 ($C^{4/5}$); **MS** (EI, 70 eV): *m/z* (%) = 203 (11), 184 (10), 175 (17), 156 (6), 146 (18), 135 (44), 130 (12), 120 (100), 92 (20); **HR-MS** (EI, 70 eV): *m/z* calculated for $[C_{12}H_{13}O_2N]^{\bullet+}$: 203.0941 ($[M]^{\bullet+}$); found: 203.0942, *m/z* calculated for $[C_{11}{}^{13}CH_{13}O_2N_1]^{\bullet+}$: 204.0974 ($[M]^{\bullet+}$); found: 204.0979; **IR**: $\tilde{\nu}$ = 3203 (m, NH), 3019 (w, sp^2-CH), 2935 (w, sp^3-CH), 2862 (w, sp^3-CH), 1682 (s, C=O), 1644 (s, C=O), 1601 (m, C=C), 1521 (m, C=C), 749 (s), 670 (m) cm^{-1}.

* low intensity in ^{13}C

2.3.3 4,5-Dihydrobenzo[*b*]azocine-2,6(1*H*,3*H*)-dione (6c)

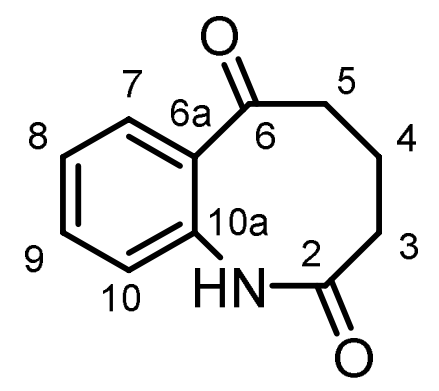

6c

According to general procedure **A**, tetrahydrocarbazole **3c** (7.86 mg, 50.0 µmol) was used to prepare ketolactam **6c**. The analytical data of compound **6c** are consistent with literature data.[20]

Colorless solid (7.38 mg, 39.0 µmol, 78%); **^{1}H NMR** (500 MHz, $CDCl_3$, 298 K): δ (ppm) = 8.18 (dd, 1H, $^3J_{H\text{-}H}$ = 8.2 Hz, $^4J_{H\text{-}H}$ = 1.7 Hz, H^7), 8.00 (br s, 1H, N*H*), 7.54 (ddd, 1H, $^3J_{H\text{-}H}$ = 7.7, 7.4 Hz, $^4J_{H\text{-}H}$ = 1.7 Hz, H^9), 7.33 (ddd, 1H, $^3J_{H\text{-}H}$ = 8.2, 7.4 Hz, $^4J_{H\text{-}H}$ = 1.2 Hz, H^8), 7.07 (dd, 1H, $^3J_{H\text{-}H}$ = 7.7 Hz, $^4J_{H\text{-}H}$ = 1.2 Hz, H^{10}), 3.08 – 3.01 (m, 2H, H^5), 2.52 (t, 2H, $^3J_{H\text{-}H}$ = 6.9 Hz, H^3), 2.23 (p, 2H, $^3J_{H\text{-}H}$ = 6.9 Hz, H^4); **^{13}C{^{1}H} NMR** (126 MHz, $CDCl_3$, 298 K): δ (ppm) = 199.9 (C^6), 174.8 (C^2), 138.1 (C^{10a}), 134.4 (C^9), 131.4 (C^7), 130.8 (C^{6a}), 126.3 (C^8), 125.1 (C^{10}), 39.3 (C^5), 31.1 (C^3), 25.3 (C^4); **MS** (EI, 70 eV): *m/z* (%) = 189 (91), 161 (50), 146 (48), 132 (25), 120 (100), 92 (29); **HR-MS** (EI, 70 eV): *m/z* calculated for $[C_{11}H_{11}NO_2]^{\bullet+}$: 189.0784 ($[M]^{\bullet+}$); found: 189.0783, *m/z* calculated for $[C_{10}{}^{13}C_1H_{11}NO_2]^{\bullet+}$: 190.0818 ($[M]^{\bullet+}$); found: 190.0818; **IR**: $\tilde{\nu}$ = 3195 (w, NH), 3058 (w, sp^2-CH), 2971 (w, sp^3-CH), 2932 (w, sp^3-CH), 1661 (s, C=O), 1597 (m, C=C), 1480 (m), 1238 (m), 756 (s) cm^{-1}.

2.3.4 9-(Trifluoromethyl)-3,4,5,6-tetrahydro-1*H*-benzo[*b*]azonine-2,7-dione (6d)

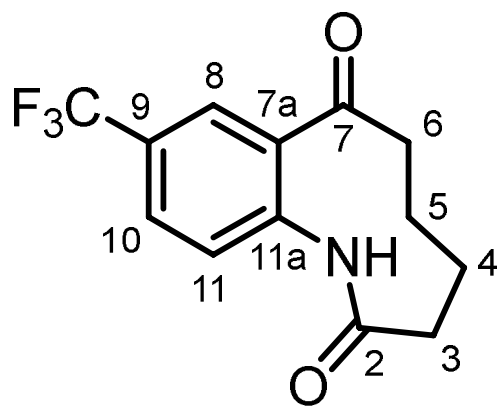

6d

According to general procedure **A**, tetrahydrocarbazole **SI-6** (12.0 mg, 50.0 µmol) was combined with urea hydrogen peroxide (18.8 mg, 200 µmol, 4.00 equiv.; instead of aqueous hydrogen peroxide) in EtOH (instead of MeOH) to prepare ketolactam **6d**.

Colorless solid (5.97 mg, 22.0 µmol, 44%); **^{1}H NMR** (500 MHz, $CDCl_3$, 298 K): δ (ppm) = 7.90 – 7.88 (m, 1H, H^8), 7.77 (dd, 1H, $^3J_{H\text{-}H}$ = 8.2 Hz, $^4J_{H\text{-}H}$ = 2.2 Hz, H^{10}), 7.72 (br s, 1H, N*H*), 7.35 (d, 1H, $^3J_{H\text{-}H}$ = 8.2 Hz, H^{11}), 2.90 – 2.88 (m, 2H, $H^{3/6}$), 2.28 – 2.26 (m, 2H, $H^{3/6}$), 2.07 – 2.00 (m, 2H, $H^{4/5}$), 1.99 – 1.93 (m, 2H, $H^{4/5}$); **^{13}C{^{1}H} NMR** (126 MHz, $CDCl_3$, 298 K): δ (ppm) = 204.5 (C^7), 176.0 (C^2), 138.5 ($C^{7a/11a}$), 137.9 ($C^{7a/11a}$), 130.1 (q, $^2J_{C\text{-}F}$ = 33.6 Hz, C^9), 129.1 (C^{10}), 128.3 (C^{11}), 126.3 (C^8), 123.5 (q, $^1J_{C\text{-}F}$ = 272.6 Hz, *C*F_3), 40.4 ($C^{3/6}$), 31.9 ($C^{3/6}$), 24.5 ($C^{4/5}$), 24.5 ($C^{4/5}$); **^{19}F NMR** (471 MHz, $CDCl_3$, 298 K): δ (ppm) = – 62.6; **MS** (EI, 70 eV): *m/z* (%) = 271 (5), 252 (8), 243 (20), 214 (24), 203 (48), 188 (100), 160 (17), 140 (8); **HR-MS** (EI, 70 eV): *m/z* calculated for $[C_{13}H_{12}F_3NO_2]^{\bullet+}$: 271.0815 ($[M]^{\bullet+}$); found: 271.0810, *m/z* calculated for $[C_{12}{}^{13}C_1H_{12}F_3NO_2]^{\bullet+}$: 272.0849 ($[M]^{\bullet+}$); found: 272.0853; **IR:** $\tilde{\nu}$ = 3349 (w, N*H*), 2945 (w, sp^3-CH), 2870 (w, sp^3-CH), 1679 (s, C=O), 1616 (w, C=C), 1492 (m, C=C), 1447 (m), 1322 (m), 1265 (m), 1241 (m), 1170 (m), 1134 (s), 1073 (m), 881 (w), 852 (m), 838 (m, sp^2-CH), 739 (w, sp^2-CH), 715 (m, sp^2-CH), 684 (m, sp^2-CH) cm^{-1}.

2.3.5 9-Chloro-3,4,5,6-tetrahydro-1*H*-benzo[*b*]azonine-2,7-dione (6e)

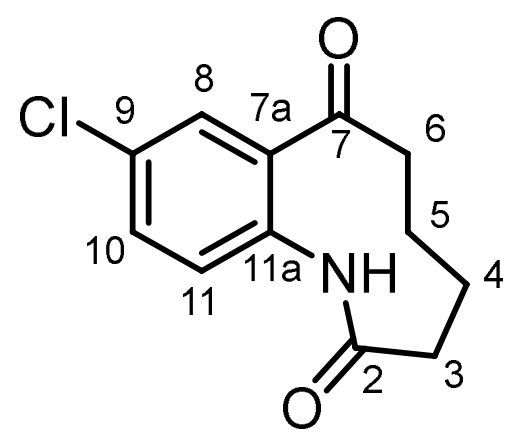

6e

According to general procedure **A**, tetrahydrocarbazole **SI-7** (10.3 mg, 50.0 µmol) was used to prepare ketolactam **6e**.

Colorless solid, (3.68 mg, 15.5 µmol, 31%); **^{1}H NMR** (500 MHz, $CDCl_3$, 298 K): δ (ppm) = 7.57 (s, 1H, H^8), 7.48 (dd, 1H, $^3J_{H\text{-}H}$ = 8.4 Hz, $^4J_{H\text{-}H}$ = 2.5 Hz, H^{10}), 7.18 (d, 1H, $^3J_{H\text{-}H}$ = 8.4 Hz, H^{11}), 7.15 (br s, 1H, N*H*), 2.91 – 2.89 (m, 2H, H^6), 2.25 – 2.23 (m, 2H, H^3), 1.91 – 1.89 (m, 4H, $H^{4,5}$); **^{13}C{^{1}H} NMR** (126 MHz, $CDCl_3$, 298 K): δ (ppm) = 204.6 (C^7), 175.9 (C^2), 140.7 ($C^{7a/11a}$), 134.4 (C^9), 132.6 ($C^{7a/11a}$), 132.1 (C^{10}), 130.0 (C^{11}), 128.6 (C^8), 41.3 (C^6), 32.3 (C^3), 24.9 ($C^{4/5}$), 24.5 ($C^{4/5}$); **MS** (EI, 70 eV): *m/z* (%) = 239 (5), 237 (16), 222 (5), 219 (12), 209 (10), 182 (5), 171 (20), 169 (70), 153 (100), 126 (16), 90 (12); **HR-MS** (EI, 70 eV): *m/z* calculated for $[C_{12}H_{12}O_2N_1{}^{35}Cl_1]^{\bullet+}$: 237.0551 ($[M]^{\bullet+}$); found: 237.0540, *m/z* calculated for $[C_{11}{}^{13}C_1H_{12}O_2N_1{}^{35}Cl_1]^{\bullet+}$: 238.0585 ($[M]^{\bullet+}$); found: 238.0576, *m/z* calculated for $[C_{12}H_{12}O_2N_1{}^{37}Cl_1]^{\bullet+}$: 239.0522 ($[M]^{\bullet+}$); found: 239.0518, *m/z* calculated for $[C_{11}{}^{13}C_1H_{12}O_2N_1{}^{37}Cl_1]^{\bullet+}$: 240.0555 ($[M]^{\bullet+}$); found: 240.0556; **IR:** $\tilde{\nu}$ = 3197 (w, OH), 3148 (w, sp^2-CH), 2998 (w, sp^3-CH), 2932 (w, sp^3-CH), 2862 (w, sp^3-CH), 1692 (m, C=O), 1641 (m, C=C), 1598 (w, C=C), 1518 (m, C=C), 1477 (m), 1399 (w), 1293 (w), 1228 (m), 1182 (m), 1139 (m), 1089 (w), 1061 (w), 999 (w), 936 (w), 825 (m), 731 (w, sp^2-CH), 684 (m, sp^2-CH) cm^{-1}.

2.3.6 9-Methoxy-3,4,5,6-tetrahydro-1*H*-benzo[*b*]azonine-2,7-dione (6f)

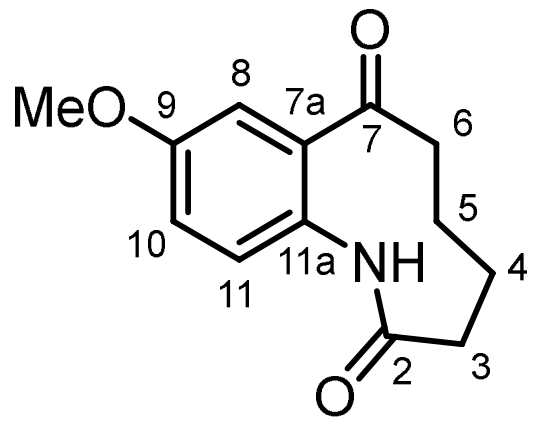

6f

According to general procedure **A**, tetrahydrocarbazole **SI-8** (10.0 mg, 50.0 µmol) was used to prepare ketolactam **6f**. The reaction was performed in EtOH (instead of MeOH). The analytical data of compound **6f** are consistent with literature values.[21]

Colorless solid (4.20 mg, 18.0 µmol, 36%); **^{1}H NMR** (500 MHz, $CDCl_3$, 298 K): δ (ppm) = 7.15 (d, 1H, $^{3}J_{H\text{-}H}$ = 8.6 Hz, H^{11}), 7.13 (br s, 1H, N*H*), 7.06 (m, 1H, H^{8}), 7.02 (dd, 1H, $^{3}J_{H\text{-}H}$ = 8.6 Hz, $^{4}J_{H\text{-}H}$ = 3.0 Hz, H^{10}), 3.85 (m, 3H, OC*H*$_3$), 2.91 (m, 2H, H^{6}), 2.25 (m, 2H, H^{4}), 1.84 (m, 4H, $H^{3,5}$). **^{13}C{^{1}H} NMR** (126 MHz, $CDCl_3$, 298 K): δ (ppm) = 206.0 (C^{7}), 176.9 (C^{2}), 159.5 (C^{9}), 141.0 (C^{7a}), 130.7 (C^{11}), 126.6 (C^{11a}), 118.2 (C^{10}), 113.0 (C^{8}), 55.9 (O*C*H$_3$), 42.0 (C^{6}), 32.7 (C^{4}), 25.6 (C^{3}), 24.6 (C^{5}); **IR:** $\tilde{\nu}$ = 3387 (m, OH), 3067 (w, sp^2-CH), 3004 (w, sp^2-CH), 2951 (m, sp^3-CH), 2915 (m, sp^3-CH), 2850 (m, sp^3-CH), 2051 (w), 1836 (w), 1716 (w, C=O), 1623 (m, C=C), 1590 (m, C=C), 1484 (s, C=C), 1468 (m), 1452 (s), 1430 (s), 1370 (w), 1322 (m), 1256 (m), 1217 (s), 1178 (m), 1136 (s, O–C), 1112 (m), 1029 (s, O–C), 1001 (w), 967 (m), 918 (m), 864 (w), 830 (s), 799 (s, sp^2-CH), 742 (w), 699 (m), 672 (w) cm^{-1}.

2.3.7 3-Methyl-3,4,5,6-tetrahydro-1*H*-benzo[*b*]azonine-2,7-dione (6g)

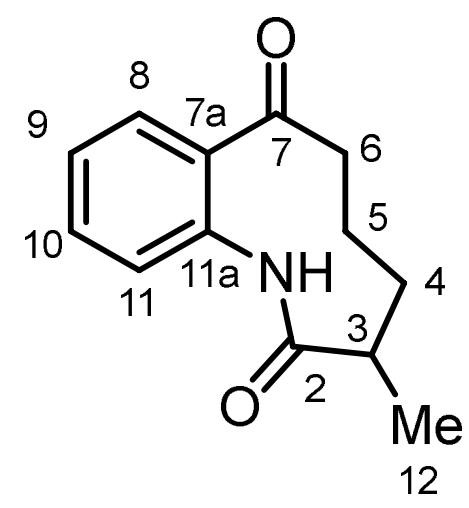

6g

According to general procedure **A**, tetrahydrocarbazole **SI-4** (9.26 mg, 50.0 µmol) was used to prepare ketolactam **6g**.

Colorless solid (2.50 mg, 11.5 µmol, 23%); **^{1}H NMR** (500 MHz, $CDCl_3$, 298 K): δ (ppm) = 7.58 (d, 1H, $^3J_{H\text{-}H}$ = 7.6 Hz, $H^{8/11}$), 7.52 (*virt.* t, 1H, $^3J_{H\text{-}H} \approx {}^3J_{H\text{-}H}$ = 7.6 Hz, $H^{9/10}$), 7.43 (*virt.* t, 1H, $^3J_{H\text{-}H} \approx {}^3J_{H\text{-}H}$ = 7.6 Hz, $H^{9/10}$), 7.21 (d, 1H, $^3J_{H\text{-}H}$ = 7.6 Hz, $H^{8/11}$), 7.11 (br s, 1H, N*H*), 3.01 – 2.97 (m, 1H, H^6), 2.82 – 2.74 (m, 1H, H^6), 2.54 – 2.45 (s, 1H, H^3), 2.02 – 1.96 (m, 1H, H^5), 1.81 – 1.72 (m, 2H, $H^{4,5}$), 1.28 – 1.18 (m, 1H, H^4), 1.09 (d, 3H, $^3J_{H\text{-}H}$ = 6.6 Hz, H^{12}); **^{13}C{^{1}H} NMR** (126 MHz, $CDCl_3$, 298 K): δ (ppm) = 206.5 (C^7), 179.1 (C^2), 139.8 (C^{11a}), 133.7 (C^{7a}), 132.3 ($C^{9/10}$), 128.8 ($C^{8/11}$), 128.8 ($C^{8/11}$), 128.6 ($C^{9/10}$), 41.4 (C^6), 35.9 (C^3), 34.3 (C^4), 23.8 (C^5), 19.3 (C^{12}); **MS** (EI, 70 eV): *m/z* (%) = 217 (10), 189 (45), 174 (19), 179 (8), 156 (15), 146 (23), 135 (30), 120 (100), 106 (4), 92 (22); **HR-MS** (EI, 70 eV): *m/z* calculated for $[C_{13}H_{15}O_2N_1]^{\bullet+}$: 217.1097 ($[M]^{\bullet+}$); found: 217.1098, *m/z* calculated for $[C_{12}{}^{13}C_1H_{15}O_2N_1]^{\bullet+}$: 218.1131 ($[M]^{\bullet+}$); found: 218.1135; **IR:** $\tilde{\nu}$ = 3220 (w, OH), 2971 (w, sp^3-CH), 2932 (w, sp^3-CH), 2861 (w, sp^3-CH), 1680 (m, C=O), 1644 (m, C=O), 1602 (m, C=C), 1516 (m, C=C), 1482 (m), 1443 (m), 1377 (w), 1344 (w), 1272 (w), 1225 (w), 1177 (m), 1156 (w), 1107 (w), 1066 (w), 1048 (w), 1024 (w), 983 (w), 953 (w), 900 (w), 868 (w), 848 (w), 792 (w, sp^2-CH), 749 (s, sp^2-CH), 714 (w, sp^2-CH), 668 (m).

General procedure B (isoalloxazinium salt catalyzed preparation of hydroxyindolenines)

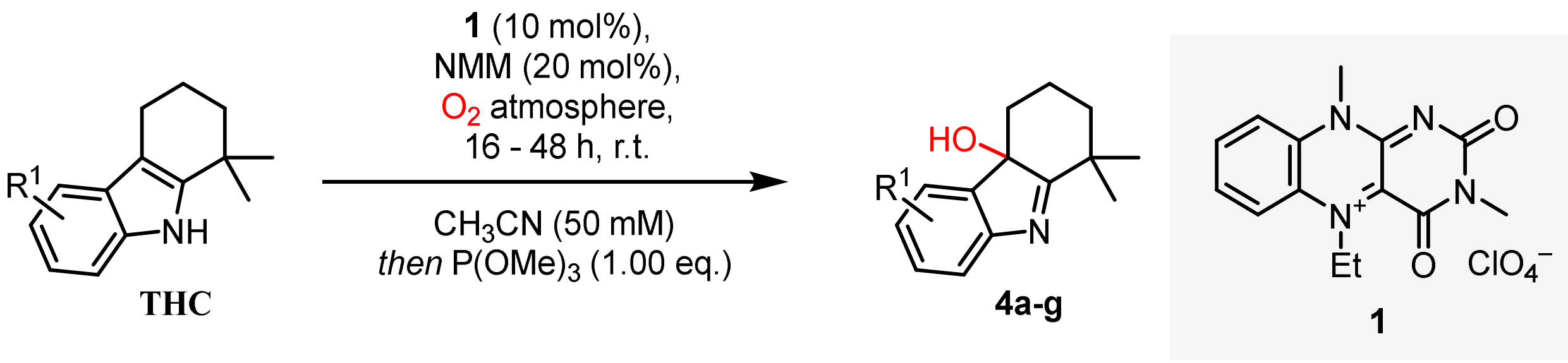

Catalyst **1** (1.85 mg, 5.00 µmol, 0.10 equiv.) and *N*-methylmorpholine (NMM) (1.11 µL, 10.0 µmol, 0.20 equiv.) in 1 mL of CH_3CN was added to the respective catalysis substrate **THC** (50.0 µmol, 1.00 equiv.) in a crimp vial. The vial was capped and purged with oxygen by bubbling through the solution for approximately 8 seconds. After stirring for 24 h at room temperature the vial was uncapped and 5.91 µL (50.0 µmol, 1.00 equiv.) of trimethyl phosphite were added. The reaction mixture was stirred open to air at room temperature for an additional 30 minutes. The solvent was removed *in vacuo* and the crude residue was dissolved in a minimal amount of acetone and loaded on a *Biotage* C18 samplet. Purification by reverse-phase flash chromatography (CH_3CN/H_2O = 1/9 → 100% CH_3CN) and removal of the solvents by lyophilization using a freeze dryer afforded hydroxyindolenines **4a-g**.

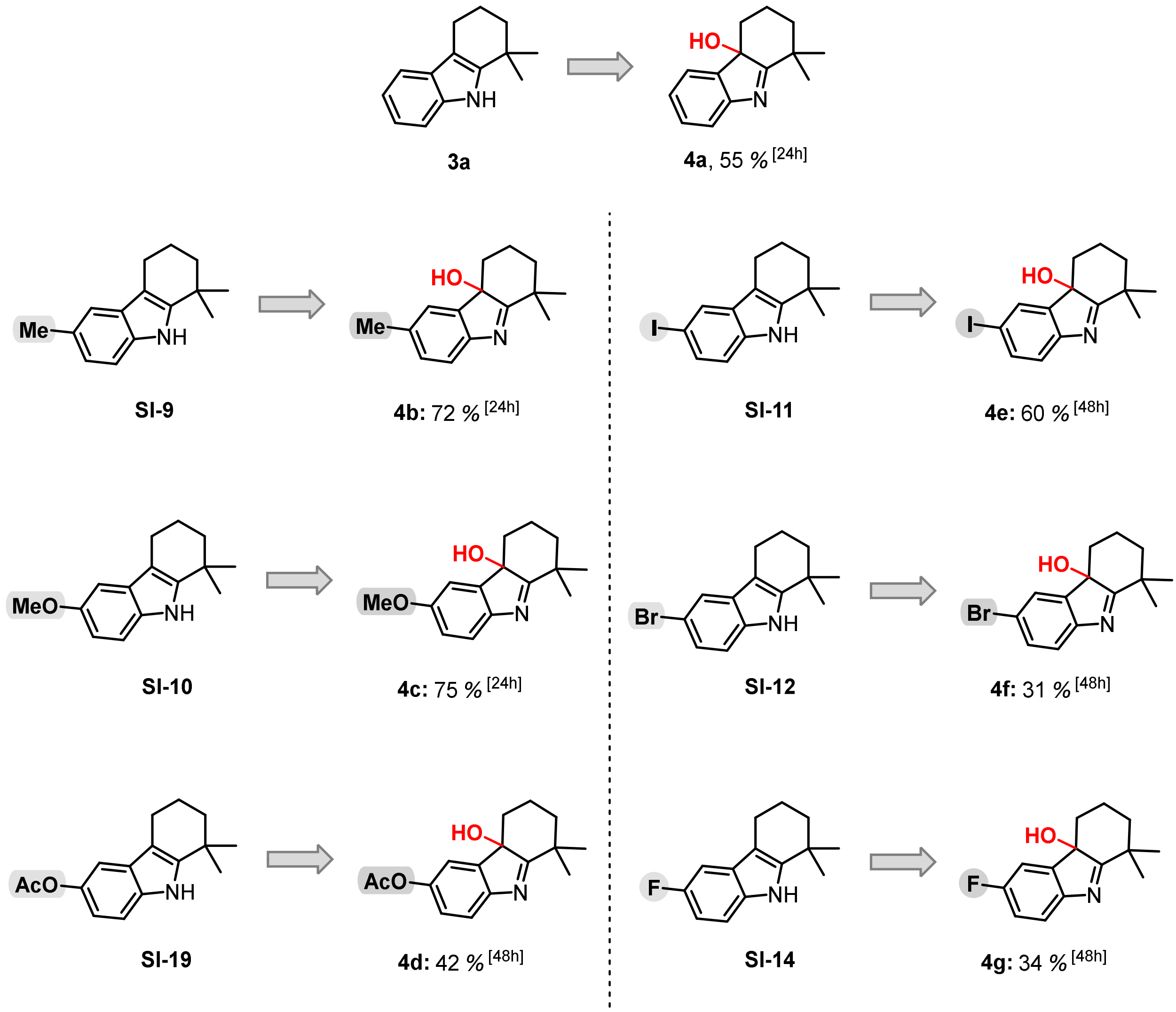

Figure SI-15. Substrate – catalysis product overview of isoalloxazinium salt catalyzed preparation of hydroxyindolenines. All yields refer to isolated ones.

2.3.8 1,1-Dimethyl-1,2,3,4-tetrahydro-4a*H*-carbazol-4a-ol (4a)

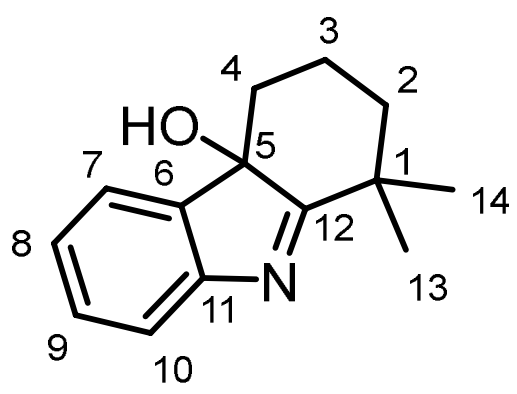

4a

According to general procedure **B**, tetrahydrocarbazole **3a** (10.0 mg, 50.0 µmol) was used to prepare hydroxyindolenine **4a**.

White solid, (5.92 mg, 27.5 µmol, 55%); **TLC** (CH_2Cl_2/Et_2O, 95/5): $R_f = 0.26$ [UV]; **^{1}H NMR** (500 MHz, $CDCl_3$, 298 K): δ (ppm) = 7.57 – 7.54 (m, 1H, H^{10}), 7.38 – 7.32 (m, 2H, $H^{7,8}$), 7.20 (*virt.* td, $^3J_{H\text{-}H} \approx {}^3J_{H\text{-}H} = 7.4$ Hz, $^4J_{H\text{-}H} = 1.0$ Hz, 1H, H^9), 2.55 – 2.50 (m, 1H, H^4), 2.27 – 2.17 (m, 1H, H^3), 1.88 – 1.83 (m, 2H, H^2, O*H*), 1.65 – 1.59 (m, 1H, H^3), 1.47 (s, 3H, $H^{13/14}$), 1.41 (s, 3H, $H^{13/14}$), 1.42 – 1.35 (m, 1H, H^2), 1.27 – 1.21 (m, 1H, H^4); **^{13}C{^{1}H} NMR** (126 MHz, $CDCl_3$, 298 K): δ (ppm) = 190.9 (C^{12}), 152.7 (C^{11}), 141.8 (C^6), 129.7 ($C^{7/8}$), 126.0 (C^9), 121.9 ($C^{7/8}$), 120.9 (C^{10}), 83.5 (C^5), 43.5 (C^2), 39.2 (C^4), 39.1 (C^1), 28.6 ($C^{13/14}$), 23.6 ($C^{13/14}$), 18.4 (C^3); **HR-MS** (ESI$^+$): *m/z* calculated for $[C_{14}H_{17}NO+H]^+$: 216.1383 ($[M+H]^+$); found: 216.1399; **IR:** $\tilde{\nu}$ = 3215 (w, OH), 2985 (w, sp^3-CH), 2958 (w, sp^3-CH), 2925 (m, sp^3-CH), 2862 (w, sp^3-CH), 1568 (m, C=C), 1459 (m), 1383 (w), 1365 (w), 1316 (w), 1273 (w), 1234 (w), 1211 (w), 1187 (m), 1156 (w), 1115 (w), 1098 (w, O–C), 1074 (s), 1034 (m), 1013 (w), 992 (w), 970 (m), 930 (m), 890 (m), 877 (w), 868 (w), 855 (w), 772 (s), 759 (s), 718 (m, sp^2-CH), 703 (m, sp^2-CH), 682 (m, sp^2-CH) cm^{-1}.

2.3.9 1,1,6-Trimethyl-1,2,3,4-tetrahydro-4a*H*-carbazol-4a-ol (4b)

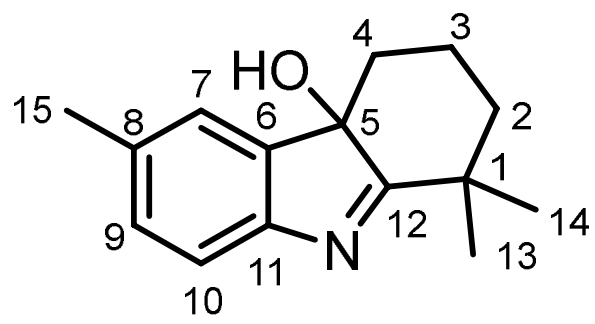

4b

According to general procedure **B**, tetrahydrocarbazole **SI-9** (10.7 mg, 50.0 µmol) was used to prepare hydroxyindolenine **4b**.

Pale-yellow solid, (8.30 mg, 36.0 µmol, 72%); **TLC:** R_f = 0.42 (*n*-pentane/EtOAc, 8/2) [UV]; **^{1}H NMR** (500 MHz, $CDCl_3$, 298 K): δ (ppm) = 7.42 (d, $^3J_{H-H}$ = 7.8 Hz, 1H, H^{10}), 7.15 (d, $^4J_{H-H}$ = 0.9 Hz, 1H, H^7), 7.13 (dd, $^3J_{H-H}$ = 7.8 Hz, $^4J_{H-H}$ = 0.9 Hz, 1H, H^9), 2.52 – 2.45 (m, 1H, H^4), 2.36 (s, 3H, H^{15}), 2.27 – 2.13 (m, 1H, H^3), 1.95 (br s, 1H, O*H*), 1.87 – 1.80 (m, 1H, H^2), 1.65 –1.55 (m, 1H, H^3), 1.46 (s, 3H, $H^{13/14}$), 1.41 – 1.32 (m, 1H, H^2), 1.38 (s, 3H, $H^{13/14}$), 1.27 – 1.17 (m, 1H, H^4); **^{13}C{^{1}H} NMR** (126 MHz, $CDCl_3$, 298 K): δ (ppm) = 190.0 (C^{12}), 150.3 (C^{11}), 142.0 (C^6), 135.9 (C^8), 130.0 (C^9), 122.8 (C^7), 120.4 (C^{10}), 83.4 (C^5), 43.6 (C^2), 39.2 (C^4), 39.0 (C^1), 28.6 ($C^{13/14}$), 23.6 ($C^{13/14}$), 21.5 (C^{15}), 18.5 (C^3); **HR-MS** (ESI^+): *m/z* calculated for $[C_{15}H_{19}NO+H]^+$: 230.1539 ($[M+H]^+$); found: 230.1540.

2.3.10 6-Methoxy-1,1-dimethyl-1,2,3,4-tetrahydro-4a*H*-carbazol-4a-ol (4c)

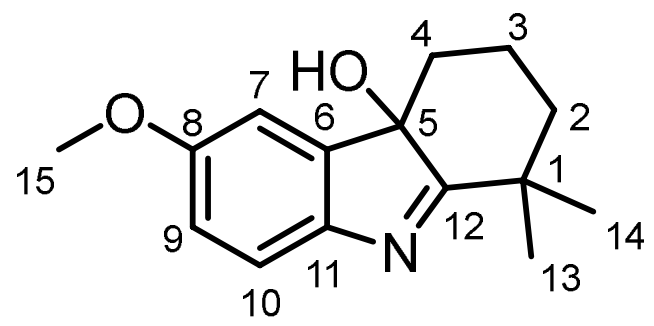

4c

According to general procedure **B**, tetrahydrocarbazole **SI-10** (11.5 mg, 50.0 µmol) was used to prepare hydroxyindolenine **4c**.

White solid, (9.20 mg, 37.5 µmol, 75%); **TLC:** R_f = 0.24 (*n*-pentane/EtOAc, 8/2) [UV]; **^{1}H NMR** (500 MHz, $CDCl_3$, 298 K): δ (ppm) = 7.44 (d, $^3J_{H\text{-}H}$ = 8.4 Hz, 1H, H^{10}), 6.91 (d, $^4J_{H\text{-}H}$ = 2.6 Hz, 1H, H^7), 6.82 (dd, $^3J_{H\text{-}H}$ = 8.4 Hz, $^4J_{H\text{-}H}$ = 2.6 Hz, 1H, H^9), 3.80 (s, 3H, H^{15}), 2.49 – 2.42 (m, 1H, H^4), 2.26 – 2.13 (m, 1H, H^3), 2.08 (br s, 1H, O*H*), 1.86 – 1.79 (m, 1H, H^2), 1.65 – 1.56 (m, 1H, H^3), 1.45 (s, 3H, $H^{13/14}$), 1.40 – 1.33 (m, 1H, H^2), 1.37 (s, 3H, $H^{13/14}$), 1.27 – 1.17 (m, 1H, H^4); **^{13}C{^{1}H} NMR** (126 MHz, $CDCl_3$, 298 K): δ (ppm) = 189.0 (C^{12}), 158.5 (C^8), 146.0 (C^{11}), 143.4 (C^6), 121.2 (C^{10}), 113.9 (C^9), 108.7 (C^7), 83.6 (C^5), 55.9 (C^{15}), 43.6 (C^2), 39.3 (C^4), 38.9 (C^1), 28.6 ($C^{13/14}$), 23.7 ($C^{13/14}$), 18.5 (C^3); **HR-MS** (ESI$^+$): *m/z* calculated for $[C_{15}H_{19}NO_2+H]^+$: 246.1489 ($[M+H]^+$); found: 246.1489; **IR:** $\tilde{\nu}$ = 3205 (br, OH), 2929 (w, sp^3-CH), 1616 (w, C=C), 1575 (m, C=C), 1476 (s, C=C), 1431 (w, sp^3-CH), 1382 (w), 1288 (s, C-O-C), 1245 (w), 1218 (m), 1195 (m), 1176 (m), 1148 (w), 1094 (m), 1075 (s), 1031 (s), 1017 (m), 975 (m), 920 (w), 860 (m), 849, 825 (vs), 791 (m), 702 (w) cm^{-1}.

2.3.11 4a-Hydroxy-1,1-dimethyl-2,3,4,4a-tetrahydro-1*H*-carbazol-6-yl acetate (4d)

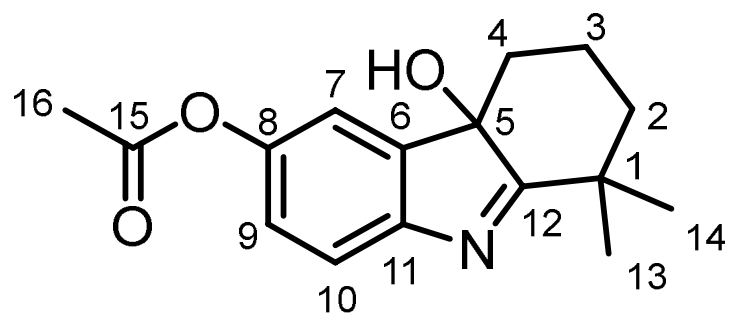

4d

According to general procedure **B**, tetrahydrocarbazole **SI-19** (12.9 mg, 50.0 µmol) was used to prepare hydroxyindolenine **4d**. The reaction time was prolongued to 48 h.

Pale yellow solid (5.70 mg, 20.9 µmol, 42%); **TLC:** R_f = 0.19 (*n*-pentane/EtOAc, 8/2) [UV]; **^{1}H NMR** (500 MHz, $CDCl_3$, 298 K): δ (ppm) = 7.53 (d, $^3J_{H-H}$ = 8.3 Hz, 1H, H^{10}), 7.07 (d, $^4J_{H-H}$ = 2.3 Hz, 1H, H^7), 7.02 (dd, $^3J_{H-H}$ = 8.3 Hz, $^4J_{H-H}$ = 2.3 Hz, 1H, H^9), 2.51 – 2.43 (m, 1H, H^4), 2.28 (s, 3H, H^{16}), 2.28 – 2.14 (m, 1H, H^3), 2.00 (br s, 1H, O*H*), 1.88 – 1.80 (m, 1H, H^2), 1.66 – 1.54 (m, 1H, H^3), 1.44 (s, 3H, $H^{13/14}$), 1.41 – 1.33 (m, 1H, H^2), 1.37 (s, 3H, $H^{13/14}$), 1.28 – 1.19 (m, 1H, H^4); **^{13}C{^{1}H} NMR** (126 MHz, $CDCl_3$, 298 K): δ (ppm) = 191.7 (C^{12}), 169.9 (C^{15}), 149.7 (C^{11}), 149.0 (C^8), 143.2 (C^6), 122.5 (C^9), 121.2 (C^{10}), 116.1 (C^7), 83.7 (C^5), 43.6 (C^2), 39.4 (C^4), 39.2 (C^1), 28.5 ($C^{13/14}$), 23.5 ($C^{13/14}$), 21.2 (C^{16}), 18.4 (C^3); **HR-MS** (ESI^+): *m/z* calculated for $[C_{16}H_{19}NO_3+H]^+$: 274.1438 ($[M+H]^+$); found: 274.1437; **IR:** $\tilde{\nu}$ = 3200 (br, OH), 2942 (w, sp^3-CH), 1754 (s, C=O), 1571 (w, C=C), 1467 (m, C=C), 1428 (w, sp^3-CH), 1372 (m), 1266 (w), 1212 (vs, C-O-C), 1184 (s), 1170 (vs), 1097 (w), 1074 (m), 1036 (m), 1014 (m), 973 (w), 935 (w), 904 (s), 883 (w), 874 (w), 842 (s), 799 (w), 704 (w), 655 (m) cm^{-1}.

2.3.12 6-Iodo-1,1-dimethyl-1,2,3,4-tetrahydro-4a*H*-carbazol-4a-ol (4e)

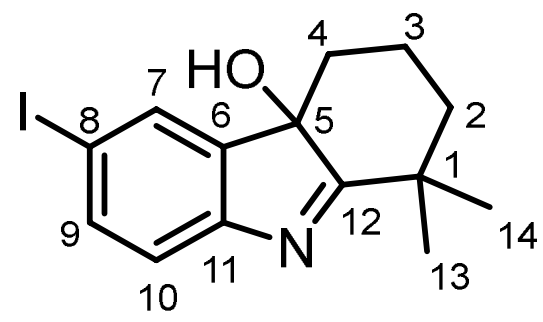

4e

According to general procedure **B**, tetrahydrocarbazole **SI-11** (16.3 mg, 50.0 µmol) was used to prepare hydroxyindolenine **4e**. The reaction time was prolongued to 48 h.

White solid (10.3 mg, 30.0 µmol, 60%); **TLC:** R_f = 0.53 (*n*-pentane/EtOAc, 8/2) [UV]; **^{1}H NMR** (500 MHz, $CDCl_3$, 298 K): δ (ppm) = 7.68 – 7.64 (m, 2H, $H^{7,10}$), 7.31 – 7.27 (m, 1H, H^9), 2.51 – 2.44 (m, 1H, H^4), 2.26 – 2.12 (m, 1H, H^3), 2.03 (br s, 1H, O*H*), 1.88 – 1.81 (m, 1H, H^2), 1.66 – 1.57 (m, 1H, H^3), 1.45 (s, 3H, $H^{13/14}$), 1.41 – 1.33 (m, 1H, H^2), 1.37 (s, 3H, $H^{13/14}$), 1.26 – 1.16 (m, 1H, H^4); **^{13}C{^{1}H} NMR** (126 MHz, $CDCl_3$, 298 K): δ (ppm) = 191.2 (C^{12}), 152.2 (C^{11}), 144.3 (C^6), 138.6 (C^7/C^{10}), 131.3 (C^7/C^{10}), 122.8 (C^9), 90.7 (C^8), 83.7 (C^5), 43.5 (C^2), 39.3 (C^4), 39.2 (C^1), 28.5 ($C^{13/14}$), 23.5 ($C^{13/14}$), 18.3 (C^3); **HR-MS** (ESI^+): *m/z* calculated for $[C_{14}H_{16}INO+H]^+$: 342.0349 ($[M+H]^+$); found: 342.0349; **IR:** $\tilde{\nu}$ = 3216 (br, OH), 2925 (w, sp^3-CH), 1569 (m, C=C), 1449 (m, C=C), 1414 (w, sp^3-CH), 1383 (m), 1315 (w), 1279 (w), 1257 (w), 1207 (w), 1187 (m), 1111 (m), 1074 (m), 1033 (m), 1017 (w), 971 (m), 887 (w), 822 (vs), 684 (m) cm^{-1}.

2.3.13 6-Bromo-1,1-dimethyl-1,2,3,4-tetrahydro-4a*H*-carbazol-4a-ol (4f)

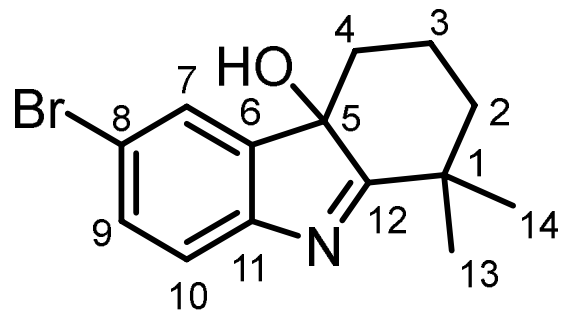

4f

According to general procedure **B**, tetrahydrocarbazole **SI-12** (13.9 mg, 50.0 µmol) was used to prepare hydroxyindolenine **4f**. The reaction time was prolongued to 48 h.

White solid (4.56 mg, 15.5 µmol, 31%); **TLC:** R_f = 0.50 (*n*-pentane/EtOAc, 8/2) [UV]; **^{1}H NMR** (500 MHz, $CDCl_3$, 298 K): δ (ppm) = 7.48 – 7.44 (d, 1H, $H^{7,9}$), 7.42 – 7.39 (m, 1H, H^{10}), 2.52 – 2.47 (m, 1H, H^4), 2.25 – 2.15 (m, 1H, H^3), 2.17 (s, 1H, O*H*), 1.88 – 1.82 (m, 1H, H^2), 1.66 – 1.58 (m, 1H, H^3), 1.47 (s, 3H, $H^{13/14}$), 1.42 – 1.34 (m, 1H, H^2), 1.38 (s, 3H, $H^{13/14}$), 1.25 – 1.18 (m, 1H, H^4); **^{13}C{^{1}H} NMR** (126 MHz, $CDCl_3$, 298 K): δ (ppm) = 191.5 (C^{12}), 151.7 (C^{11})*, 143.9 (C^6), 132.6 ($C^{7/9}$), 125.5 ($C^{7/9}$), 122.2 (C^{10}), 119.8 (C^8), 83.8 (C^5), 43.5 (C^2), 39.27 (C^4), 39.24 (C^1), 28.5 ($C^{13/14}$), 23.5 ($C^{13/14}$), 18.3 (C^3); **HR-MS** (ESI^+): *m/z* calculated for $[C_{14}H_{16}{}^{79}BrNO+H]^+$: 294.0488 ($[M+H]^+$); found: 294.0487; *m/z* calculated for $[C_{14}H_{16}{}^{81}BrNO+H]^+$: 296.0468 ($[M+H]^+$); found: 296.0467; **IR:** $\tilde{\nu}$ = 3241 (br, OH), 2944 (w, sp^3-CH), 2924 (w, sp^3-CH), 1571 (m, C=C), 1453 (m, C=C), 1418 (w, sp^3-CH), 1386 (w), 1363 (w), 1319 (w), 1280 (w), 1258 (w), 1207 (w), 1185 (m), 1120 (w), 1074 (m), 1052 (w), 1034 (w), 1013 (w), 972 (w), 889 (w), 872 (w), 824 (vs), 689 (m) cm^{-1}.

* low intensity in ^{13}C, assigned with 2D

2.3.14 6-Fluoro-1,1-dimethyl-1,2,3,4-tetrahydro-4a*H*-carbazol-4a-ol (4g)

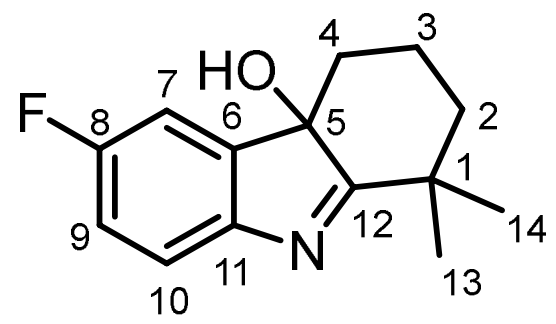

4g

According to general procedure **B**, tetrahydrocarbazole **SI-14** (10.9 mg, 50.0 µmol) was used to prepare hydroxyindolenine **4g**. The reaction time was prolongued to 48 h.

White solid (3.97 mg, 17.0 µmol, 34%); **TLC:** R_f = 0.39 (*n*-pentane/EtOAc, 8/2) [UV]; **^{1}H NMR** (500 MHz, $CDCl_3$, 298 K): δ (ppm) = 7.48 (dd, $^3J_{H-H}$ = 8.4 Hz, $^4J_{H-F}$ = 4.5 Hz, 1H, H^{10}), 7.07 (dd, $^3J_{H-F}$ = 7.5 Hz, $^4J_{H-H}$ = 2.6 Hz, 1H, H^7), 7.01 (ddd, $^3J_{H-F}$ = 9.2 Hz, $^3J_{H-H}$ = 8.4 Hz, $^4J_{H-H}$ = 2.6 Hz, 1H, H^9), 2.52 – 2.45 (m, 1H, H^4), 2.28 – 2.14 (m, 1H, H^3), 1.97 (br s, 1H, O*H*), 1.89 – 1.81 (m, 1H, H^2), 1.66 – 1.58 (m, 1H, H^3), 1.47 (s, 3H, $H^{13/14}$), 1.43 – 1.34 (m, 1H, H^2), 1.39 (s, 3H, $H^{13/14}$), 1.28 – 1.18 (m, 1H, H^4); **^{13}C{^{1}H} NMR** (126 MHz, $CDCl_3$, 298 K): δ (ppm) = 190.8 (C^{12}), 161.6 (d, $^1J_{C-F}$ = 245.4 Hz, C^8), 148.4 (C^{11}), 143.8 (d, $^3J_{C-F}$ = 8.3 Hz, C^6), 121.6 (d, $^3J_{C-F}$ = 8.5 Hz C^{10}), 115.9 (d, $^2J_{C-F}$ = 23.5 Hz, C^9), 109.9 (d, $^2J_{C-F}$ = 24.7 Hz , C^7), 83.7 (C^5), 43.6 (C^2), 39.2 (C^4), 39.1 (C^1), 28.5 ($C^{13/14}$), 23.6 ($C^{13/14}$), 18.4 (C^3); **HR-MS** (ESI$^+$): *m/z* calculated for $[C_{14}H_{16}FNO+H]^+$: 234.1289 ($[M+H]^+$); found: 234.1288; **IR:** $\tilde{\nu}$ = 3240 (br, OH), 2928 (w, sp^3-CH), 1571 (m, C=C), 1469 (s, C=C), 1427 (m, sp^3-CH), 1384 (m), 1362 (w), 1325 (w), 1255 (w), 1211 (w), 1166 (m), 1144 (m), 1114 (m), 1102 (m), 1073 (m), 1037 (m), 1012 (w), 974 (w), 923 (w), 878 (m), 852 (w), 828 (vs), 793 (vs), 761 (w), 699 (m) cm^{-1}.

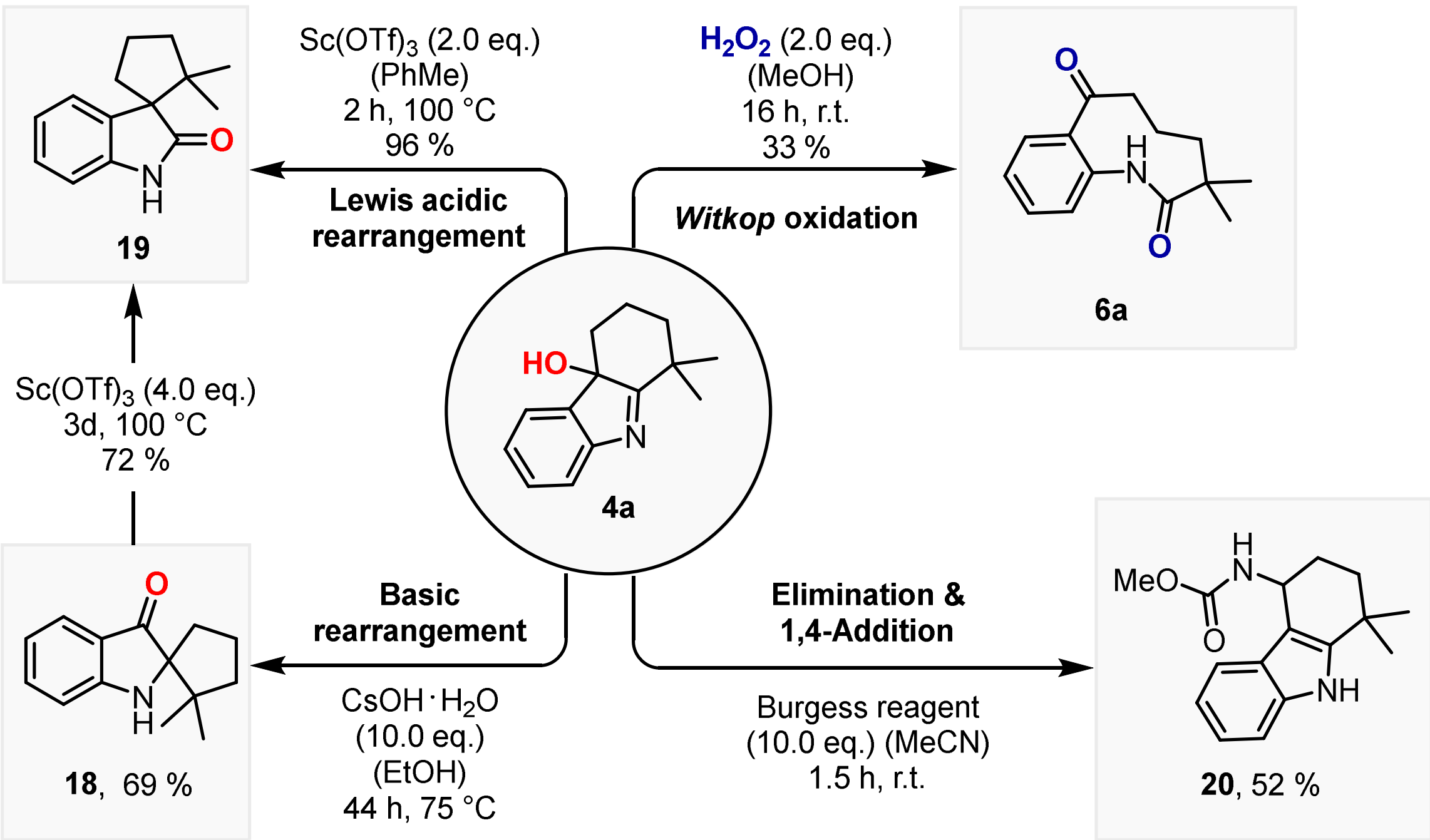

Figure SI-16. Follow-up chemistry of **4a**. All yields refer to isolated ones.

2.3.16 2,2-Dimethylspiro[cyclopentane-1,2'-indolin]-3'-one (18)

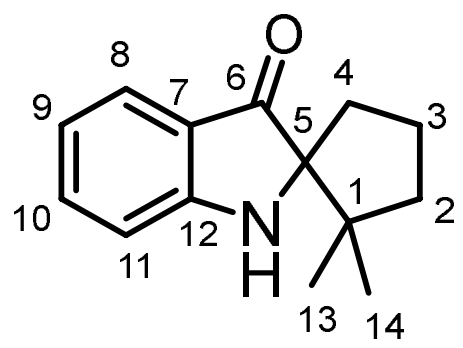

18

Method A – Oxidation of tetrahydrocarbazole **3a** *with* MnO_2: A round-bottom flask was filled with tetrahydrocarbazole **3a** (108 mg, 542 µmol, 1.00 equiv.) and ethyl acetate (70 mL). To this solution, MnO_2 (2.83 g, 32.5 mmol, 60.0 equiv.) was added and the mixture was stirred at room temperature for 4 h. The reaction mixture was then filtered over silica under reduced pressure and purified using flash chromatography (silica, P/EtOAc = 9/1) to yield compound **18** (52.6 mg, 244 µmol, 45%) as a yellow solid.

Method B – Basic rearrangement of hydroxyindolenine **4a** *with NaOMe*: **4a** (21.0 mg, 97.5 µmol, 1.00 equiv.) and NaOMe (52.7 mg, 975 µmol, 10.0 equiv.) were dissolved in ethanol (3 mL). The reaction mixture was stirred at 75 °C for 44 h. Water (20 mL) was added and the phases were allowed to separate. The aqueous phase was extracted with DCM (3 × 15 mL) and the combined organic layers were washed with water (2 × 15 mL) and a saturated aqueous NaCl solution (15 mL), dried over Na_2SO_4, filtered and concentrated *in vacuo* to afford **18** (12.6 mg, 58.5 µmol, 60%) as a yellow solid.

Method C – Basic rearrangement of hydroxyindolenine **4a** *with CsOH · H_2O*: **4a** (26.0 mg, 121 µmol, 1.00 equiv.) and CsOH · H_2O (203 mg, 1.21 mmol, 10.0 equiv.) were dissolved in ethanol (3 mL). The reaction mixture was stirred at 75 °C for 44 h. Water (20 mL) was added and the phases were allowed to separate. The aqueous phase was extracted with DCM (3 × 15 mL) and the combined organic layers were washed with water (2 × 15 mL) and a saturated aqueous NaCl solution (15 mL), dried over Na_2SO_4, filtered and concentrated *in vacuo* to afford **18** (18.0 mg, 83.6 µmol, 69%) as a yellow solid.

The analytical data of compound **18** are consistent with literature values.[22]

Yellow solid; **TLC:** R_f = 0.44 (*n*-pentane/EtOAc, 9/1) [UV]; **^{1}H NMR** (500 MHz, $CDCl_3$, 298 K): δ (ppm) = 7.54 (dd, $^3J_{H\text{-}H}$ = 7.8 Hz, $^4J_{H\text{-}H}$ = 1.2 Hz, 1H, H^8), 7.39 (ddd, $^3J_{H\text{-}H}$ = 8.3, 7.1 Hz, $^4J_{H\text{-}H}$ = 1.2 Hz, 1H, H^{10}), 6.81 (dd, $^3J_{H\text{-}H}$ = 8.3 Hz, $^4J_{H\text{-}H}$ = 0.8 Hz, 1H, H^{11}), 6.76 (ddd, $^3J_{H\text{-}H}$ = 7.8, 7.1 Hz, $^4J_{H\text{-}H}$ = 0.8 Hz, 1H, H^9), 4.48 (br s, 1H, N*H*), 2.23 – 2.15 (m, 1H, H^2), 2.12 – 2.05 (m, 1H, H^3), 2.02 – 1.92 (m, 1H, H^4), 1.91 – 1.79 (m, 2H, $H^{3,4}$), 1.61 – 1.57 (m, 1H, H^2), 1.02 (s, 3H, $H^{13/14}$), 0.89 (s, 3H, $H^{13/14}$); **^{13}C{^{1}H} NMR** (126 MHz, $CDCl_3$, 298 K): δ (ppm) = 205.3 (C^6), 160.3 (C^{12}), 136.8 (C^{10}), 124.5 (C^8), 121.2 (C^7), 118.4 (C^9), 111.7 (C^{11}), 78.0 (C^5), 45.5 (C^1), 37.8 (C^2), 33.4 (C^3), 25.1 ($C^{13/14}$), 22.5 ($C^{13/14}$), 19.3 (C^4); **MS** (EI, 70 eV): *m/z* (%) = 215 (48), 200 (3), 186 (16), 159 (17), 146 (100), 130 (18), 117 (11), 91 (8); **HR-MS** (EI, 70 eV): *m/z* calculated for $[C_{14}H_{17}O_1N_1]^{\bullet+}$: 215.1305 ($[M]^{\bullet+}$); found: 215.1305, *m/z* calculated for $[C_{13}{}^{13}C_1 H_{17}O_1N_1]^{\bullet+}$: 216.1338 ($[M]^{\bullet+}$); found: 218.1137; **IR:** $\tilde{\nu}$ = 3353 (w, NH), 2955 (m, sp^3-CH), 2928 (m, sp^3-CH), 2867 (w, sp^3-CH), 1674 (w, C=O), 1619 (m, C=C), 1490 (w, C=C), 1469 (m), 1382 (w), 1364 (w), 1324 (m), 1306 (w), 1243 (w), 1194 (w), 1153 (w), 1101 (w), 1075 (w), 1005 (w), 948 (w), 923 (w), 897 (w), 814 (w, sp^2-CH), 750 (m, sp^2-CH), 721 (w, sp^2-CH) cm^{-1}.

2.3.17 2,2-Dimethylspiro[cyclopentane-1,3'-indolin]-2'-one (19)

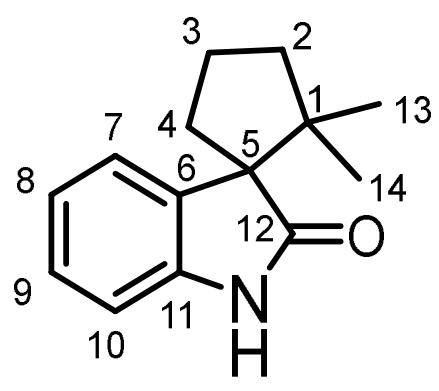

19

Rearrangement starting from hydroxyindolenine **4a**: **4a** (5.00 mg, 23.2 µmol, 1.00 equiv.) and $Sc(OTf)_3$ (22.9 mg, 46.5 µmol, 2.00 equiv.) were placed in a *Schlenk* flask and suspended in toluene (4 mL). The reaction mixture was stirred at 100 °C for 2 h, then cooled to room temperature and quenched with a saturated aqueous $NaHCO_3$ solution (5 mL). The rose-colored mixture was extracted with EtOAc (3 × 10 mL). The combined organic layers were washed with brine, dried over Na_2SO_4, filtered, and concentrated under reduced pressure to afford 2-oxindole **19** (4.80 mg, 22.3 µmol, 96%) as an orange solid.

Rearrangement starting from 3-oxindole **18**:

A round-bottom flask was charged with **18** (50.0 mg, 232 µmol, 1.00 equiv.) and toluene (4 mL). Scandium triflate (229 mg, 465 µmol, 2.00 equiv.) was added and the reaction mixture was stirred at 100 °C for 24 h. Subsequently, a second portion of scandium triflate (229 mg, 465 µmol, 2.00 equiv.) was added and stirring was continued at 100 °C for an additional 72 h. The reaction was quenched by addition of a saturated aqueous sodium bicarbonate solution (10 mL) and the mixture was extracted with DCM (3 × 10 mL). The combined organic layers were washed with a saturated aqueous NaCl solution, dried over Na_2SO_4, filtered, and concentrated under reduced pressure to afford **19** as an orange solid (35.9 mg, 167 µmol, 72%).

Orange solid; **TLC** (*n*-pentane/EtOAc, 9/1): R_f = 0.27 [UV]; **^{1}H NMR** (500 MHz, $CDCl_3$, 298 K): δ (ppm) = 7.44 (br s, 1H, N*H*), 7.21 – 7.15 (m, 2H, $H^{7,9}$), 6.99 (*virt.* t, $^3J_{H\text{-}H} \approx {}^3J_{H\text{-}H}$ = 7.6 Hz, 1H, H^8), 6.83 (d, $^3J_{H\text{-}H}$ = 7.6 Hz, 1H, H^{10}), 2.32 – 2.24 (m, 1H, H^2), 2.22 – 2.16 (m, 1H, H^4), 2.13 – 2.02 (m, 2H, $H^{3,4}$), 2.00 – 1.90 (m, 1H, H^3), 1.71 – 1.64 (m, 1H, H^2), 1.00 (s, 3H, $H^{13/14}$), 0.91 (s, 3H, $H^{13/14}$); **^{13}C{^{1}H} NMR** (126 MHz, $CDCl_3$, 298 K): δ (ppm) = 182.9 (C^{12}), 141.2 (C^{11}), 132.1 (C^6), 127.6 ($C^{7/9}$), 125.5 ($C^{7/9}$), 121.7 (C^8), 109.1 (C^{10}), 61.5 (C^5), 47.1 (C^1), 39.0 (C^2), 33.8 (C^4), 26.0 ($C^{13/14}$), 23.7 ($C^{13/14}$), 21.2 (C^3); **HR-MS** (ESI^+): *m/z* calculated for $[C_{14}H_{17}NO+H]^+$: 216.1383 ($[M+H]^+$); found: 216.1385; **IR:** $\tilde{\nu}$ = 3129 (w, NH), 3077 (w, sp^2-CH), 3032 (w, sp^2-CH), 2962 (m, sp^3-CH), 2930 (m, sp^3-CH), 2869 (w, sp^3-CH), 1699 (m,

C=O), 1618 (m, C=C), 1595 (w, C=C), 1470 (m), 1384 (w), 1366 (w), 1334 (m), 1303 (w), 1276 (w), 1253 (m), 1193 (m), 1157 (w), 1124 (w), 1111 (m), 1077 (w), 1019 (w), 990 (w), 947 (w), 894 (w), 857 (w), 812 (m, sp^2-CH), 740 (s, sp^2-CH), 681 (m) cm^{-1}.

2.3.18 Methyl (1,1-dimethyl-2,3,4,9-tetrahydro-1*H*-carbazol-4-yl)carbamate (20)

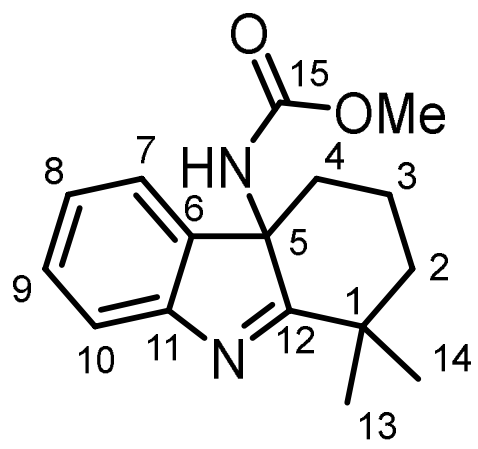

20

A *Schlenk* tube was filled with hydroxyindolenine **4a** (25.0 mg, 116 µmol, 1.00 equiv.) and acetonitrile (3 mL). *Burgess* reagent (276 mg, 1.16 mmol, 10.0 equiv.) was added and the mixture was stirred at 50 °C for 90 minutes. After removing the solvent under reduced pressure, the residue was purified using flash chromatography (silica, P/EtOAc = 9/1) to yield the title compound as a yellow oil (11.9 mg, 43.7 µmol, 38%).

Yellow oil (11.9 mg, 43.7 µmol, 38%); **TLC** (*n*-pentane/EtOAc, 9/1): R_f = 0.43 [UV]; **^{1}H NMR** (500 MHz, $CDCl_3$, 298 K): δ (ppm) = 10.91 (br s, 1H, N*H*), 7.30 – 7.27 (m, 1H, H^7), 7.23 (*virt*. td, $^3J_{H\text{-}H} \approx {}^3J_{H\text{-}H}$ = 7.7 Hz, $^4J_{H\text{-}H}$ = 1.2 Hz, 1H, H^9), 7.07 (*virt*. td, $^3J_{H\text{-}H} \approx {}^3J_{H\text{-}H}$ = 7.7 Hz, $^4J_{H\text{-}H}$ = 1.1 Hz, 1H, H^8), 6.99 – 6.88 (m, 1H, H^{10}), 3.80 (s, 3H, OCH_3), 2.51 – 2.43 (m, 1H, H^2), 2.35 – 2.27 (m, 1H, H^4), 2.23 – 2.16 (m, 1H, H^4), 2.16 – 2.08 (m, 1H, H^3), 2.06 – 1.93 (m, 1H, H^3), 1.72 – 1.65 (m, 1H, H^2), 0.98 (s, 3H, $H^{13/14}$), 0.87 (s, 3H, $H^{13/14}$); **^{13}C{^{1}H} NMR** (126 MHz, $CDCl_3$, 298 K): δ (ppm) = 180.9 (C^{12}), 164.9 (C^{15}), 141.5 (C^{11}), 132.8 (C^6), 127.9 (C^9), 125.4 (C^7), 122.8 (C^8), 110.3 (C^{10}), 64.8 (C^5), 53.1 (OCH_3), 48.5 (C^1), 39.3 (C^2), 34.6 (C^4), 26.8 ($C^{13/14}$), 23.4 ($C^{13/14}$), 21.5 (C^3); **HR-MS** (ESI$^+$): *m/z* calculated for $[C_{16}H_{20}N_2O_2+H]^+$: 273.1598 ($[M+H]^+$); found: 273.1591; **IR:** $\tilde{\nu}$ = 3318 (w, NH), 3051 (w, sp^2-CH), 2955 (w, sp^3-CH), 2862 (w, sp^3-CH), 1651 (m, C=O), 1607 (m, C=C), 1593 (m, C=C), 1575 (m, C=C), 1481 (m), 1465 (m), 1436 (m), 1347 (m), 1282 (w, O–C), 1240 (m), 1226 (m), 1185 (m), 1125 (w), 1109 (w), 1082 (w), 1058 (w), 1039 (w), 1019 (w), 976 (w), 949 (w), 925 (w), 901 (w), 888 (w), 866 (w), 807 (w, sp^2-CH), 750 (w, sp^2-CH), 697 (w, sp^2-CH) cm^{-1}.

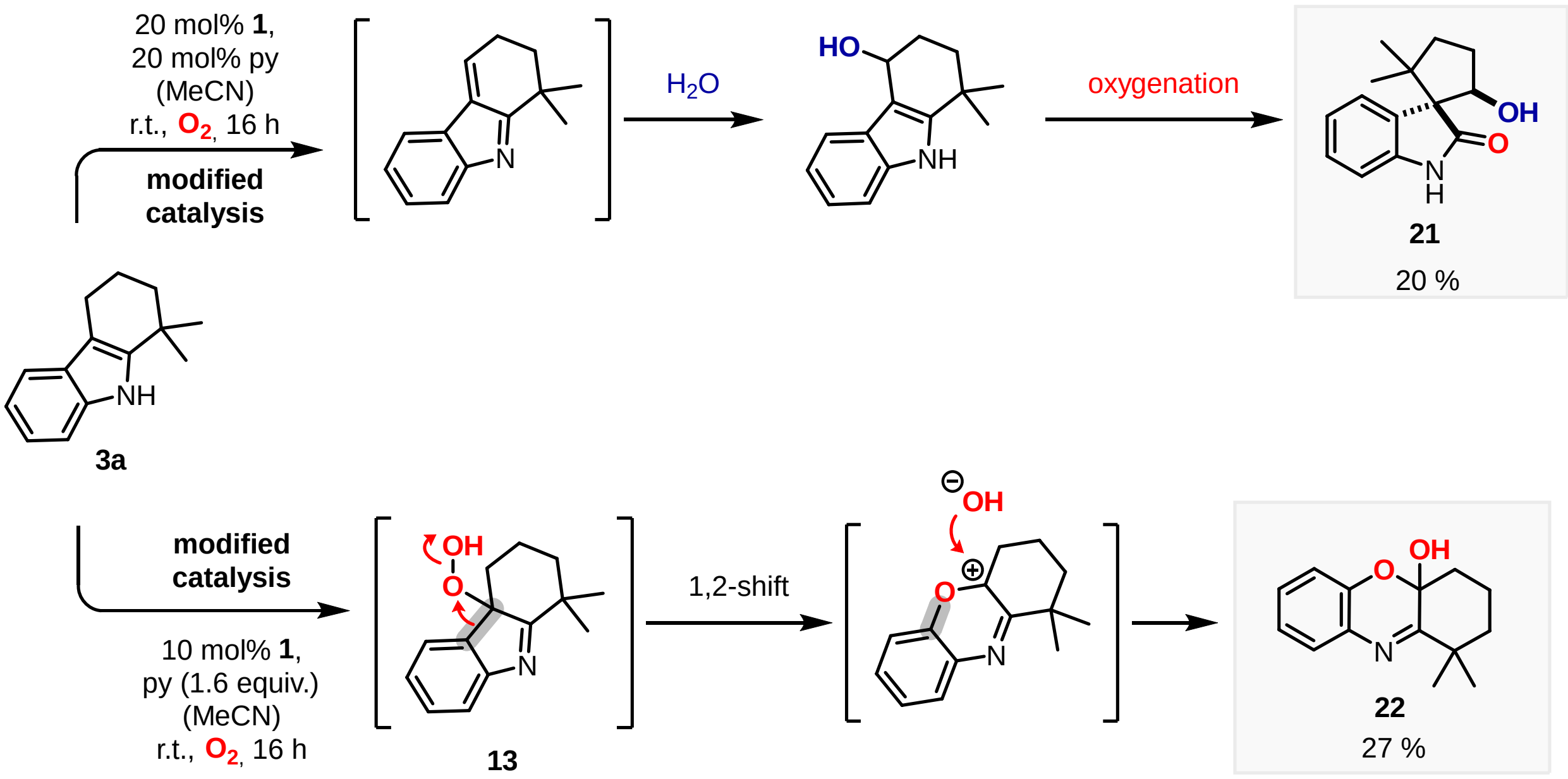

Figure SI-17. Plausible mechanism for the formation of catalysis oxygenation products **21** & **22** (py = pyridine).

2.3.19 (1S,5S)-5-Hydroxy-2,2-dimethylspiro[cyclopentane-1,3'-indolin]-2'-one (21)

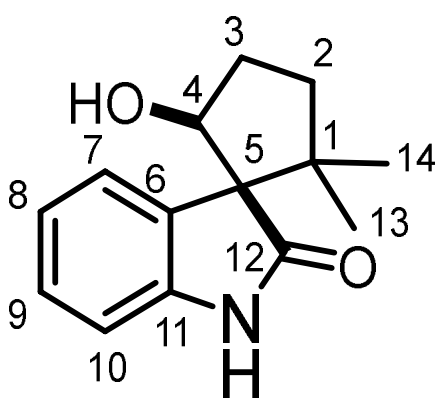

21

Tetrahydrocarbazole **3a** (100 mg, 500 µmol, 1.00 equiv.) and catalyst **1** (37.1 mg, 100 µmol, 0.20 equiv.) were placed into a *Schlenk* tube and dissolved in dry acetonitrile (10 mL, 50 mM). Pyridine (8.10 µL, 7.91 mg, 100 µmol, 0.20 equiv.) was added and the solution was sparged with oxygen for approximately 2 minutes. The reaction was then stirred at room temperature for 16 h. After that, the vial was uncapped and the solvent was removed under reduced pressure. The crude residue was dissolved in acetone and was purified by normal phase chromatography on the *Biotage* (*n*-heptane/EtOAc, gradient 10% → 100% EtOAc) to yield the title compound as a colorless solid (23.1 mg, 100 µmol, 20%).

Colorless solid (23.1 mg, 100 µmol, 20%); **TLC** (*n*-pentane/EtOAc, 7/3): R_f = 0.10 [UV]; **^{1}H NMR** (500 MHz, $CDCl_3$, 298 K): δ (ppm) = 7.46 (br s, 1H, N*H*), 7.25 – 7.19 (m, 2H, $H^{7,9}$), 7.03 (*virt.* dt, $^3J_{H\text{-}H} \approx {}^3J_{H\text{-}H}$ = 7.7 Hz, $^4J_{H\text{-}H}$ = 1.1 Hz, 1H, H^8), 6.84 (*virt.* dt, $^3J_{H\text{-}H} \approx {}^3J_{H\text{-}H}$ = 7.7

Hz, $^4J_{H-H}$ = 0.8 Hz, 1H, H^{10}), 4.72 (dd, $^3J_{H-H}$ = 9.1, 6.3 Hz, 1H, H^4), 2.59 – 2.40 (m, 2H, $H^{2,3}$), 2.11 – 1.96 (m, 1H, H^3), 1.64 – 1.58 (m, 1H, H^2), 1.11 (s, 3H, $H^{13/14}$), 0.89 (s, 3H, $H^{13/14}$); **^{13}C{^{1}H} NMR** (126 MHz, $CDCl_3$, 298 K): δ (ppm) = 179.6 (C^{12}), 141.9 (C^{11}), 129.4 (C^6), 128.2 (C^9), 125.5 (C^7), 122.1 (C^8), 109.4 (C^{10}), 79.8 (C^4), 67.4 (C^5), 45.7 (C^1), 37.0 (C^2), 31.5 (C^3), 27.4 ($C^{13/14}$), 24.7 ($C^{13/14}$); **HR-MS** (ESI$^+$): *m/z* calculated for $[C_{14}H_{17}NO_2+H]^+$: 232.1332 ($[M+H]^+$); found: 232.1327; **IR:** $\tilde{\nu}$ = 3310 (w, OH), 3220 (w, NH), 3027 (w, sp^2-CH), 2969 (m, sp^3-CH), 2938 (m, sp^3-CH), 2870 (w, sp^3-CH), 1759 (w, C=O), 1689 (m, C=O), 1618 (m, C=C), 1470 (m), 1456 (m), 1439 (m), 1403 (w), 1385 (w), 1368 (w), 1339 (m), 1301 (m), 1261 (w), 1198 (m), 1166 (w), 1122 (m), 1100 (s), 1039 (w), 1022 (w), 988 (w), 958 (w), 922 (w), 884 (w), 857 (w), 836 (w), 802 (w, sp^2-CH), 744 (s, sp^2-CH), 683 (m, sp^2-CH), 661 (m, sp^2-CH) cm^{-1}.

2.3.20 1,1-Dimethyl-1,2,3,4-tetrahydro-4a*H*-phenoxazin-4a-ol (22)

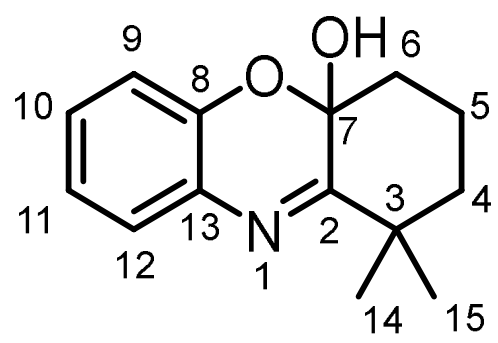

22

Tetrahydrocarbazole **3a** (100 mg, 500 µmol, 1.00 equiv.) and catalyst **1** (18.5 mg, 50.0 µmol, 10 mol%) were placed into a *Schlenk* tube and dissolved in dry acetonitrile (10 mL, 50 mM). Pyridine (64.6 µL, 63.3 mg, 800 µmol, 1.60 equiv.) was added and the solution was sparged with oxygen for approximately 2 minutes. The reaction was then stirred at room temperature for 16 h. After that, the vial was uncapped and the solvent was removed under reduced pressure. The crude residue was dissolved in acetone and was purified by normal phase chromatography on the *Biotage* (*n*-heptane/EtOAc, gradient 10% → 100% EtOAc) to yield the title compound as a colorless solid (31.2 mg, 135 µmol, 27%).

22 is not stable for prolonged times in $CDCl_3$ likely due to acid traces.

Colorless solid (31.2 mg, 135 µmol, 27%); **TLC** (*n*-pentane/EtOAc, 9/1): R_f = 0.29 [UV]; **^{1}H NMR** (500 MHz, $CDCl_3$, 298 K): δ (ppm) = 7.44 (dd, $^3J_{H-H}$ = 7.7 Hz, $^4J_{H-H}$ = 1.6 Hz, 1H, H^{12}), 7.17 (*virt*. td, $^3J_{H-H} \approx {}^3J_{H-H}$ = 7.7 Hz, $^4J_{H-H}$ = 1.7 Hz, 1H, H^{10}), 7.03 (*virt*. td, $^3J_{H-H} \approx {}^3J_{H-H}$ = 7.7 Hz, $^4J_{H-H}$ = 1.3 Hz, 1H, H^{11}), 6.94 (dd, $^3J_{H-H}$ = 7.7 Hz, $^4J_{H-H}$ = 1.4 Hz, 1H, H^9), 2.66 (br s, 1H, OH), 2.42 – 2.30 (m, 1H, H^6), 2.05 – 1.95 (m, 2H, $H^{5,6}$), 1.76 – 1.68 (m, 2H, $H^{4,5}$), 1.63

– 1.59 (m, 1H, H^4), 1.38 (s, 3H, H$^{14/15}$), 1.36 (s, 3H, H$^{14/15}$); **^{13}C{^{1}H} NMR** (126 MHz, CDCl$_3$, 298 K): δ (ppm) = 169.8 (C^2), 144.3 (C^8), 132.3 (C^{13}), 128.5 (C^{10}), 127.5 (C^{12}), 122.4 (C^{11}), 116.8 (C^9), 91.4 (C^7), 39.5 (C^6), 39.4 (C^4), 39.4 (C^3), 29.5 (C$^{14/15}$), 28.3 (C$^{14/15}$), 18.0 (C^5); **HR-MS** (ESI$^+$): *m/z* calculated for [$C_{14}H_{17}NO_2$+H]$^+$: 232.1332 ([M+H]$^+$); found: 232.1327; **IR:** $\tilde{\nu}$ = 3355 (w, O–H), 2989 (m, sp^3-CH), 2922 (s, sp^3-CH), 2872 (m, sp^3-CH), 1674 (m, C=O), 1619 (s, C=C), 1484 (s), 1384 (m), 1261 (s, C-O), 1166 (s), 1123 (s), 1075 (m), 1005 (s), 969 (m), 910 (m), 820 (m), 749 (s, sp^2-CH), 689 (m, sp^2-CH) cm^{-1}.

2.3.21 3,3-Dimethyl-4,5-dihydrobenzo[*b*]azocine-2,6(1*H*,3*H*)-dione (SI-30)

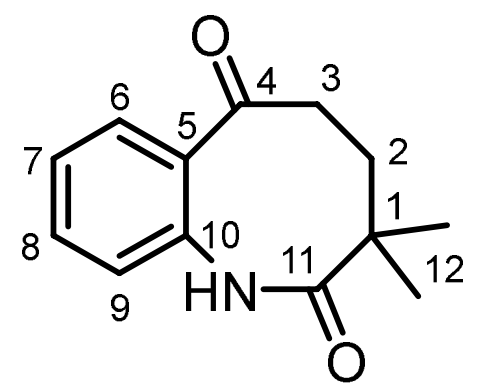

SI-30

9.26 mg (50.0 µmol, 1.00 equiv.) of 3,3-dimethyl-1,2,3,4-tetrahydrocyclopenta[*b*]indole (**SI-17**) were weighed into a crimp vial. A catalyst solution was prepared by dissolving 1.85 mg (5 µmol, 0.10 equiv.) of the flavin catalyst **1** together with 1.11 µL (10.0 µmol, 0.20 equiv.) *N*-methylmorpholine in 1 mL of MeCN and was added to the starting material. The vial was capped and purged with oxygen for approximately 8 seconds. The reaction was then stirred at room temperature for 48 h. After that, the vial was uncapped and 5.91 µL (50.0 µmol, 1.00 equiv.) of trimethyl phosphite were added. The mixtures were stirred open to air at room temperature for an additional 30 minutes and the solvent was subsequently removed under reduced pressure using a rotary evaporator. The crude residues were dissolved in acetone and applied to a reversed-phase C18 column. Purification was carried out using reverse phase *Biotage* flash chromatography (MeCN/H_2O, gradient 10% → 100% MeCN). Finally, solvents were removed by freeze-drying using a lyophilizer, resulting in 6.74 mg (31.0 µmol, 62%) of an orange solid.

Orange solid, 6.74 mg (62%); **TLC:** R_f = 0.26 (*n*-pentane/EtOAc, 8/2) [UV]; **^{1}H NMR** (500 MHz, CDCl$_3$, 298 K): δ (ppm) = 7.65 (dd, $^3J_{H-H}$ = 7.7 Hz, $^4J_{H-H}$ = 1.7 Hz, 1H, H^6), 7.50 (td, $^3J_{H-H}$ = 7.7 Hz, $^4J_{H-H}$ = 1.7 Hz, 1H, H^8), 7.36 (td, $^3J_{H-H}$ = 7.7 Hz, $^4J_{H-H}$ = 1.2 Hz, 1H, H^7), 7.35 (s, 1H, NH), 7.14 (dd, $^3J_{H-H}$ = 7.7 Hz, $^4J_{H-H}$ = 1.2 Hz, 1H, H^9), 2.93 – 2.81 (m, 2H, H^3), 1.98 – 1.85 (m, 2H, H^2), 1.19 (s, 6H, H^{12}); **^{13}C{^{1}H} NMR** (126 MHz, CDCl$_3$, 298 K):

δ (ppm) = 204.5 (C^4), 177.7 (C^{11}), 136.0 (C^{10}), 135.7 (C^5), 133.0 (C^8), 129.1 (C^6), 127.7 (C^7), 126.5 (C^9), 43.8 (C^1), 40.2 (C^3), 38.0 (C^2), 27.7 (C^{12}); **HR-MS** (ESI^+): *m/z* calculated for $[C_{13}H_{15}NO_2+H]^+$: 218.1176 ($[M+H]^+$); found: 218.1176; **IR:** $\tilde{\nu}$ = 3460 (w, NH), 3342 (w, NH), 2971 (w, sp^3-CH), 1724 (s, C=O), 1692 (s), 1649 (vs, C=O), 1616 (s), 1598 (m, C=C), 1551 (w, C=C), 1476 (m, C=C), 1452 (m, C=C), 1395 (m), 1363 (w), 1287 (m), 1262 (w), 1210 (w), 1161 (m), 1131 (w), 947 (w), 865 (w), 749 (s) cm^{-1}.

3. Reaction Development

3.1. Catalytic *Witkop* Oxidation with H_2O_2 as Terminal Oxidant

The reactions were performed according to General Procedure A, but instead of purification by chromatography, a solution of 1,3,5-trimethoxybenzene (**IS**, 0.33 equiv. respective to the starting material) in $CDCl_3$ was added after solvent removal. Yields were determined by ^{1}H NMR spectroscopy (relaxation delay ≥ 5 s) using integration against the internal standard. Below, a representative example (Table **SI-1**, Entry 14 with 18% SM **3a**, 36% **4a**, 40% **6a**) is given:

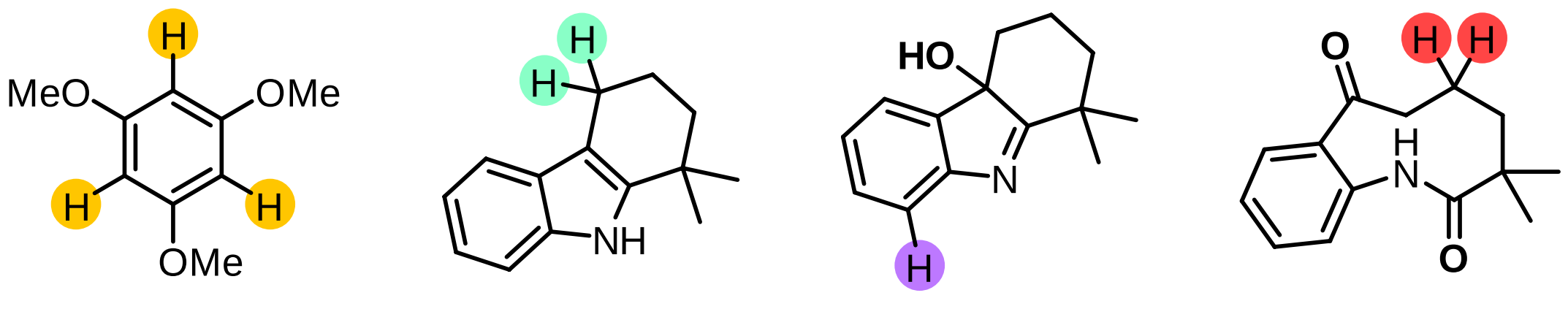

IS **3a** **4a** **6a**

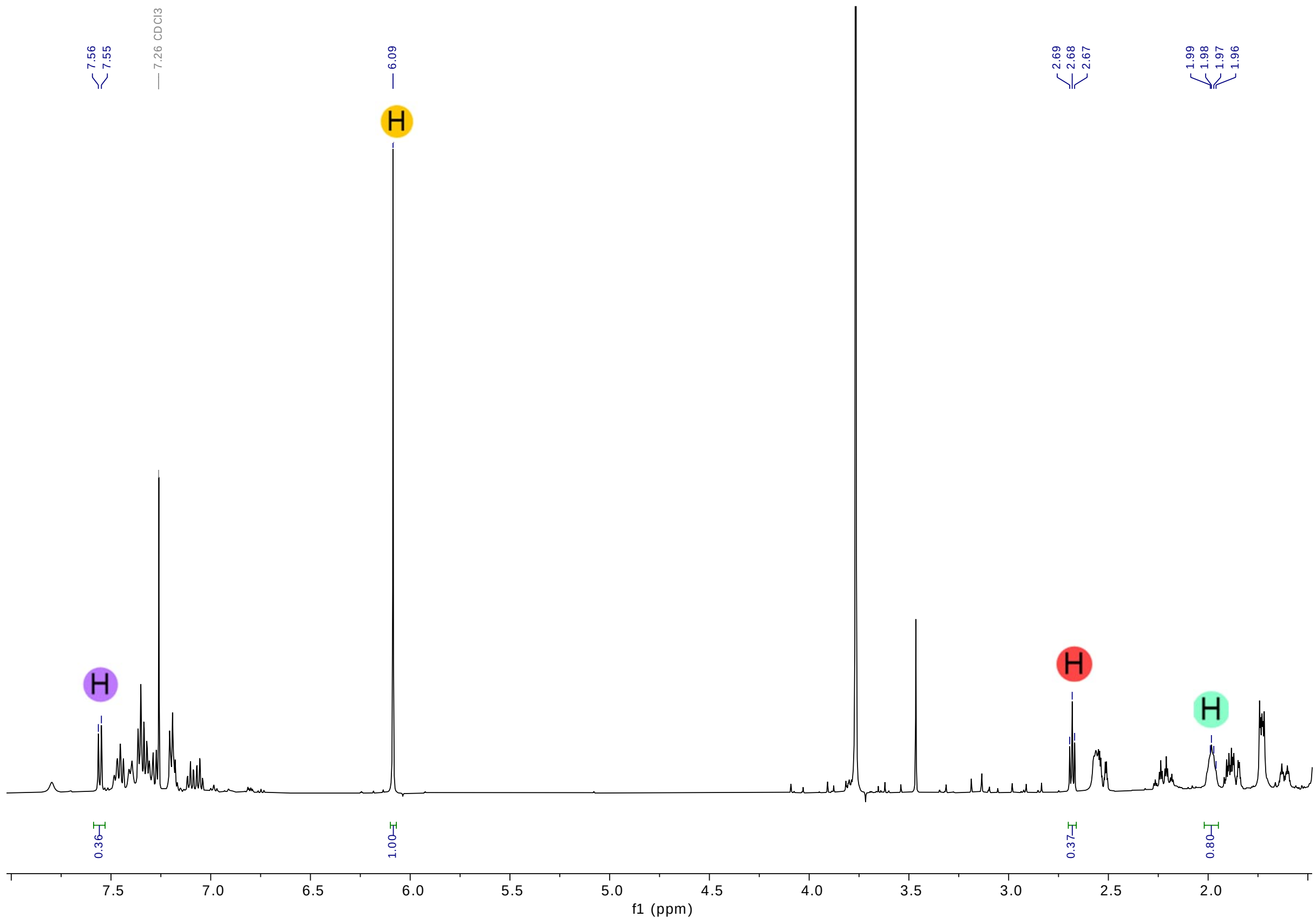

Figure SI-18. Example screening spectrum and signals chosen for NMR yield quantification.

Table SI-1. Screening of the catalytic *Witkop* oxidation with H_2O_2 as terminal oxidant.

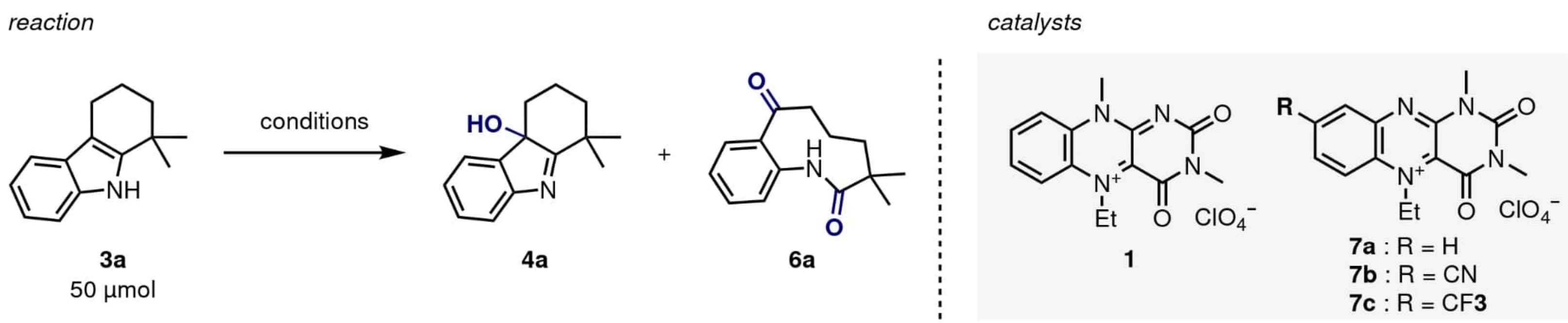

	Entry #	Catalyst (mol%)	H_2O_2 (equiv.)	Solvent [*mM*]	Variation	3a [%]	4a [%]	6a [%]	Mass balance [%]
Blanks	1	**7c** (2)	-	MeOH [50]	-	97	0	0	97
	2	-	5.0	MeOH [50]	-	98	0	0	98
	3	-	UHP (5.0)	MeOH [50]	-	100	0	0	100
Catalysts	4	**7c** (2)	1.0	MeOH [333]	-	35	40	14	89
	5	**7b** (2)	1.0	MeOH [333]	-	40	34	14	88
	6	**7a** (2)	1.0	MeOH [333]	-	93	7	0	100
	7	**1** (2)	1.0	MeOH [333]	2% $HClO_4$	61	16	9	86
	8	**7c** (2)	4.0	MeOH [333]	-	0	0	55	55
	9	**7c** (2)	4.0	MeOH [333]	-	32	16	20	68
	10	**1** (2)	4.0	MeOH [333]	-	13	0	37	50
Amount H_2O_2	11	**7c** (2)	-	MeOH [333]	-	98	0	0	98
	12	**7c** (2)	1.0	MeOH [333]	-	35	40	14	89
	13	**7c** (2)	1.5	MeOH [333]	-	27	48	30	105
	14	**7c** (2)	2.0	MeOH [333]	-	18	36	40	84
	15	**7c** (2)	5.0	MeOH [333]	-	3	0	62	65

UHP = Urea hydrogen peroxide adduct, 1,3,5-Trimethoxybenzene in $CDCl_3$ used as internal standard (IS)

(*Table from last page continued*)

	Entry #	Catalyst (mol%)	H_2O_2 (equiv.)	Solvent [*mM*]	Variation	3a [%]	4a [%]	6a [%]	Mass balance [%]
Reaction time	16	**7c** (2)	1.0	MeOH [333]	30 min	69	20	0	89
	17	**7c** (2)	1.0	MeOH [333]	1.5h	66	24	0	90
	18	**7c** (2)	1.0	MeOH [333]	2.5h	64	26	traces	100
	19	**7c** (2)	1.0	MeOH [333]	4h	38	40	7	85
	20	**7c** (2)	1.0	MeOH [333]	16h	36	24	17	77
	21	**7c** (2)	1.0	MeOH [333]	65h	29	30	22	81
Red. agents, O_2	22	**7c** (2)	-	MeOH [333]	Zn powder (1.0 eq.), O_2	99	0	0	99
	23	**7c** (2)	-	MeOH [333]	Fe powder (1.0 eq.), O_2	99	0	0	99
	24	**7c** (2)	-	MeOH [333]	BNAH (1.0 eq.), O_2	100	0	0	100
	25	**7c** (2)	-	MeOH [333]	HEH (1.0 eq.), O_2	95	0	0	95
	26	**7c** (2)	-	MeOH [333]	$Na_2S_2O_4$ (1.0 eq.), O_2	99	0	0	99
Red. flavin, H_2O_2	27	**8** (20)	5.0	MeOH [50]	18 h	27	49[a]	21	97

BNAH = 1-Benzyl-1,4-dihydronicotinamide, HEH = Hantzsch ester

[a] The yield of 49% is a combined yield of hydroxyindolenine **4a** (25%) and hydroperoxyindolenine **13**.

3.2 Catalytic Aerobic Oxygenation of Tetrahydrocarbazole 3a to Hydroxyindolenine 4a

The reactions were performed according to General Procedure B, but after stirring for 24 h at room temperature the vial was uncapped and the solvent was removed *in vacuo*. The crude residue was dissolved in a solution of 1,3,5-trimethoxybenzene (**IS**, 0.33 equiv. respective to the starting material) in $CDCl_3$. Yields were determined by ^1H NMR spectroscopy (relaxation delay ≥ 5 s) using integration against the internal standard. Below, a representative example (following Table, Entry 27 with 30% SM **3a**, 31% **4a+13**, 5% **21,** and 25% **22**) is given.

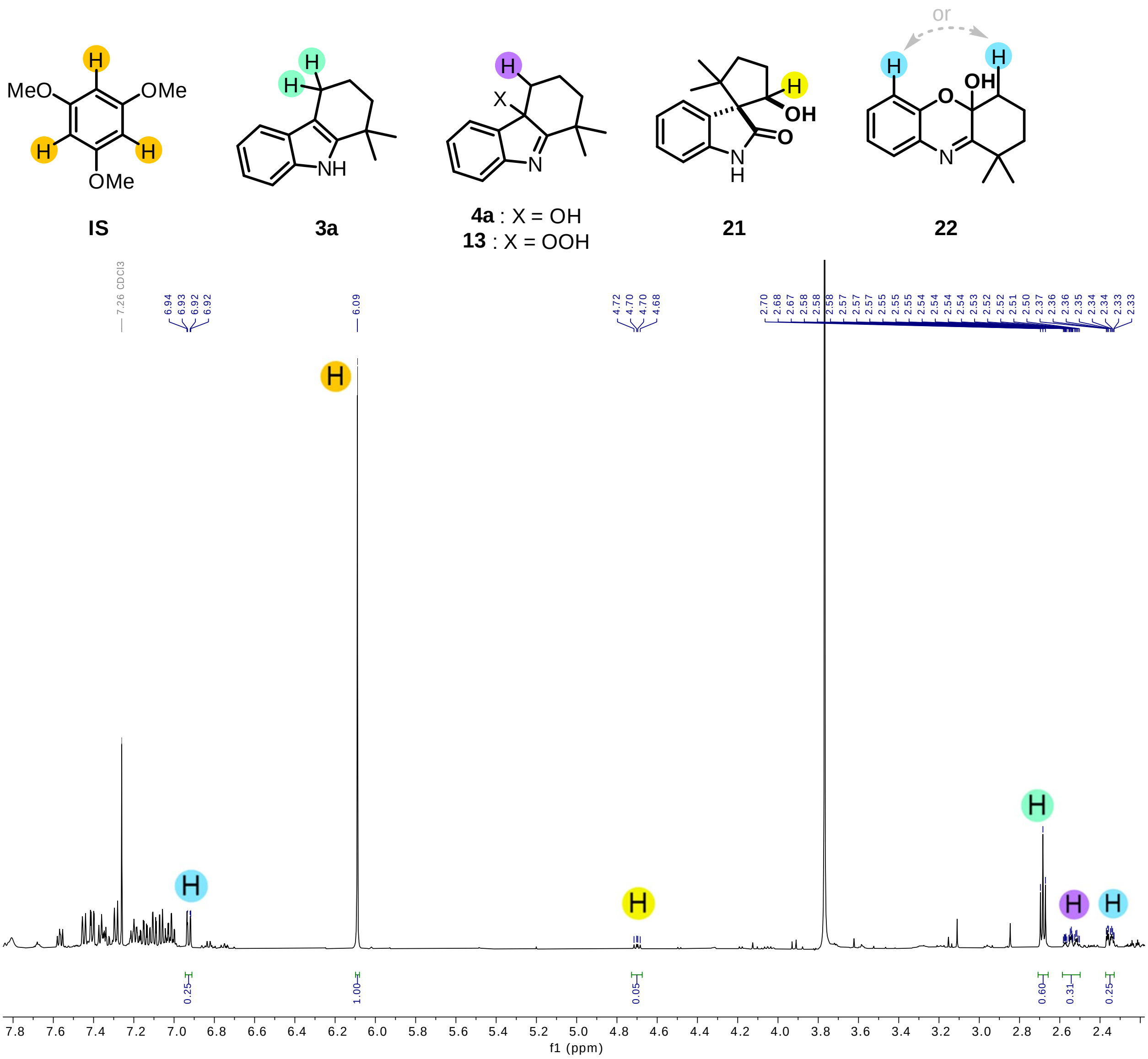

Figure SI-19. Example screening spectrum and signals chosen for NMR yield quantification.

Table SI-2. Screening for the catalytic aerobic oxygenation of tetrahydrocarbazole **3a** to hydroxyindolenine **4a**.

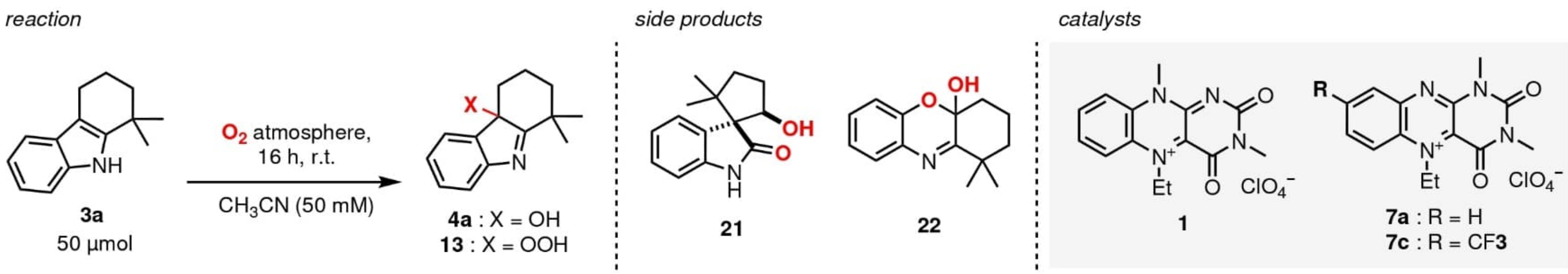

	Entry #	Catalyst (mol%)	Base (equiv.)	Variation	3a [%]	4a+13 [%]	21 [%]	22 [%]	Mass balance [%]
Blanks	1	**1** (10)	-	-	88	0	0	0	88
	2	**1** (10)	NMM (0.2)	under argon	92	0	0	0	92
	3	-	NMM (0.2)	-	98	0	0	0	98
	4	-	py (0.2)	-	94	0	0	0	94
Base amount	5	**1** (10)	py (0.1)	-	58	31	7	6	102
	6	**1** (10)	py (0.2)	-	41	30	8	21	100
	7	**1** (10)	py (0.4)	-	27	37	7	30	101
	8	**1** (10)	py (0.8)	-	20	32	7	33	92
	9	**1** (10)	py (1.6)	-	20	28	7	32	87
Catalyst loading	10	**1** (5)	py (0.2)	-	37	39	4	22	102
	11	**1** (15)	py (0.2)	-	28	50	15	10	103
	12	**1** (20)	py (0.2)	-	17	40	20	8	85
Temperature	13	**1** (10)	py (0.2)	ice bath	39	24	5	23	91
	14	**1** (10)	NMM (0.2)	40 °C	87	5	0	0	92
	15	**1** (10)	NMM (0.2)	70 °C	85	5	0	0	90
Catalysts	16	**7a** (10)	py (0.2)	-	74	13	2	0	89
	17	**7c** (10)	py (0.2)	-	72	17	0	0	89
Solvent	18	**1** (10)	py (0.2)	DMF instead of CH_3CN	89	0	0	0	89

NMM = *N*-Methyl morpholine, py = pyridine, DMF = *N*,*N*-Dimethylformamide, 1,3,5-Trimethoxybenzene in $CDCl_3$ used as internal standard (IS) added after solvent removal directly

(*Table from last page continued*)

	Entry #	Catalyst (mol%)	Base (equiv.)	Variation (equiv.)	3a [%]	4a+13 [%]	21 [%]	22 [%]	Mass balance [%]
Reductant for **13**→**4a**	19	**1** (10)	NMM (0.2)	Na_2SO_3 (1.0)	25	24	3	0	81
	20	**1** (10)	py (0.2)	$P(OMe)_3$ (1.0)	95	0	0	0	95
	21	**1** (10)	py (0.2)	PPh_3 (1.0)	100	0	0	0	100
	22	**1** (10)	py (0.2)	$P(OMe)_3$ (1.0) [a]	8	63[b]	10	0	81
	23	**1** (10)	NMM (0.2)	PPh_3 (1.0) [a]	30	55[b]	2	0	87
	24	**1** (10)	NMM (0.2)	$P(OMe)_3$ (1.0) [a]	27	70[b]	1	0	97
Water	25	**1** (10)	NMM (0.2)	H_2O (10)	10	61	3	0	74
	26	**1** (10)	py (0.2)	H_2O (36.6 ppm)	24	30	5	23	82
	27	**1** (10)	py (0.2)	H_2O (0.5)	30	31	5	25	91
	28	**1** (10)	py (0.2)	H_2O (1.0)	26	25	5	20	76
	29	**1** (10)	py (0.2)	H_2O (2.0)	28	24	4	21	77
	30	**1** (10)	py (0.2)	H_2O (5.0)	28	28	4	19	79
	31	**1** (10)	py (0.2)	H_2O (10.0)	15	49	5	11	80
	32	**1** (10)	py (0.2)	H_2O (22.2)	21	41	6	8	76
	33	**1** (10)	py (0.2)	H_2O (27.8)	21	45	6	8	80
	34	**1** (10)	py (0.2)	H_2O (38.9)	19	55	7	11	92
	35	**1** (10)	py (0.2)	H_2O (111.1)	49	37	3	0	89

1,3,5-Trimethoxybenzene used as internal standard (IS) [a] reductant added after 16 h reaction time, stirring 30 min at r.t. with reductant. [b] yield refers to only **4a**, no **13** observed in the run.

Furthermore, a screening of different bases was performed, as shown below:

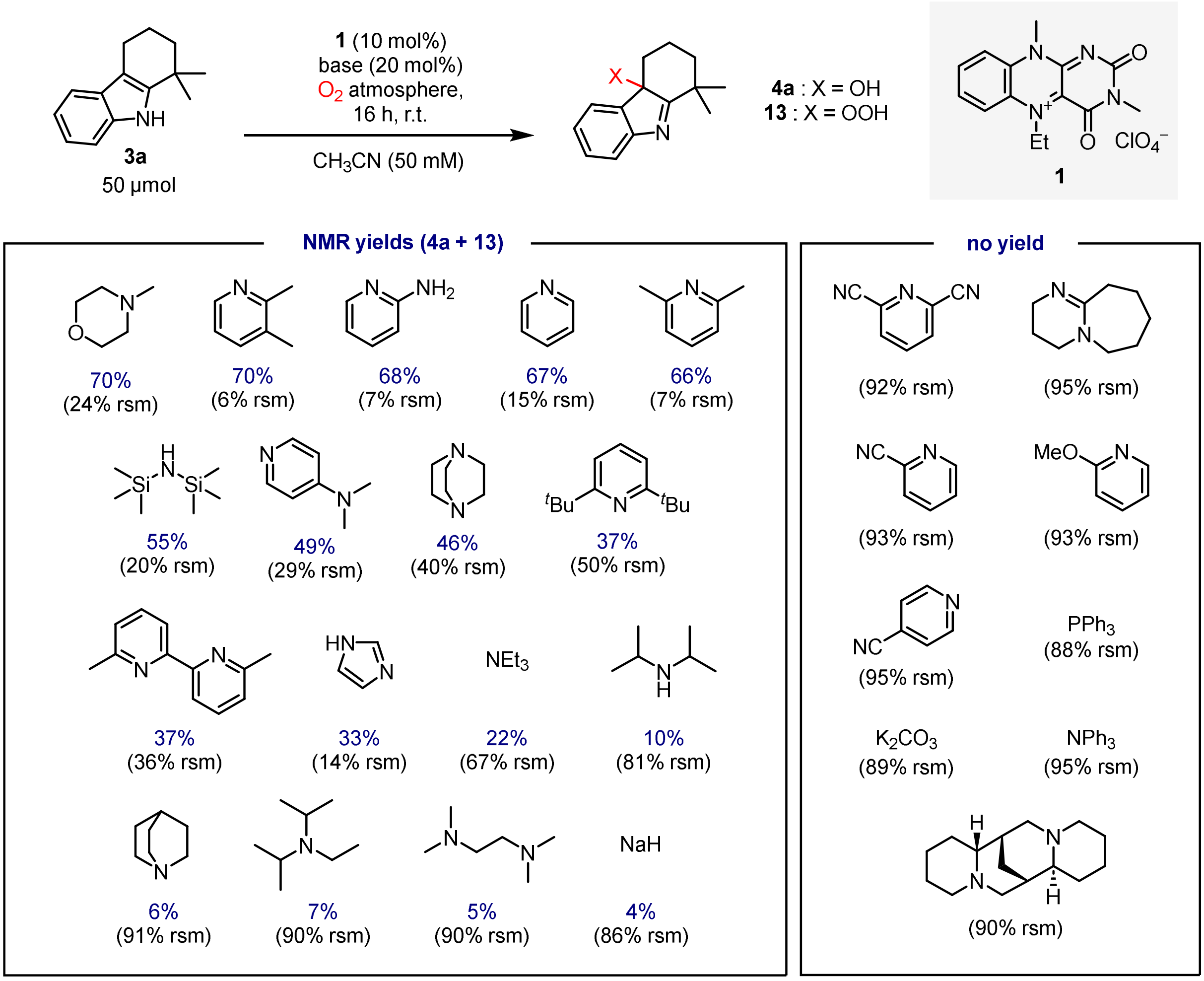

Figure SI-20. Bases screened for the catalytic aerobic oxygenation of tetrahydrocarbazole **3a** to hydroxyindolenine **4a**. Yields determined by ^{1}H NMR analysis of the crude reaction with 1,3,5-trimethoxybenzene as internal standard (rsm = remaining starting material).

3.3 Failed substrates

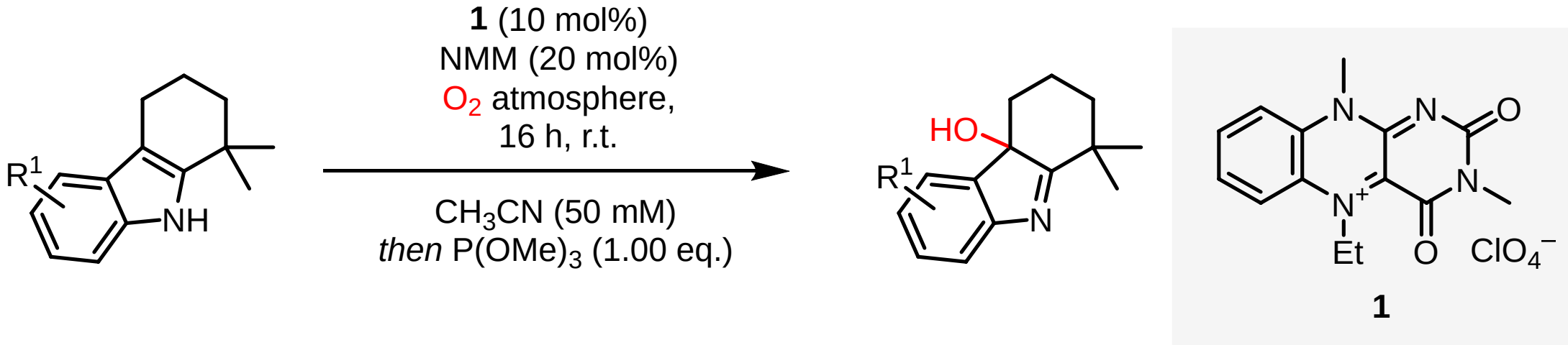

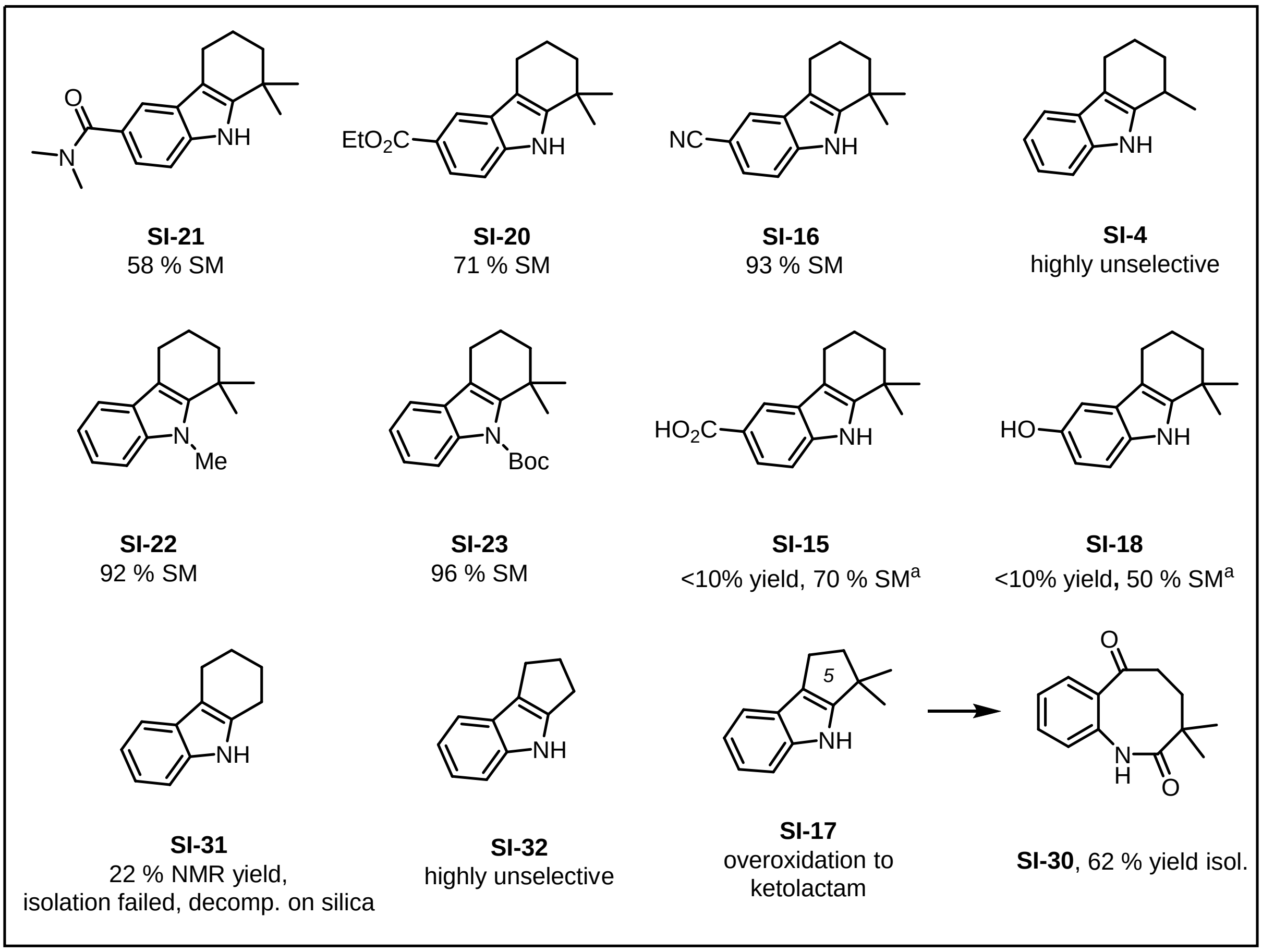

Figure SI-21. Failed and underdeveloped substrates. Yields determined by ^{1}H NMR analysis of the crude reaction mixture (except for **SI-17** → **SI-30**; **SI-30** was isolated) using 1,3,5-trimethoxybenzene as an internal standard. [a] 1.20 equiv. *N*-methyl morpholine (NMM) used.

4 UV/Vis Studies

The UV/Vis absorption spectra were measured in HPLC grade acetonitrile, which was degassed three times using the freeze–pump–thaw method. All spectra were recorded at room temperature in a quartz cuvette (0.5 mm pathlength) equipped with a *Schlenk* attachment and sealed under argon. Samples were prepared in a glovebox under strict exclusion of oxygen.

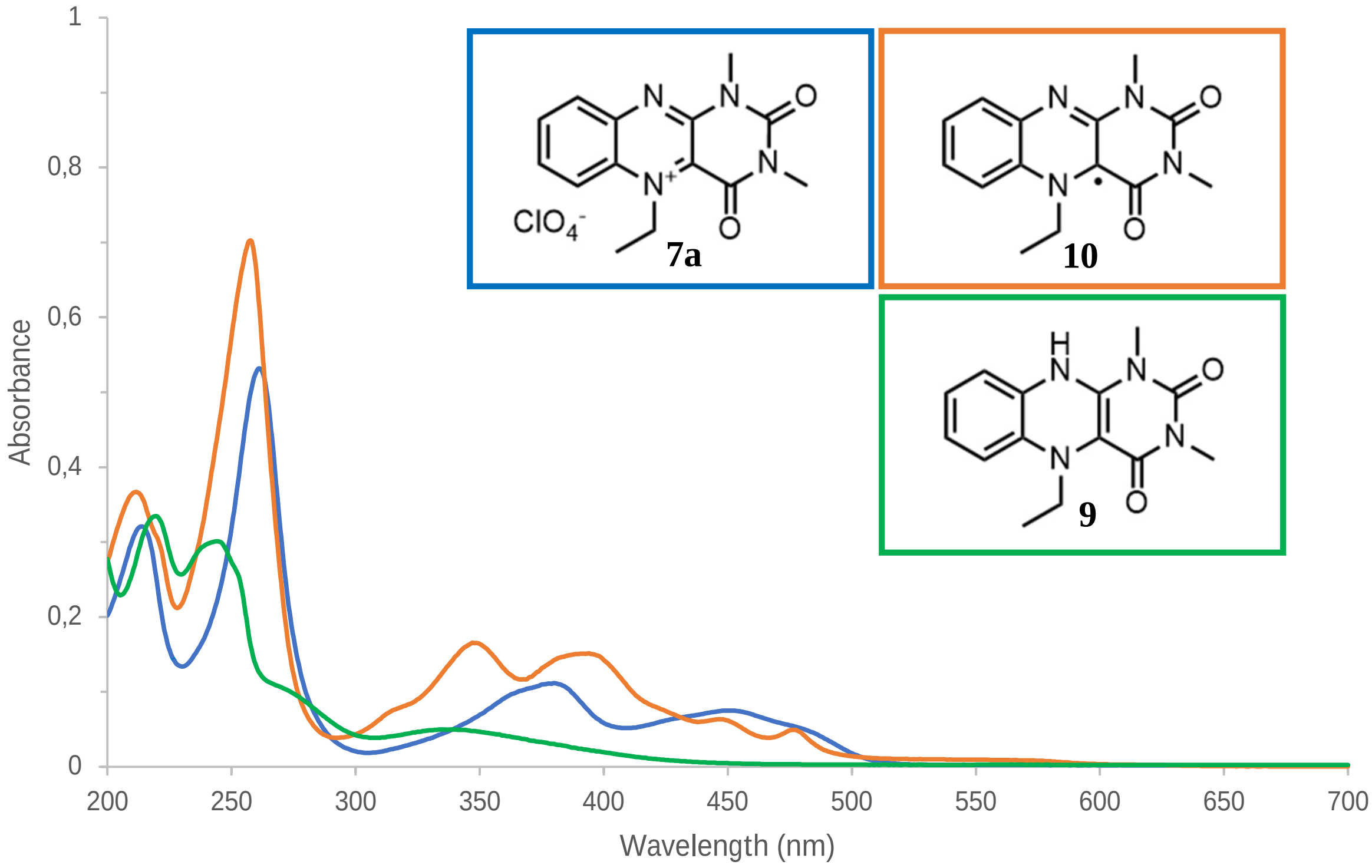

Figure SI-22. Stacked UV/Vis spectra of the different redox states of the alloxazine system. Blue = **7a**, orange = **10**, green = **9**; all spectra are measured with c = 0.368 mM.

7a: UV/Vis (CH_3CN, c = 0.368 mM): λ_{max} (absorption) = 214 (0.3209), 261 (0.5317), 378 (0.1111), 380 (0.1116), 450 nm (0.0750). Absorbances are absolute.

10: UV/Vis (CH_3CN, c = 0.368 mM): λ_{max} (ε) = 211 (19145), 258 (38182), 347 (8910), 368 (6210), 394 (8131), 447 nm (3373 $cm^{-1}M^{-1}$). ε was determined from a linear fit of absorbance vs. concentration ($R^2 \geq 0.999$).

9: UV/Vis (CH_3CN, c = 0.368 mM): λ_{max} (absorption) = 219 (0.3344), 244 (0.3012), 340 nm (0.0496). Absorbances are absolute.

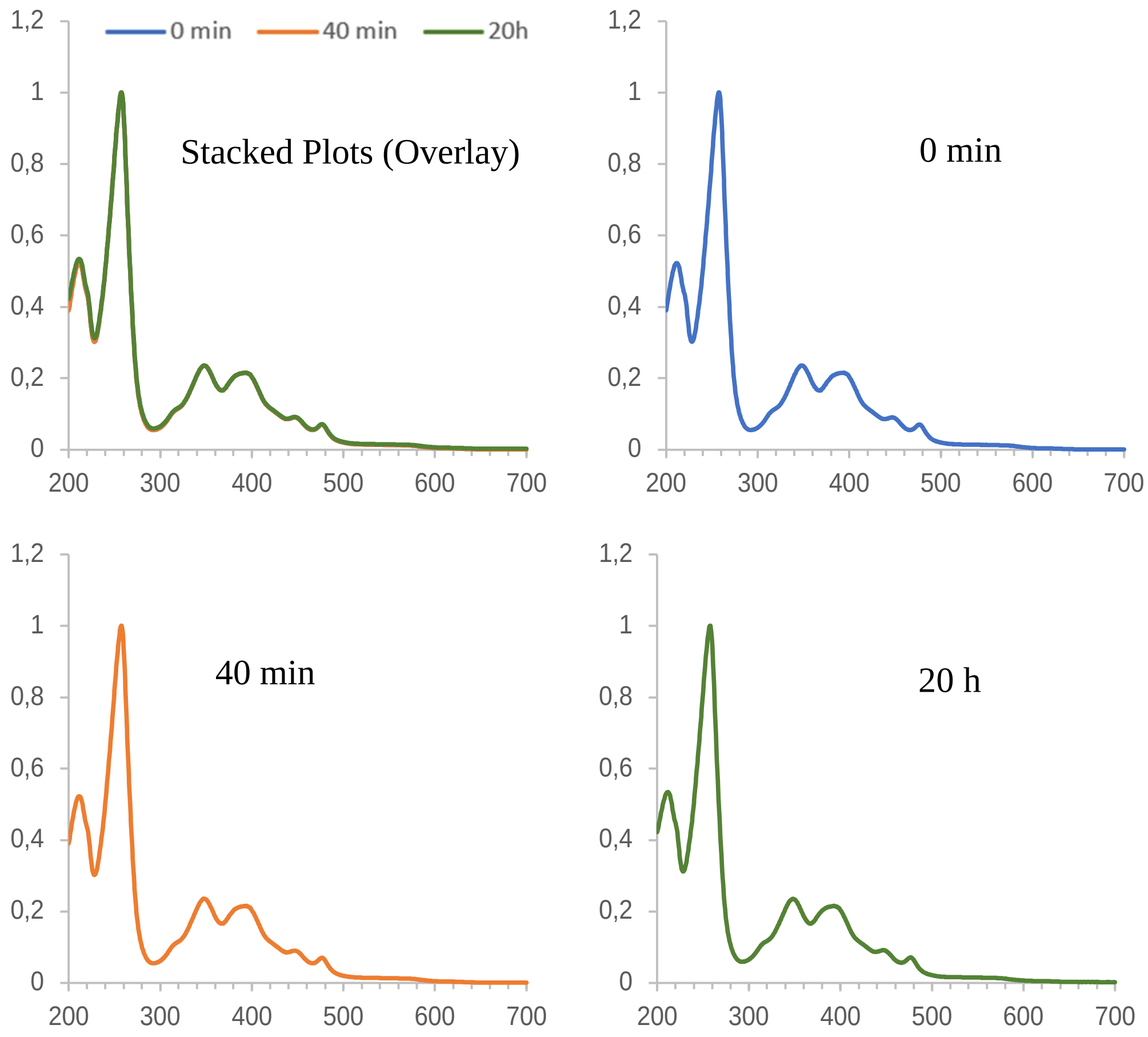

Figure SI-23. UV/Vis spectra of **10** in degassed acetonitrile under argon. The solution (c = 0.368 mM) was measured after t = 0 min (blue, top right), 40 min (orange, bottom left), 20 h (green, bottom right). All spectra are normalized and overlayed in a stack plot (top left) illustrating the stability of the radical in solution under oxygen exclusion.

5 EPR Measurements

All samples for EPR measurements were prepared in a glovebox under inert atmosphere. Solid state measurements of the alloxazine radical **10** and isoalloxazine radical **8** were recorded and showed a broad, unresolved paramagnetic signal (**Figure SI-17** and **Figure SI-18**). In $CDCl_3$ solution (c = 10^{-4} M), spectra of radicals **8** and **10** were obtained showing hyperfine coupling (**Figure SI-19** and **Figure SI-20**). For more insights into the reaction mechanism involved for the catalytic aerobic oxygenation of tetrahydrocarbazole **3a** to hydroxyindolenine **4a**, a reaction mixture under argon was prepared and measured (c = 10^{-4} M, **Figure SI-20**).

CW EPR spectra were recorded by using a JEOL JES-FA 200 spectrometer at X-band frequency. The spectra were measured at a microwave frequency of approximately 9.26 GHz with a microwave power of 5 mW, modulation amplitude of 0.04 mT, sweep time of 4 min, time constant of 0.1 s, and modulation frequency of 100 kHz. The microwave frequency was measured with a microwave frequency counter Advantest R5372. The experimental values of g values were determined by using Mn^{2+} (nuclear spin I = 5/2) embedded in MgO as a standard (using 4th line at g value of 1.981).

The experimental EPR spectra were simulated and fitted using MATLAB[23] Version: 23.2.0.2391609 (R2023b) Update 2 together with the Easyspin package (version 6.0.12).[24] Simulations and fits were performed using the garlic function. Experimental and simulated spectra were normalized prior to comparison. For improved visual comparison in the plots, the y-values of the simulated spectrum of **8** were vertically offset by +0.75, while the y-values of the simulated spectrum of **10** were offset by +0.75.

For the spectrum of alloxazine radical **10**, the best fit was obtained with a g value of 2.0039 and hyperfine couplings of A(^{14}N) = 20.41 MHz (1 nucleus), A(^{14}N) = 10.84 MHz (1 nucleus) and A(^{1}H) = 10.15 MHz (2 nuclei) using a line width for isotropic broadening lwpp = 0.351 mT.

For the spectrum of isoalloxazine **8**, the best fit was obtained with a g value of 2.0039 and hyperfine couplings of A(^{14}N) = 22.08 MHz (1 nucleus), A(^{14}N) = 8.83 MHz (1 nucleus), A(^{1}H) = 14.19 MHz (1 nucleus), A(^{1}H) = 9.60 MHz (1 nucleus), A(^{1}H) = 10.79 MHz (3 nuclei), and A(^{1}H) = 5.41 MHz (2 nuclei) using lwpp = 0.147 mT.

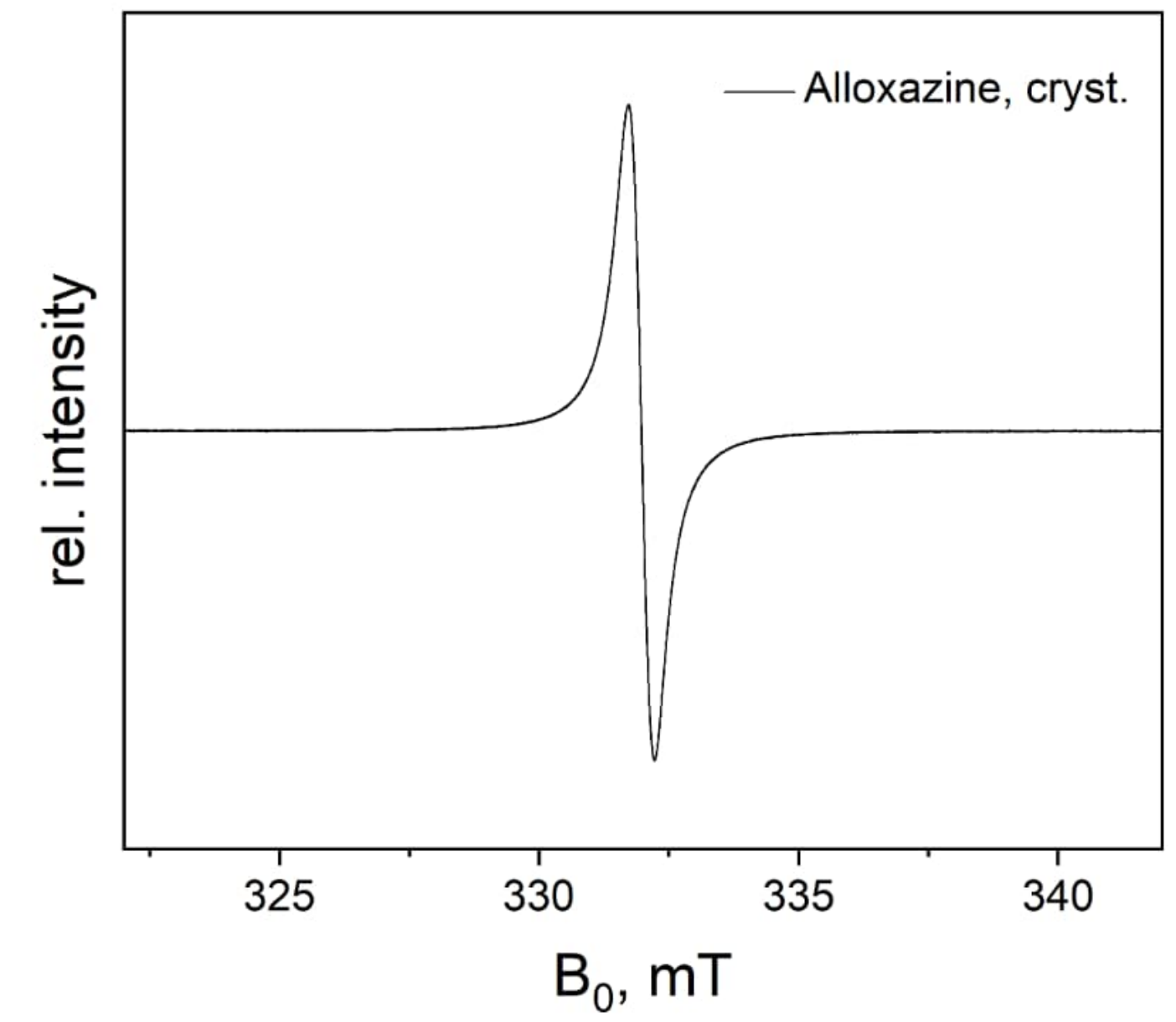

Figure SI-24. Solid state EPR spectrum of alloxazine radical **10**.

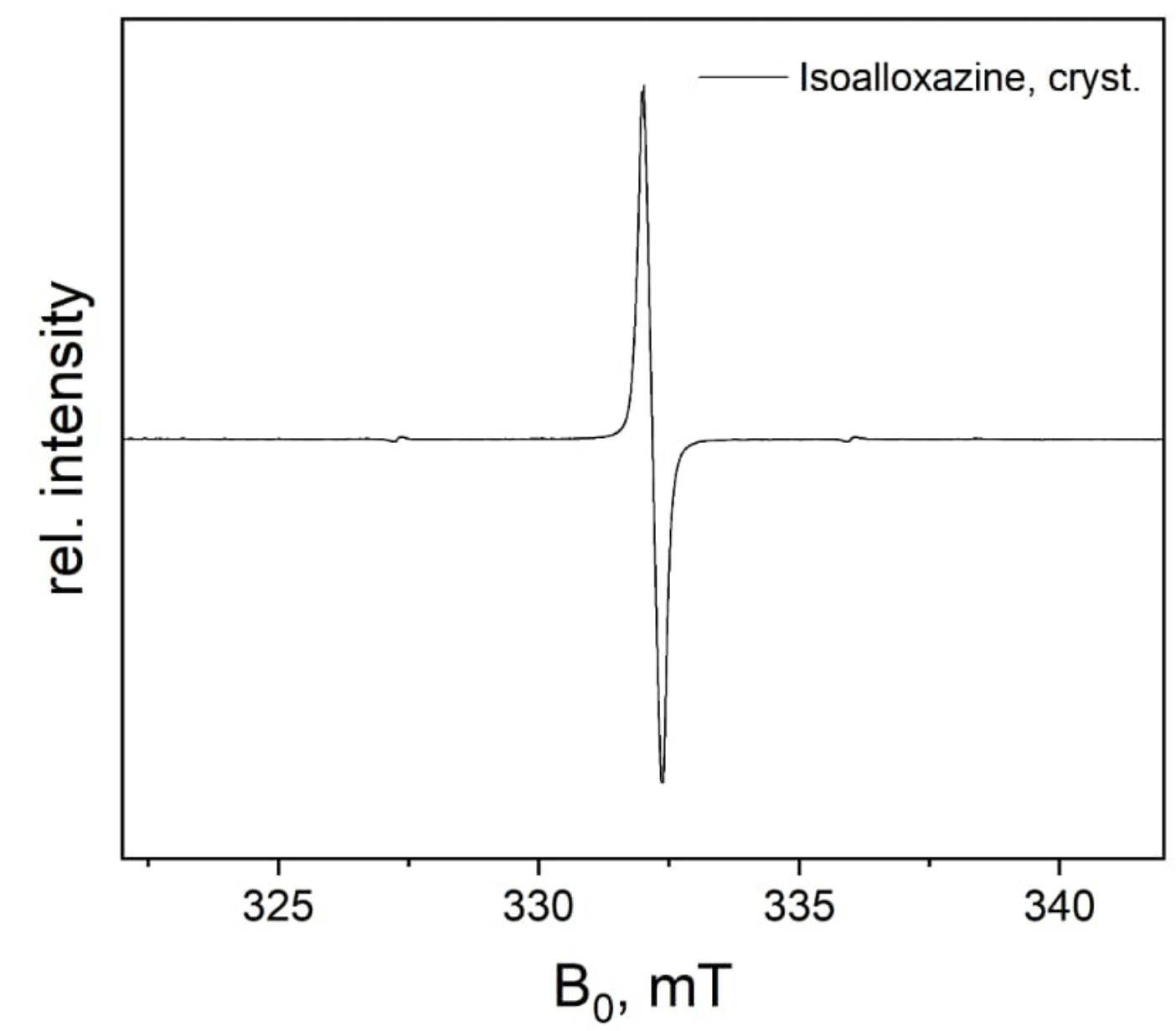

Figure SI-25. Solid state EPR spectrum of isoalloxazine radical **8**.

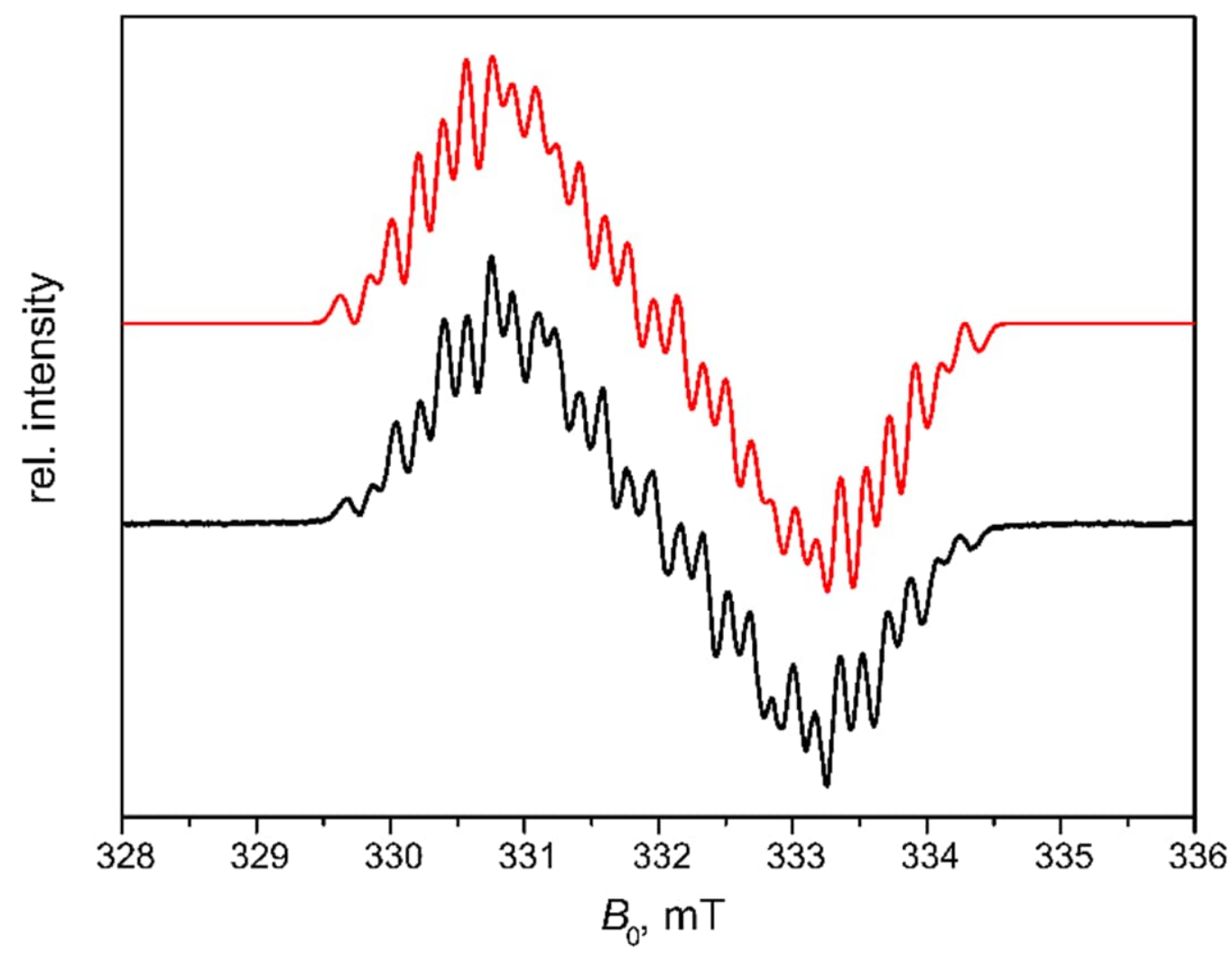

Measurement setup for 10
Modulation 0.4 x 0.1 = 0.04 mT
Receiver gain: 1 x 100
Time constant: 0.1 s
Sweep width: ± 10 mT
Power: 5 mW

Figure SI-26. Stack EPR plot of **10** in $CDCl_3$ under argon (black) and the simulated spectrum (red). A Mn^{2+} standard embedded in MgO-matrix was used to determine the g value of 2.0039 for **10**.

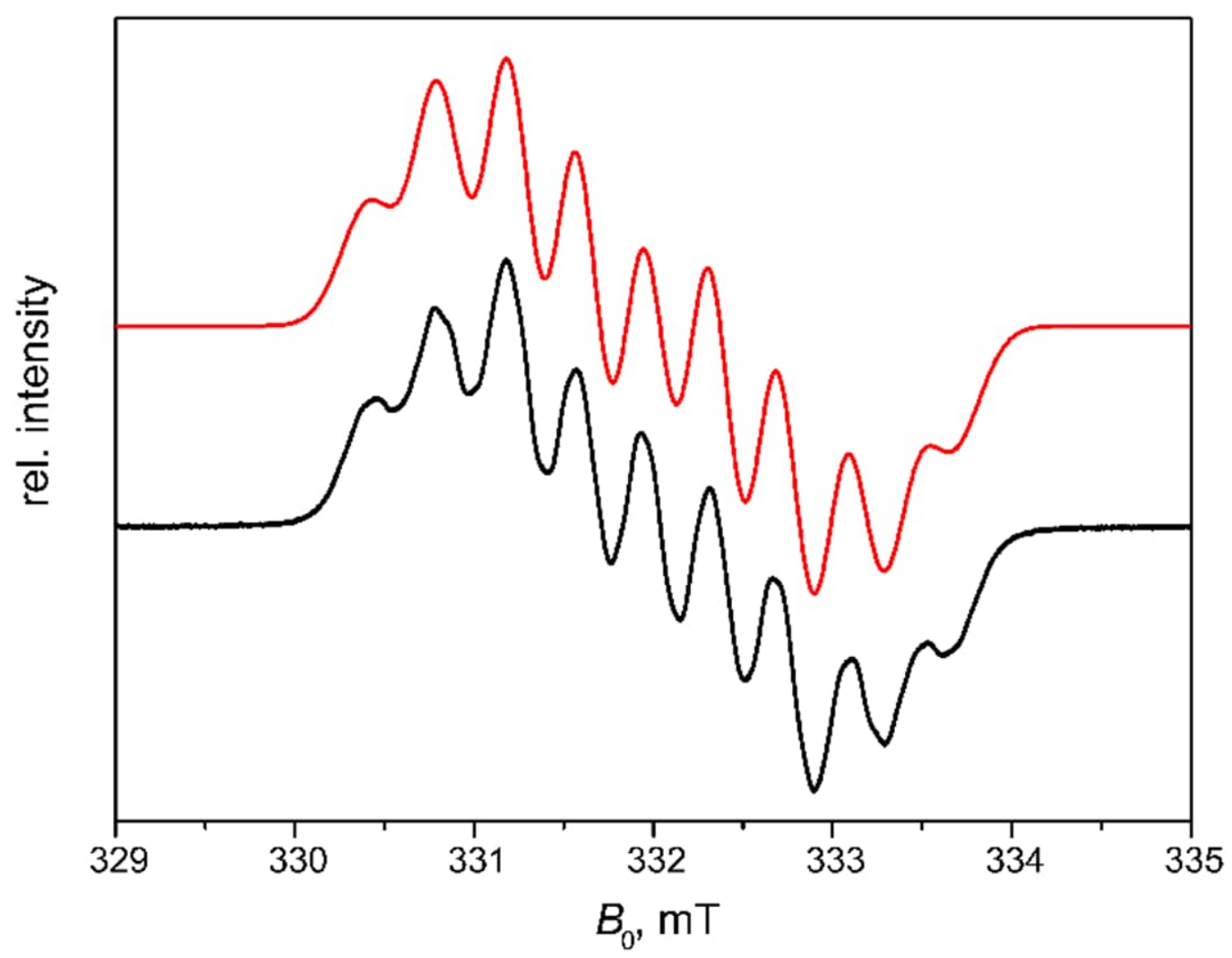

Measurement setup for 8
Modulation 0.4 x 0.1 = 0.04 mT
Receiver gain: 2 x 100
Time constant: 0.1 s
Sweep width: ± 10 mT
Power: 5 mW

Figure SI-27. Stack EPR plot of **8** in $CDCl_3$ under argon and the simulated spectrum (red). A Mn^{2+} standard embedded in MgO-matrix was used to determine the g value of 2.0039 for **8**.

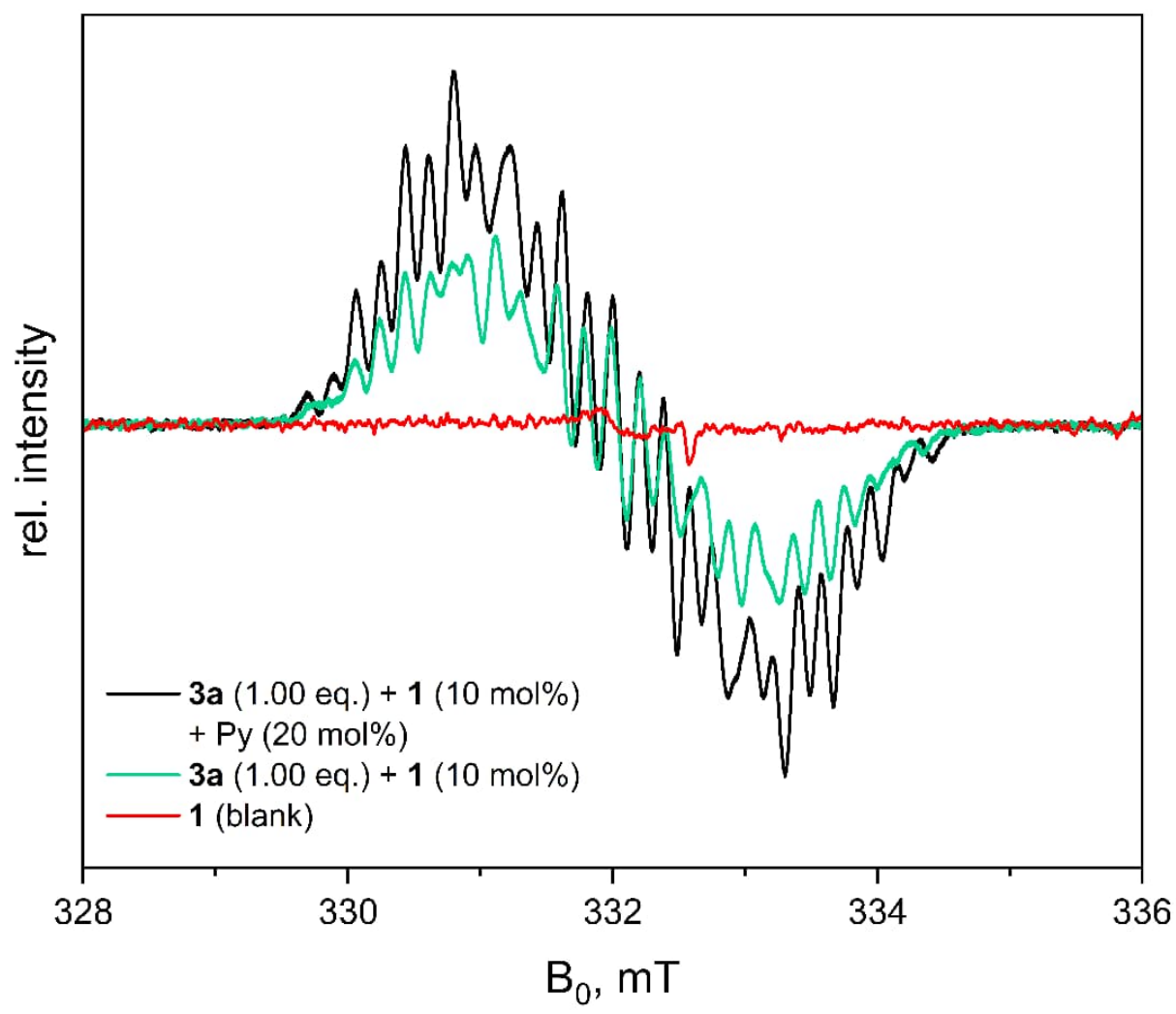

Figure SI-28. Stack EPR plot. *Black*: Tetrahydrocarbazole **3a** (1.00 eq.) + isoalloxazinium perchlorate **1** (10 mol%) with 20 mol% pyridine; *Green:* **3a** (1.00 eq.) + **1** (10 mol%); *Red:* only **1**; spectra were recorded in degassed MeCN under argon at 10^{-4} M at room temperature in a fine melting point capillary that was put in the EPR tube. Each tube was prepared separately.

6 Computational Studies

All DFT optimizations were performed with the Gaussian16, Revision C.01 program package.[25] These computations either used the UB3LYP [26–28] or the UM06-2X [29] hybrid functionals and, where indicated, the D3BJ version of Grimme's dispersion. [30] All structures were optimized with Def2-TZVPP [31,32] and maug-cc-pVTZ [33–36] basis sets. Vibrational harmonic frequencies were computed at the same level and Gibbs free energies refer to 298.15 K and 1 atm pressure employing the ideal gas, rigid rotor, and harmonic oscillator approximation. No imaginary frequencies were observed and the obtained geometries thus classified as minima. The Polarizable Continuum Model (PCM) for acetonitrile was used throughout all computations. [37] The EPR hyperfine coupling constants were obtained from optimized geometries (UB3LYP-D3BJ/Def2TZVP) on the UB3LYP/EPR-III [38] level of theory.

Spin densities were visualized using UCSF ChimeraX (version 1.10.1),[39] developed by the Resource for Biocomputing, Visualization, and Informatics at the University of California, San Francisco, with support from National Institutes of Health R01-GM129325 and the Office of Cyber Infrastructure and Computational Biology, National Institute of Allergy and Infectious Diseases, and the SEQCROW plugin (version 1.9.7).[40,41]

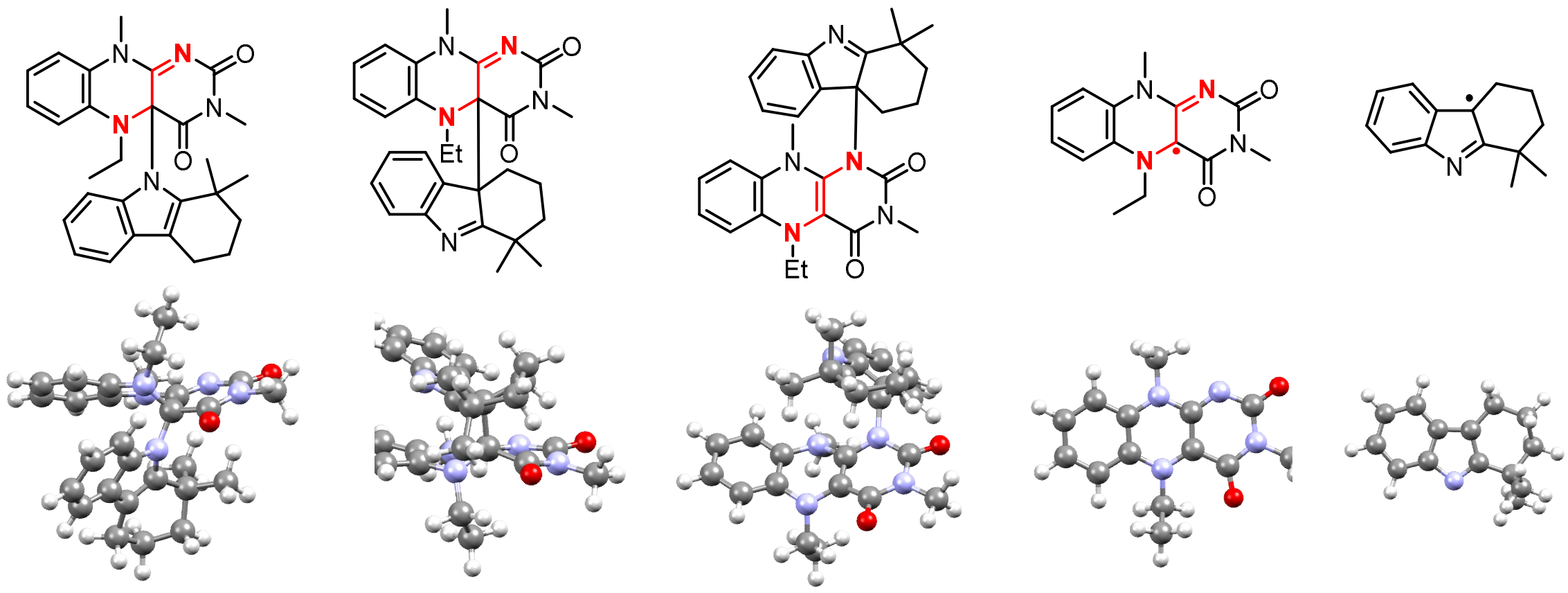

15 **16** **17** **8** **12**

Table SI-3. Summary of all optimized geometries with UB3LYP-D3BJ(PCM:CH_3CN)/Def2TZVPP.

	**15**	**16**	**17**	**8**	**12**	**Σ**
E [Hartree]	-1510.626091	-1510.617329	-1510.613675	-912.44993	-598.150135	
Δ*E* [kJ mol^{-1}]	0.0	23.0	32.6			68.3
Δ*E*+ZPE [kJ mol^{-1}]	0.0	23.5	32.5			55.7
$\Delta G_{298\,K}$ [kJ mol^{-1}]	0.0	29.9	33.3			–8.5

Table SI-4. Summary of all optimized geometries with UB3LYP-D3BJ(PCM:CH_3CN)/maug-cc-pVTZ.

	**15**	**16**	**17**	**8**	**12**	**Σ**
E [Hartree]	-1510.582711	-1510.574127	-1510.570676	-912.423349	-598.134459	
Δ*E* [kJ mol^{-1}]	0.0	22.5	31.6			65.4
Δ*E*+ZPE [kJ mol^{-1}]	0.0	22.9	31.4			52.7
$\Delta G_{298\,K}$ [kJ mol^{-1}]	0.0	29.1	31.9			–11.7

Table SI-5. Summary of all optimized geometries with UM06-2X(PCM:CH_3CN)/Def2TZVPP.

	**15**	**16**	**17**	**8**	**12**	**Σ**
E [Hartree]	-1509.859248	-1509.850717	-1509.847636	-912.004539	-597.819245	
Δ*E* [kJ mol^{-1}]	0.0	22.4	30.5			93.1
Δ*E*+ZPE [kJ mol^{-1}]	0.0	22.2	29.5			80.2
$\Delta G_{298\,K}$ [kJ mol^{-1}]	0.0	27.3	27.7			12.1

Table SI-6. Summary of all optimized geometries with UM06-2X(PCM:CH_3CN)/maug-cc-pVTZ.

	**15**	**16**	**17**	**8**	**12**	Σ
E [Hartree]	-1509.833534	-1509.825275	-1509.822346	-911.989579	-597.809293	
ΔE [kJ mol^{-1}]	0.0	21.7	29.4			91.0
ΔE+ZPE [kJ mol^{-1}]	0.0	21.4	28.2			78.2
$\Delta G_{298\,K}$ [kJ mol^{-1}]	0.0	26.4	24.8			10.6

7 Oxidation Experiments

7.1 NMR Studies on the Aerobic Oxidation of Reduced Flavins

Alloxazine 9

In a glovebox, an NMR sample of reduced alloxazine **9** in CD_3OD was prepared and measured (i). After the first measurement, the NMR tube was opened and air was introduced into the headspace (without bubbling through the solution, as this accelerates oxidation). The tube was then closed, inverted once, and measured again (ii). For measurements (iii)–(vii), the NMR tube was opened each time, additional air was introduced into the headspace, and the tube was shaken vigorously approximately ten times prior to measurement.

Isoalloxazine 5

Under air, an NMR sample of reduced isoalloxazine **5** in CD_3OD was prepared and measured (i). After each measurement, the NMR tube was shaken vigorously approximately ten times prior to measurement. Before measurements (iii) and (iv) the NMR tube was opened and air was introduced into the headspace (without bubbling through the solution, as this accelerates oxidation).

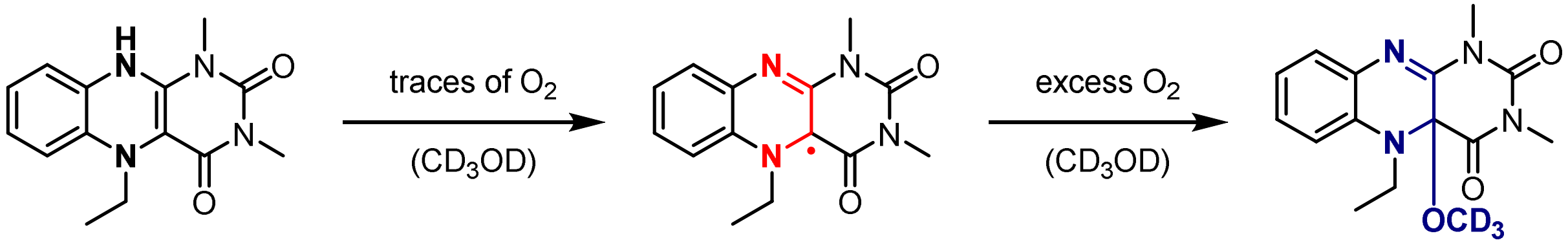

9 10 SI-33

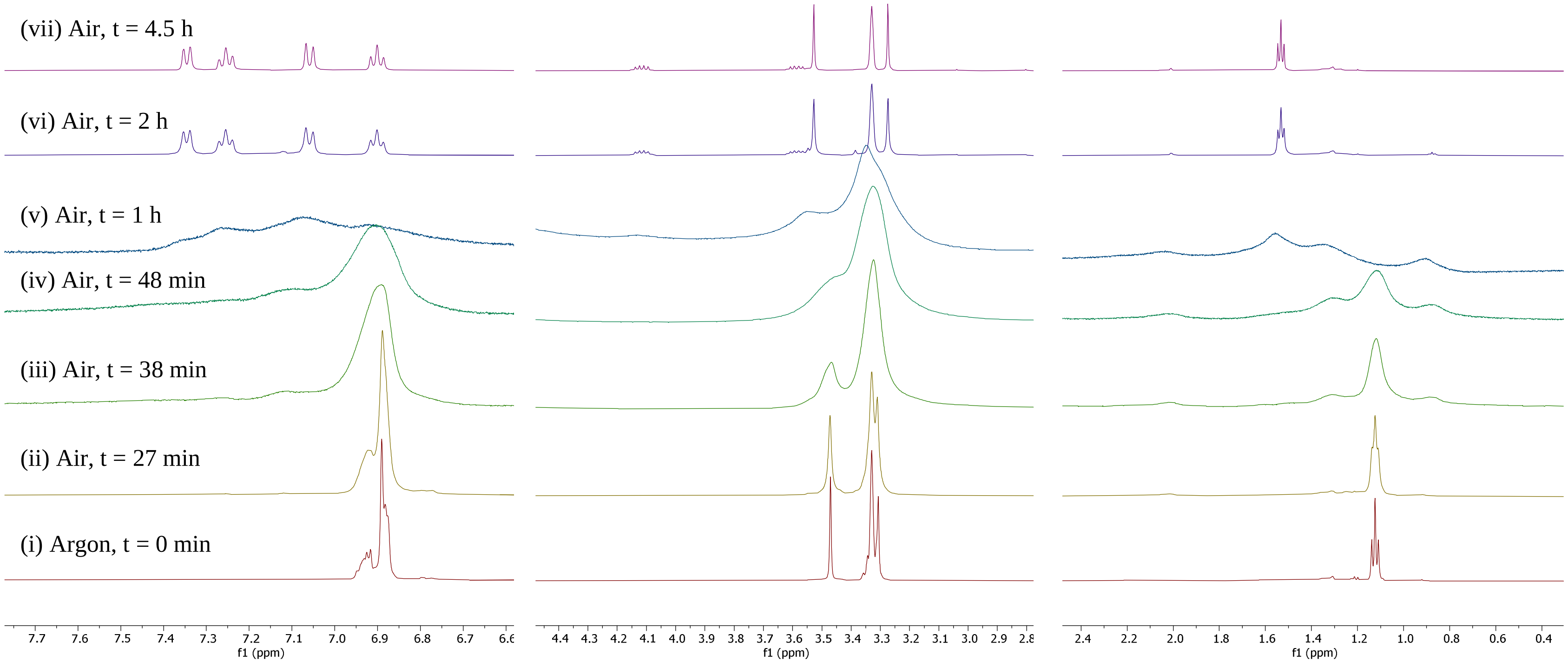

Figure SI-29. NMR reactivity study on reduced alloxazine **9** in CD_3OD upon exposure to air. Stacked NMR spectra show how the signals of **9** (trace i) broadens, likely due to the generation of radical **10** upon oxidation (traces ii – v). Further oxidation leads to the oxidized state, which forms CD_3OD adduct **SI-33** in solution (traces vi – vii). Spectra clipped for clarity.

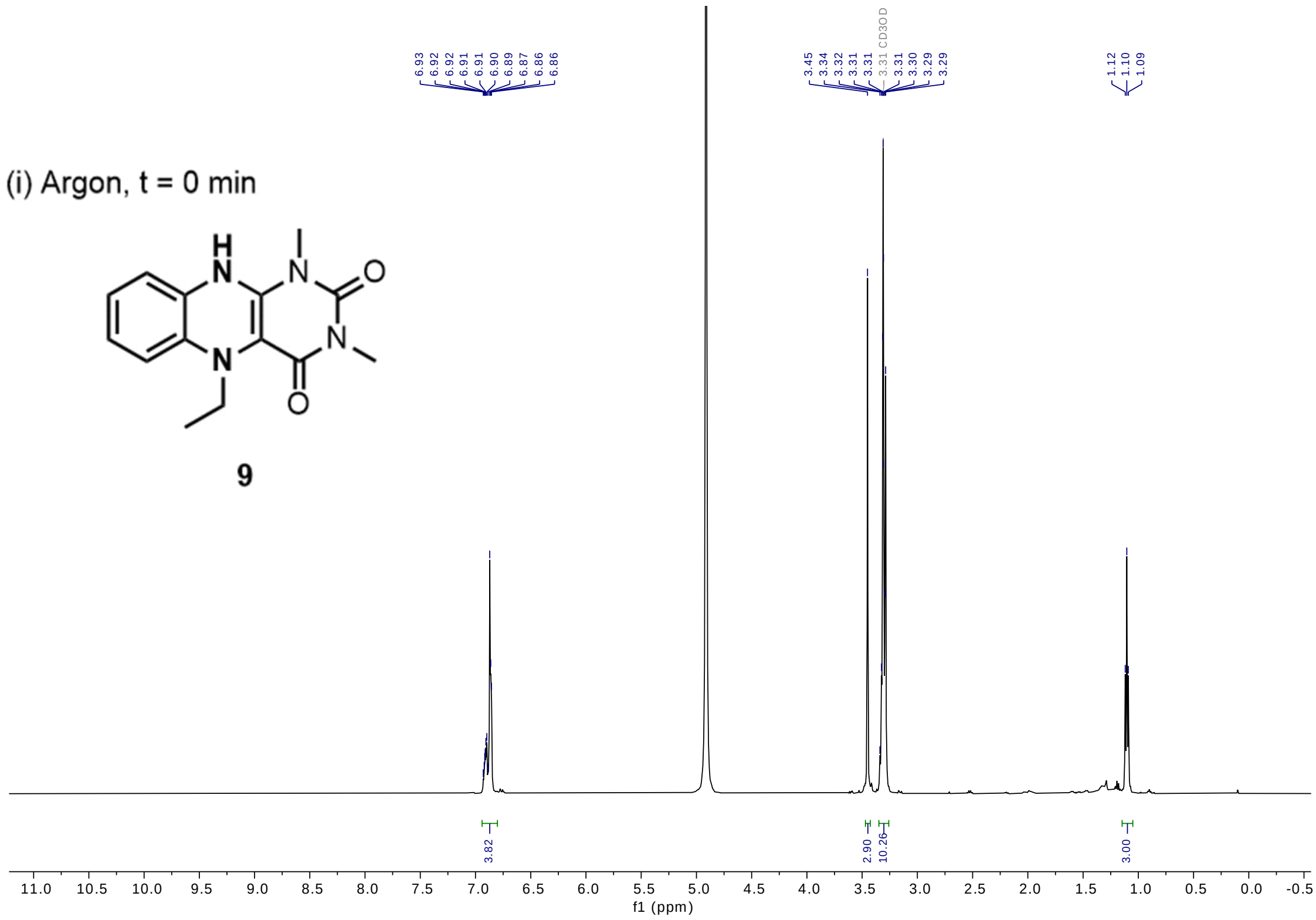
(i) Argon, t = 0 min
9
f1 (ppm)

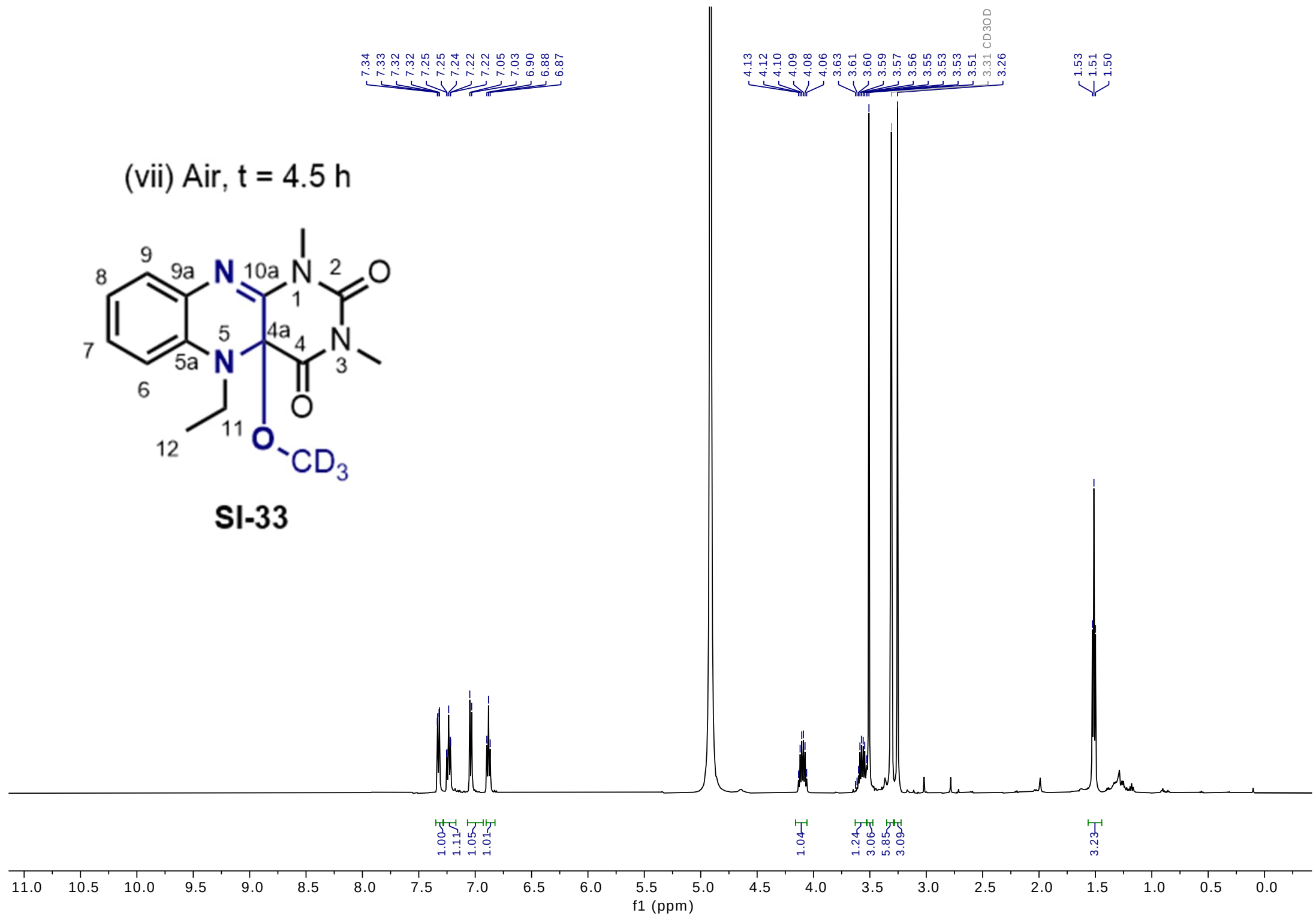
(vii) Air, t = 4.5 h
SI-33
f1 (ppm)

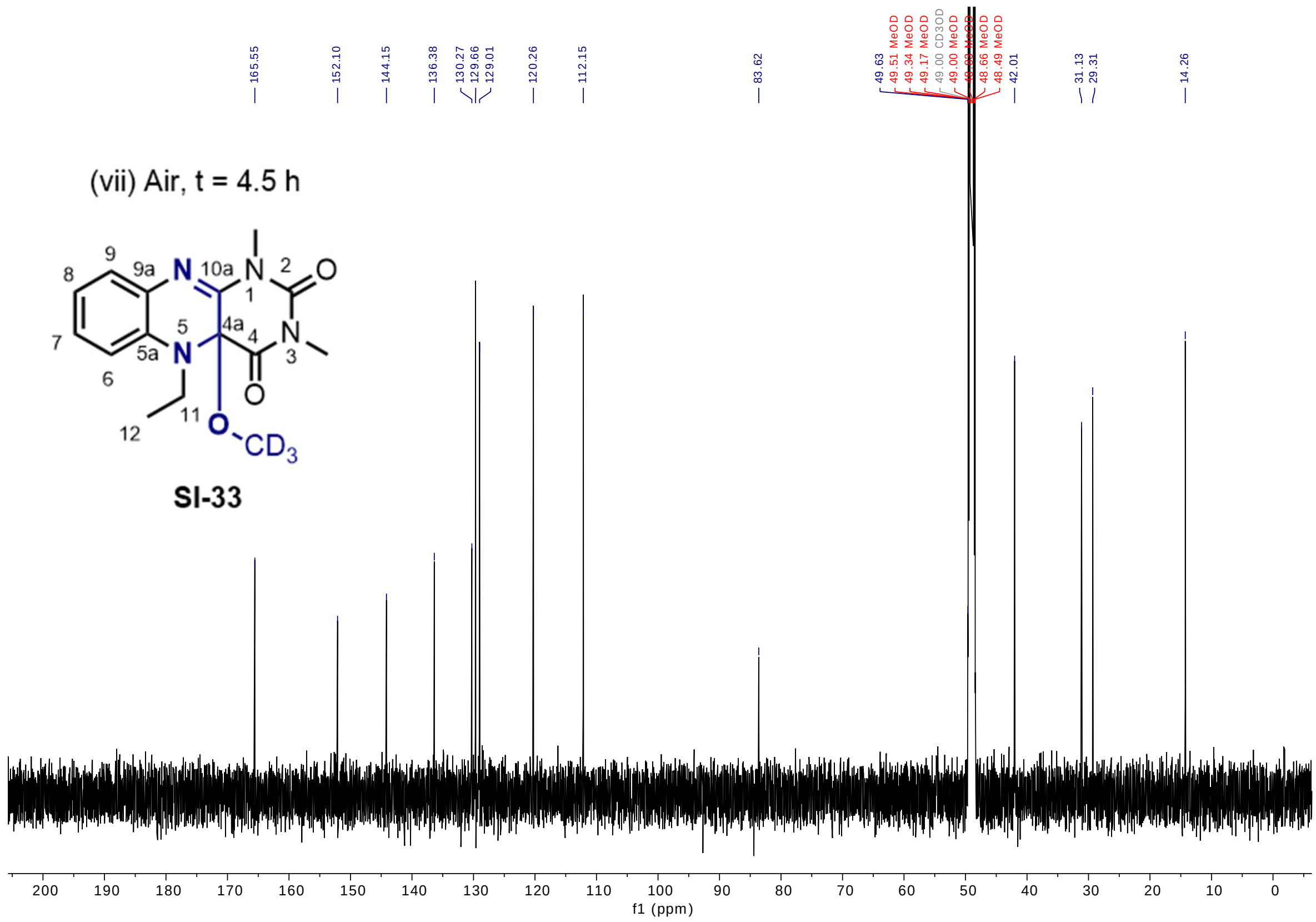

For full spectroscopic analysis of compound **9**, please refer to Experimental Sections 2.2.3.

<u>Compound **SI-33**:</u>

^{1}H NMR (500 MHz, CD_3OD, 298 K): δ (ppm) = 7.33 (dd, $^{3}J_{H\text{-}H}$ = 7.8 Hz, $^{4}J_{H\text{-}H}$ = 1.6 Hz, 1H, H^{9}), 7.28 – 7.20 (m, 1H, H^{7}), 7.06 – 7.02 (m, 1H, H^{6}), 6.90 – 6.85 (m, 1H, H^{8}), 4.10 (dq, $^{2}J_{H\text{-}H}$ = 14.4 Hz, $^{3}J_{H\text{-}H}$ = 7.0 Hz, 1H, H^{11}), 3.61 – 3.51 (m, 1H, H^{11}), 3.51 (s, 3H, $N^{1}CH_3$), 3.26 (s, 3H, $N^{3}CH_3$), 1.51 (t, $^{3}J_{H\text{-}H}$ = 6.9 Hz, 3H, H^{12}); **^{13}C{^{1}H} NMR** (126 MHz, CD_3OD, 298 K): δ (ppm) = 165.6 (C^{4}), 152.1 (C^{2}), 144.2 (C^{10a}), 136.4 (C^{5a}), 130.3 (C^{6a}), 129.7 (C^{7}), 129.0 (C^{9}), 120.3 (C^{8}), 112.2 (C^{6}), 83.6 (C^{4a}), 42.0 (C^{11}), 31.1 ($N^{1}CH_3$), 29.3 ($N^{3}CH_3$), 14.3 (C^{12}). The carbon signal of the O-CD_3 group is likely overlapped with the solvent residual signal.

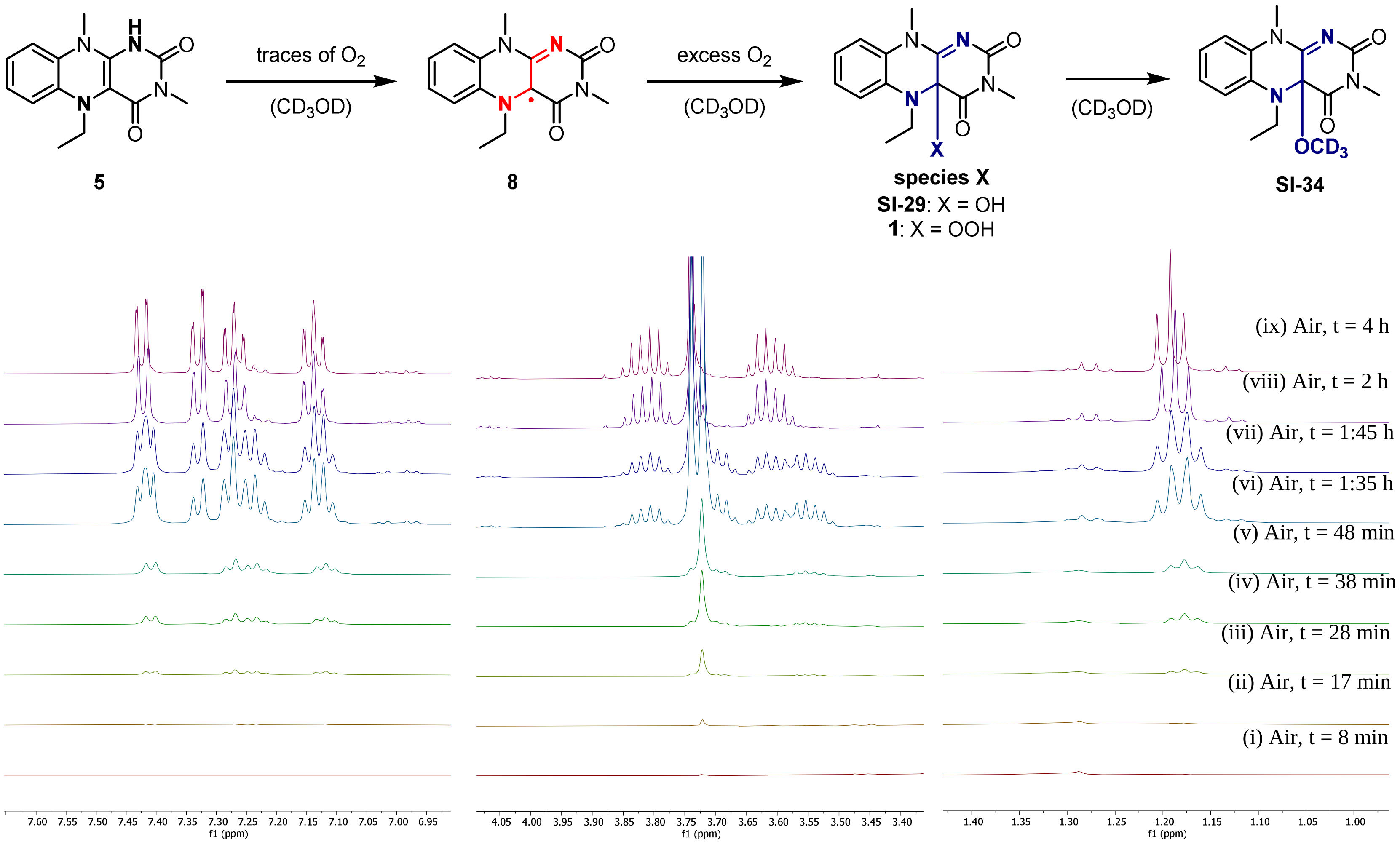

Figure SI-30. NMR reactivity study on reduced isoalloxazine **5** in CD_3OD upon exposure to air. Stacked NMR spectra exhibit no resonances of **5** in trace (i), as this spectrum was recorded 8 min after dissolving **5** under air. This may indicate that paramagnetic species **8** already formed. For full spectroscopic analysis of compound **5**, please refer to Experimental Sections 2.2.17. With time (spectra iii - v), an unidentified species **X** forms, that decomposes fully into the fully characterized CD_3OD adduct **SI-34** (traces vi – ix). Spectra clipped for clarity.

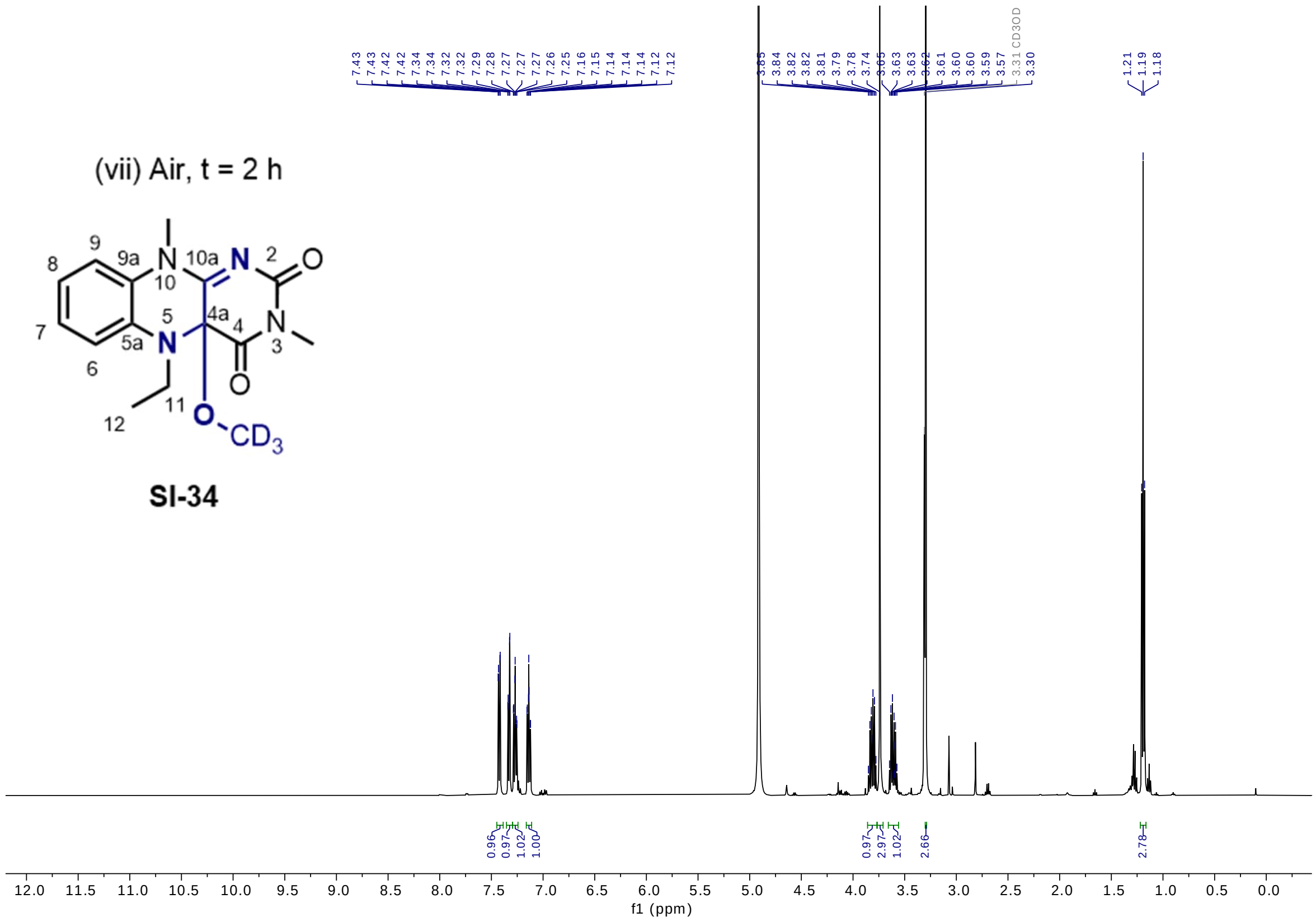

(vii) Air, t = 2 h
SI-34
7.43
7.43
7.42
7.42
7.34
7.34
7.32
7.32
7.29
7.28
7.27
7.27
7.27
7.26
7.25
7.16
7.15
7.14
7.14
7.14
7.12
7.12
3.85
3.84
3.82
3.82
3.81
3.79
3.78
3.74
3.65
3.63
3.63
3.62
3.61
3.60
3.60
3.59
3.57
3.31 CD3OD
3.30
1.21
1.19
1.18
0.96
0.97
1.02
1.00
0.97
2.97
1.02
2.66
2.78
f1 (ppm)

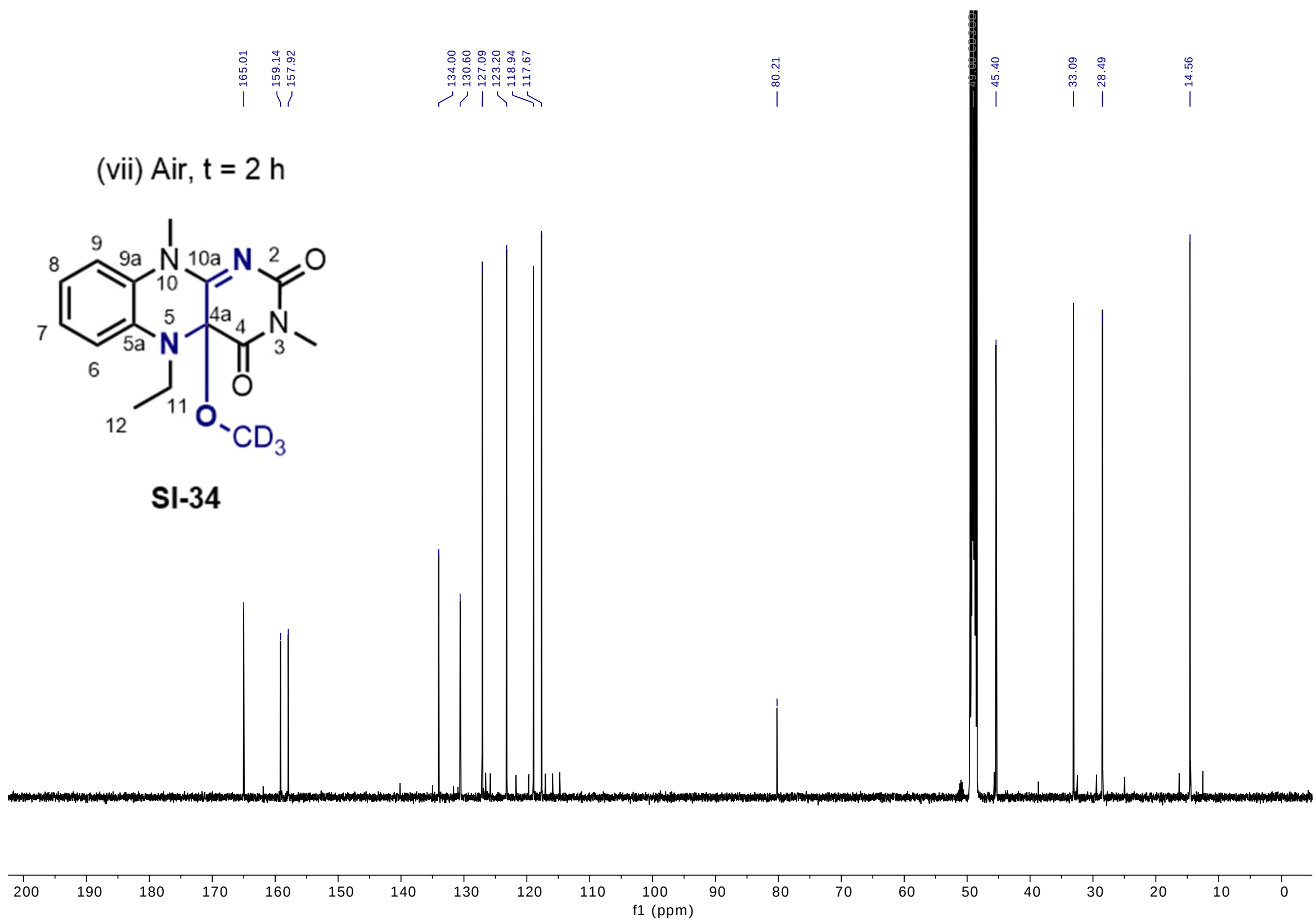

165.01
159.14
157.92
134.00
130.60
127.09
123.20
118.94
117.67
80.21
45.40
33.09
28.49
14.56
(vii) Air, t = 2 h
SI-34
f1 (ppm)

Compound **SI-34**:

^{1}H NMR (500 MHz, CD_3OD, 298 K): δ (ppm) = 7.42 (dd, $^{3}J_{H\text{-}H}$ = 8.2 Hz, $^{4}J_{H\text{-}H}$ = 1.3 Hz, 1H, H^{9}), 7.33 (dd, $^{3}J_{H\text{-}H}$ = 8.2 Hz, $^{4}J_{H\text{-}H}$ = 1.5 Hz, 1H, H^{6}), 7.27 (ddd, $^{3}J_{H\text{-}H}$ = 8.2, 7.3 Hz, $^{4}J_{H\text{-}H}$ = 1.3 Hz, 1H, H^{7}), 7.14 (ddd, $^{3}J_{H\text{-}H}$ = 8.2, 7.3 Hz, $^{4}J_{H\text{-}H}$ = 1.5 Hz, 1H, H^{8}), 3.86 – 3.76 (m, 1H, H^{11}), 3.74 (s, 3H, N^{10}-CH_3), 3.61 (dq, $^{2}J_{H\text{-}H}$ = 13.9 Hz, $^{3}J_{H\text{-}H}$ = 7.0 Hz, 1H, H^{11}), 3.30 (s, 3H, N^{3}-CH_3), 1.19 (t, $^{3}J_{H\text{-}H}$ = 7.0 Hz, 3H, H^{12}); **^{13}C{^{1}H} NMR** (126 MHz, CD_3OD, 298 K): δ (ppm) = 165.0 (C^{4}), 159.1 (C^{10a}), 157.9 (C^{2}), 134.0 (C^{5a}), 130.6 (C^{9a}), 127.1 (C^{7}), 123.2 (C^{8}), 118.9 (C^{6}), 117.7 (C^{9}), 80.2 (C^{4a}), 45.4 (C^{11}), 33.1 (N^{10}-CH_3), 28.5 (N^{3}-CH_3), 14.6 (C^{12}). The carbon signal of the O-CD_3 group is likely overlapped with the solvent residual signal.

7.2 Microscopy

The oxidation of reduced alloxazine **9** and the crystallization of semiquinone **10** were monitored by optical microscopy. A saturated solution of **9** in degassed MeOH was prepared under *Schlenk* conditions. The solution was then transferred to a 0.5 mm cuvette, and a small amount of air was introduced (start of the video). The yellow solution immediately darkened to red, and after approximately 4 min, dark needle-shaped crystals of semiquinone **10** started forming. Further exposure to air led to the disappearance of these crystals, yielding a yellow solution from which methoxide adduct crystals of **11** formed over several days (not shown in the recording). A full-length video is provided in the Supporting Information online for download.

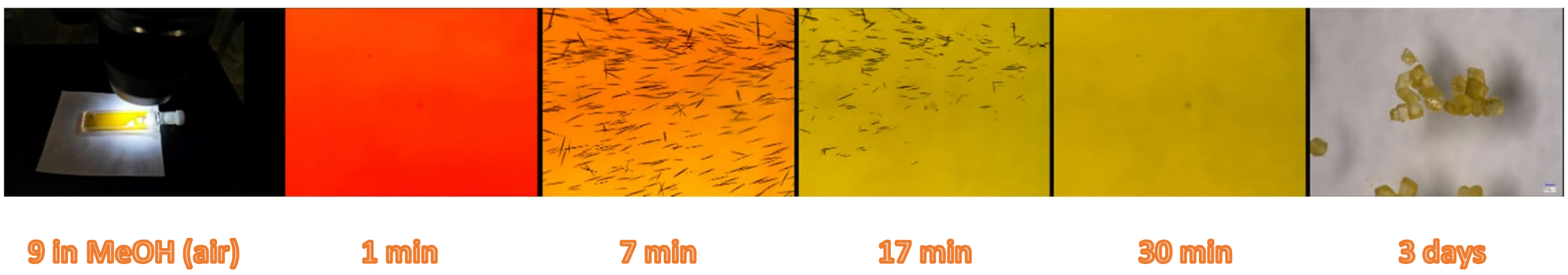

Figure SI-31. Experimental setup for recording the crystal growth of radical **10** (left), video screenshots (middle four pictures) of the appearance and disappearance of **10**, and grown methoxide adduct crystals **11** (right). The crystal growth of isoalloxazine semiquinone **8** can be achieved analogously starting from reduced isoalloxazine **5**; however, it was found to be difficult to record visually due to rapid and extensive darkening of the solution upon exposure to air, which hinders monitoring of the process by optical microscopy.

7.3 UV/Vis Study of Alloxazine Semiquinone Radical 10 under Air

A solution of isolated semiquinone **10** in CH_3CN was transferred to a *Schlenk* cuvette under argon (no grease applied; sealed with a Teflon stopper ring) and measured immediately. The cuvette was subsequently kept sealed outside the glovebox to allow slow diffusion of air into the system, and spectra were recorded again after 19 and 24 h, respectively. Alloxazinium perchlorate was plotted as a reference. All spectra were recorded at a concentration of 0.368 mM in degassed CH_3CN.

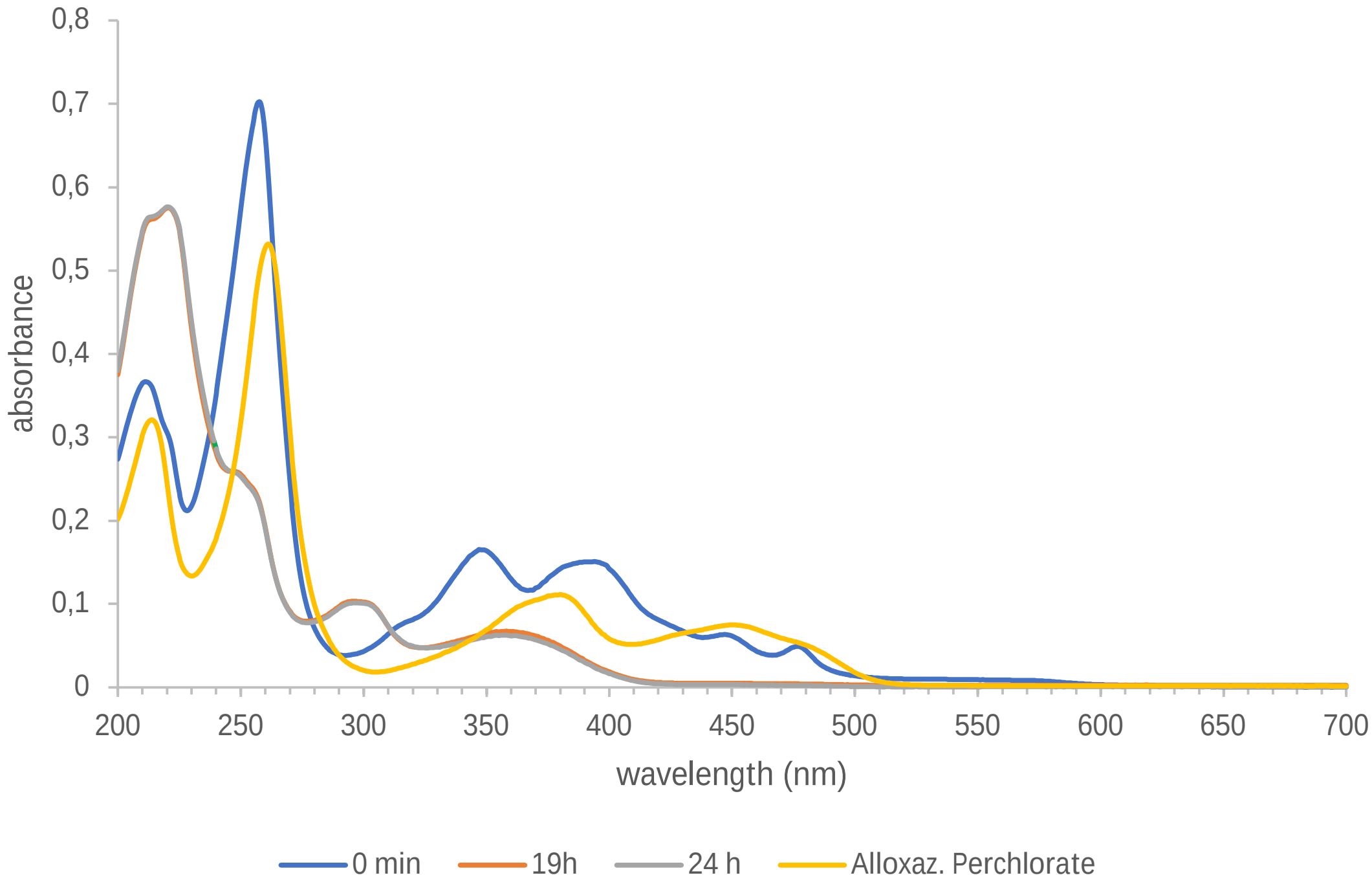

Figure SI-32. UV/Vis stack plot of semiquinone **10** in MeCN with slow oxygen exposure over time (blue = 0 min; orange = 19h; grey = 24h). Alloxazinium perchlorate **7a** plotted for reference (yellow).

In contrast to a solution stored inside the glovebox, which remained intact on the following day (see Section 4), the sample kept outside the glovebox gradually decomposed. This observation further supports that semiquinone **10** undergoes decomposition in solution under elevated oxygen exposure. **10** is therefore stable in CH_3CN under strict exclusion of oxygen, but decomposes in its presence.

8 X-Ray Crystallographic Data

Sample Preparation

Single crystals of compound **1** were obtained by slow diffusion (layering in NMR tube) of diethyl ether into an acetonitrile solution of the title compound over two weeks at ambient temperature.

Hydroperoxide **2** was crystallized as described in experimental section 2.2.19 by slow precipitation of a saturated acetonitrile solution of **1** in aqueous H_2O_2 containing phosphate buffer.

Compound **4a** crystallized after a pure fraction from column chromatography (heptane/EtOAc) was collected in a test tube and gently evaporated under a nitrogen stream. Slow evaporation of the solvent afforded crystals suitable for X-ray analysis.

Semiquinone radical crystals of **8**, **10**, and **10b** were obtained by subjecting saturated, degassed methanol solutions of their respective reduced forms, i.e. compounds **5**, **9**, and **9b** to trace amounts of oxygen, as described in section 2.2.5 or 2.2.18.

Single crystals of methanol adduct **11** were grown after leaving a methanol solution of reduced alloxazine **9** on air for three days, as decribed in section 2.2.6.

Single crystals of compound **21** were obtained by slow diffusion (layering in NMR tube) of *n*-pentane into an acetone solution of the title compound over two weeks at ambient temperature.

Compound **22** was crystallized in a GC vial with a septum cap by dissolving it in acetone and adding water dropwise until precipitation commenced. A small amount of acetone was then added to redissolve the solid, yielding a clear solution. The vial was pierced with a needle, and slow evaporation overnight afforded single crystals of the title compound.

Measurement

SC-XRD data were collected on a Bruker D8 Venture single crystal X-ray diffractometer equipped with a Bruker Photon CMOS detector, a TXS rotating anode with MoKα radiation (λ = 0.71073 Å), and a Helios optic using the APEX4 software package.[42] Measurements were performed on single crystals coated with perfluorinated ether. The crystals were fixed on top of a Kapton micro

sampler and frozen under a stream of cold nitrogen. A matrix scan was used to determine the initial lattice parameters. All data were integrated by SAINT using a narrow-frame algorithm and the reflections were corrected for Lorentz and polarisation effects, scan speed, and background.[43] Data were corrected for absorption effects including odd and even ordered spherical harmonics by the multi-scan method SADABS.[44] Space group assignment was based upon systematic absences, E statistics, and successful refinement of the structure.

Structure Solution/Refinement

The structures were solved by direct methods using SHELXT and refined by full-matrix least-squares methods against F^2 by minimizing $\Sigma w(Fo^2 - Fc^2)^2$ using SHELXL in conjunction with SHELXLE.[45–47] All non-hydrogen atoms were refined with anisotropic displacement parameters. Hydrogen atoms were refined isotropically on calculated positions using a riding model with their U_{iso} values constrained to 1.5 times the U_{eq} of their pivot atoms for terminal sp^3 carbon atoms and a C–H distance of 0.98 Å. Non-methyl hydrogen atoms were refined using a riding model with methylene, aromatic, and other C–H distances of 0.99 Å, 0.95 Å, and 1.00 Å, respectively, and U_{iso} values constrained to 1.2 times the U_{eq} of their pivot atoms.

Disorder Treatment and Twinning

Compounds **8** and **10** crystallize in the space groups $P2_1/m$ and *Pnma*, respectively. In both cases, the molecules are situated on a crystallographic mirror plane (*m*). While the molecules are largely coplanar with this symmetry element, specific groups of atoms deviate from ideal planarity. This results in a 50:50 disorder across the mirror plane, which was modeled using the PART-1 instruction in SHELXL with a fixed occupancy of 0.50. Since the diffraction data represent a spatial and temporal average of all conformers within the unit cell, the connectivity of the disordered atoms cannot be determined unambiguously. Specifically, it is impossible to distinguish between a conformer where all disordered atoms reside on the same side of the mirror plane and one where they alternate across it. Both models fit the experimental data equally well. Consequently, the meaningful determination of bond lengths and angles involving these positions require *a priori* knowledge.

To resolve this ambiguity, the *N*-butyl substituted derivative of **10** was synthesized and crystallized. This derivative crystallizes in the space group $P\bar{1}$, which lacks the symmetry constraints of a mirror plane. Although positional disorder is present in the $P\bar{1}$ structure, it is not symmetry-restricted and was successfully refined using free variables in conjunction with SADI and SIMU restraints. This yielded a high-quality structural model which serves as a reliable geometric reference for compounds **8** and **10**. The validity of this structural assignment was further corroborated by DFT calculations on the geometry of compound **8** (*vide supra*), which show excellent agreement with the experimental bond lengths (root-mean-square deviation RMSD: 0.16 Å, maximum deviation 0.29 Å).

Moreover, compound **4a** was treated as a twin with the twin law (–1 0 0, 0 –1 0, 0 0 1), representing a twofold rotation about the *c*-axis. The refined BASF parameter was 0.15. Due to this low volume fraction (15%) of the minor twin domain, twinning was only accounted for in the refinement stage (using HKLF 4 format) rather than during data collection, integration and processing.

Miscellaneous

Neutral atom scattering factors for all atoms and anomalous dispersion corrections for the non-hydrogen atoms were taken from International Tables for Crystallography.[48] Images of the crystal structures were generated with Mercury.[49,50] CCDC numbers 2532560 (compound **1**), 2532557 (compound **2**), 2532561 (compound **4a**), 2532558 (compound **8**), 2532556 (compound **10**), 2532554 (compound **10b**), 2532559 (compound **11**), 2532555 (compound **21**), 2532562 (compound **22**), contain the supplementary crystallographic data for all included structures. These data are provided free of charge by the joint Access Structures service of the Cambridge Crystallographic Data Centre and the Fachinformationszentrum Karlsruhe (www.ccdc.cam.ac.uk/structures).

Supplementary SC-XRD Figures

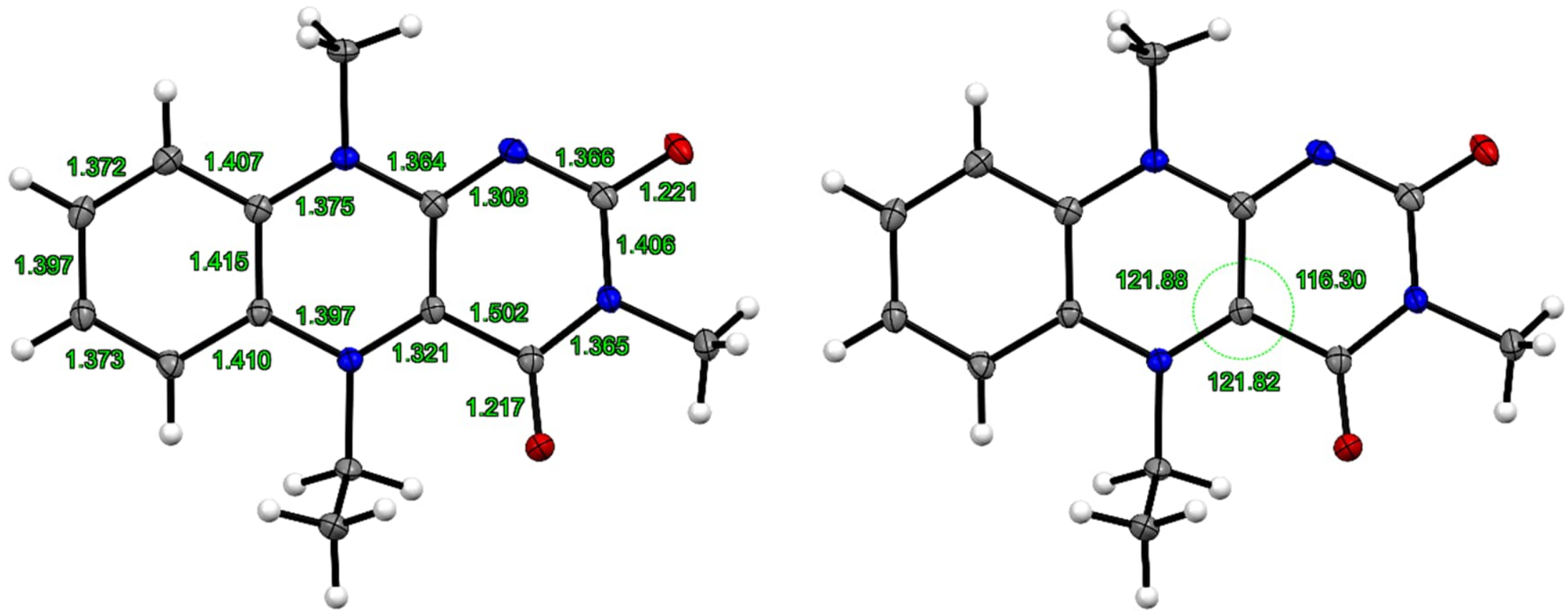

Figure SI-33. Molecular structure of compound **1** (*left*: selected bond lengths [Å], *right*: bond angles [°] around C4a).

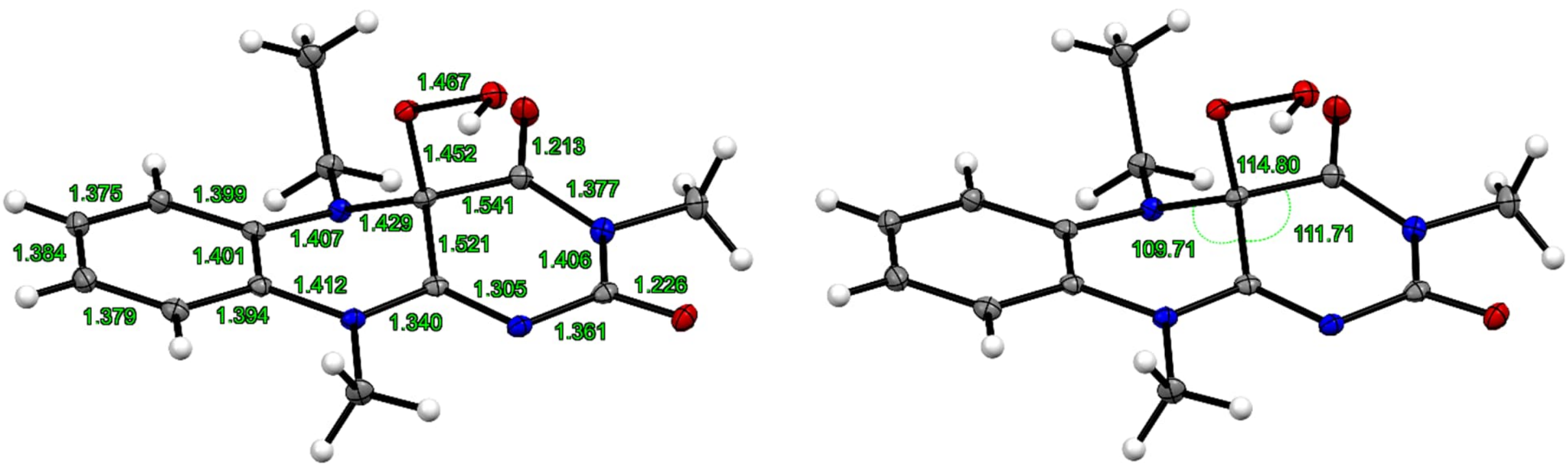

Figure SI-34. Molecular structure of compound **2** (*left*: selected bond lengths [Å], *right*: bond angles [°] around C4a).

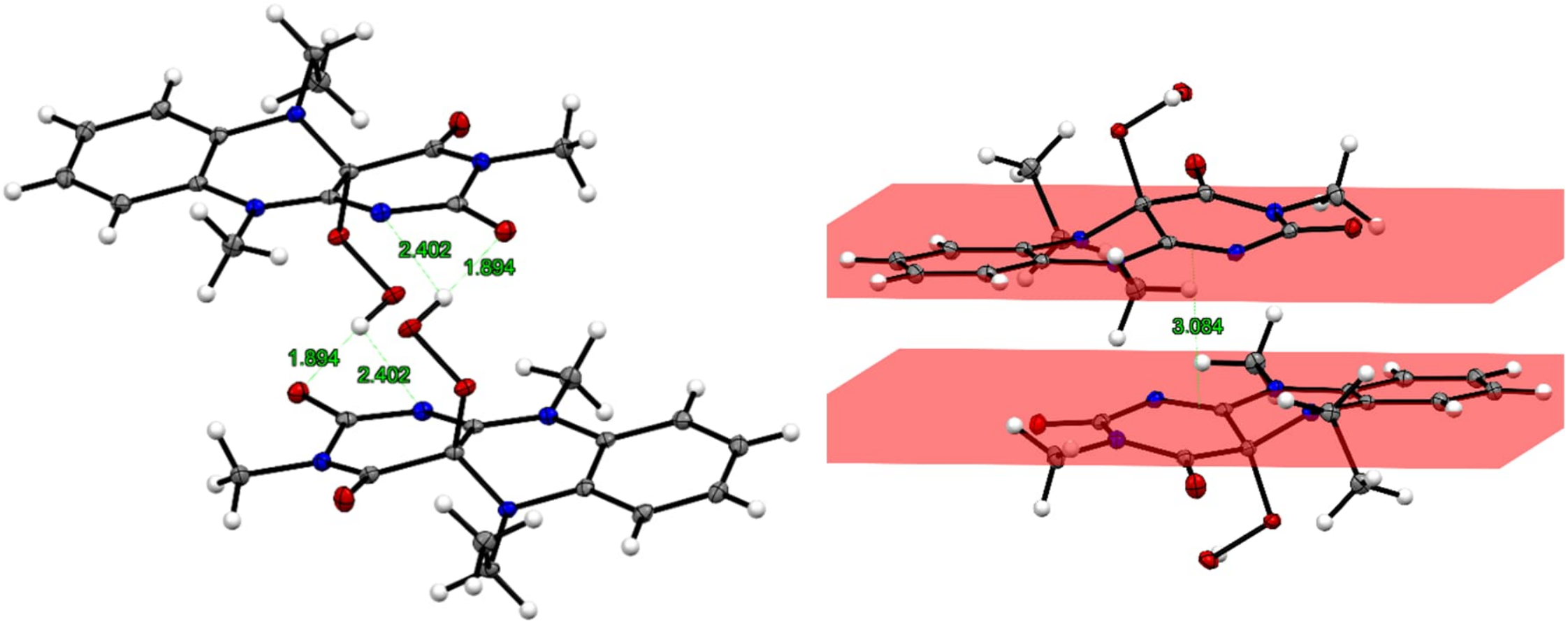

Figure SI-35. Stacking of two adjacent flavins in the crystal structure of compound **2** (*left*: intermolecular hydrogen bonding [Å], *right*: selected interplanar distance [Å]).

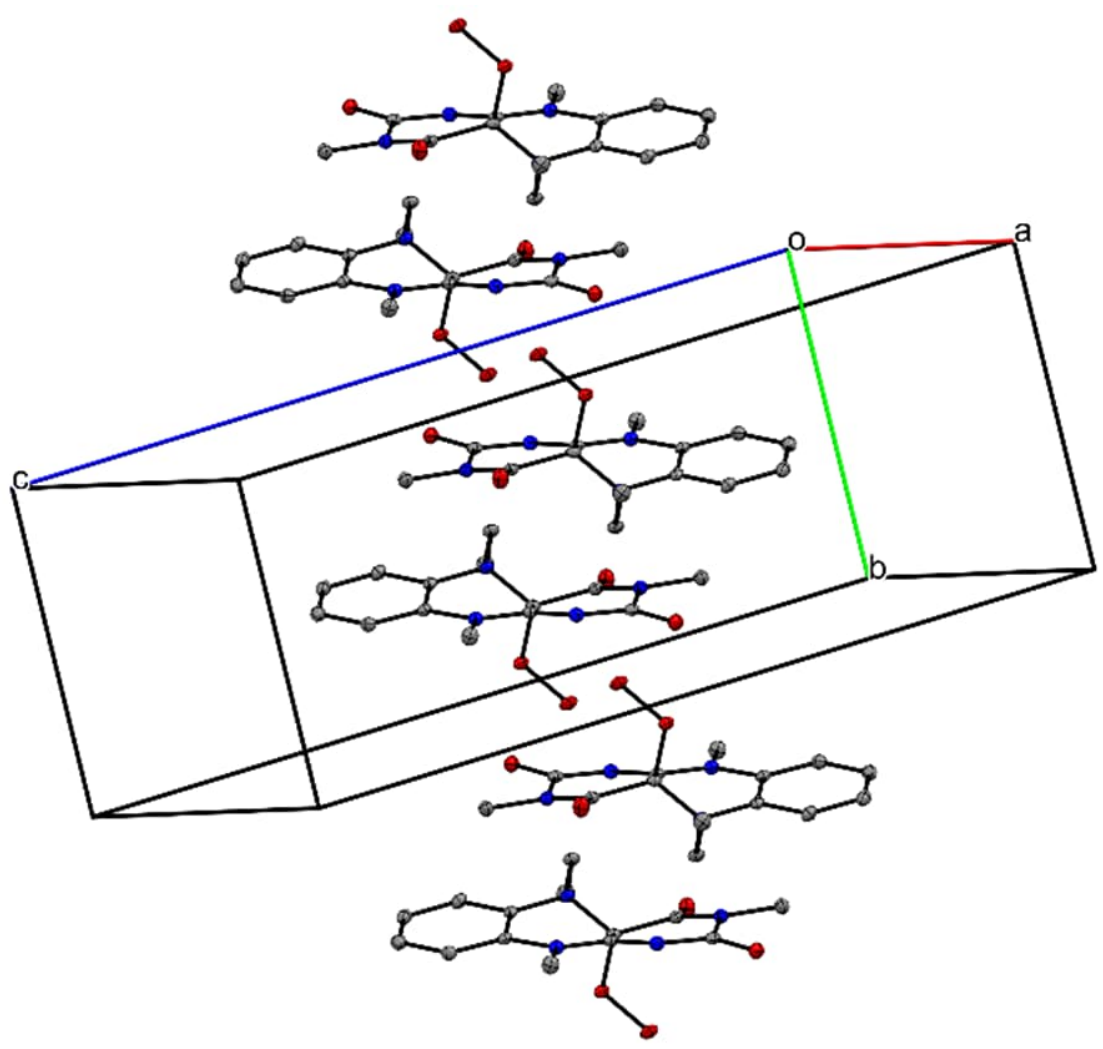

Figure SI-36. Columnar stacking of adjacent flavins in the crystal structure of compound **2** (hydrogen atoms omitted for clarity).

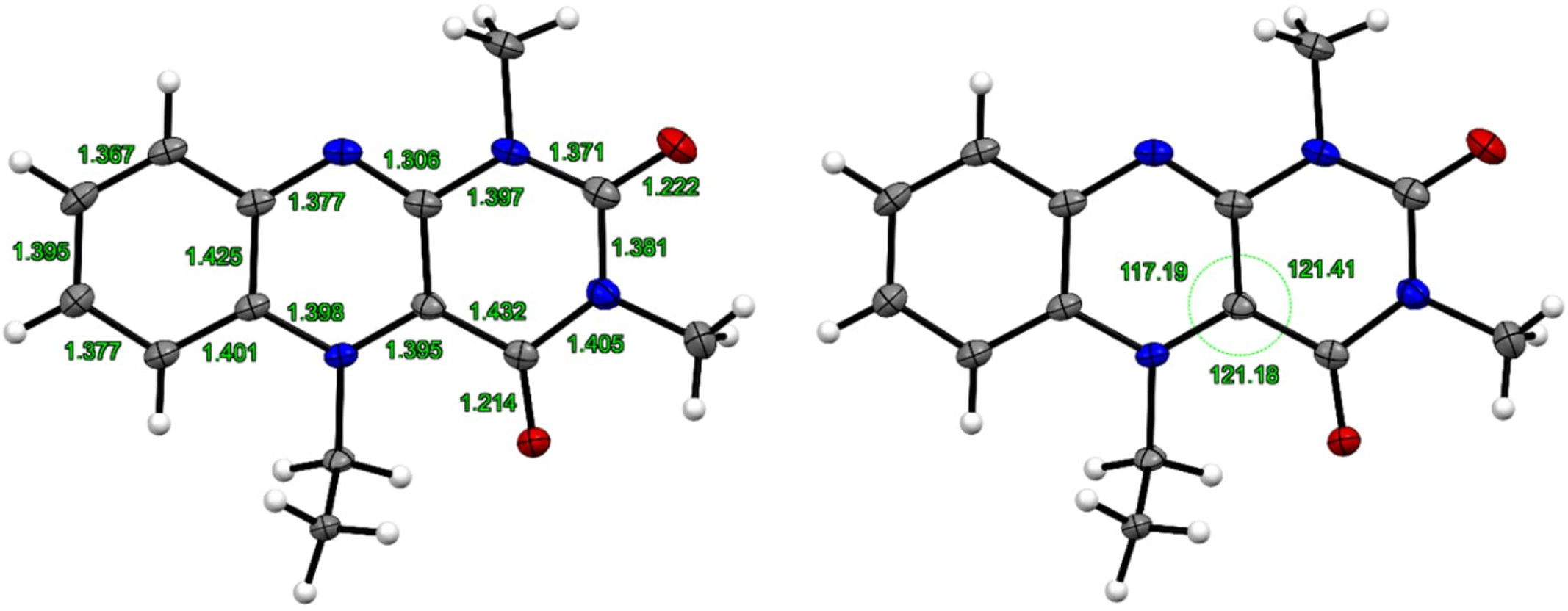

Figure SI-37. Molecular structure of compound **10** (*left*: selected bond lengths [Å], *right*: bond angles [°] around C4a).

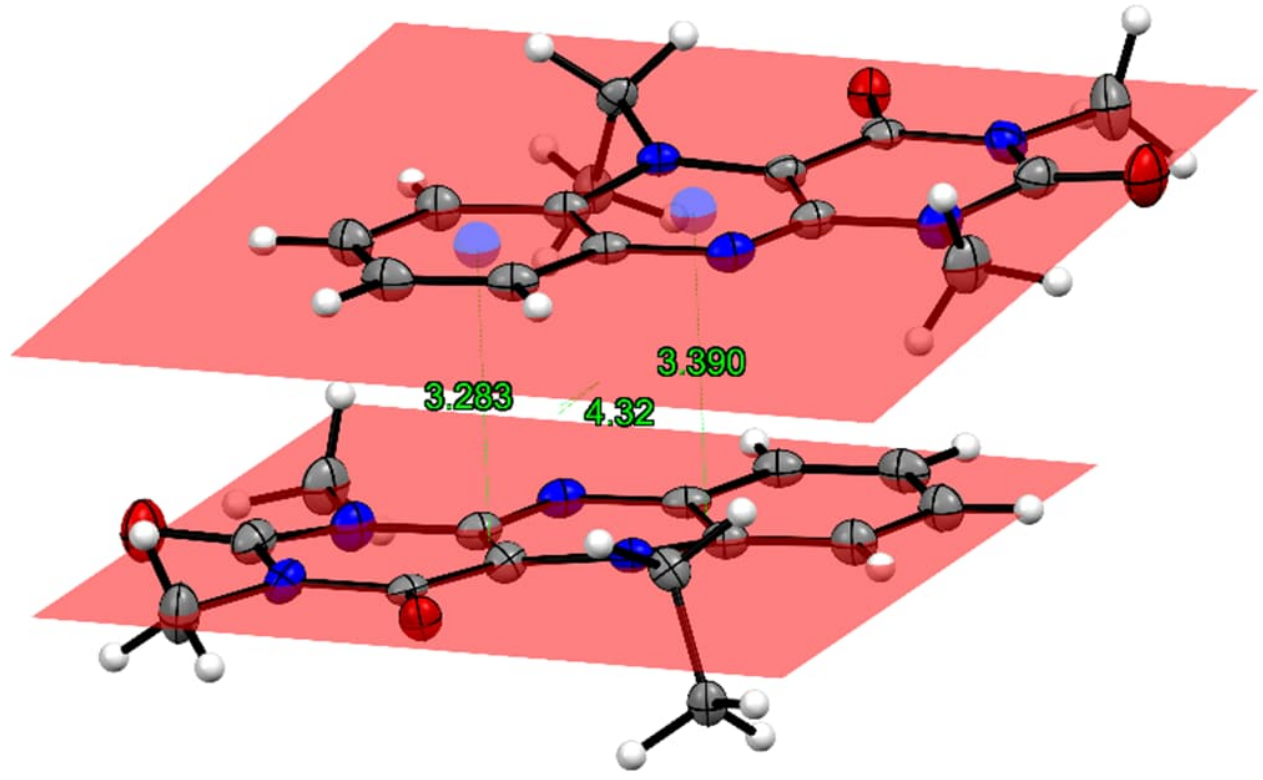

Figure SI-38. Stacking of two adjacent flavins in the crystal structure of compound **10** (selected centroid–plane distances [Å]).

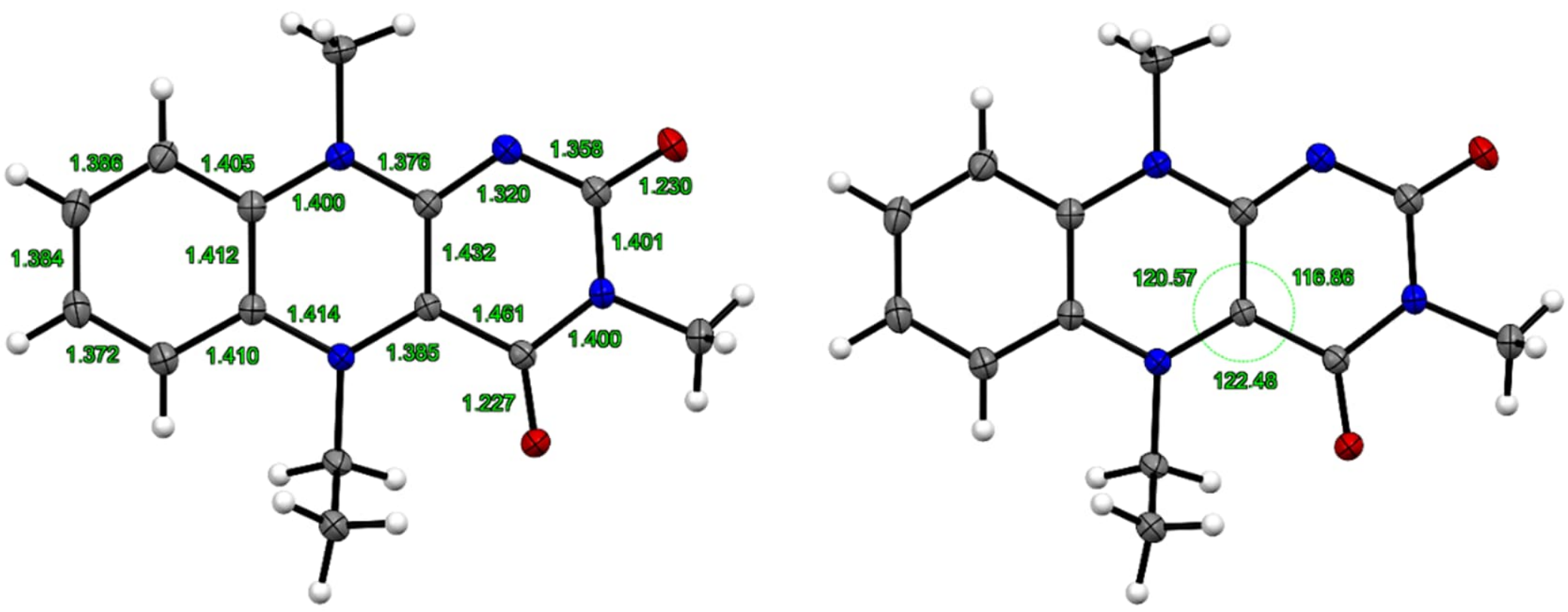

Figure SI-39. Molecular structure of compound **8** (*left*: selected bond lengths, *right*: bond angle around C4a).

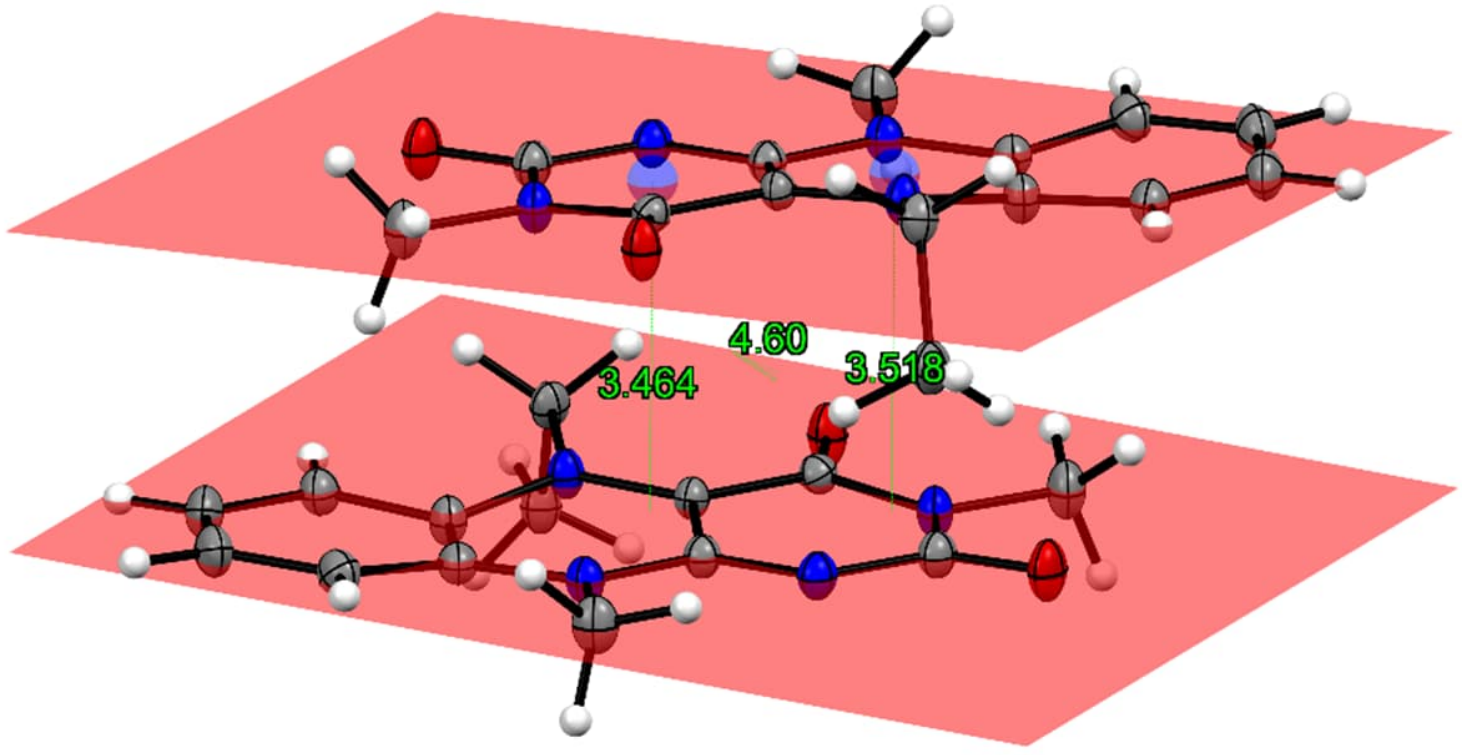

Figure SI-40. Stacking of two adjacent flavins in the crystal structure of compound **8** (selected centroid–plane distances [Å]).

Table SI-8. Crystal data and structure refinement for compound **1**.

CCDC number	2532560
Empirical formula	$C_{14}H_{15}ClN_4O_6$
Formula weight	370.75
Temperature [K]	100(2)
Crystal system	monoclinic
Space group (number)	$P2_1/n$ (14)
a [Å]	8.4598(4)
b [Å]	11.6323(5)
c [Å]	16.1510(7)
α [°]	90
β [°]	100.334(2)
γ [°]	90
Volume [Å^3]	1563.59(12)
Z	4
ρ_{calc} [gcm^{-3}]	1.575
μ [mm^{-1}]	0.287
F(000)	768
Crystal size [mm^3]	0.288×0.431×0.477
Crystal colour	red
Crystal shape	block
Radiation	MoK_α (λ=0.71073 Å)
2θ range [°]	4.34 to 52.95 (0.80 Å)
Index ranges	$-10 \leq h \leq 10$<br>$-14 \leq k \leq 14$<br>$-20 \leq l \leq 20$
Reflections collected	64182
Independent reflections	3214<br>R_{int} = 0.0403<br>R_{sigma} = 0.0122
Completeness to θ = 25.242°	100.0
Data / Restraints / Parameters	3214 / 0 / 229
Goodness-of-fit on F^2	1.151
Final R indexes [$I \geq 2\sigma(I)$]	R_1 = 0.0390<br>wR_2 = 0.0855
Final R indexes [all data]	R_1 = 0.0460<br>wR_2 = 0.0926
Largest peak/hole [eÅ^{-3}]	0.30/−0.50

Table SI-9. Bond lengths and angles for compound **1**.

Atom–Atom	Length [Å]
C1–N1	1.397(2)
C1–C6	1.410(3)
C1–C2	1.415(3)
C2–N2	1.375(2)
C2–C3	1.406(3)
C3–C4	1.373(3)
C3–H3	0.9500
C4–C5	1.397(3)
C4–H4	0.9500
C5–C6	1.373(3)
C5–H5	0.9500
C6–H6	0.9500
C7–N1	1.321(2)
C7–C8	1.441(2)
C7–C9	1.501(2)
C8–N4	1.308(2)
C8–N2	1.364(2)
C9–O1	1.217(2)
C9–N3	1.365(2)
C10–O2	1.221(2)
C10–N4	1.366(2)
C10–N3	1.406(2)
C11–N1	1.508(2)
C11–C12	1.517(3)
C11–H11A	0.9900
C11–H11B	0.9900
C12–H12A	0.9800
C12–H12B	0.9800
C12–H12C	0.9800
C13–N2	1.477(2)
C13–H13A	0.9800
C13–H13B	0.9800
C13–H13C	0.9800
C14–N3	1.471(2)
C14–H14A	0.9800
C14–H14B	0.9800
C14–H14C	0.9800
O3–Cl1	1.4422(14)
O4–Cl1	1.4393(13)
O5–Cl1	1.4412(14)
O6–Cl1	1.4398(13)

Atom–Atom–Atom	Angle [°]
N1–C1–C6	121.48(17)
N1–C1–C2	118.45(16)
C6–C1–C2	120.07(17)
N2–C2–C3	120.98(17)
N2–C2–C1	120.06(16)
C3–C2–C1	118.95(17)
C4–C3–C2	119.88(18)
C4–C3–H3	120.1
C2–C3–H3	120.1
C3–C4–C5	121.13(18)
C3–C4–H4	119.4
C5–C4–H4	119.4
C6–C5–C4	120.46(18)
C6–C5–H5	119.8
C4–C5–H5	119.8
C5–C6–C1	119.50(18)
C5–C6–H6	120.2
C1–C6–H6	120.2
N1–C7–C8	121.87(16)
N1–C7–C9	121.82(16)
C8–C7–C9	116.30(16)
N4–C8–N2	118.83(16)
N4–C8–C7	124.04(17)
N2–C8–C7	117.13(16)
O1–C9–N3	122.79(17)
O1–C9–C7	122.99(17)
N3–C9–C7	114.22(16)
O2–C10–N4	121.84(18)
O2–C10–N3	118.30(18)
N4–C10–N3	119.85(16)
N1–C11–C12	111.45(16)
N1–C11–H11A	109.3
C12–C11–H11A	109.3
N1–C11–H11B	109.3
C12–C11–H11B	109.3
H11A–C11–H11B	108.0

C11–C12–H12A	109.5
C11–C12–H12B	109.5
H12A–C12–H12B	109.5
C11–C12–H12C	109.5
H12A–C12–H12C	109.5
H12B–C12–H12C	109.5
N2–C13–H13A	109.5
N2–C13–H13B	109.5
H13A–C13–H13B	109.5
N2–C13–H13C	109.5
H13A–C13–H13C	109.5
H13B–C13–H13C	109.5
N3–C14–H14A	109.5
N3–C14–H14B	109.5
H14A–C14–H14B	109.5
N3–C14–H14C	109.5
H14A–C14–H14C	109.5

H14B–C14–H14C	109.5
C7–N1–C1	120.51(15)
C7–N1–C11	122.45(15)
C1–N1–C11	116.96(14)
C8–N2–C2	121.33(15)
C8–N2–C13	119.16(15)
C2–N2–C13	119.51(15)
C9–N3–C10	124.37(16)
C9–N3–C14	116.94(16)
C10–N3–C14	118.52(15)
C8–N4–C10	119.76(16)
O4–Cl1–O6	110.03(8)
O4–Cl1–O5	109.39(9)
O6–Cl1–O5	109.01(9)
O4–Cl1–O3	109.15(8)
O6–Cl1–O3	109.65(9)
O5–Cl1–O3	109.60(9)

Table SI-10. Crystal data and structure refinement for compound **2**.

CCDC number	2532557
Empirical formula	$C_{14}H_{16}N_4O_4$
Formula weight	304.31
Temperature [K]	100(2)
Crystal system	monoclinic
Space group (number)	$P2_1/n$ (14)
a [Å]	9.0354(12)
b [Å]	7.6972(11)
c [Å]	19.869(3)
α [°]	90
β [°]	100.296(6)
γ [°]	90
Volume [Å^3]	1359.6(3)
Z	4
ρ_{calc} [gcm^{-3}]	1.487
μ [mm^{-1}]	0.112
F(000)	640
Crystal size [mm^3]	0.020×0.075×0.101
Crystal colour	yellow
Crystal shape	plate
Radiation	MoK_α (λ=0.71073 Å)
2θ range [°]	5.36 to 56.64 (0.75 Å)
Index ranges	$-12 \leq h \leq 12$<br>$-10 \leq k \leq 10$<br>$-26 \leq l \leq 26$
Reflections collected	20973
Independent reflections	3372<br>R_{int} = 0.0738<br>R_{sigma} = 0.0485
Completeness to θ = 25.242°	99.9
Data / Restraints / Parameters	3372 / 0 / 205
Goodness-of-fit on F^2	1.025
Final R indexes [$I \geq 2\sigma(I)$]	R_1 = 0.0431<br>wR_2 = 0.0954
Final R indexes [all data]	R_1 = 0.0724<br>wR_2 = 0.1079
Largest peak/hole [eÅ^{-3}]	0.39/−0.24

Table SI-11. Bond lengths and angles for compound **2**.

Atom–Atom	Length [Å]
C1–C2	1.399(2)
C1–C6	1.401(2)
C1–N1	1.407(2)
C2–C3	1.375(2)
C2–H2	0.9500
C3–C4	1.384(2)
C3–H3	0.9500
C4–C5	1.379(2)
C4–H4	0.9500
C5–C6	1.394(2)
C5–H5	0.9500
C6–N2	1.4121(19)
C7–N3	1.3052(19)
C7–N2	1.340(2)
C7–C10	1.521(2)
C8–O3	1.2263(18)
C8–N3	1.361(2)
C8–N4	1.406(2)
C9–O4	1.2126(19)
C9–N4	1.3768(19)
C9–C10	1.541(2)
C10–N1	1.429(2)
C10–O1	1.4523(18)
C11–N2	1.473(2)
C11–H11A	0.9800
C11–H11B	0.9800
C11–H11C	0.9800
C12–N4	1.469(2)
C12–H12A	0.9800
C12–H12B	0.9800
C12–H12C	0.9800
C13–N1	1.479(2)
C13–C14	1.523(2)
C13–H13A	0.9900
C13–H13B	0.9900
C14–H14A	0.9800
C14–H14B	0.9800
C14–H14C	0.9800
O1–O2	1.4665(15)
O2–H1	0.94(2)

Atom–Atom–Atom	Angle [°]
C2–C1–C6	117.96(14)
C2–C1–N1	122.87(14)
C6–C1–N1	119.14(13)
C3–C2–C1	120.97(15)
C3–C2–H2	119.5
C1–C2–H2	119.5
C2–C3–C4	120.85(15)
C2–C3–H3	119.6
C4–C3–H3	119.6
C5–C4–C3	119.18(16)
C5–C4–H4	120.4
C3–C4–H4	120.4
C4–C5–C6	120.64(15)
C4–C5–H5	119.7
C6–C5–H5	119.7
C5–C6–C1	120.36(14)
C5–C6–N2	120.93(14)
C1–C6–N2	118.63(13)
N3–C7–N2	119.21(13)
N3–C7–C10	124.06(14)
N2–C7–C10	116.63(12)
O3–C8–N3	120.77(14)
O3–C8–N4	119.13(14)
N3–C8–N4	120.07(13)
O4–C9–N4	121.35(15)
O4–C9–C10	120.97(14)
N4–C9–C10	117.51(13)
N1–C10–O1	105.76(11)
N1–C10–C7	109.70(12)
O1–C10–C7	108.78(12)
N1–C10–C9	114.78(13)
O1–C10–C9	105.71(12)
C7–C10–C9	111.72(12)
N2–C11–H11A	109.5
N2–C11–H11B	109.5
H11A–C11–H11B	109.5
N2–C11–H11C	109.5

H11A–C11–H11C	109.5
H11B–C11–H11C	109.5
N4–C12–H12A	109.5
N4–C12–H12B	109.5
H12A–C12–H12B	109.5
N4–C12–H12C	109.5
H12A–C12–H12C	109.5
H12B–C12–H12C	109.5
N1–C13–C14	115.57(13)
N1–C13–H13A	108.4
C14–C13–H13A	108.4
N1–C13–H13B	108.4
C14–C13–H13B	108.4
H13A–C13–H13B	107.4
C13–C14–H14A	109.5
C13–C14–H14B	109.5

H14A–C14–H14B	109.5
C13–C14–H14C	109.5
H14A–C14–H14C	109.5
H14B–C14–H14C	109.5
C1–N1–C10	113.89(12)
C1–N1–C13	118.03(12)
C10–N1–C13	123.48(13)
C7–N2–C6	121.00(12)
C7–N2–C11	119.53(12)
C6–N2–C11	119.47(13)
C7–N3–C8	120.72(13)
C9–N4–C8	122.40(13)
C9–N4–C12	117.38(13)
C8–N4–C12	118.44(13)
C10–O1–O2	107.11(10)
O1–O2–H1	97.6(13)

Table SI-12. Crystal data and structure refinement for compound **4a**.

CCDC number	2532561
Empirical formula	$C_{14}H_{17}NO$
Formula weight	215.28
Temperature [K]	100(2)
Crystal system	monoclinic
Space group (number)	$P2_1/c$ (14)
a [Å]	13.6516(16)
b [Å]	9.4830(13)
c [Å]	18.220(2)
α [°]	90
β [°]	90.313(5)
γ [°]	90
Volume [Å^3]	2358.7(5)
Z	8
ρ_{calc} [gcm^{-3}]	1.213
μ [mm^{-1}]	0.076
F(000)	928
Crystal size [mm^3]	0.110×0.145×0.666
Crystal colour	colourless
Crystal shape	block
Radiation	MoK_α (λ=0.71073 Å)
2θ range [°]	4.30 to 52.98 (0.80 Å)
Index ranges	$-17 \leq h \leq 17$<br>$-11 \leq k \leq 11$<br>$-22 \leq l \leq 22$
Reflections collected	115166
Independent reflections	4852<br>R_{int} = 0.1002<br>R_{sigma} = 0.0336
Completeness to θ = 25.242°	99.9
Data / Restraints / Parameters	4852 / 0 / 300
Goodness-of-fit on F^2	1.084
Final R indexes [$I \geq 2\sigma(I)$]	R_1 = 0.0540<br>wR_2 = 0.1471
Final R indexes [all data]	R_1 = 0.0576<br>wR_2 = 0.1501
Largest peak/hole [eÅ^{-3}]	0.27/−0.28

Table SI-13. Bond lengths and angles for compound **4a**.

Atom–Atom	Length [Å]
C1_1–C2_1	1.384(3)
C1_1–C6_1	1.391(3)
C1_1–C8_1	1.501(3)
C2_1–C3_1	1.398(3)
C2_1–H2_1	0.9500
C3_1–C4_1	1.385(3)
C3_1–H3_1	0.9500
C4_1–C5_1	1.392(3)
C4_1–H4_1	0.9500
C5_1–C6_1	1.387(3)
C5_1–H5_1	0.9500
C6_1–N1_1	1.437(3)
C7_1–N1_1	1.289(3)
C7_1–C9_1	1.513(3)
C7_1–C8_1	1.541(3)
C8_1–O1_1	1.426(2)
C8_1–C12_1	1.535(3)
C9_1–C13_1	1.522(3)
C9_1–C14_1	1.539(3)
C9_1–C10_1	1.551(4)
C10_1–C11_1	1.518(4)
C10_1–H10A_1	0.9900
C10_1–H10B_1	0.9900
C11_1–C12_1	1.529(3)
C11_1–H11A_1	0.9900
C11_1–H11B_1	0.9900
C12_1–H12A_1	0.9900
C12_1–H12B_1	0.9900
C13_1–H13A_1	0.9800
C13_1–H13B_1	0.9800
C13_1–H13C_1	0.9800
C14_1–H14A_1	0.9800
C14_1–H14B_1	0.9800
C14_1–H14C_1	0.9800
O1_1–H1_1	0.82(3)
C1_2–C6_2	1.382(3)
C1_2–C2_2	1.392(3)
C1_2–C7_2	1.503(3)
C2_2–C3_2	1.381(3)
C2_2–N1_2	1.438(3)
C3_2–C4_2	1.393(3)
C3_2–H3_2	0.9500
C4_2–C5_2	1.393(3)
C4_2–H4_2	0.9500
C5_2–C6_2	1.391(3)
C5_2–H5_2	0.9500
C6_2–H6_2	0.9500
C7_2–O1_2	1.419(2)
C7_2–C9_2	1.538(3)
C7_2–C8_2	1.546(3)
C8_2–N1_2	1.283(3)
C8_2–C12_2	1.503(3)
C9_2–C10_2	1.524(3)
C9_2–H9A_2	0.9900
C9_2–H9B_2	0.9900
C10_2–C11_2	1.532(3)
C10_2–H10A_2	0.9900
C10_2–H10B_2	0.9900
C11_2–C12_2	1.550(3)
C11_2–H11A_2	0.9900
C11_2–H11B_2	0.9900
C12_2–C13_2	1.535(3)
C12_2–C14_2	1.542(3)
C13_2–H13A_2	0.9800
C13_2–H13B_2	0.9800
C13_2–H13C_2	0.9800
C14_2–H14A_2	0.9800
C14_2–H14B_2	0.9800
C14_2–H14C_2	0.9800
O1_2–H1_2	0.93(3)

Atom–Atom–Atom	Angle [°]
C2_1–C1_1–C6_1	120.23(19)
C2_1–C1_1–C8_1	132.34(19)
C6_1–C1_1–C8_1	107.42(17)
C1_1–C2_1–C3_1	118.4(2)
C1_1–C2_1–H2_1	120.8
C3_1–C2_1–H2_1	120.8
C4_1–C3_1–C2_1	120.9(2)

C4_1–C3_1–H3_1	119.5
C2_1–C3_1–H3_1	119.5
C3_1–C4_1–C5_1	120.9(2)
C3_1–C4_1–H4_1	119.5
C5_1–C4_1–H4_1	119.5
C6_1–C5_1–C4_1	117.7(2)
C6_1–C5_1–H5_1	121.1
C4_1–C5_1–H5_1	121.1
C5_1–C6_1–C1_1	121.8(2)
C5_1–C6_1–N1_1	126.65(19)
C1_1–C6_1–N1_1	111.55(18)
N1_1–C7_1–C9_1	124.87(19)
N1_1–C7_1–C8_1	113.50(18)
C9_1–C7_1–C8_1	120.66(18)
O1_1–C8_1–C1_1	115.67(16)
O1_1–C8_1–C12_1	106.84(16)
C1_1–C8_1–C12_1	112.32(17)
O1_1–C8_1–C7_1	116.82(16)
C1_1–C8_1–C7_1	100.08(16)
C12_1–C8_1–C7_1	104.68(16)
C7_1–C9_1–C13_1	111.1(2)
C7_1–C9_1–C14_1	111.21(18)
C13_1–C9_1–C14_1	108.9(2)
C7_1–C9_1–C10_1	106.52(18)
C13_1–C9_1–C10_1	109.20(19)
C14_1–C9_1–C10_1	109.8(2)
C11_1–C10_1–C9_1	114.83(18)
C11_1–C10_1–H10A_1	108.6
C9_1–C10_1–H10A_1	108.6
C11_1–C10_1–H10B_1	108.6
C9_1–C10_1–H10B_1	108.6
H10A_1–C10_1–H10B_1	107.5
C10_1–C11_1–C12_1	110.57(19)
C10_1–C11_1–H11A_1	109.5
C12_1–C11_1–H11A_1	109.5
C10_1–C11_1–H11B_1	109.5
C12_1–C11_1–H11B_1	109.5
H11A_1–C11_1–H11B_1	108.1
C11_1–C12_1–C8_1	110.25(18)
C11_1–C12_1–H12A_1	109.6
C8_1–C12_1–H12A_1	109.6
C11_1–C12_1–H12B_1	109.6

C8_1–C12_1–H12B_1	109.6
H12A_1–C12_1–H12B_1	108.1
C9_1–C13_1–H13A_1	109.5
C9_1–C13_1–H13B_1	109.5
H13A_1–C13_1–H13B_1	109.5
C9_1–C13_1–H13C_1	109.5
H13A_1–C13_1–H13C_1	109.5
H13B_1–C13_1–H13C_1	109.5
C9_1–C14_1–H14A_1	109.5
C9_1–C14_1–H14B_1	109.5
H14A_1–C14_1–H14B_1	109.5
C9_1–C14_1–H14C_1	109.5
H14A_1–C14_1–H14C_1	109.5
H14B_1–C14_1–H14C_1	109.5
C7_1–N1_1–C6_1	107.27(17)
C8_1–O1_1–H1_1	103(2)
C6_2–C1_2–C2_2	121.02(19)
C6_2–C1_2–C7_2	131.77(19)
C2_2–C1_2–C7_2	107.21(17)
C3_2–C2_2–C1_2	121.58(19)
C3_2–C2_2–N1_2	126.47(18)
C1_2–C2_2–N1_2	111.94(17)
C2_2–C3_2–C4_2	117.3(2)
C2_2–C3_2–H3_2	121.3
C4_2–C3_2–H3_2	121.3
C5_2–C4_2–C3_2	121.5(2)
C5_2–C4_2–H4_2	119.3
C3_2–C4_2–H4_2	119.3
C6_2–C5_2–C4_2	120.6(2)
C6_2–C5_2–H5_2	119.7
C4_2–C5_2–H5_2	119.7
C1_2–C6_2–C5_2	118.0(2)
C1_2–C6_2–H6_2	121.0
C5_2–C6_2–H6_2	121.0
O1_2–C7_2–C1_2	114.38(16)
O1_2–C7_2–C9_2	107.27(16)
C1_2–C7_2–C9_2	113.21(17)
O1_2–C7_2–C8_2	117.10(16)
C1_2–C7_2–C8_2	99.75(16)
C9_2–C7_2–C8_2	104.83(16)
N1_2–C8_2–C12_2	124.14(18)
N1_2–C8_2–C7_2	113.97(17)

C12_2–C8_2–C7_2	120.92(17)
C10_2–C9_2–C7_2	110.37(18)
C10_2–C9_2–H9A_2	109.6
C7_2–C9_2–H9A_2	109.6
C10_2–C9_2–H9B_2	109.6
C7_2–C9_2–H9B_2	109.6
H9A_2–C9_2–H9B_2	108.1
C9_2–C10_2–C11_2	110.73(18)
C9_2–C10_2–H10A_2	109.5
C11_2–C10_2–H10A_2	109.5
C9_2–C10_2–H10B_2	109.5
C11_2–C10_2–H10B_2	109.5
H10A_2–C10_2–H10B_2	108.1
C10_2–C11_2–C12_2	114.43(18)
C10_2–C11_2–H11A_2	108.7
C12_2–C11_2–H11A_2	108.7
C10_2–C11_2–H11B_2	108.7
C12_2–C11_2–H11B_2	108.7
H11A_2–C11_2–H11B_2	107.6
C8_2–C12_2–C13_2	110.98(17)

C8_2–C12_2–C14_2	110.66(17)
C13_2–C12_2–C14_2	108.59(19)
C8_2–C12_2–C11_2	106.39(18)
C13_2–C12_2–C11_2	109.39(18)
C14_2–C12_2–C11_2	110.83(18)
C12_2–C13_2–H13A_2	109.5
C12_2–C13_2–H13B_2	109.5
H13A_2–C13_2–H13B_2	109.5
C12_2–C13_2–H13C_2	109.5
H13A_2–C13_2–H13C_2	109.5
H13B_2–C13_2–H13C_2	109.5
C12_2–C14_2–H14A_2	109.5
C12_2–C14_2–H14B_2	109.5
H14A_2–C14_2–H14B_2	109.5
C12_2–C14_2–H14C_2	109.5
H14A_2–C14_2–H14C_2	109.5
H14B_2–C14_2–H14C_2	109.5
C8_2–N1_2–C2_2	106.87(17)
C7_2–O1_2–H1_2	107.4(17)

Table SI-14. Crystal data and structure refinement for compound **8**.

CCDC number	2532558
Empirical formula	$C_{14}H_{15}N_4O_2^{\bullet}$
Formula weight	271.30
Temperature [K]	216(2)
Crystal system	monoclinic
Space group (number)	$P2_1/m$ (11)
a [Å]	9.201(3)
b [Å]	7.008(2)
c [Å]	9.730(3)
α [°]	90
β [°]	96.908(8)
γ [°]	90
Volume [Å^3]	622.8(3)
Z	2
ρ_{calc} [gcm^{-3}]	1.447
μ [mm^{-1}]	0.101
F(000)	286
Crystal size [mm^3]	0.043×0.123×0.170
Crystal colour	dark red
Crystal shape	fragment
Radiation	MoK_α (λ=0.71073 Å)
2θ range [°]	6.50 to 52.81 (0.80 Å)
Index ranges	$-11 \leq h \leq 11$<br>$-8 \leq k \leq 8$<br>$-12 \leq l \leq 12$
Reflections collected	34447
Independent reflections	1372<br>R_{int} = 0.0499<br>R_{sigma} = 0.0143
Completeness to<br>θ = 25.242°	99.5
Data / Restraints / Parameters	1372 / 165 / 154
Goodness-of-fit on F^2	1.097
Final *R* indexes<br>[$I \geq 2\sigma(I)$]	R_1 = 0.0409<br>wR_2 = 0.1148
Final *R* indexes<br>[all data]	R_1 = 0.0517<br>wR_2 = 0.1266
Largest peak/hole [eÅ^{-3}]	0.32/−0.17

Table SI-15. Bond lengths and angles for compound **8**.

**Atom–Atom**	**Length [Å]**
N2–C10	1.376(2)
N2–C8	1.400(2)
N2–C9	1.466(2)
N3–C10	1.320(2)
N3–C11	1.358(2)
N4–C13	1.400(3)
N4–C11	1.401(2)
N4–C12	1.471(2)
O1–C11	1.230(2)
C3–C4	1.410(4)
C3–C8	1.412(2)
C3–N1	1.414(3)
C8–C7	1.405(4)
C10–C14	1.432(3)
N1–C14	1.385(3)
N1–C1	1.488(3)
C1–C2	1.516(3)
C4–C5	1.372(3)
C5–C6	1.384(3)
C6–C7	1.386(3)
C13–O2	1.227(2)
C13–C14	1.461(3)

**Atom–Atom–Atom**	**Angle [°]**
C10–N2–C8	121.41(14)
C10–N2–C9	118.94(14)
C8–N2–C9	119.65(14)
C10–N3–C11	120.04(14)
C13–N4–C11	124.60(16)
C13–N4–C12	117.72(15)
C11–N4–C12	117.20(14)
C4–C3–C8	119.02(18)
C4–C3–N1	122.0(2)
C8–C3–N1	118.75(15)
N2–C8–C7	121.04(17)
N2–C8–C3	119.61(15)
C7–C8–C3	119.00(17)
N3–C10–N2	116.65(15)
N3–C10–C14	124.43(17)
N2–C10–C14	118.46(16)
O1–C11–N3	123.01(16)
O1–C11–N4	118.41(16)
N3–C11–N4	118.58(14)
C14–N1–C3	119.6(2)
C14–N1–C1	122.5(2)
C3–N1–C1	117.89(16)
N1–C1–C2	111.7(2)
C5–C4–C3	120.7(2)
C4–C5–C6	120.4(2)
C5–C6–C7	120.3(2)
C6–C7–C8	120.4(2)
O2–C13–N4	119.6(2)
O2–C13–C14	125.46(18)
N4–C13–C14	114.6(2)
N1–C14–C10	120.57(19)
N1–C14–C13	122.51(19)
C10–C14–C13	116.8(2)

Table SI-16. Crystal data and structure refinement for compound **10**.

CCDC number	2532556
Empirical formula	$C_{14}H_{15}N_4O_2^{\bullet}$
Formula weight	271.30
Temperature [K]	100(2)
Crystal system	orthorhombic
Space group (number)	*Pnma* (62)
a [Å]	19.7701(18)
b [Å]	6.7394(6)
c [Å]	9.2263(8)
α [°]	90
β [°]	90
γ [°]	90
Volume [Å^3]	1229.30(19)
Z	4
ρ_{calc} [gcm^{-3}]	1.466
μ [mm^{-1}]	0.102
F(000)	572
Crystal size [mm^3]	0.053×0.101×0.231
Crystal colour	dark red
Crystal shape	plate
Radiation	MoK_α (λ=0.71073 Å)
2θ range [°]	4.87 to 56.56 (0.75 Å)
Index ranges	$-26 \leq h \leq 26$<br>$-8 \leq k \leq 6$<br>$-12 \leq l \leq 12$
Reflections collected	14953
Independent reflections	1638<br>R_{int} = 0.0627<br>R_{sigma} = 0.0305
Completeness to θ = 25.242°	99.6
Data / Restraints / Parameters	1638 / 57 / 154
Goodness-of-fit on F^2	1.095
Final *R* indexes [$I \geq 2\sigma(I)$]	R_1 = 0.0634<br>wR_2 = 0.1715
Final *R* indexes [all data]	R_1 = 0.0786<br>wR_2 = 0.1835
Largest peak/hole [eÅ^{-3}]	0.41/−0.32

Table SI-17. Bond lengths and angles for compound **10**.

Atom–Atom	Length [Å]
C5–N3	1.307(4)
C5–N2	1.397(4)
C5–C6_1	1.410(9)
N3–C14	1.377(4)
C14–C13	1.396(4)
C14–C9	1.426(3)
C9–N4_1	1.397(4)
C9–C10	1.401(4)
C10–C11	1.377(4)
C12–C13	1.367(4)
C12–C11	1.395(4)
N2–C3_1	1.371(5)
N2–C4	1.472(3)
O1_1–C1_1	1.214(4)
C1_1–N1_1	1.406(4)
C1_1–C6_1	1.431(8)
N1_1–C3_1	1.380(4)
N1_1–C2_1	1.464(5)
N4_1–C6_1	1.395(12)
N4_1–C7_1	1.485(4)
C7_1–C8_1	1.510(5)
C3_1–O2_1	1.222(5)

Atom–Atom–Atom	Angle [°]
N3–C5–N2	116.2(2)
N3–C5–C6_1	125.9(4)
N2–C5–C6_1	117.9(3)
C5–N3–C14	116.4(2)
N3–C14–C13	118.9(2)
N3–C14–C9	122.7(2)
C13–C14–C9	118.4(3)
N4_1–C9–C10	122.1(2)
N4_1–C9–C14	117.3(2)
C10–C9–C14	119.9(2)
C11–C10–C9	119.5(2)
C13–C12–C11	120.2(3)
C12–C13–C14	121.1(2)
C10–C11–C12	120.8(3)

C3_1–N2–C5	123.6(3)
C3_1–N2–C4	118.5(3)
C5–N2–C4	117.4(2)
O1_1–C1_1–N1_1	118.8(3)
O1_1–C1_1–C6_1	126.4(3)
N1_1–C1_1–C6_1	114.8(3)
C3_1–N1_1–C1_1	125.7(3)
C3_1–N1_1–C2_1	116.7(3)
C1_1–N1_1–C2_1	117.6(3)
C6_1–N4_1–C9	119.1(11)
C6_1–N4_1–C7_1	122.3(9)
C9–N4_1–C7_1	118.6(3)
N4_1–C6_1–C5	117.2(4)
N4_1–C6_1–C1_1	121.2(13)
C5–C6_1–C1_1	121.4(3)
N4_1–C7_1–C8_1	111.9(4)
O2_1–C3_1–N2	122.9(4)
O2_1–C3_1–N1_1	120.9(4)
N2–C3_1–N1_1	115.9(4)

Table SI-18. Crystal data and structure refinement for compound **11**.

CCDC number	2532559
Empirical formula	$C_{15}H_{18}N_4O_3$
Formula weight	302.33
Temperature [K]	100(2)
Crystal system	monoclinic
Space group (number)	$P2_1/n$ (14)
a [Å]	10.4120(7)
b [Å]	12.6623(8)
c [Å]	10.9162(6)
α [°]	90
β [°]	100.067(2)
γ [°]	90
Volume [Å^3]	1417.03(15)
Z	4
ρ_{calc} [gcm^{-3}]	1.417
μ [mm^{-1}]	0.102
F(000)	640
Crystal size [mm^3]	0.106×0.123×0.184
Crystal colour	faint yellow
Crystal shape	block
Radiation	MoK_α (λ=0.71073 Å)
2θ range [°]	4.97 to 56.59 (0.75 Å)
Index ranges	$-13 \leq h \leq 13$<br>$-16 \leq k \leq 16$<br>$-14 \leq l \leq 14$
Reflections collected	85078
Independent reflections	3530<br>R_{int} = 0.0902<br>R_{sigma} = 0.0243
Completeness to θ = 25.242°	100.0
Data / Restraints / Parameters	3530 / 0 / 203
Goodness-of-fit on F^2	1.025
Final R indexes [$I \geq 2\sigma(I)$]	R_1 = 0.0401<br>wR_2 = 0.1036
Final R indexes [all data]	R_1 = 0.0535<br>wR_2 = 0.1143
Largest peak/hole [eÅ^{-3}]	0.36/−0.20

Table SI-19. Bond lengths and angles for compound **11**.

Atom–Atom	Length [Å]
O1–C7	1.2109(16)
O2–C9	1.4361(16)
O2–C8	1.4490(15)
O3–C2	1.2139(16)
N1–C7	1.3741(16)
N1–C2	1.3981(18)
N1–C1	1.4719(16)
N2–C2	1.3769(17)
N2–C3	1.3980(16)
N2–C11	1.4672(17)
N3–C3	1.2780(16)
N3–C4	1.3986(16)
N4–C10	1.3987(15)
N4–C8	1.4362(16)
N4–C14	1.4770(16)
C1–H3	0.9800
C1–H18	0.9800
C1–H1	0.9800
C3–C8	1.5166(17)
C4–C5	1.3966(17)
C4–C10	1.4058(17)
C5–C6	1.3799(19)
C5–H12	0.9500
C6–C12	1.394(2)
C6–H2	0.9500
C7–C8	1.5455(17)
C9–H4	0.9800
C9–H5	0.9800
C9–H6	0.9800
C10–C13	1.4029(17)
C11–H7	0.9800
C11–H8	0.9800
C11–H9	0.9800
C12–C13	1.3882(18)
C12–H11	0.9500
C13–H10	0.9500
C14–C15	1.522(2)
C14–H16	0.9900
C14–H17	0.9900
C15–H13	0.9800
C15–H14	0.9800
C15–H15	0.9800

Atom–Atom–Atom	Angle [°]
C9–O2–C8	114.91(10)
C7–N1–C2	125.60(11)
C7–N1–C1	116.92(11)
C2–N1–C1	117.10(11)
C2–N2–C3	123.24(11)
C2–N2–C11	116.70(11)
C3–N2–C11	118.31(11)
C3–N3–C4	117.84(11)
C10–N4–C8	120.27(10)
C10–N4–C14	116.99(10)
C8–N4–C14	118.46(10)
N1–C1–H3	109.5
N1–C1–H18	109.5
H3–C1–H18	109.5
N1–C1–H1	109.5
H3–C1–H1	109.5
H18–C1–H1	109.5
O3–C2–N2	122.19(13)
O3–C2–N1	120.75(12)
N2–C2–N1	117.03(11)
N3–C3–N2	118.06(11)
N3–C3–C8	126.24(11)
N2–C3–C8	115.33(11)
C5–C4–N3	118.14(11)
C5–C4–C10	120.46(12)
N3–C4–C10	121.38(11)
C6–C5–C4	121.06(12)
C6–C5–H12	119.5
C4–C5–H12	119.5
C5–C6–C12	118.86(12)
C5–C6–H2	120.6
C12–C6–H2	120.6
O1–C7–N1	120.97(12)
O1–C7–C8	123.55(11)
N1–C7–C8	115.28(11)

N4–C8–O2	113.32(10)
N4–C8–C3	110.34(10)
O2–C8–C3	109.91(10)
N4–C8–C7	112.81(10)
O2–C8–C7	99.24(9)
C3–C8–C7	110.78(10)
O2–C9–H4	109.5
O2–C9–H5	109.5
H4–C9–H5	109.5
O2–C9–H6	109.5
H4–C9–H6	109.5
H5–C9–H6	109.5
N4–C10–C13	122.56(11)
N4–C10–C4	119.50(11)
C13–C10–C4	117.94(11)
N2–C11–H7	109.5
N2–C11–H8	109.5
H7–C11–H8	109.5
N2–C11–H9	109.5
H7–C11–H9	109.5

H8–C11–H9	109.5
C13–C12–C6	120.83(12)
C13–C12–H11	119.6
C6–C12–H11	119.6
C12–C13–C10	120.82(12)
C12–C13–H10	119.6
C10–C13–H10	119.6
N4–C14–C15	112.76(11)
N4–C14–H16	109.0
C15–C14–H16	109.0
N4–C14–H17	109.0
C15–C14–H17	109.0
H16–C14–H17	107.8
C14–C15–H13	109.5
C14–C15–H14	109.5
H13–C15–H14	109.5
C14–C15–H15	109.5
H13–C15–H15	109.5
H14–C15–H15	109.5

Table SI-20. Crystal data and structure refinement for compound **21**.

CCDC number	2532555
Empirical formula	$C_{14}H_{17}NO_2$
Formula weight	231.28
Temperature [K]	100(2)
Crystal system	monoclinic
Space group (number)	$P2_1/c$ (14)
a [Å]	6.1939(5)
b [Å]	9.3622(6)
c [Å]	20.2218(16)
α [°]	90
β [°]	98.373(3)
γ [°]	90
Volume [$Å^3$]	1160.13(15)
Z	4
ρ_{calc} [gcm^{-3}]	1.324
μ [mm^{-1}]	0.088
F(000)	496
Crystal size [mm^3]	0.027×0.085×0.135
Crystal colour	colourless
Crystal shape	block
Radiation	MoK_α (λ=0.71073 Å)
2θ range [°]	4.80 to 52.85 (0.80 Å)
Index ranges	$-7 \leq h \leq 7$<br>$-9 \leq k \leq 11$<br>$-25 \leq l \leq 25$
Reflections collected	15795
Independent reflections	2378<br>R_{int} = 0.0494<br>R_{sigma} = 0.0247
Completeness to θ = 25.242°	99.9
Data / Restraints / Parameters	2378 / 0 / 162
Goodness-of-fit on F^2	1.031
Final *R* indexes [$I \geq 2\sigma(I)$]	R_1 = 0.0354<br>wR_2 = 0.0900
Final *R* indexes [all data]	R_1 = 0.0423<br>wR_2 = 0.0949
Largest peak/hole [$eÅ^{-3}$]	0.33/−0.26

Table SI-21. Bond lengths and angles for compound **21**.

**Atom–Atom**	**Length [Å]**
C1–C2	1.3832(17)
C1–C6	1.3977(18)
C1–C8	1.5052(16)
C2–C3	1.3959(17)
C2–H2	0.9500
C3–C4	1.388(2)
C3–H3	0.9500
C4–C5	1.3948(19)
C4–H4	0.9500
C5–C6	1.3851(17)
C5–H5	0.9500
C6–N1	1.4043(16)
C7–O1	1.2290(15)
C7–N1	1.3552(16)
C7–C8	1.5361(16)
C8–C9	1.5411(16)
C8–C12	1.5764(16)
C9–O2	1.4184(14)
C9–C10	1.5311(16)
C9–H9	1.0000
C10–C11	1.5448(17)
C10–H10A	0.9900
C10–H10B	0.9900
C11–C12	1.5546(16)
C11–H11A	0.9900
C11–H11B	0.9900
C12–C13	1.5278(17)
C12–C14	1.5329(17)
C13–H13A	0.9800
C13–H13B	0.9800
C13–H13C	0.9800
C14–H14A	0.9800
C14–H14B	0.9800
C14–H14C	0.9800
N1–H6	0.883(18)
O2–H1	0.856(18)

**Atom–Atom–Atom**	**Angle [°]**
C2–C1–C6	119.18(11)
C2–C1–C8	131.50(11)
C6–C1–C8	109.13(10)
C1–C2–C3	119.10(12)
C1–C2–H2	120.4
C3–C2–H2	120.4
C4–C3–C2	120.74(12)
C4–C3–H3	119.6
C2–C3–H3	119.6
C3–C4–C5	120.94(11)
C3–C4–H4	119.5
C5–C4–H4	119.5
C6–C5–C4	117.32(12)
C6–C5–H5	121.3
C4–C5–H5	121.3
C5–C6–C1	122.55(11)
C5–C6–N1	128.57(12)
C1–C6–N1	108.83(10)
O1–C7–N1	125.39(11)
O1–C7–C8	126.17(11)
N1–C7–C8	108.38(10)
C1–C8–C7	101.63(9)
C1–C8–C9	114.99(10)
C7–C8–C9	112.75(10)
C1–C8–C12	117.63(10)
C7–C8–C12	108.36(9)
C9–C8–C12	101.72(9)
O2–C9–C10	116.07(10)
O2–C9–C8	114.70(9)
C10–C9–C8	104.62(9)
O2–C9–H9	107.0
C10–C9–H9	107.0
C8–C9–H9	107.0
C9–C10–C11	105.02(10)
C9–C10–H10A	110.7
C11–C10–H10A	110.7
C9–C10–H10B	110.7
C11–C10–H10B	110.7
H10A–C10–H10B	108.8
C10–C11–C12	107.75(9)

C10–C11–H11A	110.2
C12–C11–H11A	110.2
C10–C11–H11B	110.2
C12–C11–H11B	110.2
H11A–C11–H11B	108.5
C13–C12–C14	107.63(10)
C13–C12–C11	112.48(10)
C14–C12–C11	110.24(10)
C13–C12–C8	112.86(10)
C14–C12–C8	111.26(10)
C11–C12–C8	102.38(9)
C12–C13–H13A	109.5
C12–C13–H13B	109.5
H13A–C13–H13B	109.5

C12–C13–H13C	109.5
H13A–C13–H13C	109.5
H13B–C13–H13C	109.5
C12–C14–H14A	109.5
C12–C14–H14B	109.5
H14A–C14–H14B	109.5
C12–C14–H14C	109.5
H14A–C14–H14C	109.5
H14B–C14–H14C	109.5
C7–N1–C6	111.71(10)
C7–N1–H6	121.1(10)
C6–N1–H6	127.1(10)
C9–O2–H1	109.3(11)

Table SI-22. Crystal data and structure refinement for compound **22**.

CCDC number	2532562
Empirical formula	$C_{14}H_{17}NO_2$
Formula weight	231.29
Temperature [K]	100(2)
Crystal system	monoclinic
Space group (number)	$P2_1/c$ (14)
a [Å]	10.7126(8)
b [Å]	26.1689(19)
c [Å]	13.8208(11)
α [°]	90
β [°]	109.458(3)
γ [°]	90
Volume [Å^3]	3653.2(5)
Z	12
ρ_{calc} [gcm^{-3}]	1.262
μ [mm^{-1}]	0.084
F(000)	1488
Crystal size [mm^3]	0.084×0.126×0.661
Crystal colour	colourless
Crystal shape	block
Radiation	MoK_α (λ=0.71073 Å)
2θ range [°]	4.32 to 52.76 (0.80 Å)
Index ranges	−13 ≤ h ≤ 13<br>−32 ≤ k ≤ 32<br>−17 ≤ l ≤ 17
Reflections collected	98734
Independent reflections	7480<br>R_{int} = 0.0684<br>R_{sigma} = 0.0282
Completeness to<br>θ = 25.242°	99.9
Data / Restraints / Parameters	7480 / 441 / 475
Goodness-of-fit on F^2	1.024
Final R indexes<br>[I≥2σ(I)]	R_1 = 0.0371<br>wR_2 = 0.0882
Final R indexes<br>[all data]	R_1 = 0.0493<br>wR_2 = 0.0962
Largest peak/hole [eÅ^{-3}]	0.29/−0.22

Table SI-23. Bond lengths and angles for compound **22**.

Atom–Atom	Length [Å]
O1–C6	1.3667(16)
O1–C7	1.4289(15)
O2–C7	1.4159(15)
O2–H2A	0.881(17)
O3–C20	1.3663(16)
O3–C21	1.4430(15)
O4–C21	1.3951(15)
O4–H4A	0.868(18)
O5–C34	1.3710(16)
O5–C35	1.4498(15)
O6–C35	1.4054(15)
O6–H6A	0.866(18)
N1–C14	1.2829(16)
N1–C1	1.4222(16)
N2–C28	1.2838(16)
N2–C15	1.4194(16)
N3–C42	1.2818(17)
N3–C29	1.4113(17)
C2–C3	1.3857(19)
C2–C1	1.3943(18)
C2–H2	0.9500
C1–C6	1.3934(18)
C3–C4	1.389(2)
C3–H3	0.9500
C4–C5	1.384(2)
C4–H4	0.9500
C5–C6	1.3870(18)
C5–H5	0.9500
C7–C8	1.5181(18)
C7–C14	1.5358(17)
C8–C9	1.5154(18)
C8–H8A	0.9900
C8–H8B	0.9900
C9–C10	1.5249(18)
C9–H9A	0.9900
C9–H9B	0.9900
C10–C11	1.5407(18)
C10–H10A	0.9900
C10–H10B	0.9900
C11–C12	1.5286(17)
C11–C14	1.5298(17)
C11–C13	1.5482(17)
C12–H12A	0.9800
C12–H12B	0.9800
C12–H12C	0.9800
C13–H13A	0.9800
C13–H13B	0.9800
C13–H13C	0.9800
C15–C20	1.3886(19)
C15–C16	1.3964(19)
C16–C17	1.385(2)
C16–H16	0.9500
C17–C18	1.387(2)
C17–H17	0.9500
C18–C19	1.386(2)
C18–H18	0.9500
C19–C20	1.3890(19)
C19–H19	0.9500
C21–C22	1.5169(17)
C21–C28	1.5355(17)
C22–C23	1.5191(18)
C22–H22A	0.9900
C22–H22B	0.9900
C23–C24	1.5231(18)
C23–H23A	0.9900
C23–H23B	0.9900
C24–C25	1.5415(17)
C24–H24A	0.9900
C24–H24B	0.9900
C25–C28	1.5249(17)
C25–C27	1.5292(18)
C25–C26	1.5450(18)
C26–H26A	0.9800
C26–H26B	0.9800
C26–H26C	0.9800
C27–H27A	0.9800
C27–H27B	0.9800
C27–H27C	0.9800
C29–C30	1.391(2)

C29–C34	1.3932(19)
C30–C31	1.385(2)
C30–H30	0.9500
C31–C32	1.385(2)
C31–H31	0.9500
C32–C33	1.393(2)
C32–H32	0.9500
C33–C34	1.3876(19)
C33–H33	0.9500
C35–C36	1.5212(18)
C35–C42	1.5345(18)
C36–C37	1.5230(18)
C36–H36A	0.9900
C36–H36B	0.9900
C37–C38	1.5255(18)
C37–H37A	0.9900
C37–H37B	0.9900
C38–C39	1.5440(18)
C38–H38A	0.9900
C38–H38B	0.9900
C39–C42	1.5257(18)
C39–C40	1.5315(18)
C39–C41	1.5425(18)
C40–H40A	0.9800
C40–H40B	0.9800
C40–H40C	0.9800
C41–H41A	0.9800
C41–H41B	0.9800
C41–H41C	0.9800

**Atom–Atom–Atom**	**Angle [°]**
C6–O1–C7	114.86(10)
C7–O2–H2A	110.9(10)
C20–O3–C21	115.44(10)
C21–O4–H4A	106.7(11)
C34–O5–C35	116.21(10)
C35–O6–H6A	108.8(11)
C14–N1–C1	117.53(11)
C28–N2–C15	117.97(11)
C42–N3–C29	118.08(12)
C3–C2–C1	120.14(13)
C3–C2–H2	119.9

C1–C2–H2	119.9
C6–C1–C2	118.64(12)
C6–C1–N1	120.84(12)
C2–C1–N1	120.44(12)
C2–C3–C4	120.37(13)
C2–C3–H3	119.8
C4–C3–H3	119.8
C5–C4–C3	120.24(13)
C5–C4–H4	119.9
C3–C4–H4	119.9
C4–C5–C6	119.07(13)
C4–C5–H5	120.5
C6–C5–H5	120.5
O1–C6–C5	118.72(12)
O1–C6–C1	119.74(11)
C5–C6–C1	121.50(12)
O2–C7–O1	110.33(10)
O2–C7–C8	113.18(10)
O1–C7–C8	104.12(10)
O2–C7–C14	102.93(9)
O1–C7–C14	110.61(10)
C8–C7–C14	115.81(10)
C9–C8–C7	112.02(10)
C9–C8–H8A	109.2
C7–C8–H8A	109.2
C9–C8–H8B	109.2
C7–C8–H8B	109.2
H8A–C8–H8B	107.9
C8–C9–C10	109.40(11)
C8–C9–H9A	109.8
C10–C9–H9A	109.8
C8–C9–H9B	109.8
C10–C9–H9B	109.8
H9A–C9–H9B	108.2
C9–C10–C11	113.91(11)
C9–C10–H10A	108.8
C11–C10–H10A	108.8
C9–C10–H10B	108.8
C11–C10–H10B	108.8
H10A–C10–H10B	107.7
C12–C11–C14	110.45(10)
C12–C11–C10	108.09(10)

C14–C11–C10	110.80(10)
C12–C11–C13	109.64(10)
C14–C11–C13	107.92(10)
C10–C11–C13	109.95(10)
C11–C12–H12A	109.5
C11–C12–H12B	109.5
H12A–C12–H12B	109.5
C11–C12–H12C	109.5
H12A–C12–H12C	109.5
H12B–C12–H12C	109.5
C11–C13–H13A	109.5
C11–C13–H13B	109.5
H13A–C13–H13B	109.5
C11–C13–H13C	109.5
H13A–C13–H13C	109.5
H13B–C13–H13C	109.5
N1–C14–C11	120.76(11)
N1–C14–C7	120.76(11)
C11–C14–C7	118.04(11)
C20–C15–C16	119.25(12)
C20–C15–N2	120.87(12)
C16–C15–N2	119.84(12)
C17–C16–C15	120.00(14)
C17–C16–H16	120.0
C15–C16–H16	120.0
C16–C17–C18	119.97(13)
C16–C17–H17	120.0
C18–C17–H17	120.0
C19–C18–C17	120.73(13)
C19–C18–H18	119.6
C17–C18–H18	119.6
C18–C19–C20	118.97(14)
C18–C19–H19	120.5
C20–C19–H19	120.5
O3–C20–C15	119.73(11)
O3–C20–C19	119.08(12)
C15–C20–C19	121.03(13)
O4–C21–O3	109.96(10)
O4–C21–C22	113.84(10)
O3–C21–C22	103.65(10)
O4–C21–C28	105.65(10)
O3–C21–C28	110.83(10)

C22–C21–C28	113.00(10)
C21–C22–C23	111.70(11)
C21–C22–H22A	109.3
C23–C22–H22A	109.3
C21–C22–H22B	109.3
C23–C22–H22B	109.3
H22A–C22–H22B	107.9
C22–C23–C24	109.51(10)
C22–C23–H23A	109.8
C24–C23–H23A	109.8
C22–C23–H23B	109.8
C24–C23–H23B	109.8
H23A–C23–H23B	108.2
C23–C24–C25	113.85(11)
C23–C24–H24A	108.8
C25–C24–H24A	108.8
C23–C24–H24B	108.8
C25–C24–H24B	108.8
H24A–C24–H24B	107.7
C28–C25–C27	111.68(10)
C28–C25–C24	110.80(10)
C27–C25–C24	108.41(10)
C28–C25–C26	107.01(10)
C27–C25–C26	108.77(11)
C24–C25–C26	110.15(10)
C25–C26–H26A	109.5
C25–C26–H26B	109.5
H26A–C26–H26B	109.5
C25–C26–H26C	109.5
H26A–C26–H26C	109.5
H26B–C26–H26C	109.5
C25–C27–H27A	109.5
C25–C27–H27B	109.5
H27A–C27–H27B	109.5
C25–C27–H27C	109.5
H27A–C27–H27C	109.5
H27B–C27–H27C	109.5
N2–C28–C25	120.28(11)
N2–C28–C21	121.55(11)
C25–C28–C21	117.97(10)
C30–C29–C34	119.22(13)
C30–C29–N3	119.56(13)

C34–C29–N3	121.18(12)
C31–C30–C29	120.18(15)
C31–C30–H30	119.9
C29–C30–H30	119.9
C30–C31–C32	120.07(14)
C30–C31–H31	120.0
C32–C31–H31	120.0
C31–C32–C33	120.64(14)
C31–C32–H32	119.7
C33–C32–H32	119.7
C34–C33–C32	118.80(14)
C34–C33–H33	120.6
C32–C33–H33	120.6
O5–C34–C33	119.10(13)
O5–C34–C29	119.70(12)
C33–C34–C29	121.08(13)
O6–C35–O5	109.26(10)
O6–C35–C36	113.32(11)
O5–C35–C36	104.39(10)
O6–C35–C42	106.16(10)
O5–C35–C42	110.45(10)
C36–C35–C42	113.28(10)
C35–C36–C37	111.90(11)
C35–C36–H36A	109.2
C37–C36–H36A	109.2
C35–C36–H36B	109.2
C37–C36–H36B	109.2
H36A–C36–H36B	107.9
C36–C37–C38	109.72(11)
C36–C37–H37A	109.7
C38–C37–H37A	109.7
C36–C37–H37B	109.7
C38–C37–H37B	109.7
H37A–C37–H37B	108.2
C37–C38–C39	113.30(11)
C37–C38–H38A	108.9
C39–C38–H38A	108.9
C37–C38–H38B	108.9
C39–C38–H38B	108.9
H38A–C38–H38B	107.7
C42–C39–C40	110.18(11)
C42–C39–C41	107.91(10)

C40–C39–C41	108.57(11)
C42–C39–C38	110.34(10)
C40–C39–C38	109.08(11)
C41–C39–C38	110.73(11)
C39–C40–H40A	109.5
C39–C40–H40B	109.5
H40A–C40–H40B	109.5
C39–C40–H40C	109.5
H40A–C40–H40C	109.5
H40B–C40–H40C	109.5
C39–C41–H41A	109.5
C39–C41–H41B	109.5
H41A–C41–H41B	109.5
C39–C41–H41C	109.5
H41A–C41–H41C	109.5
H41B–C41–H41C	109.5
N3–C42–C39	118.82(11)
N3–C42–C35	122.55(11)
C39–C42–C35	118.51(11)

Table SI-24. Crystal data and structure refinement for compound **10b**.

CCDC number	2532554
Empirical formula	$C_{16}H_{19}N_4O_2^{\bullet}$
Formula weight	299.35
Temperature [K]	100(2)
Crystal system	triclinic
Space group (number)	$P\bar{1}$ (2)
a [Å]	6.8022(11)
b [Å]	10.3308(18)
c [Å]	10.5981(18)
α [°]	71.084(6)
β [°]	87.921(6)
γ [°]	86.264(6)
Volume [$Å^3$]	702.9(2)
Z	2
ρ_{calc} [gcm^{-3}]	1.414
μ [mm^{-1}]	0.097
F(000)	318
Crystal size [mm^3]	0.110×0.534×0.559
Crystal colour	dark red
Crystal shape	plate
Radiation	MoK_{α} (λ=0.71073 Å)
2θ range [°]	4.79 to 50.88 (0.83 Å)
Index ranges	$-8 \leq h \leq 8$<br>$-11 \leq k \leq 12$<br>$0 \leq l \leq 12$
Reflections collected	5558
Independent reflections	5558<br>R_{int} = 0.0701<br>R_{sigma} = 0.0624
Completeness to<br>θ = 25.242°	99.6
Data / Restraints / Parameters	5558 / 264 / 240
Goodness-of-fit on F^2	1.075
Final *R* indexes<br>[$I \geq 2\sigma(I)$]	R_1 = 0.0914<br>wR_2 = 0.1955
Final *R* indexes<br>[all data]	R_1 = 0.1372<br>wR_2 = 0.2216
Largest peak/hole [$eÅ^{-3}$]	0.46/−0.32

Table SI-25. Bond lengths and angles for compound **10b**.

**Atom–Atom**	**Length [Å]**
C10–N4	1.322(5)
C10–N3	1.395(5)
C10–C5	1.401(6)
C11–N4	1.380(5)
C11–C12	1.397(6)
C11–C16	1.411(6)
C12–C13	1.381(6)
C13–C14	1.397(6)
C14–C15	1.386(6)
C15–C16	1.402(6)
C16–N1A	1.400(18)
C16–N1	1.407(8)
C4–C3	1.526(11)
C4–C3A	1.53(2)
C5–N1	1.399(8)
C5–N1A	1.410(18)
C5–C6	1.447(6)
N2–C8	1.388(5)
N2–C6	1.397(5)
N2–C7	1.465(5)
N3–C8	1.382(5)
N3–C9	1.463(5)
C6–O1	1.223(5)
C8–O2	1.213(5)
C1–N1	1.483(10)
C1–C2	1.511(9)
C2–C3	1.528(14)
C1A–N1A	1.487(19)
C1A–C2A	1.501(17)
C2A–C3A	1.52(2)

**Atom–Atom–Atom**	**Angle [°]**
N4–C10–N3	115.9(4)
N4–C10–C5	125.1(4)
N3–C10–C5	118.9(4)
N4–C11–C12	117.4(4)
N4–C11–C16	123.3(4)
C12–C11–C16	119.3(4)
C13–C12–C11	121.6(4)
C12–C13–C14	118.7(4)
C15–C14–C13	121.2(4)
C14–C15–C16	120.0(4)
N1A–C16–C15	123.2(8)
C15–C16–N1	122.6(5)
N1A–C16–C11	116.3(9)
C15–C16–C11	119.2(4)
N1–C16–C11	117.9(5)
N1–C5–C10	118.2(5)
C10–C5–N1A	116.7(8)
N1–C5–C6	121.3(5)
C10–C5–C6	120.3(4)
N1A–C5–C6	121.8(8)
C8–N2–C6	126.4(3)
C8–N2–C7	117.5(4)
C6–N2–C7	116.1(3)
C8–N3–C10	123.9(3)
C8–N3–C9	115.3(3)
C10–N3–C9	120.7(3)
C10–N4–C11	116.2(3)
O1–C6–N2	118.6(4)
O1–C6–C5	126.2(4)
N2–C6–C5	115.2(4)
O2–C8–N3	121.7(4)
O2–C8–N2	123.2(4)
N3–C8–N2	115.1(4)
N1–C1–C2	115.0(7)
C1–C2–C3	111.4(9)
C4–C3–C2	113.4(9)
C5–N1–C16	118.2(6)
C5–N1–C1	121.6(6)
C16–N1–C1	120.0(6)
N1A–C1A–C2A	117.6(19)
C1A–C2A–C3A	111(3)
C2A–C3A–C4	114.1(19)
C16–N1A–C5	117.9(14)
C16–N1A–C1A	117.9(13)
C5–N1A–C1A	123.5(13)

9 NMR Data

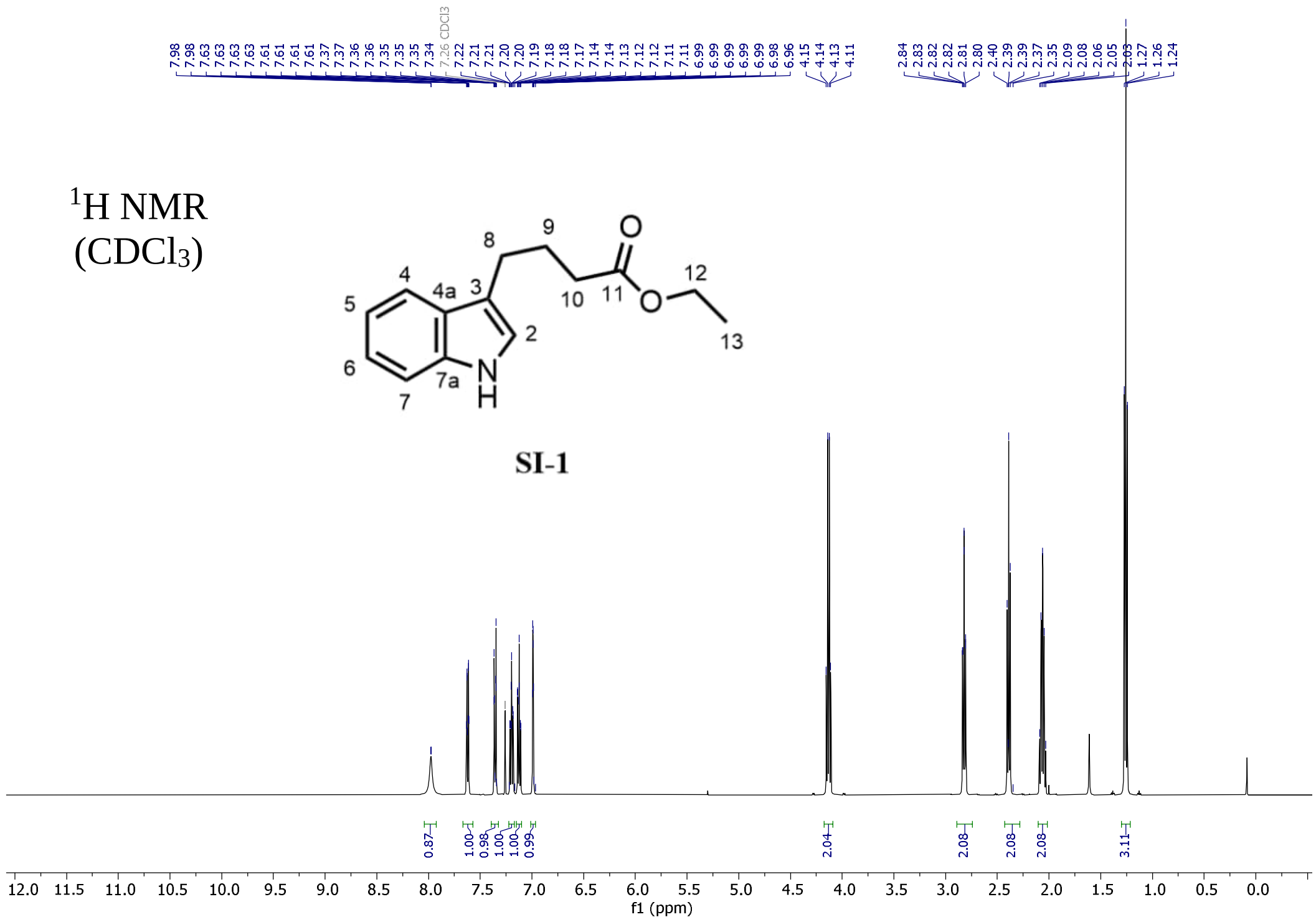

^{1}H NMR
($CDCl_3$)
SI-1
f1 (ppm)

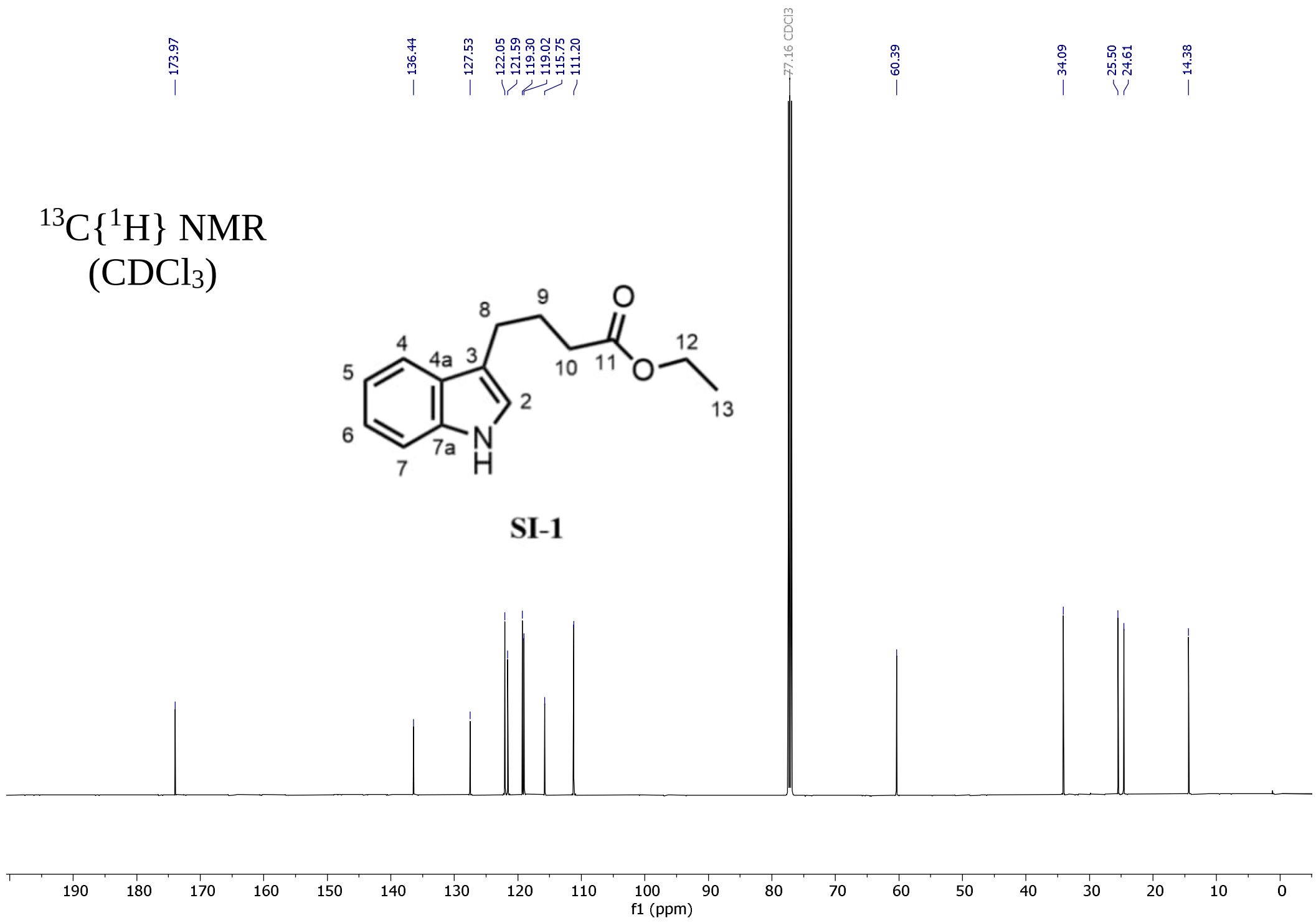

^{13}C{^{1}H} NMR
($CDCl_3$)
SI-1
173.97
136.44
127.53
122.05
121.59
119.30
119.02
115.75
111.20
77.16 CDCl3
60.39
34.09
25.50
24.61
14.38
f1 (ppm)

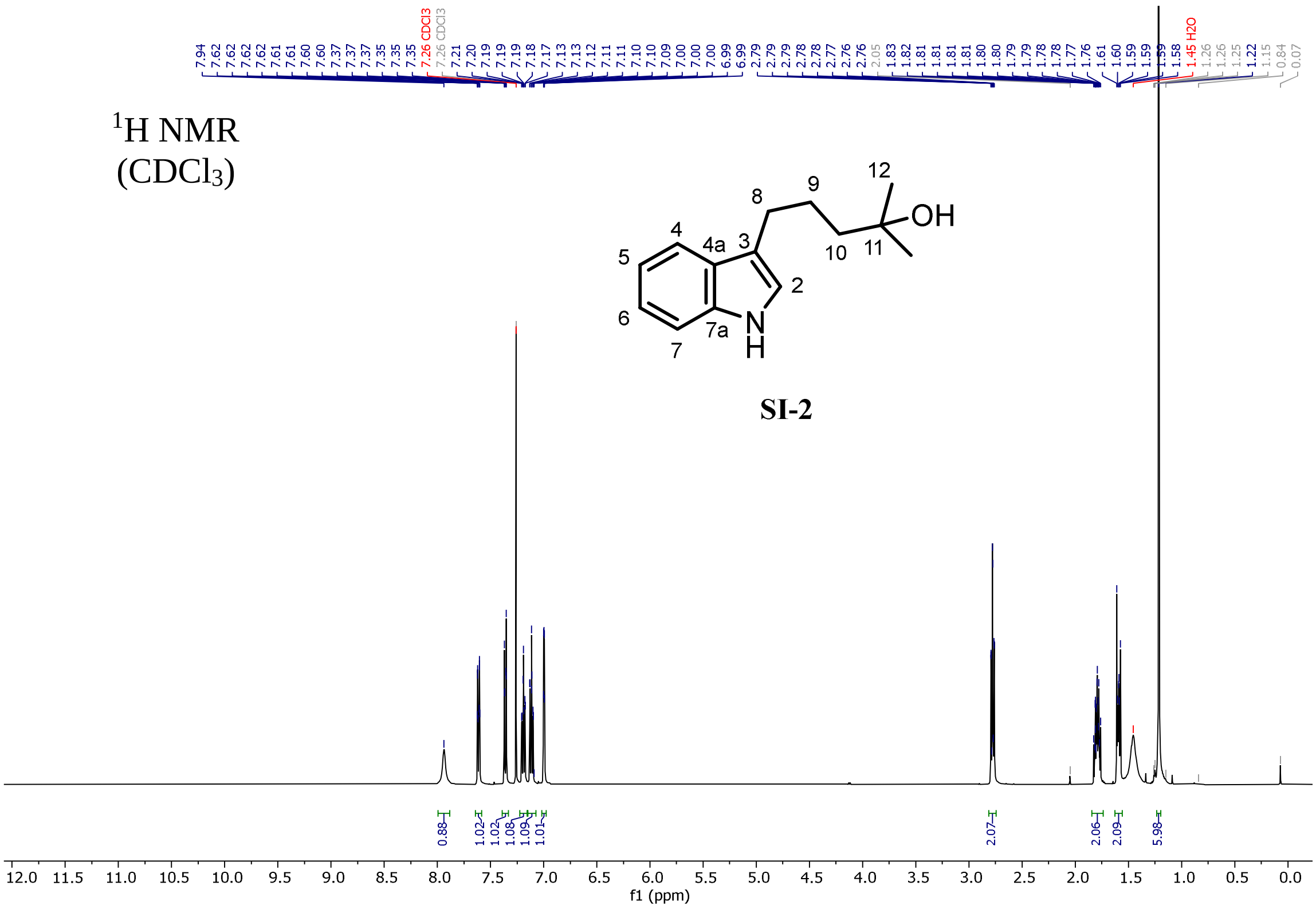

¹H NMR
(CDCl3)
SI-2
f1 (ppm)

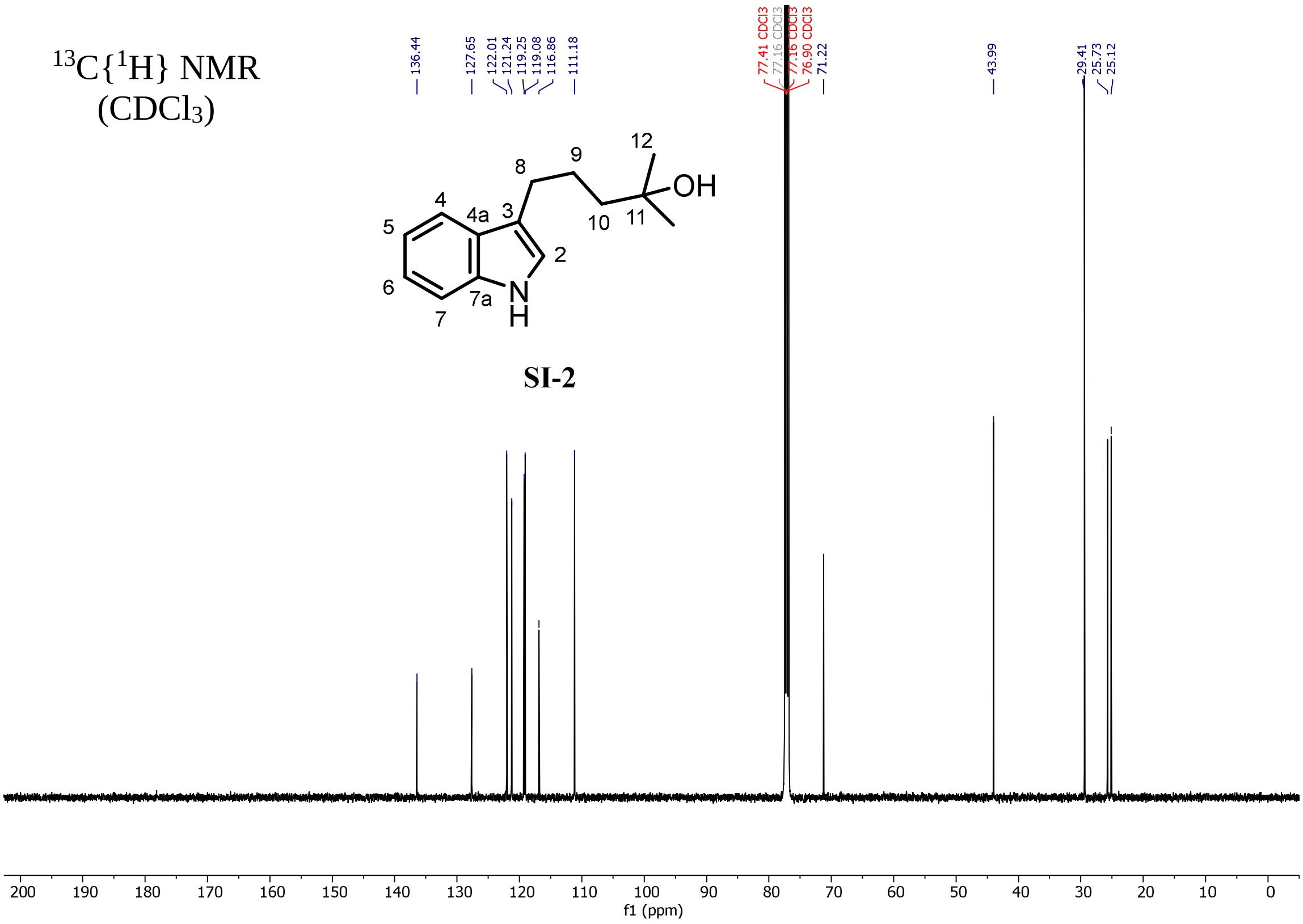

13C{1H} NMR
(CDCl3)
136.44
127.65
122.01
121.24
119.25
119.08
116.86
111.18
77.41 CDCl3
77.16 CDCl3
76.90 CDCl3
71.22
43.99
29.41
25.73
25.12
SI-2
f1 (ppm)

^1H NMR ($CDCl_3$)

3a

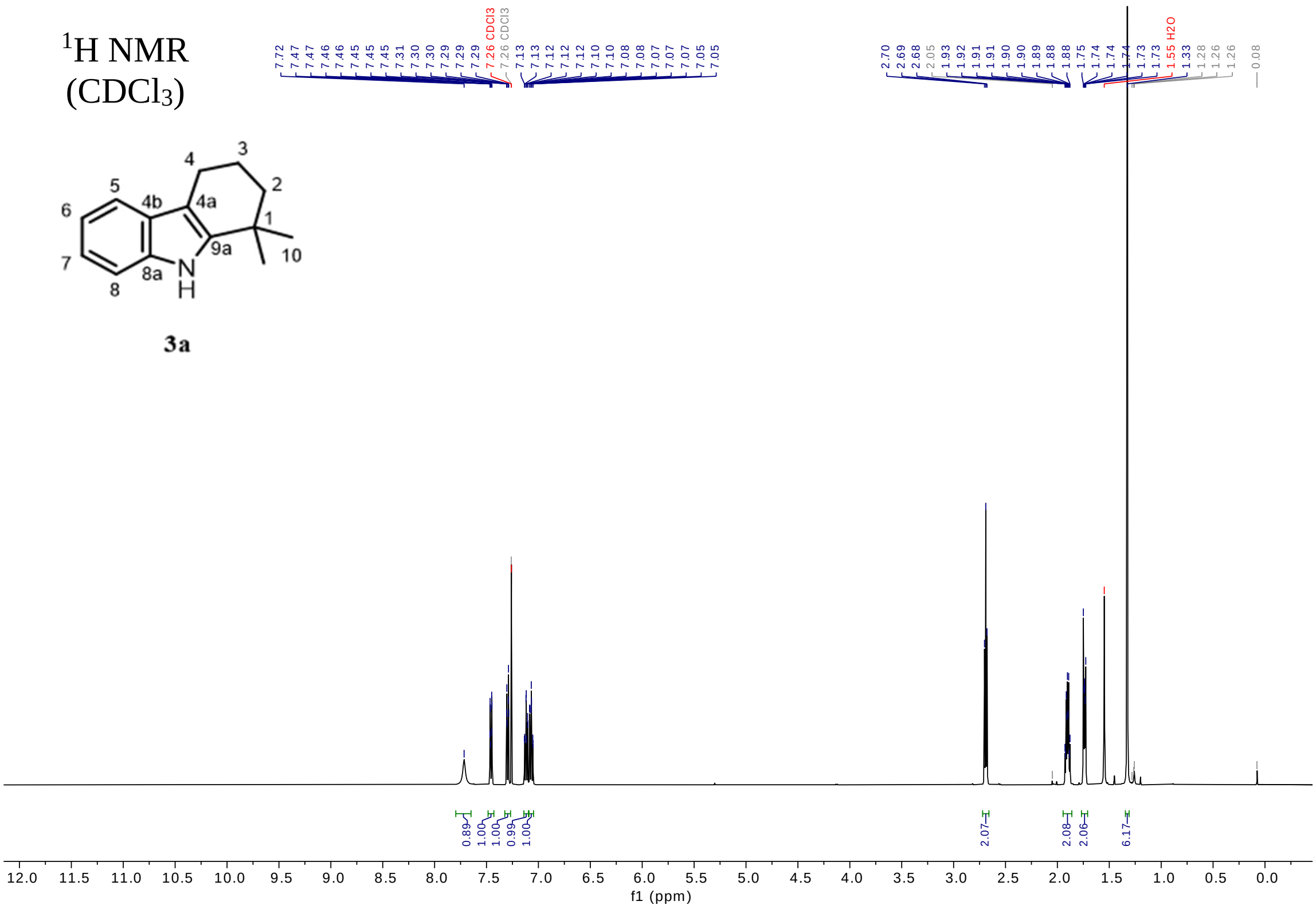

$^{13}C\{^1H\}$ NMR ($CDCl_3$)

3a

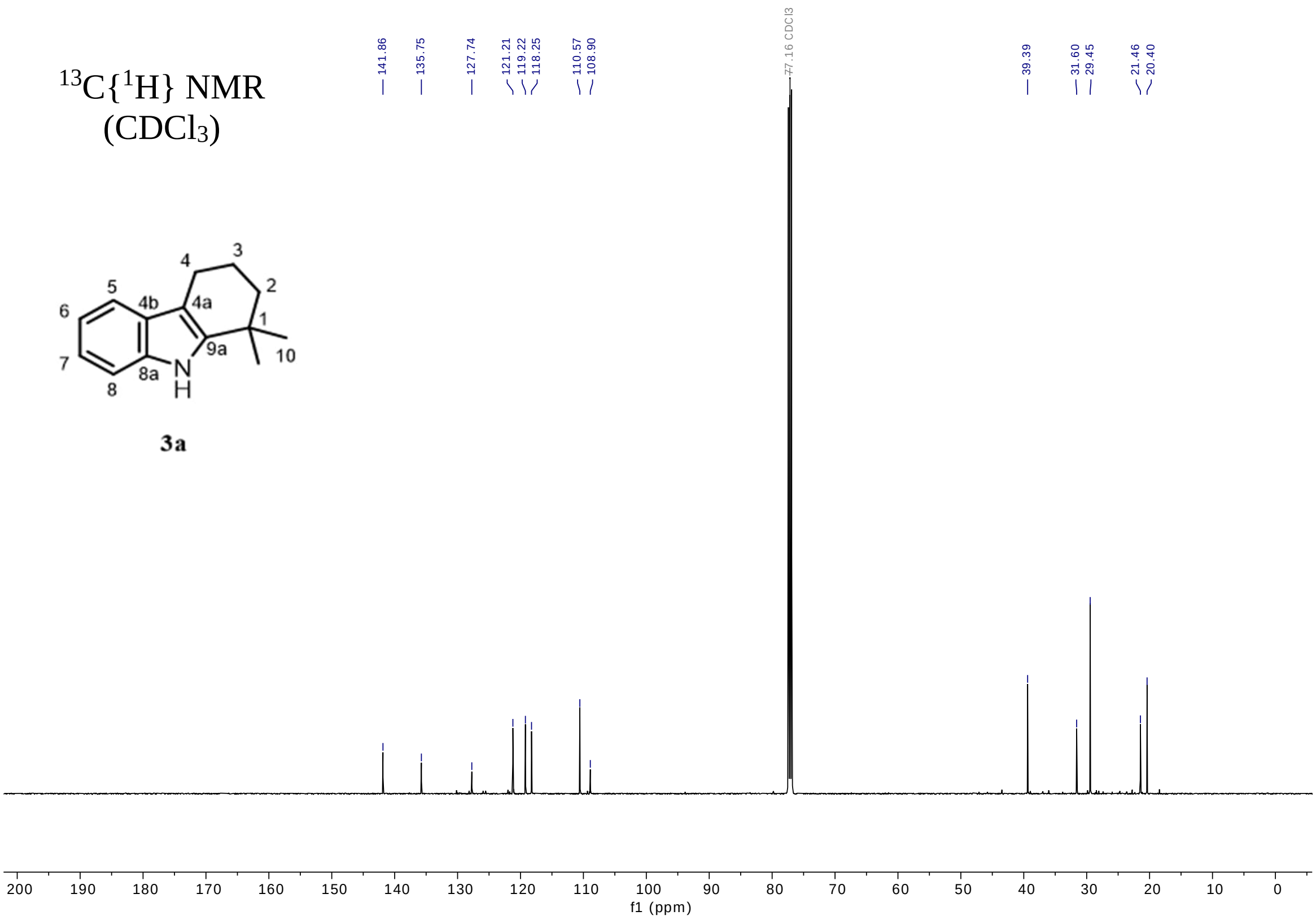

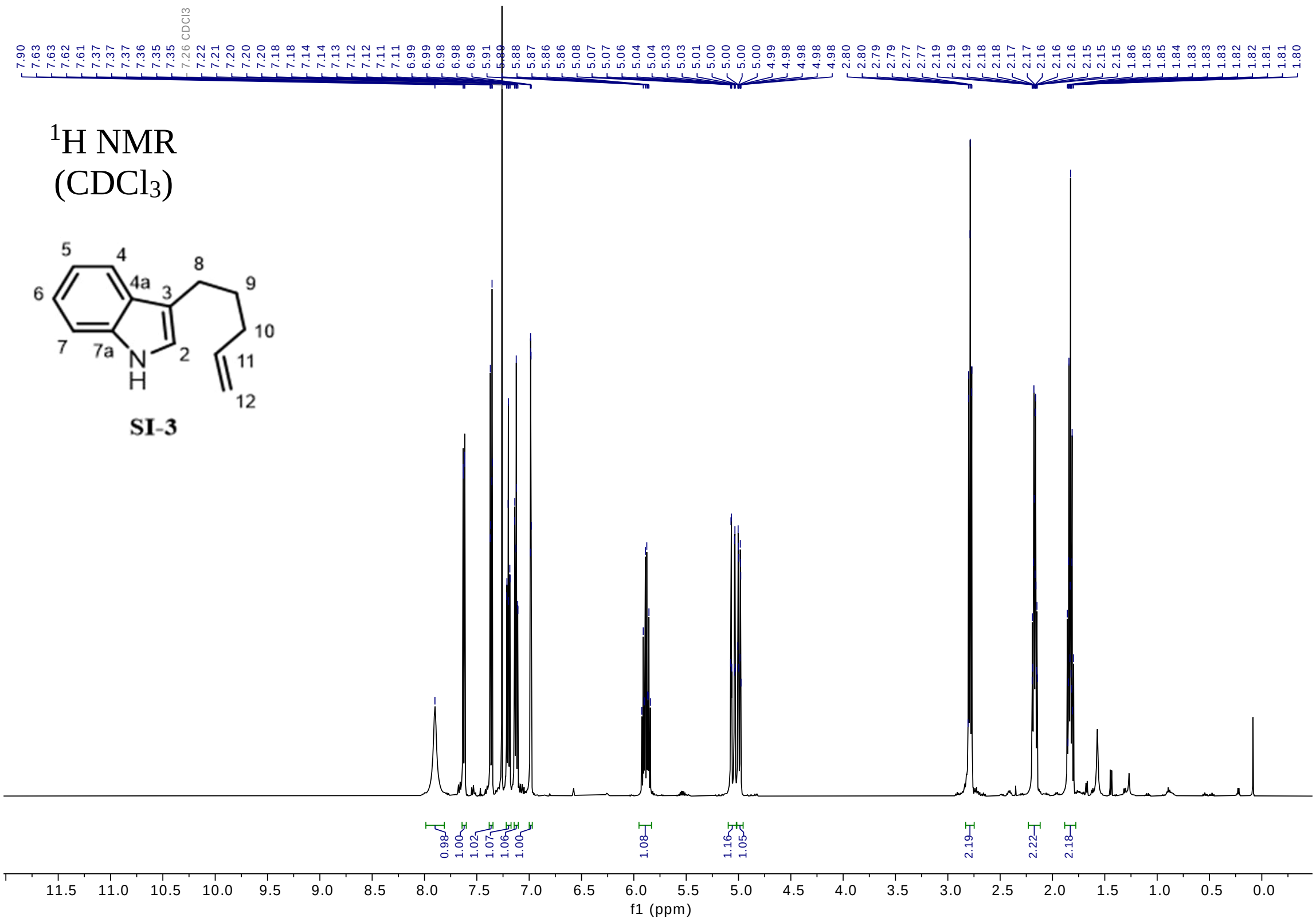
1H NMR
(CDCl3)
SI-3
0.98
1.00
1.02
1.07
1.06
1.00
1.08
1.16
1.05
2.19
2.22
2.18
f1 (ppm)

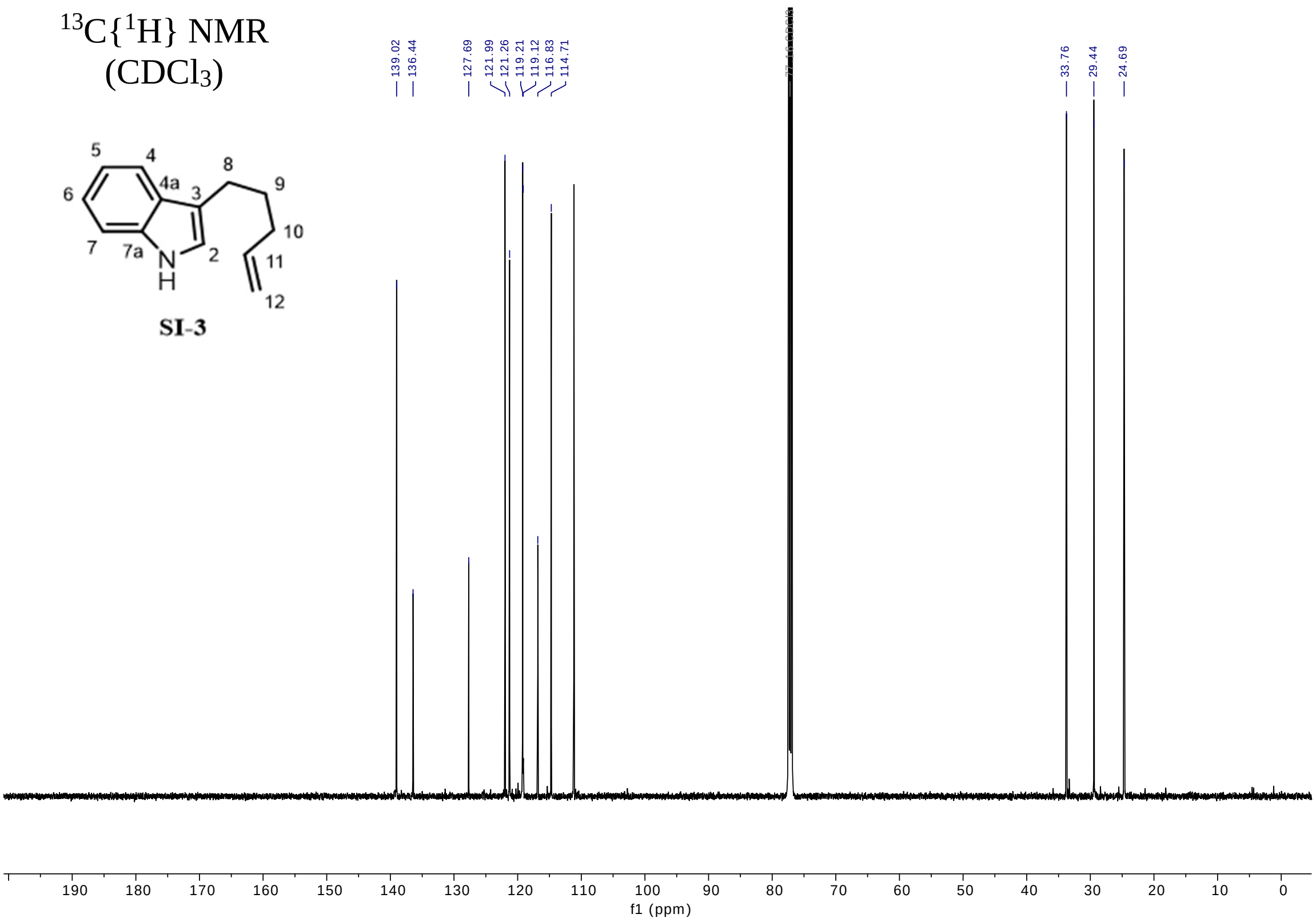
13C{1H} NMR
(CDCl3)
SI-3
139.02
136.44
127.69
121.99
121.26
119.21
119.12
116.83
114.71
33.76
29.44
24.69
f1 (ppm)

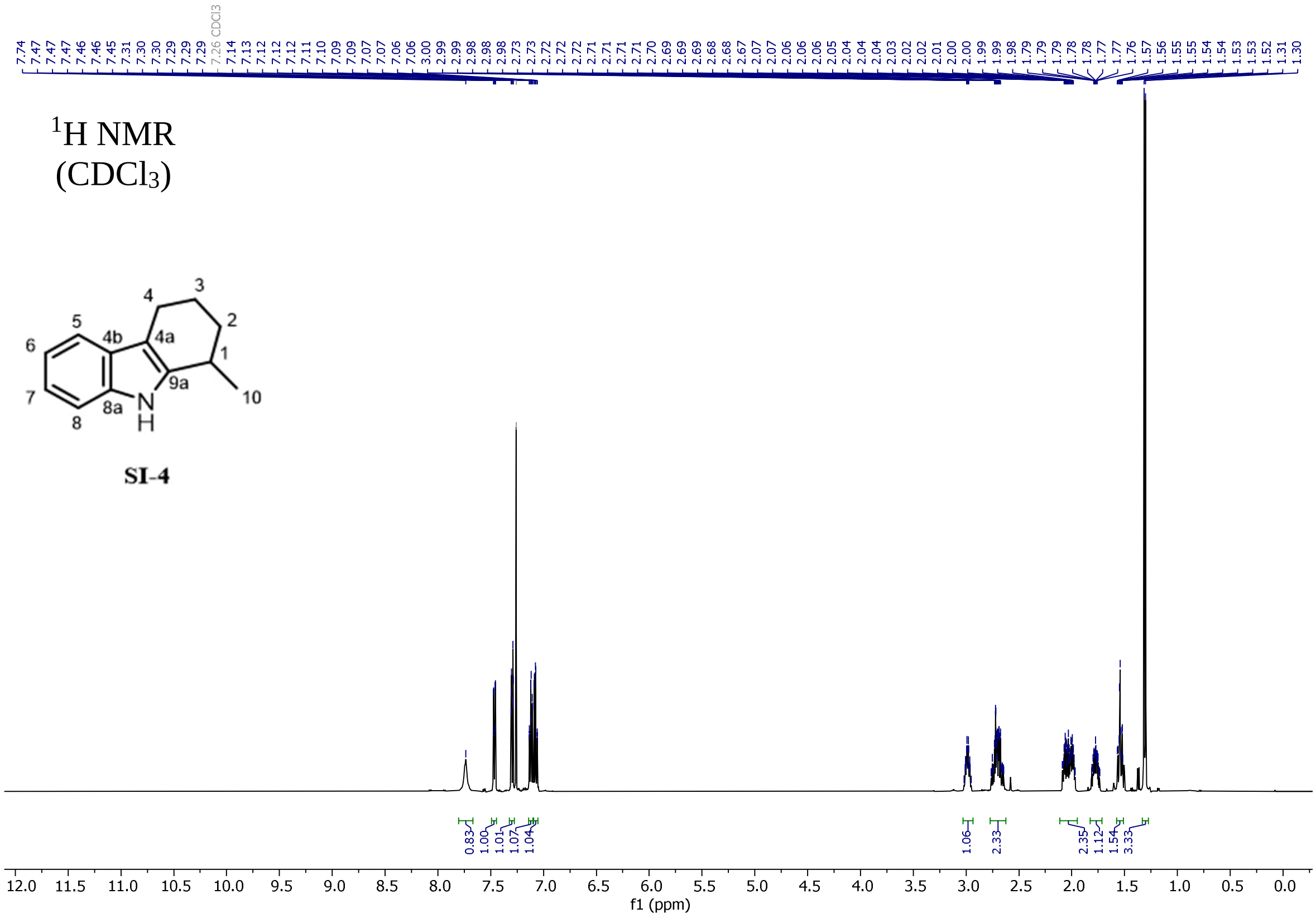

7.74
7.47
7.47
7.47
7.46
7.46
7.45
7.31
7.30
7.30
7.29
7.29
7.29
7.26 CDCl3
7.14
7.13
7.12
7.12
7.12
7.11
7.10
7.09
7.09
7.07
7.07
7.06
7.06
3.00
2.99
2.99
2.98
2.98
2.98
2.73
2.73
2.72
2.72
2.72
2.71
2.71
2.71
2.71
2.70
2.69
2.69
2.69
2.68
2.68
2.67
2.07
2.07
2.06
2.06
2.06
2.05
2.04
2.04
2.04
2.03
2.02
2.02
2.01
2.00
2.00
1.99
1.99
1.98
1.79
1.79
1.79
1.78
1.78
1.77
1.77
1.76
1.57
1.56
1.55
1.55
1.54
1.54
1.53
1.53
1.52
1.31
1.30
^{1}H NMR
(CDCl$_3$)
0.83
1.00
1.01
1.07
1.04
1.06
2.33
2.35
1.12
1.54
3.33
f1 (ppm)
4
3
5
4b
4a
2
6
1
9a
7
8a
N
H
10
8

SI-4

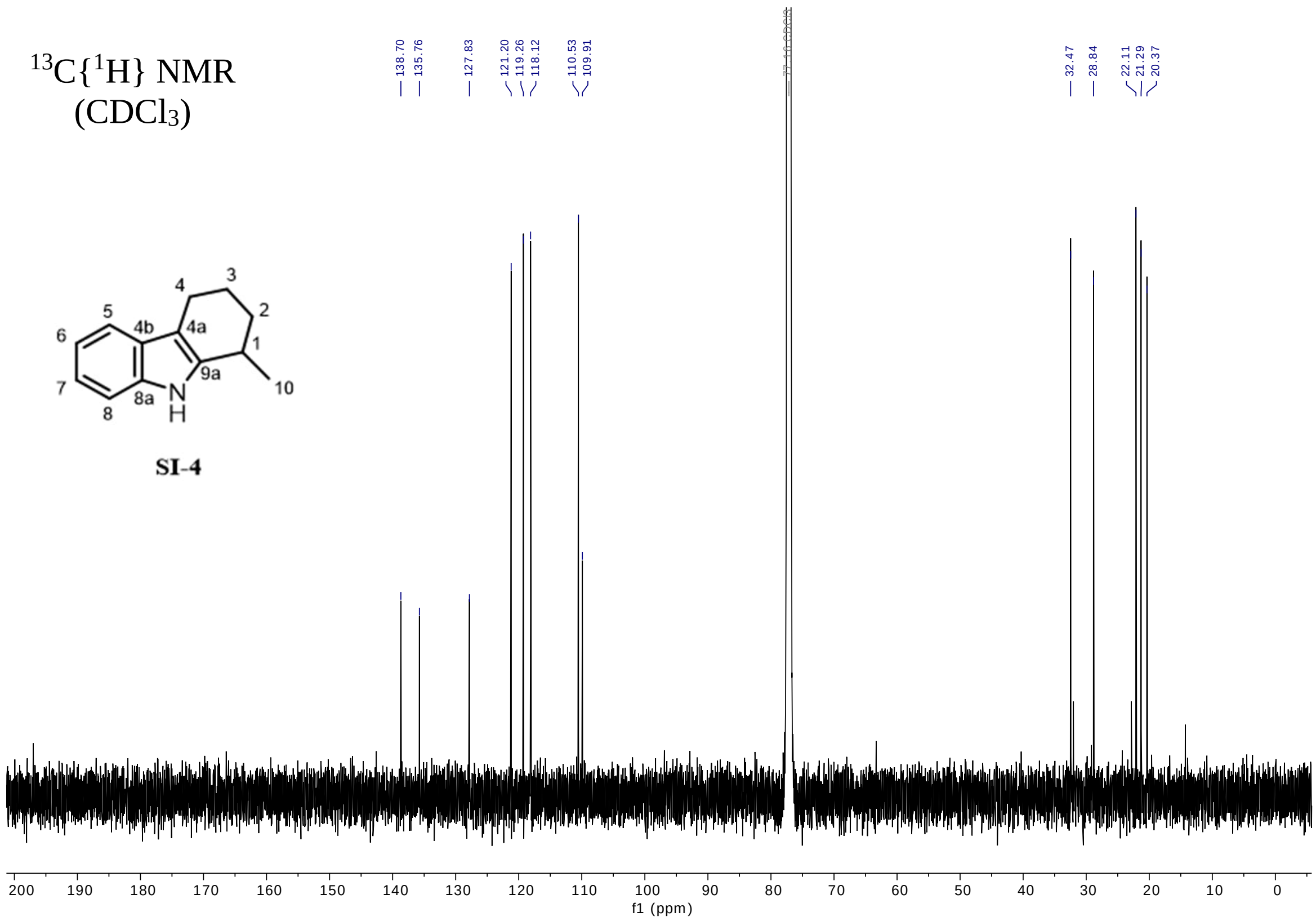

^{13}C{^{1}H} NMR
(CDCl$_3$)
138.70
135.76
127.83
121.20
119.26
118.12
110.53
109.91
32.47
28.84
22.11
21.29
20.37
f1 (ppm)
4
3
5
4b
4a
2
6
1
9a
7
8a
N
H
10
8

SI-4

^{1}H NMR
($CDCl_3$)

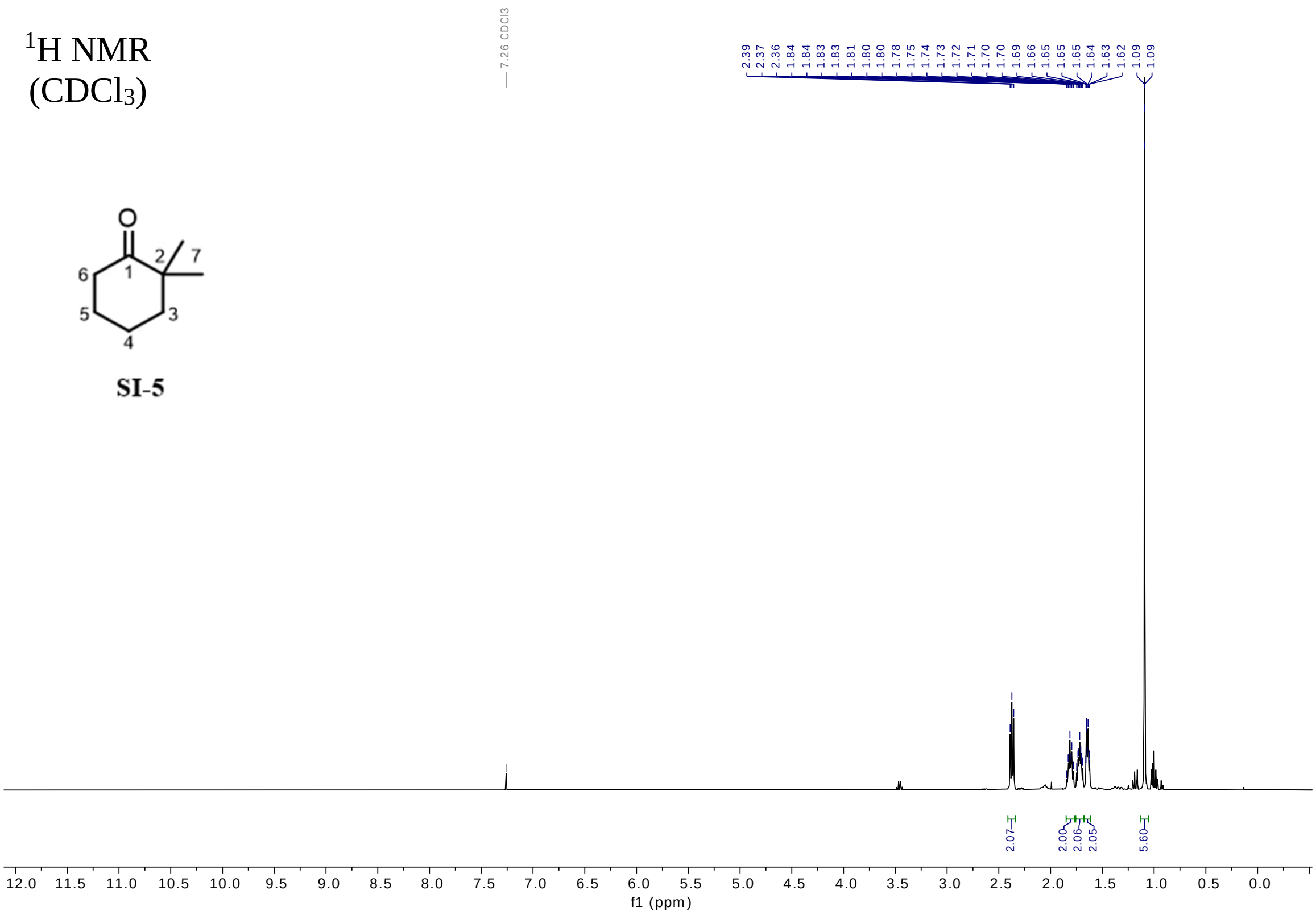

SI-5

^{13}C{^{1}H} NMR
($CDCl_3$)

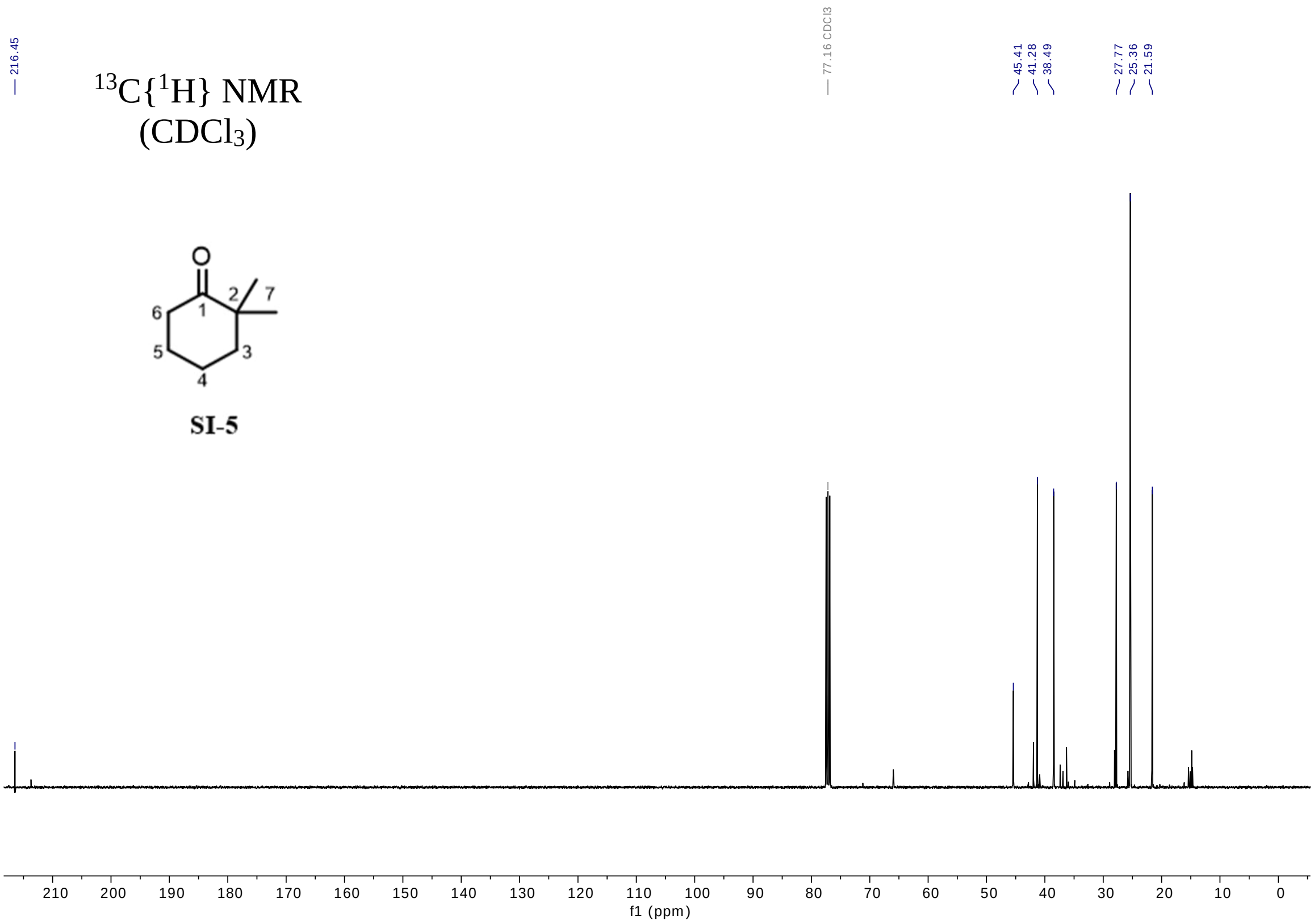

SI-5

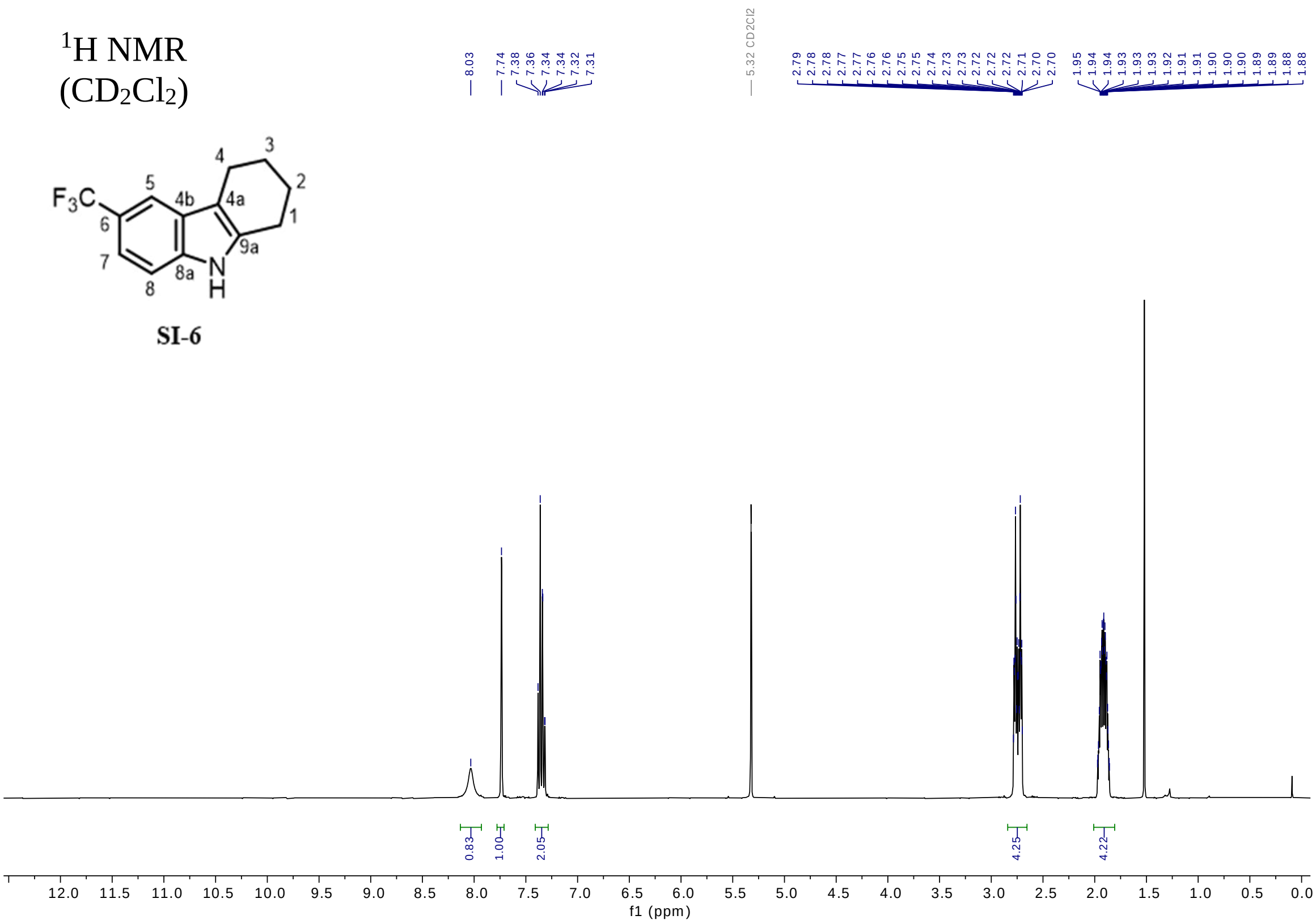

1H NMR
(CD2Cl2)
F3C
SI-6
8.03
7.74
7.38
7.36
7.34
7.34
7.32
7.31
5.32 CD2Cl2
2.79
2.78
2.78
2.77
2.77
2.76
2.76
2.75
2.75
2.74
2.73
2.73
2.72
2.72
2.72
2.71
2.70
2.70
1.95
1.94
1.94
1.93
1.93
1.93
1.92
1.91
1.91
1.90
1.90
1.90
1.89
1.89
1.88
1.88
0.83
1.00
2.05
4.25
4.22
f1 (ppm)

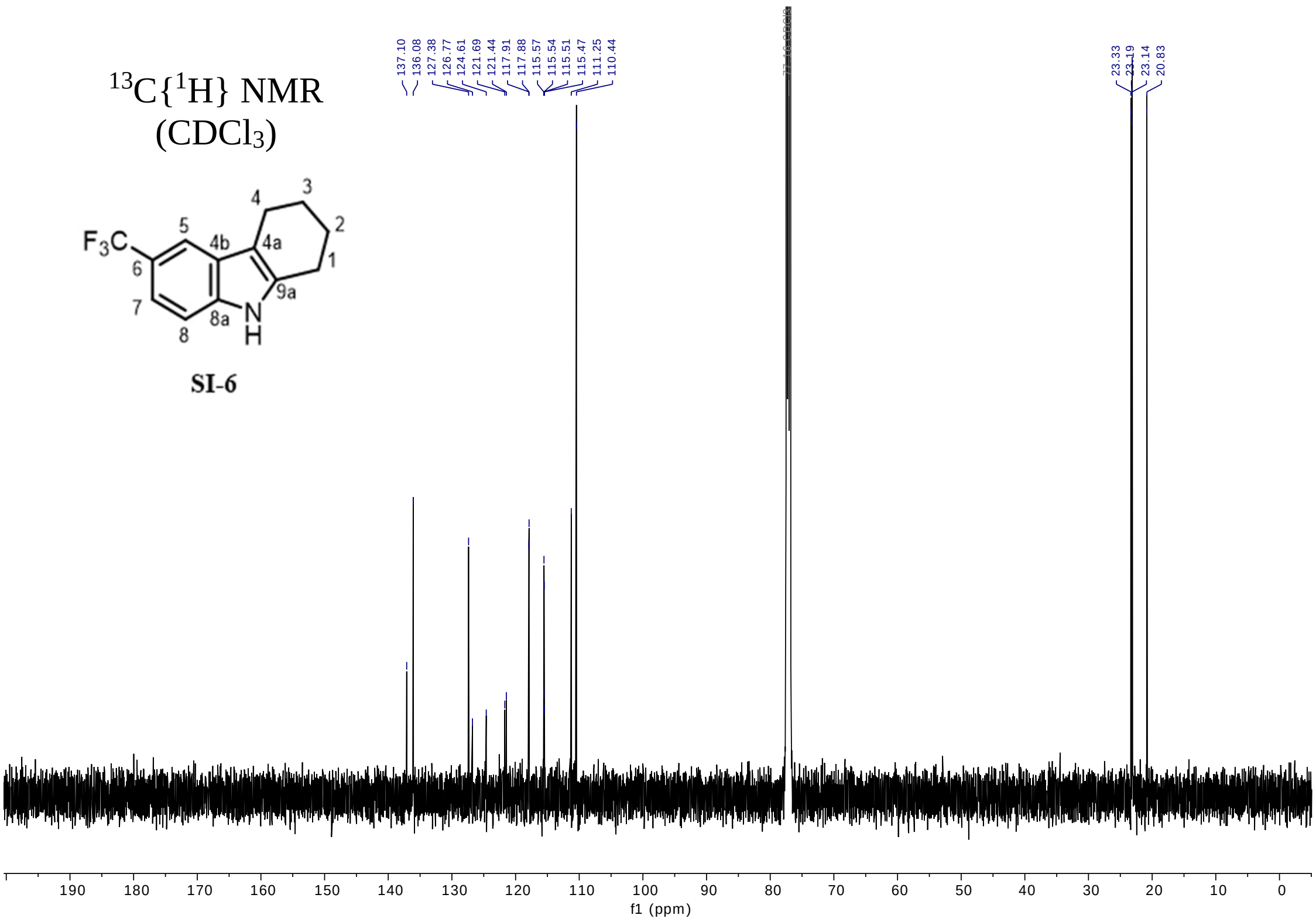

13C{1H} NMR
(CDCl3)
F3C
SI-6
137.10
136.08
127.38
126.77
124.61
121.69
121.44
117.91
117.88
115.57
115.54
115.51
115.47
111.25
110.44
23.33
23.19
23.14
20.83
f1 (ppm)

^{19}F NMR
($CDCl_3$)

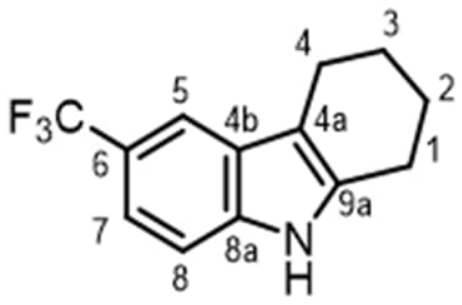

SI-6

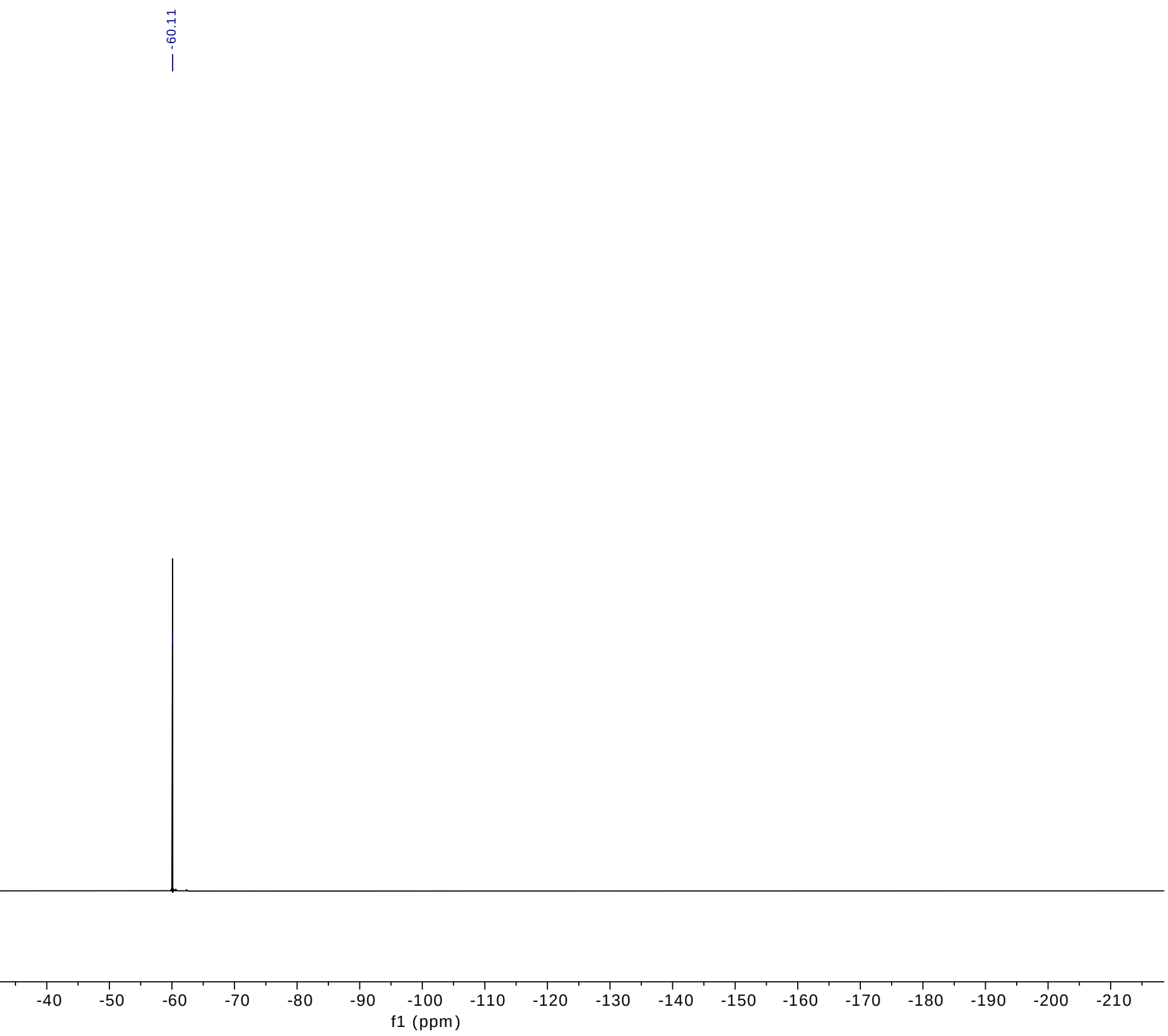

^{1}H NMR
($CDCl_3$)

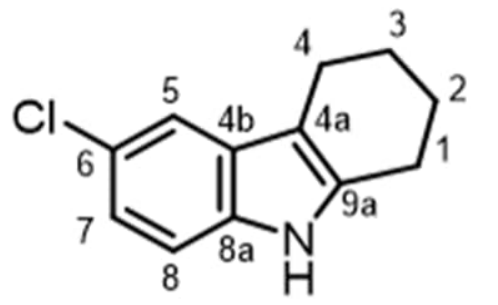

SI-7

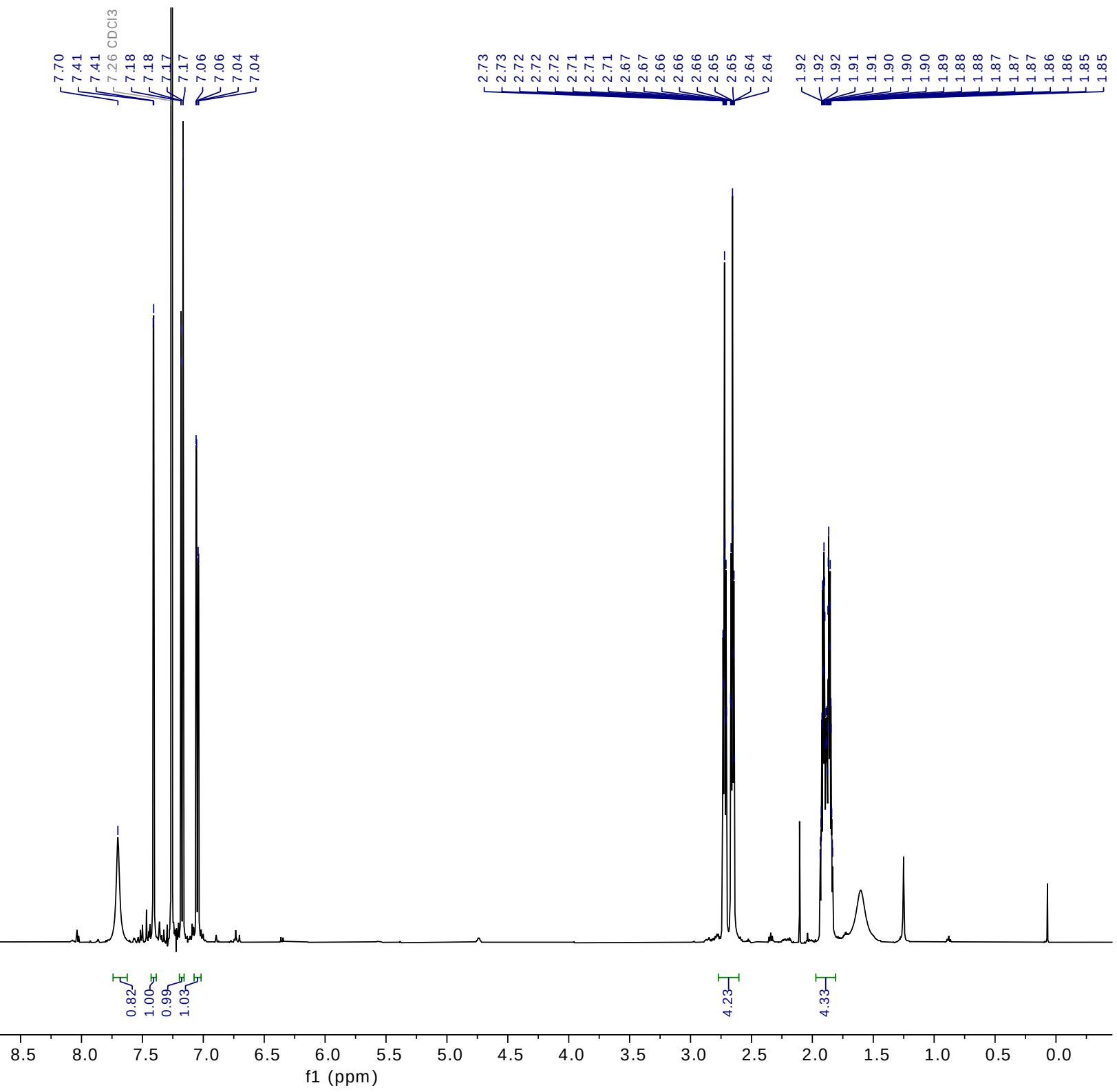

$^{13}C\{^{1}H\}$ NMR
($CDCl_3$)

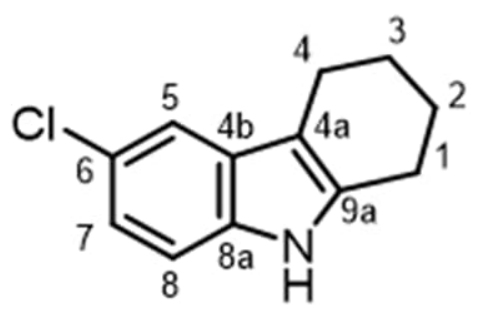

SI-7

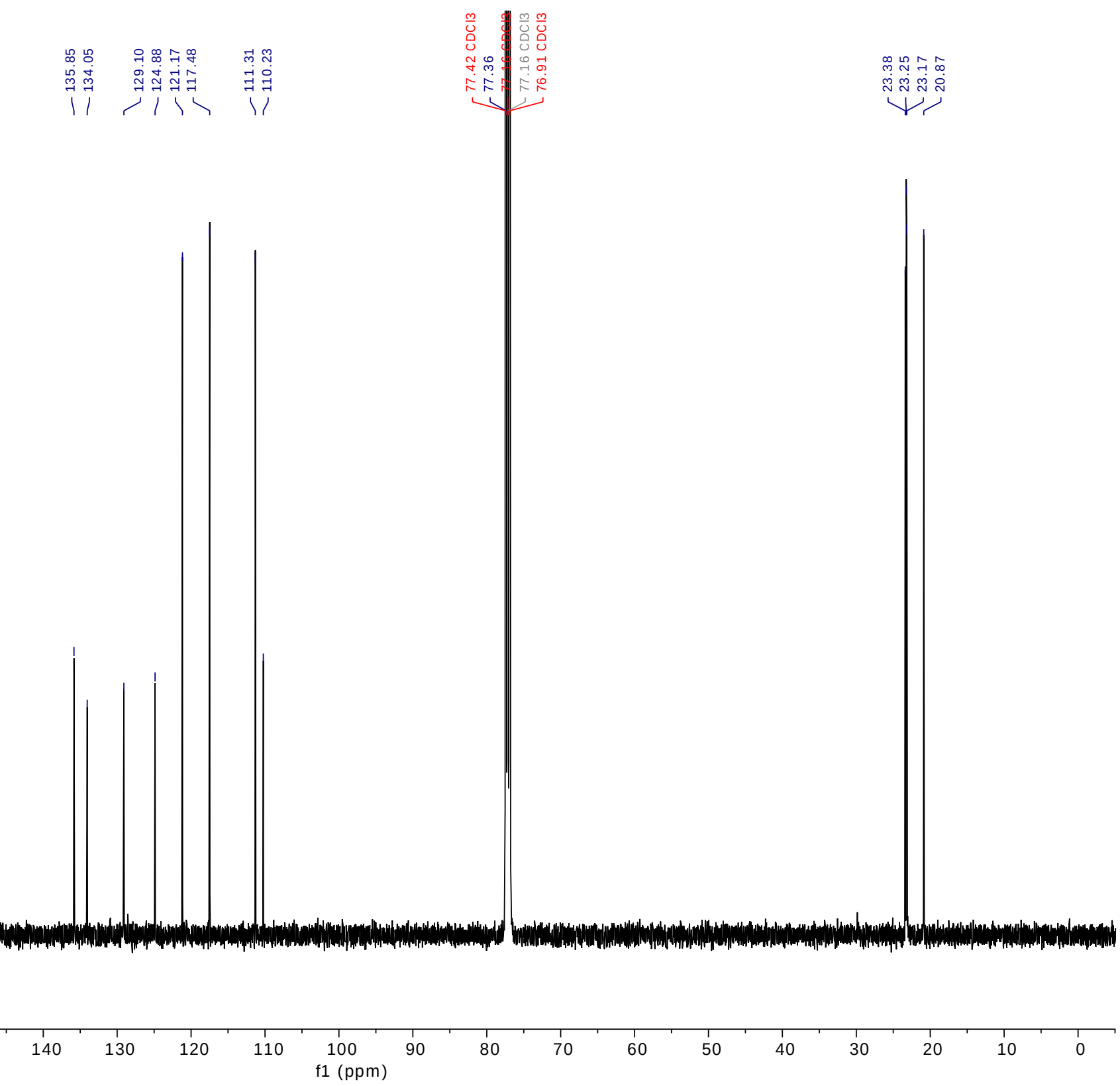

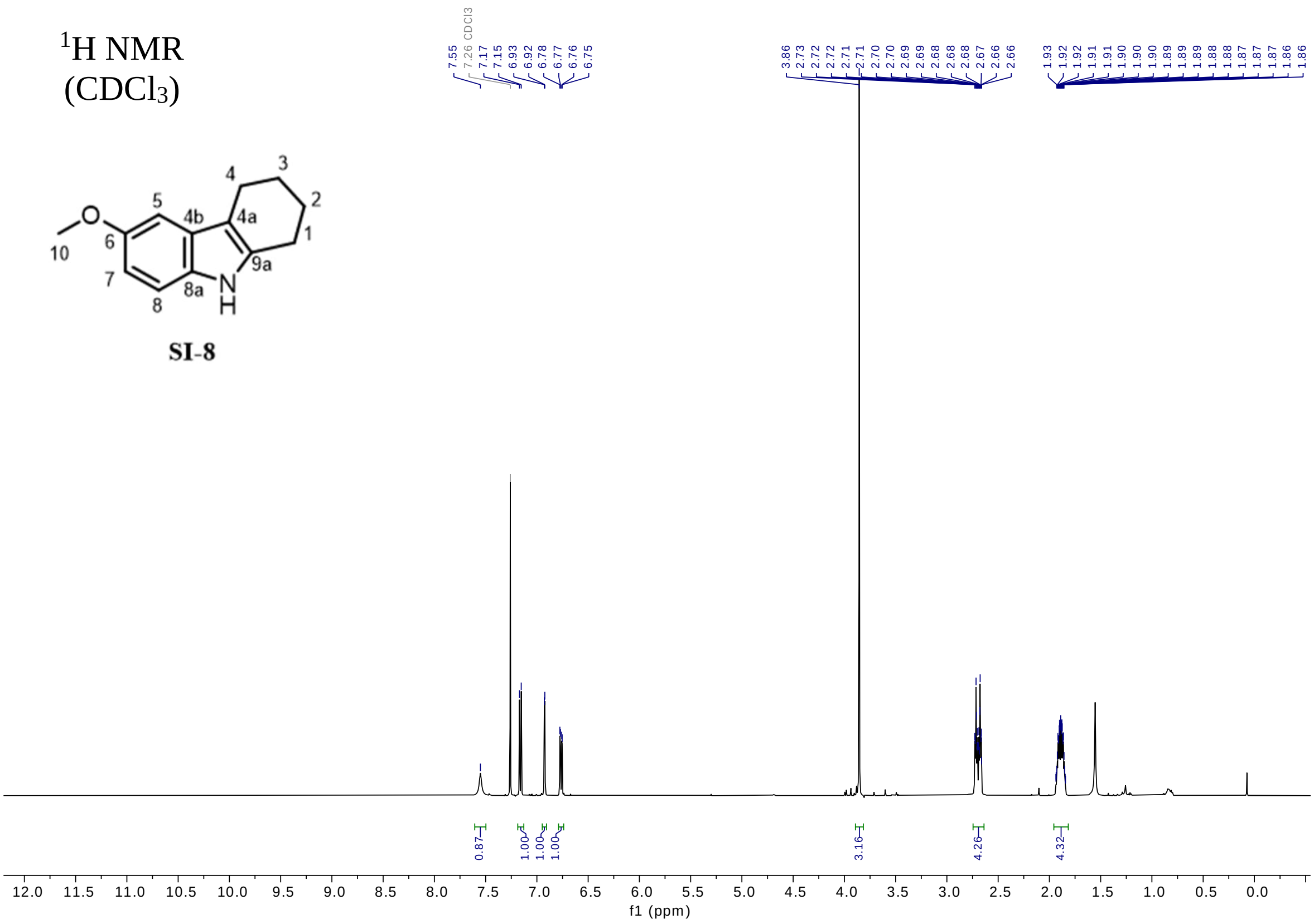

^{1}H NMR
($CDCl_3$)
SI-8
f1 (ppm)

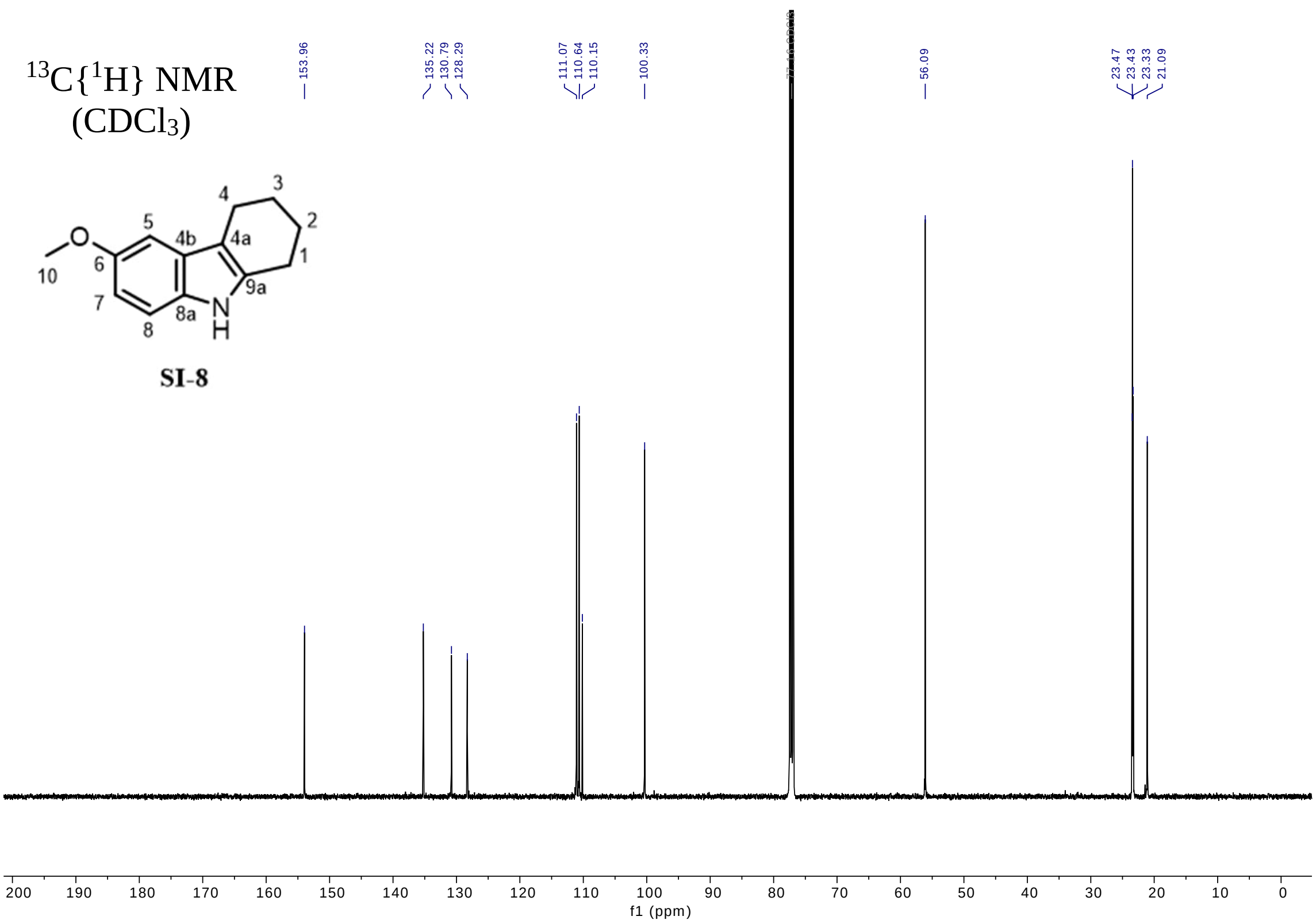

$^{13}C\{^1H\}$ NMR
($CDCl_3$)
SI-8
f1 (ppm)

^{1}H NMR
($CDCl_3$)

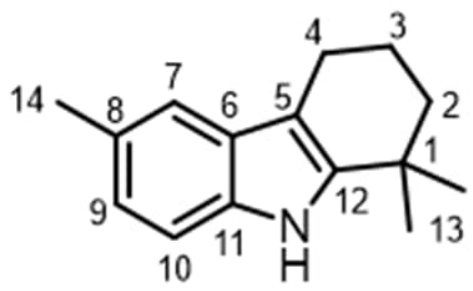

SI-9

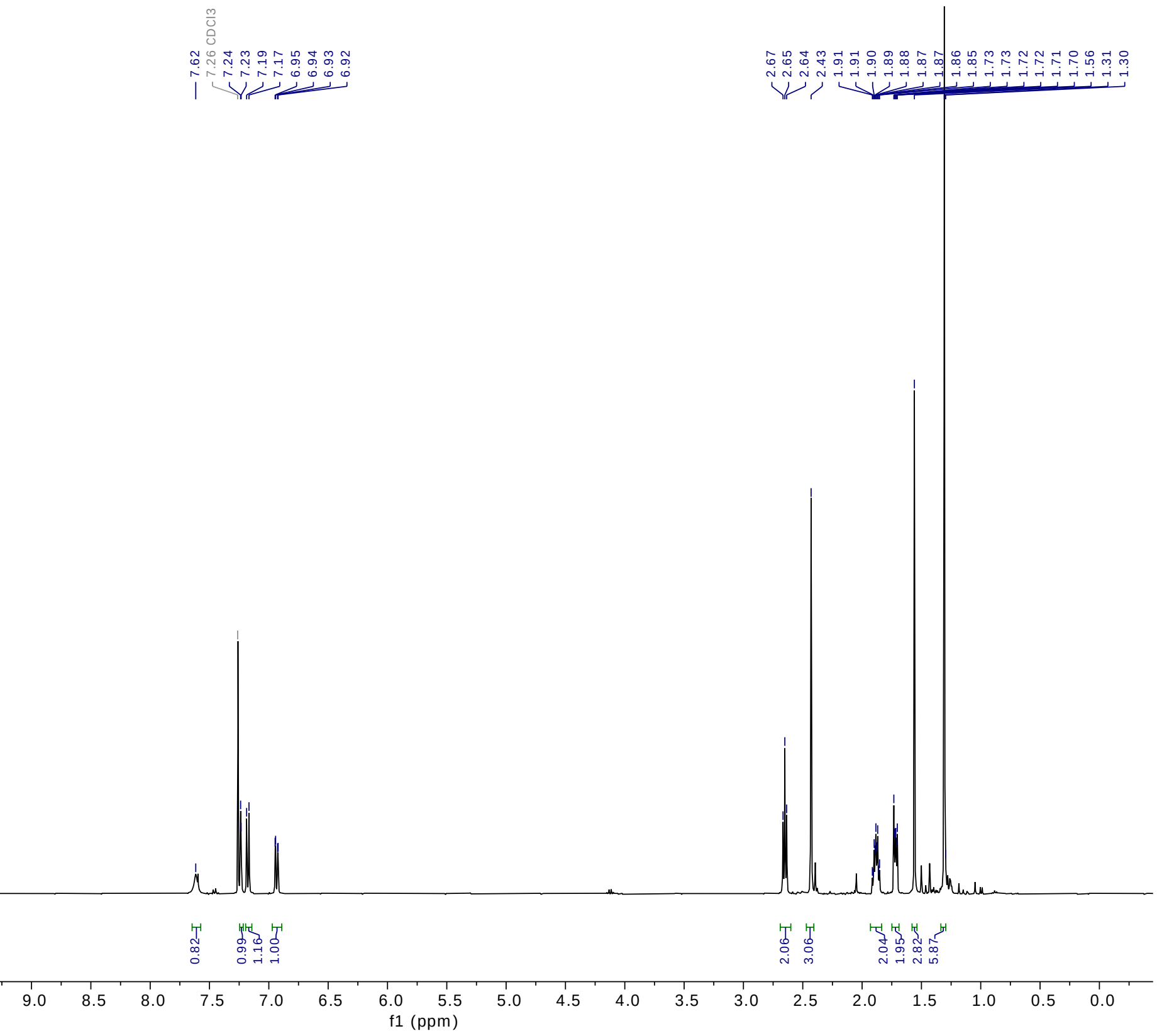

$^{13}C\{^{1}H\}$ NMR
($CDCl_3$)

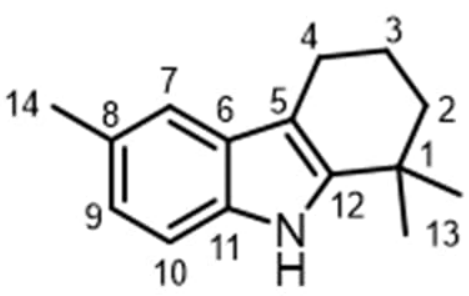

SI-9

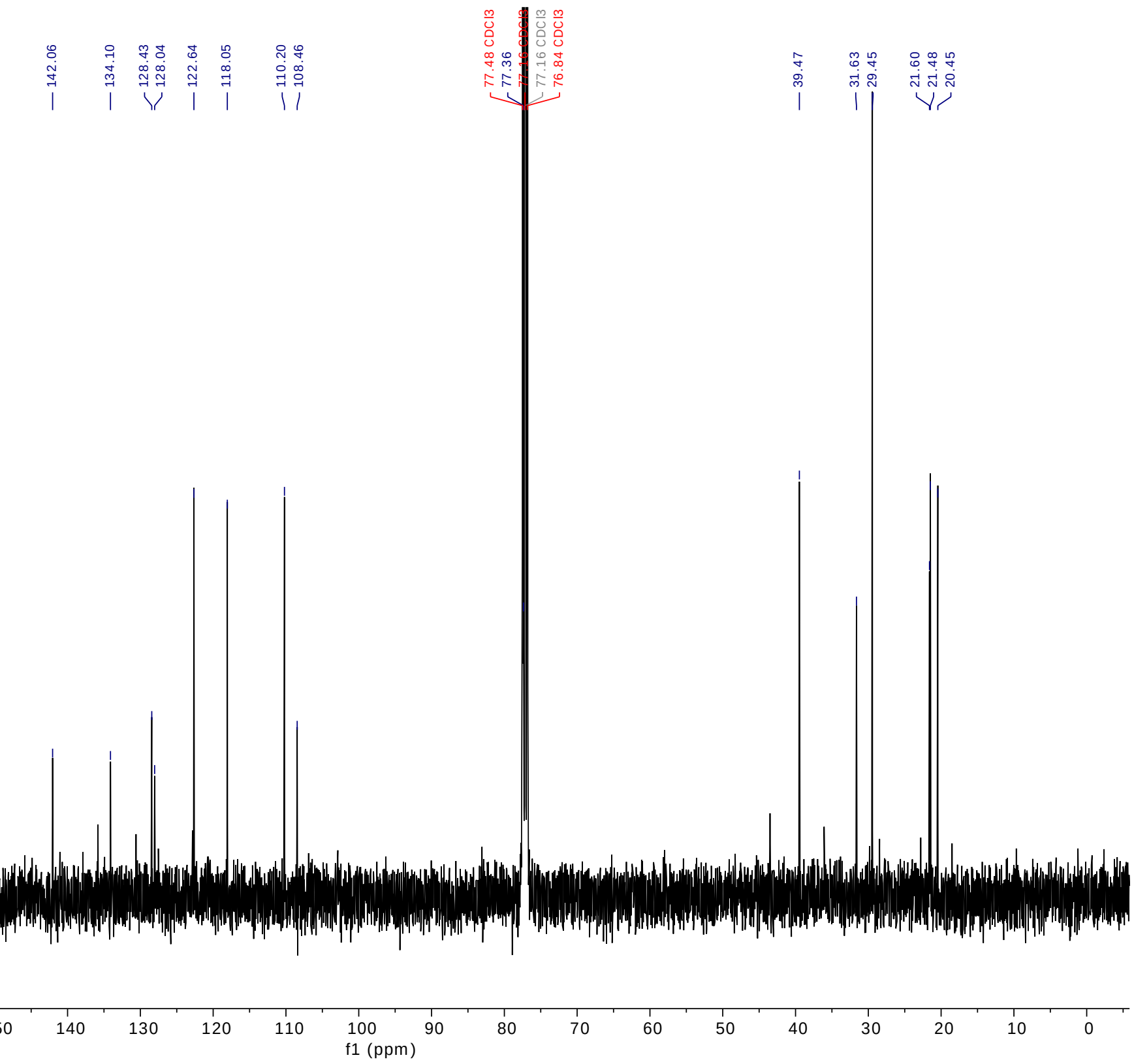

^{1}H NMR ($CDCl_3$)

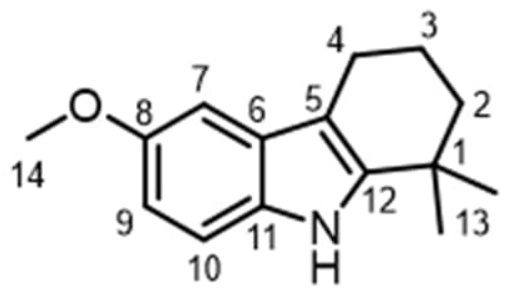

SI-10

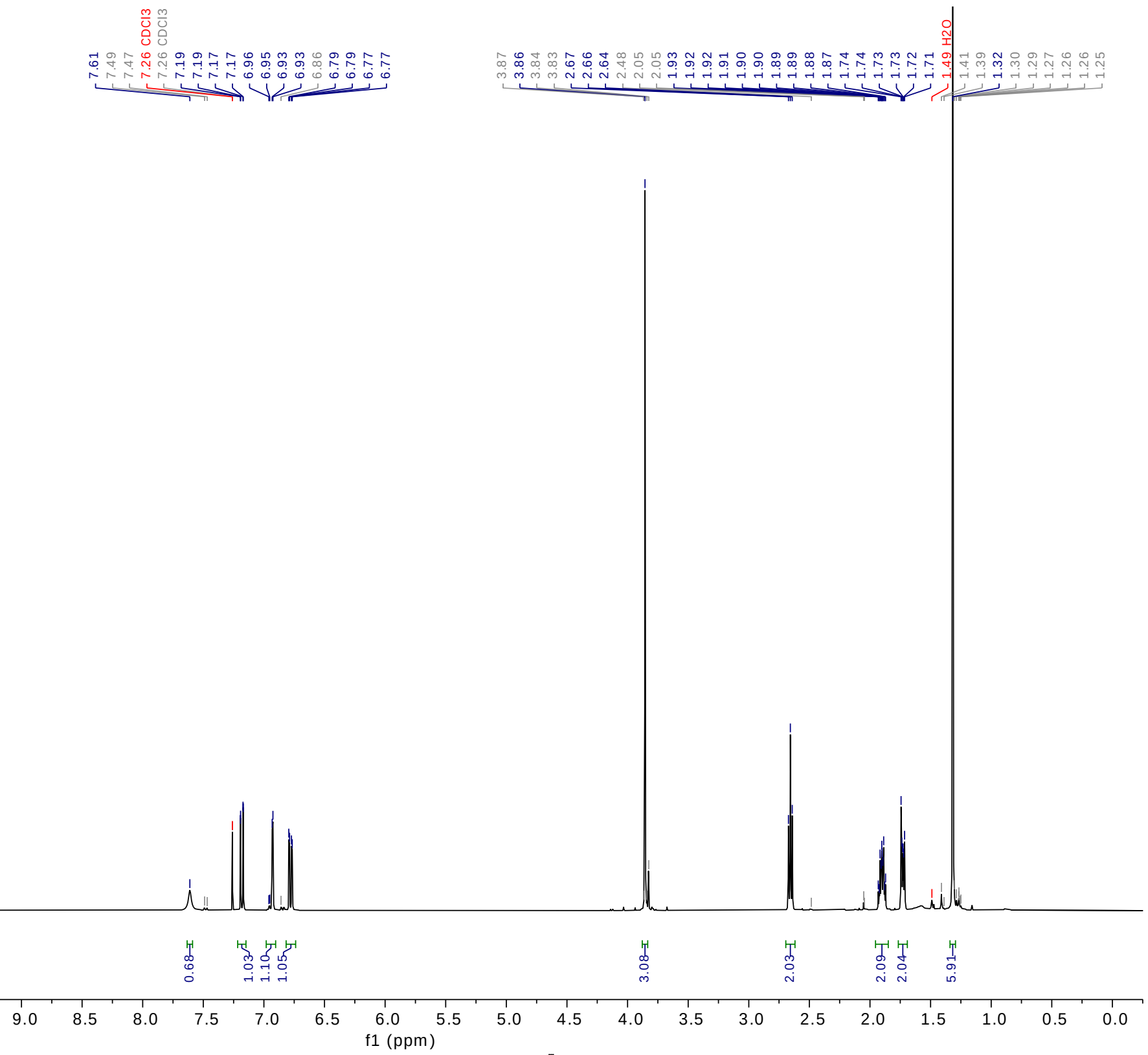

$^{13}C\{^{1}H\}$ NMR ($CDCl_3$)

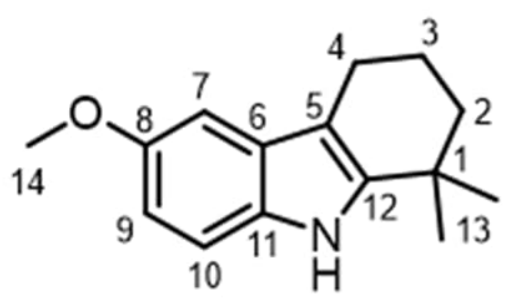

SI-10

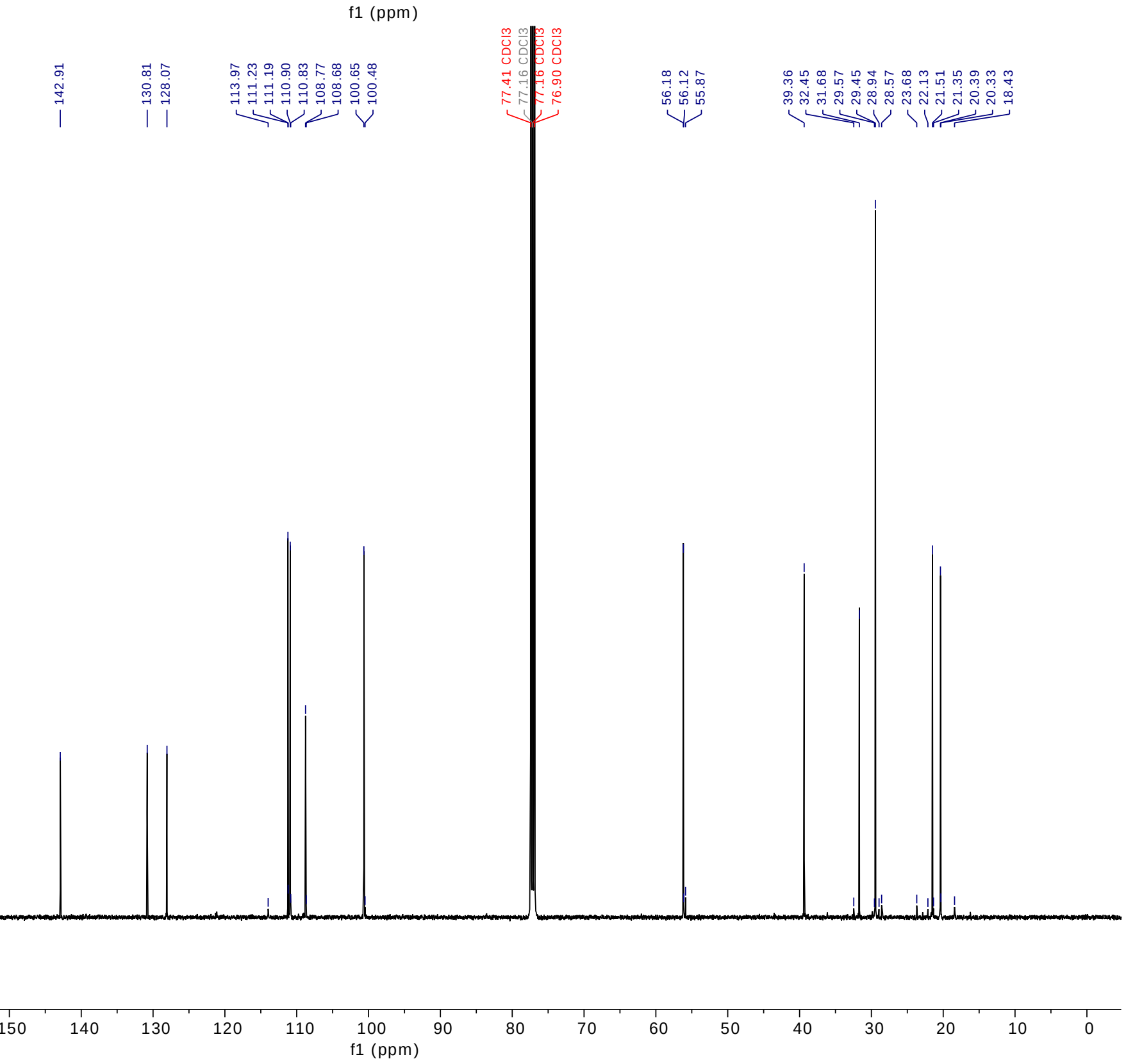

^{1}H NMR
($CDCl_3$)

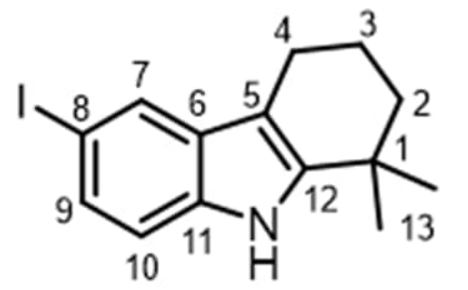

SI-11

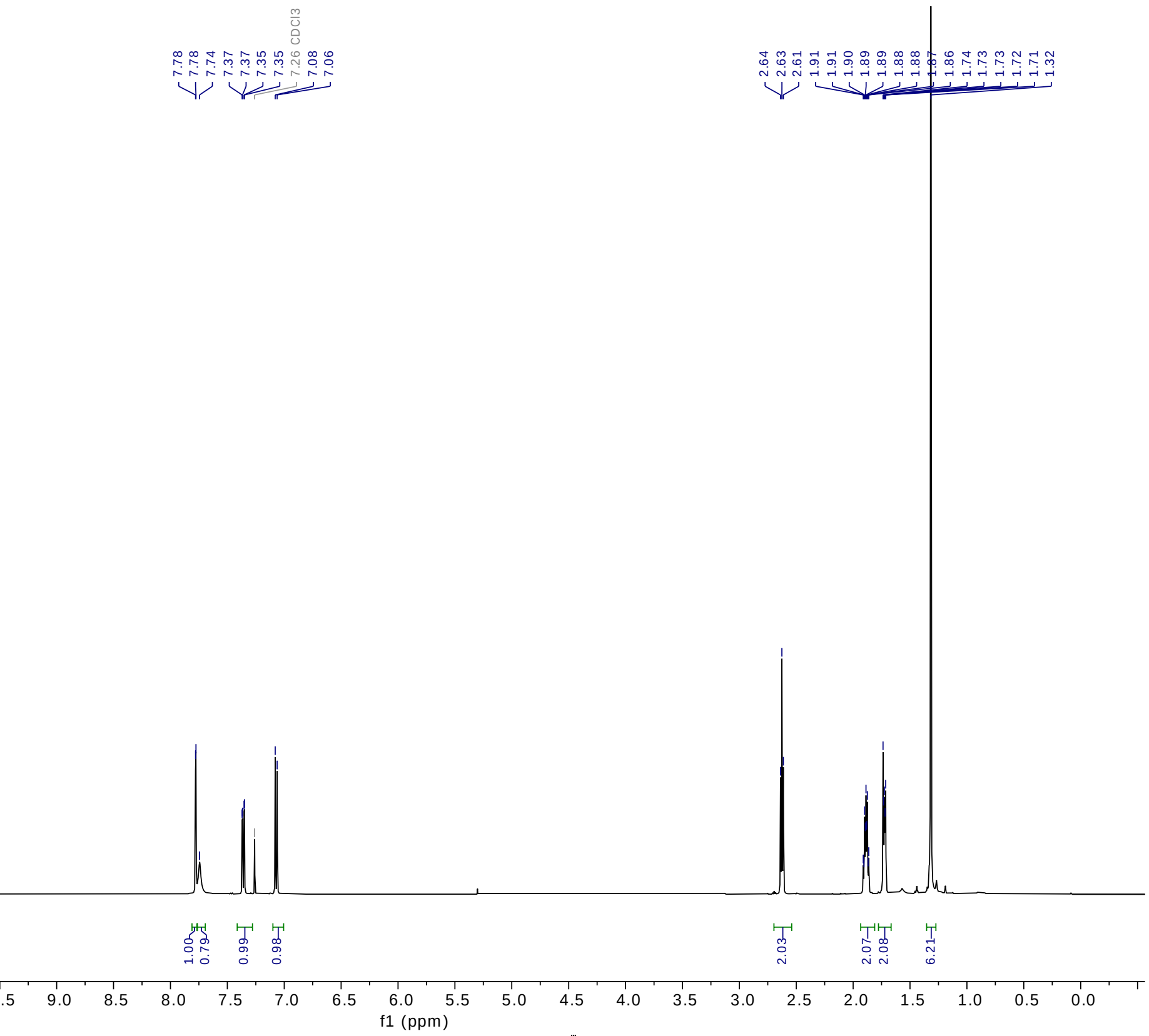

$^{13}C\{^1H\}$ NMR
($CDCl_3$)

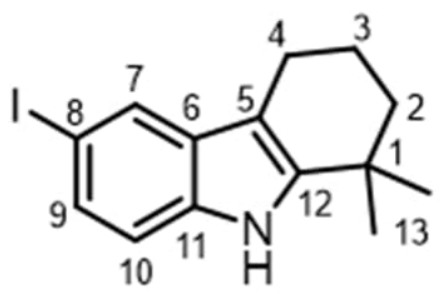

SI-11

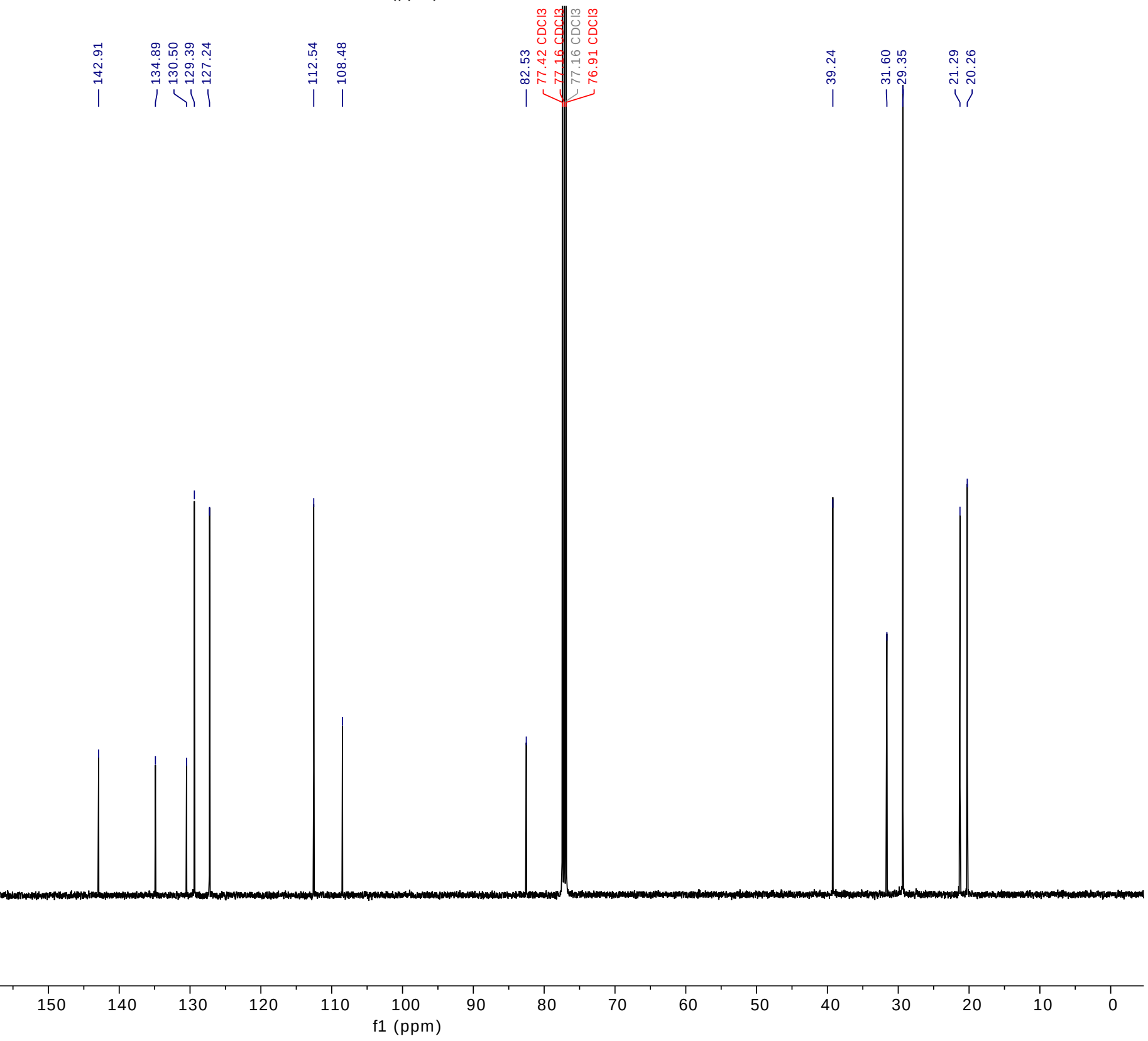

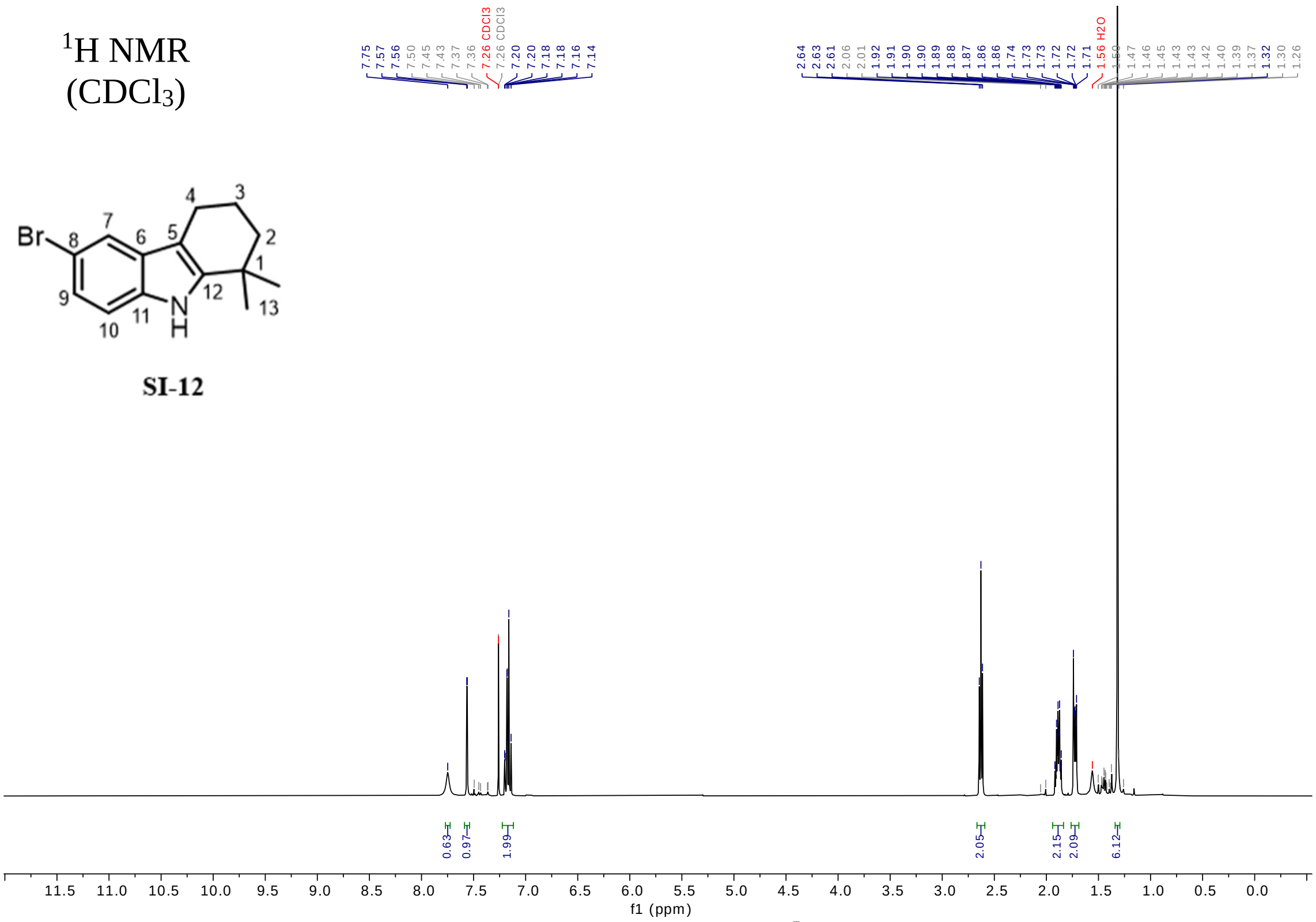

1H NMR
(CDCl3)
SI-12
7.75 7.57 7.56 7.50 7.45 7.43 7.37 7.36 7.26 CDCl3 7.26 CDCl3 7.20 7.20 7.18 7.18 7.16 7.14
2.64 2.63 2.61 2.06 2.01 1.92 1.91 1.90 1.90 1.89 1.88 1.87 1.86 1.86 1.74 1.73 1.73 1.72 1.72 1.71 1.56 H2O
0.63 0.97 1.99 2.05 2.15 2.09 6.12
f1 (ppm)

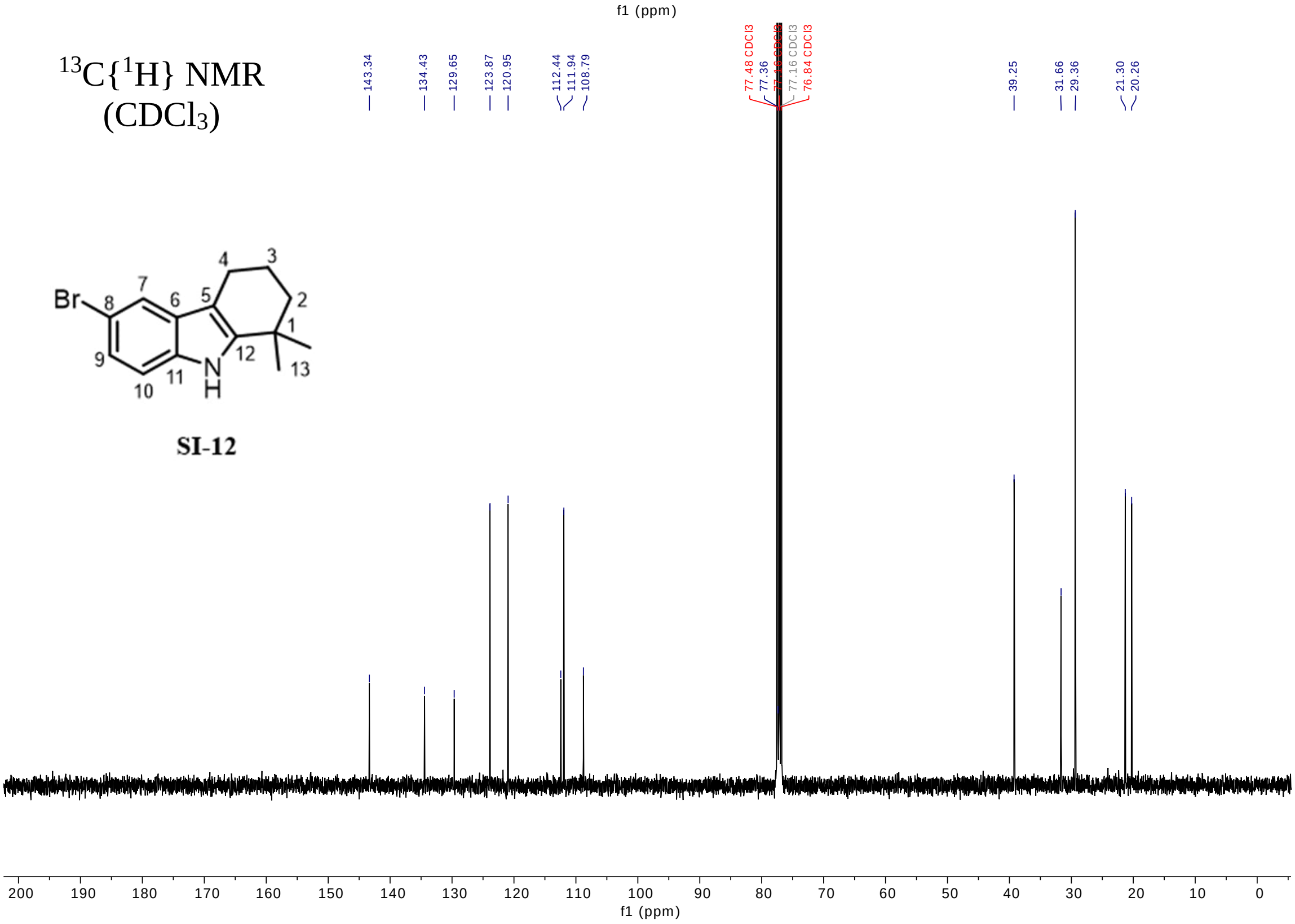

13C{1H} NMR
(CDCl3)
SI-12
143.34 134.43 129.65 123.87 120.95 112.44 111.94 108.79
77.48 CDCl3 77.36 77.16 CDCl3 76.84 CDCl3
39.25 31.66 29.36 21.30 20.26
f1 (ppm)

^{1}H NMR
($CDCl_3$)

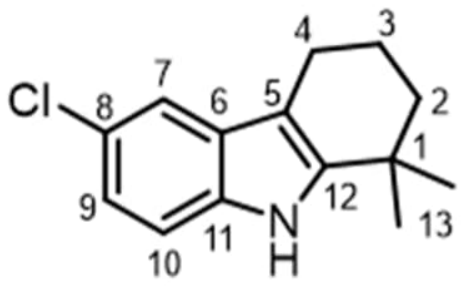

SI-13

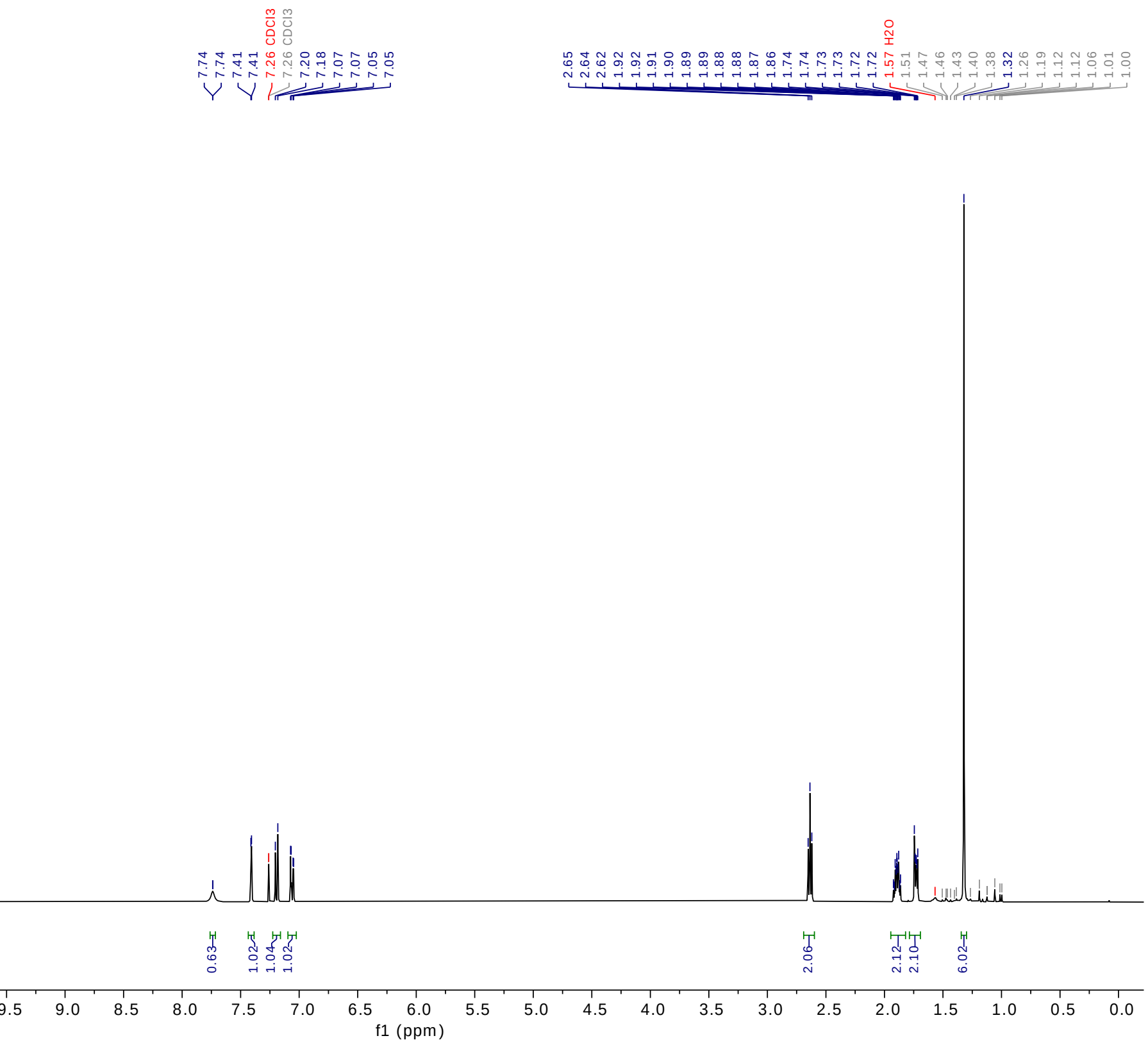

^{13}C{^{1}H} NMR
($CDCl_3$)

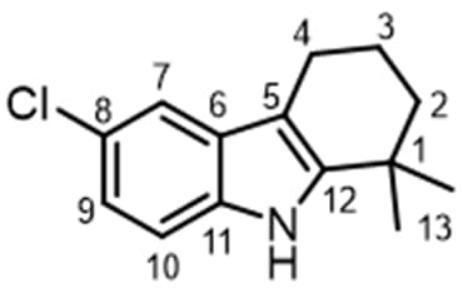

SI-13

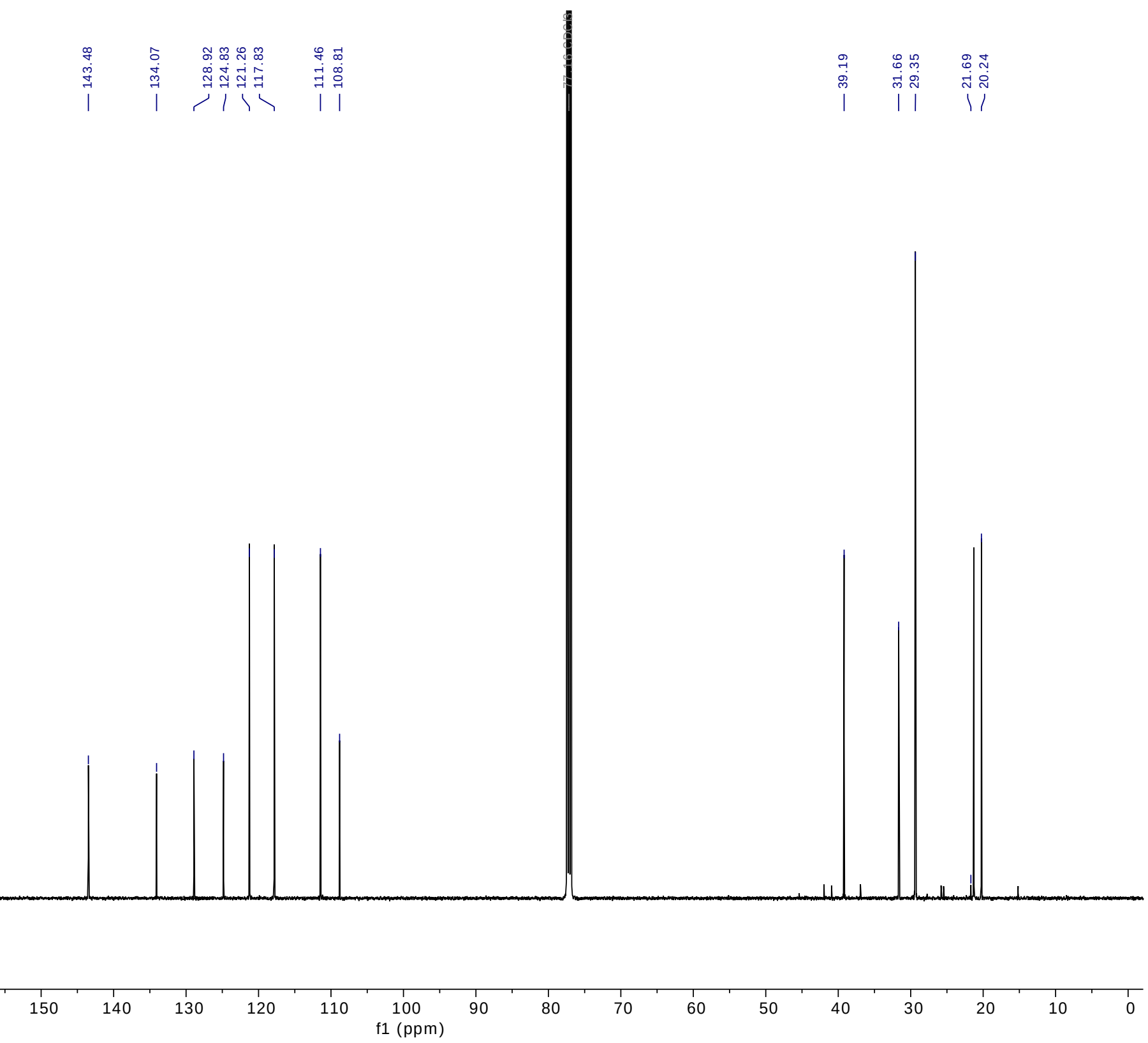

^{1}H NMR
($CDCl_3$)

SI-14

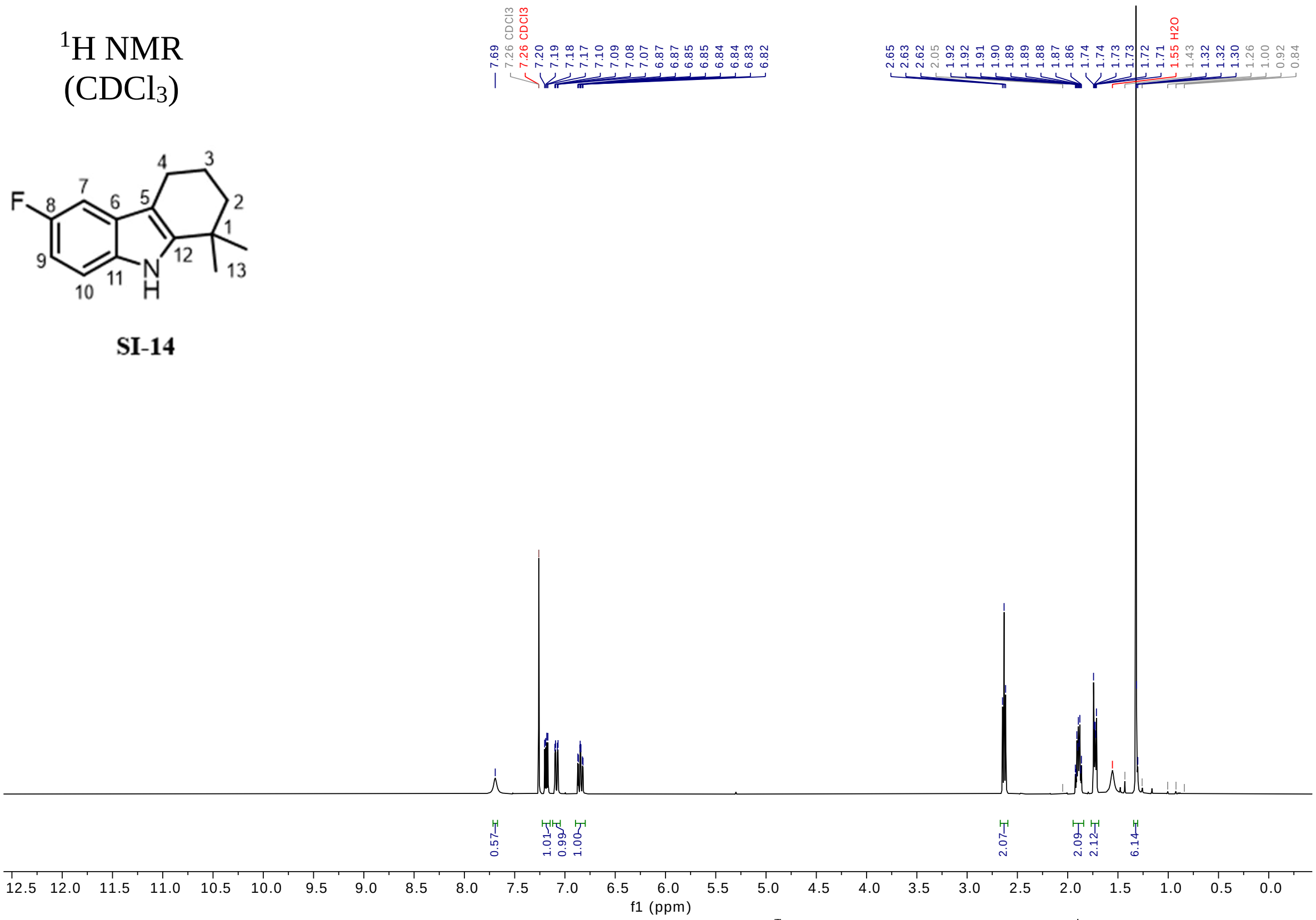

$^{13}C\{^{1}H\}$ NMR
($CDCl_3$)

SI-14

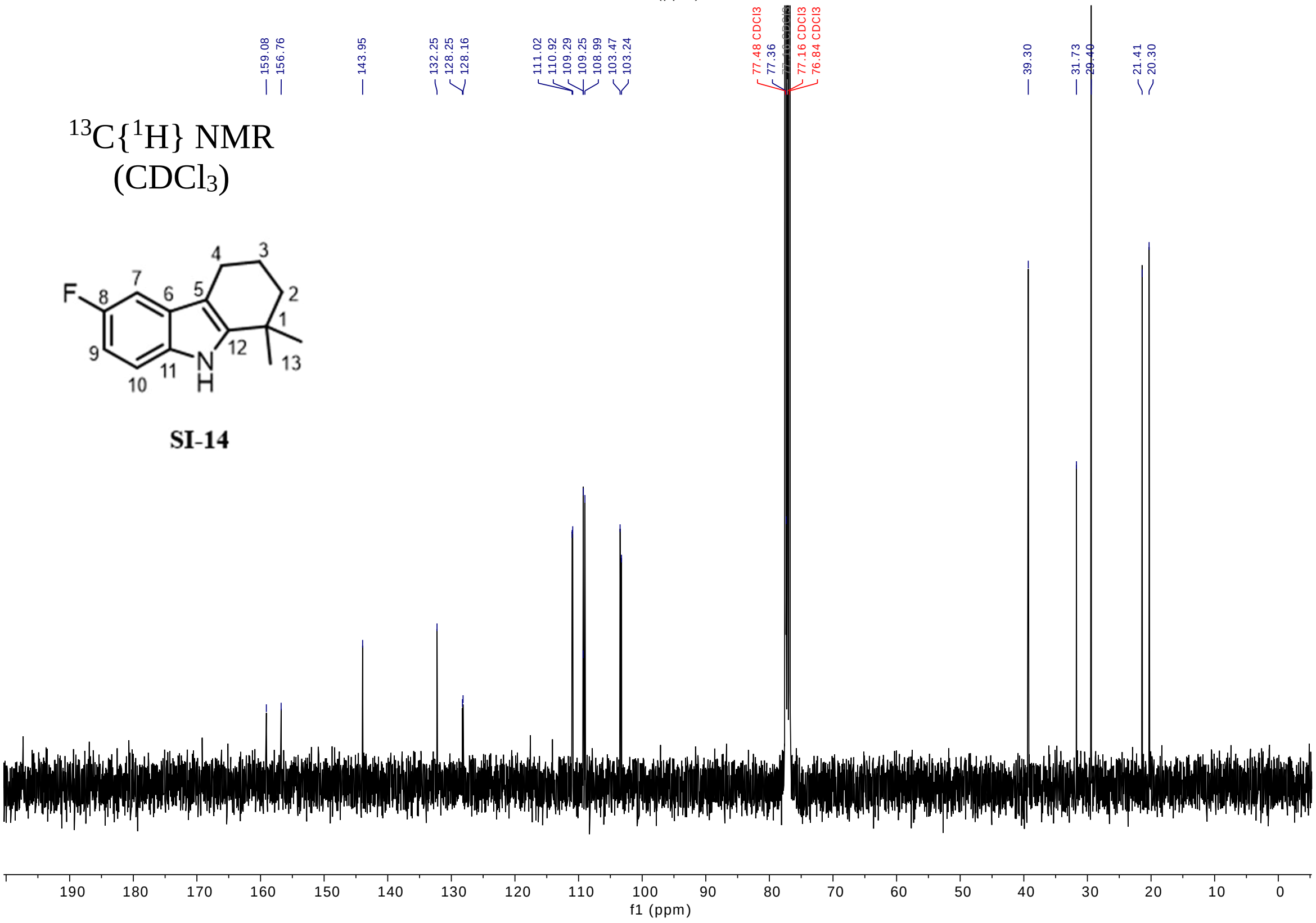

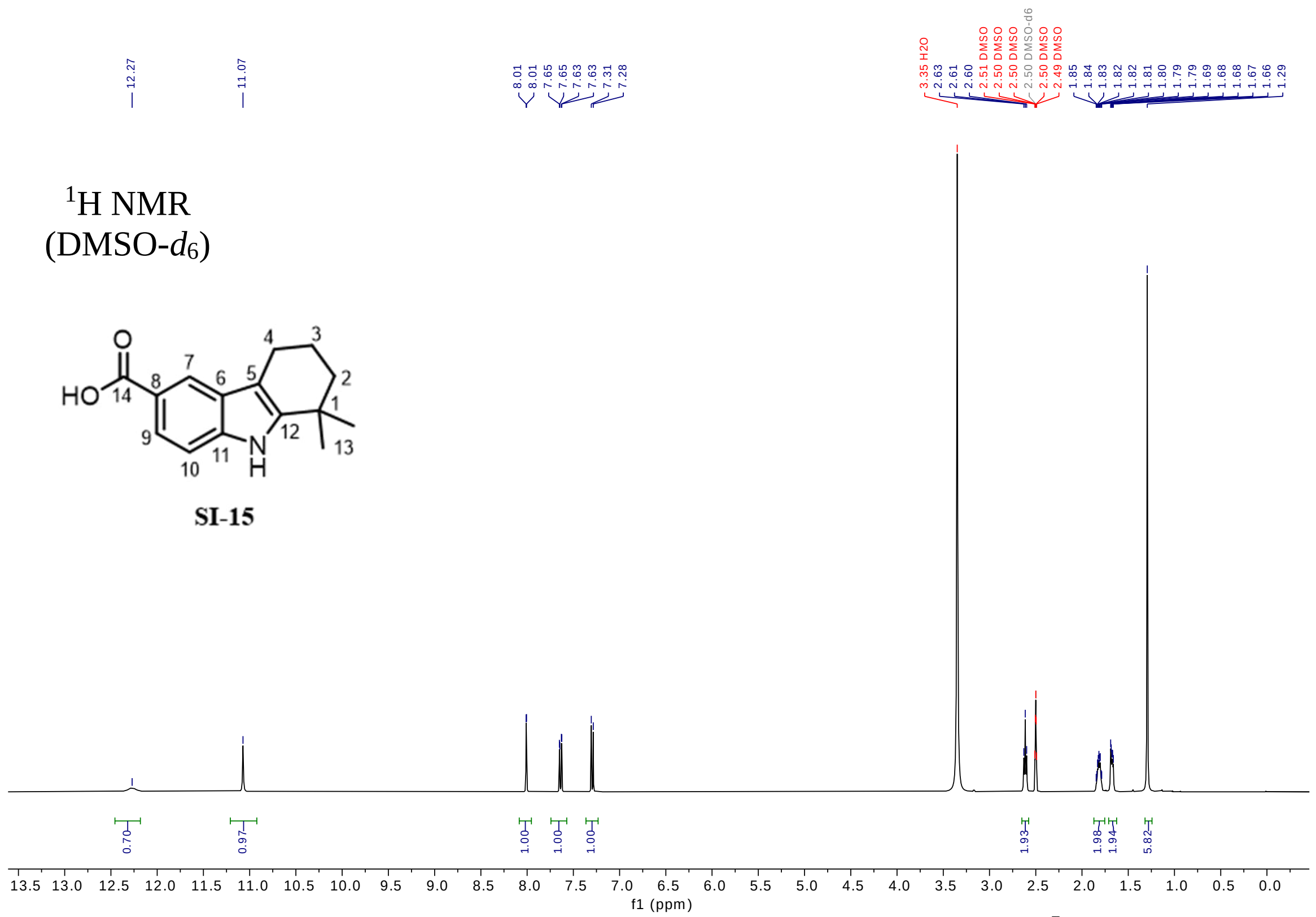

^{1}H NMR
(DMSO-d_6)
SI-15
f1 (ppm)

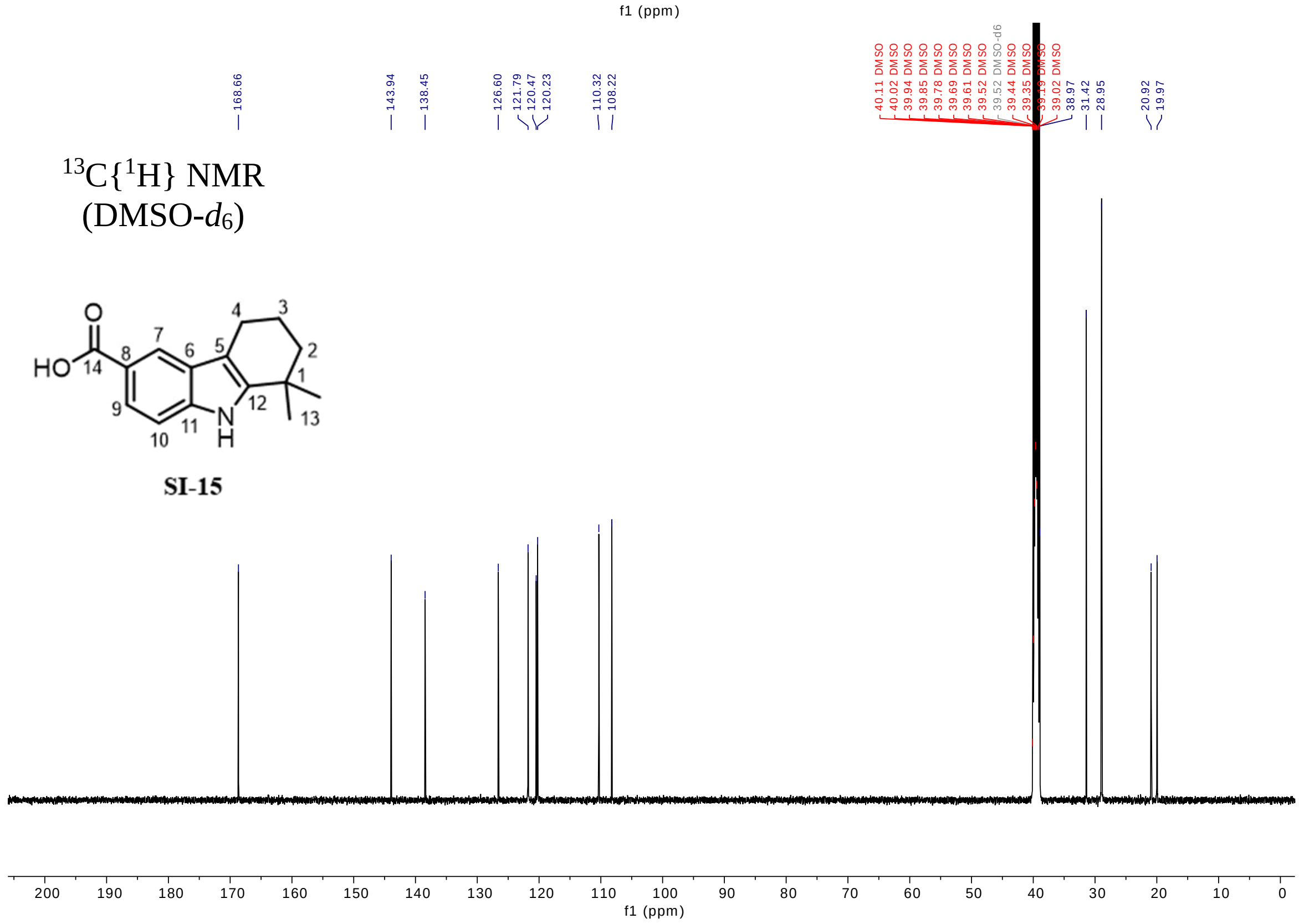

^{13}C{^{1}H} NMR
(DMSO-d_6)
SI-15
f1 (ppm)

^{1}H NMR
($CDCl_3$)

SI-16

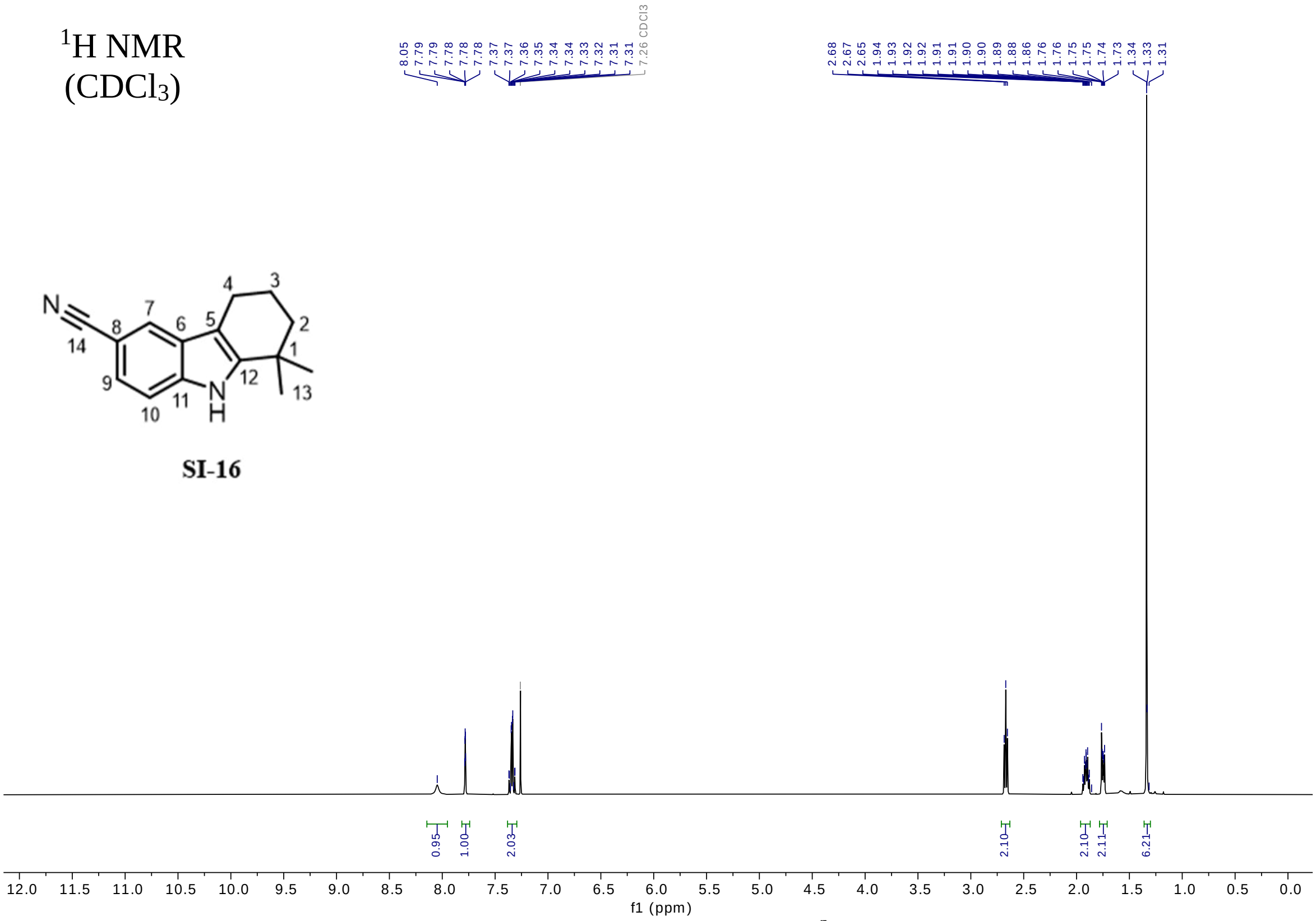

^{13}C{^{1}H} NMR
($CDCl_3$)

SI-16

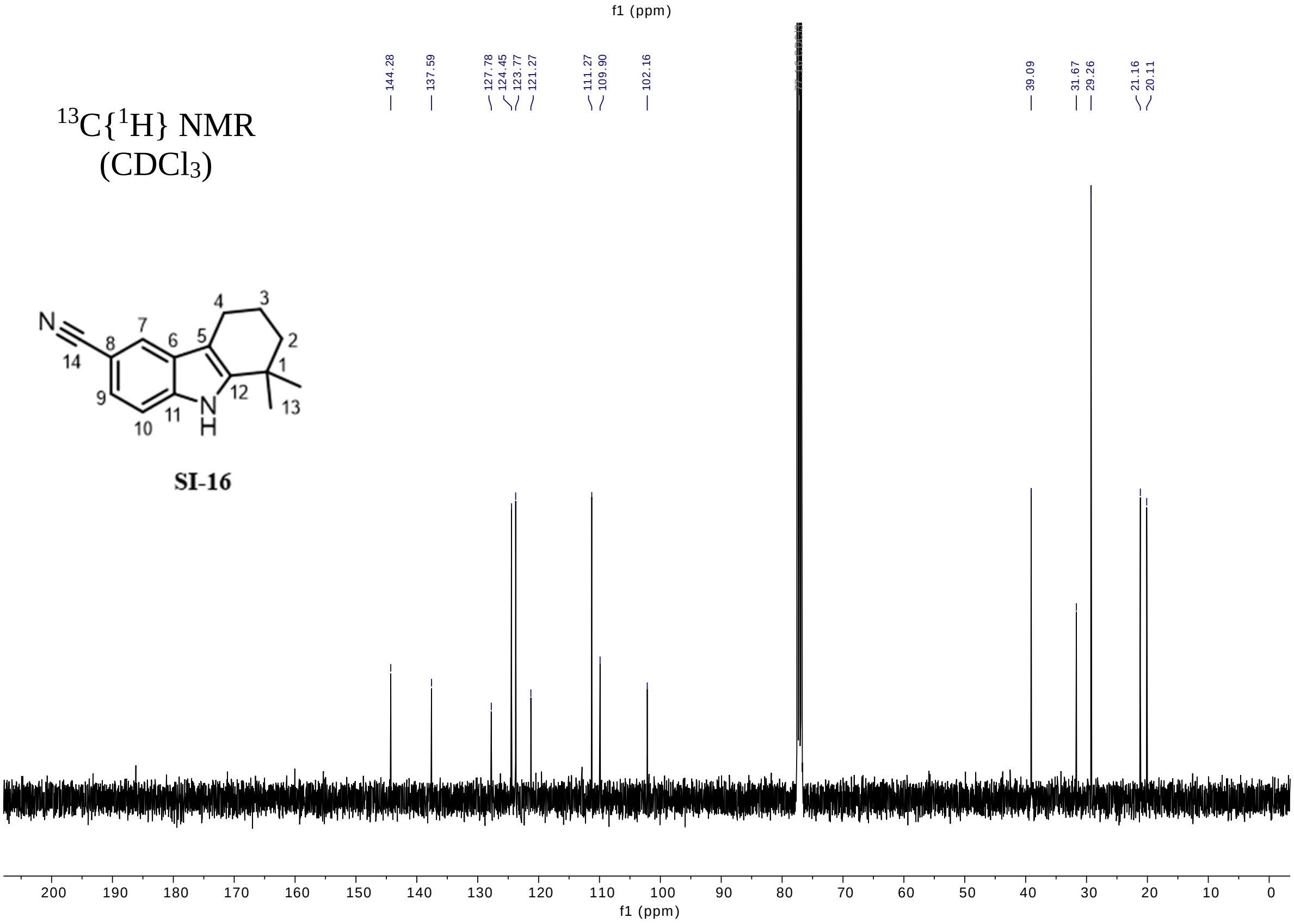

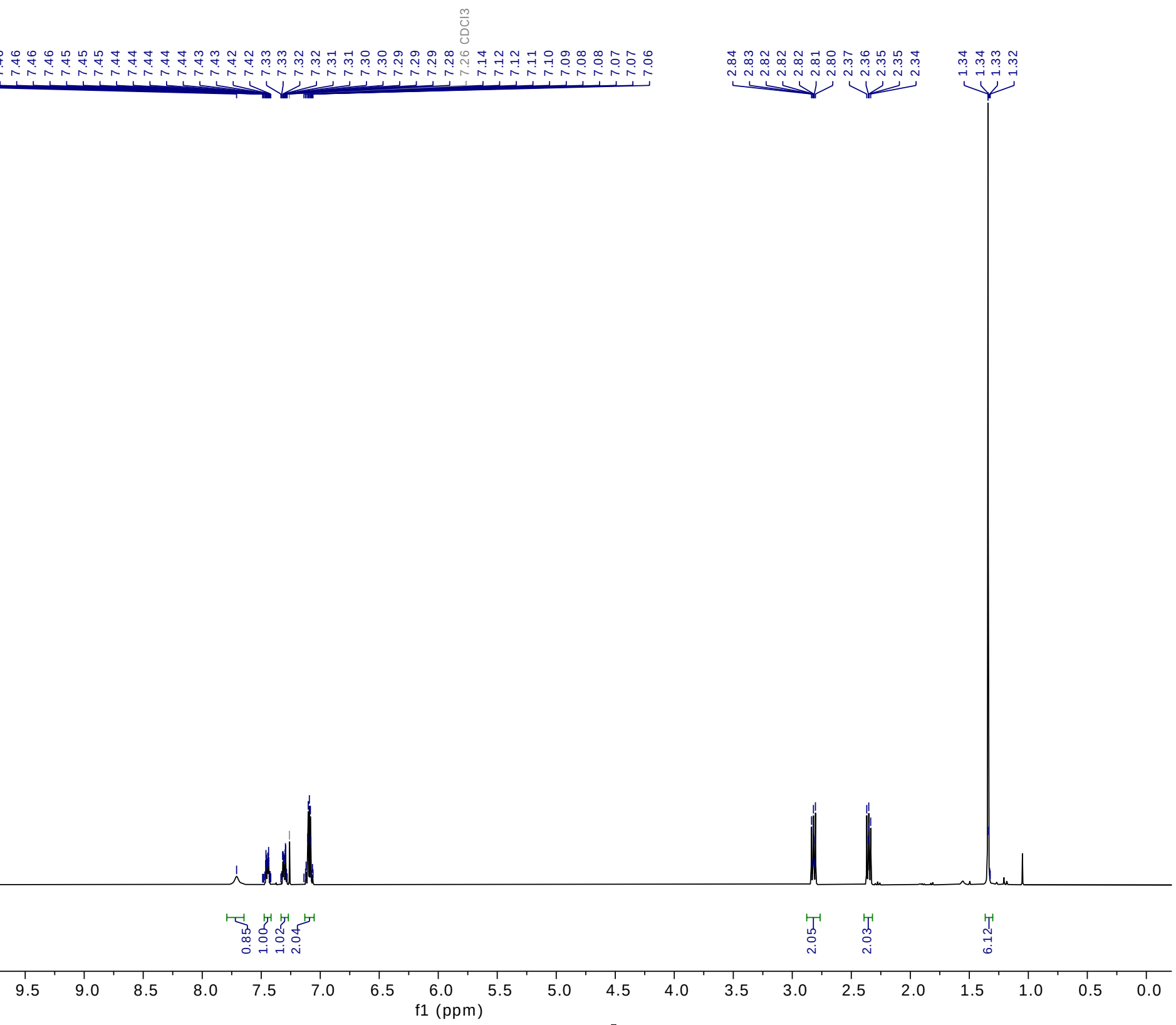

^{1}H NMR
($CDCl_3$)

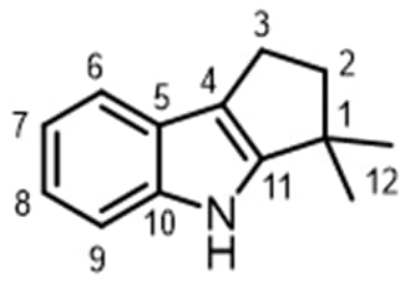

SI-17

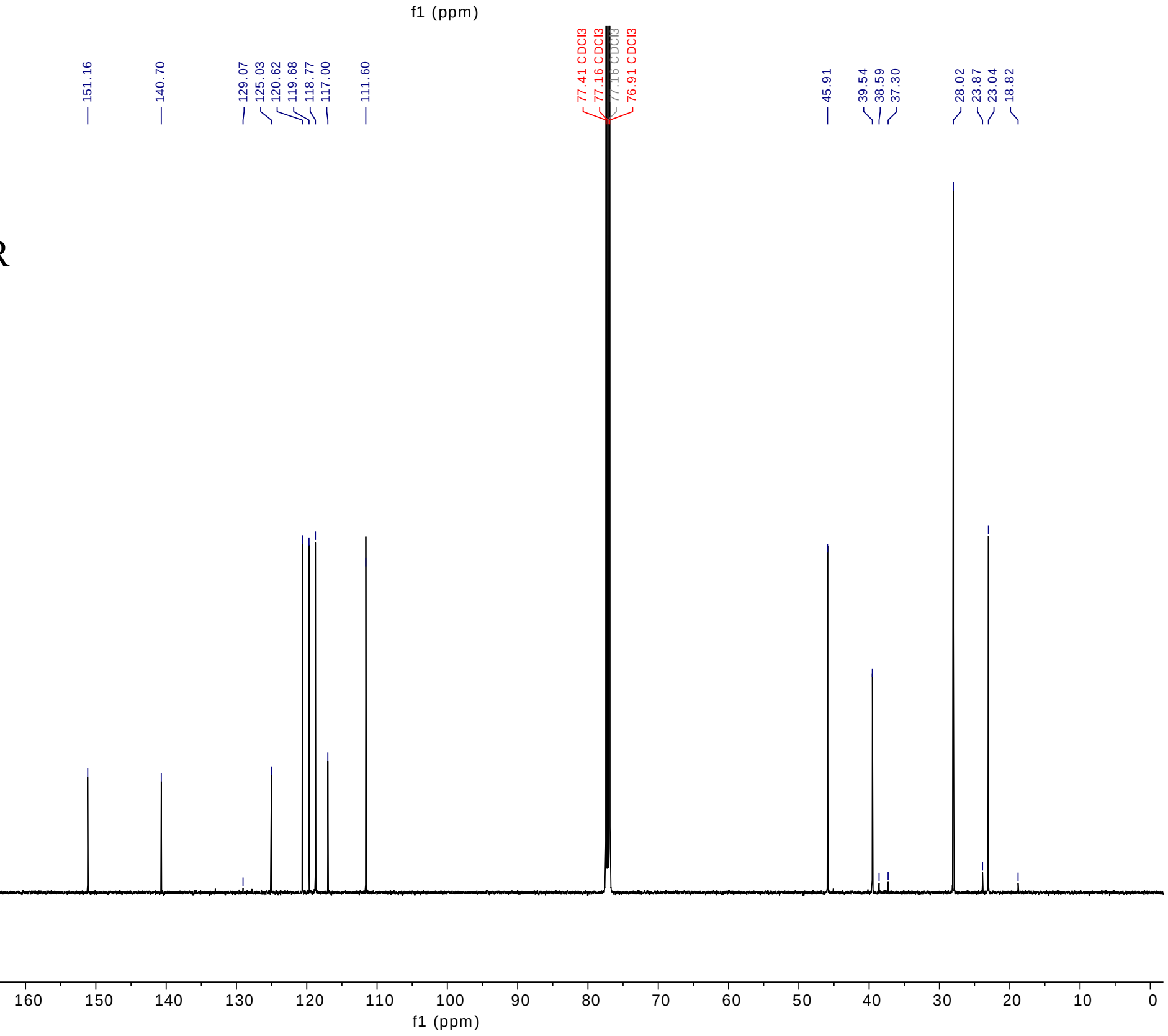

$^{13}C\{^1H\}$ NMR
($CDCl_3$)

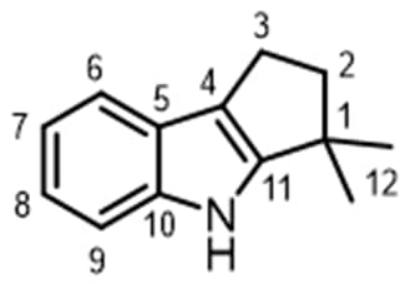

SI-17

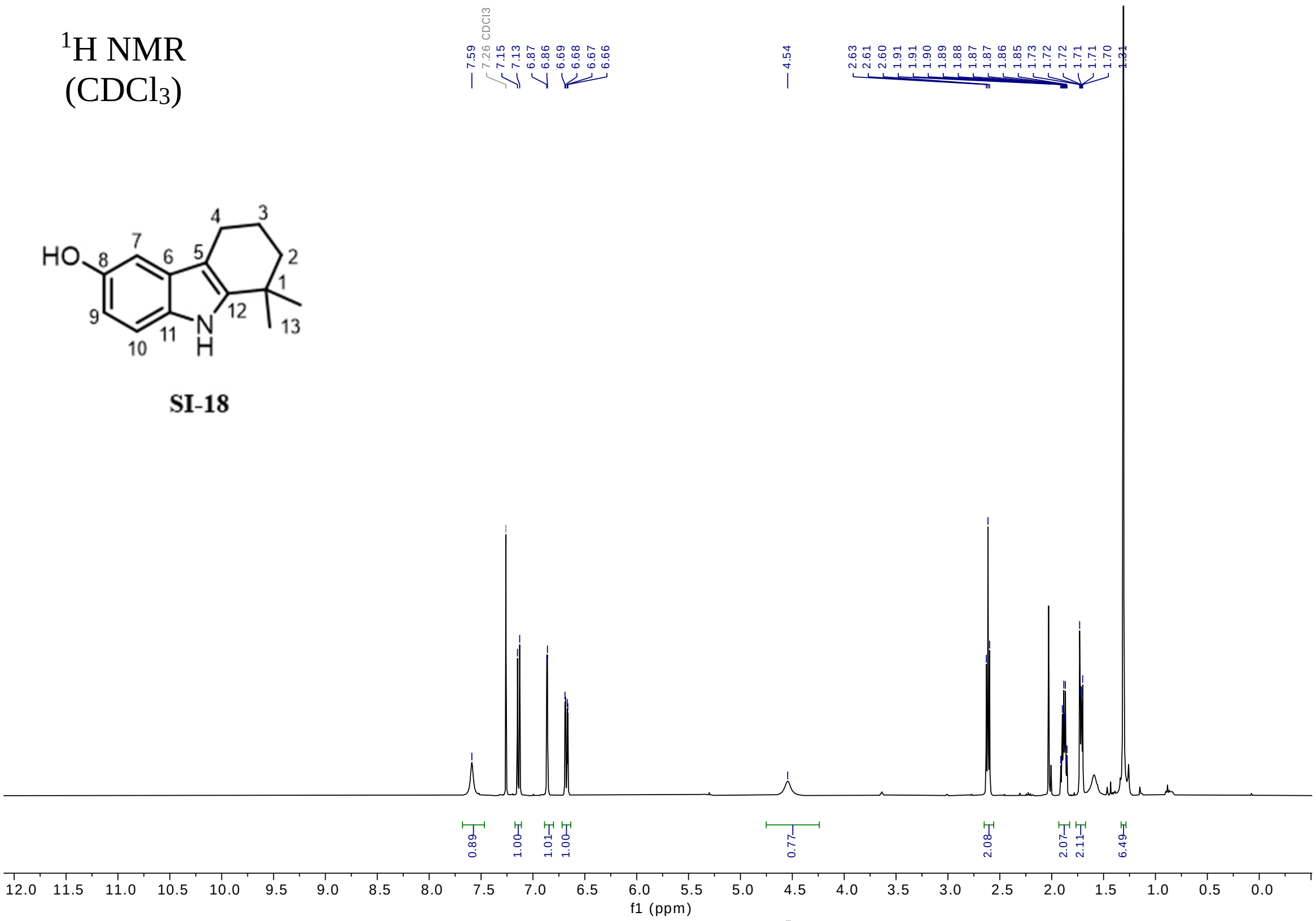
1H NMR
(CDCl3)
SI-18
7.59
7.26 CDCl3
7.15
7.13
6.87
6.86
6.69
6.68
6.67
6.66
4.54
2.63
2.61
2.60
1.91
1.91
1.90
1.89
1.88
1.87
1.87
1.86
1.85
1.73
1.72
1.72
1.71
1.71
1.70
1.31
0.89
1.00
1.01
1.00
0.77
2.08
2.07
2.11
6.49
f1 (ppm)

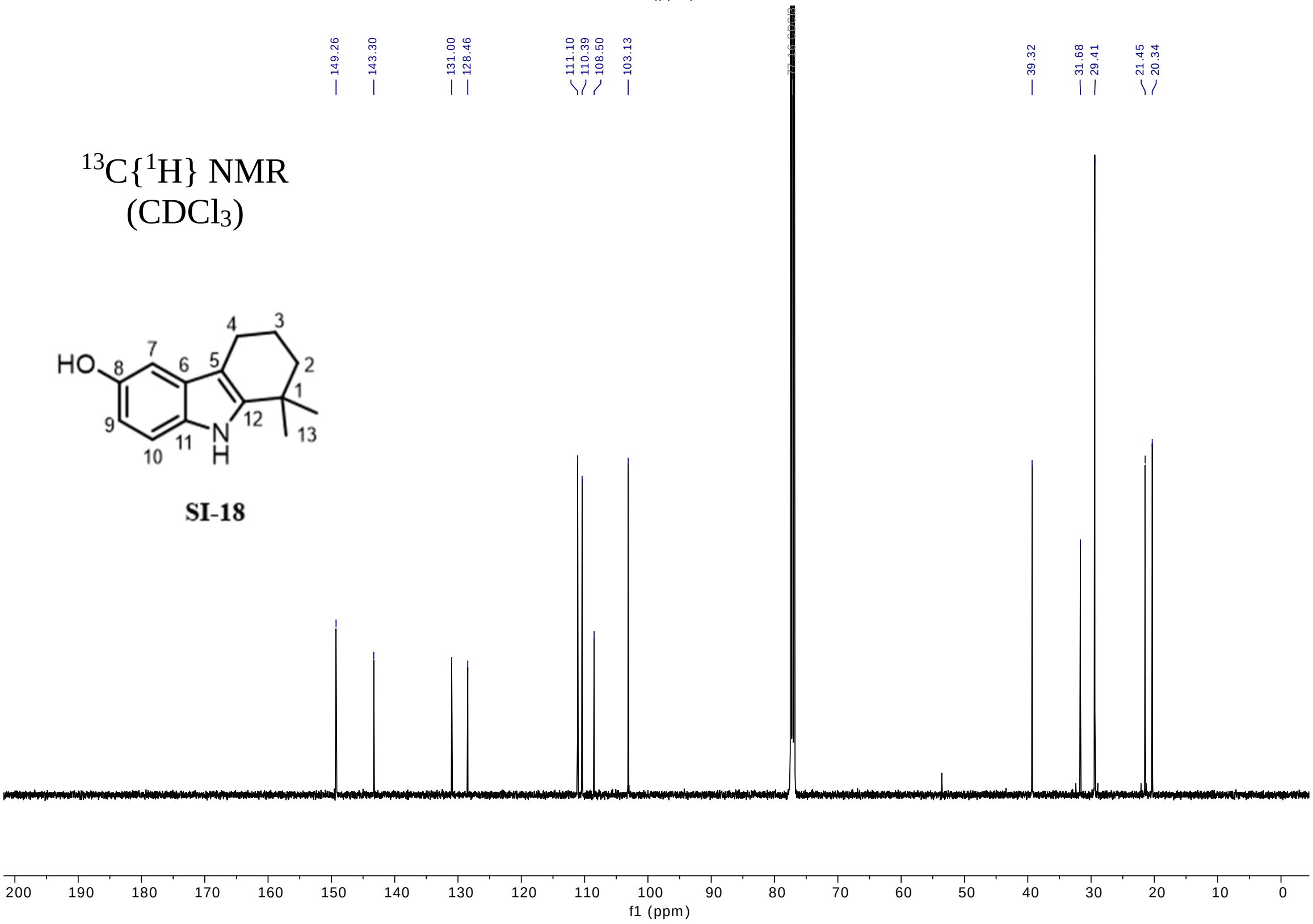
13C{1H} NMR
(CDCl3)
SI-18
149.26
143.30
131.00
128.46
111.10
110.39
108.50
103.13
39.32
31.68
29.41
21.45
20.34
f1 (ppm)

^{1}H NMR
($CDCl_3$)

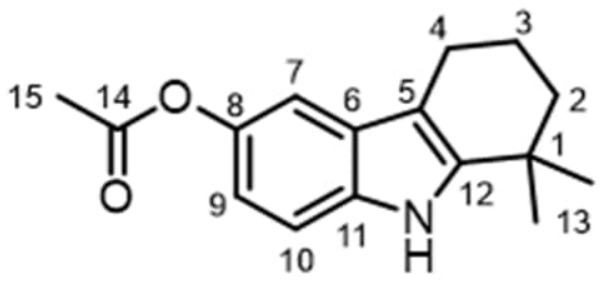

SI-19

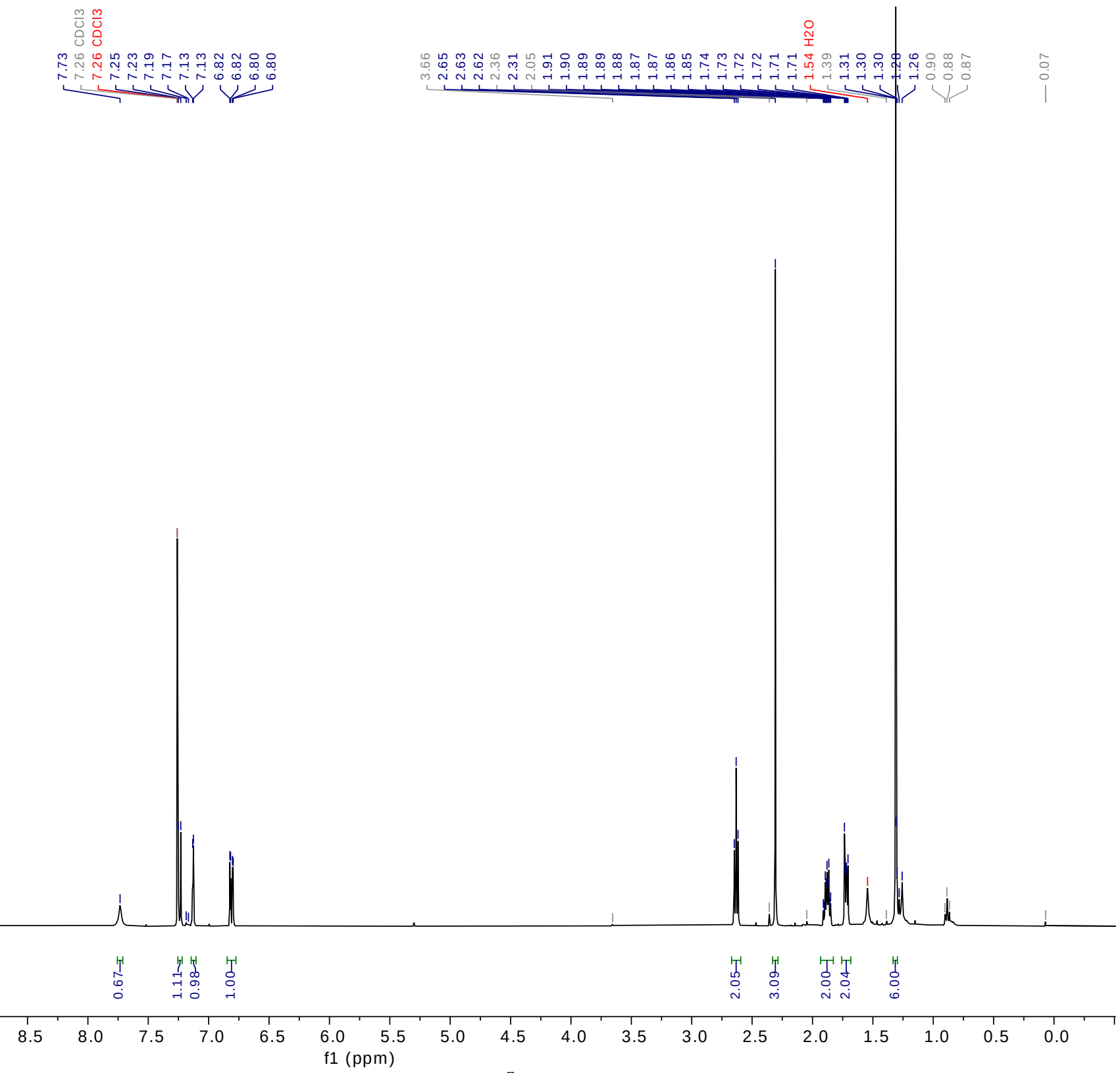

$^{13}C\{^{1}H\}$ NMR
($CDCl_3$)

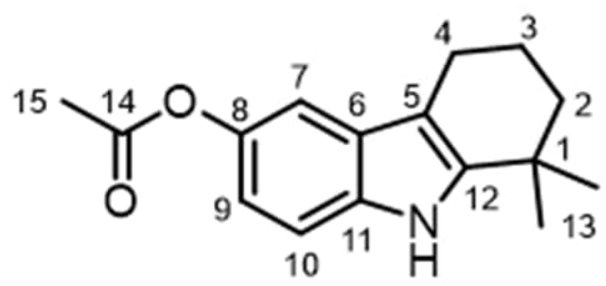

SI-19

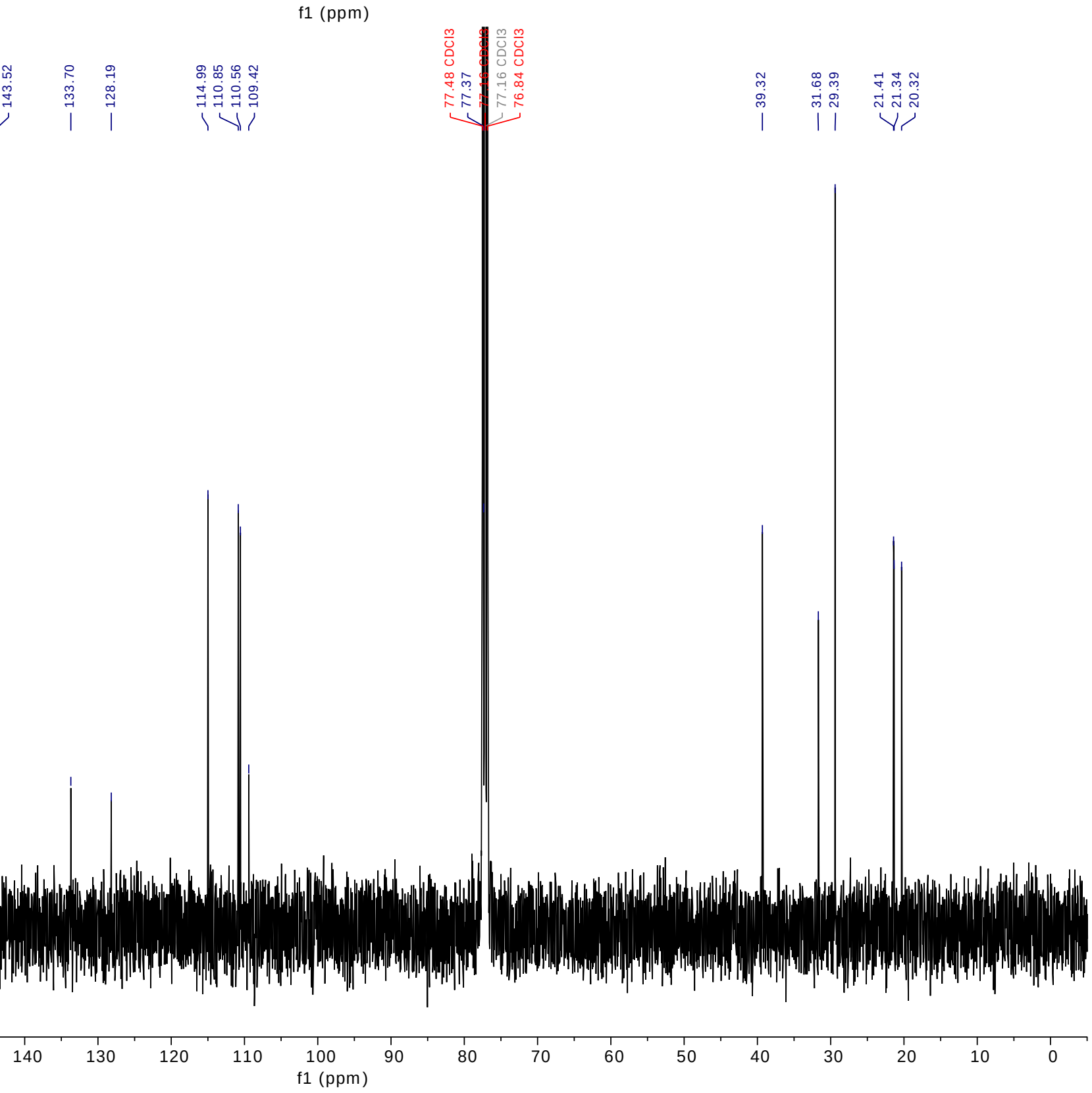

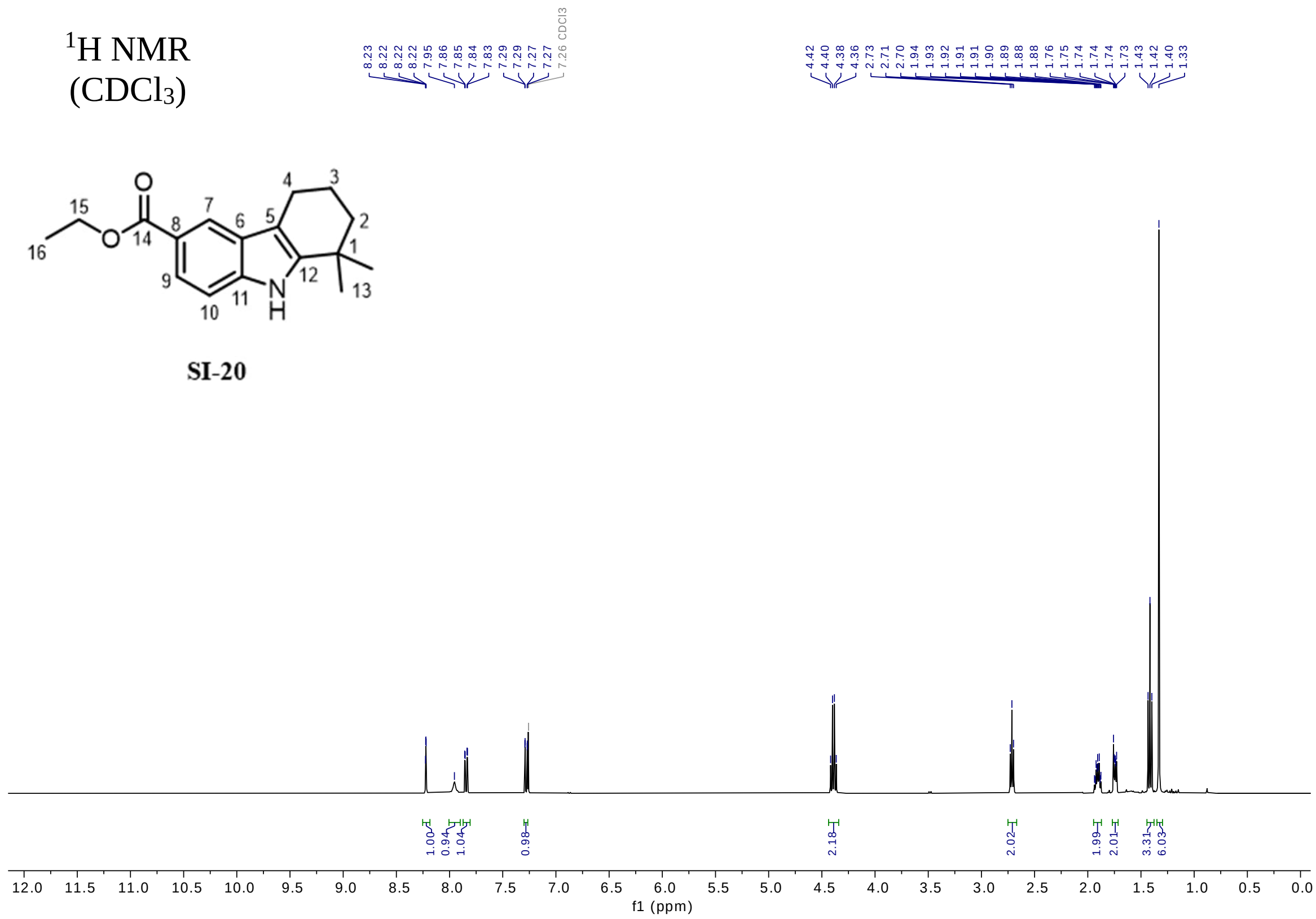

^{1}H NMR
($CDCl_3$)
SI-20
8.23 8.22 8.22 8.22 7.95 7.86 7.85 7.84 7.83 7.29 7.29 7.27 7.27
7.26 CDCl3
4.42 4.40 4.38 4.36 2.73 2.71 2.70 1.94 1.93 1.92 1.91 1.91 1.90 1.89 1.88 1.88 1.76 1.75 1.74 1.74 1.74 1.73 1.43 1.42 1.40 1.33
1.00 0.94 1.04 0.98 2.18 2.02 1.99 2.01 3.31 6.03
f1 (ppm)

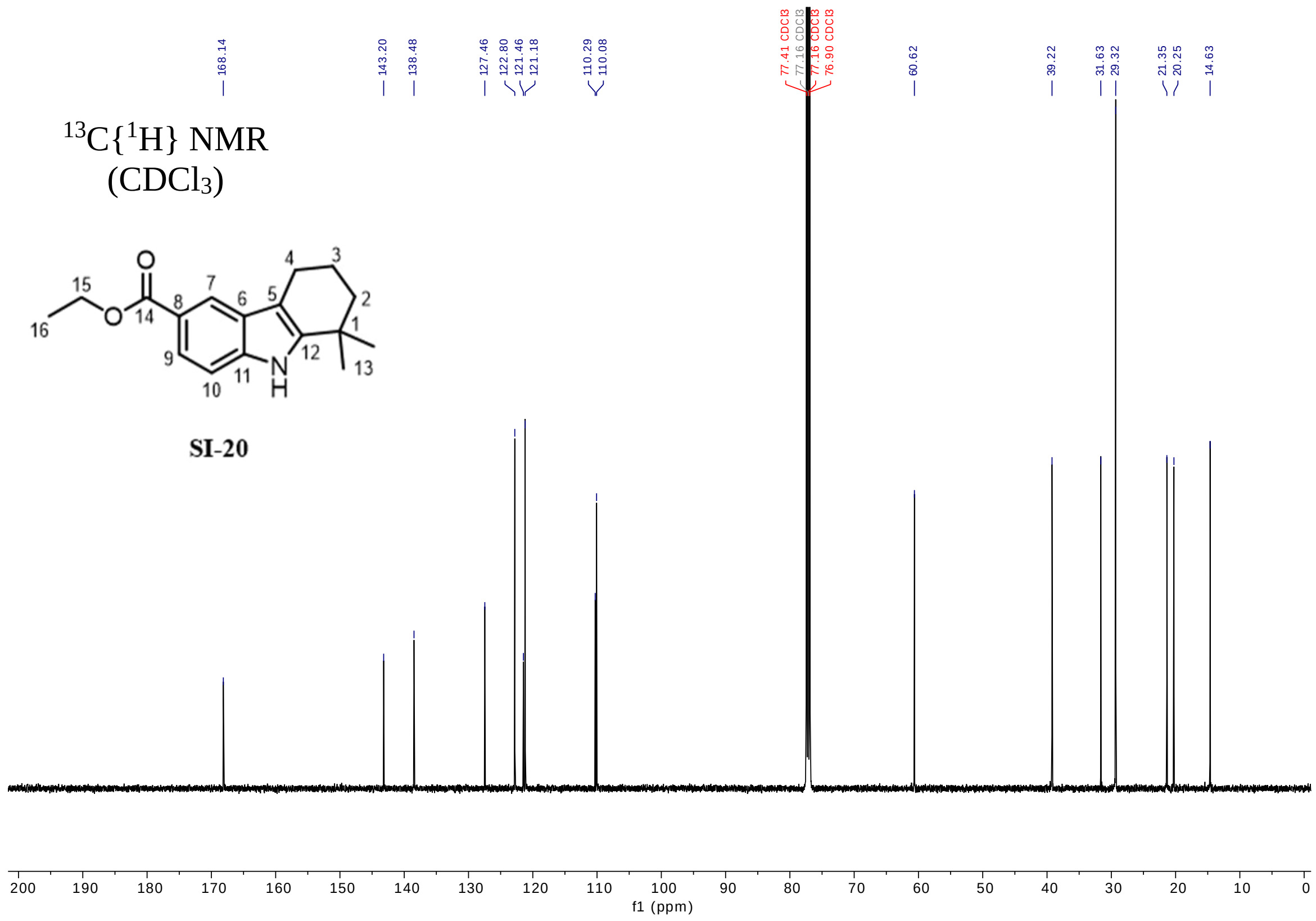

^{13}C{^{1}H} NMR
($CDCl_3$)
SI-20
168.14 143.20 138.48 127.46 122.80 121.46 121.18 110.29 110.08
77.41 CDCl3 77.16 CDCl3 77.16 CDCl3 76.90 CDCl3
60.62 39.22 31.63 29.32 21.35 20.25 14.63
f1 (ppm)

^{1}H NMR
($CDCl_3$)

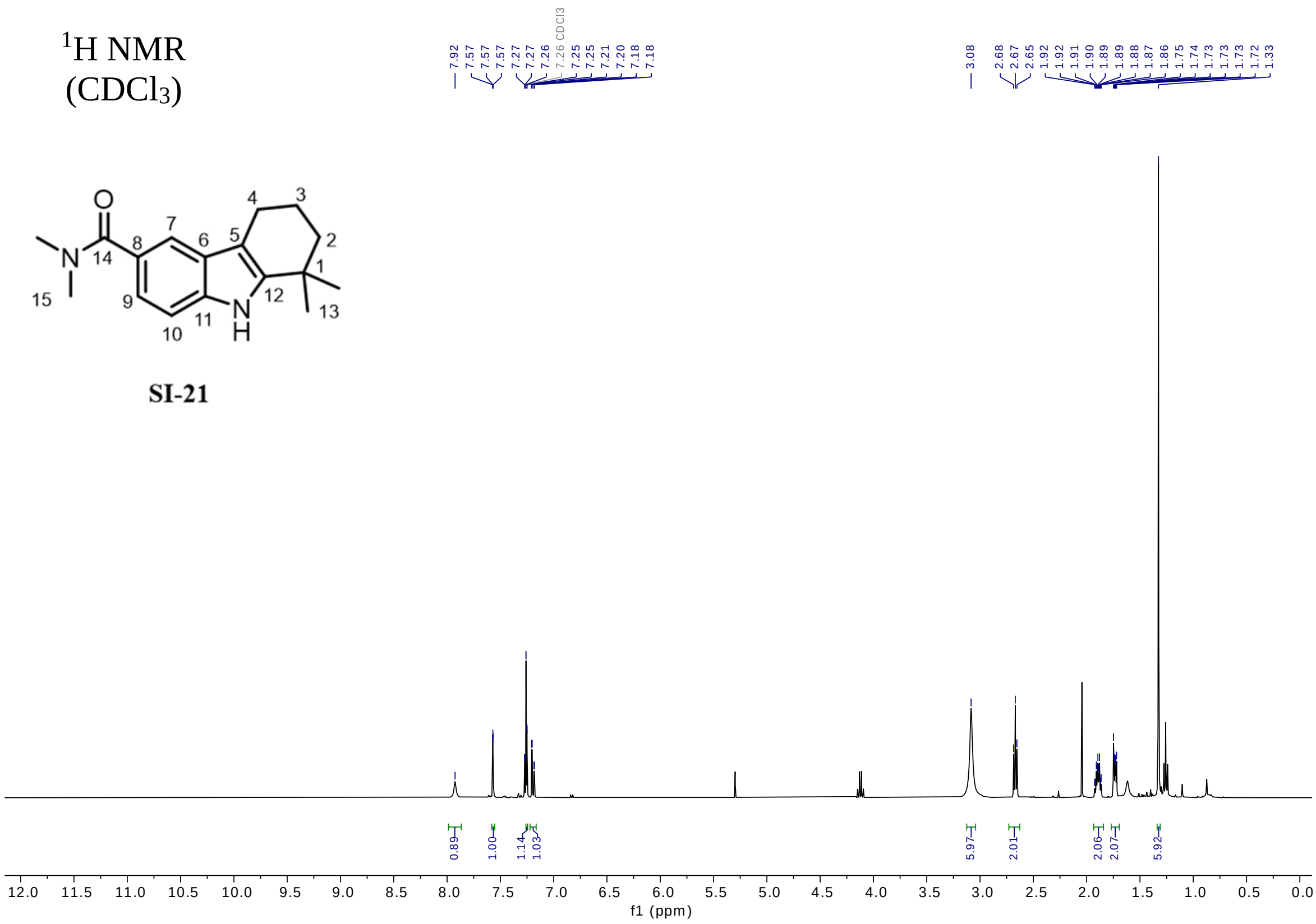

SI-21

$^{13}C\{^{1}H\}$ NMR
($CDCl_3$)

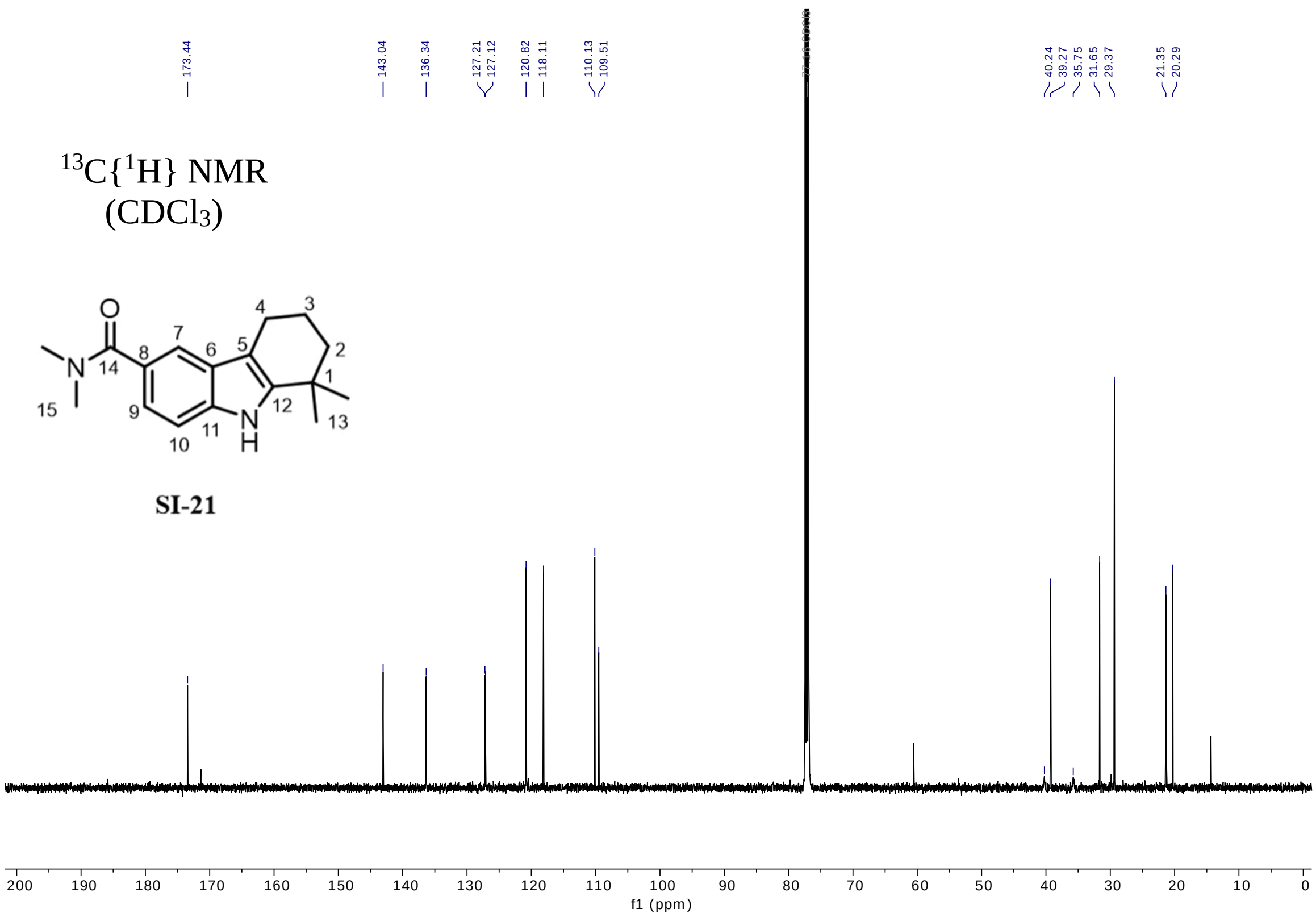

SI-21

^{1}H NMR
($CDCl_3$)

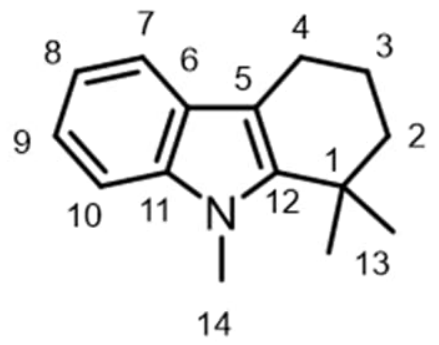

SI-22

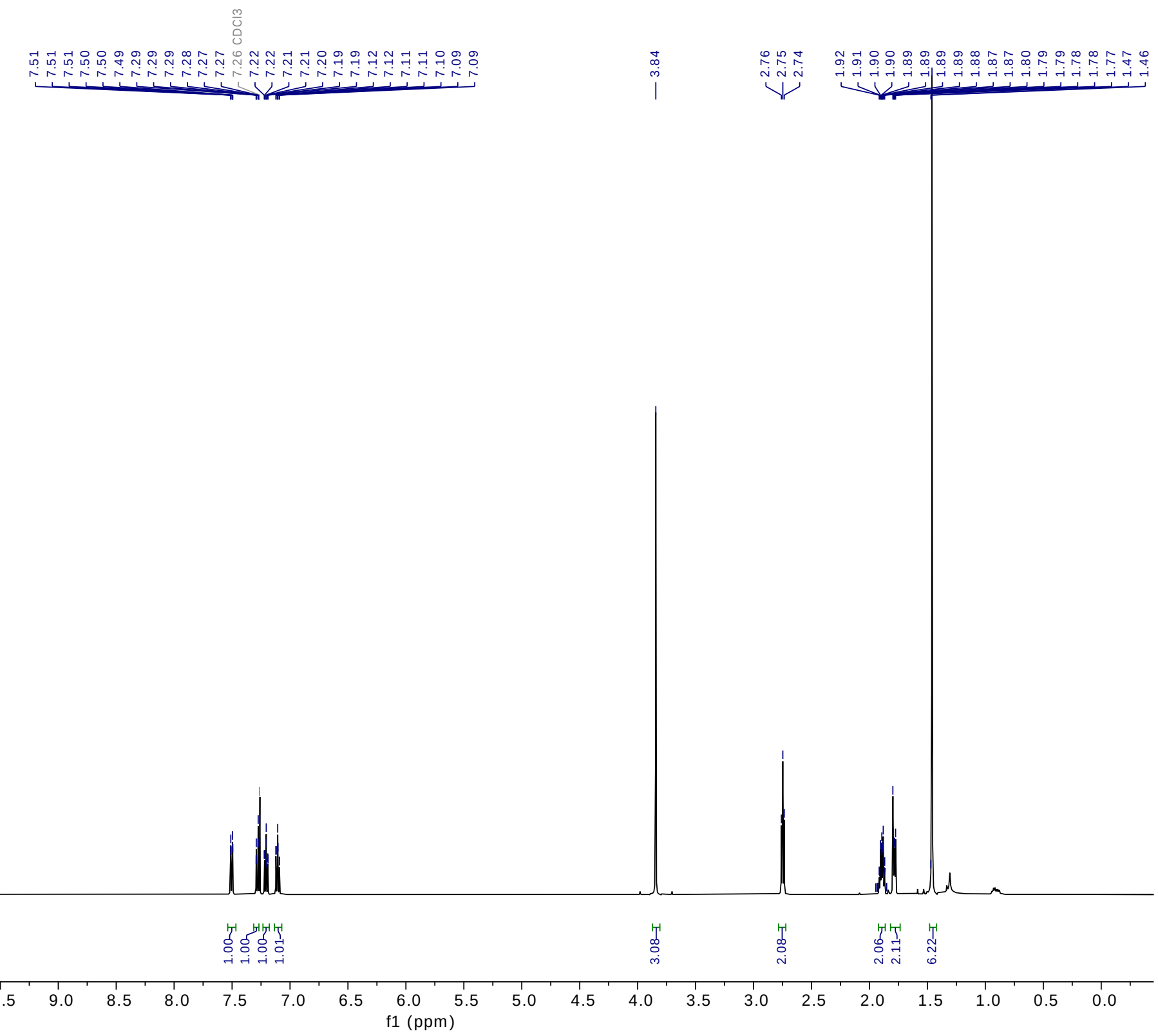

$^{13}C\{^1H\}$ NMR
($CDCl_3$)

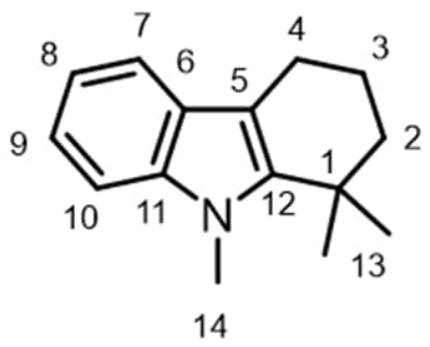

SI-22

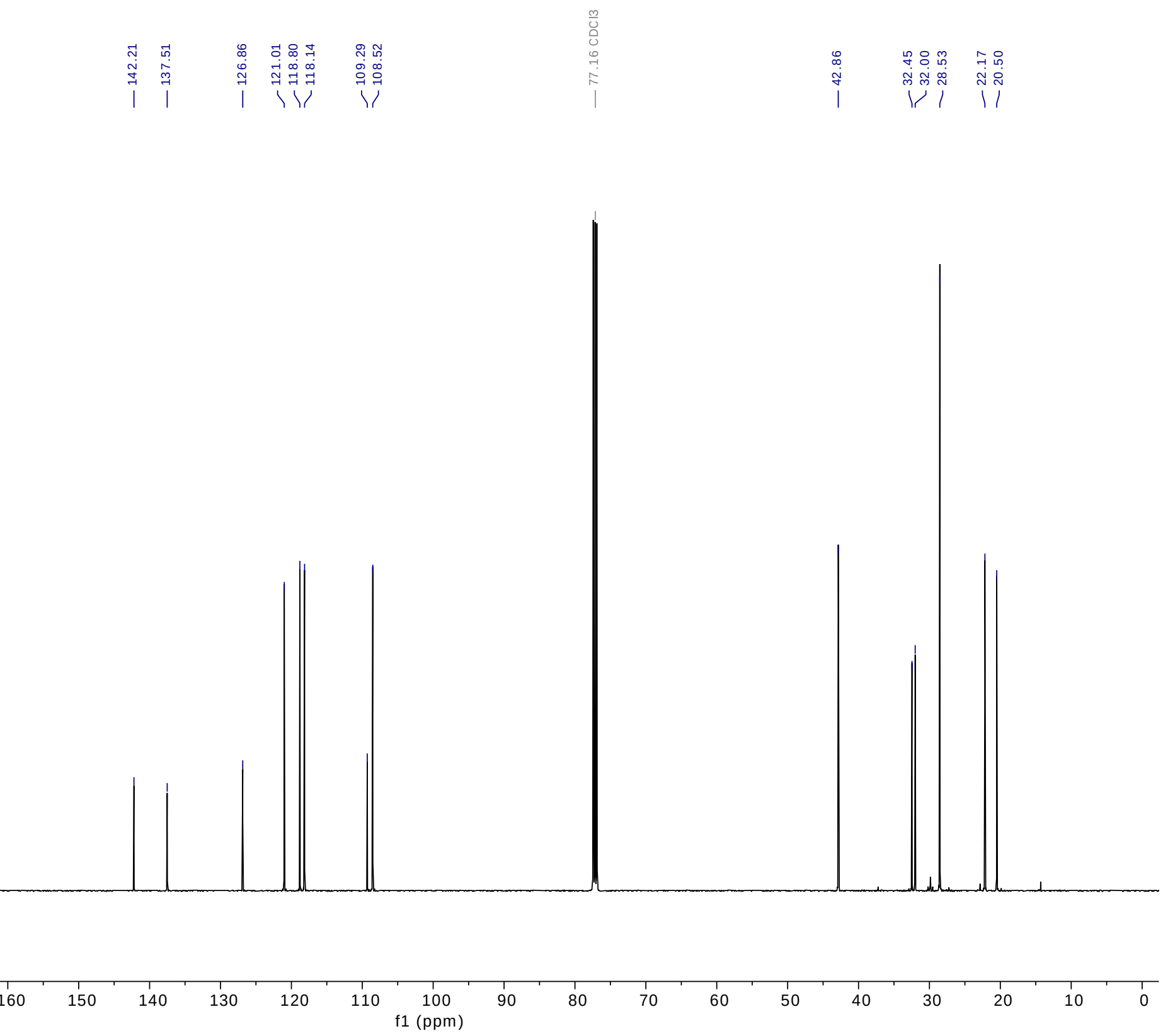

^{1}H NMR
($CDCl_3$)

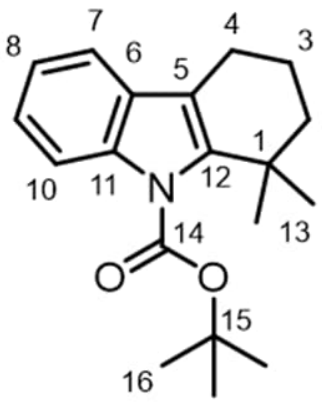

SI-23

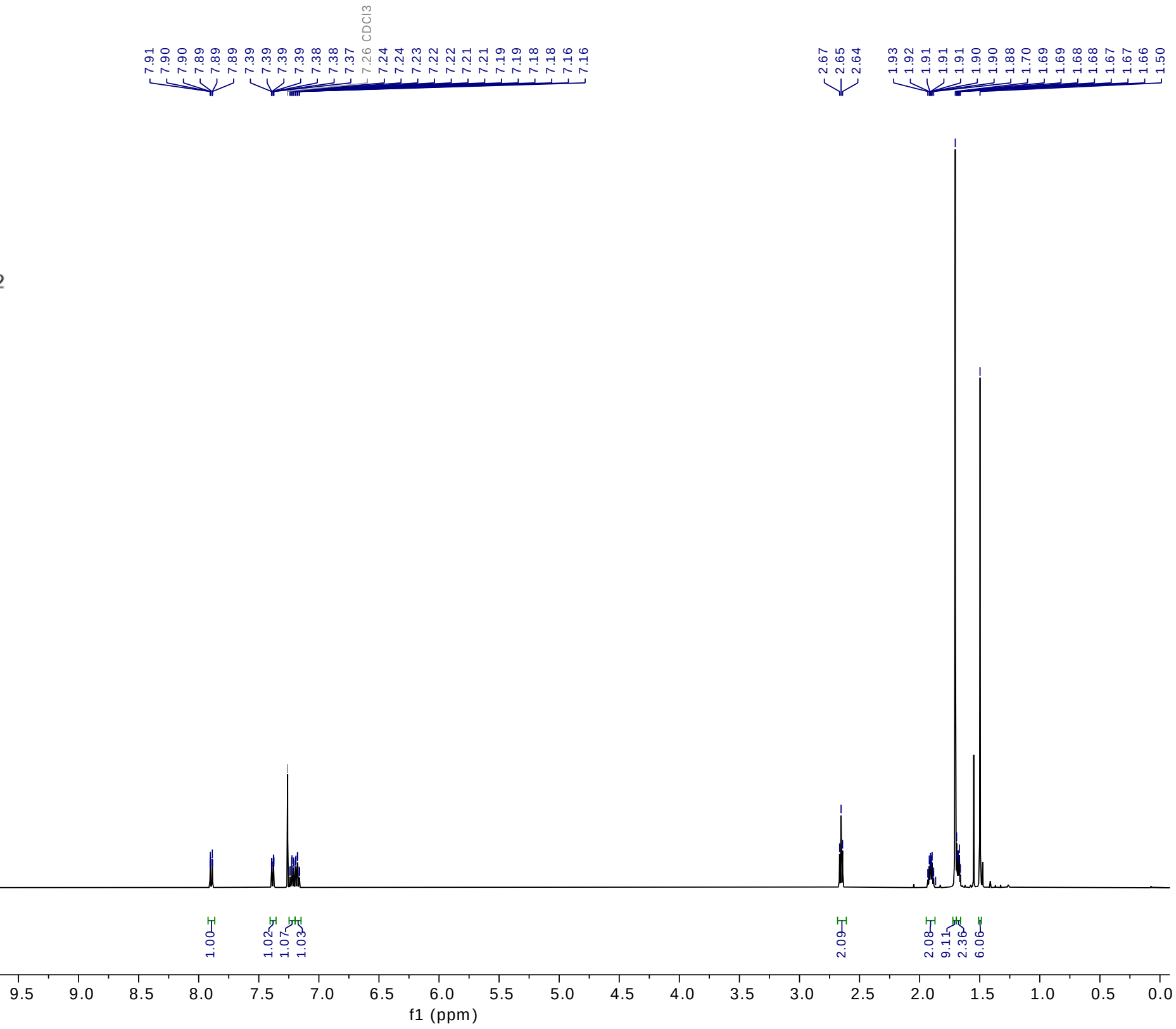

$^{13}C\{^1H\}$ NMR
($CDCl_3$)

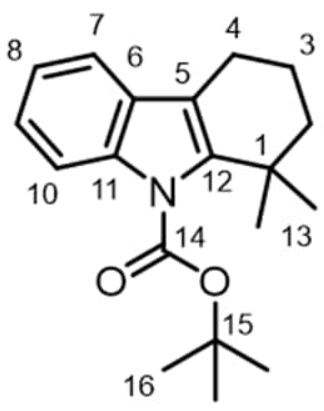

SI-23

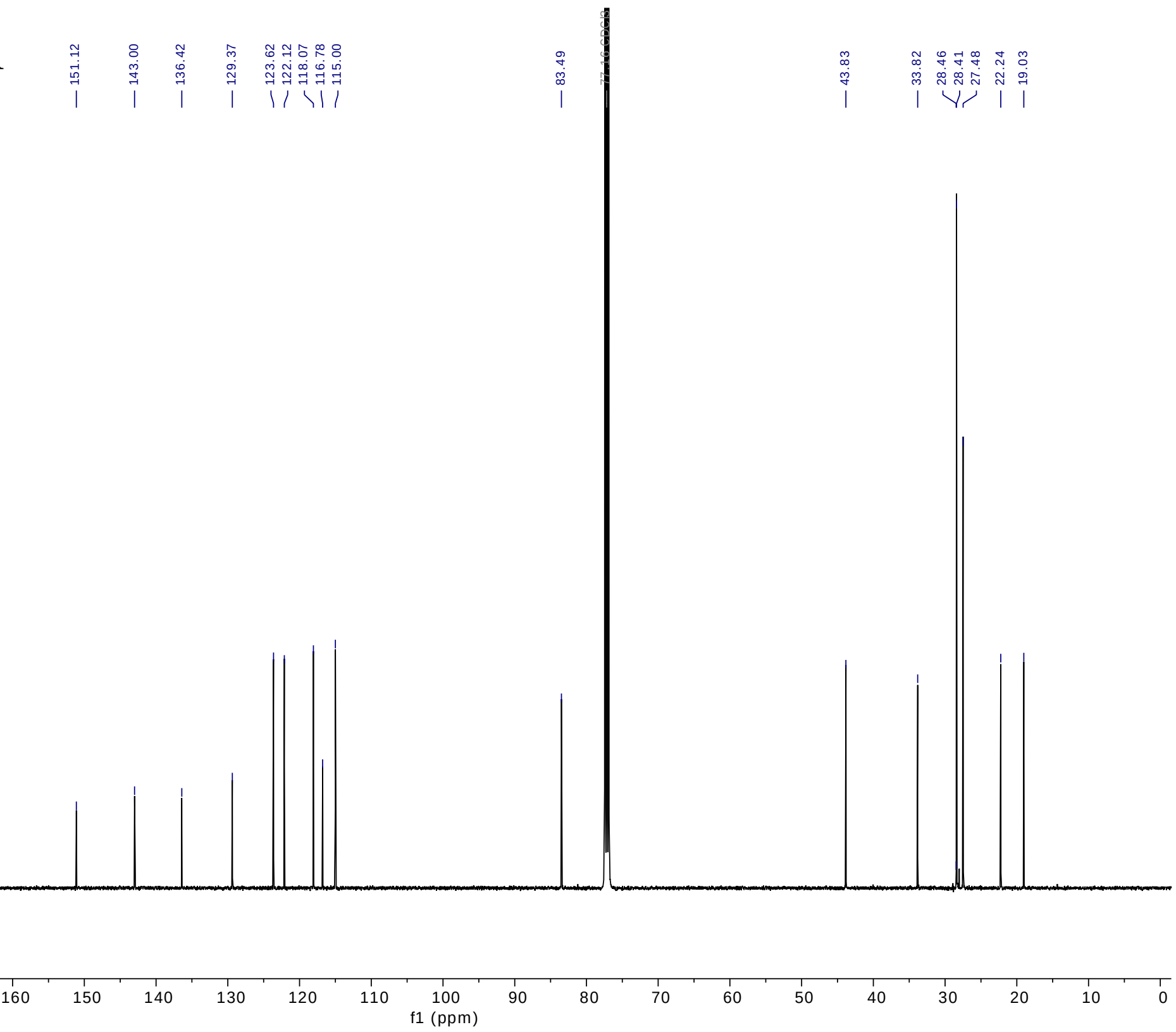

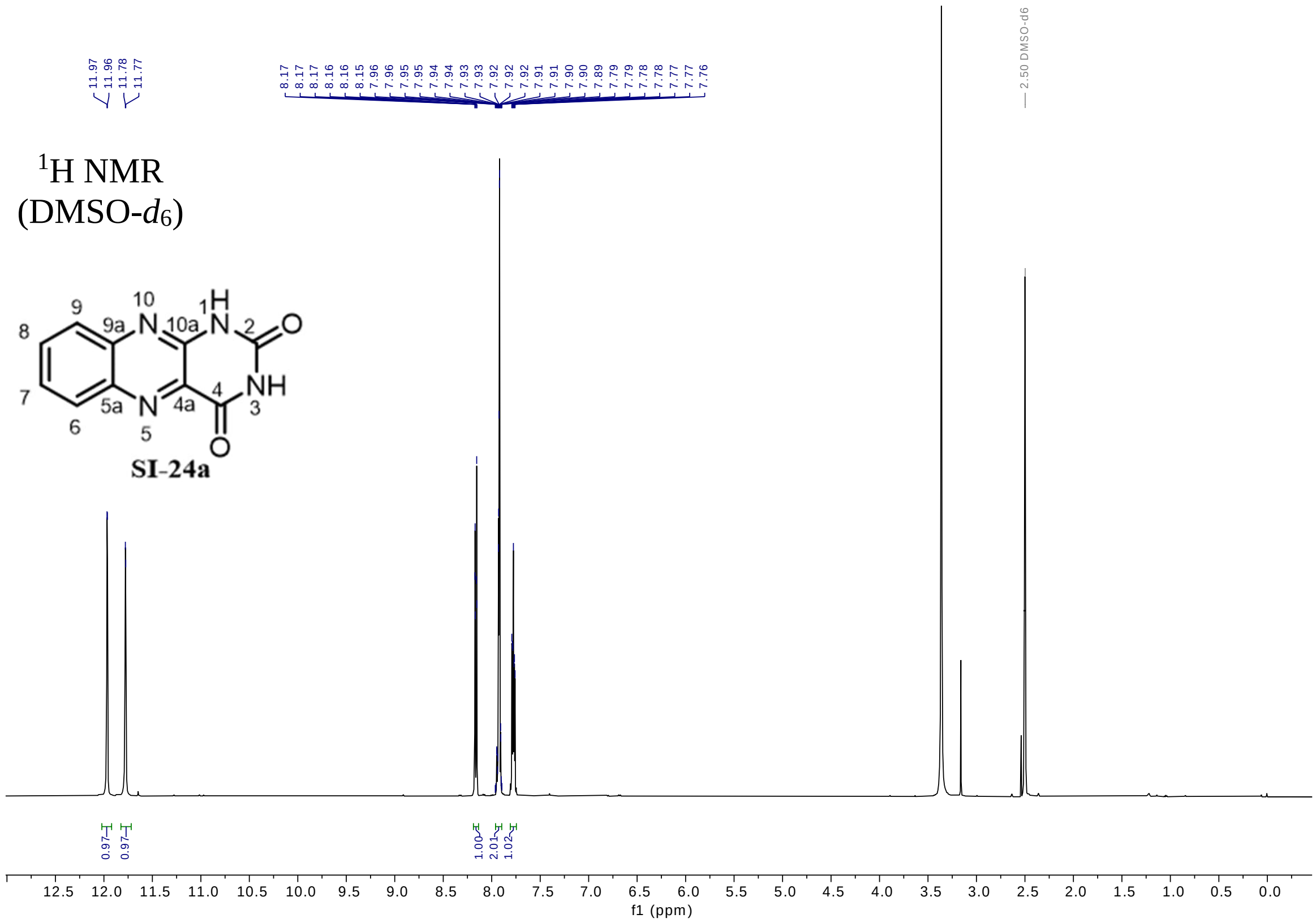

11.97
11.96
11.78
11.77
8.17
8.17
8.17
8.16
8.16
8.15
7.96
7.96
7.95
7.95
7.94
7.94
7.93
7.93
7.92
7.92
7.91
7.91
7.90
7.90
7.89
7.79
7.79
7.78
7.78
7.77
7.77
7.76
2.50 DMSO-d6
1H NMR
(DMSO-d6)
SI-24a
0.97
0.97
1.00
2.01
1.02
f1 (ppm)

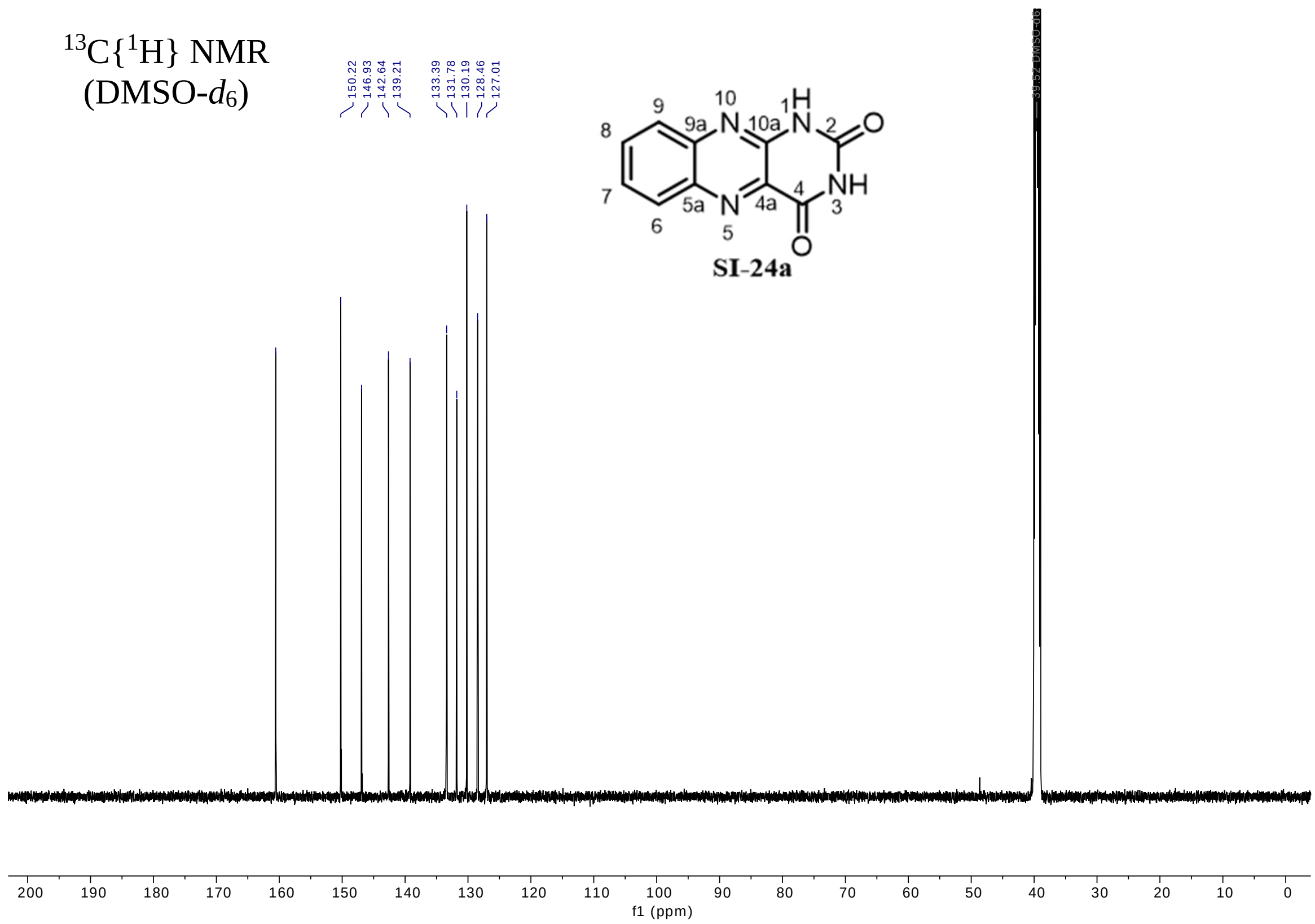

13C{1H} NMR
(DMSO-d6)
150.22
146.93
142.64
139.21
133.39
131.78
130.19
128.46
127.01
SI-24a
f1 (ppm)

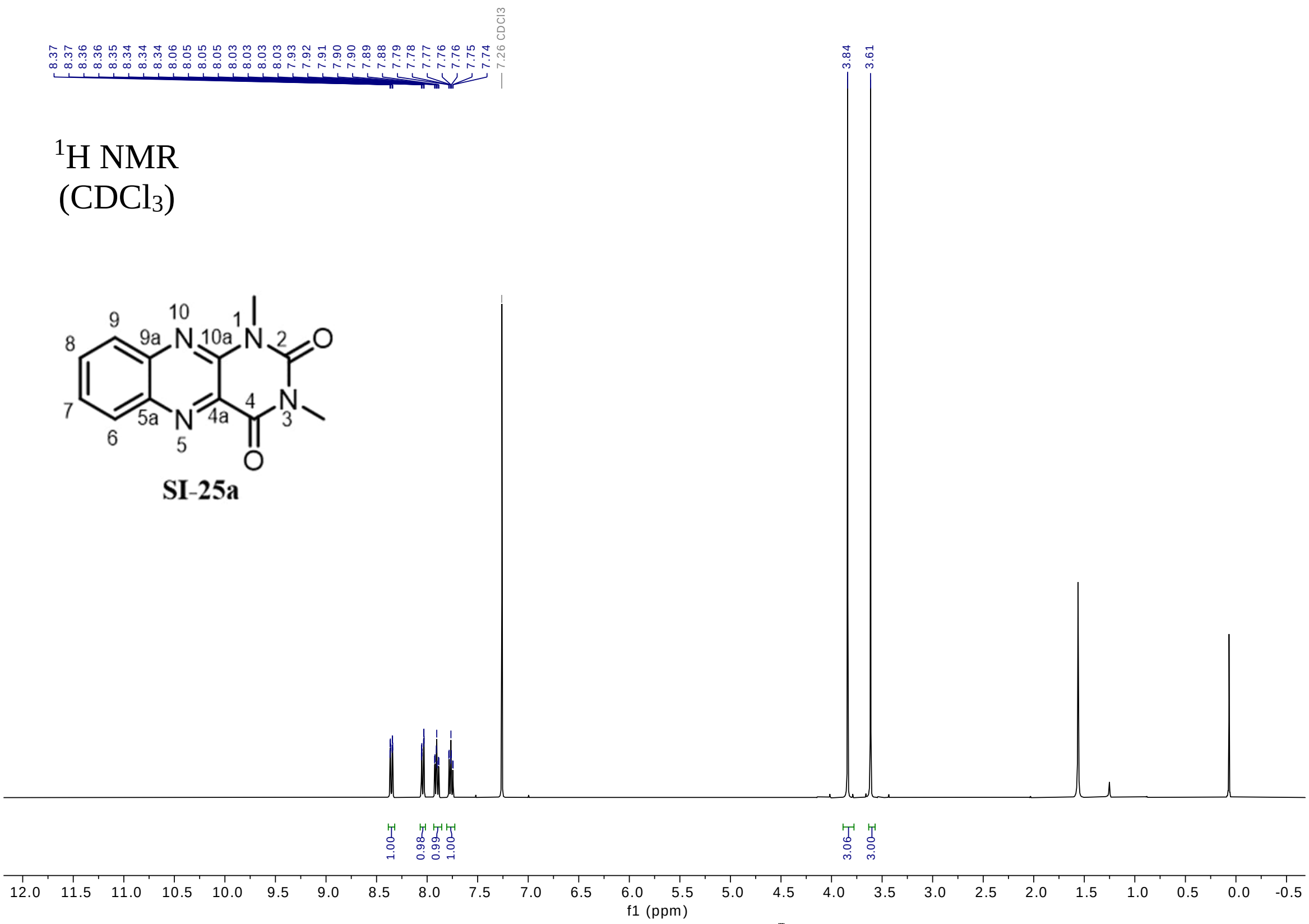
8.37 8.37 8.36 8.35 8.34 8.34 8.34 8.06 8.05 8.05 8.05 8.03 8.03 8.03 8.03 7.93 7.92 7.91 7.90 7.90 7.89 7.88 7.79 7.78 7.77 7.76 7.76 7.75 7.74
7.26 CDCl3
3.84
3.61
^1H NMR
($CDCl_3$)
SI-25a
1.00 0.98 0.99 1.00
3.06 3.00
f1 (ppm)

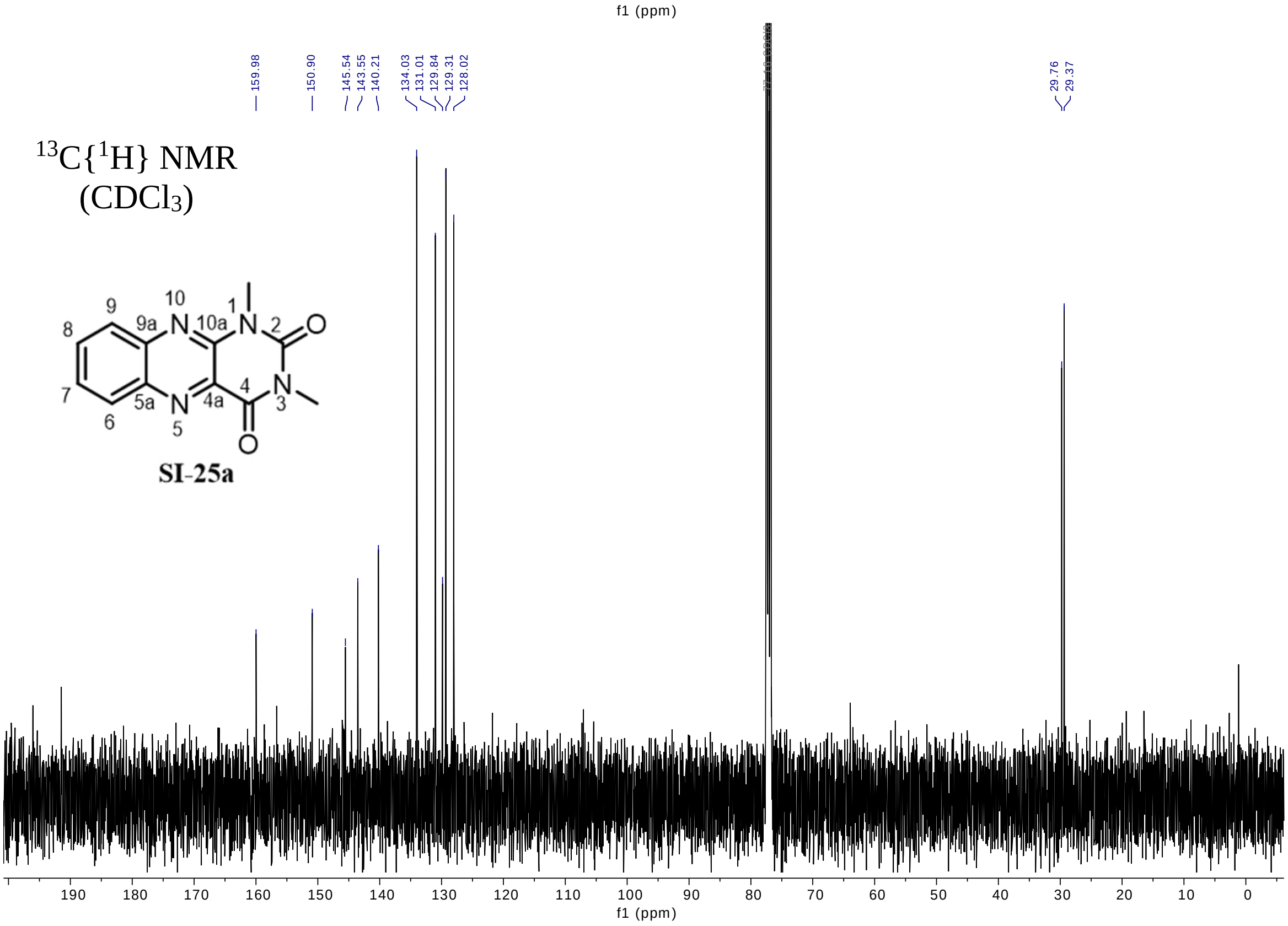
159.98
150.90
145.54 143.55 140.21
134.03 131.01 129.84 129.31 128.02
29.76 29.37
$^{13}C\{^1H\}$ NMR
($CDCl_3$)
SI-25a
f1 (ppm)

^{1}H NMR
($CDCl_3$)

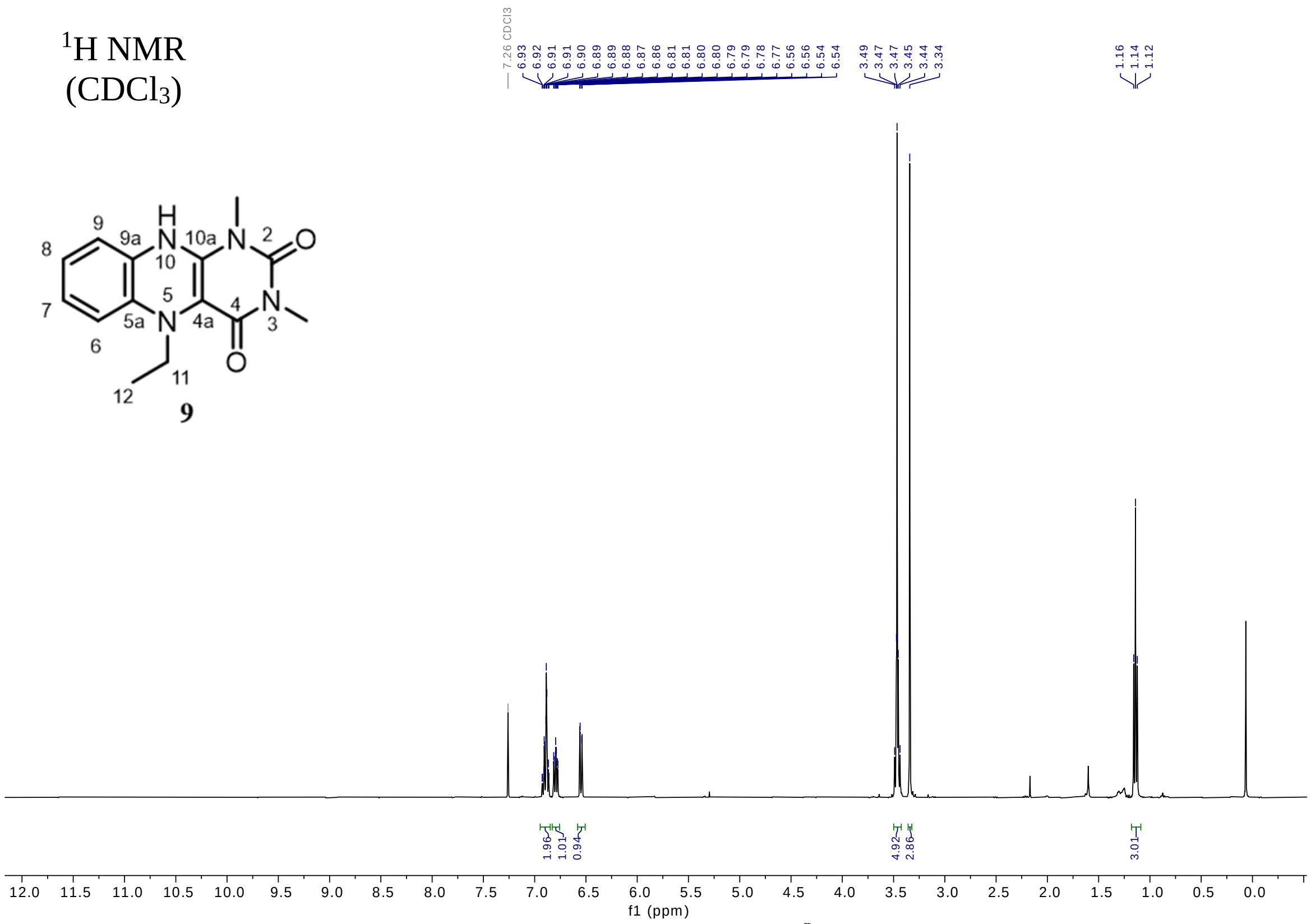

9

$^{13}C\{^{1}H\}$ NMR
($CDCl_3$)

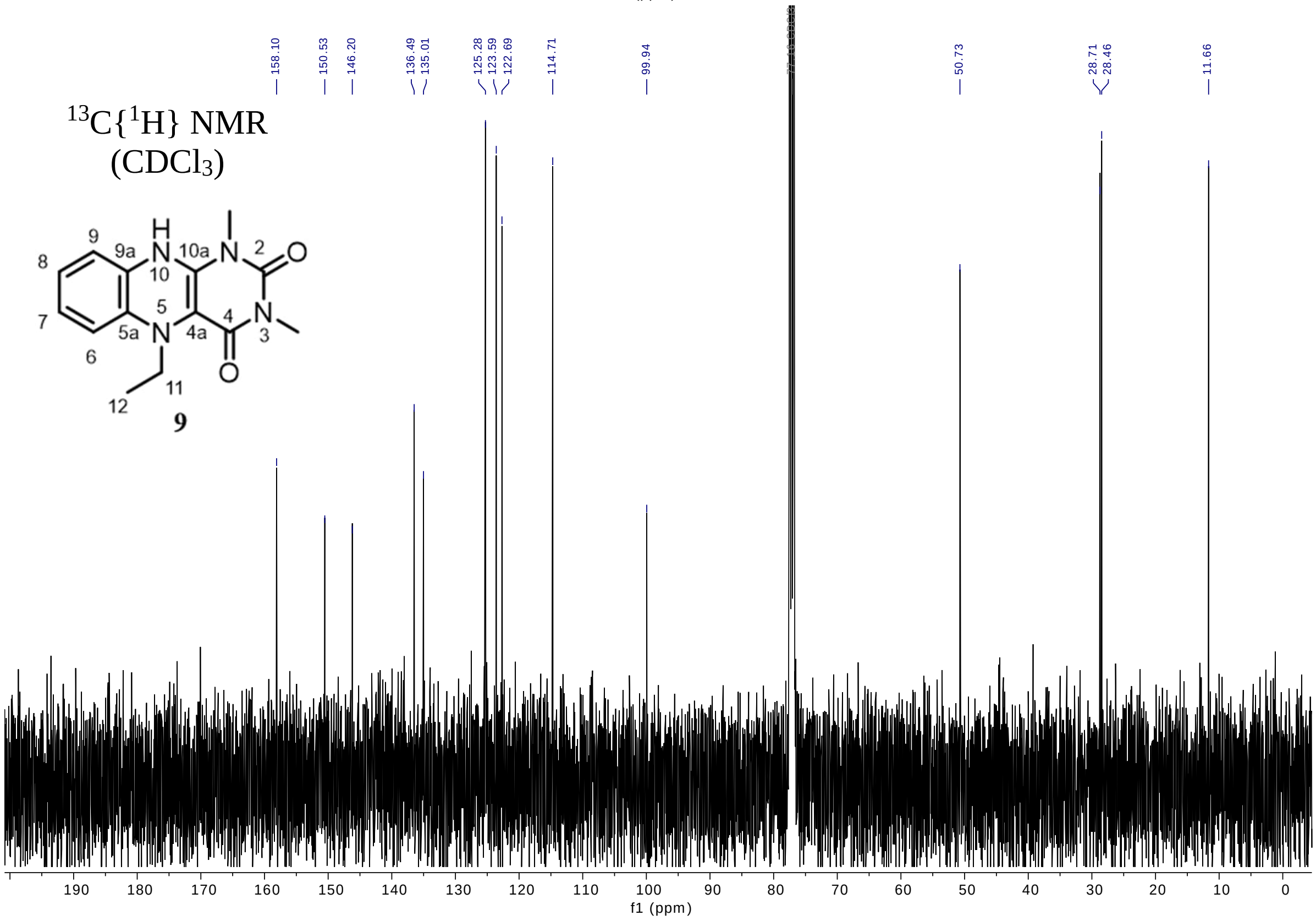

9

^{1}H NMR
(CD_3CN)

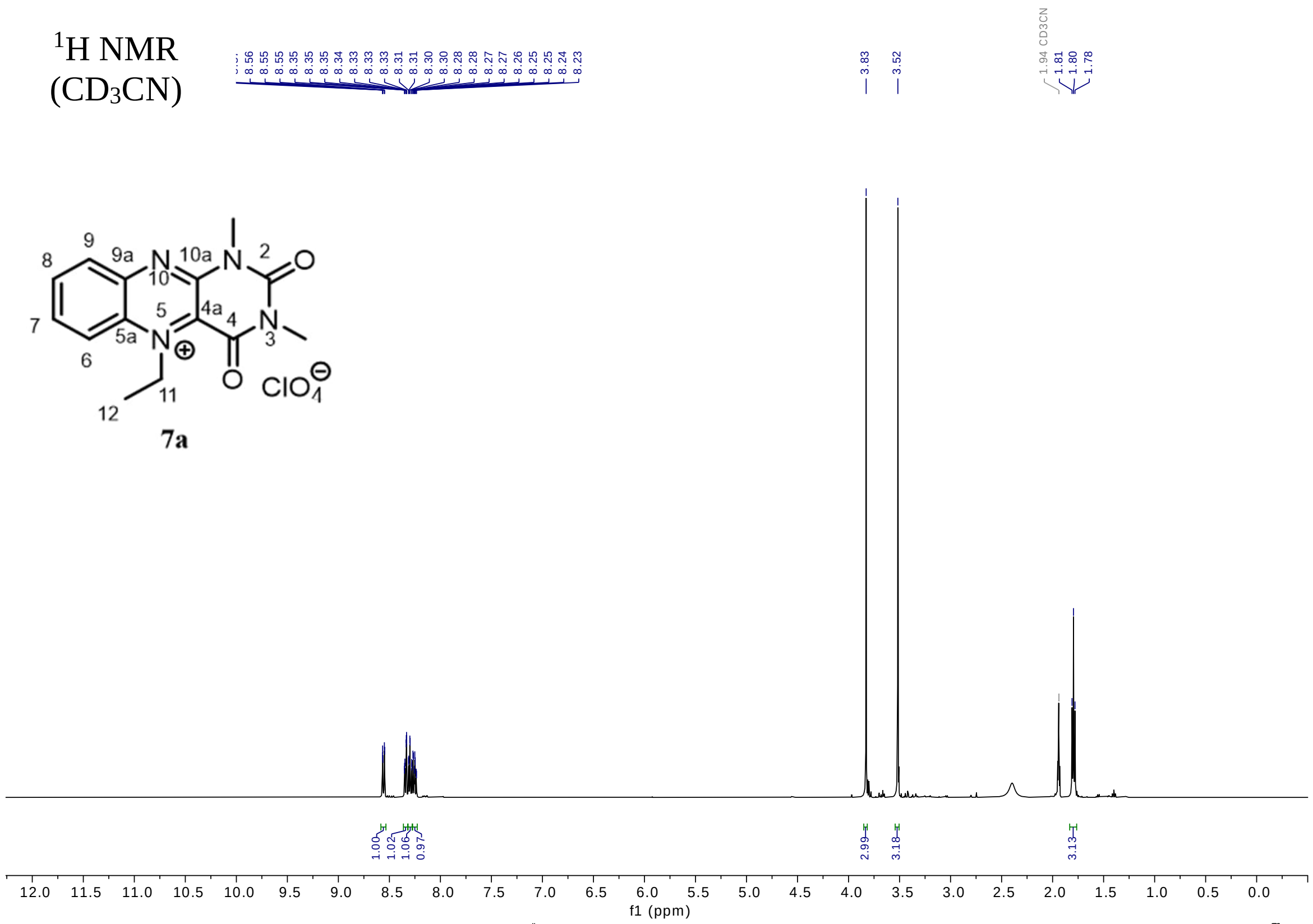

7a

$^{13}C\{^{1}H\}$ NMR
(CD_3CN)

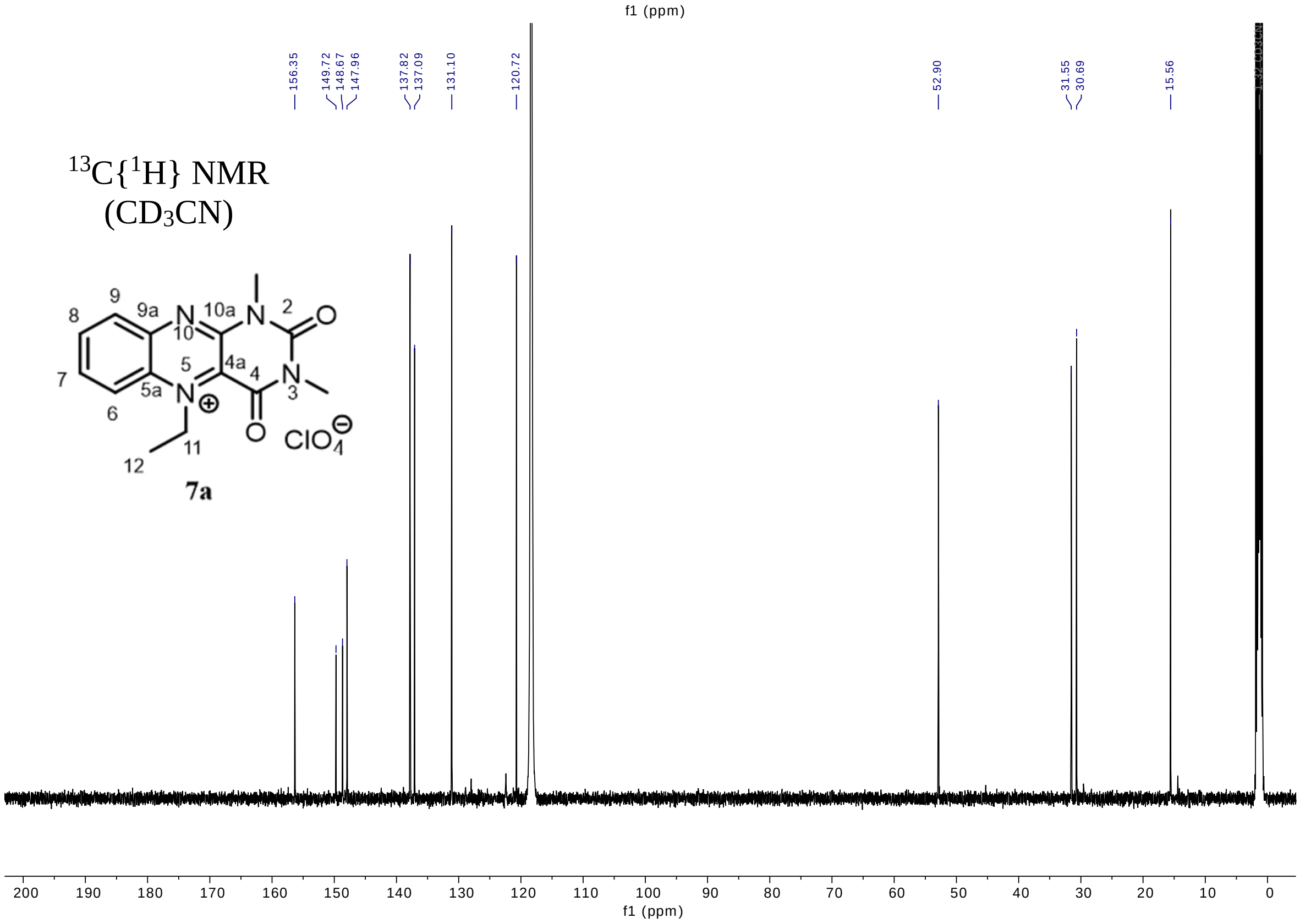

7a

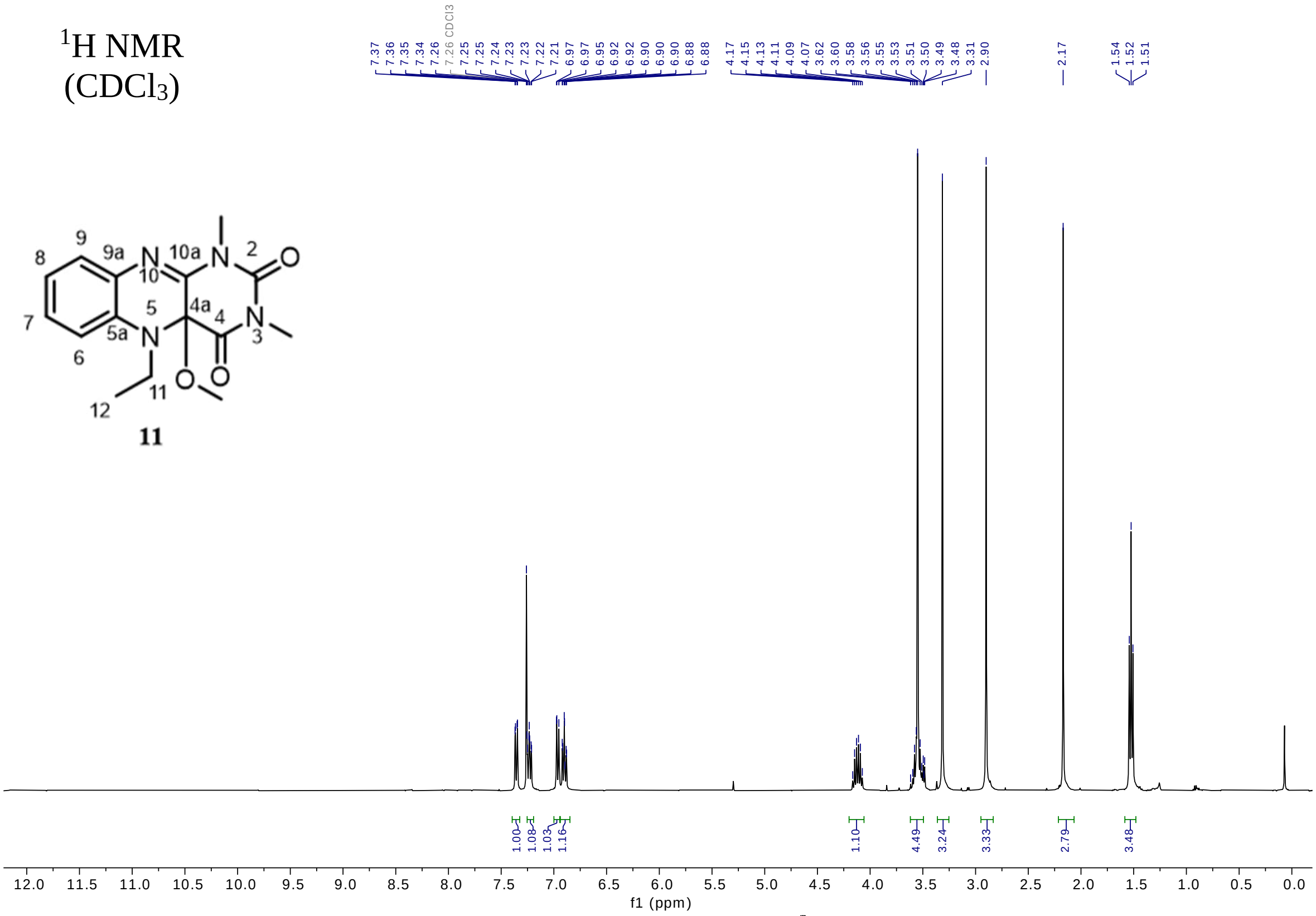
^{1}H NMR
($CDCl_3$)
11
7.37
7.36
7.35
7.34
7.26
7.26 CDCl3
7.25
7.25
7.24
7.23
7.23
7.22
7.21
6.97
6.97
6.95
6.92
6.92
6.90
6.90
6.90
6.88
6.88
4.17
4.15
4.13
4.11
4.09
4.07
3.62
3.60
3.58
3.56
3.55
3.53
3.51
3.50
3.49
3.48
3.31
2.90
2.17
1.54
1.52
1.51
1.00
1.08
1.03
1.16
1.10
4.49
3.24
3.33
2.79
3.48
f1 (ppm)

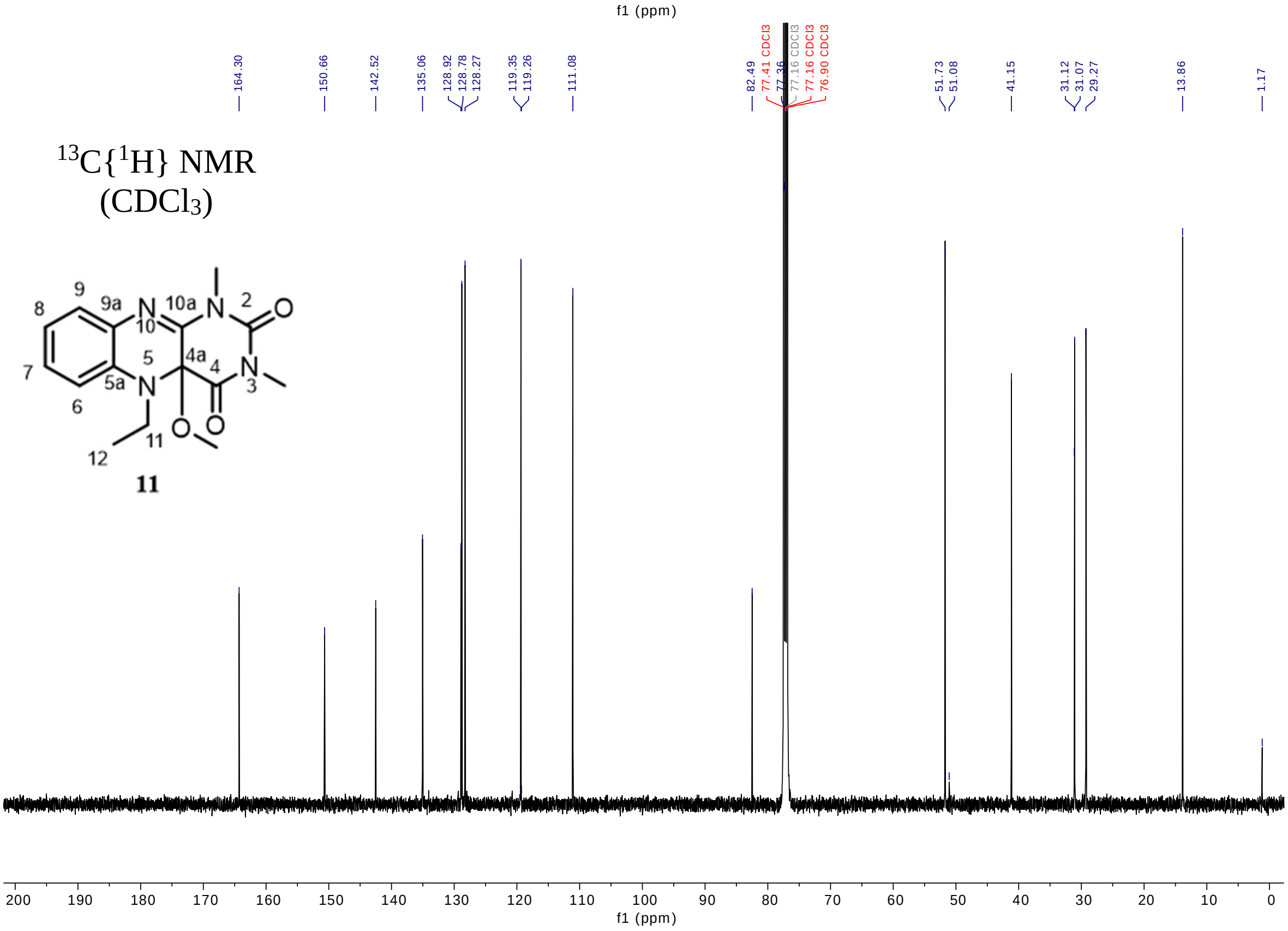
^{13}C{^{1}H} NMR
($CDCl_3$)
11
164.30
150.66
142.52
135.06
128.92
128.78
128.27
119.35
119.26
111.08
82.49
77.41 CDCl3
77.36
77.16 CDCl3
77.16 CDCl3
76.90 CDCl3
51.73
51.08
41.15
31.12
31.07
29.27
13.86
1.17
f1 (ppm)

^{1}H NMR
($CDCl_3$)

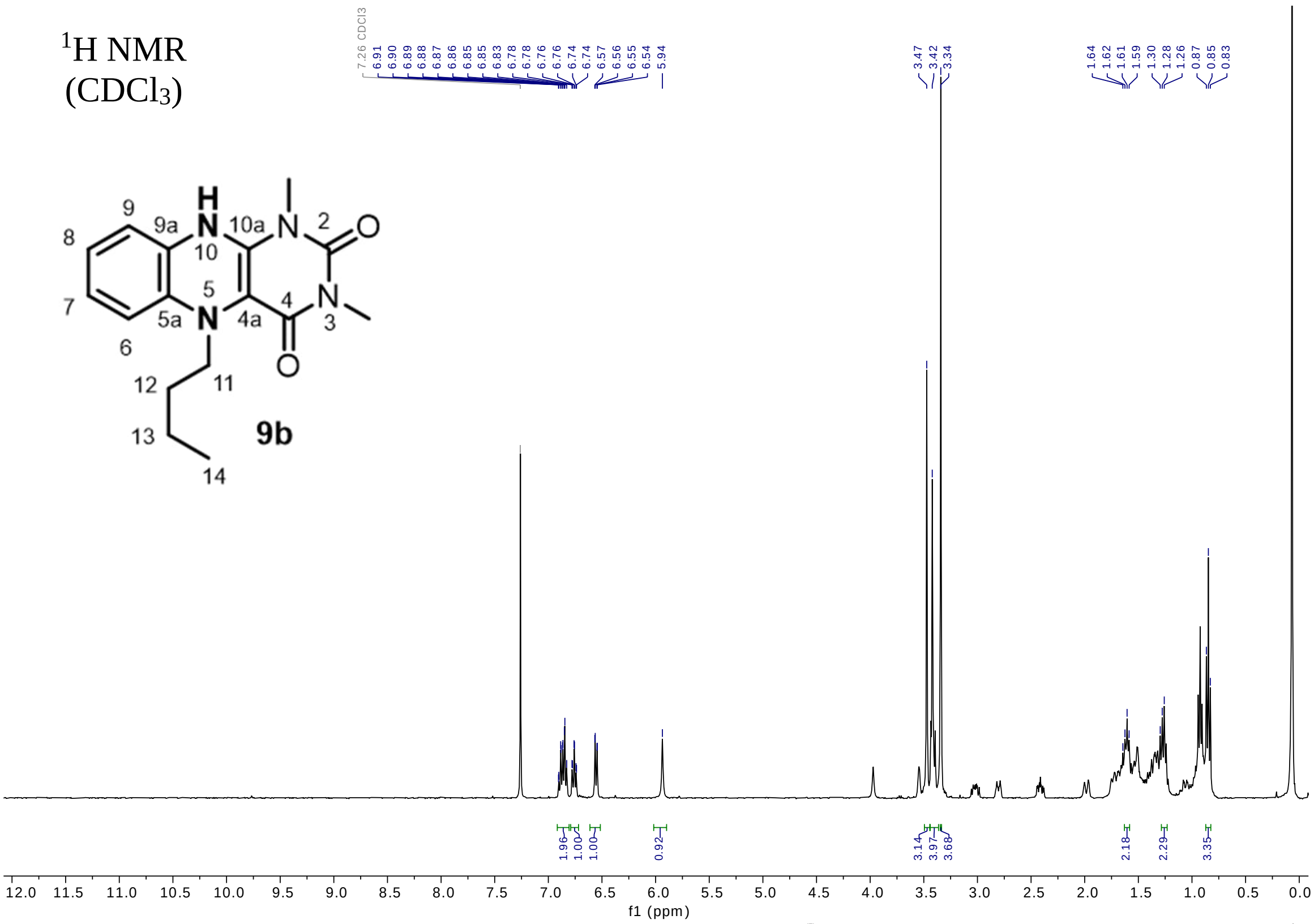

$^{13}C\{^{1}H\}$ NMR
($CDCl_3$)

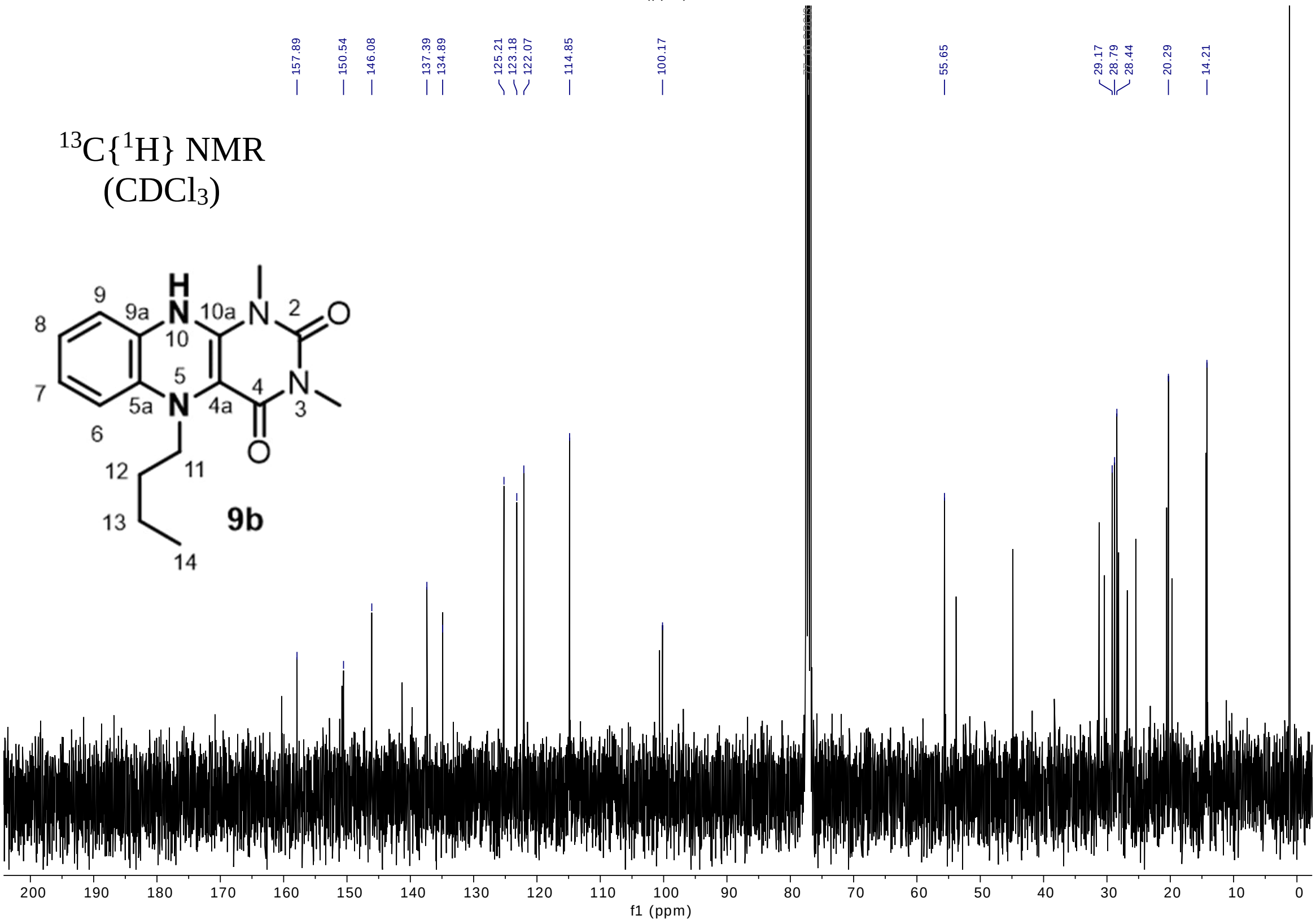

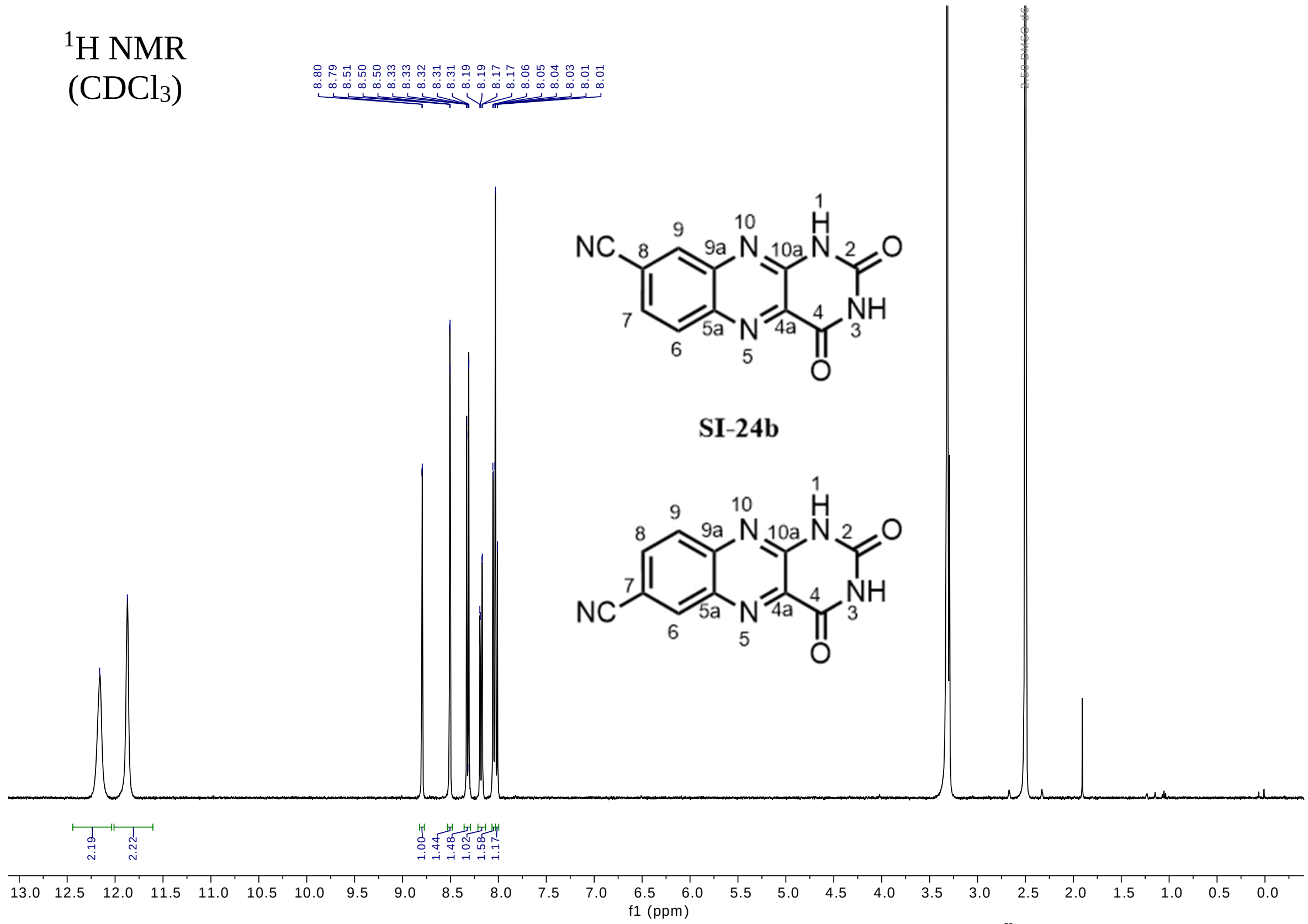
1H NMR
(CDCl3)
8.80 8.79 8.51 8.50 8.50 8.33 8.33 8.32 8.31 8.31 8.19 8.19 8.17 8.17 8.06 8.05 8.04 8.03 8.01 8.01
2.19 2.22 1.00 1.44 1.48 1.02 1.58 1.17
SI-24b
f1 (ppm)

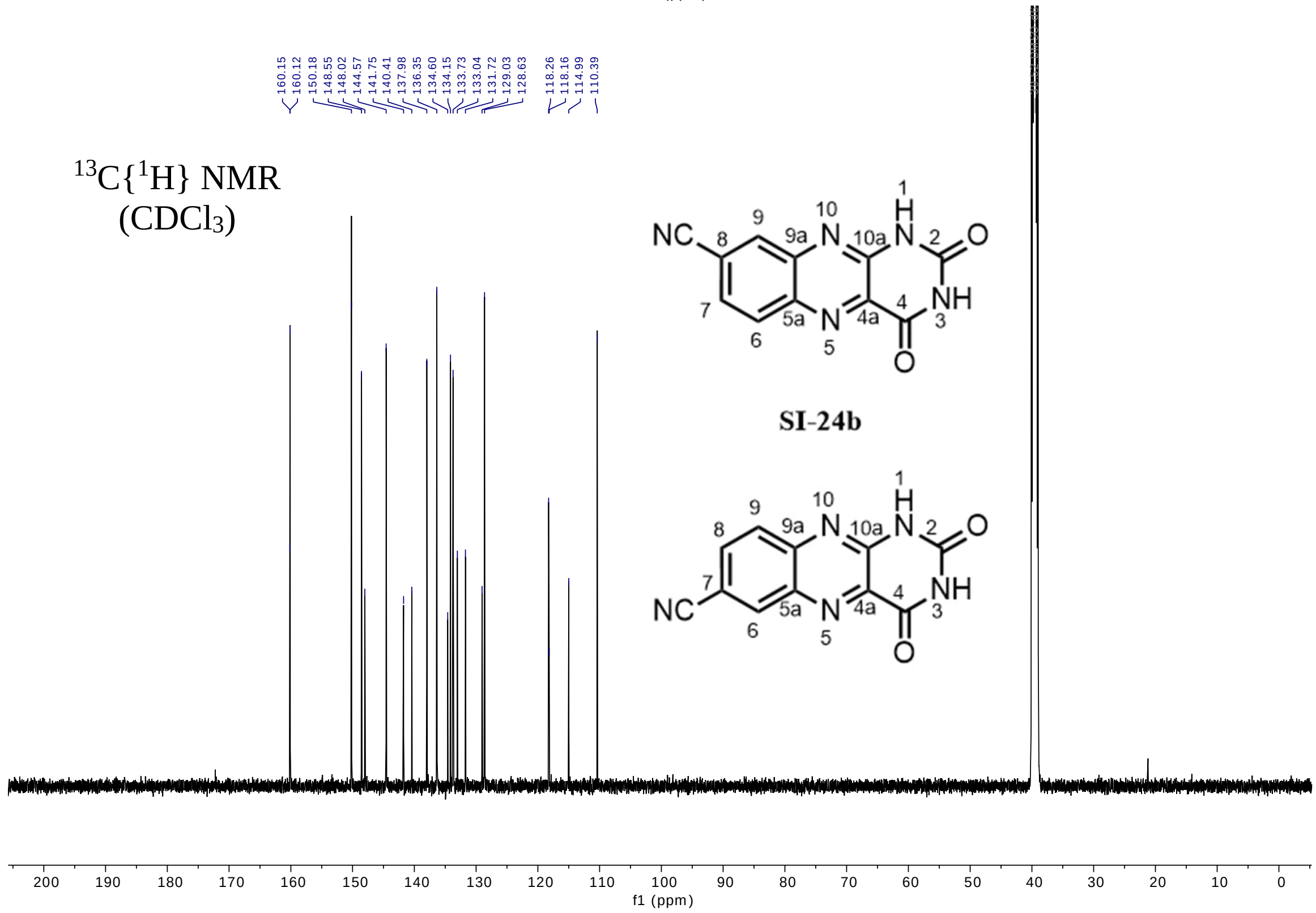
13C{1H} NMR
(CDCl3)
160.15 160.12 150.18 148.55 148.02 144.57 141.75 140.41 137.98 136.35 134.60 134.15 133.73 133.04 131.72 129.03 128.63 118.26 118.16 114.99 110.39
SI-24b
f1 (ppm)

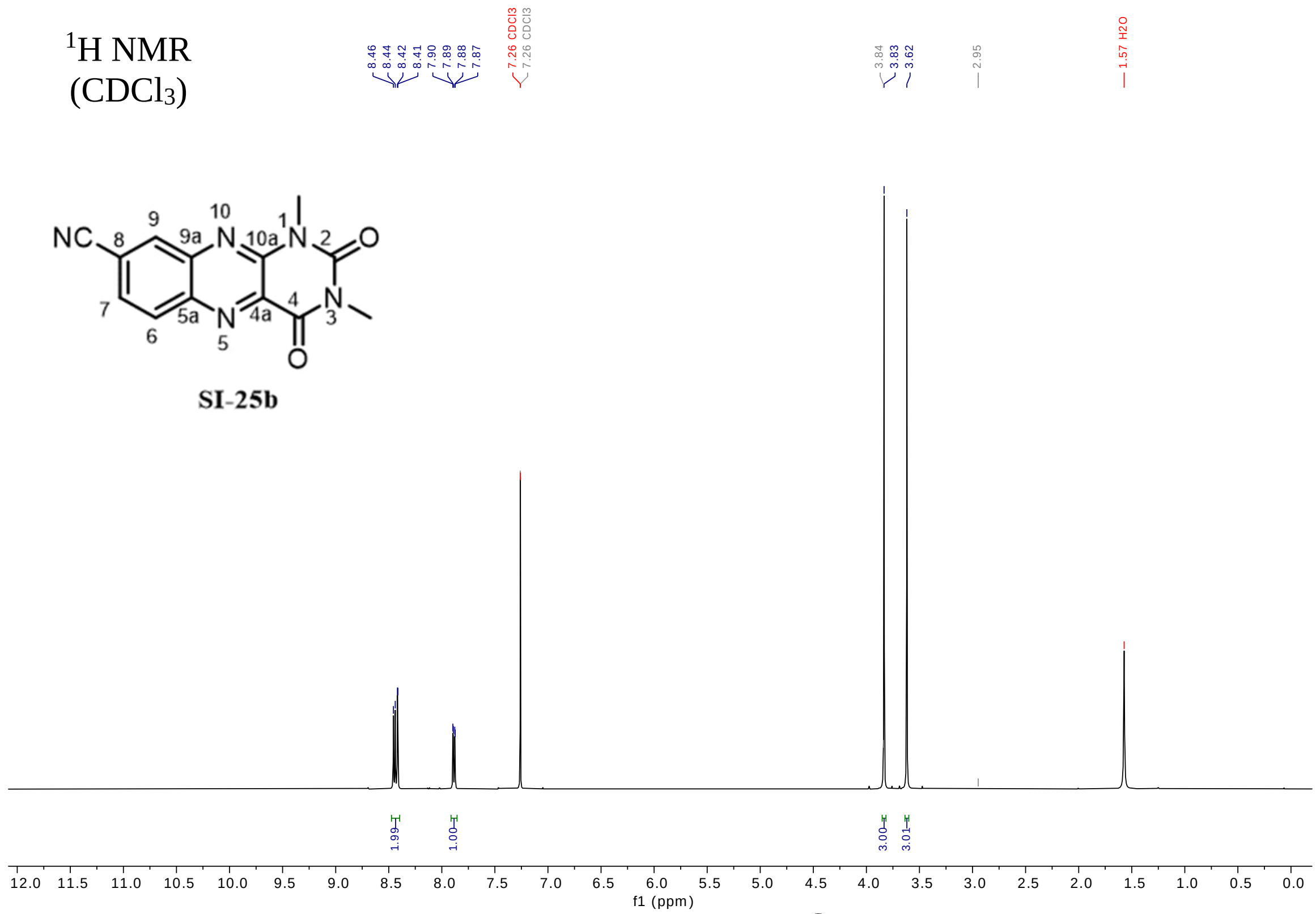

¹H NMR
(CDCl3)
SI-25b
8.46 8.44 8.42 8.41 7.90 7.89 7.88 7.87
7.26 CDCl3
7.26 CDCl3
3.84 3.83 3.62
2.95
1.57 H2O
1.99
1.00
3.00
3.01
f1 (ppm)

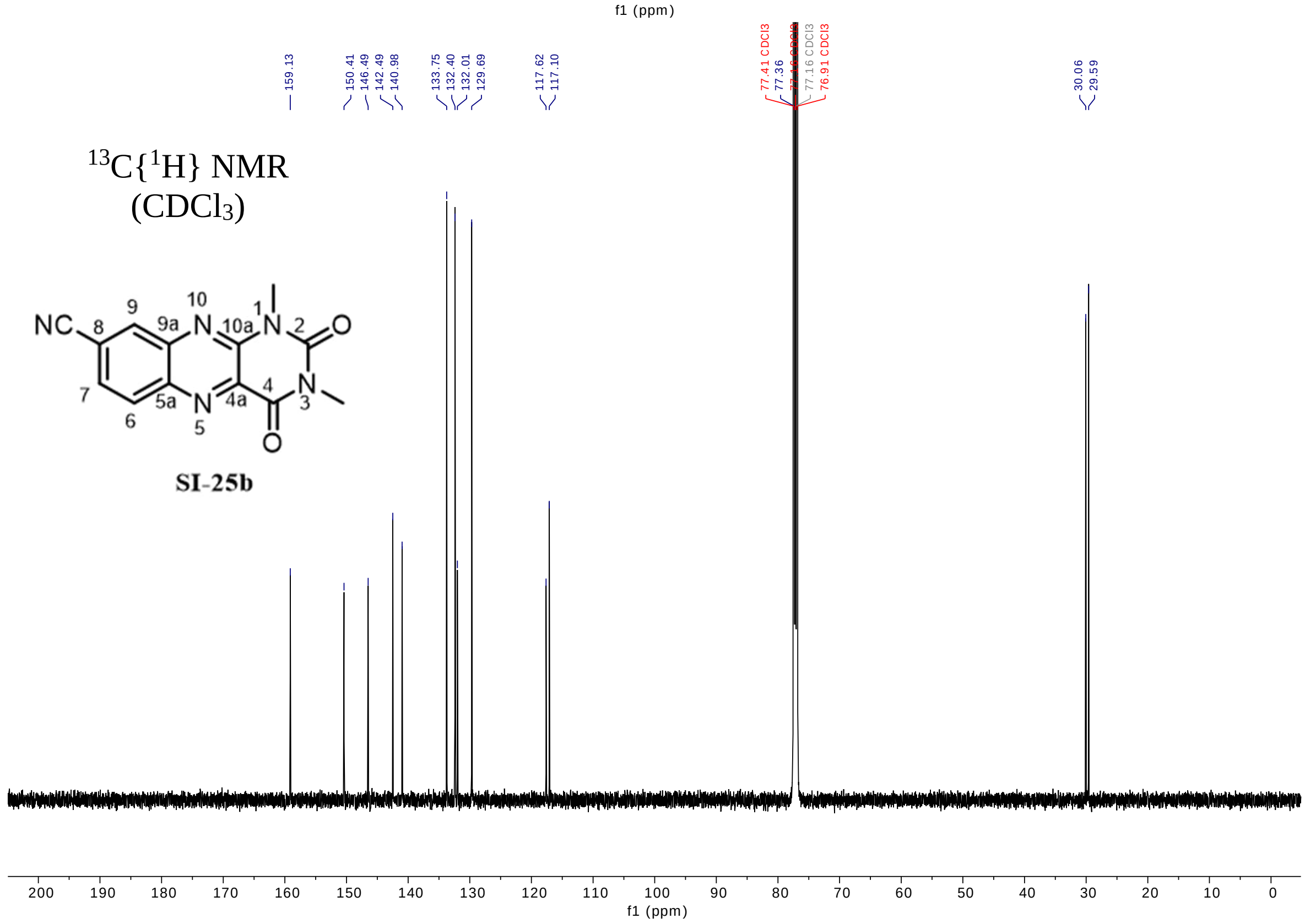

¹³C{¹H} NMR
(CDCl3)
SI-25b
159.13
150.41 146.49 142.49 140.98
133.75 132.40 132.01 129.69
117.62 117.10
77.41 CDCl3
77.36
77.16 CDCl3
76.91 CDCl3
30.06 29.59
f1 (ppm)

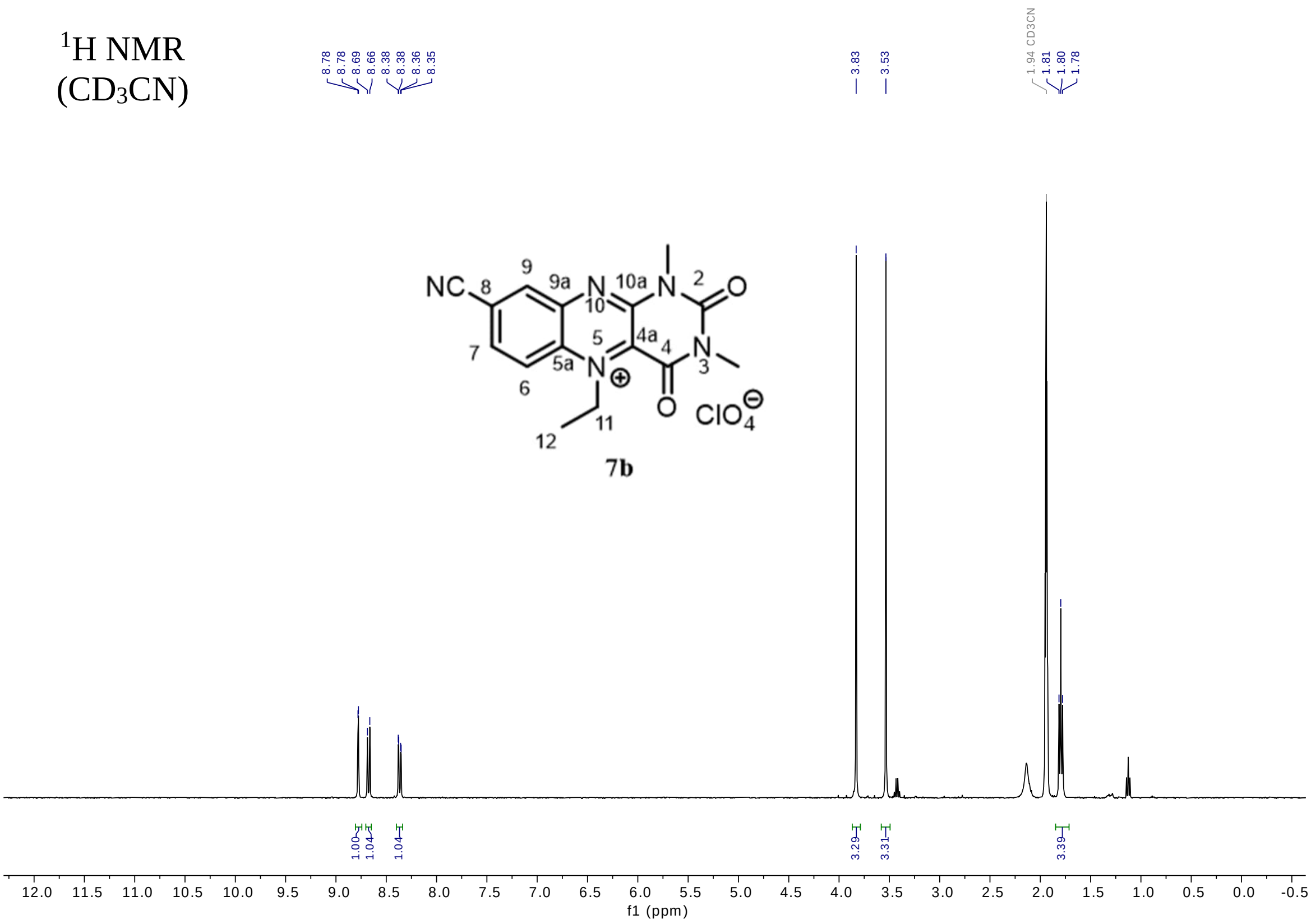

^{1}H NMR
(CD_3CN)
8.78
8.78
8.69
8.66
8.38
8.38
8.36
8.35
3.83
3.53
1.94 CD3CN
1.81
1.80
1.78
NC
8
9
9a
N
10
10a
N
2
O
7
6
5a
5
N
4a
4
N
3
O
11
12
ClO4
7b
1.00
1.04
1.04
3.29
3.31
3.39
f1 (ppm)

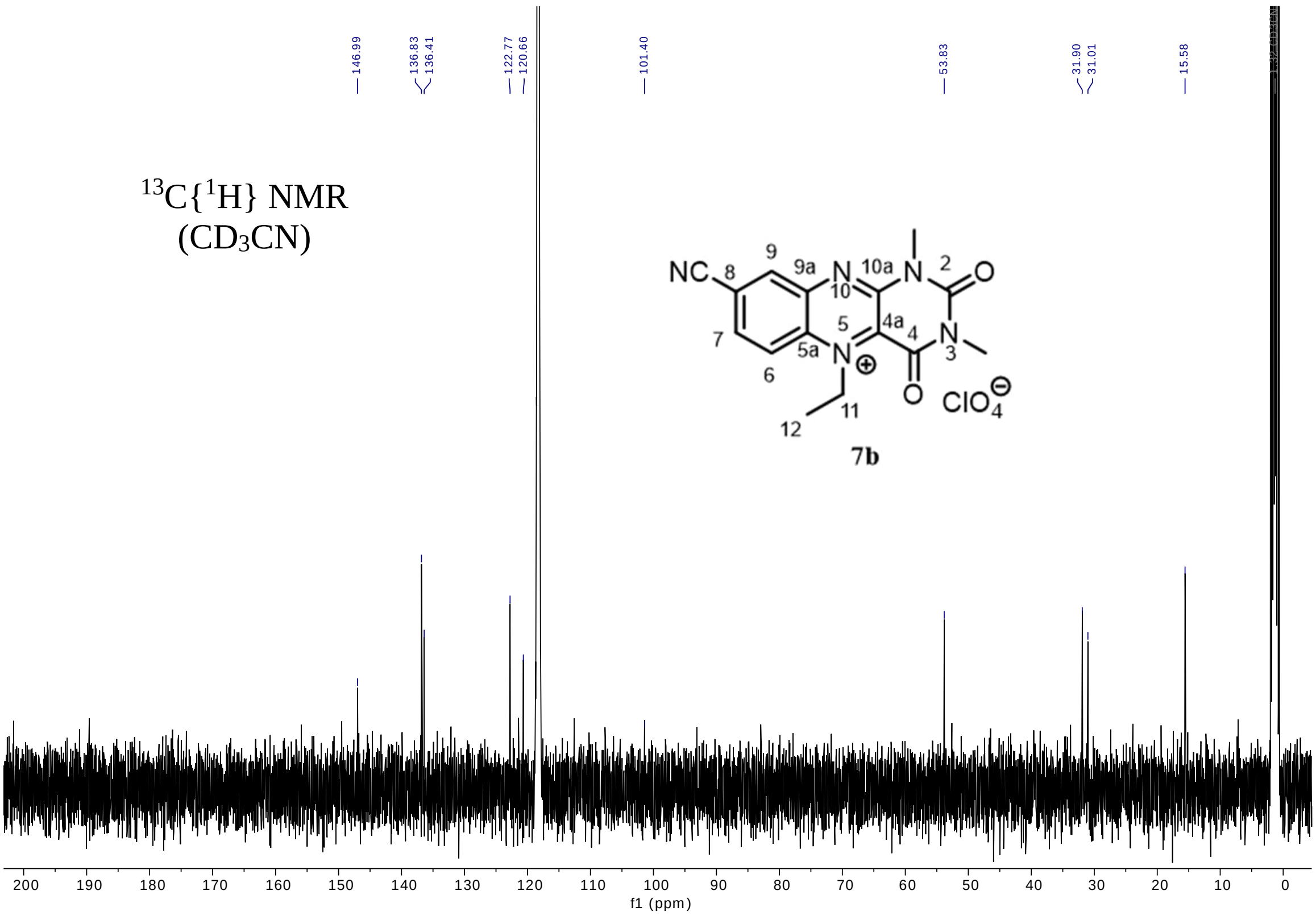

146.99
136.83
136.41
122.77
120.66
101.40
53.83
31.90
31.01
15.58
^{13}C{^{1}H} NMR
(CD_3CN)
NC
8
9
9a
N
10
10a
N
2
O
7
6
5a
5
N
4a
4
N
3
O
11
12
ClO4
7b
f1 (ppm)

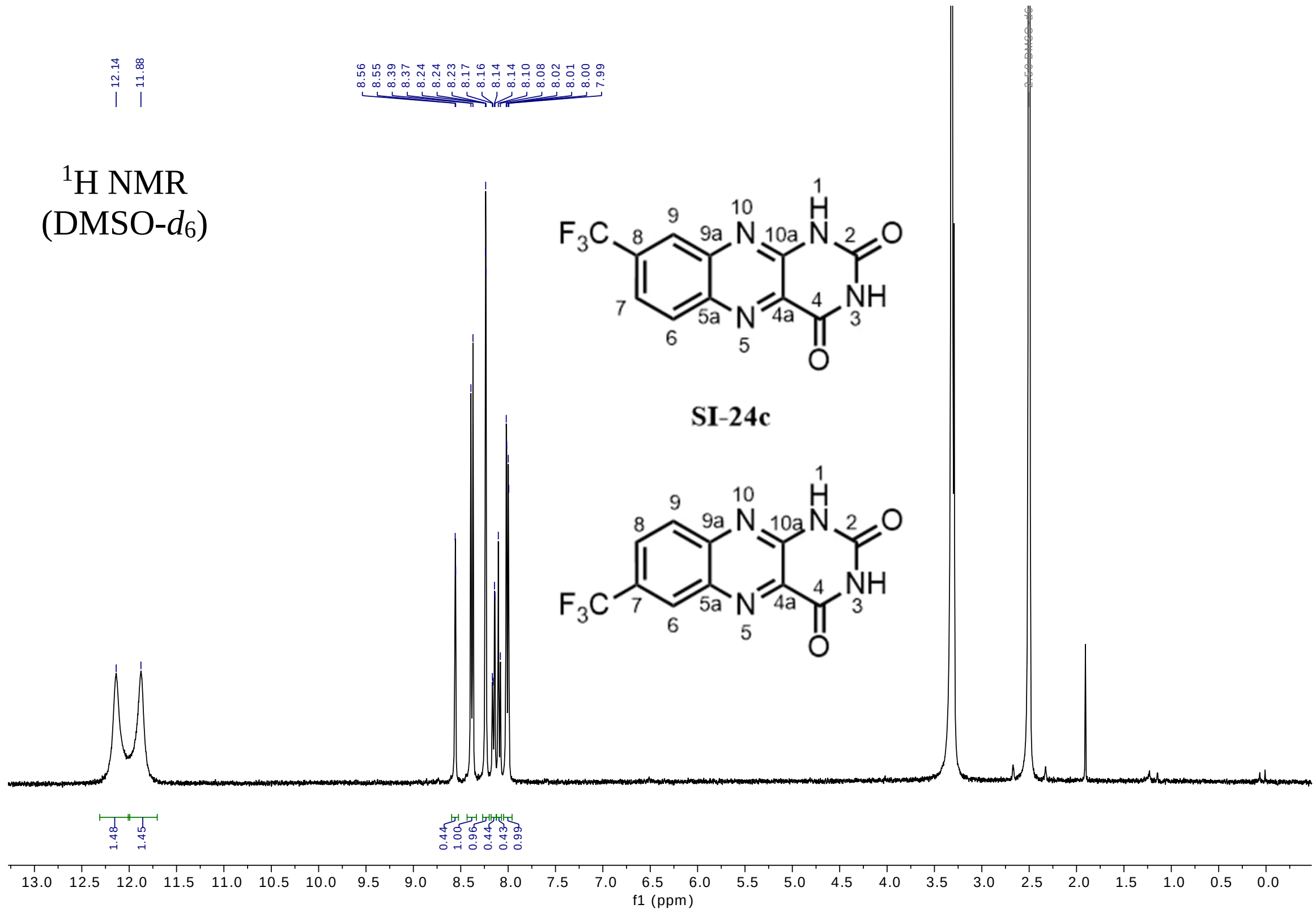
12.14
11.88
8.56
8.55
8.39
8.37
8.24
8.24
8.23
8.17
8.16
8.14
8.14
8.10
8.08
8.02
8.01
8.00
7.99
1H NMR
(DMSO-d6)
SI-24c
1.48
1.45
0.44
1.00
0.96
0.44
0.43
0.99
f1 (ppm)

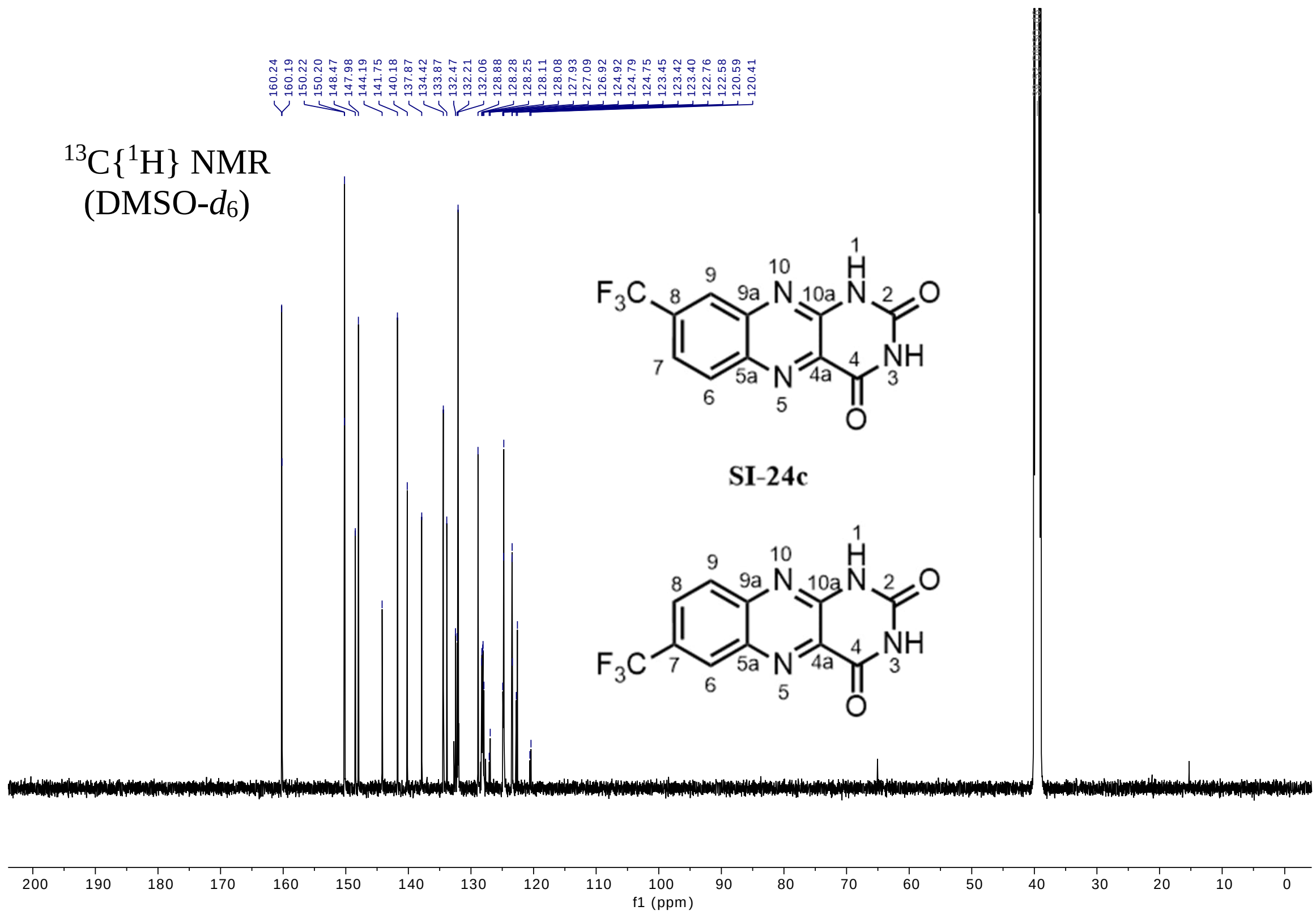
160.24
160.19
150.22
150.20
148.47
147.98
144.19
141.75
140.18
137.87
134.42
133.87
132.47
132.21
132.06
128.88
128.28
128.25
128.11
128.08
127.93
127.09
126.92
124.92
124.79
124.75
123.45
123.42
123.40
122.76
122.58
120.59
120.41
13C{1H} NMR
(DMSO-d6)
SI-24c
f1 (ppm)

^{19}F NMR
(DMSO-d_6)

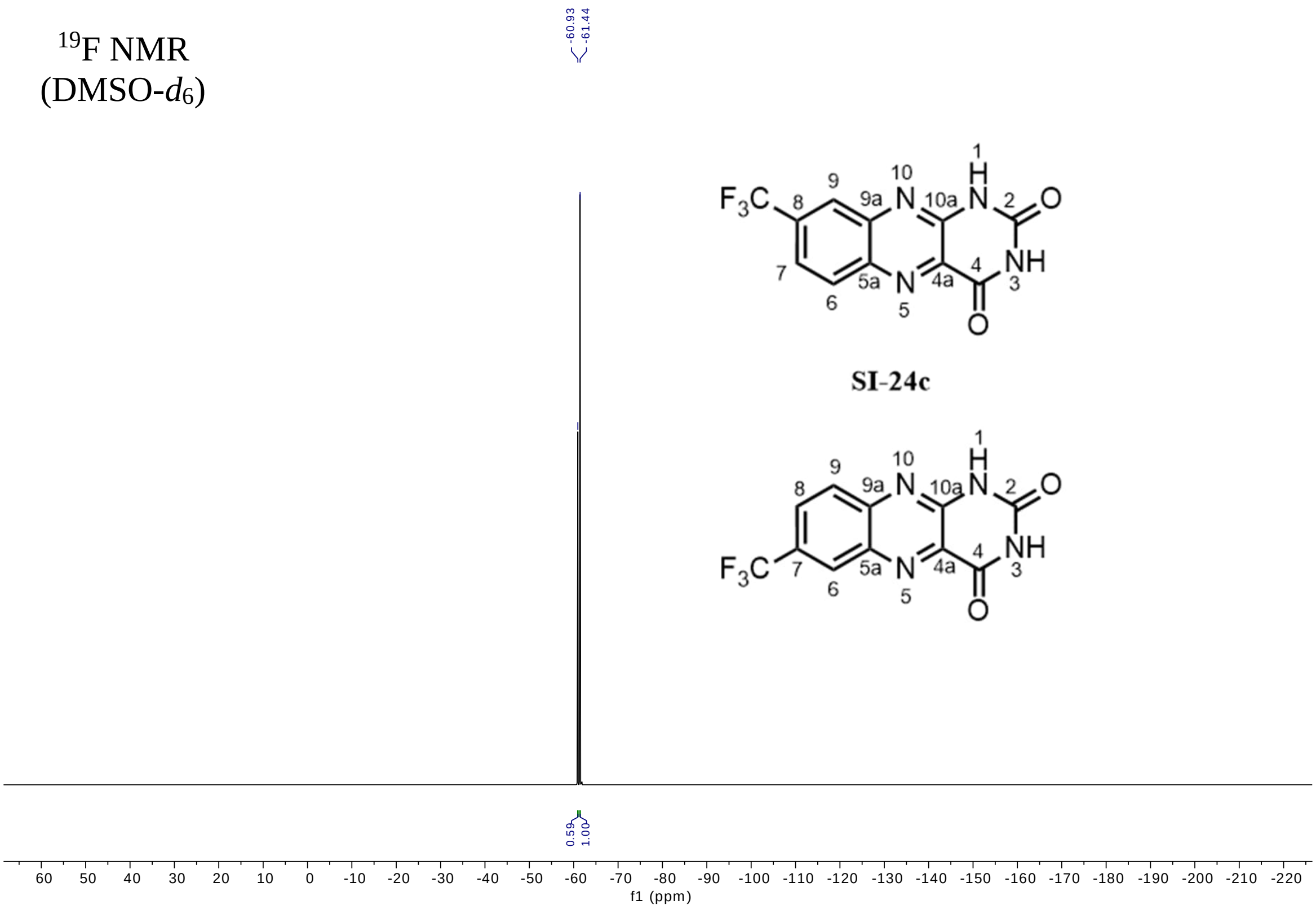

SI-24c

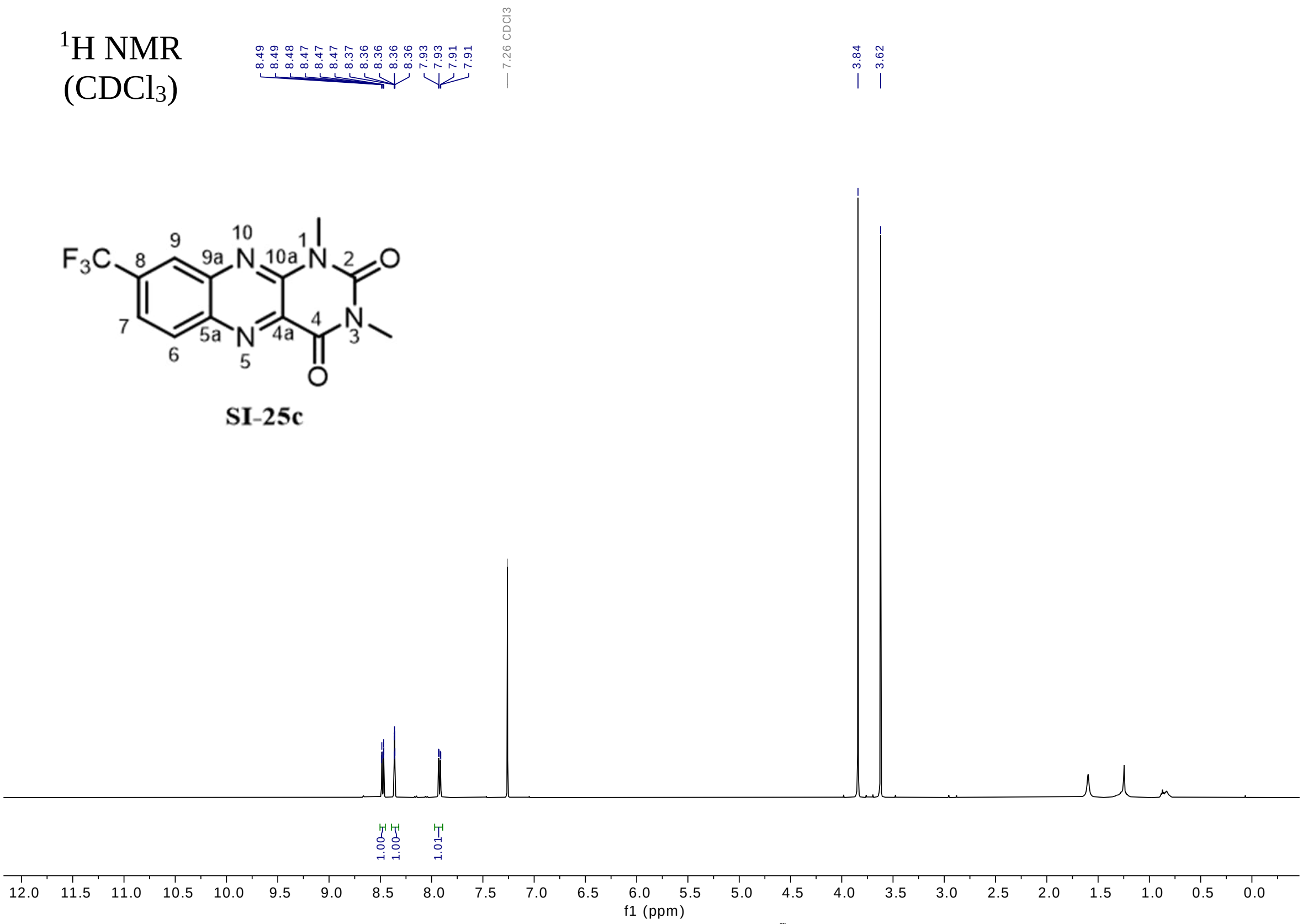

1H NMR
(CDCl3)
8.49
8.49
8.48
8.47
8.47
8.47
8.37
8.36
8.36
8.36
8.36
7.93
7.93
7.91
7.91
7.26 CDCl3
3.84
3.62
F3C
8
9
9a
10
10a
1
2
3
4
4a
5
5a
6
7
SI-25c
1.00
1.00
1.01
12.0
11.5
11.0
10.5
10.0
9.5
9.0
8.5
8.0
7.5
7.0
6.5
6.0
5.5
5.0
4.5
4.0
3.5
3.0
2.5
2.0
1.5
1.0
0.5
0.0
f1 (ppm)

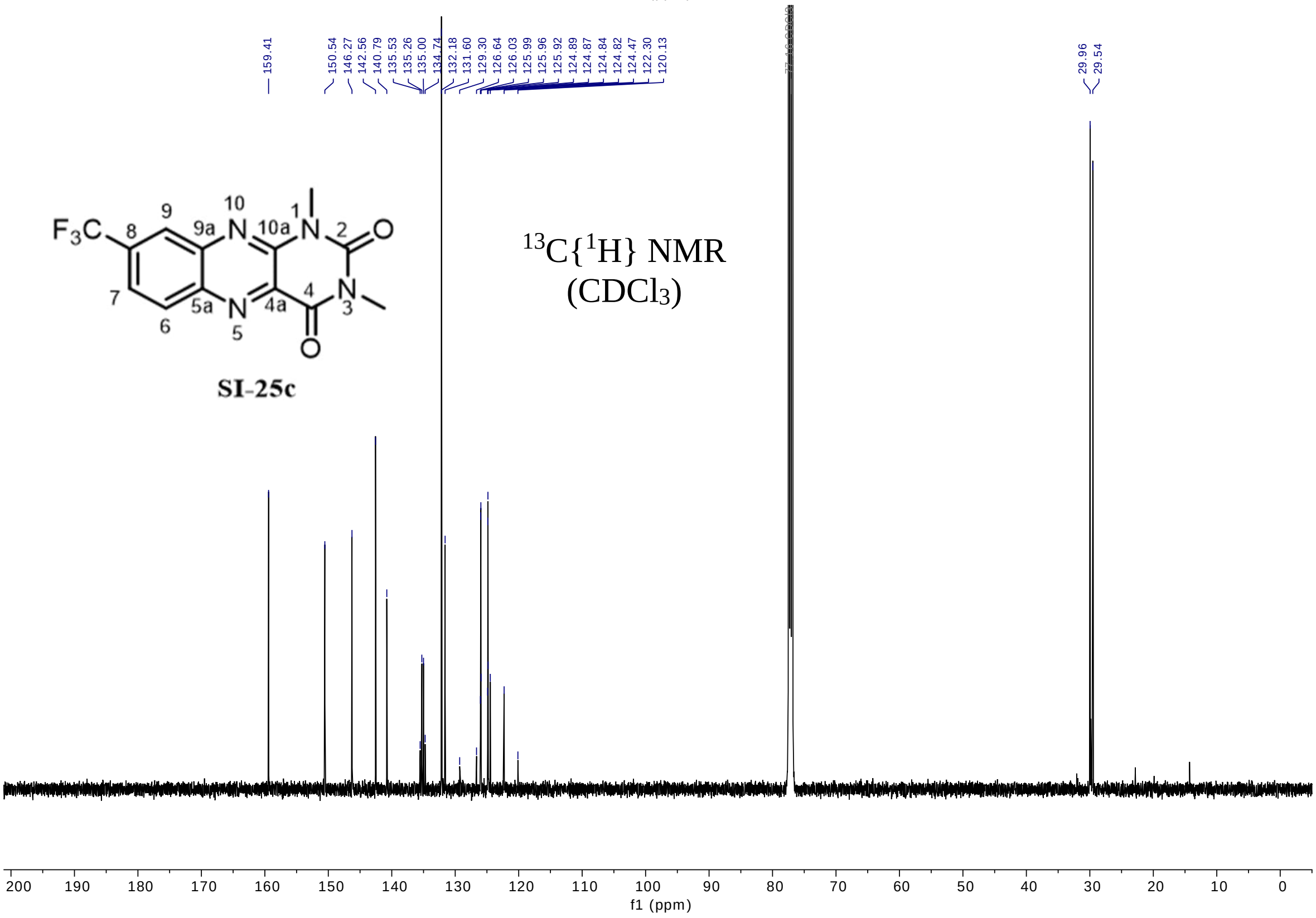

159.41
150.54
146.27
142.56
140.79
135.53
135.26
135.00
134.74
132.18
131.60
129.30
126.64
126.03
125.99
125.96
125.92
124.89
124.87
124.84
124.82
124.47
122.30
120.13
29.96
29.54
F3C
8
9
9a
10
10a
1
2
3
4
4a
5
5a
6
7
SI-25c
13C{1H} NMR
(CDCl3)
200
190
180
170
160
150
140
130
120
110
100
90
80
70
60
50
40
30
20
10
0
f1 (ppm)

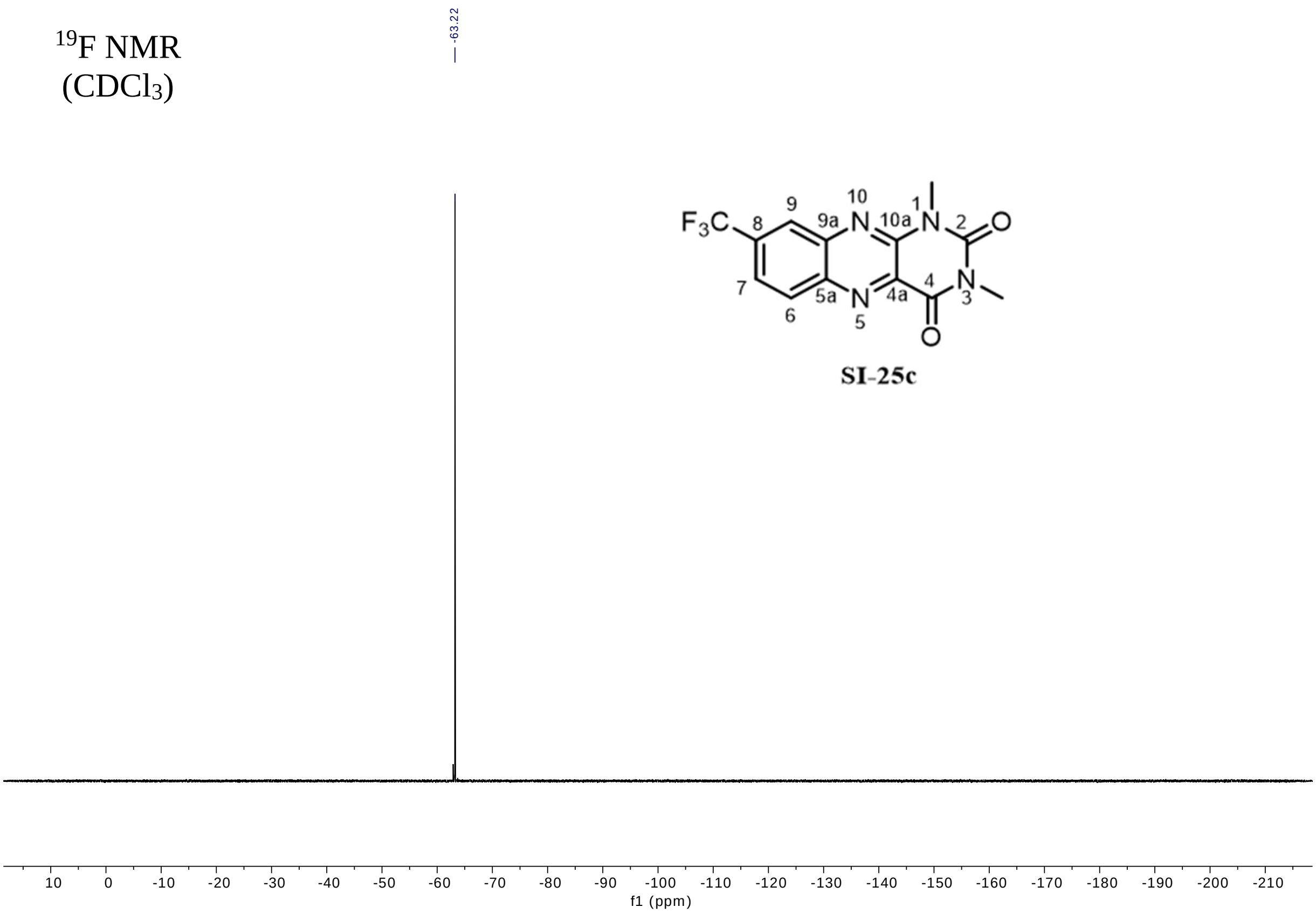
^{19}F NMR
($CDCl_3$)
-63.22
SI-25c
10 0 -10 -20 -30 -40 -50 -60 -70 -80 -90 -100 -110 -120 -130 -140 -150 -160 -170 -180 -190 -200 -210
f1 (ppm)

^{1}H NMR
(CD_3CN)

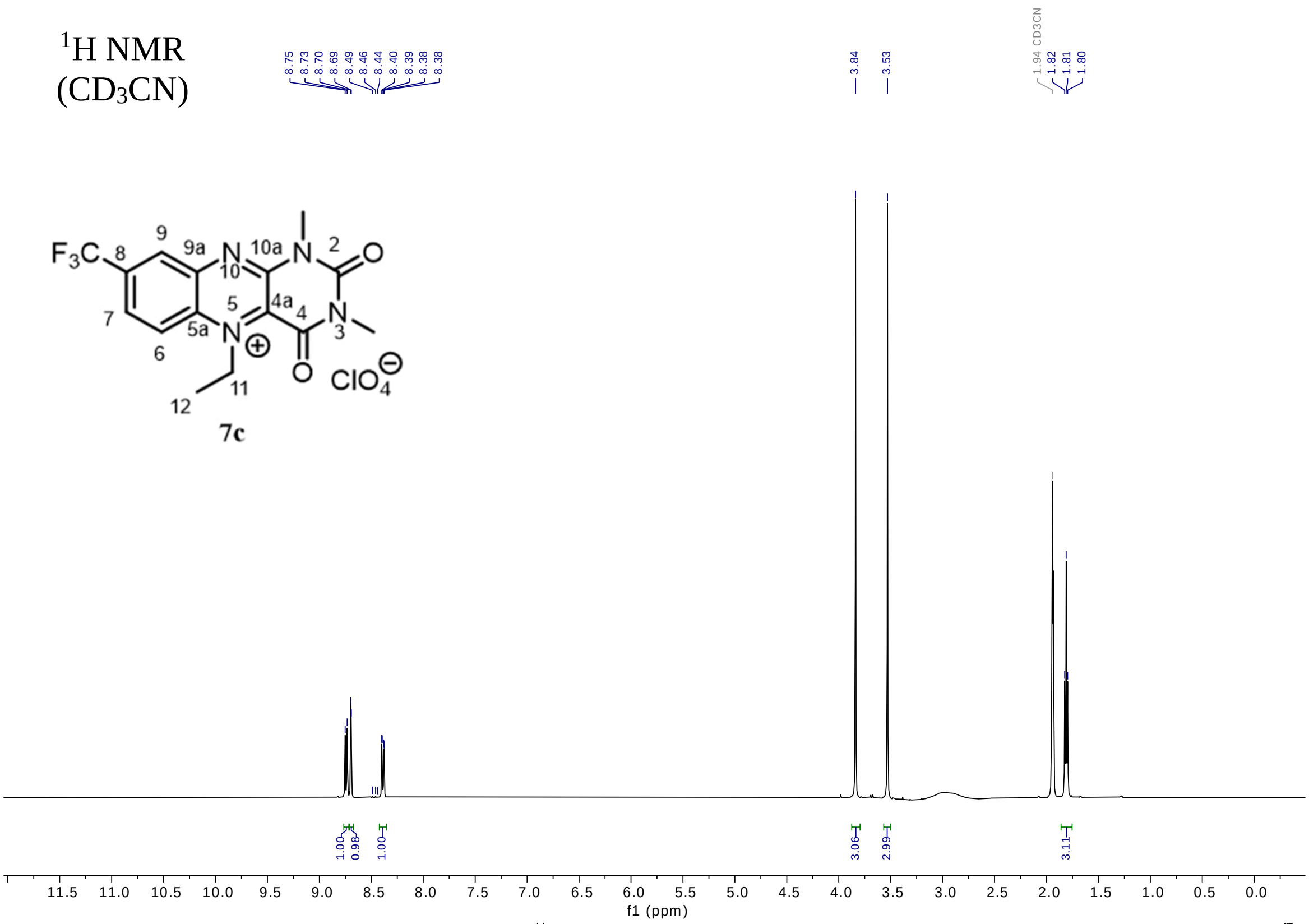

$^{13}C\{^{1}H\}$ NMR
(CD_3CN)

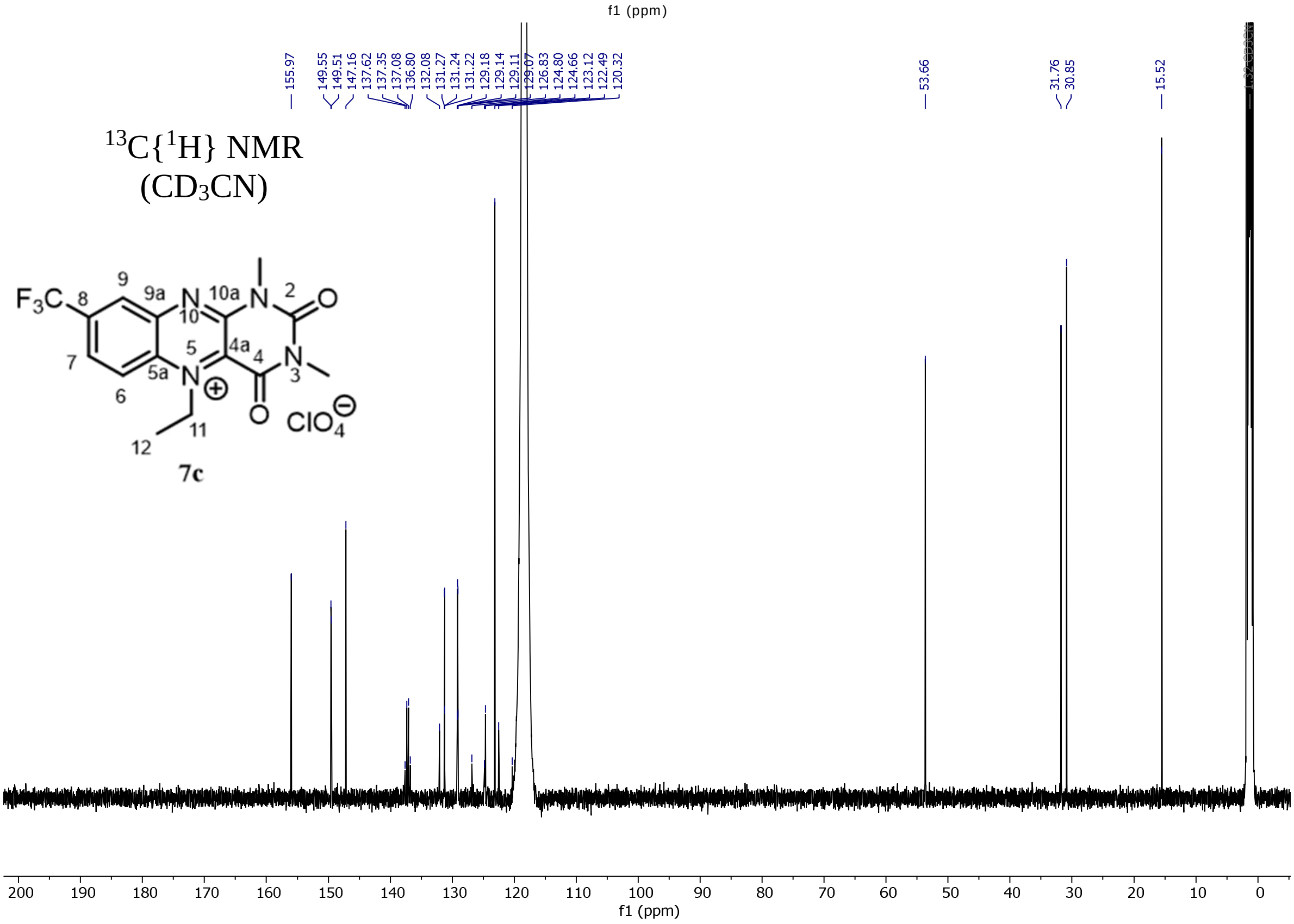

^{19}F NMR
(CD_3CN)

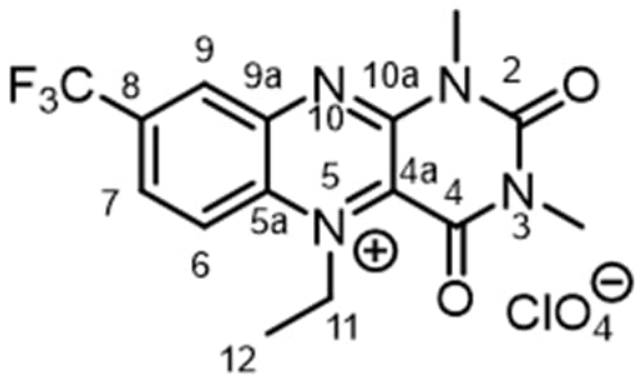

7c

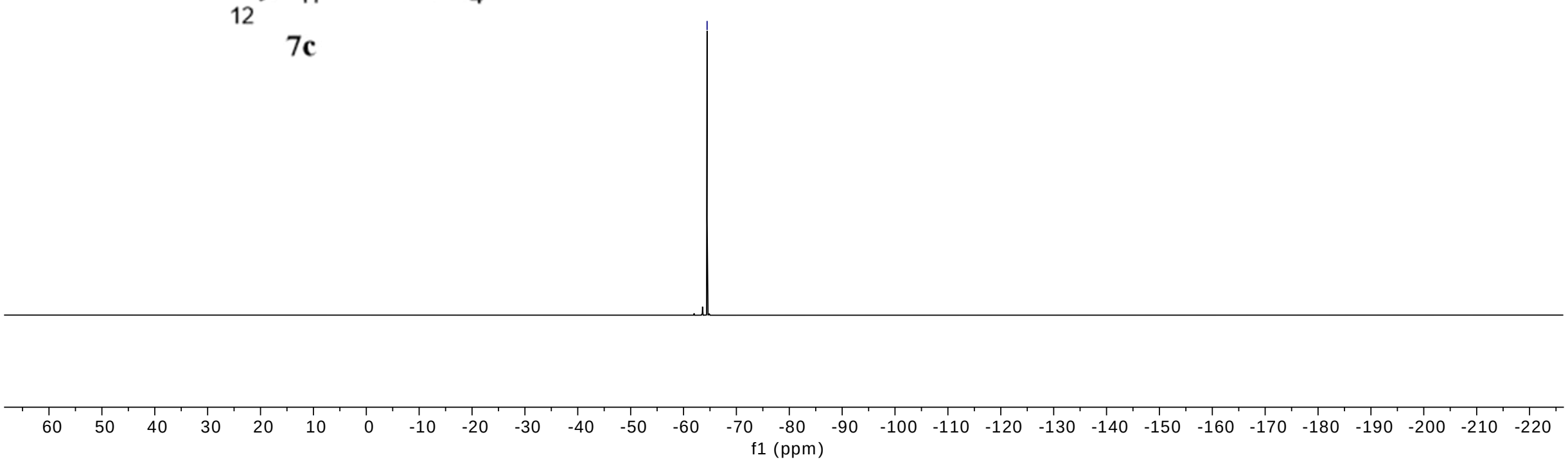

^{1}H NMR
(DMSO-d_6)

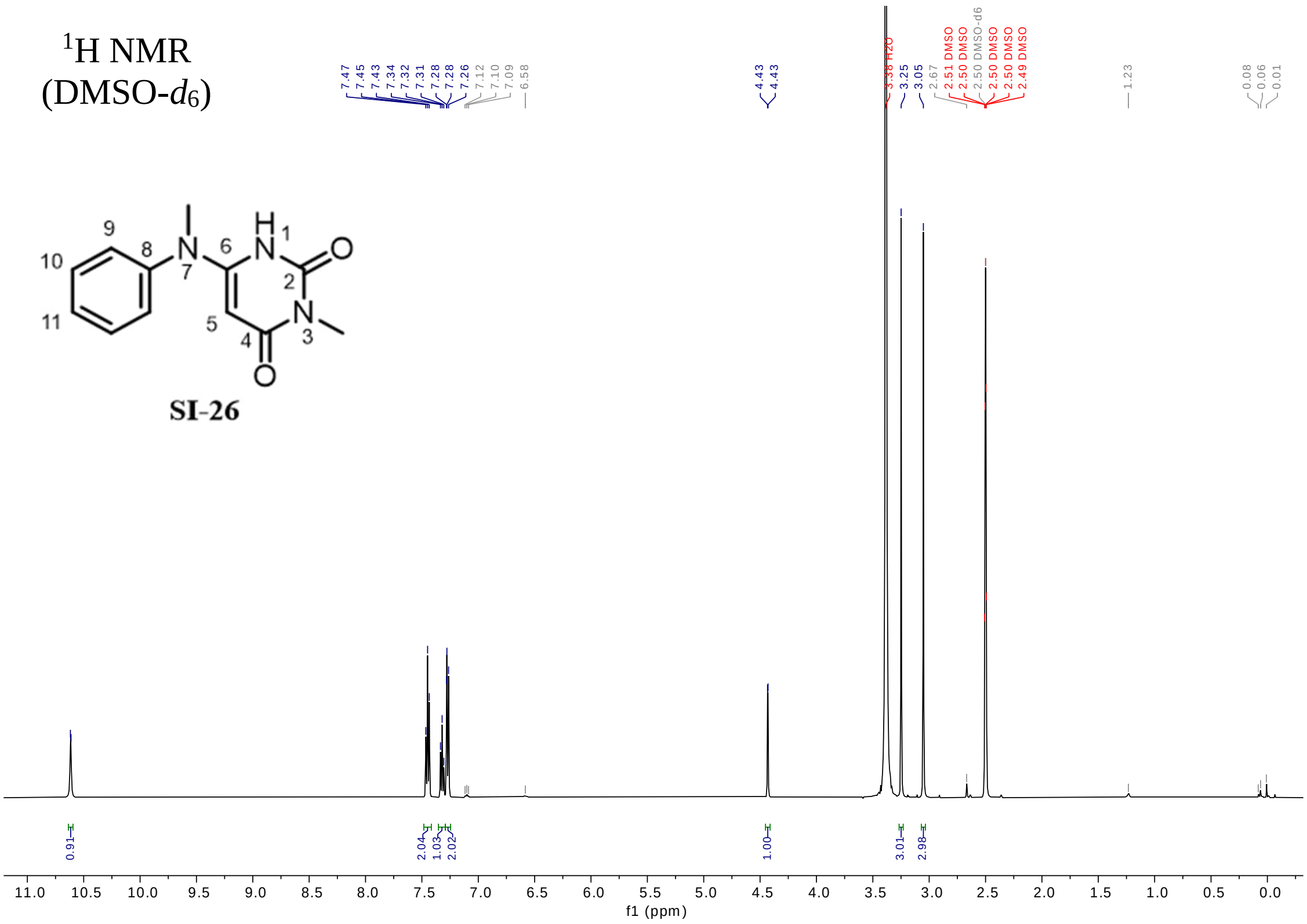

SI-26

^{13}C{^{1}H} NMR
(DMSO-d_6)

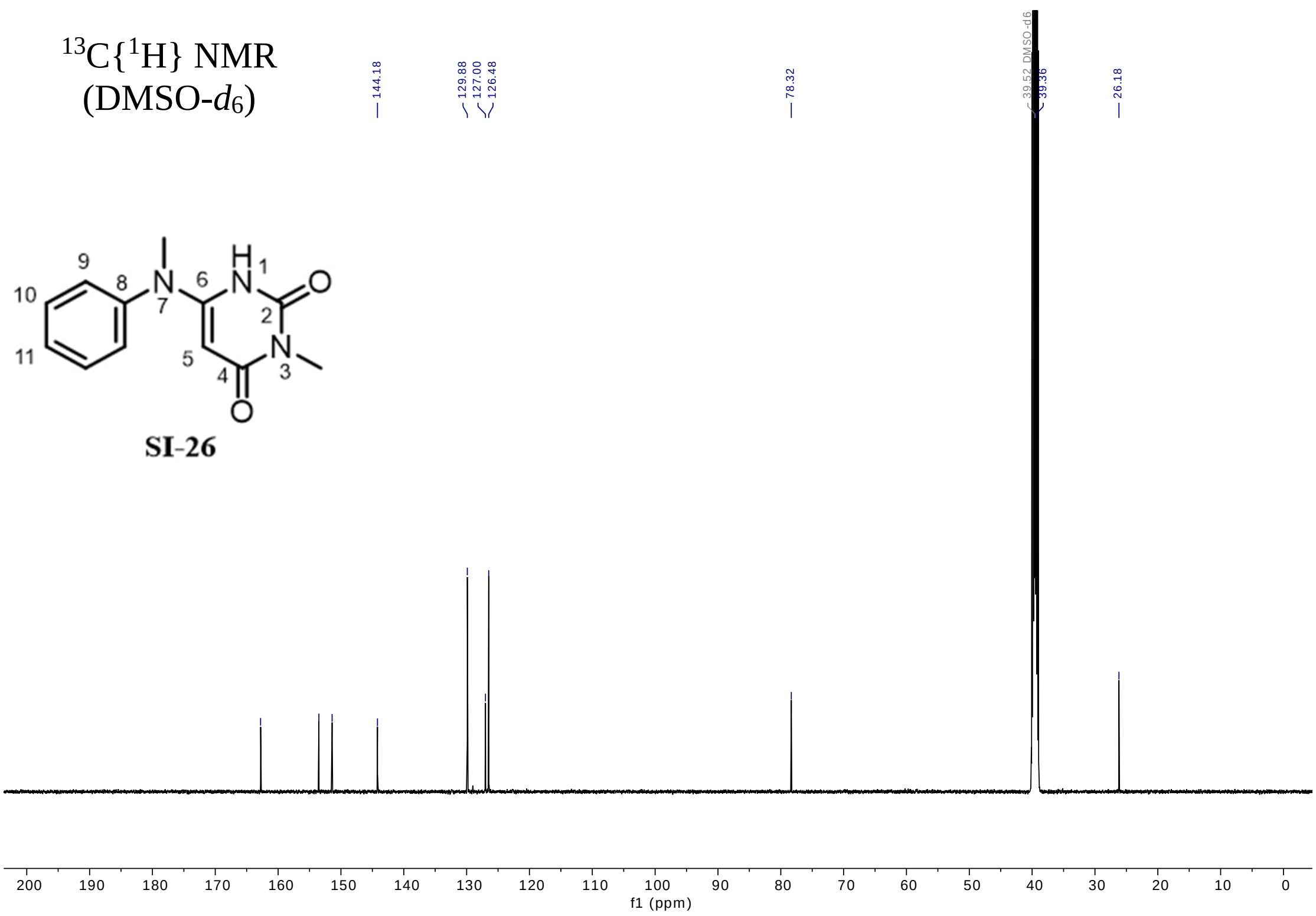

SI-26

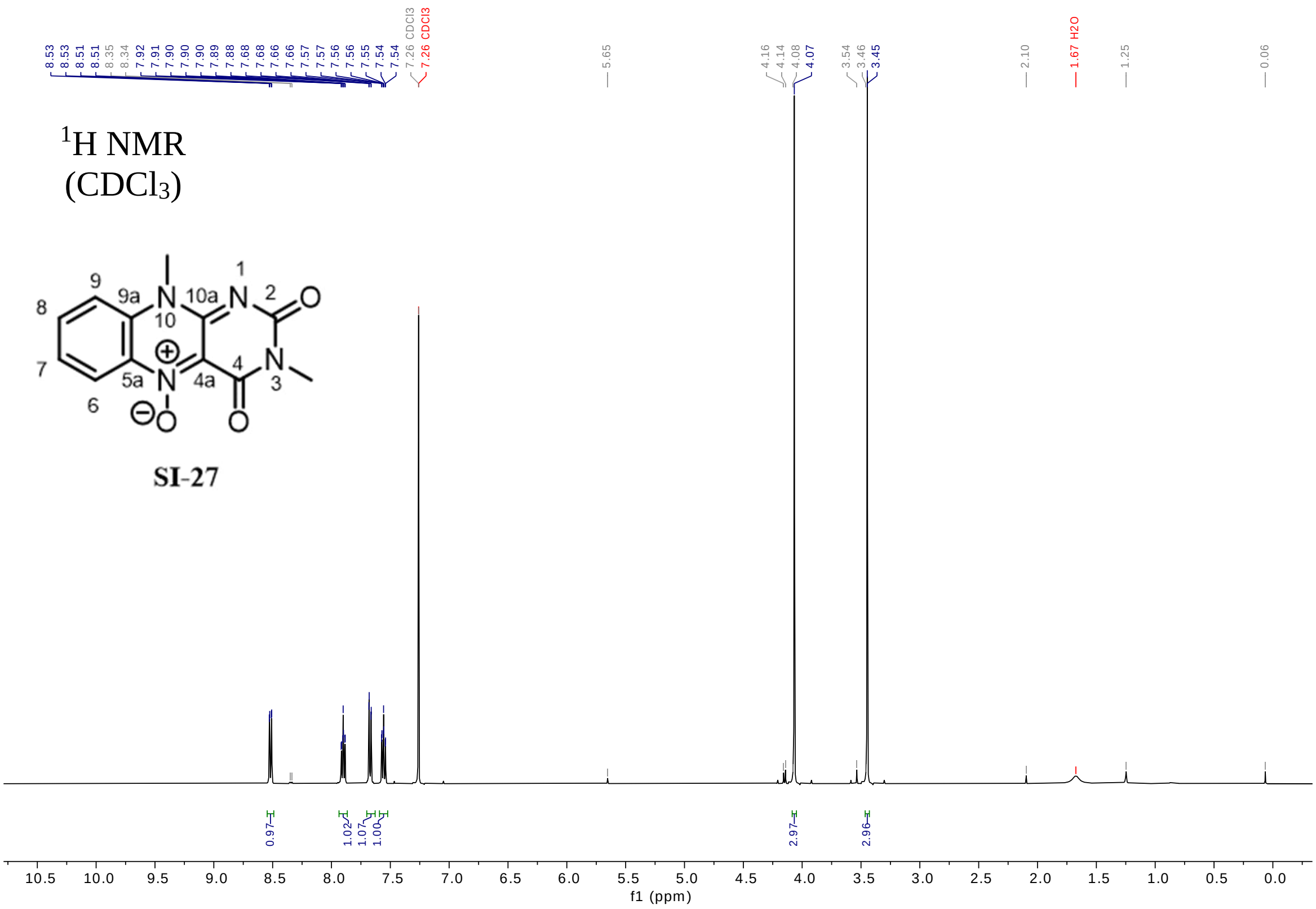

^{1}H NMR
($CDCl_3$)
SI-27
8.53 8.53 8.51 8.51 8.35 8.34 7.92 7.91 7.90 7.90 7.90 7.89 7.88 7.68 7.68 7.66 7.66 7.57 7.57 7.56 7.56 7.55 7.54 7.54
7.26 CDCl3
7.26 CDCl3
5.65
4.16 4.14 4.08 4.07
3.54 3.46 3.45
2.10
1.67 H2O
1.25
0.06
0.97 1.02 1.07 1.00 2.97 2.96
f1 (ppm)

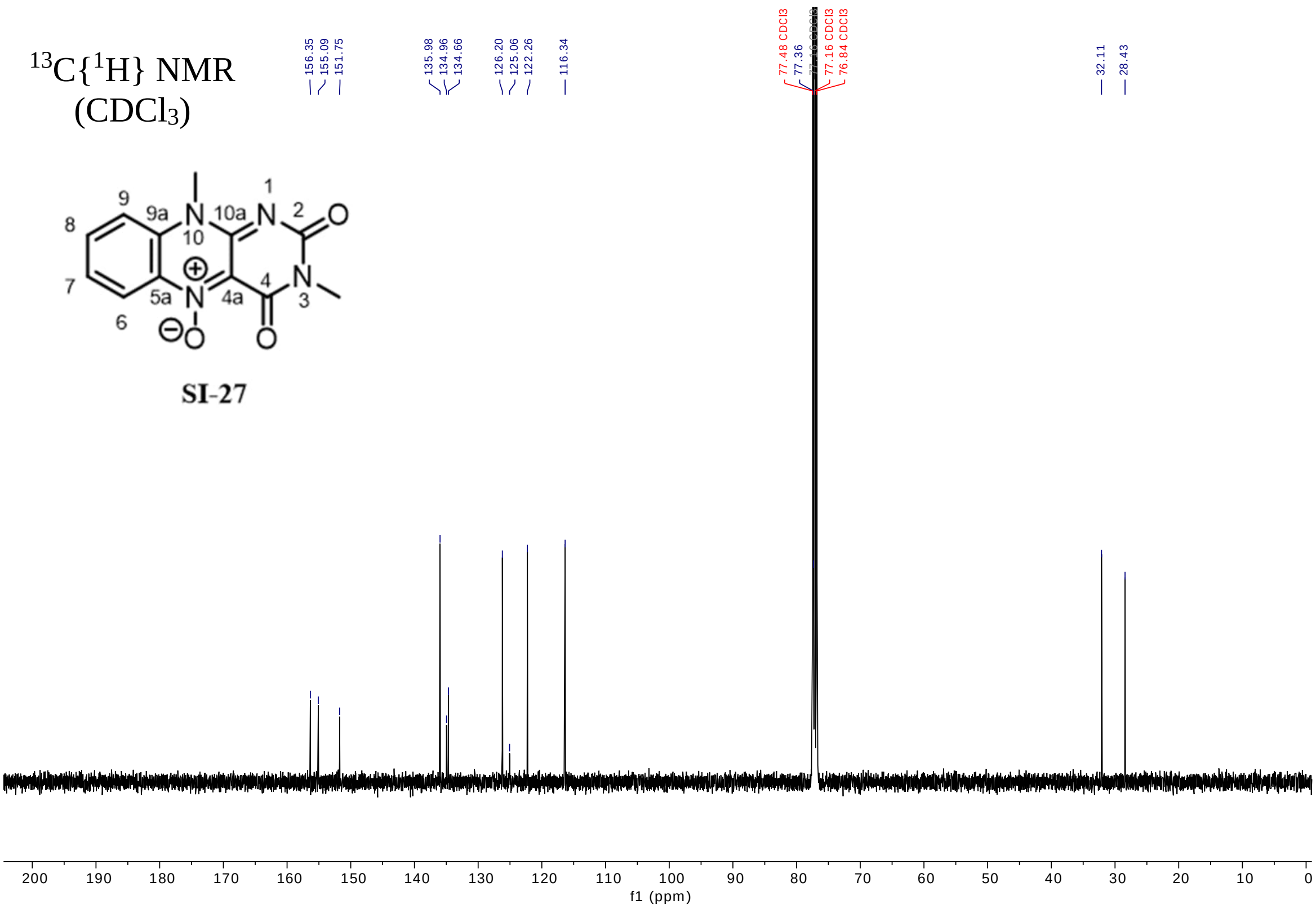

$^{13}C\{^{1}H\}$ NMR
($CDCl_3$)
SI-27
156.35 155.09 151.75
135.98 134.96 134.66
126.20 125.06 122.26
116.34
77.48 CDCl3
77.36
77.16 CDCl3
76.84 CDCl3
32.11
28.43
f1 (ppm)

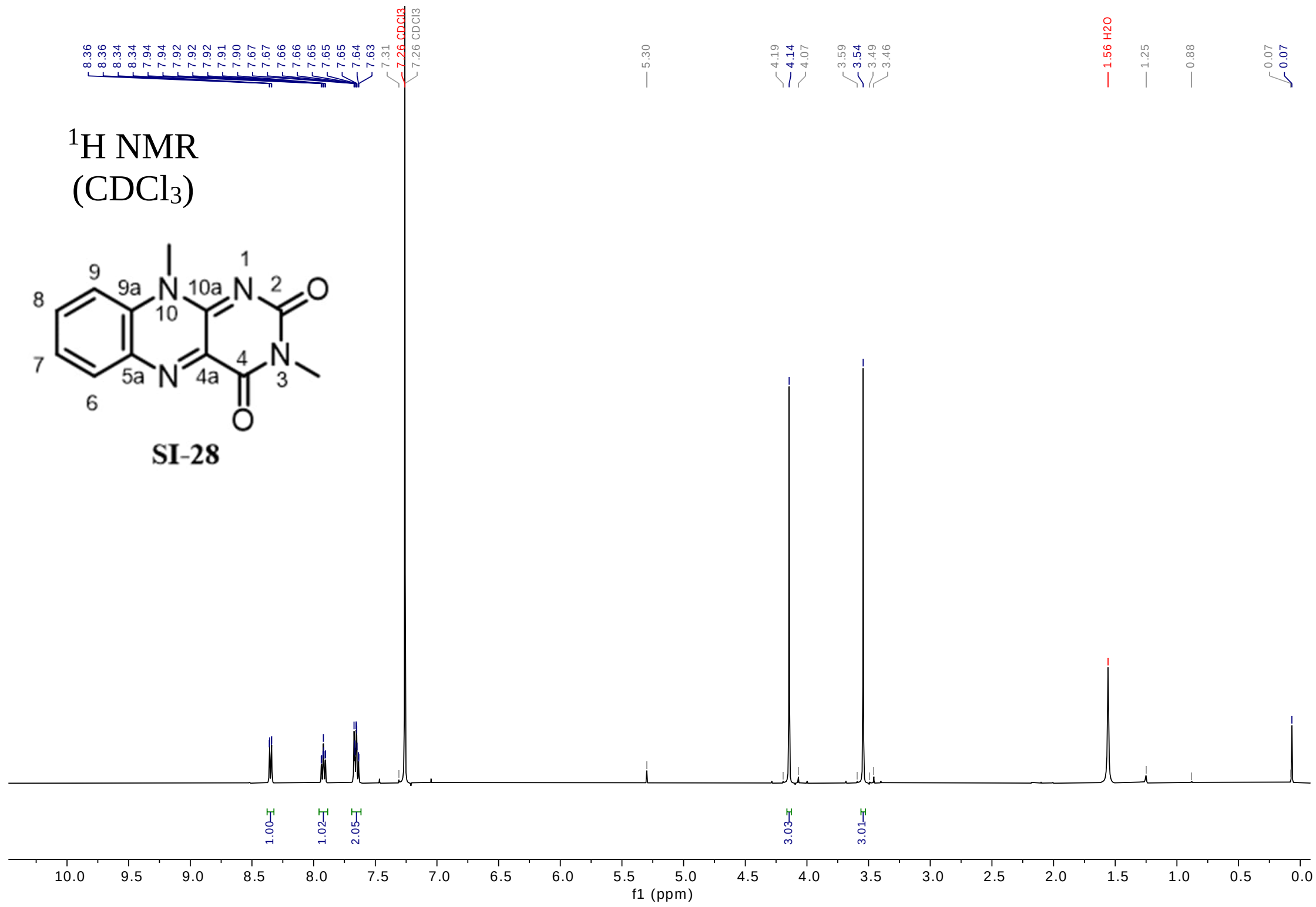
1H NMR
(CDCl3)
SI-28
8.36 8.36 8.34 8.34 7.94 7.94 7.92 7.92 7.92 7.91 7.90 7.67 7.67 7.66 7.66 7.65 7.65 7.65 7.64 7.63
7.31
7.26 CDCl3
7.26 CDCl3
5.30
4.19 4.14 4.07
3.59 3.54 3.49 3.46
1.56 H2O
1.25
0.88
0.07 0.07
1.00
1.02
2.05
3.03
3.01
f1 (ppm)

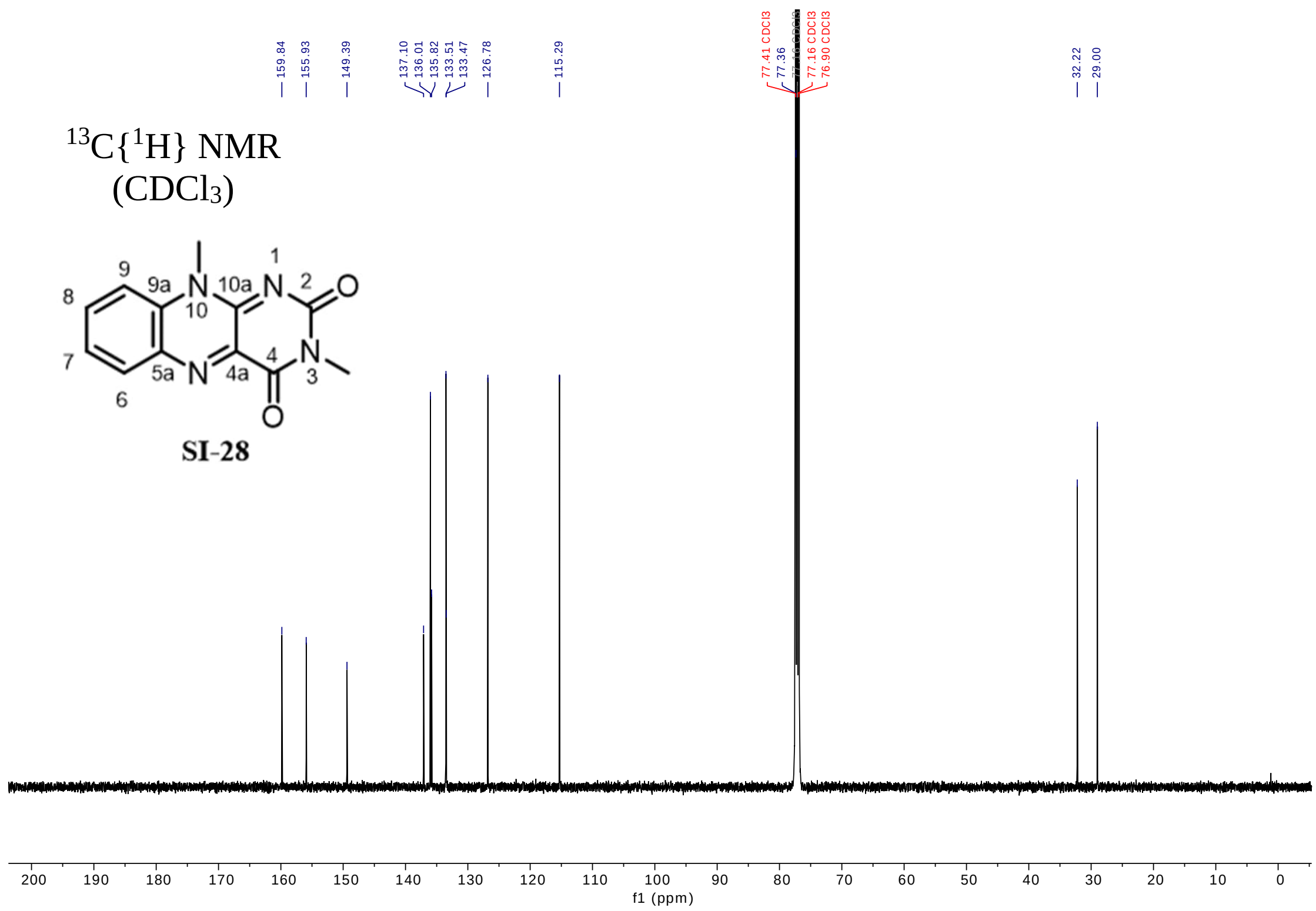
13C{1H} NMR
(CDCl3)
SI-28
159.84
155.93
149.39
137.10 136.01 135.82 133.51 133.47
126.78
115.29
77.41 CDCl3
77.36
77.16 CDCl3
76.90 CDCl3
32.22
29.00
f1 (ppm)

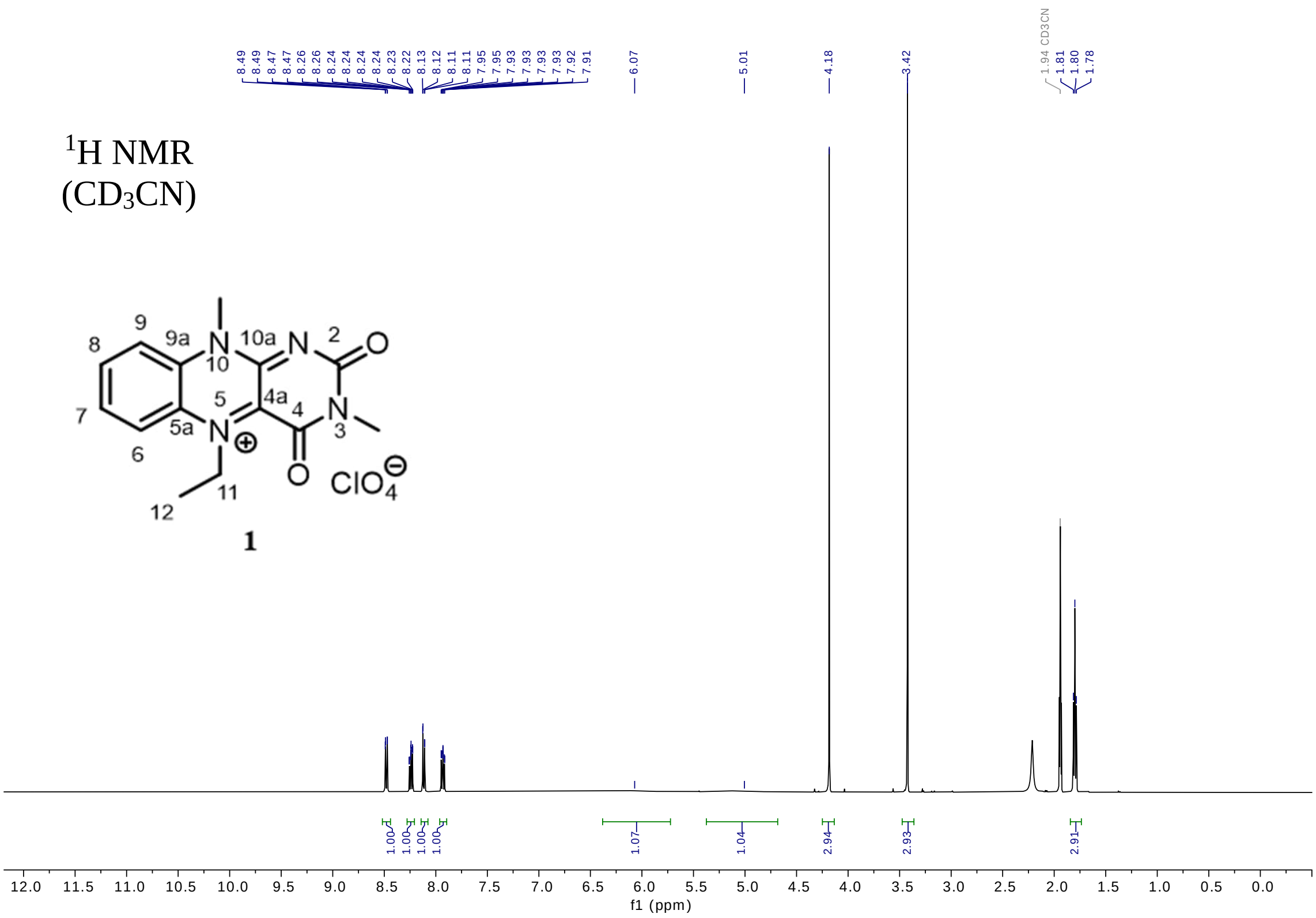

1H NMR
(CD3CN)
8.49 8.49 8.47 8.47 8.26 8.26 8.24 8.24 8.24 8.24 8.24 8.23 8.22 8.13 8.12 8.11 8.11 7.95 7.95 7.93 7.93 7.93 7.93 7.92 7.91
6.07
5.01
4.18
3.42
1.94 CD3CN
1.81
1.80
1.78
1.00
1.00
1.00
1.00
1.07
1.04
2.94
2.93
2.91
9
8
9a
10a
2
10
5
4a
4
7
5a
3
6
11
12
ClO4
1
12.0 11.5 11.0 10.5 10.0 9.5 9.0 8.5 8.0 7.5 7.0 6.5 6.0 5.5 5.0 4.5 4.0 3.5 3.0 2.5 2.0 1.5 1.0 0.5 0.0
f1 (ppm)

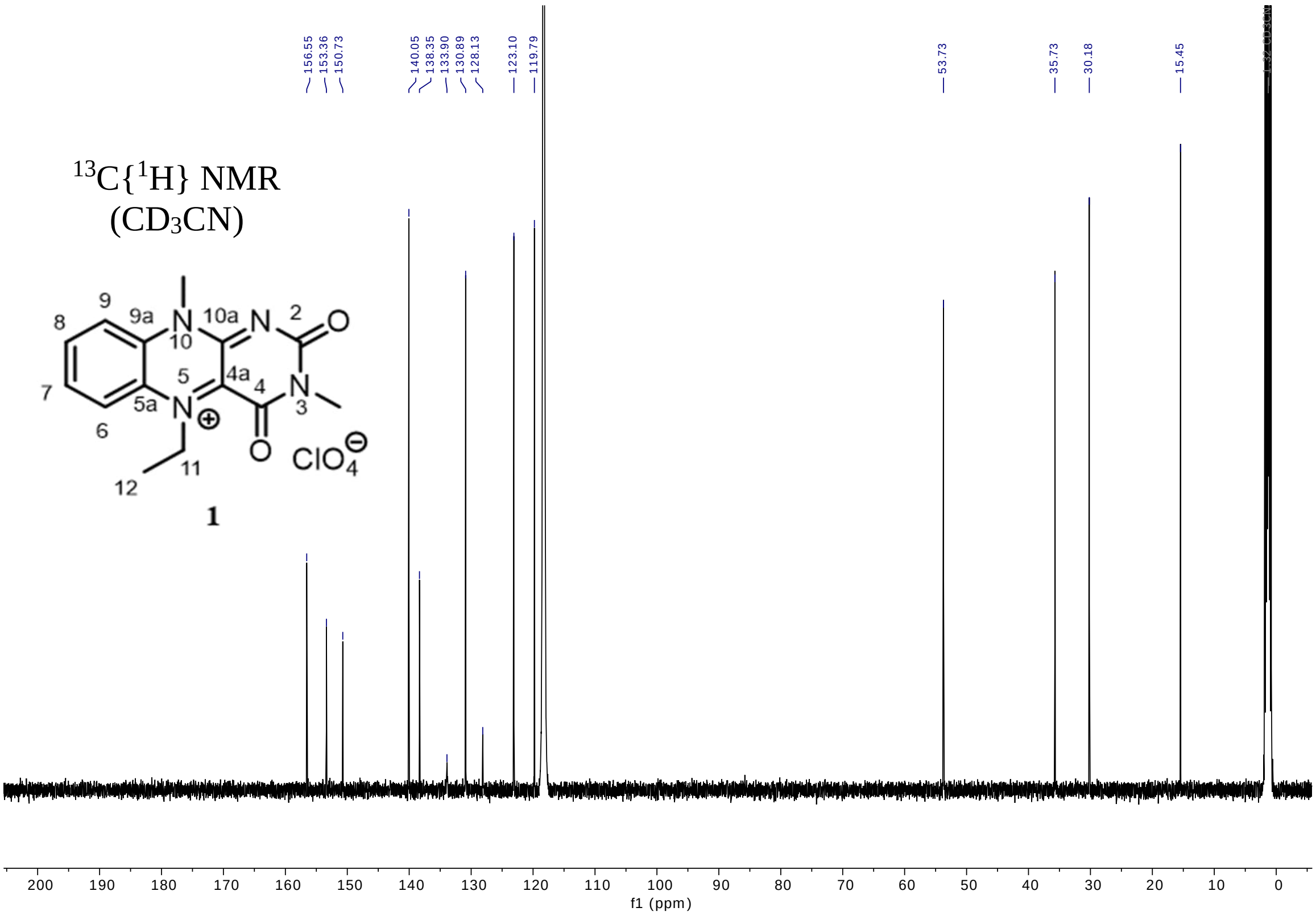

13C{1H} NMR
(CD3CN)
156.55
153.36
150.73
140.05
138.35
133.90
130.89
128.13
123.10
119.79
53.73
35.73
30.18
15.45
9
8
9a
10a
2
10
5
4a
4
7
5a
3
6
11
12
ClO4
1
200 190 180 170 160 150 140 130 120 110 100 90 80 70 60 50 40 30 20 10 0
f1 (ppm)

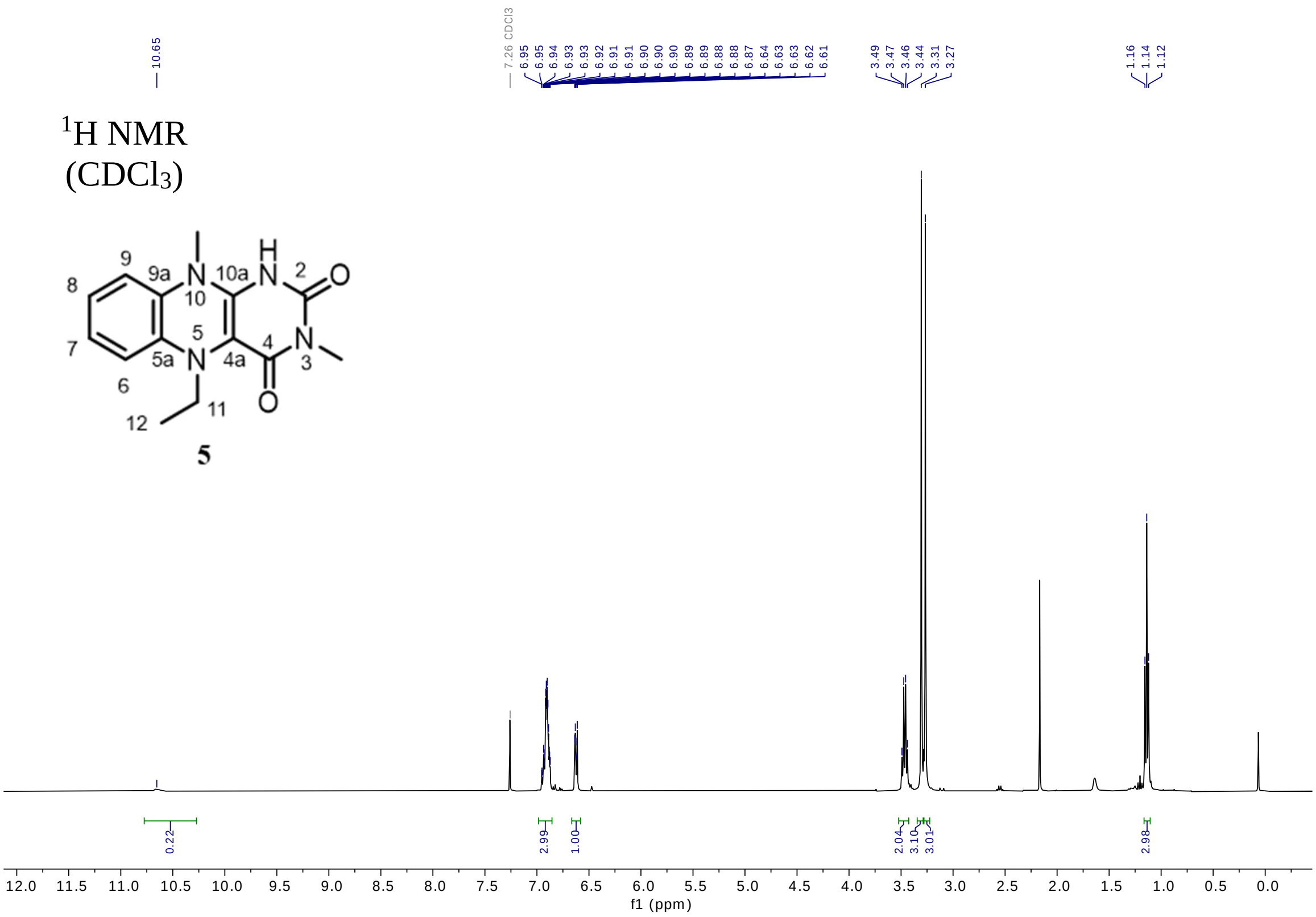

1H NMR
(CDCl3)
5
10.65
7.26 CDCl3
6.95
6.95
6.94
6.93
6.93
6.92
6.91
6.91
6.90
6.90
6.90
6.89
6.89
6.88
6.87
6.64
6.63
6.63
6.62
6.61
3.49
3.47
3.46
3.44
3.31
3.27
1.16
1.14
1.12
0.22
2.99
1.00
2.04
3.10
3.01
2.98
f1 (ppm)

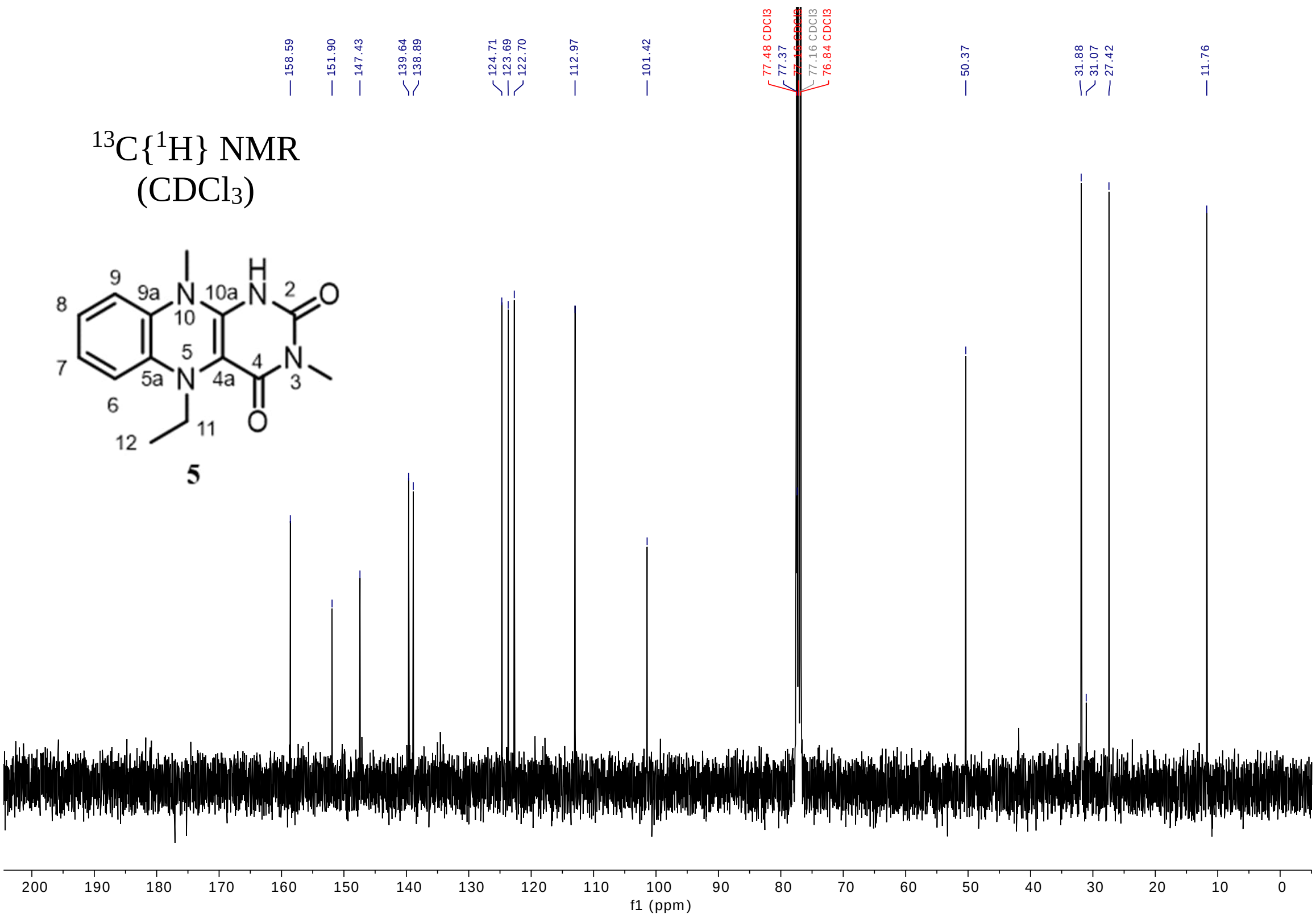

13C{1H} NMR
(CDCl3)
5
158.59
151.90
147.43
139.64
138.89
124.71
123.69
122.70
112.97
101.42
77.48 CDCl3
77.37
77.16 CDCl3
76.84 CDCl3
50.37
31.88
31.07
27.42
11.76
f1 (ppm)

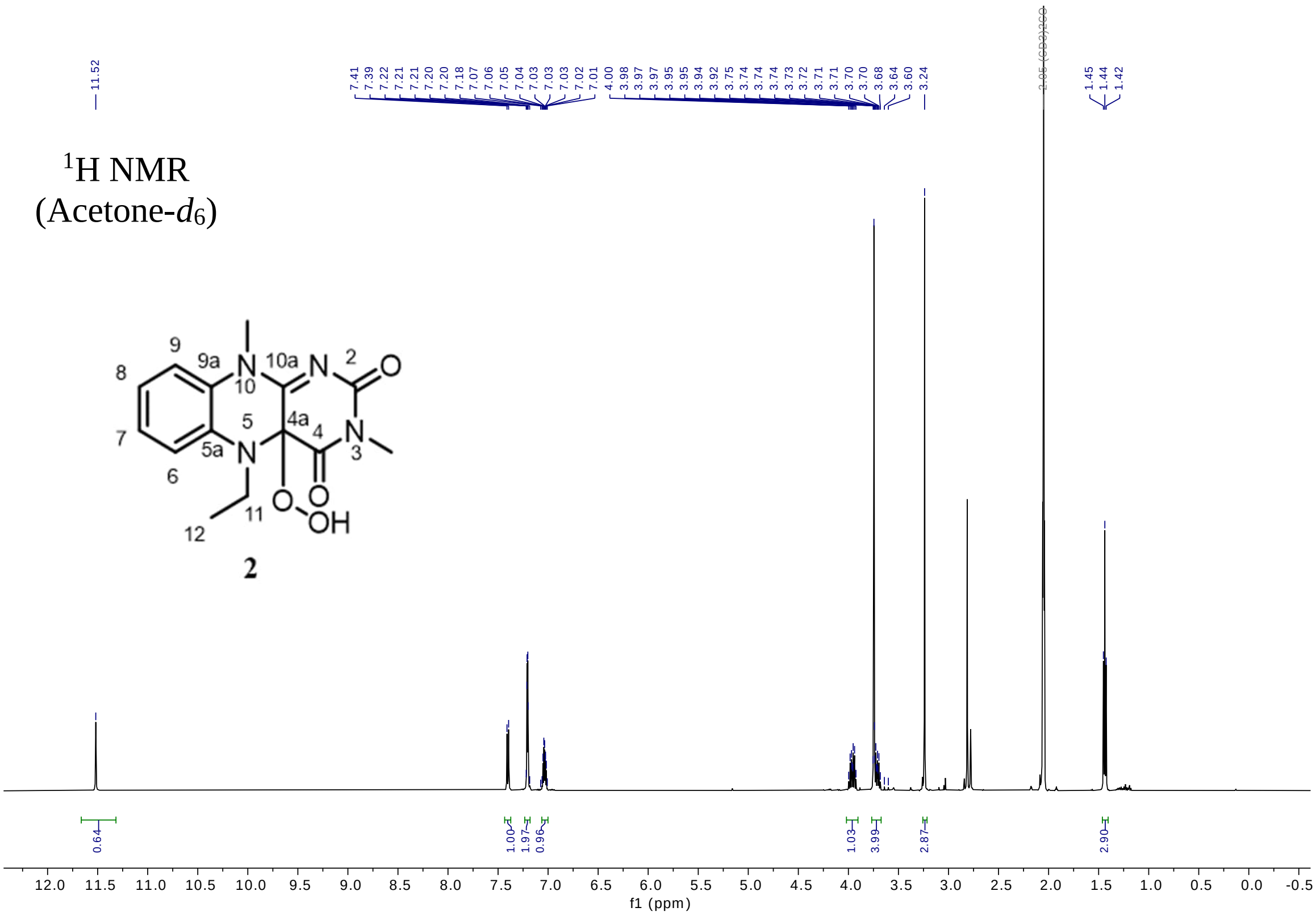
^{1}H NMR
(Acetone-d_6)
2
f1 (ppm)

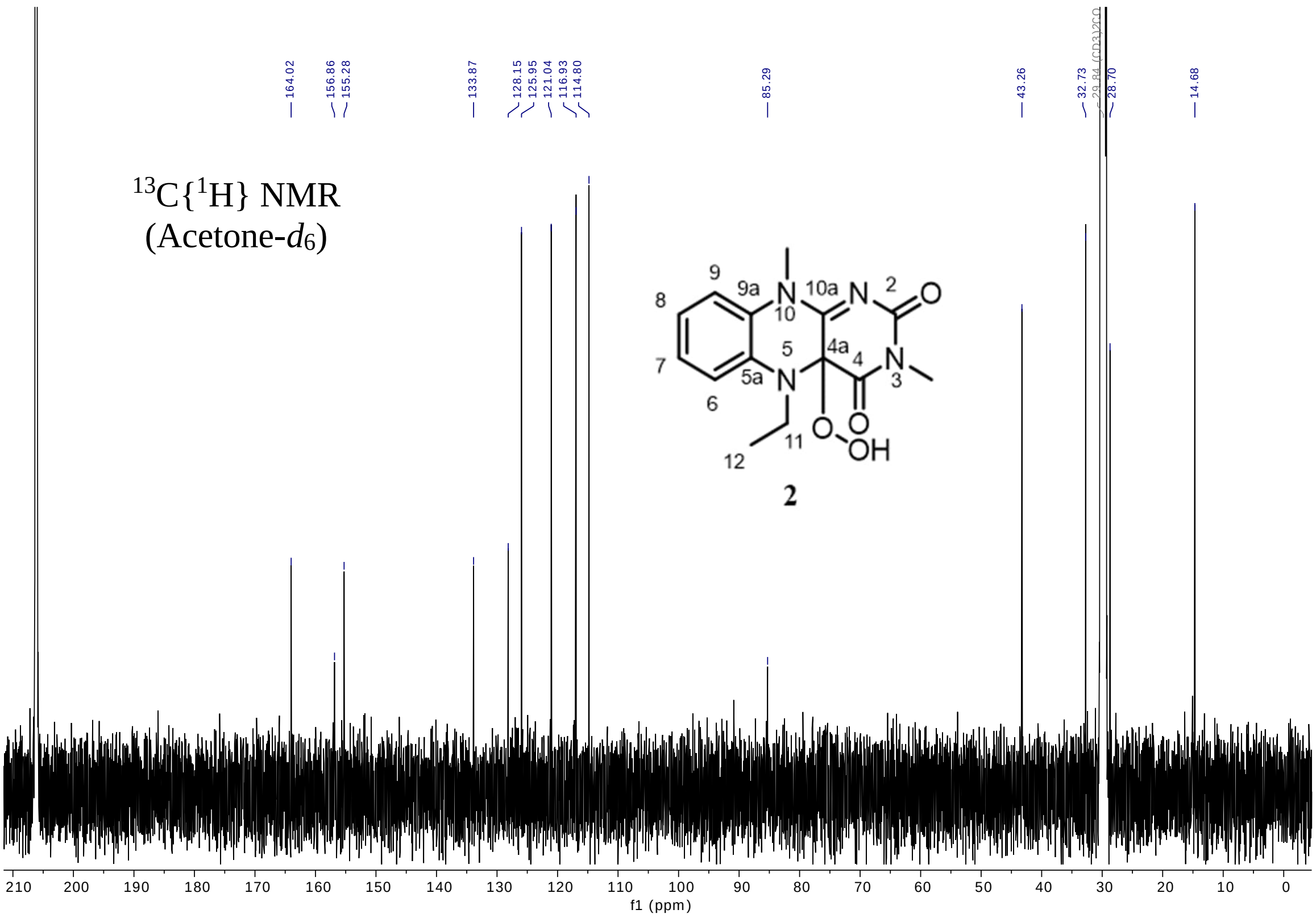
^{13}C{^{1}H} NMR
(Acetone-d_6)
2
f1 (ppm)

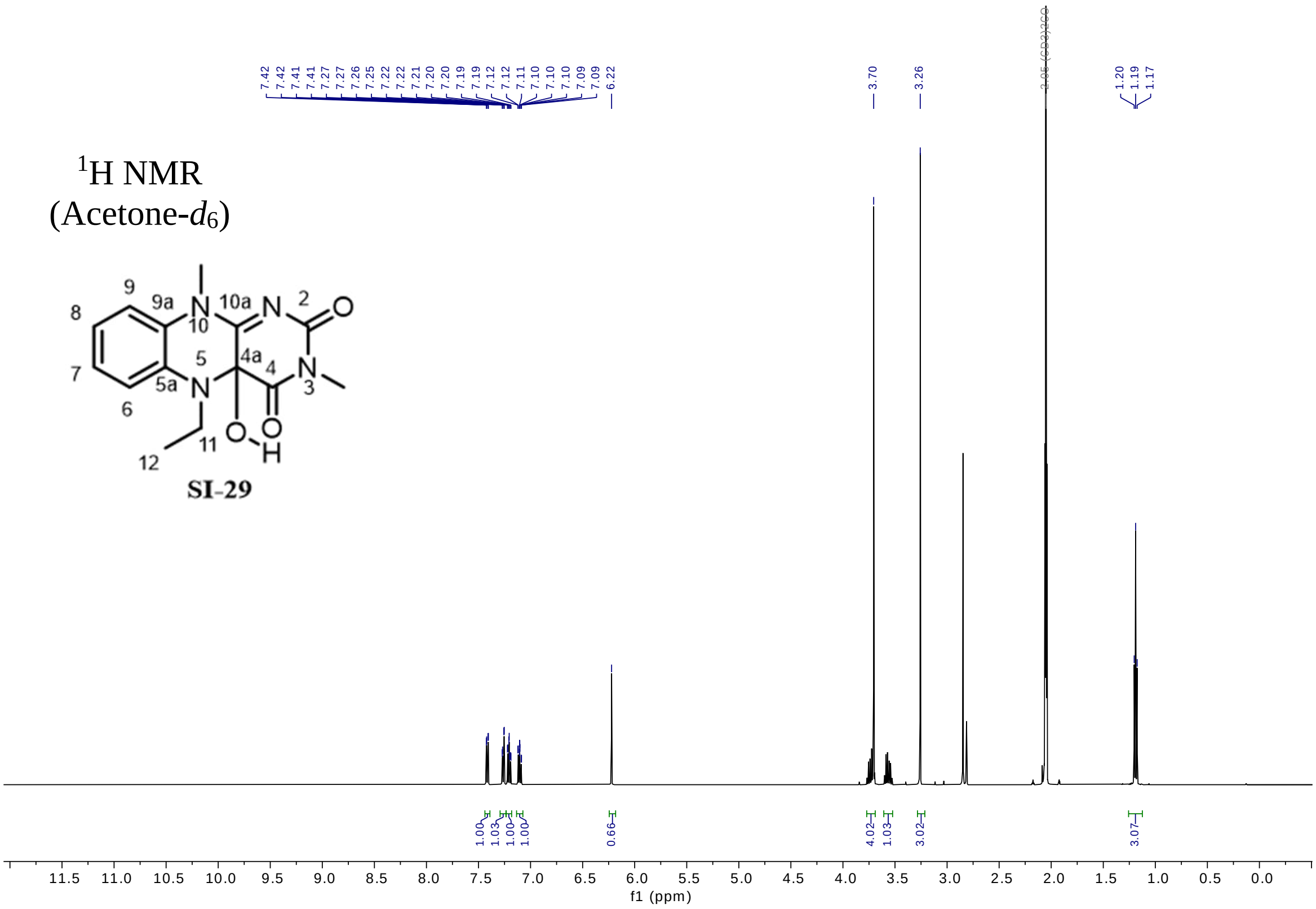
1H NMR
(Acetone-d6)
SI-29
7.42
7.42
7.41
7.41
7.27
7.27
7.26
7.25
7.22
7.22
7.21
7.20
7.20
7.19
7.19
7.12
7.12
7.11
7.10
7.10
7.10
7.09
6.22
3.70
3.26
1.20
1.19
1.17
1.00
1.03
1.00
1.00
0.66
4.02
1.03
3.02
3.07
f1 (ppm)

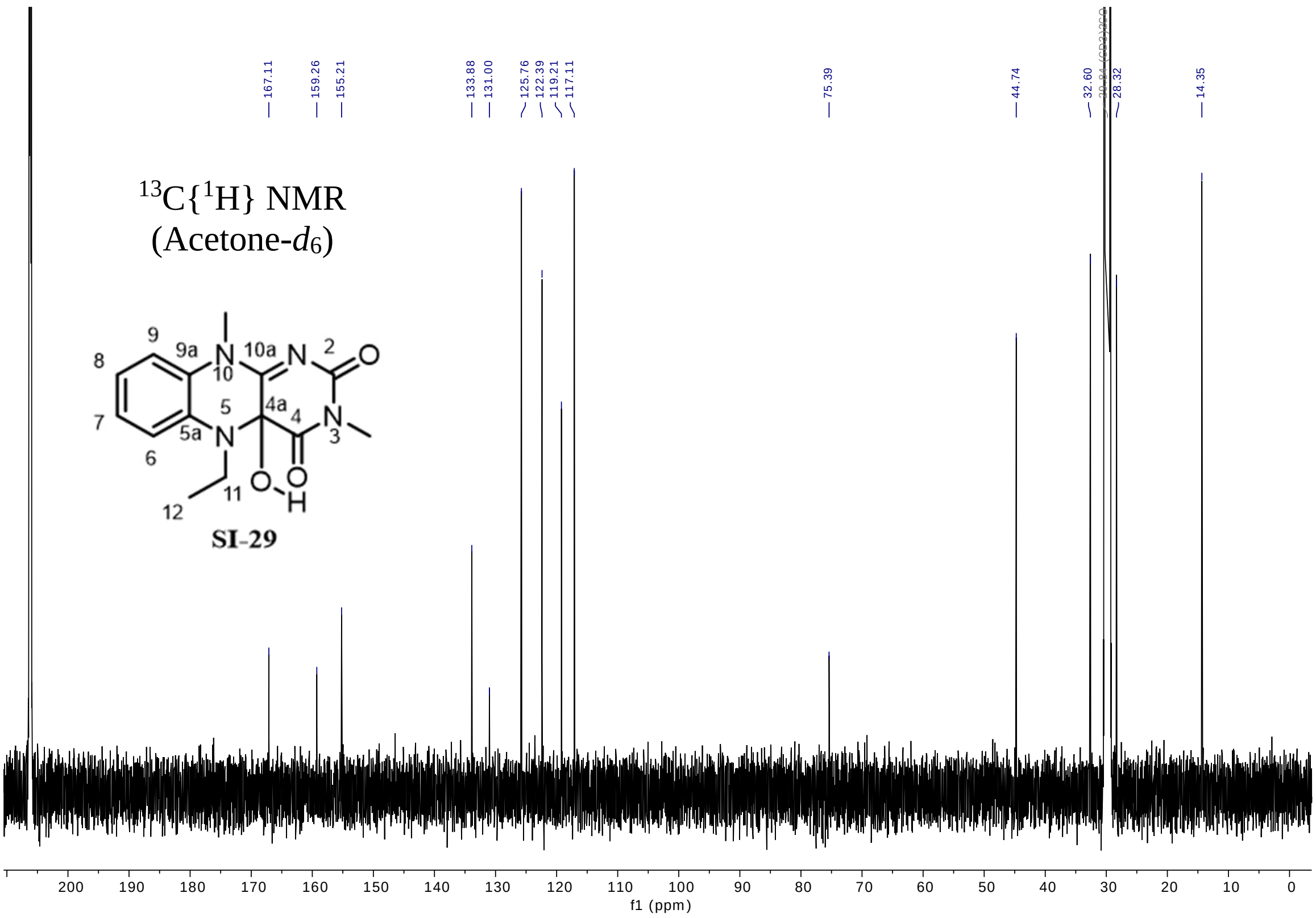
13C{1H} NMR
(Acetone-d6)
SI-29
167.11
159.26
155.21
133.88
131.00
125.76
122.39
119.21
117.11
75.39
44.74
32.60
28.32
14.35
f1 (ppm)

^{1}H NMR ($CDCl_3$)

6a

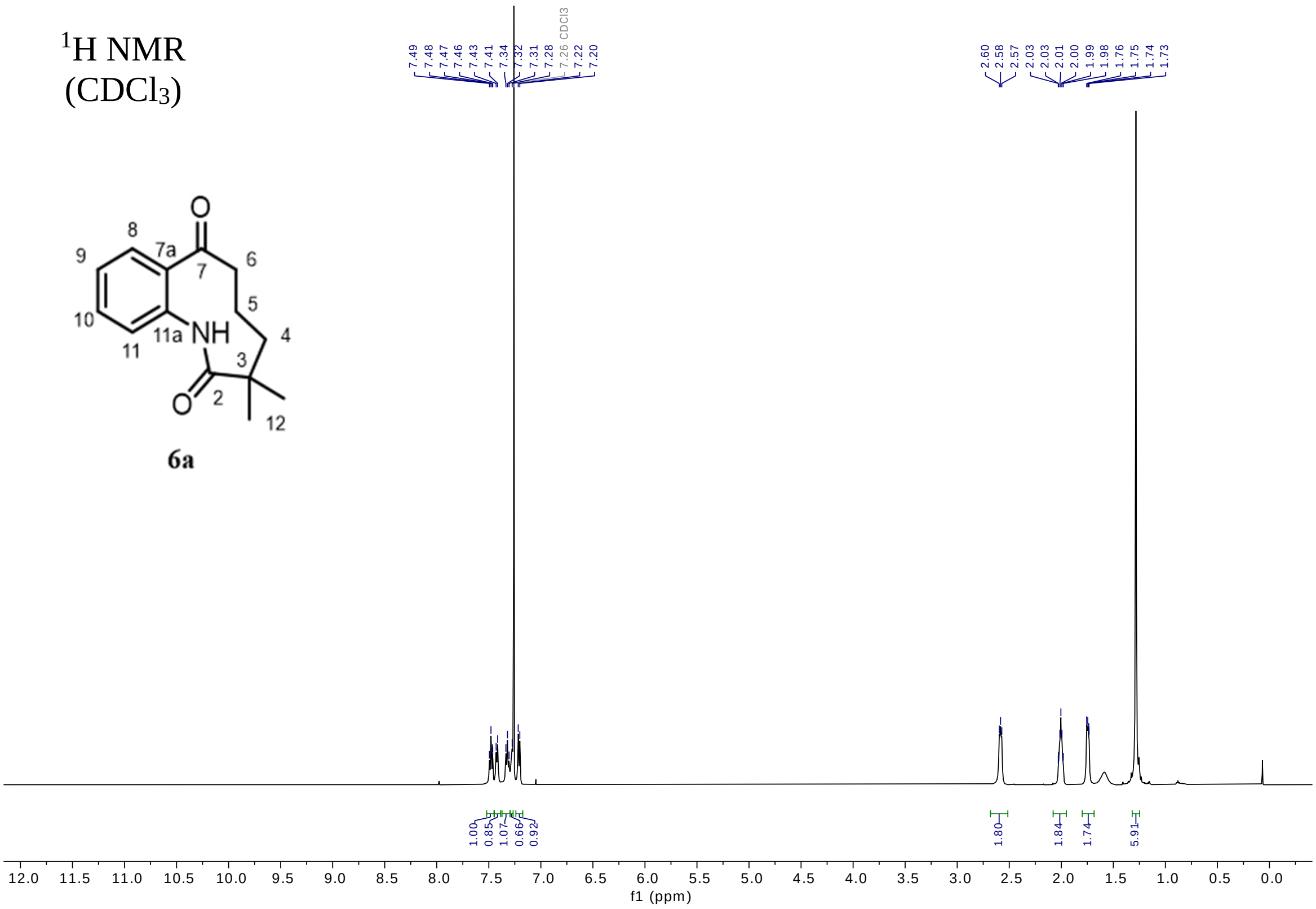

$^{13}C\{^{1}H\}$ NMR ($CDCl_3$)

6a

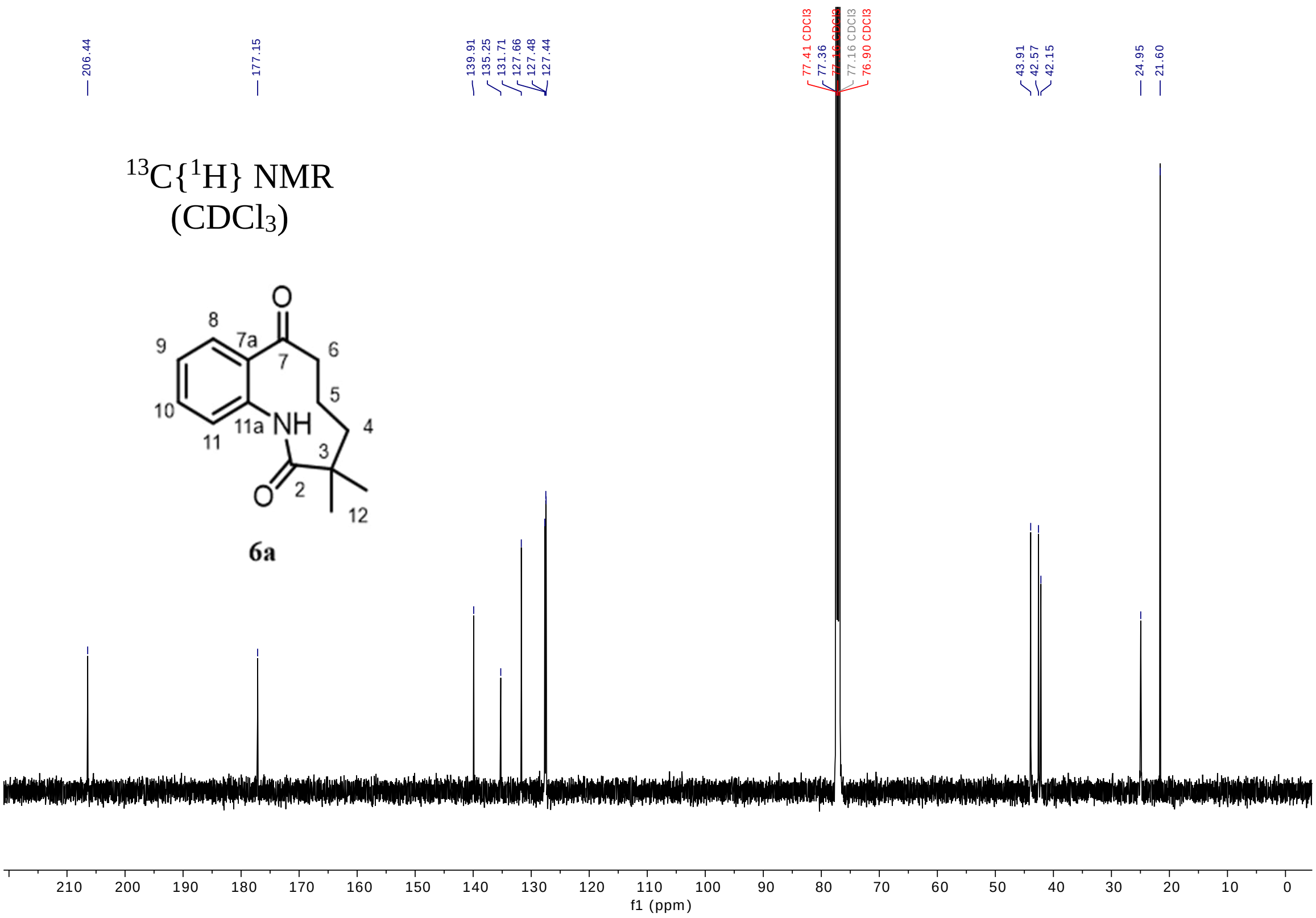

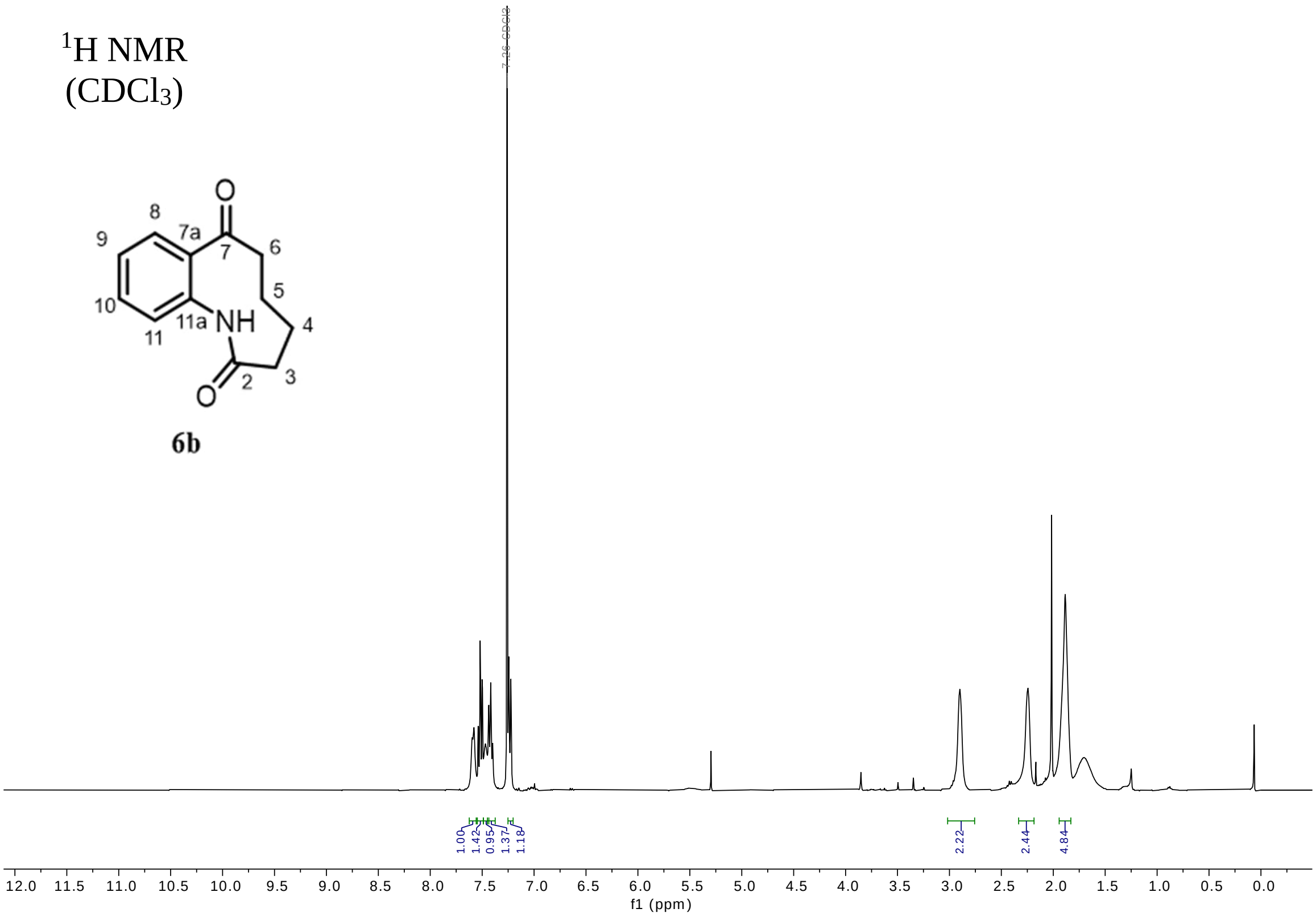
^{1}H NMR
(CDCl$_3$)
6b
8
9
7a
7
6
5
4
3
2
10
11
11a
NH
O
7.26 CDCl3
1.00
1.42
0.95
1.37
1.18
2.22
2.44
4.84
12.0 11.5 11.0 10.5 10.0 9.5 9.0 8.5 8.0 7.5 7.0 6.5 6.0 5.5 5.0 4.5 4.0 3.5 3.0 2.5 2.0 1.5 1.0 0.5 0.0
f1 (ppm)

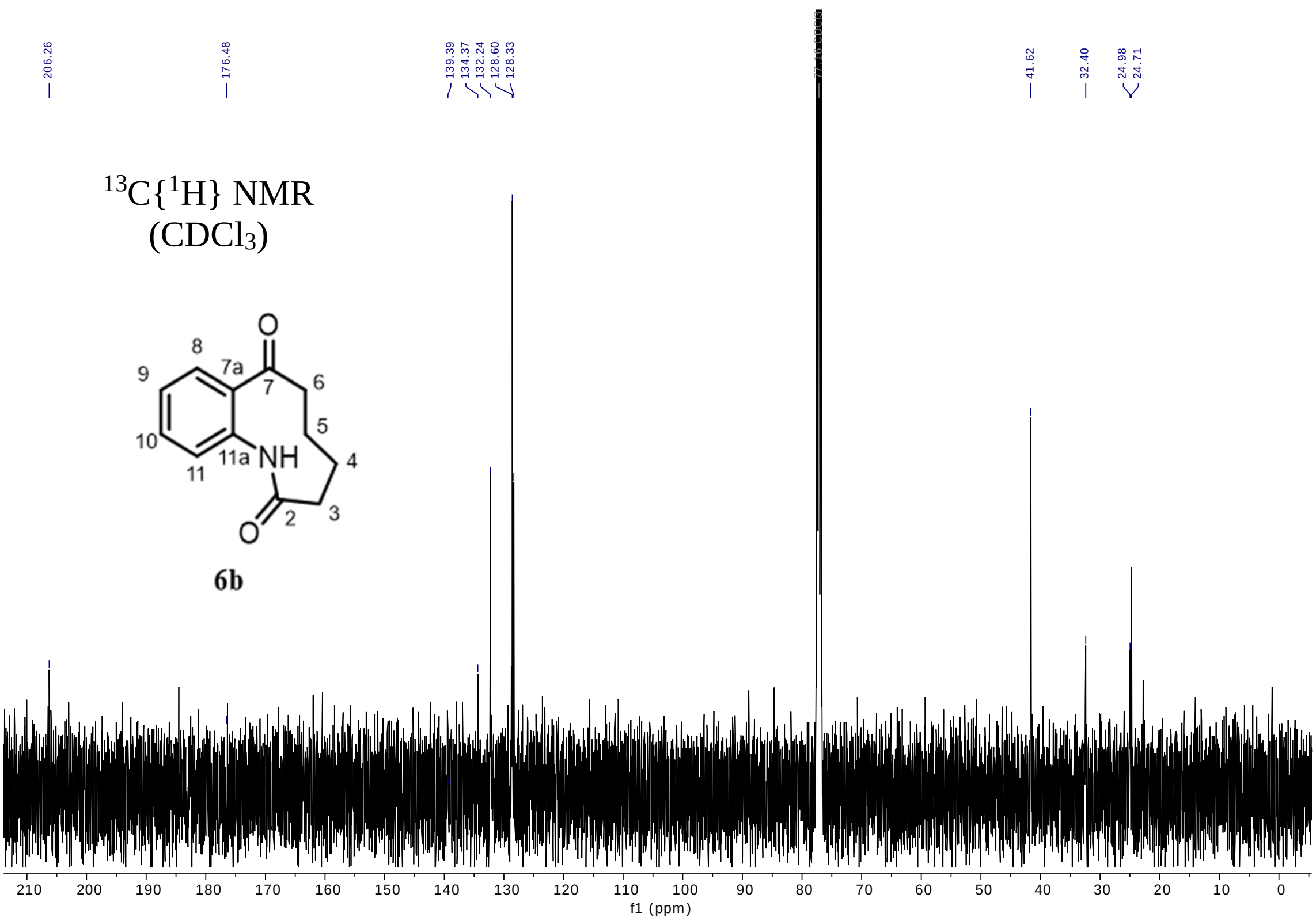
206.26
176.48
139.39
134.37
132.24
128.60
128.33
41.62
32.40
24.98
24.71
^{13}C{^{1}H} NMR
(CDCl$_3$)
6b
8
9
7a
7
6
5
4
3
2
10
11
11a
NH
O
210 200 190 180 170 160 150 140 130 120 110 100 90 80 70 60 50 40 30 20 10 0
f1 (ppm)

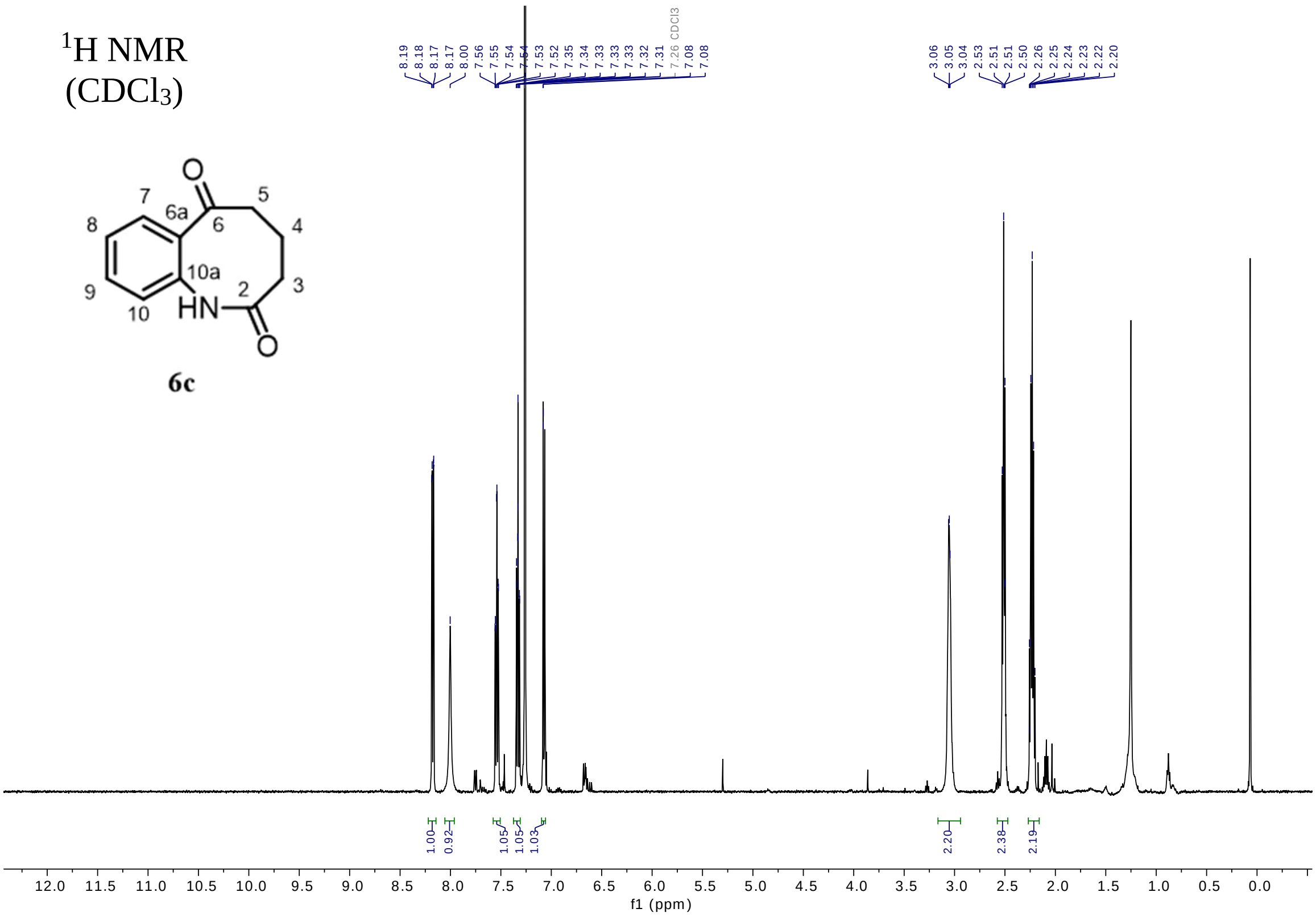
^{1}H NMR
($CDCl_3$)
6c
8.19 8.18 8.17 8.17 8.00 7.56 7.55 7.54 7.54 7.53 7.52 7.35 7.34 7.33 7.33 7.33 7.32 7.31 7.26 CDCl3 7.08 7.08
3.06 3.05 3.04 2.53 2.51 2.51 2.50 2.26 2.25 2.24 2.23 2.22 2.20
1.00 0.92 1.05 1.05 1.03 2.20 2.38 2.19
f1 (ppm)

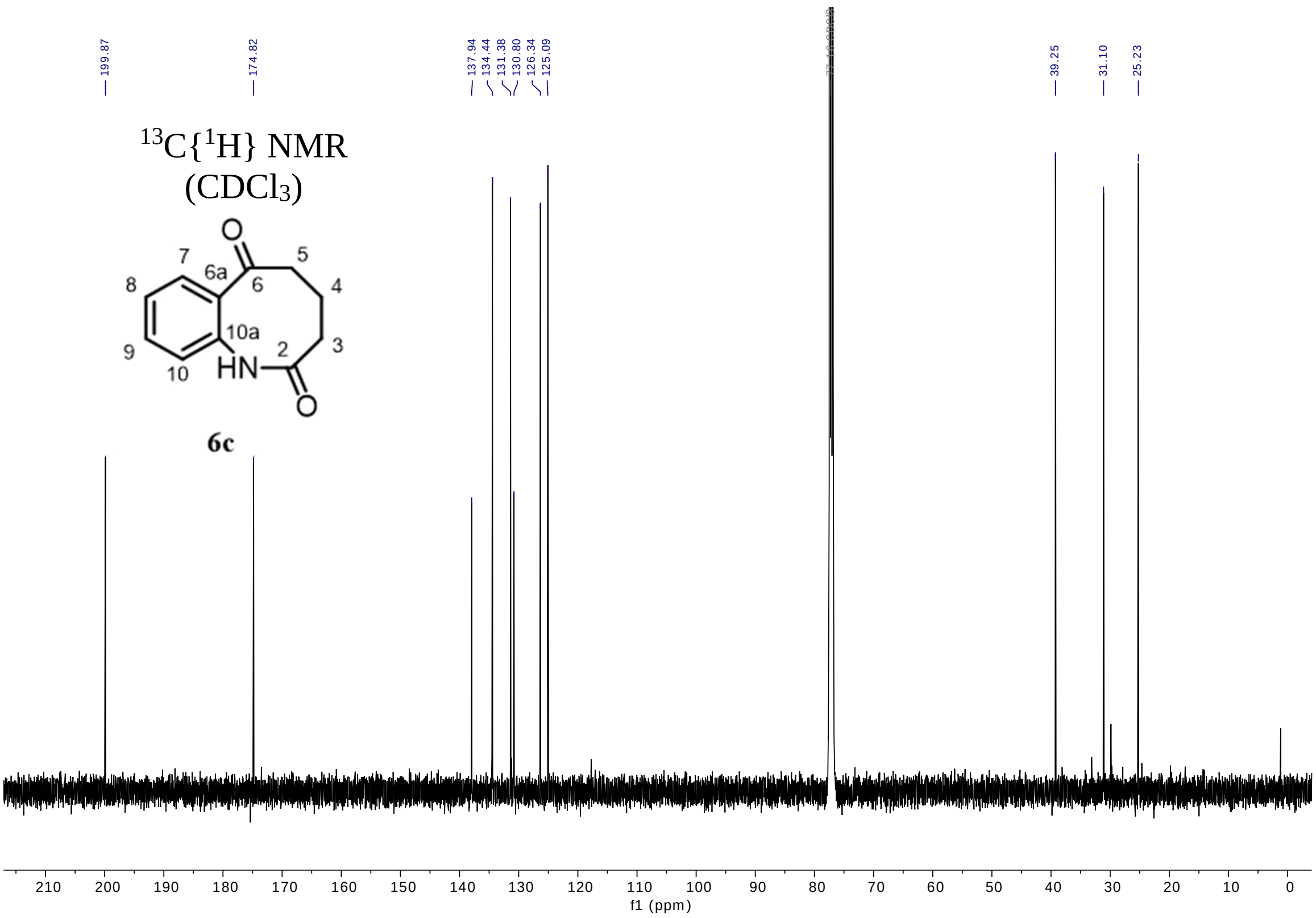
199.87
174.82
137.94 134.44 131.38 130.80 126.34 125.09
39.25
31.10
25.23
^{13}C{^{1}H} NMR
($CDCl_3$)
6c
f1 (ppm)

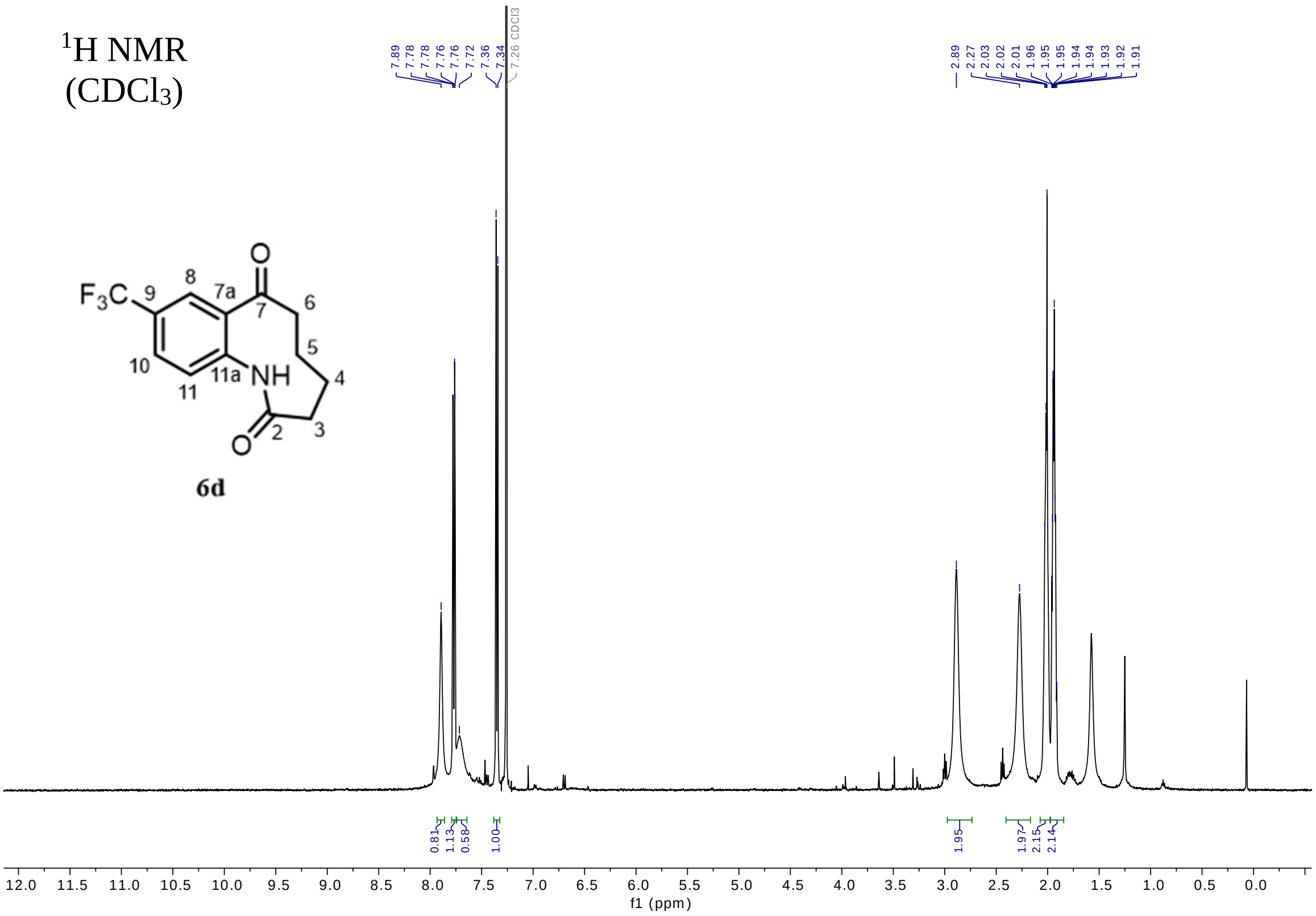
1H NMR
(CDCl3)
F3C 9 8 7a 7 6 5 4 3 2 11a NH 11 10 O
6d
7.89
7.78
7.78
7.76
7.76
7.72
7.36
7.34
7.26 CDCl3
2.89
2.27
2.03
2.02
2.01
1.96
1.95
1.95
1.94
1.94
1.93
1.92
1.91
0.81
1.13
0.58
1.00
1.95
1.97
2.15
2.14
12.0 11.5 11.0 10.5 10.0 9.5 9.0 8.5 8.0 7.5 7.0 6.5 6.0 5.5 5.0 4.5 4.0 3.5 3.0 2.5 2.0 1.5 1.0 0.5 0.0
f1 (ppm)

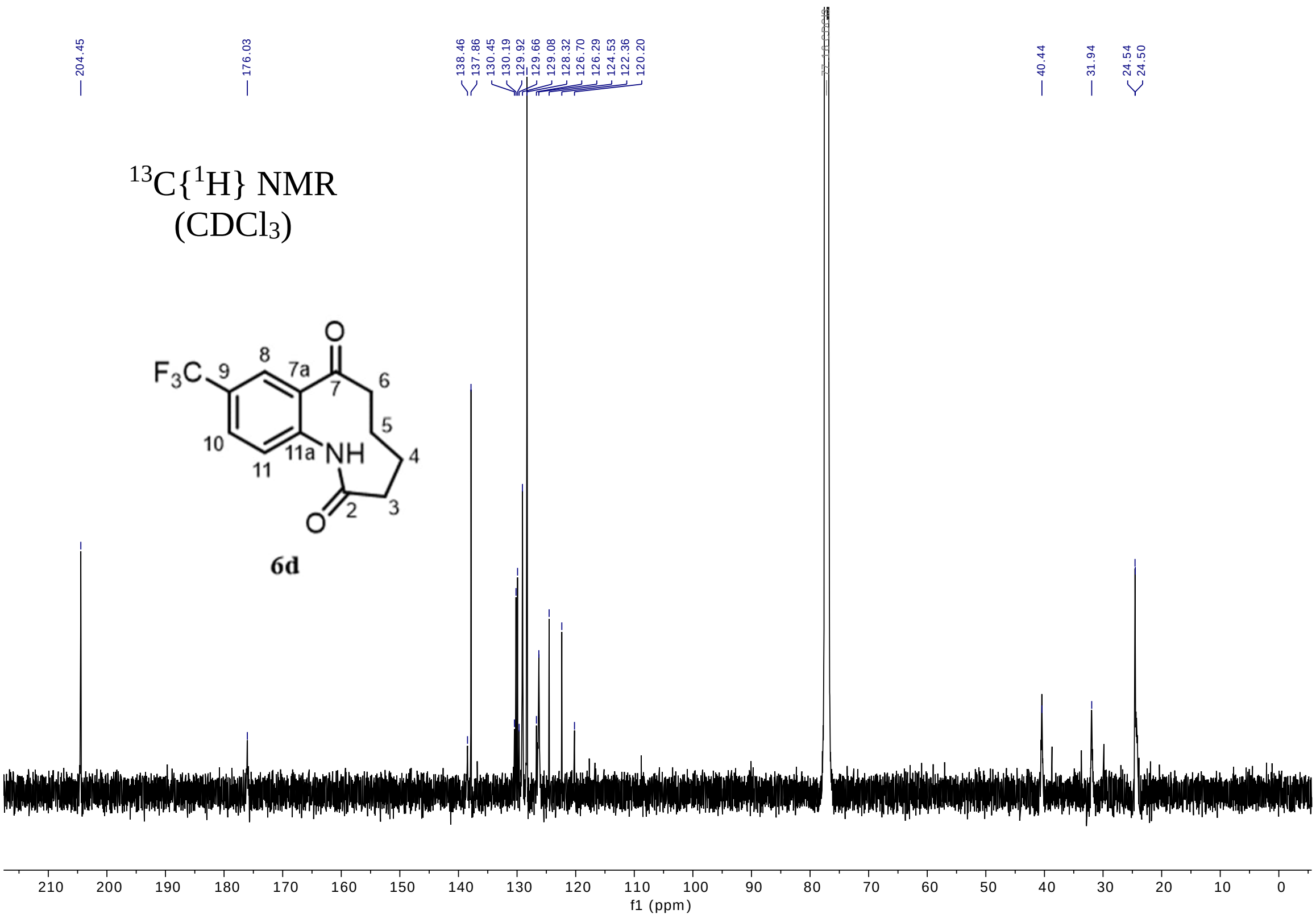
204.45
176.03
138.46
137.86
130.45
130.19
129.92
129.66
129.08
128.32
126.70
126.29
124.53
122.36
120.20
40.44
31.94
24.54
24.50
13C{1H} NMR
(CDCl3)
F3C 9 8 7a 7 6 5 4 3 2 11a NH 11 10 O
6d
210 200 190 180 170 160 150 140 130 120 110 100 90 80 70 60 50 40 30 20 10 0
f1 (ppm)

^{19}F NMR
($CDCl_3$)

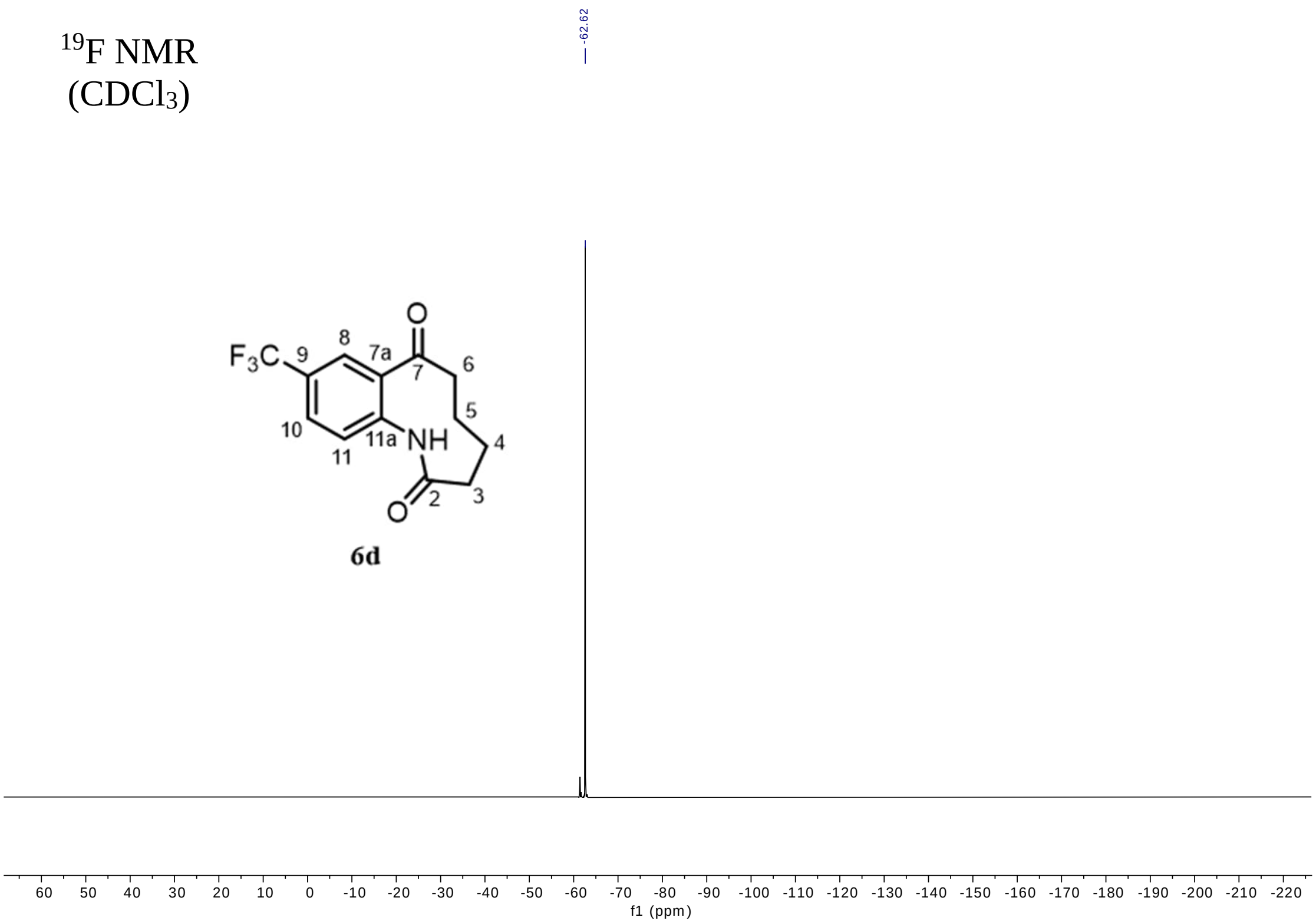

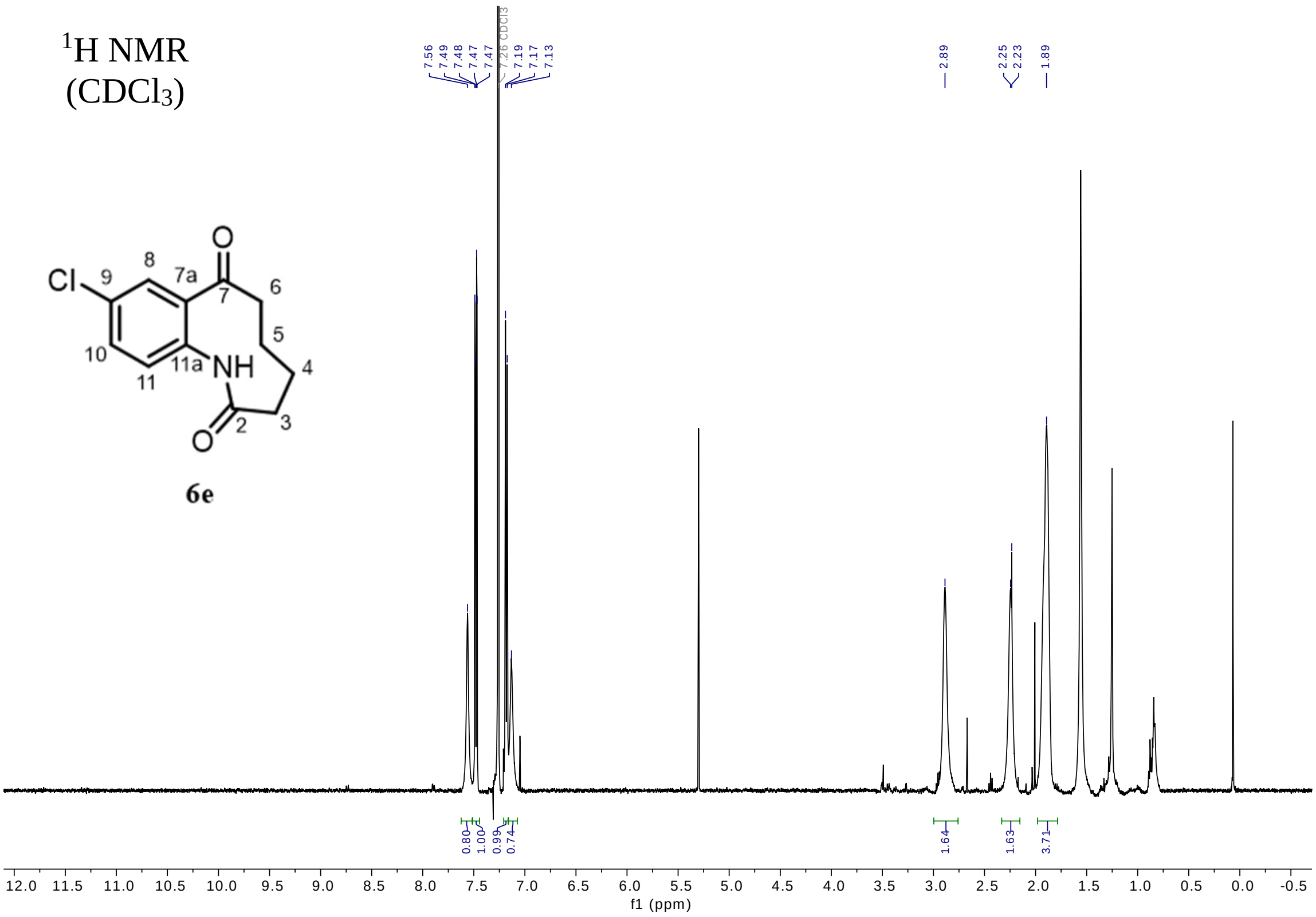

1H NMR
(CDCl3)
7.56
7.49
7.48
7.47
7.47
7.26 CDCl3
7.19
7.17
7.13
2.89
2.25
2.23
1.89
Cl
9
8
7a
7
6
5
4
3
2
10
11
11a
NH
O
O
6e
0.80
1.00
0.99
0.74
1.64
1.63
3.71
f1 (ppm)

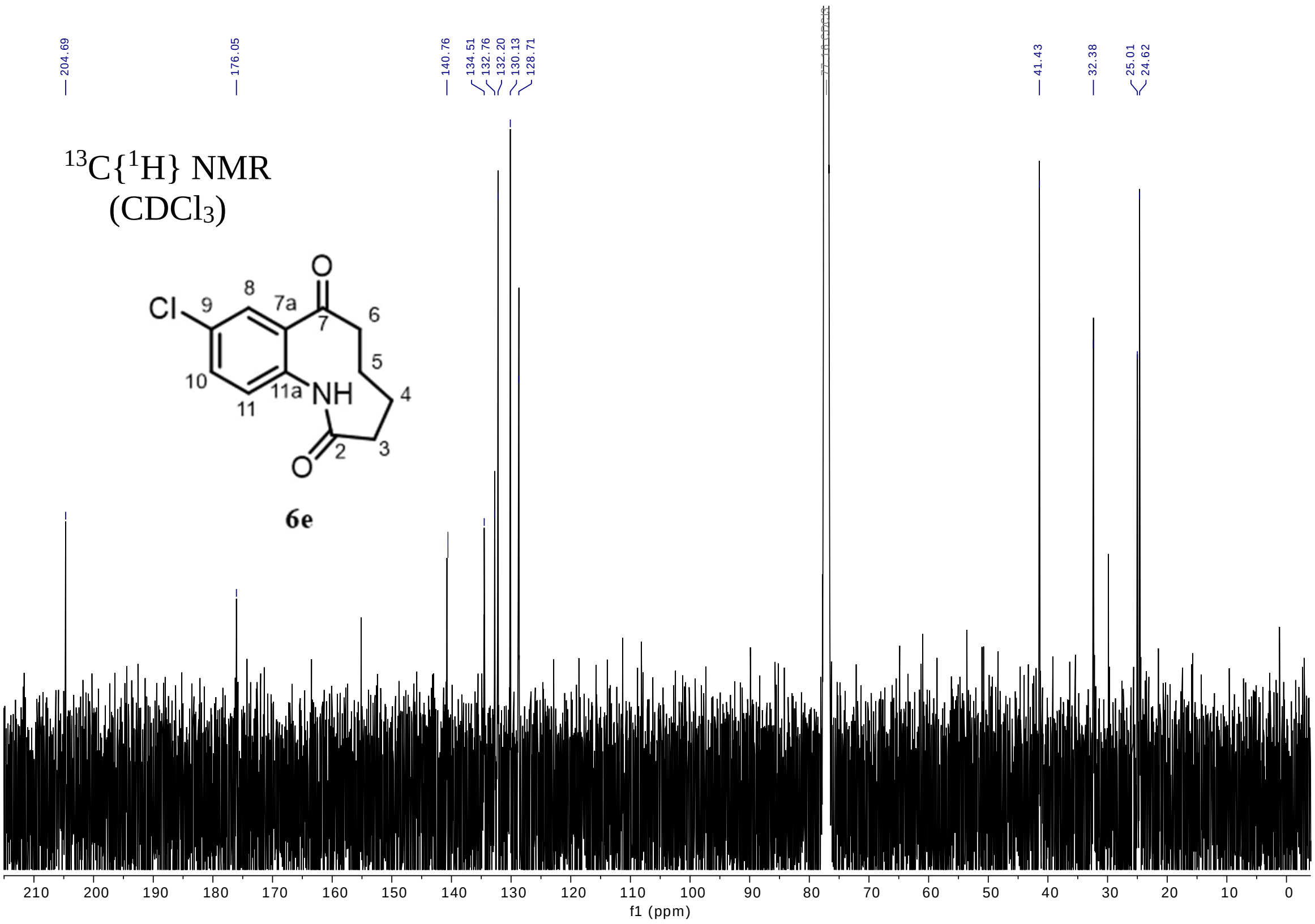

204.69
176.05
140.76
134.51
132.76
132.20
130.13
128.71
41.43
32.38
25.01
24.62
13C{1H} NMR
(CDCl3)
Cl
9
8
7a
7
6
5
4
3
2
10
11
11a
NH
O
O
6e
f1 (ppm)

^{1}H NMR
($CDCl_3$)

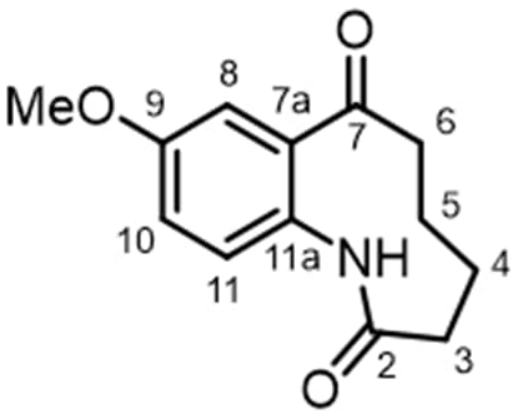

6f

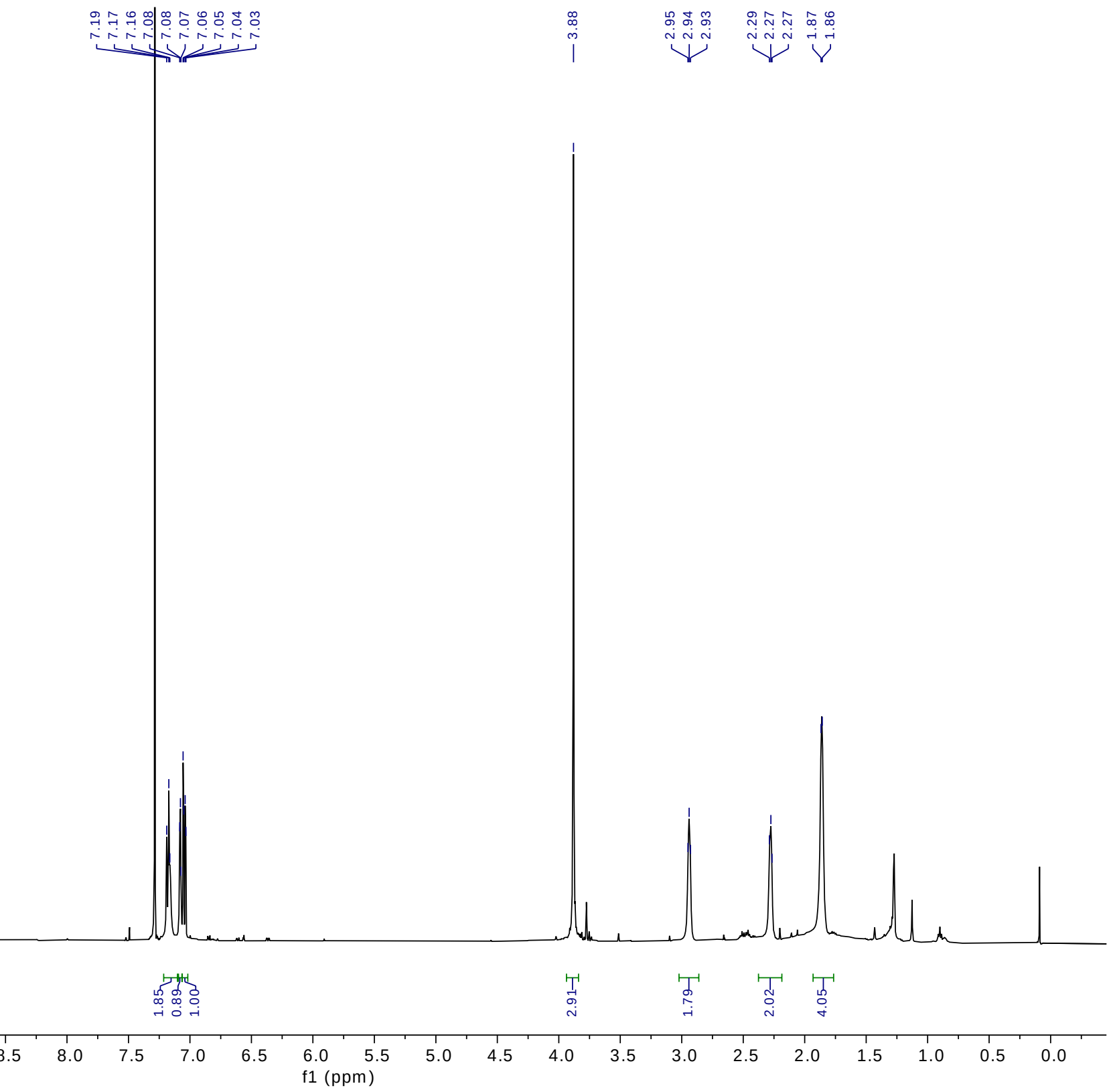

$^{13}C\{^{1}H\}$ NMR
($CDCl_3$)

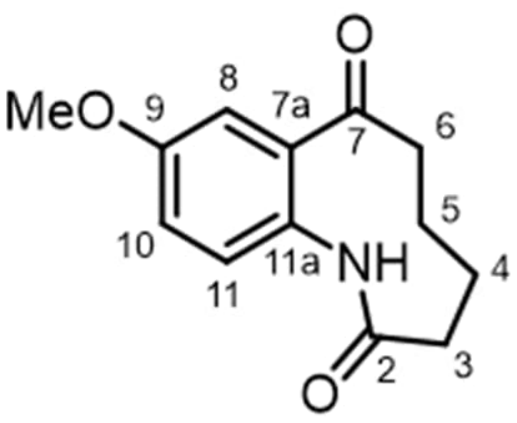

6f

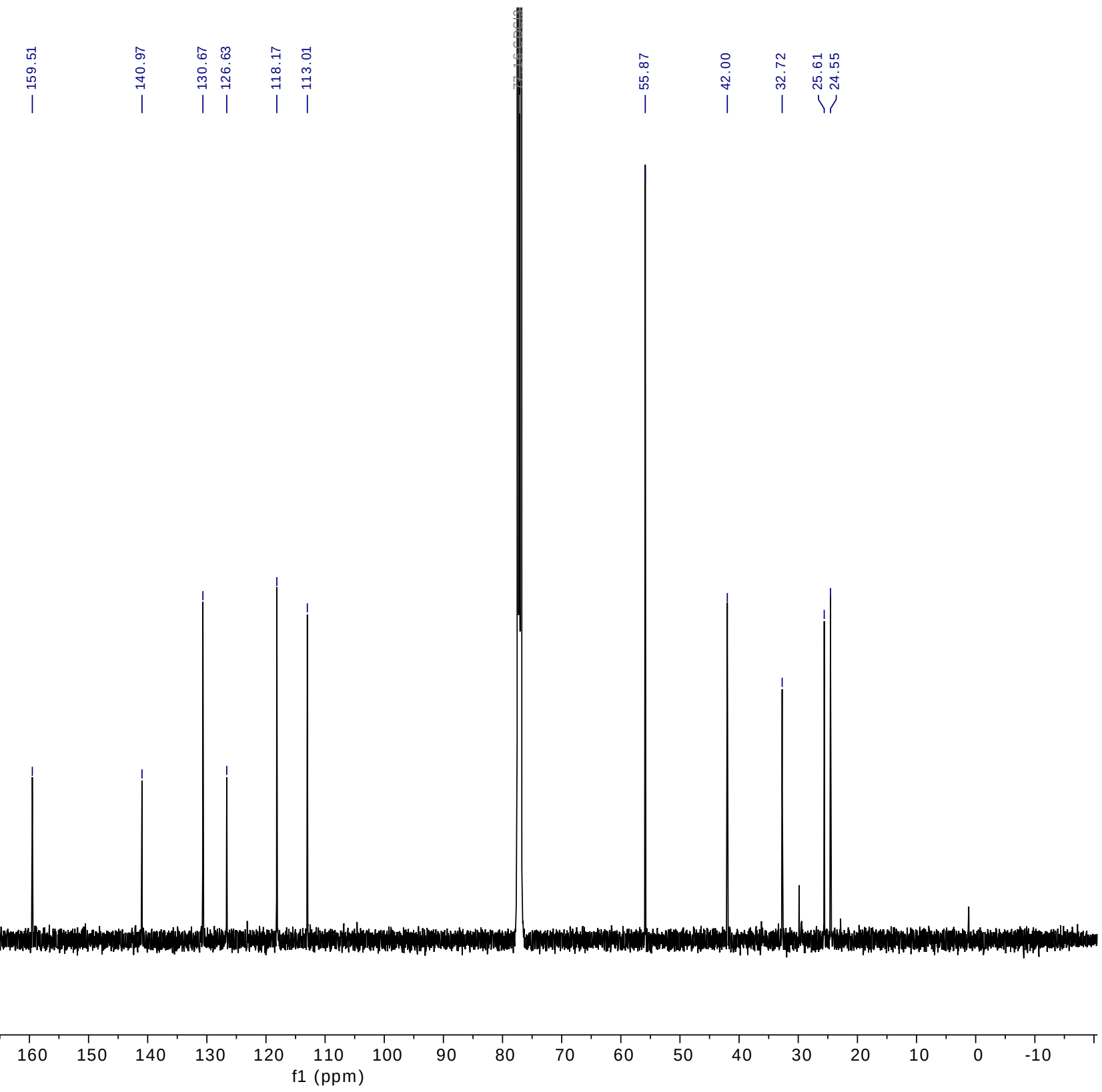

^{1}H NMR
($CDCl_3$)

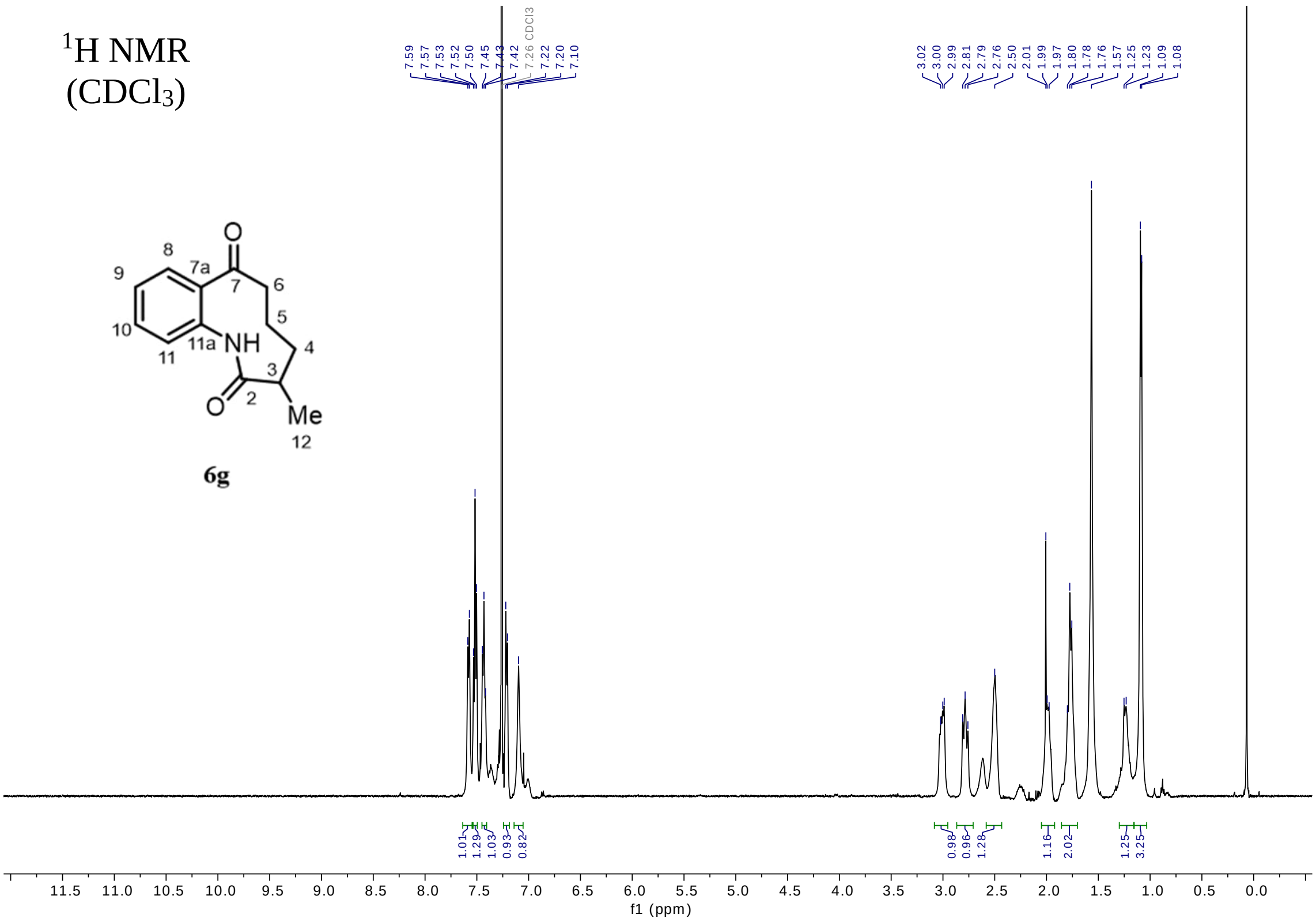

6g

^{13}C{^{1}H} NMR
($CDCl_3$)

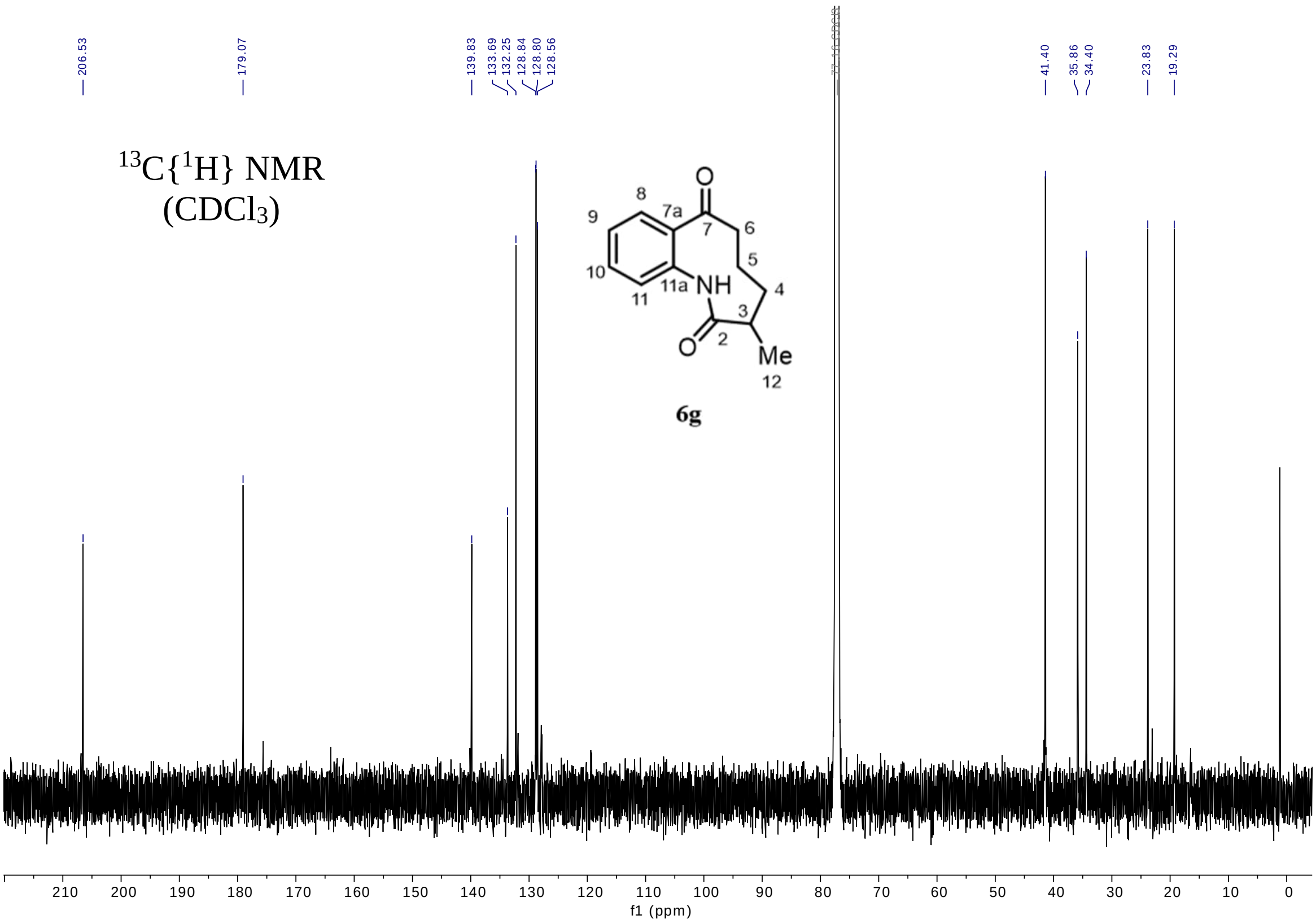

6g

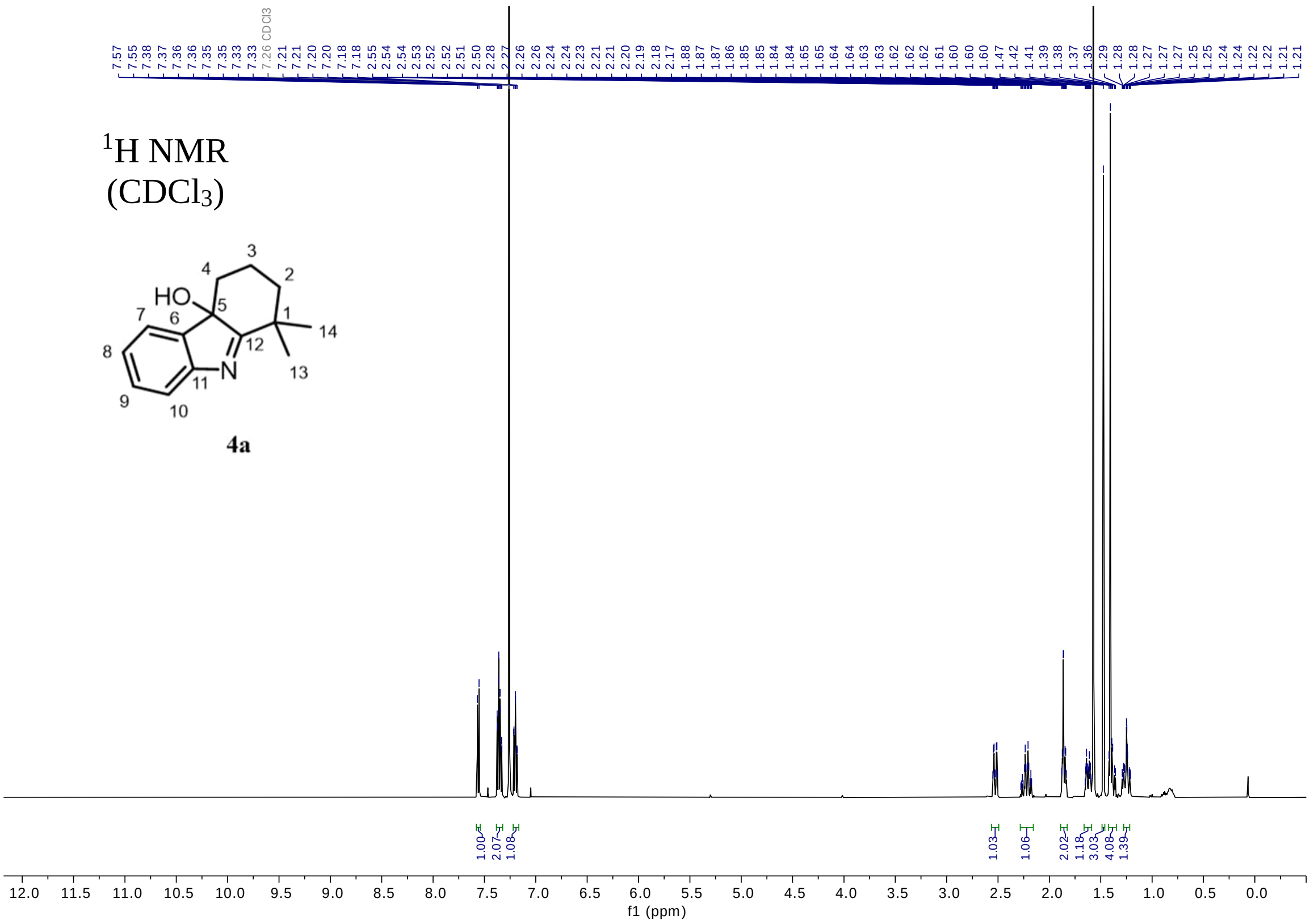
^{1}H NMR (CDCl$_3$)
HO
N
4a
1.00
2.07
1.08
1.03
1.06
2.02
1.18
3.03
4.08
1.39
f1 (ppm)

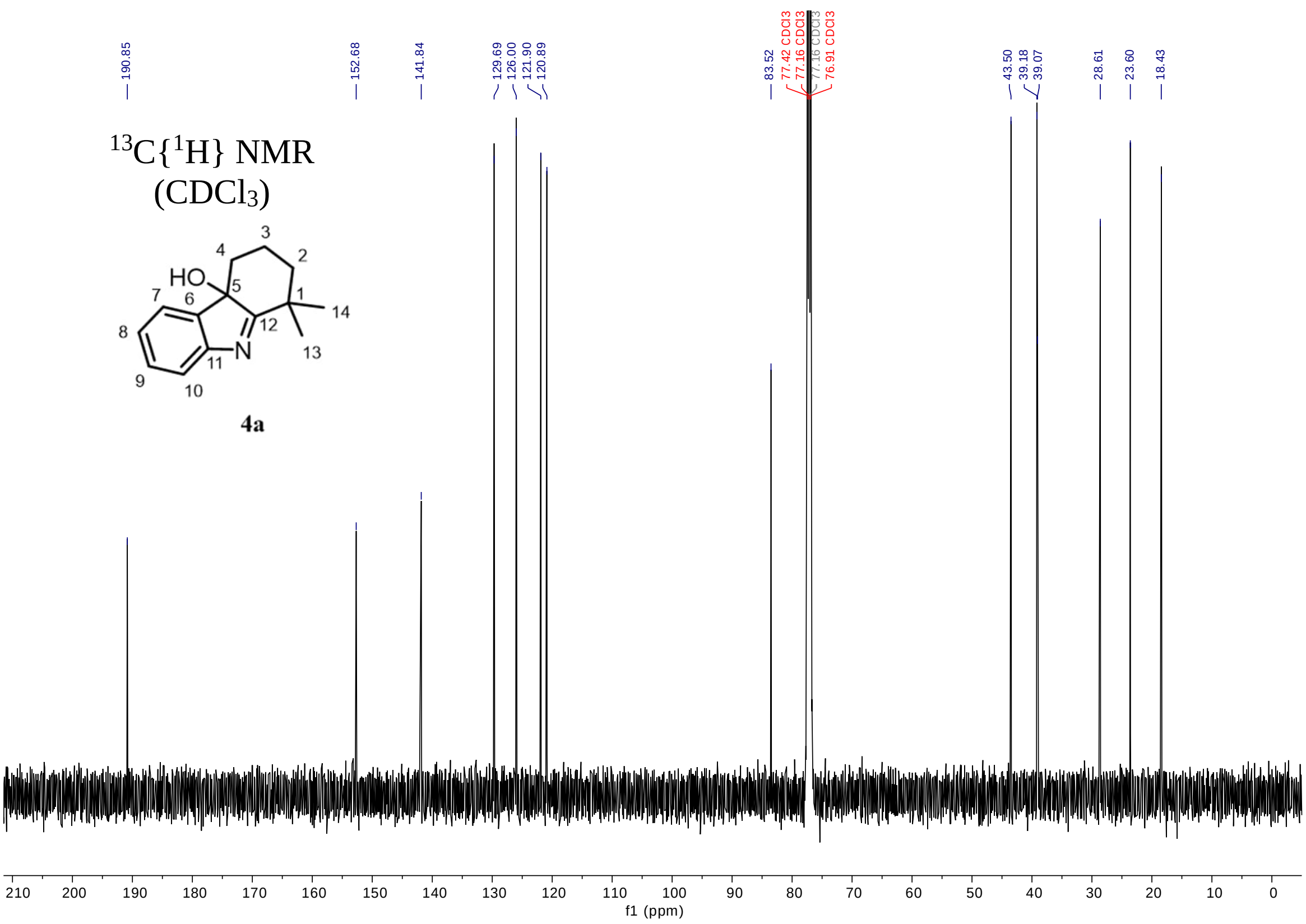
^{13}C{^{1}H} NMR (CDCl$_3$)
HO
N
4a
190.85
152.68
141.84
129.69
126.00
121.90
120.89
83.52
77.42 CDCl3
77.16 CDCl3
76.91 CDCl3
43.50
39.18
39.07
28.61
23.60
18.43
f1 (ppm)

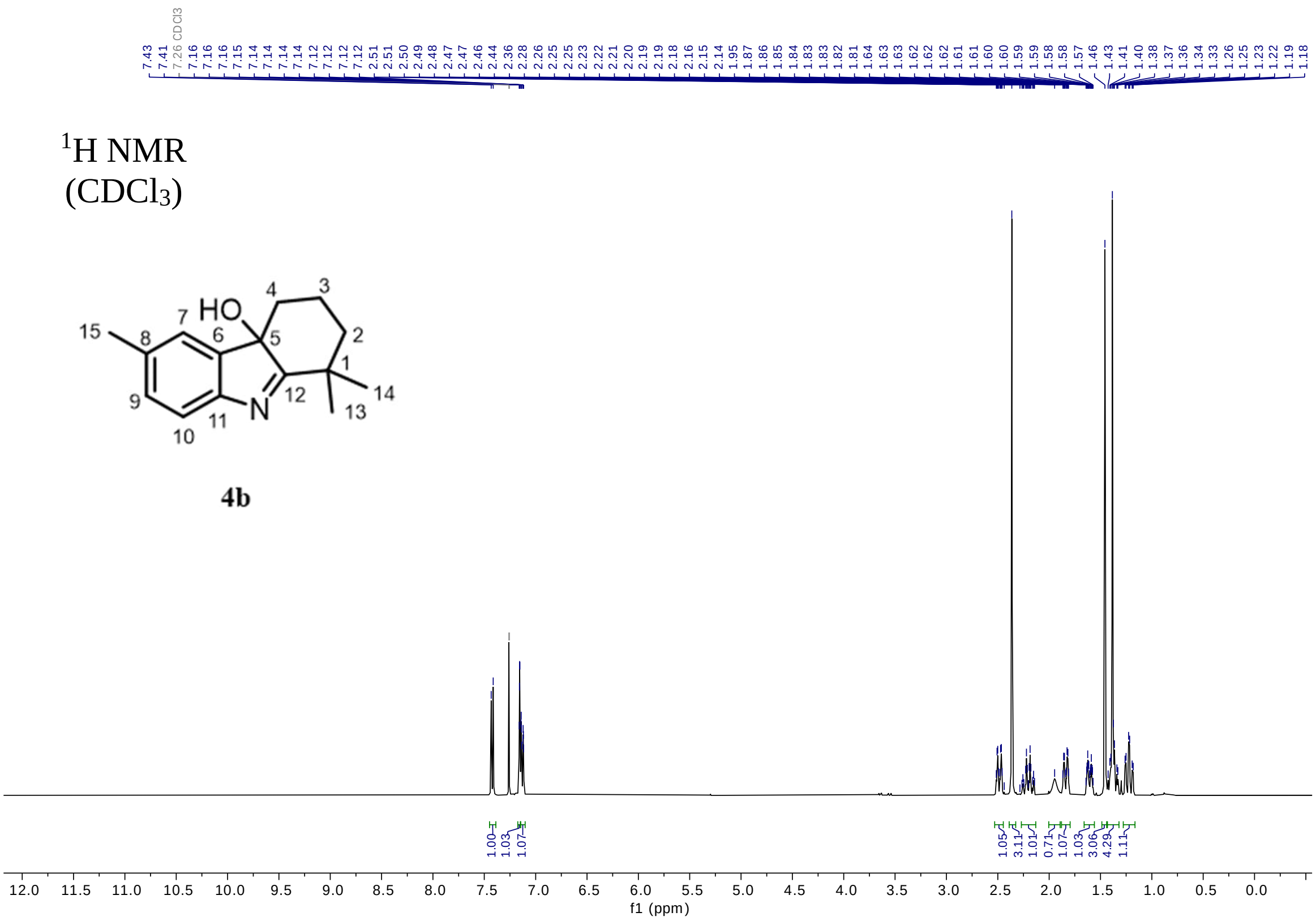

1H NMR
(CDCl3)
4b

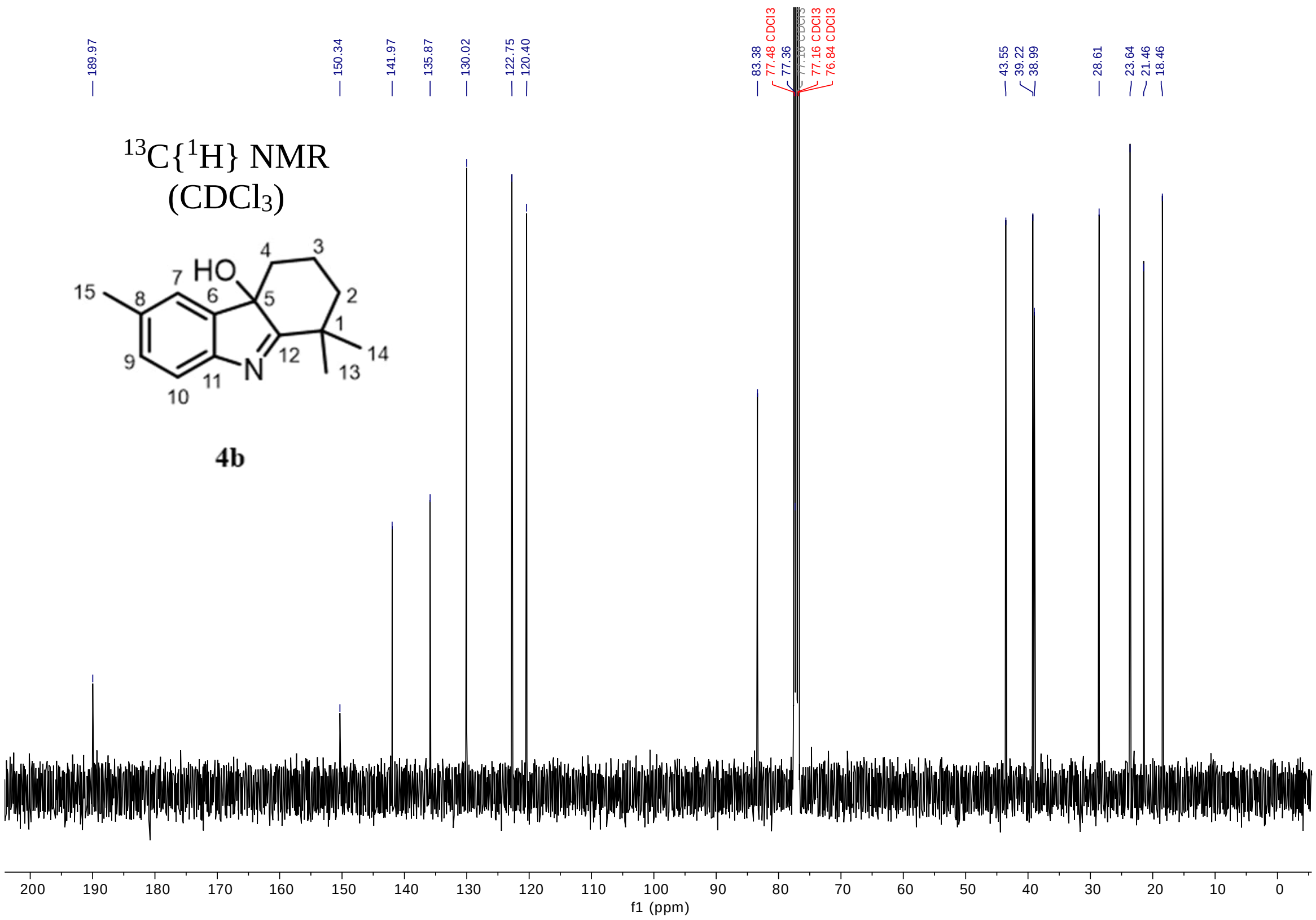

13C{1H} NMR
(CDCl3)
4b

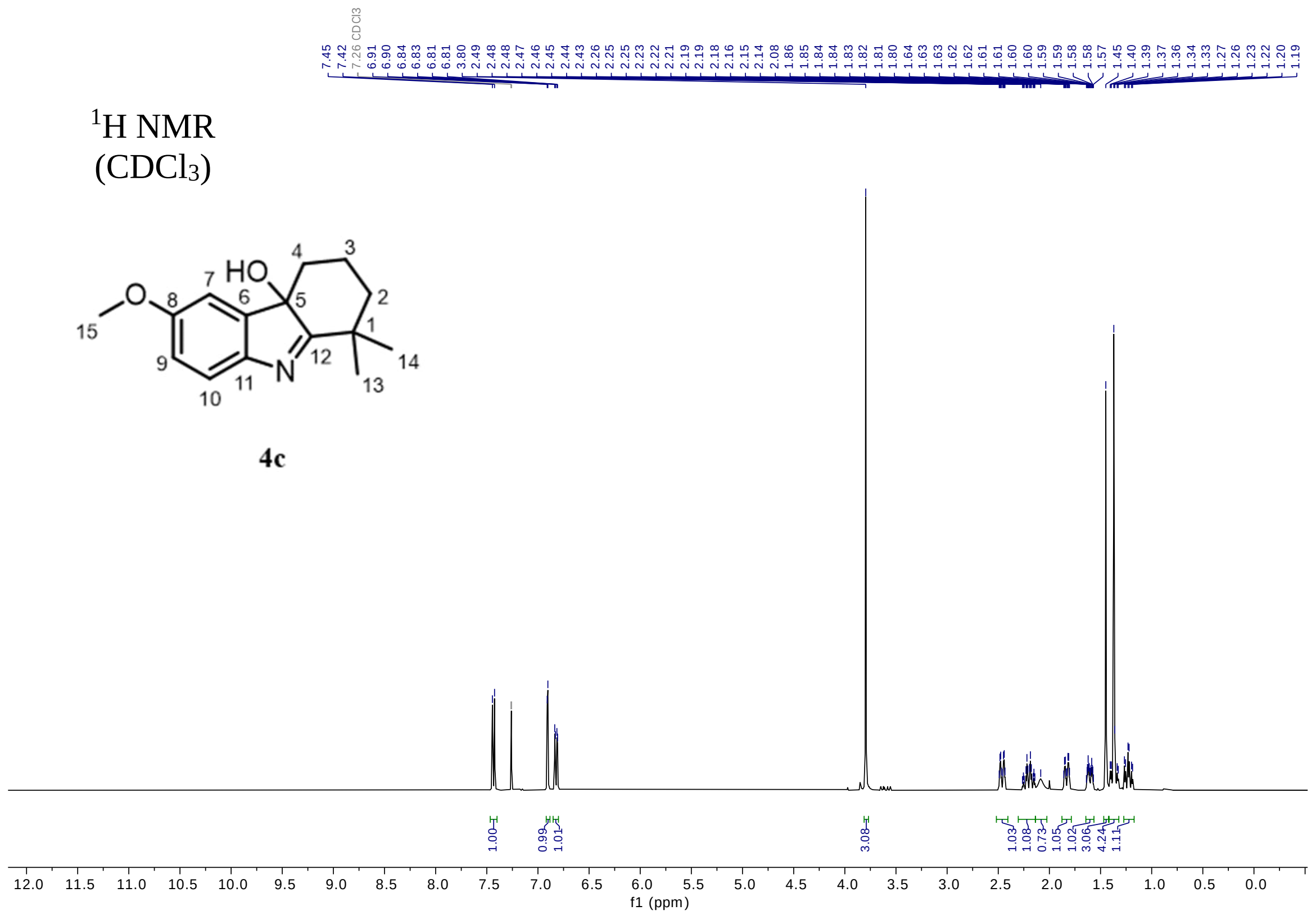
7.45
7.42
7.26 CDCl3
6.91
6.90
6.84
6.83
6.81
6.81
3.80
2.49
2.48
2.48
2.47
2.46
2.45
2.44
2.43
2.26
2.25
2.25
2.23
2.22
2.21
2.19
2.19
2.18
2.16
2.15
2.14
2.08
1.86
1.85
1.84
1.84
1.83
1.82
1.81
1.80
1.64
1.63
1.63
1.62
1.62
1.61
1.61
1.60
1.60
1.59
1.59
1.58
1.58
1.57
1.45
1.40
1.39
1.37
1.36
1.34
1.33
1.27
1.26
1.23
1.22
1.20
1.19
1H NMR
(CDCl3)
HO
O
N
1
2
3
4
5
6
7
8
9
10
11
12
13
14
15
4c
1.00
0.99
1.01
3.08
1.03
1.08
0.73
1.05
1.02
3.06
4.24
1.11
12.0 11.5 11.0 10.5 10.0 9.5 9.0 8.5 8.0 7.5 7.0 6.5 6.0 5.5 5.0 4.5 4.0 3.5 3.0 2.5 2.0 1.5 1.0 0.5 0.0
f1 (ppm)

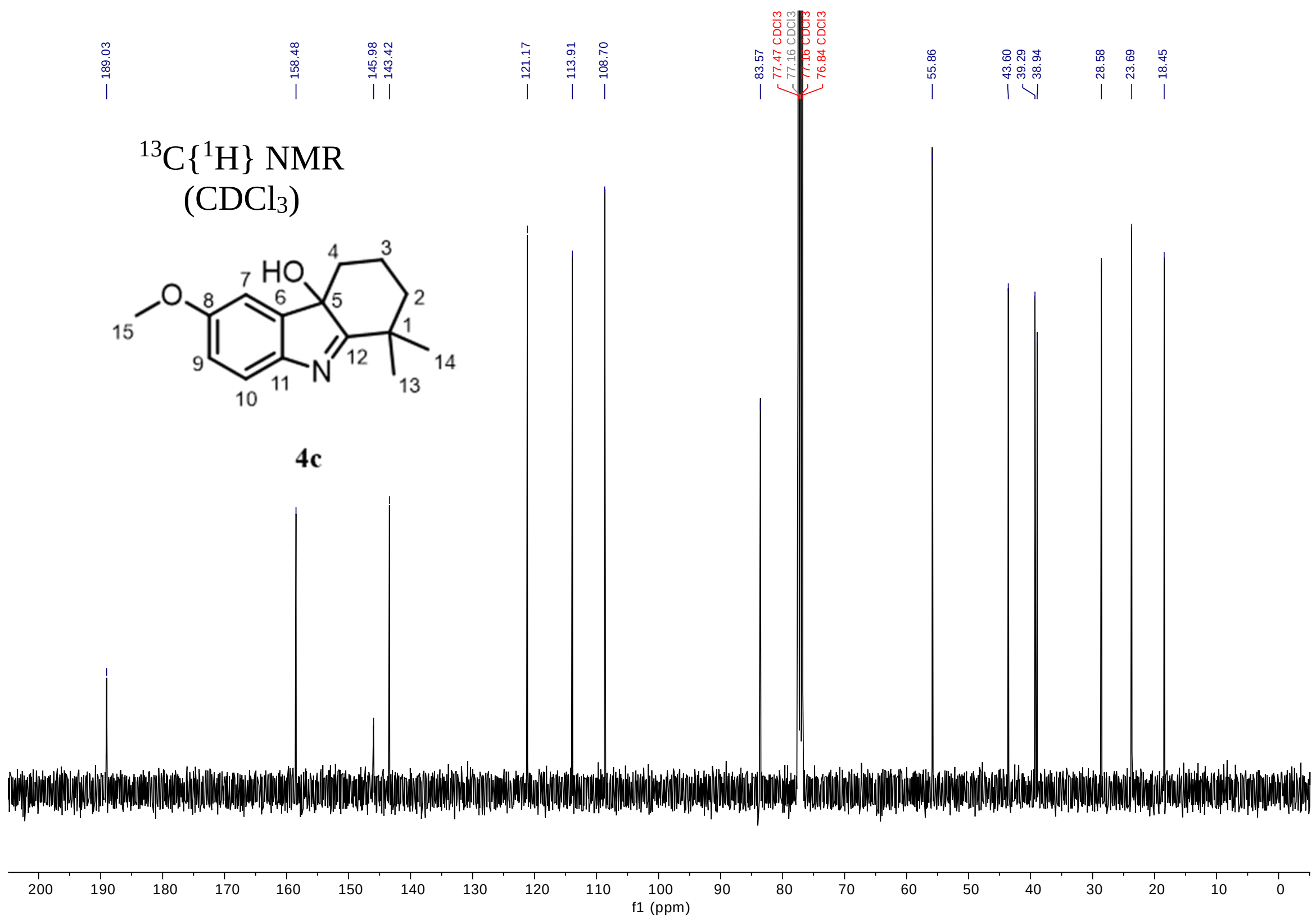
189.03
158.48
145.98
143.42
121.17
113.91
108.70
83.57
77.47 CDCl3
77.16 CDCl3
77.16 CDCl3
76.84 CDCl3
55.86
43.60
39.29
38.94
28.58
23.69
18.45
13C{1H} NMR
(CDCl3)
HO
O
N
1
2
3
4
5
6
7
8
9
10
11
12
13
14
15
4c
200 190 180 170 160 150 140 130 120 110 100 90 80 70 60 50 40 30 20 10 0
f1 (ppm)

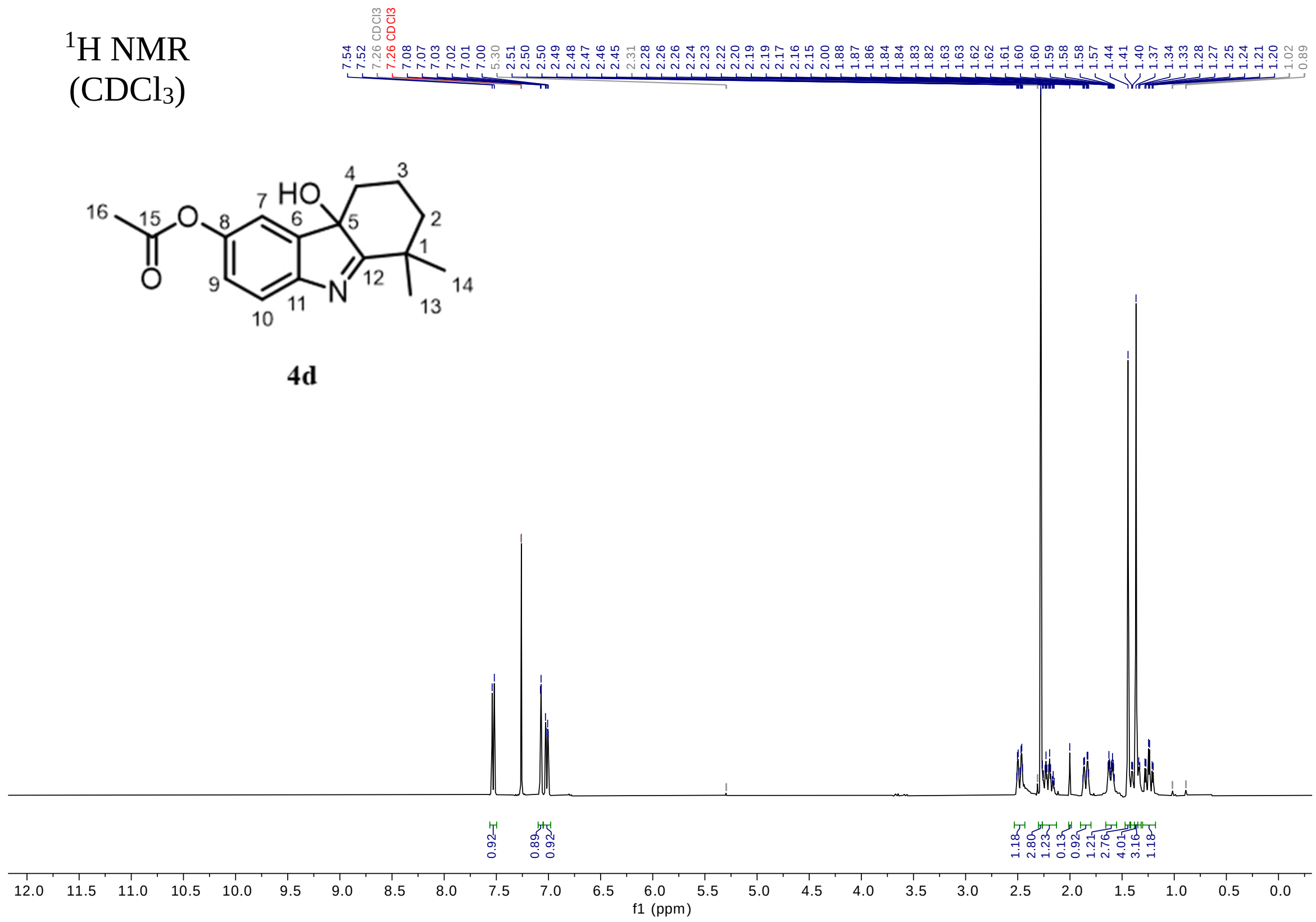

1H NMR
(CDCl3)
4d
7.54
7.52
7.26 CDCl3
7.26 CDCl3
7.08
7.07
7.03
7.02
7.01
7.00
5.30
2.51
2.50
2.50
2.49
2.48
2.47
2.46
2.45
2.31
2.28
2.26
2.26
2.24
2.23
2.22
2.20
2.19
2.19
2.17
2.16
2.15
2.00
1.88
1.87
1.86
1.84
1.84
1.83
1.82
1.63
1.63
1.62
1.62
1.61
1.60
1.60
1.59
1.58
1.58
1.57
1.44
1.41
1.40
1.37
1.34
1.33
1.28
1.27
1.25
1.24
1.21
1.20
1.02
0.89
0.92
0.89
0.92
1.18
2.80
1.23
0.13
0.92
1.21
2.76
4.01
3.16
1.18
f1 (ppm)

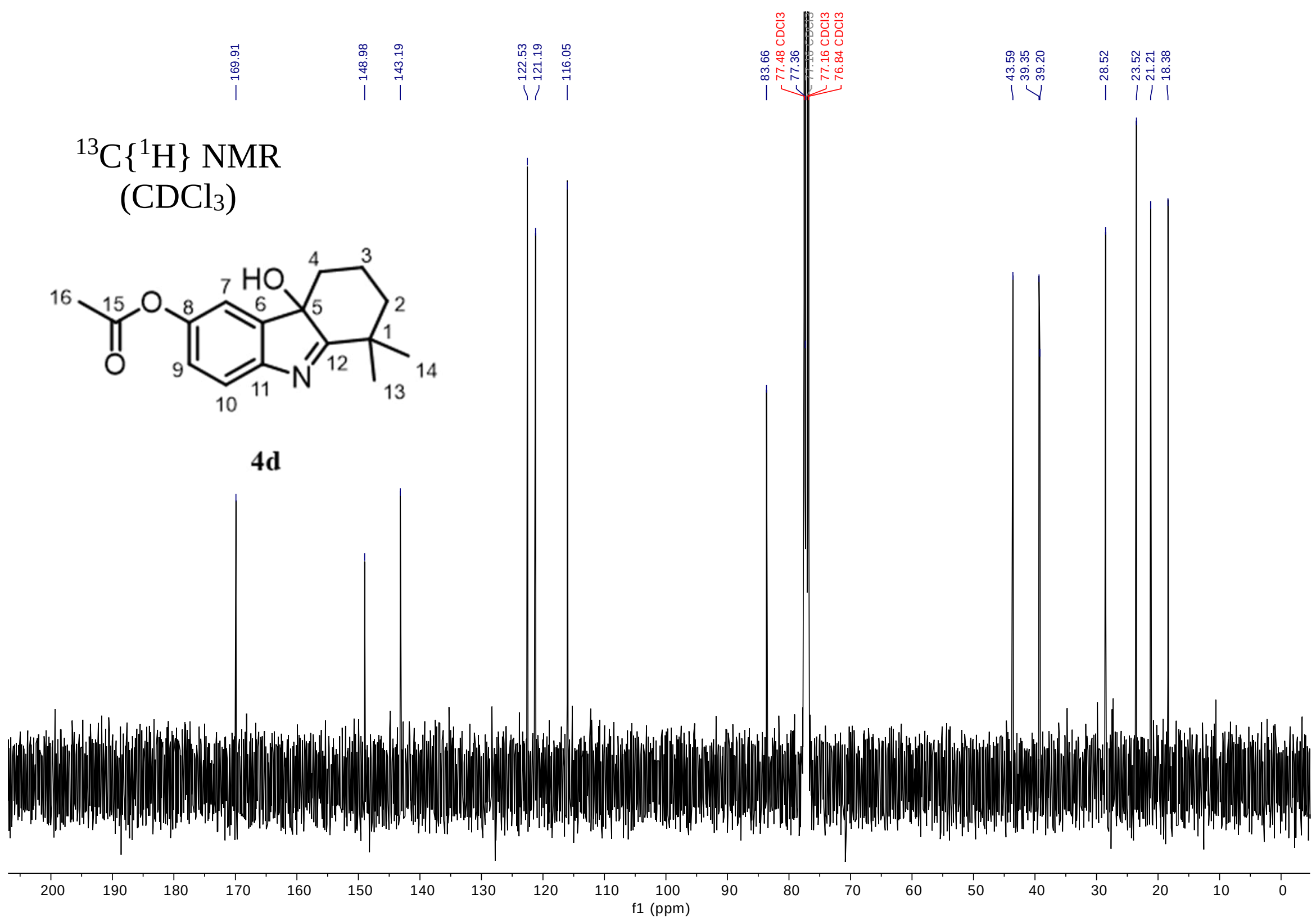

13C{1H} NMR
(CDCl3)
4d
169.91
148.98
143.19
122.53
121.19
116.05
83.66
77.48 CDCl3
77.36
77.16 CDCl3
76.84 CDCl3
43.59
39.35
39.20
28.52
23.52
21.21
18.38
f1 (ppm)

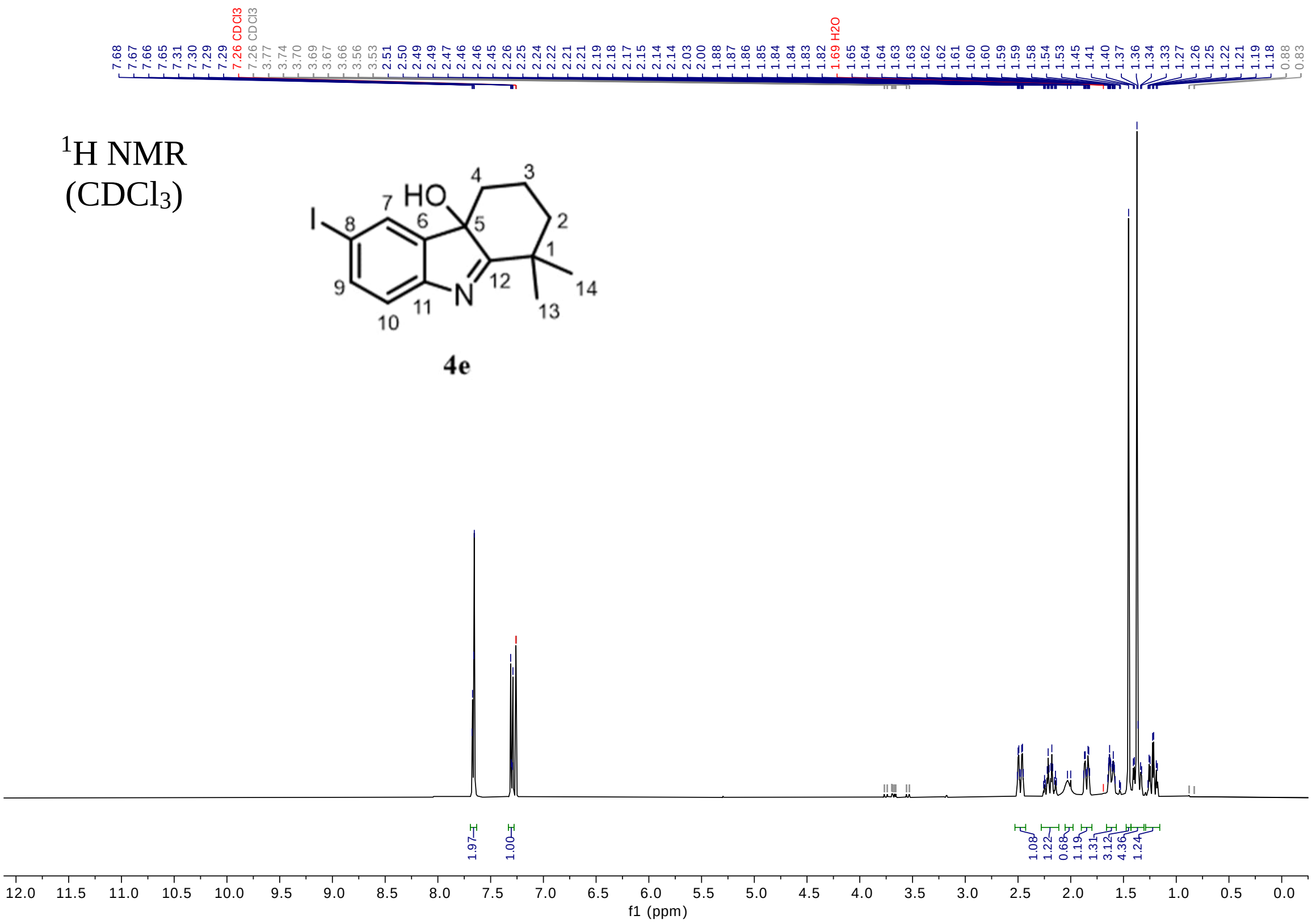
1H NMR
(CDCl3)
4e
f1 (ppm)

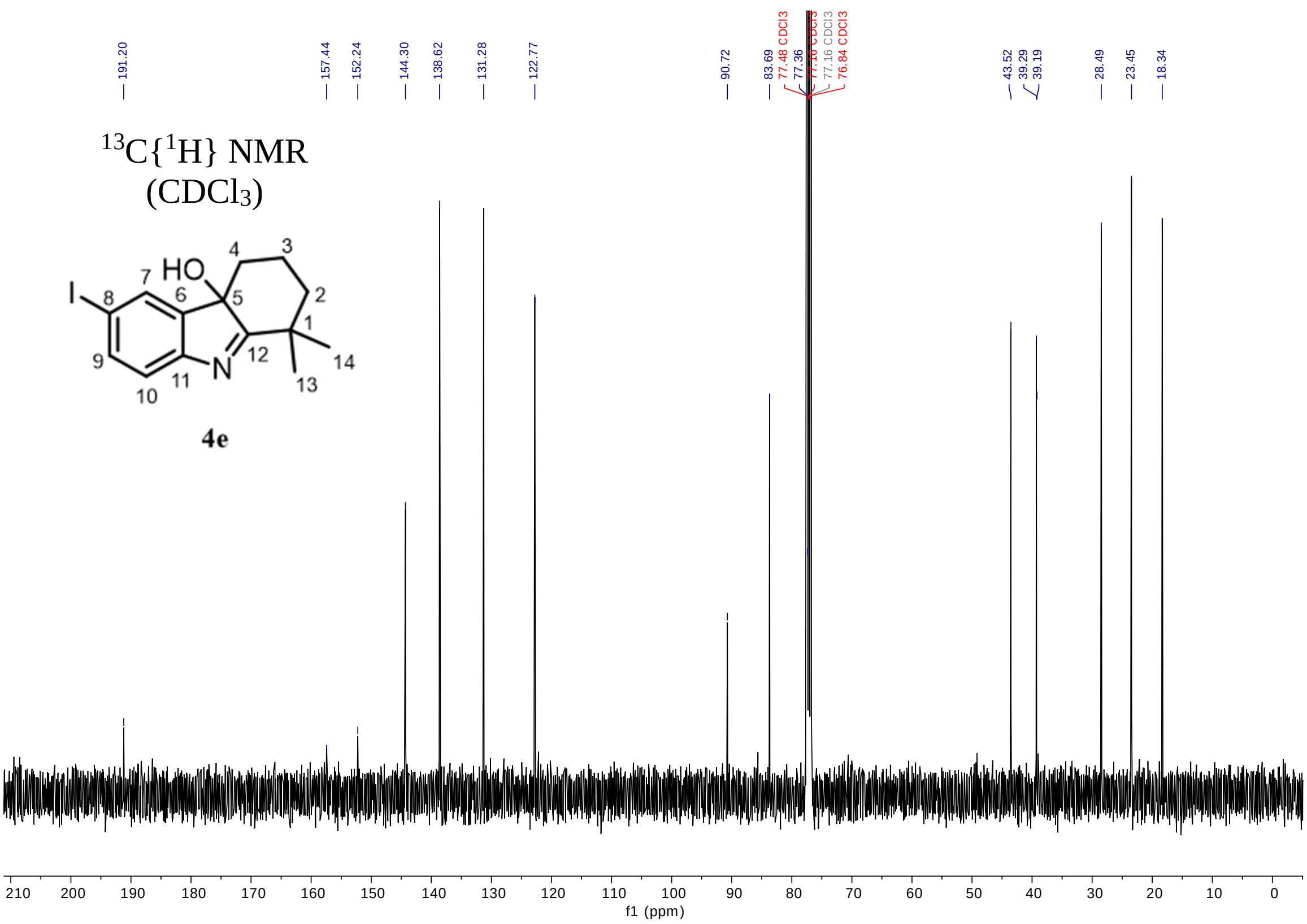
13C{1H} NMR
(CDCl3)
4e
f1 (ppm)

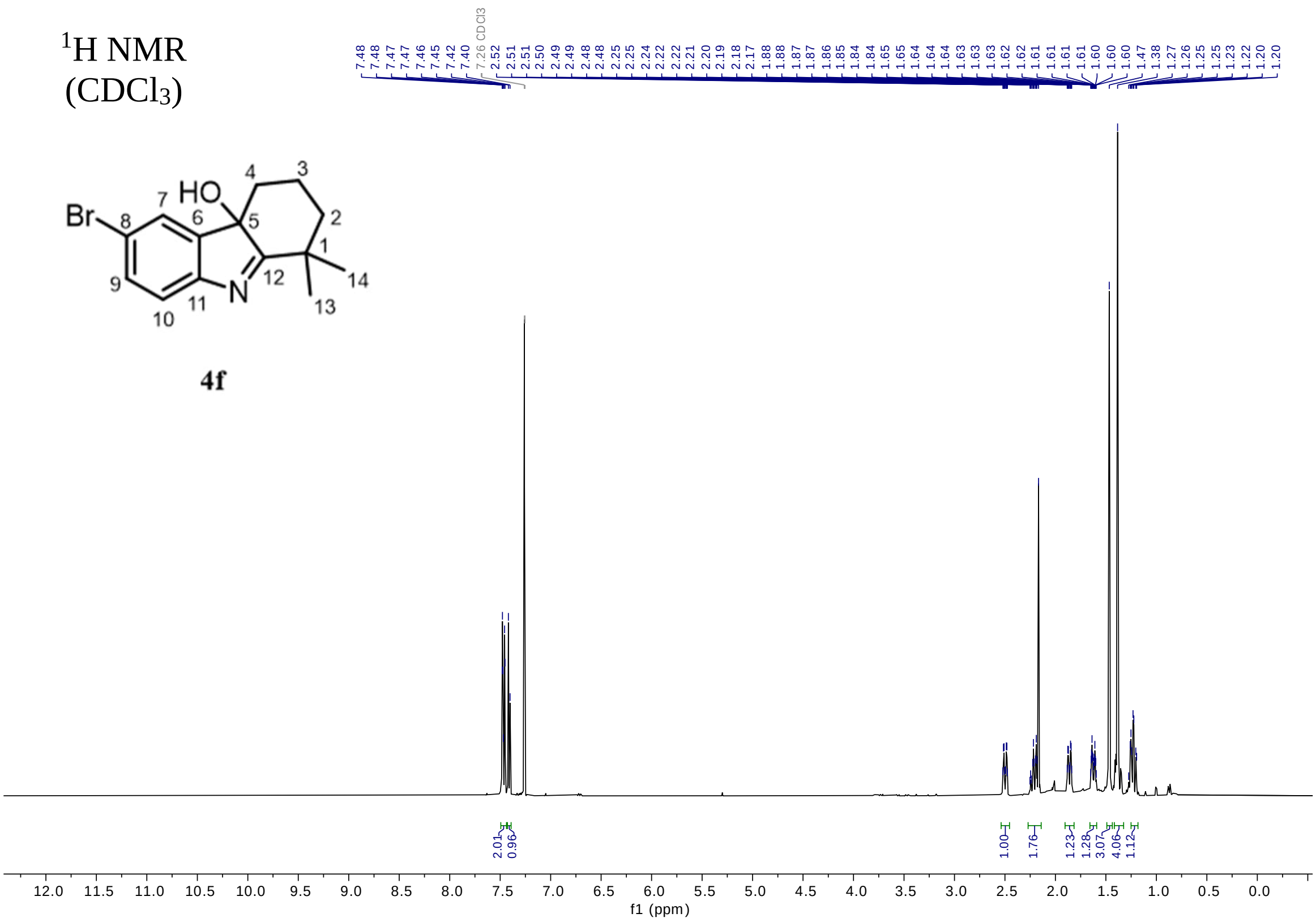

1H NMR
(CDCl3)
Br
HO
N
4f
7.26 CDCl3
2.01
0.96
1.00
1.76
1.23
1.28
3.07
4.06
1.12
f1 (ppm)

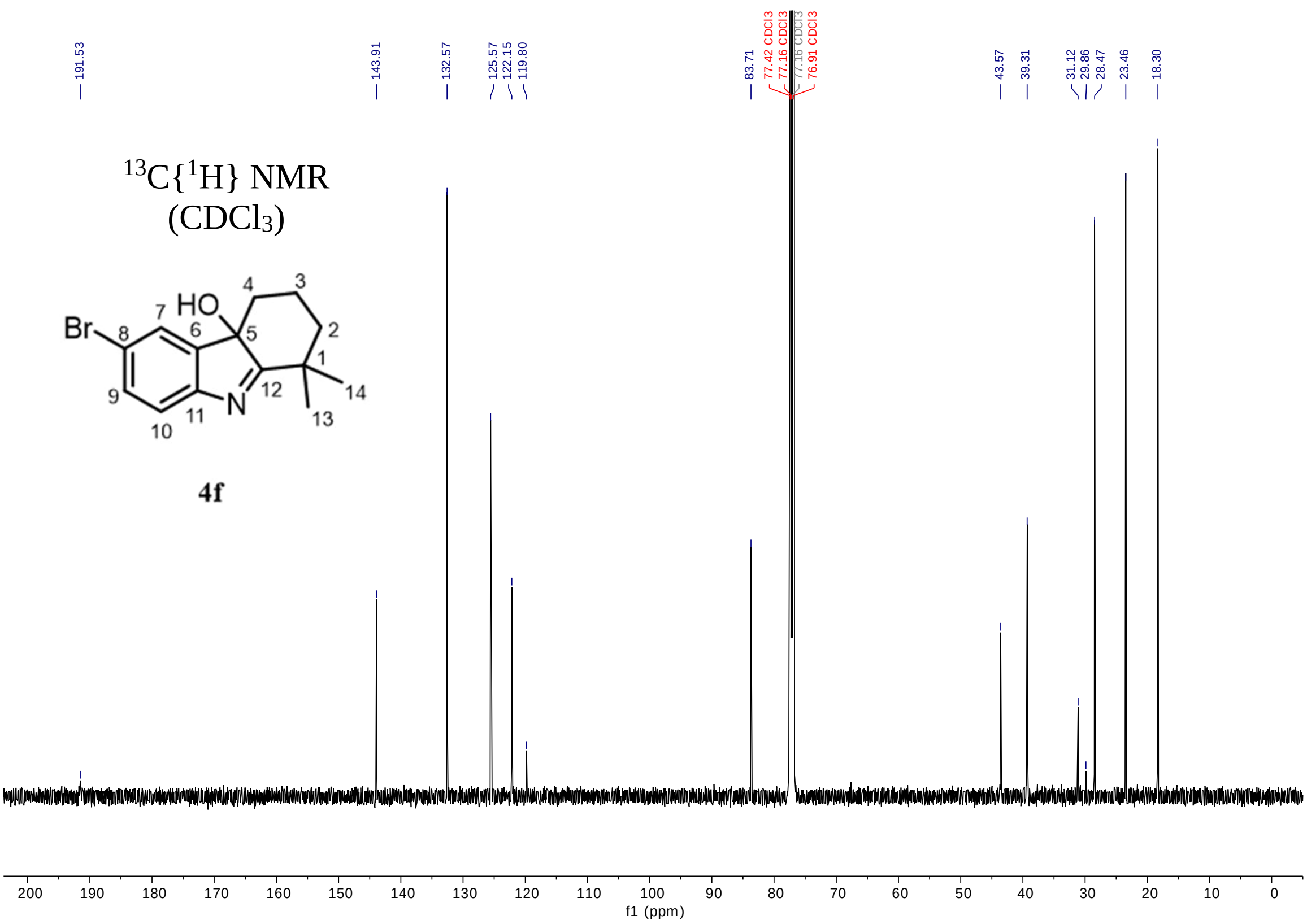

13C{1H} NMR
(CDCl3)
Br
HO
N
4f
191.53
143.91
132.57
125.57
122.15
119.80
83.71
77.42 CDCl3
77.16 CDCl3
77.16 CDCl3
76.91 CDCl3
43.57
39.31
31.12
29.86
28.47
23.46
18.30
f1 (ppm)

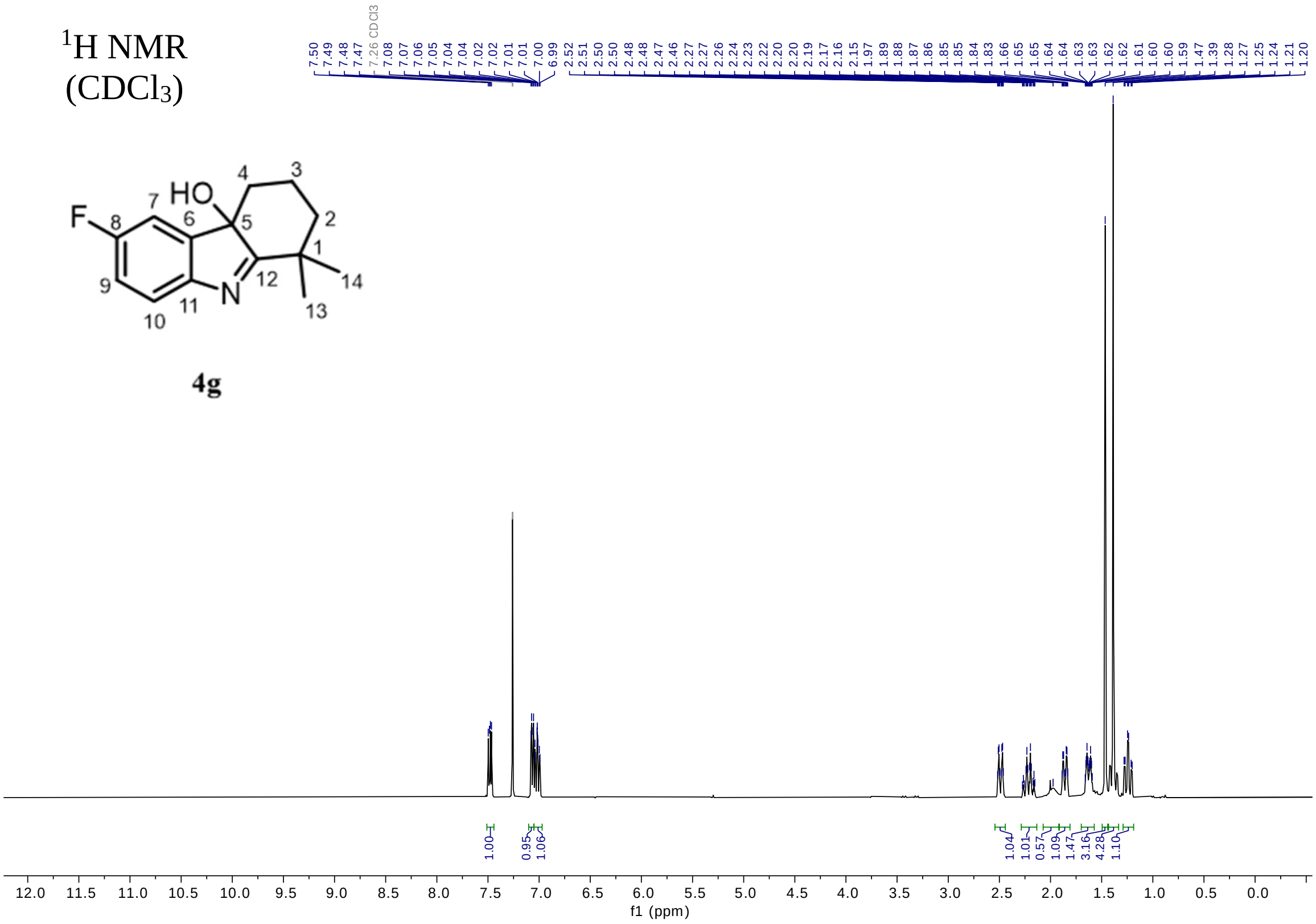
1H NMR
(CDCl3)
4g
f1 (ppm)

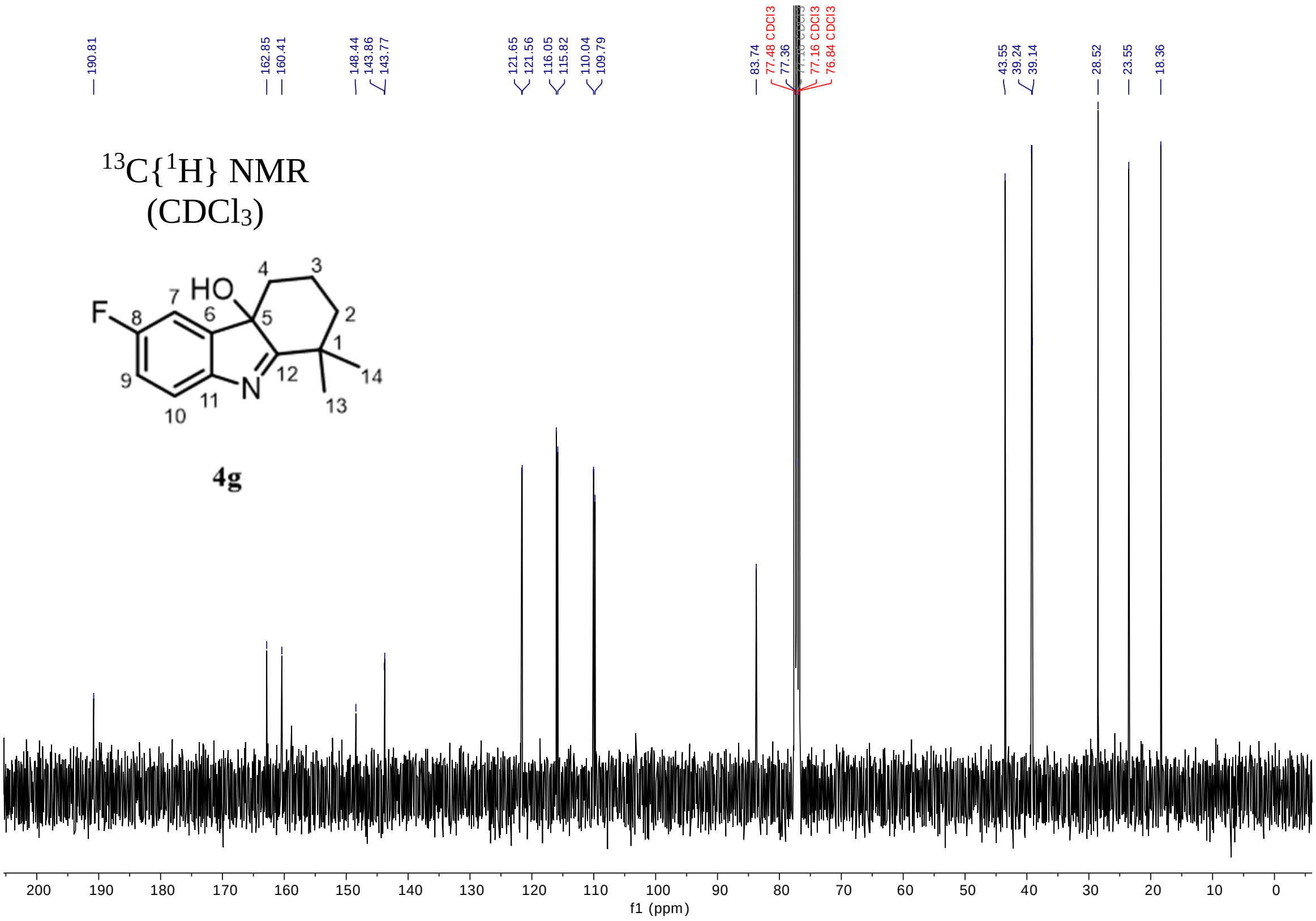
13C{1H} NMR
(CDCl3)
4g
f1 (ppm)

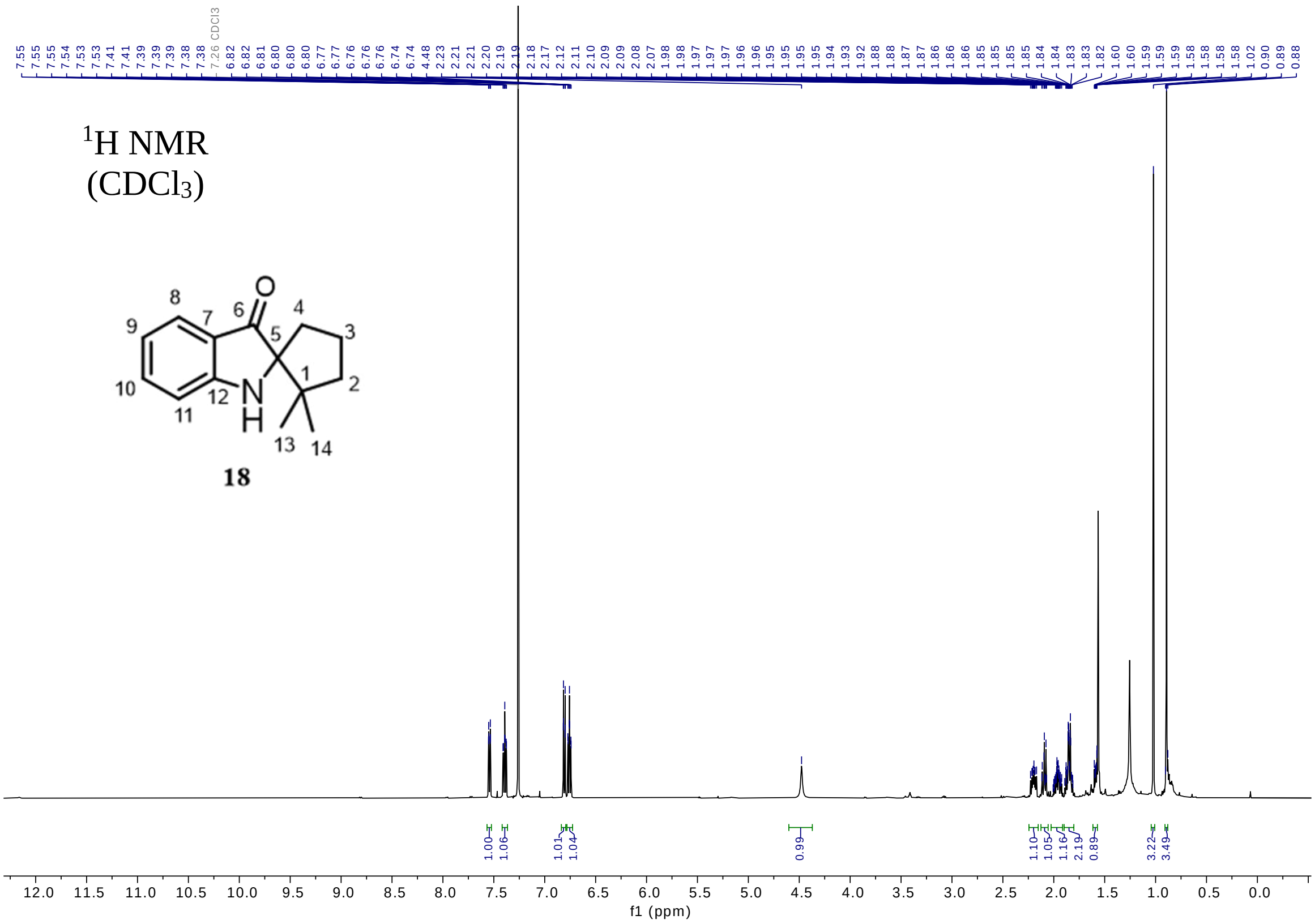
¹H NMR
(CDCl₃)
18
f1 (ppm)

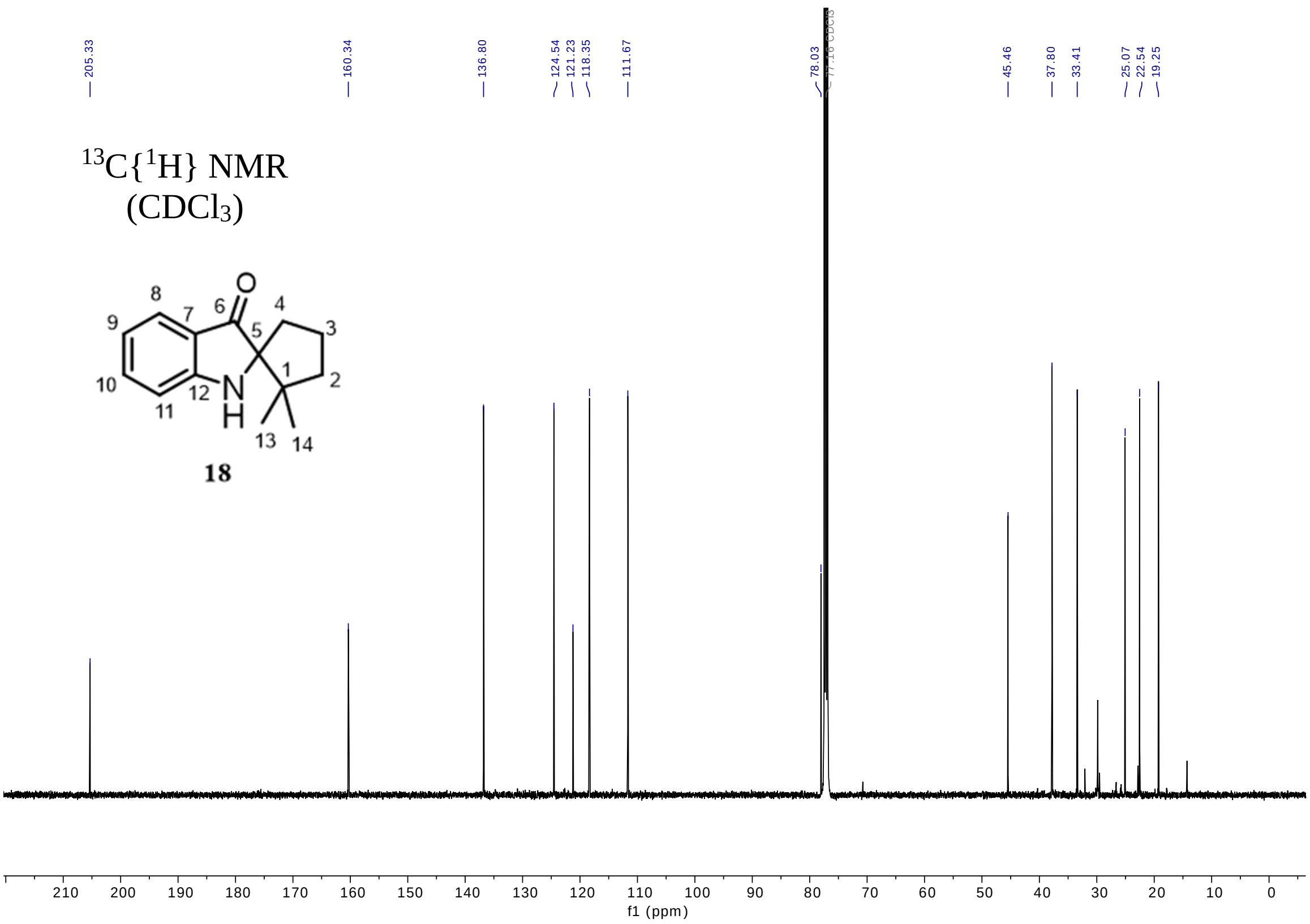
205.33
160.34
136.80
124.54
121.23
118.35
111.67
78.03
77.16 CDCl3
45.46
37.80
33.41
25.07
22.54
19.25
¹³C{¹H} NMR
(CDCl₃)
18
f1 (ppm)

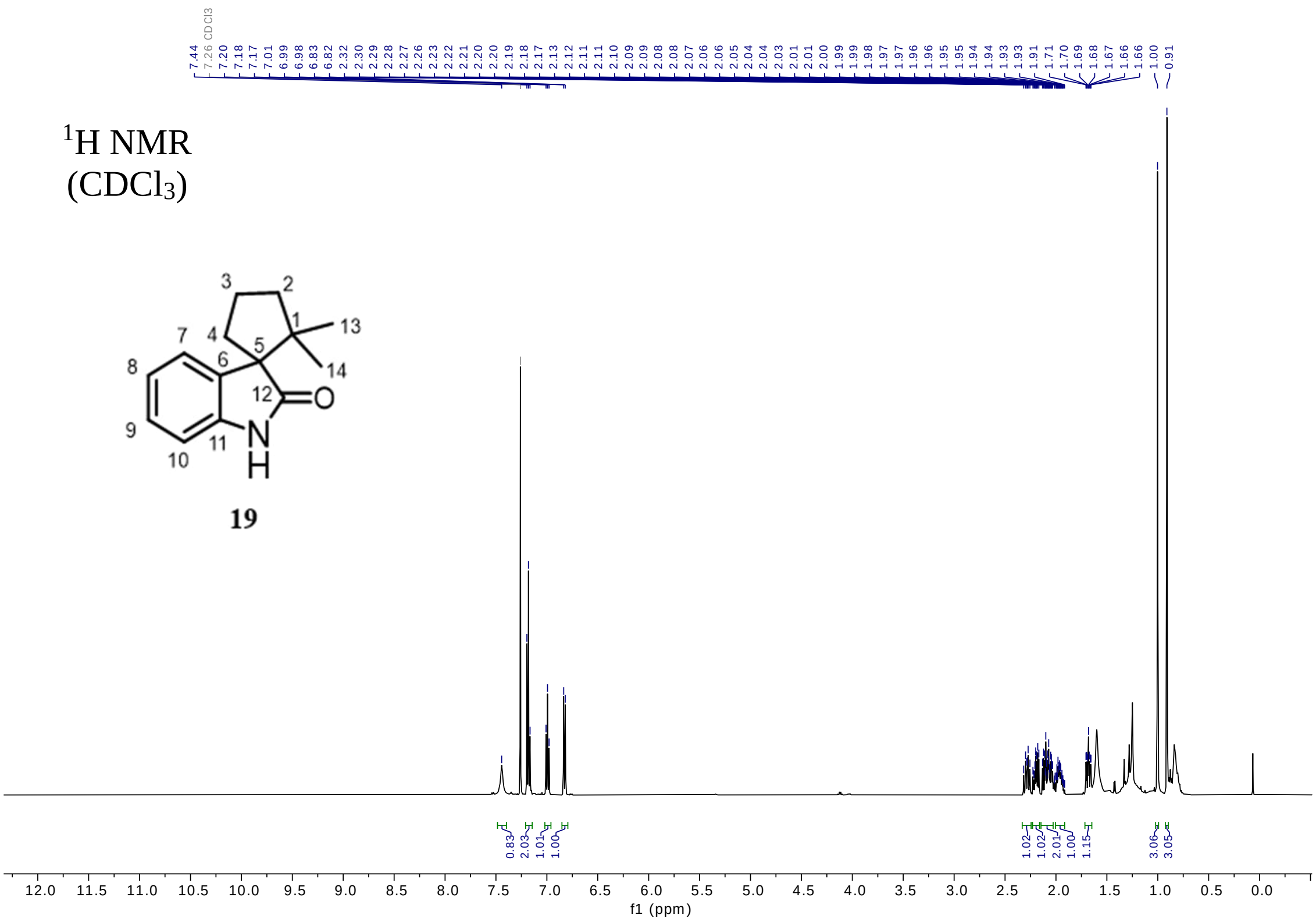
7.44
7.26 CDCl3
7.20
7.18
7.17
7.01
6.99
6.98
6.83
6.82
2.32
2.30
2.29
2.28
2.27
2.26
2.23
2.22
2.21
2.20
2.20
2.19
2.18
2.17
2.13
2.12
2.11
2.11
2.10
2.09
2.09
2.08
2.08
2.07
2.06
2.06
2.05
2.04
2.04
2.03
2.01
2.01
2.00
1.99
1.99
1.98
1.97
1.97
1.96
1.96
1.95
1.95
1.94
1.94
1.93
1.93
1.91
1.71
1.70
1.69
1.68
1.67
1.66
1.00
0.91
1H NMR
(CDCl3)
19
0.83
2.03
1.01
1.00
1.02
1.02
2.01
1.00
1.15
3.06
3.05
f1 (ppm)

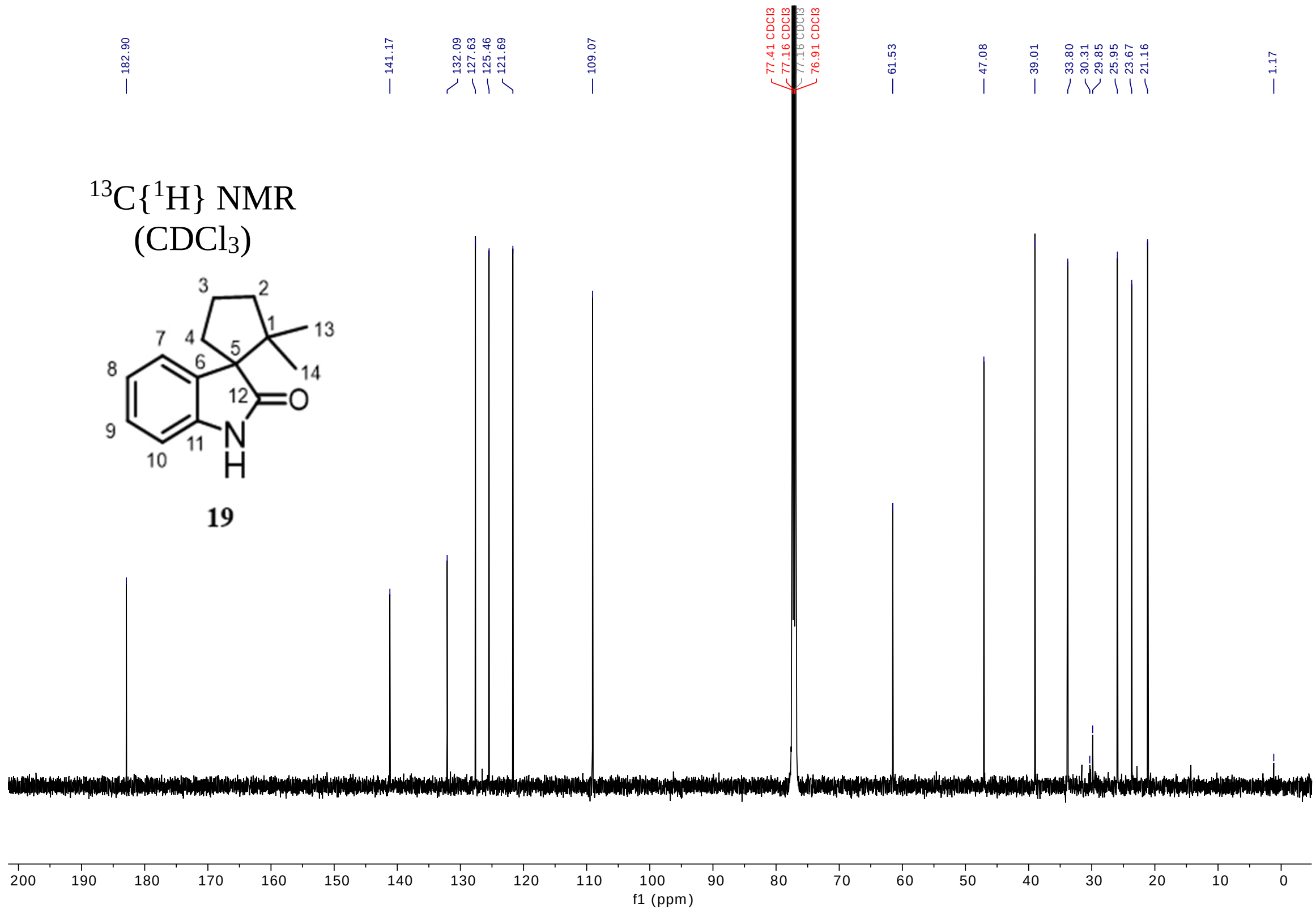
182.90
141.17
132.09
127.63
125.46
121.69
109.07
77.41 CDCl3
77.16 CDCl3
76.91 CDCl3
61.53
47.08
39.01
33.80
30.31
29.85
25.95
23.67
21.16
1.17
13C{1H} NMR
(CDCl3)
19
f1 (ppm)

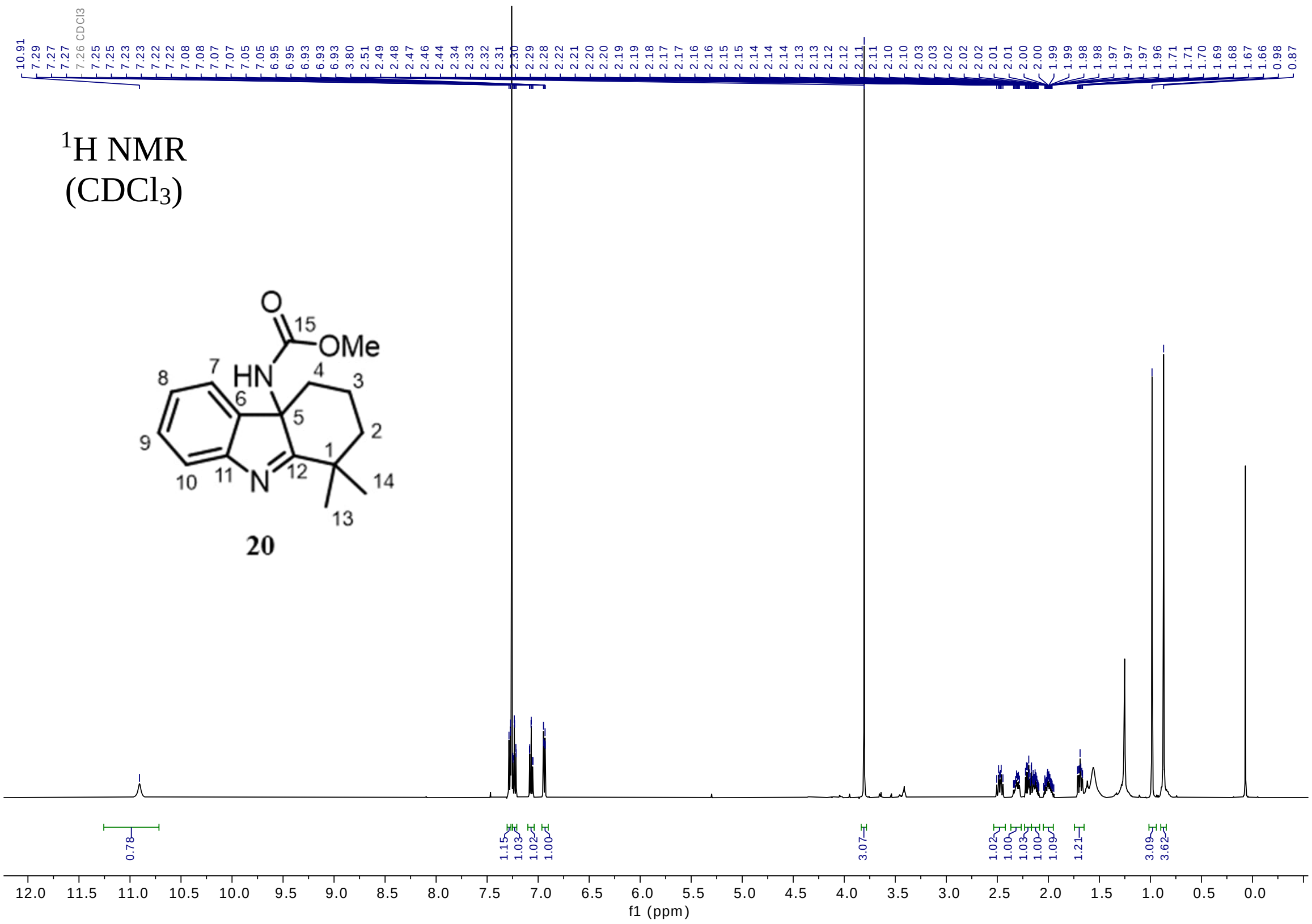
10.91 7.29 7.27 7.27 7.26 CDCl3 7.25 7.25 7.23 7.23 7.22 7.22 7.08 7.08 7.07 7.07 7.05 7.05 6.95 6.95 6.93 6.93 6.93 3.80 2.51 2.49 2.48 2.47 2.46 2.44 2.34 2.33 2.32 2.31 2.30 2.29 2.28 2.22 2.21 2.20 2.20 2.19 2.19 2.18 2.17 2.17 2.16 2.15 2.15 2.14 2.14 2.14 2.13 2.13 2.12 2.12 2.11 2.11 2.10 2.10 2.03 2.03 2.02 2.02 2.02 2.01 2.01 2.00 2.00 1.99 1.99 1.98 1.98 1.97 1.97 1.97 1.96 1.71 1.71 1.70 1.69 1.68 1.67 1.66 0.98 0.87
^{1}H NMR
$(CDCl_3)$
O
15
OMe
HN
4
3
8
7
6
5
2
9
1
10
11
N
12
14
13
20
0.78
1.15
1.03
1.02
1.00
3.07
1.02
1.00
1.03
1.00
1.09
1.21
3.09
3.62
12.0 11.5 11.0 10.5 10.0 9.5 9.0 8.5 8.0 7.5 7.0 6.5 6.0 5.5 5.0 4.5 4.0 3.5 3.0 2.5 2.0 1.5 1.0 0.5 0.0
f1 (ppm)

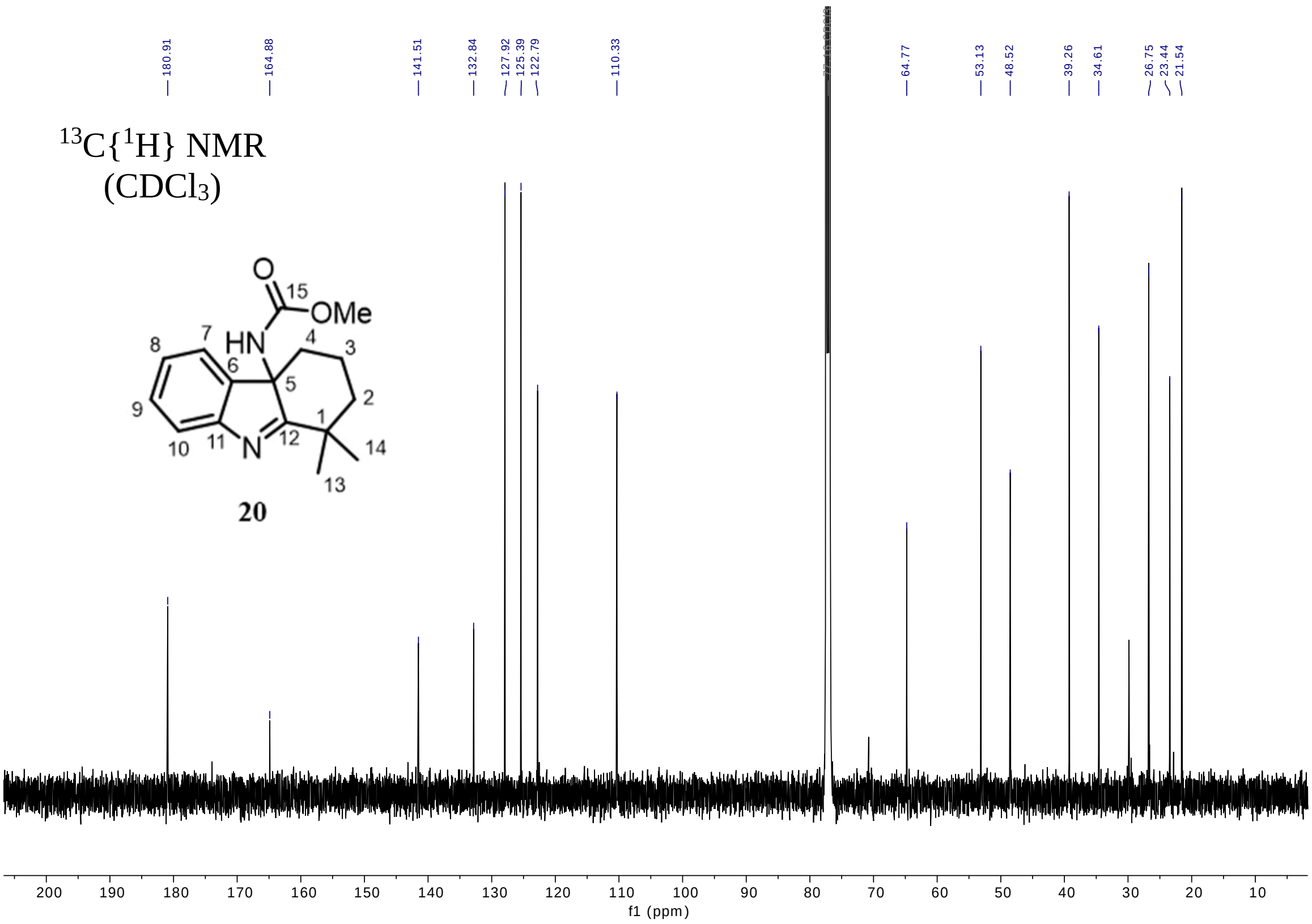
180.91
164.88
141.51
132.84
127.92
125.39
122.79
110.33
64.77
53.13
48.52
39.26
34.61
26.75
23.44
21.54
$^{13}C\{^{1}H\}$ NMR
$(CDCl_3)$
O
15
OMe
HN
4
3
8
7
6
5
2
9
1
10
11
N
12
14
13
20
200 190 180 170 160 150 140 130 120 110 100 90 80 70 60 50 40 30 20 10
f1 (ppm)

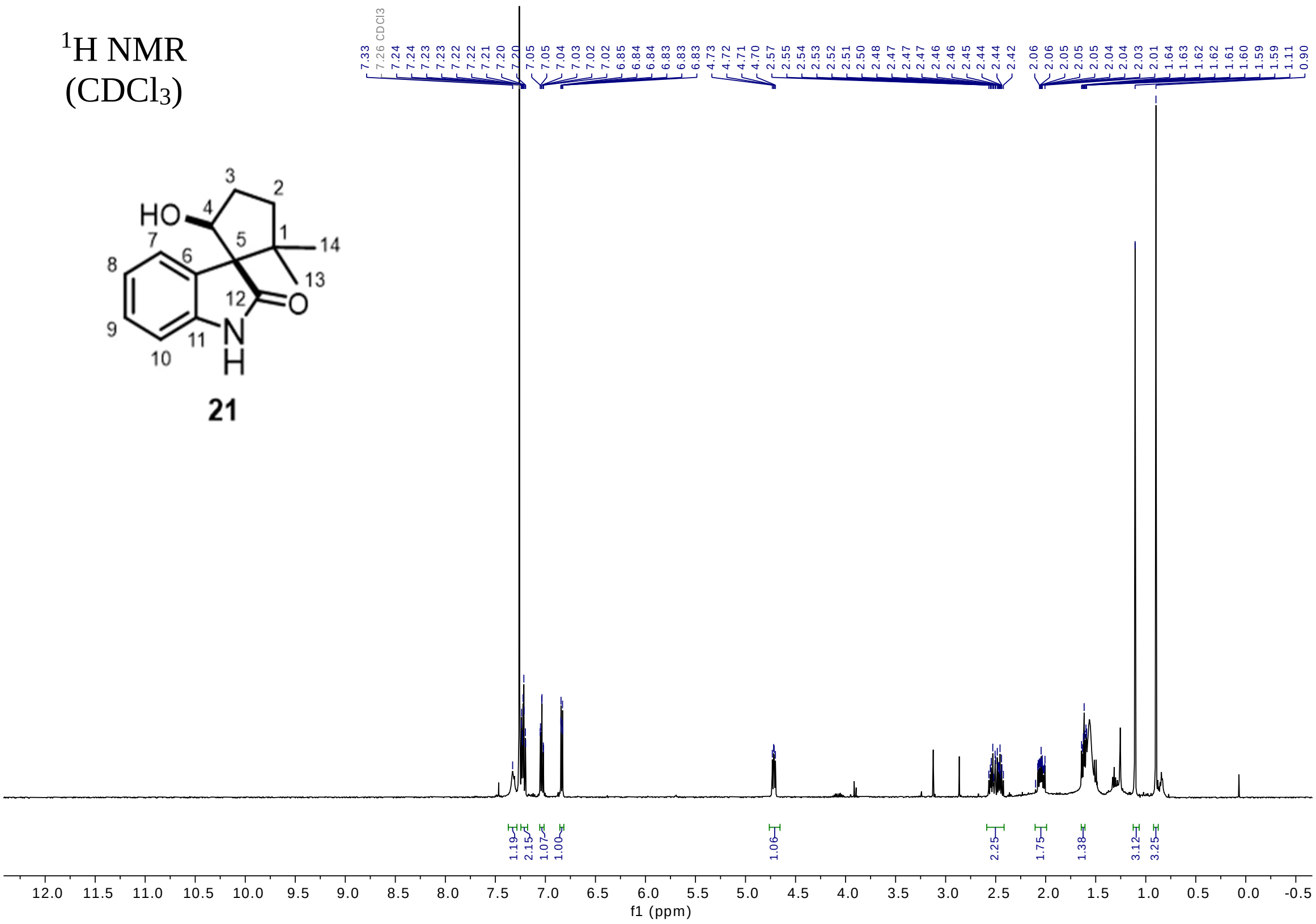

^{1}H NMR
($CDCl_3$)
21
f1 (ppm)

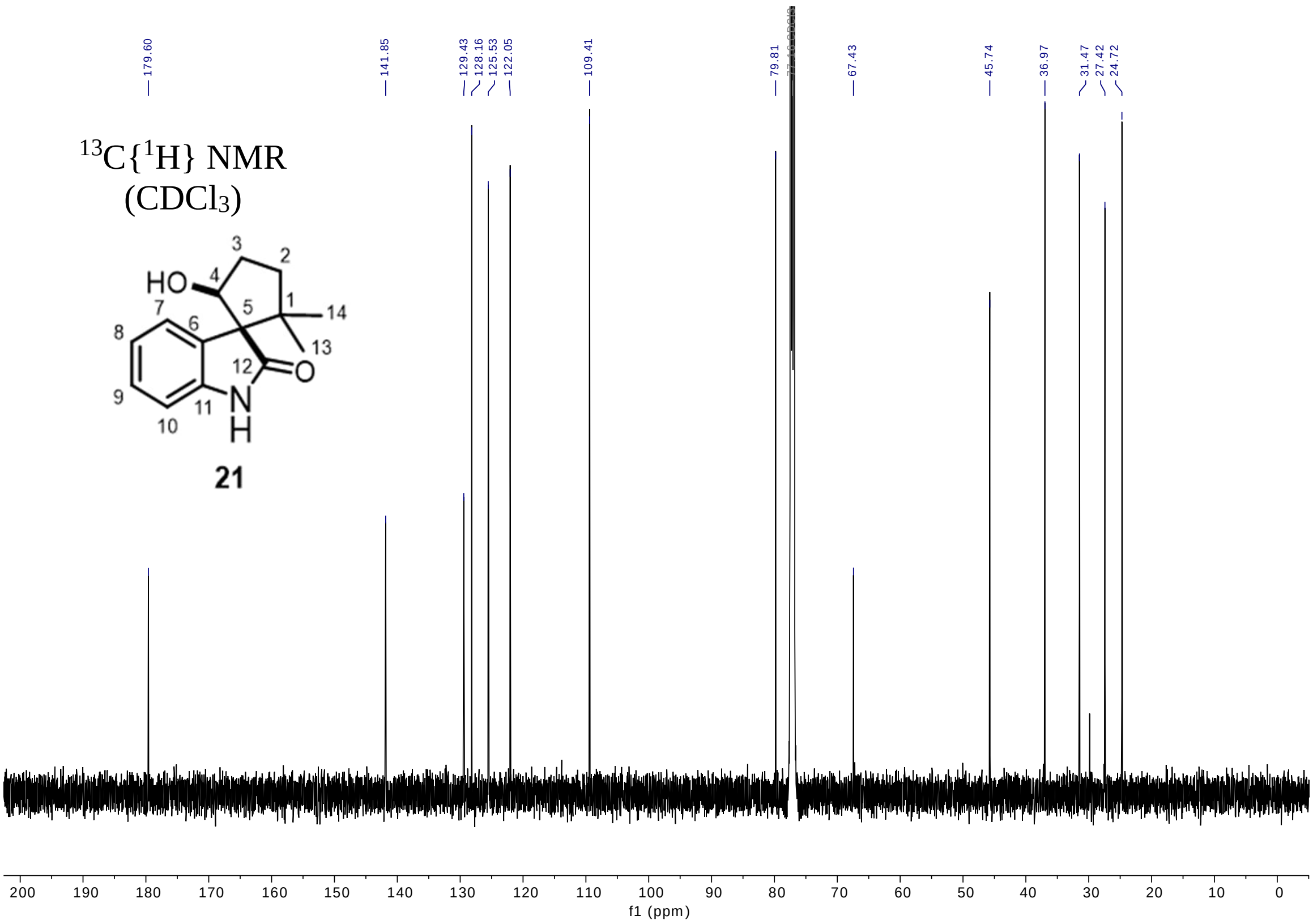

$^{13}C\{^{1}H\}$ NMR
($CDCl_3$)
21
179.60
141.85
129.43
128.16
125.53
122.05
109.41
79.81
67.43
45.74
36.97
31.47
27.42
24.72
f1 (ppm)

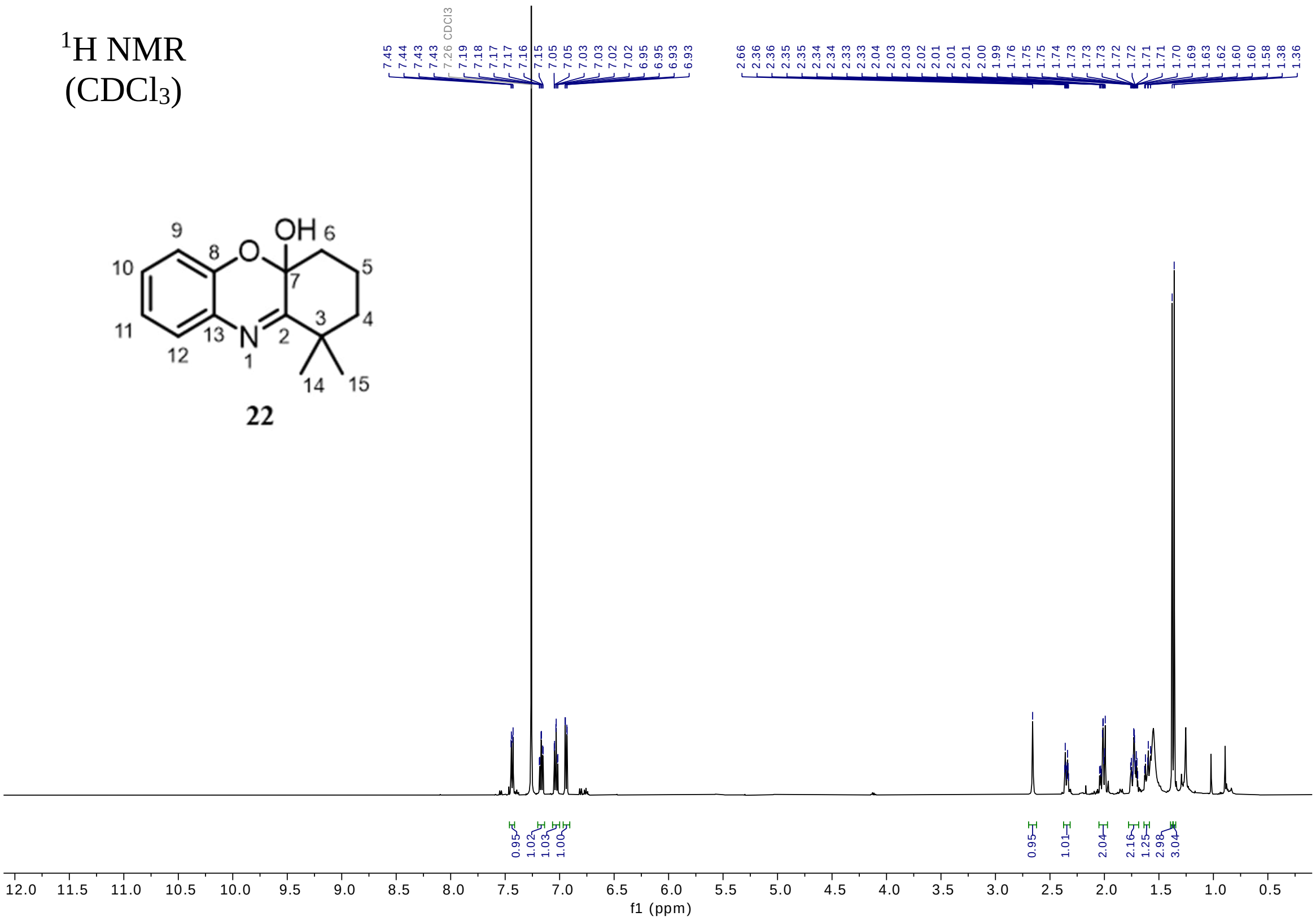

^{1}H NMR
($CDCl_3$)
22
f1 (ppm)

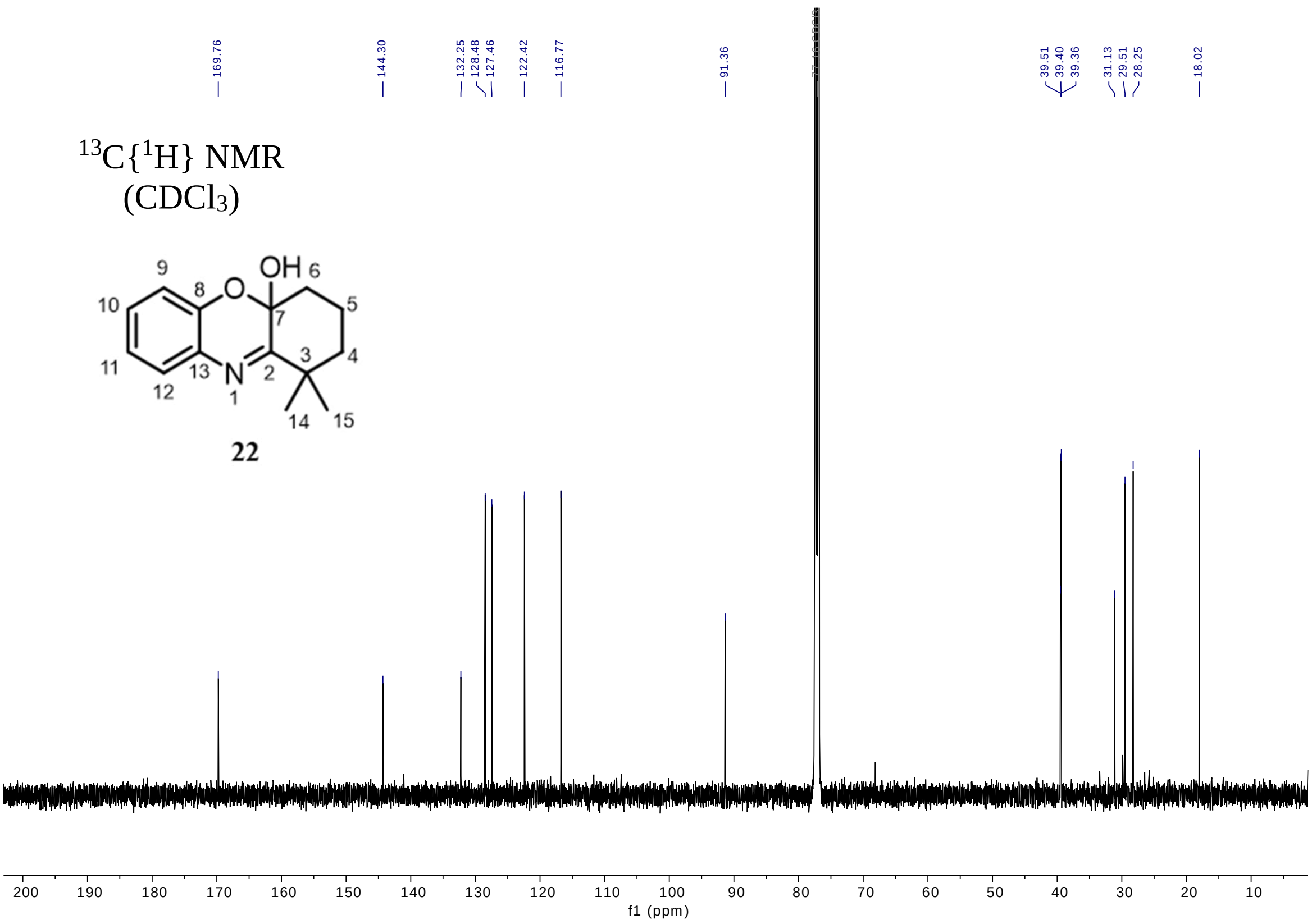

^{13}C{^{1}H} NMR
($CDCl_3$)
22
f1 (ppm)

^{1}H NMR
($CDCl_3$)

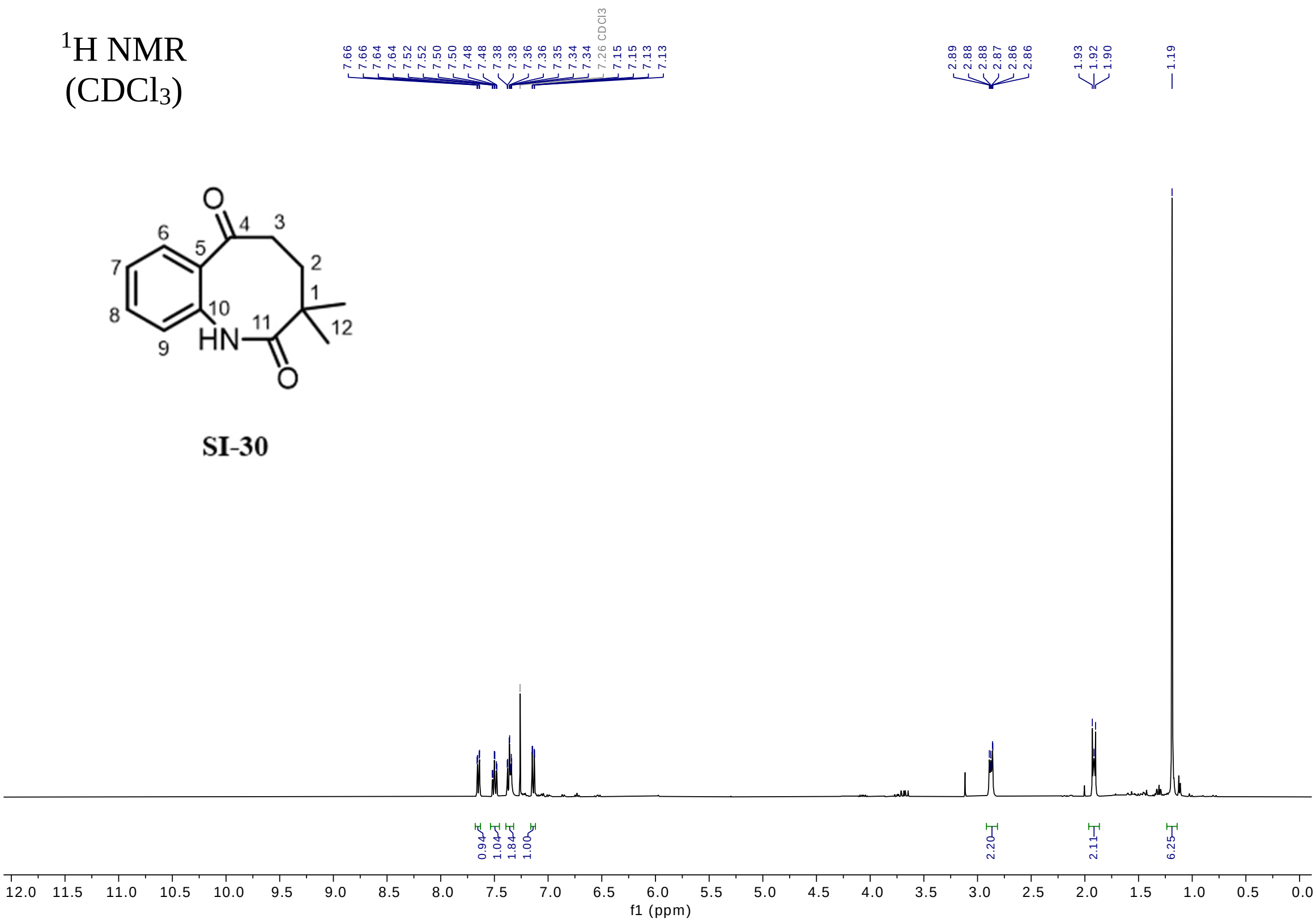

^{13}C{^{1}H} NMR
($CDCl_3$)

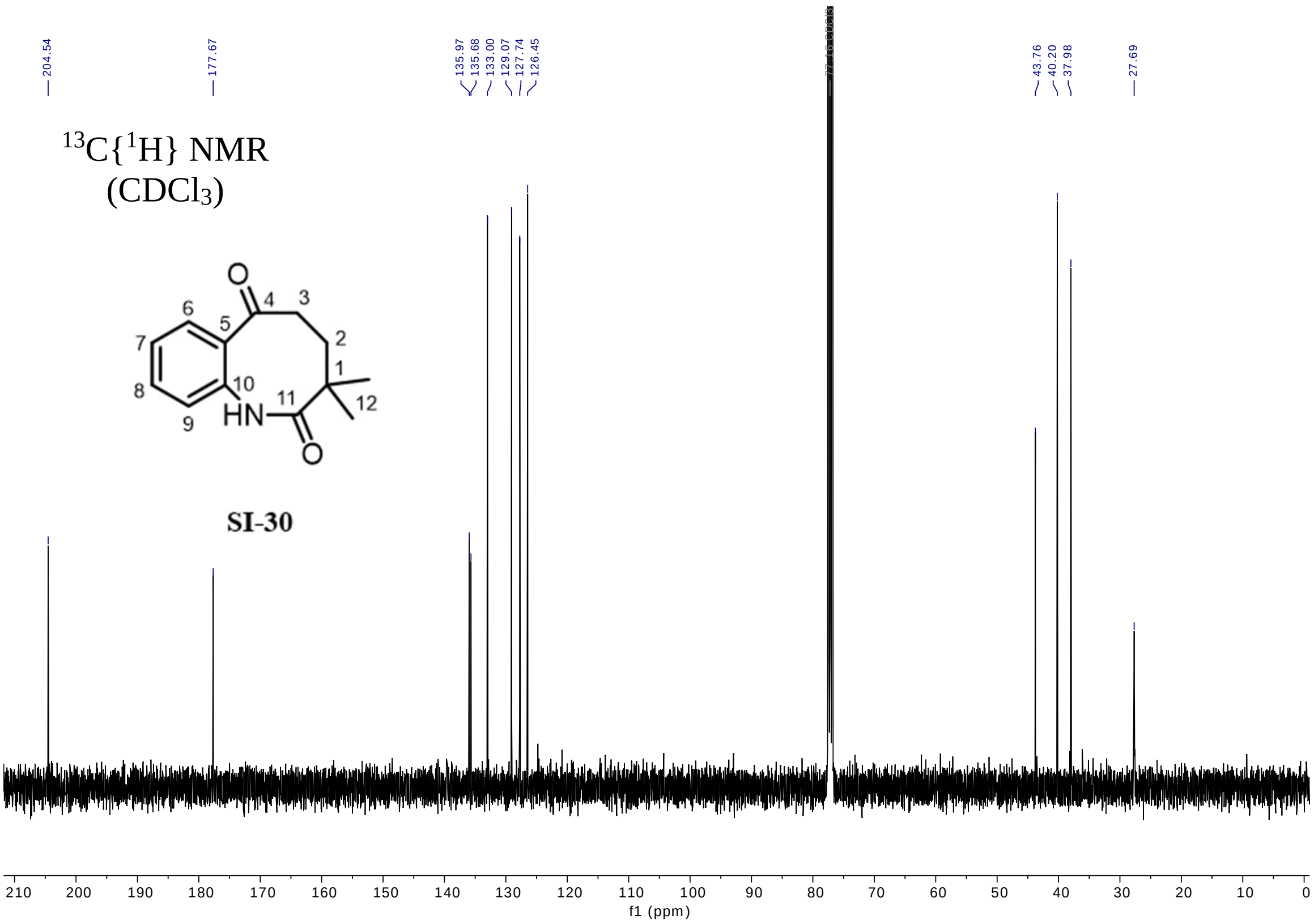

10 References

References

(1) Fulmer, G. R.; Miller, A. J. M.; Sherden, N. H.; Gottlieb, H. E.; Nudelman, A.; Stoltz, B. M.; Bercaw, J. E.; Goldberg, K. I. NMR Chemical Shifts of Trace Impurities: Common Laboratory Solvents, Organics, and Gases in Deuterated Solvents Relevant to the Organometallic Chemist. *Organometallics* **2010**, *29* (9), 2176–2179. DOI: 10.1021/om100106e.

(2) Hansen, C. L.; Ohm, R. G.; Olsen, L. B.; Ascic, E.; Tanner, D.; Nielsen, T. E. Catalytic Enantioselective Synthesis of Tetrahydocarbazoles and Exocyclic Pictet-Spengler-Type Reactions. *Org. Lett.* **2016**, *18* (23), 5990–5993. DOI: 10.1021/acs.orglett.6b02718.

(3) Nanda, L. N.; Rangari, V. A. TfOH catalyzed synthesis of 1-substituted tetrahydrocarbazoles. *Tetrahedron Lett.* **2018**, *59* (33), 3194–3197. DOI: 10.1016/j.tetlet.2018.07.023.

(4) Youn, S. W.; Pastine, S. J.; Sames, D. Ru(III)-catalyzed cyclization of arene-alkene substrates via intramolecular electrophilic hydroarylation. *Org. Lett.* **2004**, *6* (4), 581–584. DOI: 10.1021/ol036385i.

(5) Cernijenko, A.; Risgaard, R.; Baran, P. S. 11-Step Total Synthesis of (-)-Maoecrystal V. *J. Am. Chem. Soc.* **2016**, *138* (30), 9425–9428. DOI: 10.1021/jacs.6b06623.

(6) Lin, Y.; Ye, J.; Zhang, W.; Gao, Y.; Chen, H. Catalytic Oxidative Coupling Cyclization for Construction of Benzofuroindolenines under Mild Reaction Conditions. *Adv. Synth. Catal.* **2019**, *361* (3), 432–435. DOI: 10.1002/adsc.201801184.

(7) Maier, A. F. G.; Tussing, S.; Schneider, T.; Flörke, U.; Qu, Z.-W.; Grimme, S.; Paradies, J. Frustrated Lewis Pair Catalyzed Dehydrogenative Oxidation of Indolines and Other Heterocycles. *Angew. Chem. Int. Ed.* **2016**, *55* (40), 12219–12223. DOI: 10.1002/anie.201606426.

(8) Liu, C.; Han, X.; Wang, X.; Widenhoefer, R. A. Platinum-catalyzed intramolecular alkylation of indoles with unactivated olefins. *J. Am. Chem. Soc.* **2004**, *126* (12), 3700–3701. DOI: 10.1021/ja031814t.

(9) Bergstad, K.; Bäckvall, J.-E. Mild and Efficient Flavin-Catalyzed H 2 O 2 Oxidation of Tertiary Amines to Amine N -Oxides. *J. Org. Chem.* **1998**, *63* (19), 6650–6655. DOI: 10.1021/jo980926d.

(10) Chen, S.; Foss, F. W. Aerobic organocatalytic oxidation of aryl aldehydes: flavin catalyst turnover by Hantzsch's ester. *Org. Lett.* **2012**, *14* (19), 5150–5153. DOI: 10.1021/ol302479b.

(11) Ménová, P.; Dvořáková, H.; Eigner, V.; Ludvík, J.; Cibulka, R. Electron-Deficient Alloxazinium Salts: Efficient Organocatalysts of Mild and Chemoselective Sulfoxidations with Hydrogen Peroxide. *Adv. Synth. Catal.* **2013**, *355* (17), 3451–3462. DOI: 10.1002/adsc.201300617.

(12) Hartman, T.; Reisnerová, M.; Chudoba, J.; Svobodová, E.; Archipowa, N.; Kutta, R. J.; Cibulka, R. Photocatalytic Oxidative 2+2 Cycloelimination Reactions with Flavinium Salts: Mechanistic Study and Influence of the Catalyst Structure. *ChemPlusChem* **2021**, *86* (3), 373–386. DOI: 10.1002/cplu.202000767.

(13) Muhr, M.; Heiß, P.; Schütz, M.; Bühler, R.; Gemel, C.; Linden, M. H.; Linden, H. B.; Fischer, R. A. Enabling LIFDI-MS measurements of highly air sensitive organometallic compounds: a combined MS/glovebox technique. *Dalton Trans.* **2021**, *50* (26), 9031–9036. DOI: 10.1039/D1DT00978H.

(14) Ménová, P.; Eigner, V.; Čejka, J.; Dvořáková, H.; Šanda, M.; Cibulka, R. Synthesis and structural studies of flavin and alloxazine adducts with O-nucleophiles. *J. Mol. Struct.* **2011**, *1004* (1), 178–187. DOI: 10.1016/j.molstruc.2011.08.002.

(15) Yoneda, F.; Sakuma, Y.; Ichiba, M.; Shinomura, K. Syntheses of isoalloxazines and isoalloxazine 5-oxides. A new synthesis of riboflavin. *J. Am. Chem. Soc.* **1976**, *98* (3), 830–835. DOI: 10.1021/ja00419a034.

(16) Imada, Y.; Iida, H.; Ono, S.; Masui, Y.; Murahashi, S.-I. Flavin-catalyzed oxidation of amines and sulfides with molecular oxygen: biomimetic green oxidation. *Chem. Asian J.* **2006**, *1* (1-2), 136–147. DOI: 10.1002/asia.200600080.

(17) Ménová, P.; Cibulka, R. Insight into the catalytic activity of alloxazinium and isoalloxazinium salts in the oxidations of sulfides and amines with hydrogen peroxide. *J. Mol. Catal. A Chem.* **2012**, *363-364*, 362–370. DOI: 10.1016/j.molcata.2012.07.012.

(18) Kemal, C.; Bruice, T. C. Simple synthesis of a 4a-hydroperoxy adduct of a 1,5-dihydroflavine: preliminary studies of a model for bacterial luciferase. *Proc. Natl. Acad. Sci. U.S.A.* **1976**, *73* (4), 995–999. DOI: 10.1073/pnas.73.4.995.

(19) Yang, Z.; Swanson, C. R. B.; Unsworth, W. P. A Consecutive Ring-Expansion Strategy towards the Macrocyclic Core of the Solomonamide Natural Products. *Synlett* **2023**, *34* (14), 1694–1698. DOI: 10.1055/a-1932-9717.
(20) Shi, J.; Wang, R.-A.; Wu, W.; Song, J.-R.; Chi, Q.; Pan, W.-D.; Ren, H. Copper-Catalyzed Aerobic Selective Oxidation of Tetrahydrocarbolines. *Org. Lett.* **2022**, *24* (18), 3358–3362. DOI: 10.1021/acs.orglett.2c01059.
(21) Takemoto, M.; Iwakiri, Y.; Suzuki, Y.; Tanaka, K. A mild procedure for the oxidative cleavage of substituted indoles catalyzed by plant cell cultures. *Tetrahedron Lett.* **2004**, *45* (43), 8061–8064. DOI: 10.1016/j.tetlet.2004.08.175.
(22) Ding, W.; Zhou, Q.-Q.; Xuan, J.; Li, T.-R.; Lu, L.-Q.; Xiao, W.-J. Photocatalytic aerobic oxidation/semipinacol rearrangement sequence: a concise route to the core of pseudoindoxyl alkaloids. *Tetrahedron Lett.* **2014**, *55* (33), 4648–4652. DOI: 10.1016/j.tetlet.2014.06.102.
(23) The MathWorks Inc. *MATLAB*; The MathWorks Inc., 2023. https://www.mathworks.com/.
(24) Stoll, S.; Schweiger, A. EasySpin, a comprehensive software package for spectral simulation and analysis in EPR. *J. Magn. Reson.* **2006**, *178* (1), 42–55. DOI: 10.1016/j.jmr.2005.08.013.
(25) Frisch, M. J.; Trucks, G. W.; Schlegel, H. B.; Scuseria, G. E.; Robb, M. A.; Cheeseman, J. R.; Scalmani, G.; Barone, V.; Petersson, G. A.; Nakatsuji, H.; Li, X.; Caricato, M.; Marenich, A. V.; Bloino, J.; Janesko, B. G.; Gomperts, R.; Mennucci, B.; Hratchian, H. P.; Ortiz, J. V.; Izmaylov, A. F.; Sonnenberg, J. L.; Williams-Young, D.; Ding, F.; Lipparini, F.; Egidi, F.; Goings, J.; Peng, B.; Petrone, A.; Henderson, T.; Ranasinghe, D.; Zakrzewski, V. G.; J. Gao, N. R.; Zheng, G.; Liang, W.; Hada, M.; Ehara, M.; Toyota, K.; Fukuda, R.; Hasegawa, J.; Ishida, M.; Nakajima, T.; Honda, Y.; Kitao, O.; Nakai, H.; Vreven, T.; Throssell, K.; J. A. Montgomery, JR.; Peralta, J. E.; Ogliaro, F.; Bearpark, M. J.; Heyd, J. J.; Brothers, E. N.; Kudin, K. N.; Staroverov, V. N.; Keith, T. A.; Kobayashi, R.; Normand, J.; Raghavachari, K.; Rendell, A. P.; Burant, J. C.; Iyengar, S. S.; Tomasi, J.; Cossi, M.; Millam, J. M.; Klene, M.; Adamo, C.; Cammi, R.; Ochterski, J. W.; Martin, R. L.; Morokuma, K.; Farkas, O.; Foresman, J. B.; Fox, D. J. *Gaussian 16, Revision C.01*; Gaussian, Inc., 2019.
(26) Stephens, P. J.; Devlin, F. J.; Chabalowski, C. F.; Frisch, M. J. Ab Initio Calculation of Vibrational Absorption and Circular Dichroism Spectra Using Density Functional Force Fields. *J. Phys. Chem.* **1994**, *98* (45), 11623–11627. DOI: 10.1021/j100096a001.

(27) Becke, A. D. Density-functional thermochemistry. III. The role of exact exchange. *J. Chem. Phys.* **1993**, *98* (7), 5648–5652. DOI: 10.1063/1.464913.

(28) Lee, C.; Yang, W.; Parr, R. G. Development of the Colle-Salvetti correlation-energy formula into a functional of the electron density. *Phys. Rev. B* **1988**, *37* (2), 785–789.

(29) Zhao, Y.; Truhlar, D. G. The M06 suite of density functionals for main group thermochemistry, thermochemical kinetics, noncovalent interactions, excited states, and transition elements: two new functionals and systematic testing of four M06-class functionals and 12 other functionals. *Theor. Chem. Acc.* **2008**, *120* (1), 215–241. DOI: 10.1007/s00214-007-0310-x.

(30) Grimme, S.; Ehrlich, S.; Goerigk, L. Effect of the damping function in dispersion corrected density functional theory. *J. Comput. Chem.* **2011**, *32* (7), 1456–1465. DOI: 10.1002/jcc.21759.

(31) Weigend, F. Accurate Coulomb-fitting basis sets for H to Rn. *Phys. Chem. Chem. Phys.* **2006**, *8* (9), 1057–1065. DOI: 10.1039/B515623H.

(32) Weigend, F.; Ahlrichs, R. Balanced basis sets of split valence, triple zeta valence and quadruple zeta valence quality for H to Rn: Design and assessment of accuracy. *Phys. Chem. Chem. Phys.* **2005**, *7* (18), 3297–3305. DOI: 10.1039/B508541A.

(33) Dunning, T. H. Gaussian basis sets for use in correlated molecular calculations. I. The atoms boron through neon and hydrogen. *J. Chem. Phys.* **1989**, *90* (2), 1007–1023. DOI: 10.1063/1.456153.

(34) Kendall, R. A.; Dunning, T. H.; Harrison, R. J. Electron affinities of the first-row atoms revisited. Systematic basis sets and wave functions. *J. Chem. Phys.* **1992**, *96* (9), 6796–6806. DOI: 10.1063/1.462569.

(35) Woon, D. E.; Dunning, T. H. Gaussian basis sets for use in correlated molecular calculations. III. The atoms aluminum through argon. *J. Chem. Phys.* **1993**, *98* (2), 1358–1371. DOI: 10.1063/1.464303.

(36) Davidson, E. R. Comment on “Comment on Dunning's correlation-consistent basis sets”. *Chem. Phys. Lett.* **1996**, *260* (3-4), 514–518. DOI: 10.1016/0009-2614(96)00917-7.

(37) Tomasi, J.; Mennucci, B.; Cammi, R. Quantum Mechanical Continuum Solvation Models. *Chem. Rev.* **2005**, *105* (8), 2999–3094. DOI: 10.1021/cr9904009.

(38) D. P. Chong, Ed. *Recent Advances in Density Functional Methods, Part I*; World Scientific Publ. Co., 1996.

(39) Meng, E. C.; Goddard, T. D.; Pettersen, E. F.; Couch, G. S.; Pearson, Z. J.; Morris, J. H.; Ferrin, T. E. UCSF ChimeraX: Tools for structure building and analysis. *Protein Sci.* **2023**, *32* (11), e4792. DOI: 10.1002/pro.4792.

(40) Ingman, V. M.; Schaefer, A. J.; Andreola, L. R.; Wheeler, S. E. QChASM : Quantum chemistry automation and structure manipulation. *WIREs Comput. Mol. Sci.* **2021**, *11* (4), e1510. DOI: 10.1002/wcms.1510.

(41) Schaefer, A. J.; Ingman, V. M.; Wheeler, S. E. SEQCROW: A ChimeraX bundle to facilitate quantum chemical applications to complex molecular systems. *J. Comput. Chem.* **2021**, *42* (24), 1750–1754. DOI: 10.1002/jcc.26700.

(42) Bruker. *APEX 4*:*Crystallography data collection software*, 2022.

(43) Bruker. *SAINT*:*Crystallography data integration software*, 2019.

(44) Bruker. *SADABS*:*Absorption correction software for crystallography*, 2016.

(45) Sheldrick, G. M. SHELXT - integrated space-group and crystal-structure determination. *Acta. Cryst. A* **2015**, *71* (Pt 1), 3–8. DOI: 10.1107/S2053273314026370.

(46) Sheldrick, G. Crystal structure refinement with SHELXL. *Acta Cryst. C* **2015**, *71* (1), 3–8. DOI: 10.1107/S2053229614024218.

(47) Hübschle, C. B.; Sheldrick, G. M.; Dittrich, B. ShelXle: a Qt graphical user interface for SHELXL. *J. Appl. Crystallogr.* **2011**, *44* (Pt 6), 1281–1284. DOI: 10.1107/S0021889811043202.

(48) Prince, E.; Fuess, H.; Hahn, T.; Wondratschek, H.; Müller, U.; Shmueli, U.; Authier, A.; Kopský, V.; Litvin, D. B.; Rossmann, M. G.; Arnold, E.; Hall, S.; McMahon, B. *International Tables for Crystallography*, C; International Union of Crystallography, 2006. DOI: 10.1107/97809553602060000103.

(49) Macrae, C. F.; Edgington, P. R.; McCabe, P.; Pidcock, E.; Shields, G. P.; Taylor, R.; Towler, M.; van de Streek, J. Mercury : visualization and analysis of crystal structures. *J. Appl. Crystallogr.* **2006**, *39* (3), 453–457. DOI: 10.1107/S002188980600731X.

(50) Macrae, C. F.; Sovago, I.; Cottrell, S. J.; Galek, P. T. A.; McCabe, P.; Pidcock, E.; Platings, M.; Shields, G. P.; Stevens, J. S.; Towler, M.; Wood, P. A. Mercury 4.0: from visualization to analysis, design and prediction. *J. Appl. Crystallogr.* **2020**, *53* (Pt 1), 226–235. DOI: 10.1107/S1600576719014092.
